# Comparative analyses of genotype dependent expressed sequence tags and stress-responsive transcriptome of chickpea wilt illustrate predicted and unexpected genes and novel regulators of plant immunity

**DOI:** 10.1186/1471-2164-10-415

**Published:** 2009-09-05

**Authors:** Nasheeman Ashraf, Deepali Ghai, Pranjan Barman, Swaraj Basu, Nagaraju Gangisetty, Mihir K Mandal, Niranjan Chakraborty, Asis Datta, Subhra Chakraborty

**Affiliations:** 1 National Institute of Plant Genome Research, Aruna Asaf Ali Marg, New Delhi-110067, India

## Abstract

**Background:**

The ultimate phenome of any organism is modulated by regulated transcription of many genes. Characterization of genetic makeup is thus crucial for understanding the molecular basis of phenotypic diversity, evolution and response to intra- and extra-cellular stimuli. Chickpea is the world's third most important food legume grown in over 40 countries representing all the continents. Despite its importance in plant evolution, role in human nutrition and stress adaptation, very little ESTs and differential transcriptome data is available, let alone genotype-specific gene signatures. Present study focuses on *Fusarium *wilt responsive gene expression in chickpea.

**Results:**

We report 6272 gene sequences of immune-response pathway that would provide genotype-dependent spatial information on the presence and relative abundance of each gene. The sequence assembly led to the identification of a *Ca*Unigene set of 2013 transcripts comprising of 973 contigs and 1040 singletons, two-third of which represent new chickpea genes hitherto undiscovered. We identified 209 gene families and 262 genotype-specific SNPs. Further, several novel transcription regulators were identified indicating their possible role in immune response. The transcriptomic analysis revealed 649 non-cannonical genes besides many unexpected candidates with known biochemical functions, which have never been associated with pathostress-responsive transcriptome.

**Conclusion:**

Our study establishes a comprehensive catalogue of the immune-responsive root transcriptome with insight into their identity and function. The development, detailed analysis of *Ca*EST datasets and global gene expression by microarray provide new insight into the commonality and diversity of organ-specific immune-responsive transcript signatures and their regulated expression shaping the species specificity at genotype level. This is the first report on differential transcriptome of an unsequenced genome during vascular wilt.

## Background

Living cells have evolved to perceive and integrate different signals from their surroundings and to respond by modulating the appropriate gene expression. Expressed sequence tags (ESTs) provide an invaluable resource for analysis of gene expression associated with specific organs, growth conditions, developmental processes and responses to various environmental stresses [1-3]. It bridges the gap between the genome sequences and gene function. ESTs have been useful for intra- and intergenomic comparisons, gene discovery, generation of single nucleotide polymorphisms (SNPs), cloning of genes from MStag peptide sequences, transcript pattern characterization, identifying splice variants, erroneous annotations in the genome database and incomplete prediction of gene structure [4,5]. Further, the transcriptome of cells and organs comprise a focused set of transcripts that fulfills discrete but varied cellular functions. The analyses of organ specific transcriptome provide additional information about localization of gene function and pathway compartmentalization. Whereas the transcriptome research is quite advanced in animals, yeast, bacteria and reference plants like Arabidopsis and rice [6-10] there is relatively less information in crop plants.

Legumes are valuable agricultural and commercial crops that serve as important nutrient sources for human diet and animal feed. About one third of human nutrition comes from legumes and in many developing countries, legumes serve as the only source of protein. Many secondary metabolites in legumes have been implicated in defense and are of particular interest as novel pharmaceuticals [11]. Five tribes constitute the family fabaceae, of which one representative genus each from four tribes have been used to generate ESTs [4,12,13]. However, the tribe ciceri having a single genus *Cicer*, remained as the understudied legume. Chickpea, the most important member of the genus *Cicer*, represents the world's third important pulse crop. It is grown on about 10 mha area worldwide and the global production exceeds 8 million tons [14]. In many water-deficient regions of the world, it serves as an important protein-rich food and an increasingly valuable traded commodity. Chickpea has one of the highest nutritional compositions of edible legume and does not contain any specific major anti-nutritional factor, rather it is used in herbal medicine. Despite the importance of chickpea in the study of plant evolution, its role in nutritional requirement in humans, and stress adaptation, nothing is known about the genes responsible for these traits-primarily because it is recalcitrant to genetic analysis. Unlike genetically tractable plants such as tomato, maize and *Arabidopsis*, chickpea produces a limited number of seeds. Furthermore, its genome is large (732 Mbp) as compared to *Arabidopsis *(125 Mbp). Consequently, chickpea has remained outside the realm of both modern genome-sequencing initiatives and large scale functional genomics studies. Currently available completely annotated plant genome sequences make it possible to study the genomes of agriculturally important genetically complex crop plants such as chickpea by comparing the ESTs derived from them. Only very recently, attention has been given from both genomics and proteomics perspect to this important food legume [15-19]. Because of its evolutionary position as a key node within legumes as well as its nutritional and medicinal significance to humans, chickpea is ideally suited for genomic prospecting.

Transcriptional programs that regulate development and stress response are exquisitely controlled in space and time. Elucidating these programs that underlie development is essential to understand the acquisition of cell and tissue identity. Root in higher plants is a highly organized structure that plays a key role in nutrient acquisition and water uptake besides its primary function of mechanical support to the plant. Nevertheless, it is essentially the entry point for the soil borne pathogens into the plant body. Of the soil borne root pathogen, vascular wilt is the most important disease. Vascular wilt caused by *Fusarium *is ubiquitous evolutionarily and effects crop plants across families. In particular, chickpea wilt, is widespread in occurrence and on an average causes substantial loss of 10 to 15% in production every year worldwide. During the infection process the fungus invades roots and spreads systemically through the host's vascular system, breaking down the cell walls to form gels that block the plant's transport system thereby causing yellowing and wilting symptoms. In general, the wilt symptoms appear as chlorotic spots on the lower leaves followed by discoloration and necrosis. Vascular discoloration occurs from the roots to the young stems, followed by a yellowing and wilting of the leaves before final necrosis [20]. When uprooted the stem is split vertically and internal discoloration is visible in pith and xylem [21]. The susceptible genotypes take less than 25 days for wilting whereas the resistant ones do not show any symptoms of wilting upto 60 days. In the present study, two genotypes JG-62 and WR-315 were used which are known to be susceptible and resistant to *Fusarium *wilt, respectively. The localization of the fungus was limited in the resistant cultivar while extensive colonization occurs in susceptible one by fourth to fifth day post infection. Externally the extent of mycelial colonization is manifested in the form of browning of the roots after 72 hours in the susceptible genotype while no such discoloration is evident in the resistant one.

To understand the regulatory networks and metabolic pathways governing genotype-specific cellular responses towards different signals, we have initiated ESTablishing *Ca*Unigene dataset and a genome-wide analysis of gene expression in this food legume. As a first step towards this, we have developed ESTs and stress responsive transcriptome of chickpea, as a basis for future transcriptome and proteome comparisons of genetic mutants, pathogen-infected and/or environmentally challenged plants. A total of 6272 high quality ESTs were identified, functionally annotated, computationally analysed and classified into different functional categories. Of the 2013 Unigenes, 807 were identified as previously uncharacterized. In addition, we have developed a cDNA based microarray chip and examined the global state of gene expression in the chickpea root during vascular wilt to identify the potential innate immune responsive candidates involved in the complex regulatory network that may function in this organ. These transcriptional signatures often predict previously unknown cellular functions. ESTablished set of pathostress-responsive organ-specific unigenes and the putative differentially expressed genes identified from the present study will provide a foundation for future investigation of the expression and function of the genes and proteins to build the interactome map at system level of chickpea and other legumes. In addition, gene mining of these databases, aided by microarray chip, can be used to select candidate genes involved in plant immunity. In the future, it will also be interesting to compare the spatial and temporal transcriptional complexity that underlies organ-specific innate immune response in other multicellular organisms.

## Results

### Chickpea EST database

**Figure 1:**
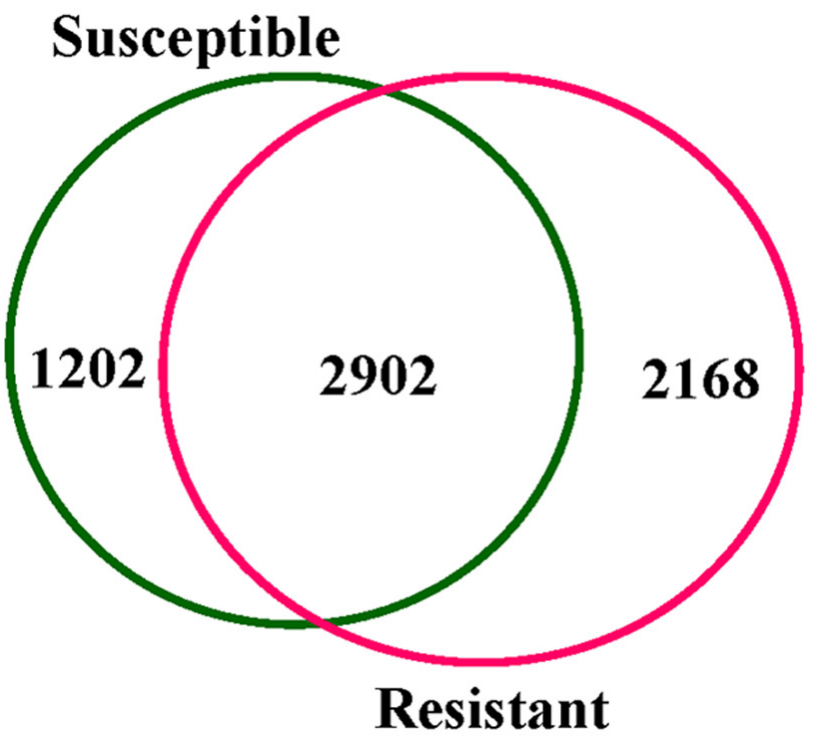
**Venn diagram depicting exclusive and overlapping *Ca*ESTs**. The numbers signify ESTs specific to the susceptible and resistant genotypes and common between them.

In an attempt to construct a functional EST dataset for discovering subset of genotype dependent, organ specific and extra and intra cellular stimuli responsive genes expressed in chickpea, in this study we have constructed two suppression-subtracted cDNA libraries, one from vascular wilt susceptible genotype (JG-62) and the other from resistant genotype (WR-315). The source of RNA for each library was root and collar tissue from the 25-d-old chickpea seedlings challenged with wilt pathogen, *Fusarium oxysporum ciceri *race 1, vs control tissue. In total, 6955 ESTs were generated and sequenced from the two libraries out of which 2908 were from susceptible and 4047 from resistant genotypes. Although no chimeric sequence was found but about 9.8% of the sequences were discarded due to low quality of the sequence, short sequence length, sequence from other organellar origin or the absence of the insert and the remaining 6272 ESTs were considered for sequence assembly (Table 1). The high quality ESTs that passed the base calling, appropriate length, vector masking and mitochondrial filters were assembled into contigs using CAP3 program [22]. The 6272 high quality sequences had an average read of 366 bp and a cumulative length of 2.29 Mbp. Of the total 6272 ESTs, 1040 were found to be classified as singletons whereas 5232 assembled into 973 contigs (Table 2). The additional information about contigs and singletons is given in additional file 1 and 2 respectively. The redundancy of clones in the contigs ranged from 2 to 230. The total contigs and singletons comprised a non redundant chickpea unigene (*Ca*Unigene) set of 2013 different transcripts (see Additional file 3). 3.9% of the unigenes (79) contained more than 10 sequences per contig. Of the total high quality ESTs, 1202 were specific to susceptible genotype whereas 2168 were specific to resistant genotype and 2902 were common to both (Figure 1). The abundance of transcripts, shapes or results in a genotype specific function in response to stimuli in a given space and time. Absence of chimeras, high percentage of good quality sequences and good average insert size suggested that the libraries were of high quality (Table 1).

**Table 1:** Chickpea library and EST characterization

	**Genotype**		
	
	**Total**	**WR**	**JG**
	
Sequence reads	6955	4047	2908
Failed base calling QC	8	6	2
Low quality sequence	214	121	93
Short insert sequence	422	246	176
No insert	30	30	0
E. coli	0	0	0
Mitochondrial	9	1	8
Total high quality	6272	3643	2629

**Table 2:** Sequencing and contigging statistics of chickpea ESTs.

**Genotype**	**Total No. of ESTs sequenced**	**Sequencing success percentage^a^**	**Good-quality ESTs used for contigging^b^**	**ESTs in contigs**	**EST singletons**
JG-62	2908	90.4	2629	2316	313
WR-315	4047	90.0	3643	2916	727
Total ESTs	6955	90.2	6272	5232	1040

^a^Sequencing success was determined by removing low quality, short insert, no insert and mitochondrial ESTs from the total number of ESTs sequenced.

### Functional annotation and classification of chickpea ESTs

**Figure 2:**
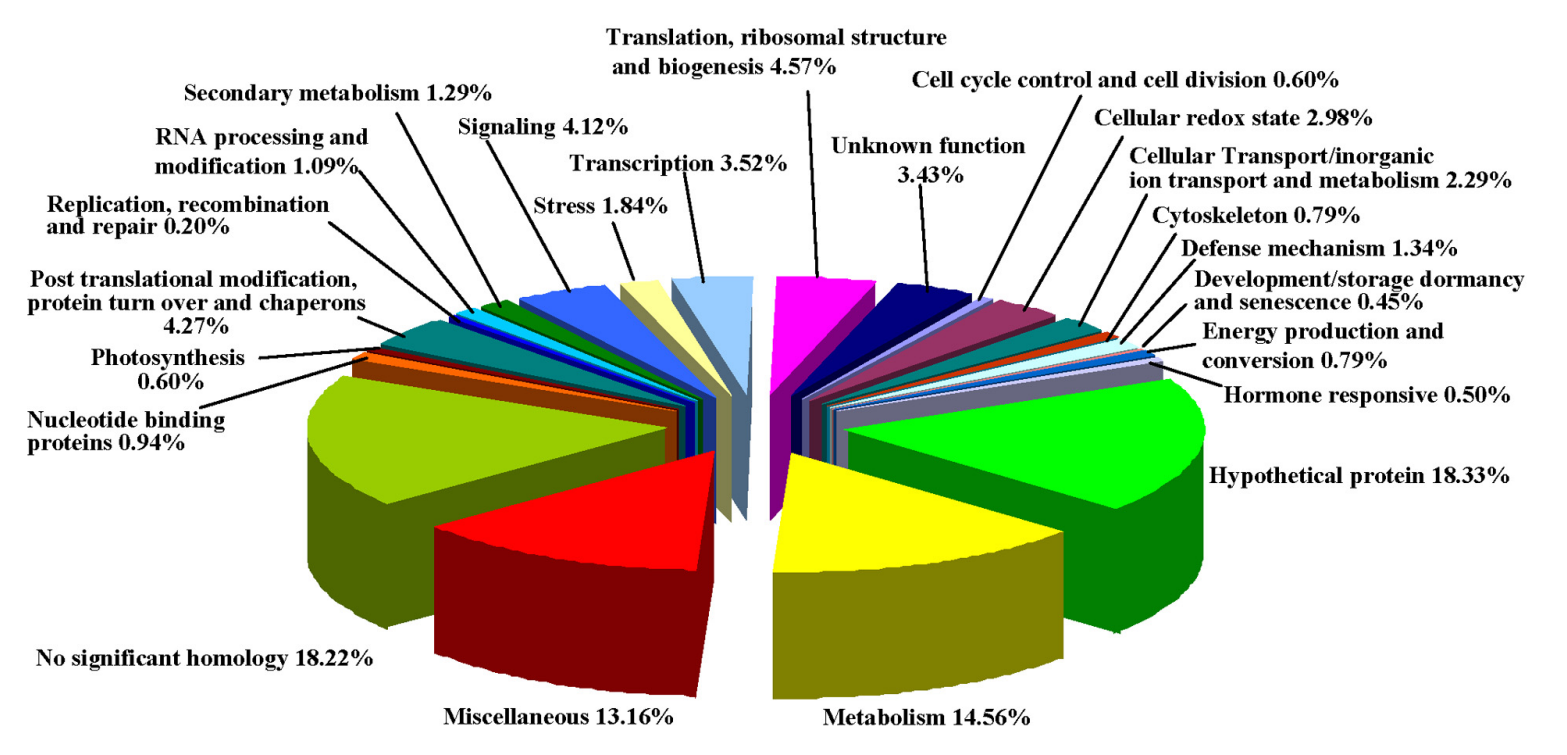
**Functional classification of *Ca*Unigenes from root transcriptome**. The genes identified were grouped into 23 functional classes as shown in the piechart based on metacyc, KOG and GO databases. The values represent the percentage of unigenes assigned to a particular functional class.

In order to understand the function of immune-responsive ESTs, the entire set of 2013 *Ca*Unigenes were annotated on the basis of similarities to the known or putative ESTs in the NCBI database. Using the best hits found by BLAST, an inferred putative function was assigned to the sequences and were sorted into various functional categories as shown in Figure 2. We were able to assign function to 60% of the genes while our data analysis revealed that 18.28% of the *Ca*Unigenes belong to no significant homology (NSH) class. In addition, 18.33% and 3.47% of the unigenes matched with hypothetical and unknown proteins respectively. Genes were assigned to the functional classes according to their biochemical function using gene function databases like GO , metacyc  and COG . However, the classification of transcripts is only tentative, since the biological function of many genes identified has not yet been established experimentally. The distributions of ESTs into diverse functional classes as shown in additional file 3 are described below.

### Genes involved in regulatory pathways (cell signaling and transcription and RNA processing and modification)

Genes involved in cell signaling, transcription and RNA processing are known to regulate many cellular responses in an organism. Our data showed the presence of different types of protein kinases in both susceptible and tolerant chickpea genotypes. While serine/threonine protein kinase (contig86), Serine/threonine protein kinase active site (CaF1_JIE_03_A_05) and somatic embryogenesis receptor-like kinase were present only in the susceptible genotype; flag-tagged protein kinase domain of putative MAPKKK and protein kinase (CaF1_WIE_51_C_11) were found in the resistant one. Protein kinases are involved in disease response via a signaling cascade presumably conserved in plants, insects and mammals [23] while somatic embryogenesis related kinases are associated with brassinosteroid signaling pathway that plays a key role in plant defense [24]. The EST data revealed many components of calcium signaling such as calmodulin, calcium dependent calmodulin independent kinase, CIP kinase and calcium/calmodulin-regulated receptor-like kinase in both the genotypes indicating an elaborate role of calcium signaling in immune response. The subsets of 14-3-3 proteins were also identified in both susceptible and resistant genotypes. For example, contigs 527, 568, 947 and singletons CaF1_WIE_13_A_05, CaF1_WIE_55_G_01 representing 14-3-3 gene family were present in the resistant genotype while the other member of this gene family, contig 133, was present only in the susceptible one. 14-3-3 has earlier been shown to interact with fusicoccin, the fungal toxin that causes membrane hyperpolarization through activation of plasma membrane H^+^ATPase [25]. Other pathostress-responsive signaling components identified are WD40-like protein, response regulator receiver, and leucine-rich repeat (LRR) proteins. While WD40 and LRR proteins were found in both the genotypes, response regulator receiver was exclusive to the susceptible genotype. The LRR proteins are known to play role in defense signaling [26]. While WD40-like proteins are reported to be involved in varied functions including organogenesis [27]; vacuolar trafficking [28] and light signaling [29], their role in immune response is not known.

The potentially increased activity of various signaling pathways is associated with differential expression of many families of transcription factors during plant pathogenesis [30-32]. The functional class of transcription associated genes, in this study, constituted about 3.58% of the total ESTs that comprised families of transcription factors including zinc finger, MYB, BEL1-like homeodomain transcription factor, homeodomain leucine zipper HDZ3, G-box binding PG2, Zinc finger GATA-type, putative AP2-binding protein, bHLH and WRKY. While exploring the source of the ESTs coding for these transcription factors, an important and noteworthy observation was that a particular class of transcription regulators showed predominance or was specific to either susceptible or resistant genotype. For example, Myb and HDZ classes were predominant, whereas, PR related transcription factor and WRKY were exclusive to the resistant genotype. On the other hand BEL1-like homeodomain transcription factor, NOT2/NOT3/NOT5 and G-box binding protein PG2 were found only in the susceptible genotype. This data suggests that interplay of a broad spectrum of transcription factors possibly regulates multiple signaling cascades during immune-stress.

Various RNA binding proteins have potential to modulate gene expression and might be involved in processes like RNA metabolism, mRNA splicing, ribosome biogenesis, transport and translation. We observed the presence of dead box RNA helicase, pre-mRNA processing ribonucleoprotein binding region, splicing factor 3B subunit 10 and RNA-binding region RNP-1 during vascular wilt of chickpea. All these genes were more abundant in resistant genotype. While dead box RNA helicase was reported to be involved in development and stress responses [33], the role of splicing factor and RNA-binding region RNP-1 in pathostress is yet to be established.

### Genes involved in translation, post translational modifications and protein turnover

Cellular processes like translation, post translational event and protein turnover are crucial for cell survival under different developmental stages and varied environmental conditions. In the present study, this class constituted about 8.84% of ESTs and comprised predominantly the ribosomal proteins apart from some translation initiation factors. Genes encoding major ribosomal proteins such as S6, S8, S9, and L24/L26 were found both in susceptible and resistant genotypes. Translation factor SUI1 homolog and eukaryotic elongation factors (EF-1α and EF-2) play a pivotal role in protein biosynthesis. Their presence in *Ca*Unigene set is indicative of their role in immune response.

A particularly sensitive, rapid and reversible response to environmental stimuli or to a programmatic change in cell state is the post-translational modification of specific proteins. In our study, 4.27% of the *Ca*Unigenes correspond to proteins involved in protein modifications and turnover. The presence of cysteine proteinases and cyclophilin-type peptidyl-prolyl cis-trans isomerases more predominantly in resistant genotype indicate their role in pathostress responses. The presence of heat shock protein, DnaJ is interesting as it has been shown to be involved in different environmental stresses including high temperature and salinity treatment [34]. Our results revealed the presence of a wide range of proteins involved in protein turnover that include ubiquitin conjugating enzyme E2, ubiquitin-protein ligase, 20S proteasome alpha 6 subunit, F-box protein and protein disulphide in both the genotypes. In plants, ubiquitin/proteasome pathway of protein degradation has been implicated in defense [35]. These results suggest that regulated protein synthesis, modification and protein turnover may play central role in enabling plants to alter their proteome to maximize their chances of survival under adverse conditions.

### Genes encoding nucleotide binding proteins

In our study, GTP binding proteins were found to be the major class of nucleotide binding proteins in susceptible as well as resistant genotypes. The stress-responsive predominant GTP binding proteins were of Ras, Rab and Ran type. ESTs encoding ras-like small monomeric GTP-binding protein and GMPase were present in the susceptible genotype while Rab, RAB7C, GTP cyclohydrolase and RabGAP/TBC were found only in the resistant one. However, ESTs encoding Ras small GTPase and RAB1X were identified in both the genotypes. GTP binding proteins are reported to be associated with a wide range of growth and developmental processes [36] besides their role in plant defense [37].

### Genes involved in metabolism

Developmental changes and stress responses are often correlated with or result in adjustments in various metabolic pathways. The functional class of metabolism comprised of about 14.56% of the unigenes and represented the largest class apart from NSH and hypothetical proteins. The genes present in this class represented several biochemical pathways such as carbohydrate, fatty acid, energy and protein metabolism besides nitrogen and nucleotide metabolism. The genes involved in carbohydrate metabolism comprised alcohol dehydrogenase (ADH), glyceraldehyde3-phosphate dehydrogenase (GAPDH), enolase, fructose-bisphosphate aldolase, malate dehydrogenase, phosphoenol pyruvate (PEP) carboxylase and triosephosphate isomerase and were present in both the genotypes. Most of these enzymes are known to show altered expression in response to stress [38,39]. Though very little is known about the direct role of these proteins in defense, many such enzymes are involved in maintaining metabolite pool that may drive the metabolic processes to overcome such stresses. Polygalactouronase inhibiting protein (PGIP) was present in both the susceptible and resistant genotypes. The PGIPs are cell wall proteins, which act against fungal polygalactouronases that are important pathogenecity factors [40]. Besides their classical function, they also form long chain oligogalactouronides and facilitate the activation of plant defense responses [41]. We observed the presence of β1,3-glucanase, a PR 2 family protein [42], which was earlier shown to be expressed in response to pathogen infection [43].

The genes involved in lipid biosynthesis, for example, acyl Co-A synthetase and acyl Co-A binding proteins were found to be present in both the genotypes. The enzyme acyl Co-A synthetase has been shown to be induced in response to compatible and incompatible interactions between *Xanthomonas *and *Capsicum annum *[44], while Acyl Co-A binding proteins play an important role in intracellular transport and formation of acyl Co-A pools [45]. ESTs encoding desaturases in the CaUnigene set showed varied substrate specificity between the two genotypes. For example, oleate and sterol desaturases were found in the susceptible genotype and putative desaturase-like protein was present in the resistant one. Desaturases catalyze the formation of double bonds in lipids thereby increasing the lipid fluidity, which might be required as an adjustment associated with membrane stability under progressive wilting. ESTs corresponding to other lipid metabolism genes such as serine C-palmitoyltransferase, was also found to be present in stressed tissues. Identification of sterol metabolism related enzymes such as sterol methyl transferases and oxysterol binding proteins suggest the occurrence of lipid modifications during pathostress. Presence of HMG Co-A reductase which catalyzes the synthesis of mevalonate, a specific precursor of plant defense metabolite sesquiterpene phytoelexins, is indicative of its role in cell defense.

Several amino acids act as precursors for the synthesis of specialized metabolites, during varied cellular adaptations. ESTs encoding enzymes involved in amino acid metabolism, for example, arginine decarboxylase, aspartate aminotransferase, and cysteine synthase were identified in both the genotypes. Recent evidence suggests that arginine decarboxylase, involved in putrescine biosynthesis, is induced in response to various environmental stresses [46,47]. Proline dehydrogenase and many proline-rich proteins were identified in resistant genotype. Proline dehydrogenase is the rate-limiting enzyme in proline degradation and serves important functions in stress responses [48].

Nucleotide metabolism related genes such as nuleoside diphosphate kinase (NDK), adenine nucleotide translocator and polynucleotide phosphorylase were also identified in both the genotypes. Previously, it has been shown that the overexpressing NDK provides higher ability to eliminate H_2_O_2_, indicating its potential role in the management of reactive oxygen species under stress [49].

### Genes involved in cellular transport and homeostasis

Stress-induced reorganization and spatial distribution of many key metabolites in plants require efficient transport machinery. It is well known that Arabidopsis has diverse array of genes for multi-efflux transport and response to stress signals, and rice has more secondary transporter genes for carbohydrate and nutrient transport [50]. Various transport associated genes were identified in this study. Of these, clathrin adaptor complexes and putative polyol transporter protein 4 were expressed in the susceptible genotype while multidrug resistance protein, Sec Y protein, ABC transporter family protein, Ctr copper transporter, SKS3 copper ion binding protein showed expression only in the resistant genotype. Although few of these transport associated proteins showed genotype specificity, others like general substrate transporter, intracellular chloride channel, phosphate transporter 5, were representative of both the genotypes. ABC transporters and multidrug resistance genes are known to function in plant defense [51,52], while the role of other above mentioned genes in plant defense is yet to be discovered. The chloride channel was reported to be activated by CDPK in guard cell [53] whereas the chloride transporter has been found to be involved in hypo-osmotic turgor regulation [54]. Further, polyol transporter was shown to be expressed during maturation of common plantain companion cells [55], though its role in plant defense remains to be ambiguous. These results suggest that transport of both organic and inorganic substances may play a crucial role in immune response.

### Genes involved in hormone responses

Salicylic acid (SA), jasmonic acid (JA) and ethylene play key roles in developmental regulation and stress responses through cross communicating signal transduction pathways. These hormones accumulate in response to pathogen infection and in turn lead to the activation of distinct sets of defense related genes [56]. We observed presence of ethylene signaling pathway genes, for example, ethylene responsive transcoactivator and putative ethylene response protein in the susceptible genotype while ethylene receptor was present only in the resistant genotype. Coronatine-insensitive 1 and BRU1 precursor which are associated with JA and brassinosteroid pathways respectively, were specific to the resistant genotype. In addition, we found auxin signaling related proteins, of which, auxin-responsive SAUR was found in both the genotypes but auxin-regulated dual specificity cytosolic kinase was present only in the resistant genotype. Aux/IAA protein, auxin-induced protein IAA12 and auxin-induced putative aldo/keto reductase family protein were found specifically in the susceptible genotype. Our future efforts would be the investigation of their possible role in plant immunity.

### Genes involved in cell cycle and DNA metabolism

Cell division and cell cycle progression in plants is often altered in response to various environmental stresses [57]. Many cell division and cell-cycle related proteins, for example, putative kinetochore, cell division protein FtsZ and cdc2MsF identified in this study were predominant in the resistant genotype suggesting pathostress responsive alterations of cell cycle in chickpea. Genes involved in DNA replication and repair like DNA repair protein RadA, putative polyprotein and putative gag-pol polyprotein were identified from susceptible genotype while putative helicase was common in both the genotypes. These findings are interesting because very little is known about the role of such genes in plant immune responses.

### Genes involved in development and cytoskeletal organization

The genes involved in development and cytoskeletal organization account for 1.24% of the total *Ca*Unigene set. The candidate genes involved in development were those encoding enzymes associated with fruit ripening and senescence, and several storage proteins like albumin and agglutinin. Most of the genes in this class were identified in both the susceptible and resistant genotypes, however agglutinin was specific to the resistant one. While there have been reports on association of the processes of plant defense and senescence [58], the involvement of ripening related proteins has never been implicated in immune responses. Seed storage proteins like germin and albumin have been shown to be involved in stress responses [59], however, role of agglutinin in plant immunity is not known.

Cytoskeleton is thought to contribute to the establishment of effective barriers at the cell periphery against pathogen ingression. Substantiating this phenomenon, several structural proteins were identified that include actin, microtubule bundling polypeptide, and beta tubulin, besides genes associated with cytoskeletal reorganization like actin-depolymerizing factor and putative spindle disassembly related genes. Although actin was present in both the genotypes, actin-depolymerizing factor and putative spindle disassembly related genes were specific to resistant genotype.

### Genes involved in cellular redox and energy metabolism

The oxidative burst is one of the earliest cellular responses following successful pathogen recognition. Several enzymes involved in oxidative burst were identified in both susceptible and resistant genotypes that include peroxidase, superoxide dismutase, glutathione-S-transferase and quinone oxidoreductase. Apoplastic generation of superoxide or its dismutation product, hydrogen peroxide, has been shown in response to a variety of pathogens [60]. These enzymes restrict the ROS-dependent damage and may lead to the activation of plant immune response [61].

Energy production has an impact on the overall metabolic state and the energy supply is the key factor for the maintenance of cell intactness under various stress conditions. We observed the presence of genes encoding different proteins involved in ATP biosynthesis like alternative NAD(P)H dehydrogenase and putative ADP, ATP carrier-like protein in susceptible genotype. However, alternative oxidase 2b and NADH dehydrogenase were identified in the resistant genotype only. Nevertheless, vacuolar H^+^-ATPase subunit A and ATP synthase beta subunit were common to both genotypes. While the involvement of these proteins in abiotic stress, energy conservation and maintenance of redox potential is well established [62], their exact role in plant immune response is yet to be elucidated.

### Genes involved in secondary metabolism

Most secondary metabolites of phenyl propanoid pathway, including lignins, isoflavonoid-phytoalexins and other phenolic compounds are instrumental in plant's ability to enforce successful defenses against invading pathogens. In this study, several genes were identified that are associated with biosynthesis of secondary metabolites. The important enzymes in this category include phenyl ammonia lyase (PAL), chalcone synthase, chalcone isomerase, and chalcone-flavonone isomerase-1 which were found in both compatible and incompatible interactions. These enzymes are known to modulate plant defense response against invading pathogens and insects [63,64]. Some of the secondary metabolism related genes, for example, squalene epoxidase was found to be specific to susceptible genotype whereas pterocarpan reductase and oxysterol-binding family protein were specific to the resistant one. Other members of this class are caffeic acid O-methyltransferase II, isoflavone 3'-hydroxylase, squalene monooxygenase 2, trans-cinnamate 4-monooxygenase and dihydroflavonol reductase. Earlier studies have shown that transgenic rice plants overexpressing dihydroflavonol reductase can provide tolerance to biotic and abiotic stresses [65]. Caffeic acid O-methyltransferase catalyzes a key step in lignin biosynthesis [66], thereby giving protection against pathogen attack.

### Defense and stress responsive genes

Pathogen attack is often accompanied by the accumulation of elevated levels of transcripts of disease related proteins, the PR genes. The ESTs encoding proteins implicated in stress and defense responses account for 3.18% of the total unigene set. Most dominant candidates among the defense responsive genes were disease resistance response protein DRRG49C and PR10. Other genes identified include those encoding chitinase, non-specific lipid transfer proteins, thaumatin and pathogenesis-related protein. The involvement of these proteins in plant defense responses is well known [67]. One of the important observations was that DRRG49C, thaumatin and pathogenesis-related protein were expressed only during incompatible interaction which may lead to increased tolerance to pathogen attack. Also, we found the presence of ESTs encoding proteins like dirigent and harpin-induced 1 in the resistant genotype, implying their role in pathostress response. Dirigent protein has recently been shown to be involved in lignification and hence imparting disease resistance [68]. Many other stress induced proteins, for example, extensin, universal stress proteins, aquaporin, annexin, cold acclimation responsive protein BudCAR4 and metallothionien were also identified as more abundant in the resistant genotype implicating their involvement in pathostress response besides their key role in abiotic and other stresses [69,70].

### Comparison with other legume genome and EST databases

To investigate how many of the chickpea ESTs from this study are highly orthologous to other legume ESTs, we performed a comparative analysis of our *Ca*EST dataset to publicly available dataset comprising ESTs from *Arachis hypogea, Cajanus cajan, Pisum sativum, Robinia pseudoacacia, Lotus japonicus, Medicago trunculata, Glycine max *and *Phaseolus vulgaris*. Except *Phaseolus *and *Robinia*, these databases were inclusive of root ESTs. The highest number of common orthologous ESTs was found to be present in *Pisum sativum*, although, *Cajanus cajan *and *Arachis hypogea *also showed higher similarity with *Ca*Unigenes. The percentage of chickpea ESTs that matched to *Pisum sativum *was 88.8% and 79.43% at DNA sequence identity of ≥ 80% and 90%, respectively. A total of 87.6% and 83% Cajanus cajan ESTs and 85.89% and 55.54% of Arachis ESTS were common with *Ca*Unigenes. Chickpea and Pisum show close phylogenetic affliations, both being representatives of galegoid, a group of cool season legumes [71] and is reflected in their sequence similarities. However, high sequence similarity with Arachis and Cajanus came as a surprise since these legumes are phylogenetically distant relatives of Cicer and belong to tropical season legumes (phaseoloid). The comparative analysis of chickpea ESTs with that of *Glycine max *showed homology of 80.32% and 31.54%. Interestingly, Medicago and Lotus showed more divergent trend with only 58.02% and 30.50% and 43.31% and 14.25% ESTs respectively, having counterpart in *Ca*Unigene set. A recent phylogenetic study based on penalized likelihood analysis [72] indicated that Medicago, Lotus and Cicer are closer and fall in the same galegoid clade, however, the divergence/difference in the pattern of ESTs may be attributed to the fact that gene expression is shaped by cellular environment besides ecological niche of the corresponding organism. Chickpea ESTs showed lesser homology with *Robinia pseudoacacia *and was about 12% and 3.92% at DNA sequence identity of 80% and 90%, respectively. This can be due to the fact that Robinia is a perennial tree while chickpea is an annual herb. Detailed comparative analysis of ESTs is shown in Table 3. Thus the current study documents substantial conservation as well as genome divergence amongst legume crops and in future can facilitate cross species analysis of gene function.

**Table 3:** Comparative matching of *Ca*ESTs to the ESTs of other legume databases.

**Gene indices**	**Identity >80%**	**Identity>90%**
Arachis hypogea	1686 (83.75)	1118 (55.54)
Cajanus cajan	1764 (87.6)	1729 (85.89)
Pisum sativum	1788 (88.8)	1599 (79.43)
Robinia pseudoacacia	245 (12.17)	79 (3.92)
Lotus japonicus	872 (43.31)	287 (14.25)
Medicago trunculata	1168 (58.02)	614 (30.50)
Glycine max	1617 (80.32)	635 (31.54)
phaseolus vulgaris	928 (46.10)	234 (11.62)

The chickpea ESTs isolated in this study were compared to other legume databases like *Arachis hypogea, Cajanus cajan, Pisum sativua, Robinia psedoacacia, Lotus japonicus, Medicago trunculata, Glycine max and Phaseolus vulgaris *collected from NCBI and TIGR gene indices. The criteria for stand alone BLASTN were (1) exact match = 11; (2) e-value cutoff 1e-5; and (3) identity > 80% and 90% at DNA sequence level.

### Identification of chickpea specific transcripts and enrichment of CaEST

In an attempt to identify the genotype-specific transcript signatures in chickpea, we performed a blast analysis of *Ca*Unigene. Of the total 2013 *Ca*Unigenes, about 18.22% sequences that belong to NSH group represented potential chickpea specific sequences. To verify whether these sequences were indeed chickpea specific, TBLASTX was used for comparing them to the EST_others database. Nearly 64% of the NSH class of *Ca*EST dataset did not show any significant match thereby confirming that these ESTs represent unique chickpea sequences. Thus this dataset represents 234 novel chickpea specific ESTs, which were never reported earlier. Further, we compared our *Ca*Unigenes with the earlier reported ESTs of chickpea. TBLASTX with e-value cutoff of 10^-10 ^showed that 532 unigenes from the *Ca*Unigene set of 2013 had a significant match, while the remaining 1483 did not have any counterpart. Thus, 73.67% of our *Ca*Unigenes represent new chickpea genes yet unidentified. Full length sequencing of these unigenes and their expression analyses may provide further insight into the species-specific functions of these genes.

### Comparative stress responsive transcriptome reveals canonical and non-canonical genes

**Figure 3:**
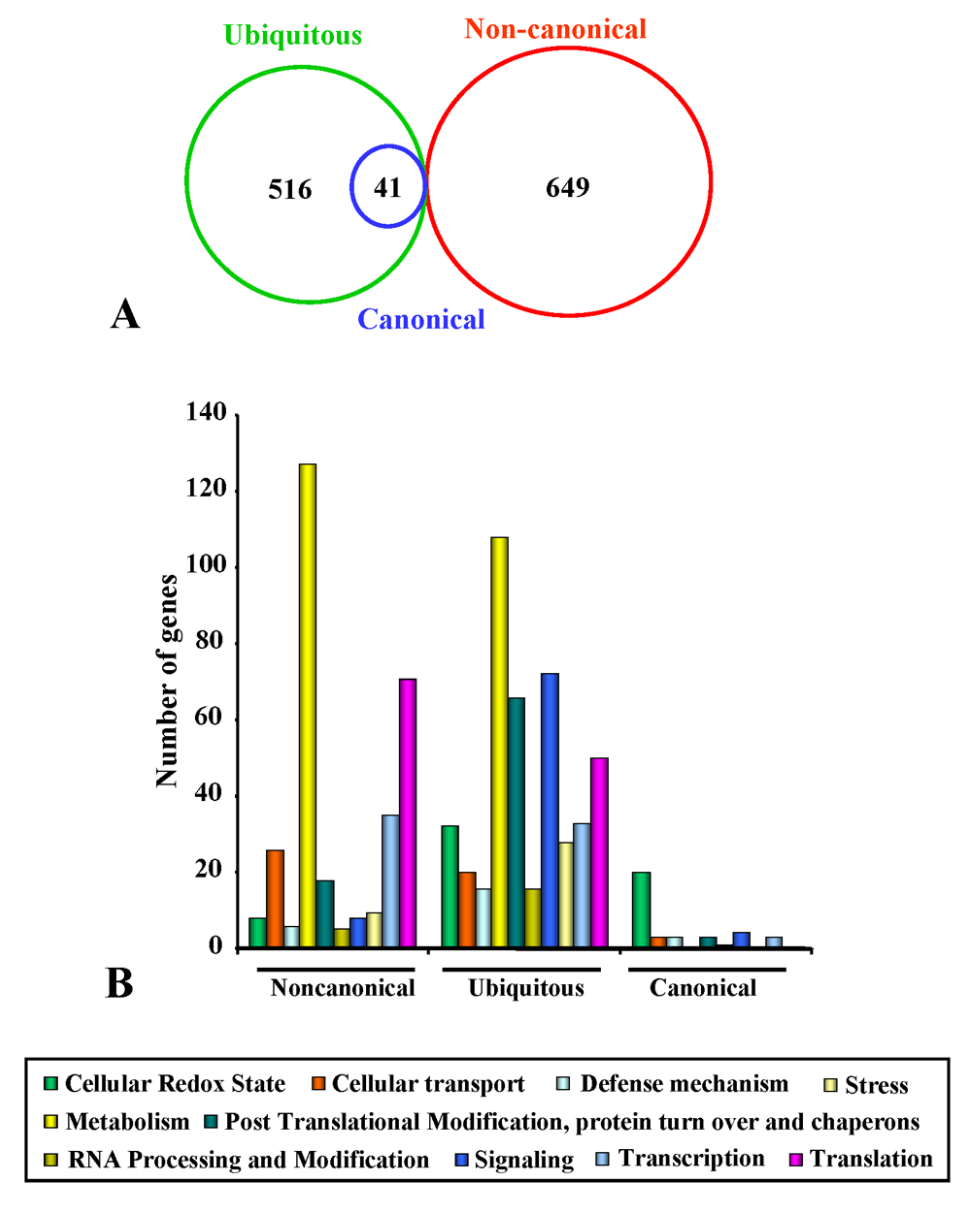
**Comparative analysis of chickpea stress responsive genes with earlier known stress related genes**. (A) Venn represents the overlap between ubiquitous, canonical and non canonical genes and the numbers signify unique and common stress responsive genes. (B) Expression pattern and prevalence of functional classes within the three groups. Each bar represents the number of genes in the respective functional class.

Although a number of studies have catalogued stress-responsive genes against different environmental conditions into various functional classes, comparative genomics of stress-responsive transcriptome is still lacking. To investigate the comparative biology of stress-responsive transcriptome at organismal level with the currently available data, we compared the *Ca*Unigenes with previously reported stress-responsive genes from other organisms. We classified genes known to be involved in multi-stress responses as ubiquitous, while those found to be specific to *Fusarium *infection were categorized as canonical. All other genes, which have never been implicated in any stress, were designated as noncanonical (Figure 3A). We found that around 516 genes are ubiquitous, suggesting broad similarities in stress responses across most of the organisms and confirming cross communicating pathways playing role in different kinds of stresses. However, only 41 genes were found to be canonical all of which showed an overlap with ubiquitous class. A significantly large number of genes (649) were found to be noncanonical. This difference in the pattern of immune-responsive root transcriptome may be attributed to the fact that the gene expression in an organism is shaped by the cellular environment and the epigenetic factors. Metabolism was one of the most abundant functional class in all the three groups (Figure 3B), suggesting that any stress response results in the alteration of the metabolic pathways of an organism. A few of the metabolic enzymes found to be expressed in response to multiple stresses represented in the ubiquitous category included S-adenosylmethionine decarboxylase, glyceraldehyde 3-phosphate dehydrogenase, methionine sulfoxide reductase, and copper amine oxidase [38,73-75]. Cell signaling related genes also formed an important class that included leucine-rich repeat receptor-like kinase and multiple bridging factor among others [76,77]. As expected, many canonical genes belonged to functional class of cellular redox, defense and signaling; included Cationic peroxidase 2, chitinase and 14-3-3 protein [78]. Our data on immune responsive transcriptome and comparative analysis thereof provide evidence for molecular diversity vs. commonality in gene expression profile at organismal level. The comparative analysis revealed few canonical genes, several ubiquitous genes across different stresses while most of the genes were found to be non-cannonical. An interesting observation was that apart from metabolism, most of the noncanonical genes belonged to the functional classes of translation, posttranslational modifications, transcription, and signaling suggesting the occurrence of new, yet undiscovered immune response pathways. This study thus provides a comprehensive catalogue of non-canonical immune responsive genes or might suggest their species specificity with new insight into their identity and function.

### Identification of gene families

To assign *Ca*Unigenes from this study to putative gene families, we used single linkage clustering. The individual contigs and singletons were combined into a single dataset which was then compared to itself using TBLASTX with an e-value cutoff of 1e^-15^. Sequences with overlapping BLAST reports were assigned to a putative gene family. Altogether, we identified 209 gene families ranging in size from 2 to 29 members (see Additional file 4). This analysis gives insights into following three areas: firstly, it identifies the genes that are likely to cross hybridize during the microarray hybridizations. Secondly, it helps in assigning possible function to genes that had no significant homology to known proteins or belonged to class of UF but clustered with proteins of known function. For example, gene family 23 had two members of which one showed homology to CAP protein while the other showed homology to hypothetical protein. Similarly, one of the members of gene family 32 showed homology to cinnamoyl-CoA reductase, while the other two belonged to NSH class. Also, one member of gene family 124 showed homology with phosphoesterase but the other did not have any significant match. Therefore, on the basis of the family to which such genes either of NSH or UF belong, possible function can be assigned to them and further verified by comparing sequence alignment of these sequences with representative members of the known gene family. Thirdly, the identification of gene families provides a base for uncovering and understanding the biological rationale of functional novelty and partitioning following gene duplications. Towards this, it was interesting to note that in several cases the distribution of different members of the same gene family varied between the genotypes. For example, histone deacetylase HDT1 (contig212) was found to be specific to susceptible genotype while the other member of the same gene family represented by contig713 was present only in the resistant genotype. This was also the case with methionine sulfoxide reductase A, where contig152 and contig555 were specific to susceptible and resistant genotypes respectively. Putative esterase family protein (CaF1_JIE_28_D_10, CaF1_WIE_32_H_04) and putative desaturase (contig454, CaF1_JIE_22_A_09) also showed the same trend. This opens up a new area for finding out whether presence of different gene family members leads to genotype specific response to a particular kind of stress.

### Analysis of genotype-specific SNPs of chickpea

SNPs between genotypes/haplotypes once discovered are extensively used for many applications for instance generation of very dense genetic maps, to construct the specific genotypes required for quantitative genetic studies, to enhance understanding of genome organization and function and address fundamental questions relating to evolution. SNPs can also be used for genome-wide linkage disequilibrium and association studies that assign genes to specific functions or traits. Furthermore, transcript-associated SNPs can be used to develop allele-specific assays for the examination of *cis *regulatory variation within a species [79]. In the present study, SNPs were identified between JG-62, a susceptible and WR-315, a resistant genotype of chickpea to vascular wilt. A total of 279 contigs (28.67% of the total) contained at least one sequence from both the genotypes and were mined for potential SNPs. We identified 262 SNPs which were further classified into high and low quality SNPs depending upon the number of sequences from each genotype showing the same base change (see Additional file 5). High-quality SNPs were confirmed by two or more sequences from each genotype showing the same base change, while low-quality SNPs were confirmed by one sequence from one genotype and at least two from the other. Thus, we identified 136 high-quality and 126 low-quality SNPs. In the present analysis only base pair mutations were taken into consideration and among these transitions (73.3%) were more common than transversions (26.7%). Within the transitions, occurrence of both adenine to guanine and cytosine to thymidine base changes were found to be almost equal. The maximum number of SNPs were present in contigs that comprised of highly abundant ESTs, for example, contig 722 represents the most abundant contig and maximum number of SNPs were present in this contig. Similarly, contig 680 and 575 also had high percentage of SNPs and were also very abundant as far as total number of ESTs in these contigs is concerned.

### Immune responsive gene networks in root transcriptome of chickpea by microarray analysis

**Figure 4:**
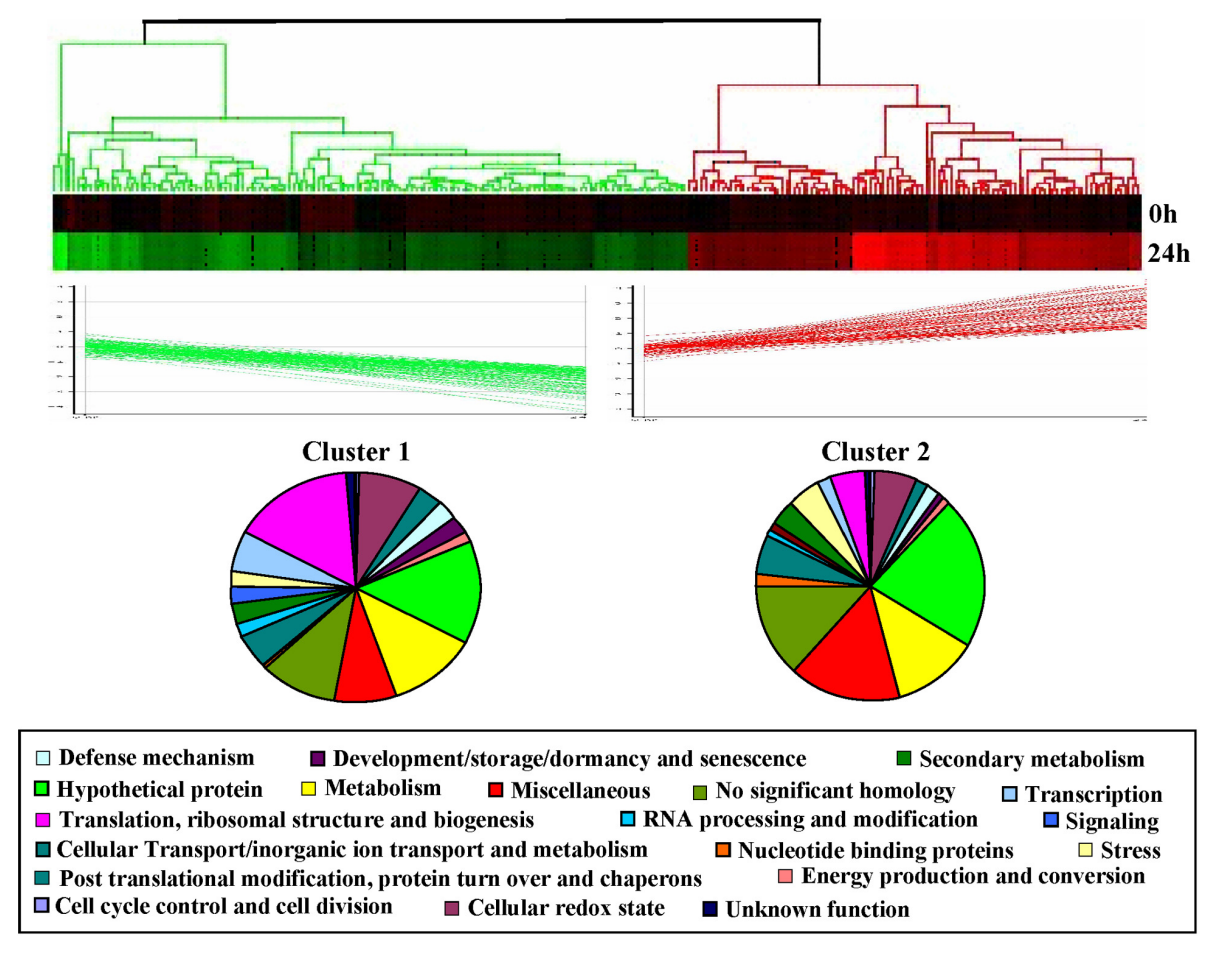
**Tree view and functional categories of hierarchically clustered genes during *Ca*-*Fusarium *incompatible interaction**. Tree view represents the expression patterns of genes displaying significant regulation in response to *Fusarium *infection at 24 hours post pathogen inoculation. Genes were organized using hierarchical clustering and displayed, including Cluster1 that represents the significantly repressed genes and Cluster2 that corresponds to significantly induced genes. Each row represents a single gene and each column corresponds to log2 (ratio) of an experimental sample. Black indicates the median level of expression, red indicates greater expression than the median and green less expression. Pie below each cluster shows the distribution of clustered genes into various functional categories. Detailed information on genes within each cluster can be found in additional file 6.

To study the transcriptional remodeling in immune response during vascular wilt, we have developed cDNA microarray using *Ca*EST clones of the subtracted cDNA libraries from susceptible and resistant genotypes. Root tissue sample from WR-315 (resistant genotype) harvested at 24 h post *Fusarium *infection was used to evaluate the expression profile during early phase of incompatible interaction. We used indirect labeling of cDNAs following TSA protocol that incorporates flourscein and biotin labeled dUTP into the nascent cDNA from the target RNA instead of Cy3 and Cy5 modified nucleotides since it is known to negate any dye bias during the microarray experiment [80-82]. The transcript level for each cDNA was calculated as the average intensity of four data points from duplicate spots of two replicate slides. We used a fold cut off of 2.5 and Students t-test with P-value ≤ 0.05 ranking with false discovery rate (FDR) multiple testing corrections to identify differentially expressed genes. A total of 257 differentially expressed unigenes were found to be associated with the early signaling pathway, of which 107 were induced and 150 repressed during incompatible interaction (see Additional file 6). Further, to identify co-expression kinetics amongst the differentially expressed genes, we applied hierarchical clustering analysis to the transcriptome data set that grouped the genes in two clusters based on their positive and negative fold change. Cluster 1 represents repressed genes while cluster 2 comprised of genes induced at 24 h post *Fusarium *infection. Almost all the 23 functional classes are represented in these two clusters but differed in the type of the representative genes and their relative abundance (Figure 4). Moreover, the transcriptional response to immune sensitivity of genes involved in major biological processes and molecular functions showed different dynamics within repressed vs. induced gene clusters. The detailed expression level has been highlighted in additional file 6. The repressed gene cluster is enriched for genes affecting translation, ribosome structure and biogenesis, while the induced cluster is dominated by genes encoding hypothetical proteins, metabolism and miscellaneous groups. Genes involved in metabolism GO category are over-represented in both cluster 1 and 2, but the magnitude of over-representation is higher for cluster 1 than cluster 2. UF, NSH and Miscellaneous categories notably formed a major group of overrepresented genes.

Genes involved in translation, ribosome structure and biogenesis form the largest functional category in cluster 1 that includes genes for ribosomal proteins, translation initiation and elongation factors. Several genes encoding proteins involved in post translational modifications and protein turnover, for example, protein disufide isomerase-like protein and ubiquitin were also grouped in cluster 1. Many of these genes have previously been implicated in specific stresses in different organisms [83,84]. In the repressed set of genes other major functional classes included metabolism, hypothetical proteins, cellular redox state and transcription. These include genes encoding cytochrome P450, thioredoxin, superoxide dismutase and glutathione-S-transferase that are known to play critical role in plant stress [85-87]. Many carbohydrate metabolism genes like GAPDH, triosephosphate isomerase and malate dehydrogenase precursor were downregulated. Earlier reports showed altered expression of GAPDH during plant stress [38], however the specific function of this enzyme during stress is not known. Moreover GAPDH has also been known to act as an essential component of transcriptional activator complex regulating histone expression [88]. We also observed defense related genes like chitinase 1 and PR10, and secondary metabolism genes like chalcone synthase and isoflavone 3'-hydroxylase to be repressed during *Fusarium *infection. These genes are known to be associated with pathogen stress [63,64].

In contrary, genes encoding hypothetical proteins, metabolism and miscellaneous groups form the dominant functional class in cluster 2. The induced set of metabolic genes include some carbohydrate metabolism genes like ADH1, FBF, aldolase and pfkB-type carbohydrate kinase family protein of carbohydrate metabolism and oleate desaturase involved in lipid metabolism. The role of these enzymes in plant immunity has not yet been defined and hence will be of great interest in future. Post-translational modification and protein turnover related genes like cystatin, Sec61beta and ubiquitin extension protein were also induced consistent with the notion that damaged or partially denatured proteins need to be degraded to prevent the accumulation of protein aggregates. Many such genes are involved in regulating protein metabolism in response to stress [89,90]. Other cell stress induced genes include classical phenylpropanoid pathway genes like chalcone reductase and chalcone--flavonone isomerase 1. Role played by these genes during cell defense is a well known phenomenon [64]. Many signaling related genes like serine/threonine protein kinase, leucine-rich repeat transmembrane protein kinase to name a few were induced attesting their role in plant defense.

More than thirty percent of the repressed and induced gene clusters represented NSH and hypothetical proteins those do not have known cellular roles, these can now be annotated as being involved in immune response pathway.

### Expression analysis of selected ESTs by RNA blot analysis

**Figure 5:**
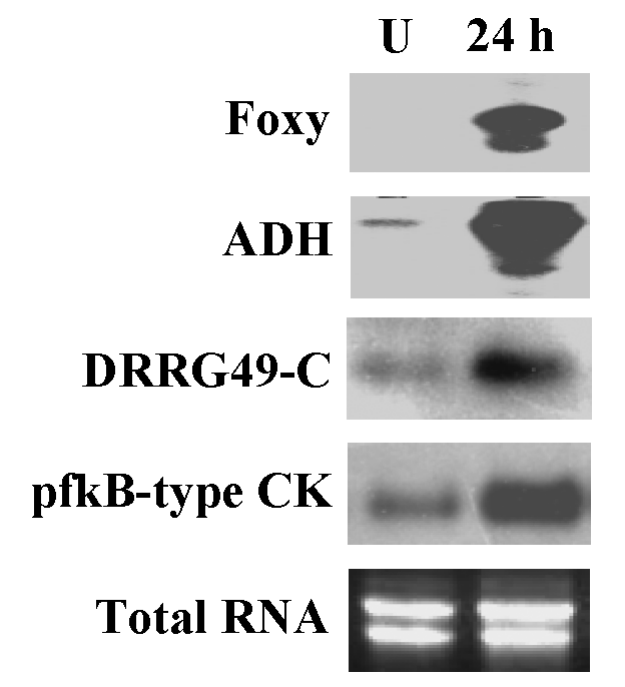
**RNA blot analysis of few immunity induced genes of *Ca*-*Fusarium *incompatible interaction**. 20 μg of total root RNA isolated from 25-day old chickpea seedlings were separated by 1.5% agarose gel. Lane U represents signal from the water treated control plants whereas lane 24 h represents RNA from 24 hours post pathogen challenge. The blots were hybridized with the respective gene specific probes as revealed by the microarray results.

To confirm the differential expression of immune-responsive genes, four highly abundant genes in our *CaEST *dataset showing induced gene expression were selected for RNA blot analysis. The results showed that all four ESTs had a strong induction in root tissue at 24 h post infection and the level of gene expression corroborated to that of microarray analyses (Figure 5, see Additional file 6). The genes encoding alcohol dehydrogenase and pfkB-type kinase, showed a very high expression suggesting that the carbohydrate metabolic pathways are altered in response to vascular wilt. A disease resistance response gene for defense signaling also showed upregulation indicating its role in immune response. The expression level of a fungal gene FOXY, found to be highly adundant both in susceptible and resistant genotypes was consistent with that of microarray data. This gene encodes a transposable element and its induction during plant-pathogen interaction is of interest for the study of such elements and their involvement in immunity vs. disease.

## Discussion

EST sequencing is likely to make its greatest impact on understudied genomes where full genome sequence projects may not be undertaken in near future. Although as of February, 2009 GenBank contained over 45,71,266 entires for the ESTs from plants, most publicly available ESTs are derived from different organs and developmental stages, while the species-specific EST dataset, let alone the genotype-specific ESTs are negligible. Very recently, the focus has been given towards the SNP and copy number variation as a basis for species specific genomic signatures. A recent study in *Arabidopsis *has revealed one million SNPs in a total population comprising different ecotypes [91]. Further, 78,373 in rice and 65,83,583 in humans were found to be associated with the coding sequences, intron and splice sites . The regulation of gene expression is an important mechanism, which controls cellular processes and it is conceivable that the functional transcripts resulted from a controllable transcription and posttranscriptional processes may shape the specificity at genotype/haplotype level.

In an attempt to understand the molecular basis of immune response in chickpea during vascular wilt, the transcriptional changes were monitored by EST sequencing approach. The sequences reported in this study have enriched the collection of ESTs in this food legume which would form a basis in our understanding of plant metabolism, development and adaptation to pathogen stress. These data are particularly important, at least in part, due to the fact that there are only 7464 chickpea EST sequences available currently in the NCBI database and we have doubled the number. This would broaden the current chickpea database for further functional studies. Although a large percentage of ESTs (46.27%) was common to both the susceptible and resistant genotypes, a significant percentage of ESTs was specific to each of them. The percentage of unigenes specific to susceptible genotype was 19.16%, while that of resistant genotype was 34.56% (Figure 1) indicating the possible divergence in immune signaling pathways. The occurrence of almost the double the number of ESTs in the resistant genotype as compared to the susceptible one may be attributed to the fact that responses in the susceptible and resistant genotypes to pathogen stress are largely quantitative and kinetically controlled. It is known that resistant genotype shows immediate response upon pathogen infestation which is of high amplitude, in contrarary, the susceptible genotype shows late response [92]. Further, among the entire set of ESTs, 40% represented the sequences of hypothetical proteins or unknown proteins or showed no significant homology to the previously identified genes or proteins in the NCBI database. These ESTs may represent unique chickpea sequences. It is of interest that the *Fusarium *infection of chickpea causes substantial changes in the expression of numerous unknown transcripts yet to assign a cellular function.

### Comparative abundance of ESTs in resistant and susceptible genotypes

Analysis of EST frequency comprising a contig and the source of the contig provides an insight with respect to the spatial expression level and biochemical functions even without subsequent microarray analysis. In most of the cases and within statistical limitations [93], the abundance of a specific transcript in the EST dataset serves as a measure of gene expression. Therefore, we identified and compared relative abundance of ESTs in different contigs to unravel the transcript signatures, which might determine the genotype-specific immune response. Contigs having ESTs from a single genotype were designated as specific. Of the 973 contigs, 433 were found to be specific to the resistant genotype, 261 to the susceptible genotype, and 279 contigs represented both the genotypes. While looking at the abundance of ESTs, we observed that the genes involved in the pathways that could be employed in cell defense, were represented more in resistant genotype than susceptible one. In this context, the gene encoding transaldolase that is involved in pentose phosphate pathway was found to be associated with the resistant genotype. Expression of this gene may be explained to the possible need of NADPH required for biosynthesis of various secondary metabolites like PR proteins. Similar was the case with enolase, a glycolytic enzyme, involved in the formation of phosphoenol pyruvate, which contributes to the formation of aromatic amino acids via shikimic acid pathway [94]. Aromatic amino acids act as primary substrates for phenylpropanoid pathway. Many genes involved in phenylpropanoid pathway, for example, chalcone reductase, chalcone synthase, and isoflavone reductase were more abundant in resistant genotype. These are canonical genes well known for their definitive role in defense pathways [66,67]. Many other stress related genes were also found to be more abundant in resistant genotype. Some of the examples are putative imbibition protein, aquaporin, and DNAJ-like protein. Although aquaporin is known to be induced in response to abiotic stresses [95] its role in patho-stress remains to be investigated. Plant pathogenesis related genes like cationic peroxidase, disease resistance related protein DRRG 49C, pathogen and wound-inducible antifungal protein CBP20 and pathogenesis related protein were also represented more in the resistant genotype. Cationic peroxidase has been shown to be associated with incompatible plant-pathogen interaction [96]. It is known that ECM/cell wall modifying enzymes play important role against invading pathogens. Genes encoding many such enzymes, for example, callose synthase was present in the resistant genotype. Furthermore, brassinosteroid regulated protein, BRU1 was present more in the resistant genotype. Domain search of BRU1 showed that it has xyloglucan endotransglycosylase activity and is involved in cell wall remodeling. Selenium binding proteins were found to be more abundant in the incompatible genotype suggesting their possible role in imparting plant disease resistance. Studies have shown that overexpression of a homologue of mammalian selenium binding protein in rice results in enhanced resistance to blast fungus and bacterial blight [97]. Nonaspanin, an endomembrane protein, involved in intracellular vesicular trafficking was present specifically in the resistant genotype. Further, presence of ESTs encoding proteins like narbonin, cupin domain protein and AKIN gamma specifically in the resistant genotype is indicative of their possible role in pathogen stress. While narbonin is known to be a seed storage protein [98], cupin domain proteins have diverse functional roles in seed germination, seedling development and modification of cell wall carbohydrates [99], however, their role in pathogen stress is yet to be elucidated.

It was intriguing that most of the genes reported, earlier, to be involved in auxin signaling pathway were specific to susceptible genotype, thereby suggesting a possible role of auxin signalling in compatible plant-pathogen interaction. Such genes included auxin-induced putative aldo/keto reductase family protein, aux/IAA protein and putative auxin-induced protein. However, auxin responsive SAUR protein was present in both the genotypes but with a much higher frequency in susceptible one. Auxin-regulated dual specificity cytosolic kinase was the only auxin-responsive gene found in the resistant genotype. A contemporary study has suggested the involvement of auxin signaling pathway in the susceptibility of a plant to pathogen attack [100]. Many other ESTs were also found to be more redundant in susceptible genotype. These include ESTs encoding chlorophyll a/b binding protein, cystatin and branched chain amino acid (BCAA) aminotransferase. Cystatin belongs to cysteine proteinase inhibitor superfamily and has been reported to have antifungal activity [89]. BCAA aminotransferase serves in detoxification mechanism that maintains the pool of free branched chain amino acids at low and non toxic levels under dehydration [101]. *Fusarium *grows in the vascular system of the plant and the mycelial growth of the fungus may cause blockage of vascular tissue, which mimics the symptoms of dehydration and therefore, may lead to the accumulation of BCAA aminotransferase. Interestingly, we found many genes that have never been implicated in plant stress response, for example, deoxyhypusine synthase (DHS), which was found to be present in the susceptible genotype. Recent studies have shown that the silencing of DHS delays ripening in tomato [102]. Ripening often leads to weakening of cell wall and by doing so it may predispose a plant to pathogen attack. Of very highly abundant contigs with total number of ESTs exceeding 20, thereby inferring their strong role in stress response, we found that genes encoding putative extensin, putative senescence-associated protein and RNA-binding region RNP-1 were more abundant in resistant genotype. On the other hand, genes encoding pfkB-type carbohydrate kinase known to be involved in preservation of intracellular adenylate pools and alcohol dehydrogenase 1 were more abundant in the susceptible genotype.

Next, it was of interest to find out the relationship between the genotype specific EST abundance with their expression pattern during incompatible interaction. Comparison of microarray expression data with the origin of the respective genes revealed the following three types of correlations. Firstly, genes specific to or more abundant in the resistant genotype like those encoding chalcone reductase, DRRG 49C, cytochrome P450, cytochrome P450 monooxygenase CYP83G1, peroxidase, putative kinetochore protein, GOS11 and serine/threonine kinase showed induction during incompatible plant pathogen interaction. Secondly, genes which were represented only in the susceptible genotype did not show any significant expression in the incompatible interaction. These included drought responsive element binding protein, Calcium-binding EF-hand, Targeting for Xklp2 and Protein kinase TonB box. Thirdly, many genes which were either absent or represented to a lesser extent in the resistant genotype but abundant in the susceptible one were found to be downregulated and included putative ripening related protein, putative ADP, ATP carrier-like protein, probable pyridoxal biosynthesis protein PDX1 and triosephosphate isomerase.

### Identification of putative new regulators of plant immune response

Although biological processes including immune response are controlled by intricate regulatory networks of gene expression that is thought to be regulated by many regulatory genes, only few such genes have been reported till date. While exploring the gene expression patterns during chickpea-*Fusarium *interactions, we identified several novel regulatory genes specifically showing differential expression of transcripts, which strongly suggests putative functions for them in plant immune response. These include ESTs encoding putative protein kinase APK1A, SAR DNA-binding protein-1, BEL1-like homeodomain transcription factor, NAC domain containing protein, RabGAP/TBC and GTP-binding protein. Protein kinase cascades are critical for regulating plant defense systems against various kinds of pathogens and operate as efficient signal transmission networks linking extracellular stimuli with intracellular targets. The upregulation of APK1 strongly suggests its possible role in defense signaling. This protein was identified in Arabidopsis, however, its role has not yet been identified [103]. The expression of BEL1 and SAR DNA-binding protein-1 was found to be downregulated. BEL transcription factor plays role in pattern formation in ovules [104], SAR DNA binding protein is known to have multifunctional roles in chromatin organisation and ribosome biogenesis [105] while NAC domain protein is implicated in developmental processes. Thus, it will be of great interest to elucidate their role in plant immunity. The expression of RabGAP/TBC and GTP-binding protein was induced at 24 h after *Fusarium *infection suggesting their possible involvement in plant defense. A gene of RabGAP class has been shown to be involved in spermatogenesis in rats [106], however, there is no report regarding their role in immune response. These results support the existence of different regulatory systems that operates in vascular wilt inducible root specific immune gene expression during plant defense. This divergence may be attributed to the specific interaction of a host to one of the specific pathogen of many that it encounters.

## Conclusion

Genetic make up of any organism results from a subset of genes that it inherits. The regulated expression of genes is tightly controlled and shaped by the transcription machinery of the cell. Therefore, development of genotype dependent organ specific kinetically controlled functional ESTs, identification of gene families and their associated transcriptomic and proteomic studies would reveal more and more yet unidentified sense and antisense transcripts; and transcript associated SNPs. This approach would essentially unravel the molecular basis of specificity at individual level that ultimately dictates the cellular physiology leading to the phenome variation.

Our study is directed towards creating a gene resource (*Ca*EST database) of chickpea for the systematic analysis of genotype-dependent organ-specific stimuli-responsive gene signatures that would provide an initial platform for gene discovery and functional genomics of this third most important but understudied food legume. This approach may be used in future to dissect diverse and overlapping biochemical pathways encompassed by the identified transcripts at individual level. This will also be important in long-term efforts to develop faithful, quantitative models for plant processes. The efficiency of subtracted genotype specific immune responsive EST sequencing is especially reflected in the number of new genes identified in this study, which is still moderate in size. A total of 2013 unigenes are reported that characterize the immune responsive transcripts of this important legume, of which approximately twelve percent represent new chickpea genes hitherto undiscovered. Our discovery of a large number of SNPs in expressed sequences should allow the genetic mapping of many genes underlying agricultural characteristics in chickpea. Although WR315 and JG62 may be presumably quite similar to one another at the nucleotide sequence level, we find they are equally different to each other in their transcript profile. In addition, the use of this approach in our present study led to the identification of 807 genes of unknown function now assigned to the immune response pathway with an organ specific localization, opens up a new area of investigation wherein the combining of our *Ca*Unigene dataset with other databases can be used to draw relationships at system level. The results identified a set of 649 non-cannonical stress responsive genes which has never been associated with immune response. Further, homology search of the derived amino acid sequence data from the present study provides a firm indication of a number of components of the root to which function is still to be ascribed. The ESTs identified in this study represent the major attempt so far to define chickpea transcriptome. The microarray analysis of immune-responsive transcriptome uncovers new regulators of host-pathogen interaction. Assigning a functional role to each of these new immune regulators will be challenging and quite valuable in order to know the physiological significance of plant immunity. Nevertheless, the sequence information of chickpea is unavailable and the number of functionally annotated ESTs is low at this time, as this number increases, the transcriptome data will be proven even more useful. The recent identification of immune responsive genes from other organisms would eventually lead to develop a comparative species-specific immune responsive transcriptome in which this report becomes one of the first attempts. This is an initial attempt in the direction that will be expanded upon during future transcriptomic studies of plant processes. Our future efforts will focus onto enriching *Ca*Unigene database and increasing the number of analyzed genes thereof, with an aim to draw a complete functional map of organ specific stimuli responsive transcriptome at individual level. Further, we will focus onto identifying the dynamics associated with the organ specific transcriptome towards cells metabolic and regulatory pathways at different physiological conditions shaping the phenome diversity.

## Methods

### Plant material and in planta infection

The chickpea (*Cicer arietinum L.*) seeds were sterilized with 70% ethanol and 0.1% HgCl_2 _followed by repetitive washing in autoclaved water. The seedlings were grown in sterile conditions in glass tubes containing *MS *basal medium solidified with 0.6% agar in an environmentally controlled growth room and maintained at 25 ± 2°C, 50 ± 5% relative humidity under 16 h photoperiod (270 *μ*mol m^-2 ^s^-1 ^light intensity). *Fusarium oxysporum ciceri race 1 *was grown in potato dextrose broth (PDB) at 28°C. The 3-week old seedlings were inoculated with *Fusarium *spore suspension at a concentration of 1 × 10^6^/ml while the control plants were treated with water. Root and collar (root and shoot junction) tissue was sampled as experimental material, harvested at 6, 12, 24, 48 h and 5 days after inoculation and stored at -80°C after quick-freezing in liquid nitrogen.

### Construction of cDNA libraries

Two suppression subtracted cDNA libraries were constructed, one from a susceptible (JG-62) and the other from a resistant (WR-315) genotype using PCR select cDNA subtraction kit (Clontech, CA). Total RNA was isolated by GITC method as described earlier [107] from uninfected seedlings (used as driver) and *Fusarium *infected seedlings (used as tester) collected at different time points as mentioned above and subsequently combined. The poly (A)^+ ^RNA from the pooled total RNA was purified by using Dyna beads oligo (dT) (Dynal biotech, USA). The cDNAs were cloned into pGEMT vector (Promega, Wisconsin) and transformed into E. coli cells (DH5ά). The individual clones were picked up and grown in deep well plates in 2× YT media containing ampicillin (75 μg/ml) at 37°C for 16-18 hours under shaking at 250 rpm. All individual clones were stored in 96 well U bottom plates by mixing 220 μl of grown culture and 30 μl of 80% glycerol for long term storage.

### Sequencing, processing and assembly of ESTs into contigs

Plasmid DNAs from both the subtracted cDNA libraries were isolated and purified by Perfectprep Plasmid 96 Vac Direct bind kit according to the manufacturer's instructions (Eppendorf, Germany). The quality of the plasmid DNAs was analyzed by 0.8% agarose gel electrophoresis and the good quality plasmids having appropriate DNA concentration and free from any mix up were selected for sequencing. The individual plasmids were sequenced using the BigDye terminator cycle sequencing kit (Perkin Elmer, Applied Biosystems) with M13 forward and reverse primers for 5' and 3' single pass sequencing respectively, in ABI Prism 3700 sequencer (Applied biosystems, CA). Sequence base calls were made using Phred [108] with a quality cutoff of 15. Vector filtering was performed using the cross match program (P. Green, ) followed by trimming of low quality sequences. The sequences were individually inspected for chimeras, short reads, *E. coli *and mitochondrial sequences which were subsequently removed. The processed ESTs with 100 bases or longer were assembled into contigs by CAP3 programme using standard parameters [20]. The final assembly of contigs and singletons constituted the chickpea gene index. All EST sequences have been submitted to GenBank [GenBank: GR911733 to GR918004].

### Blast analysis, annotation and comparison

The EST contig and singleton sequences were annotated for homology using BLASTX and BLASTN algorithms against non redundant protein and nucleotide databases respectively. For BLASTX, an e-value cut-off of 10^-15 ^was used and the sequences with e-value below this cutoff were then subjected to BLASTN analysis with e-value cutoff of 10^-20^. In addition, BLASTN was used to compare the chickpea sequences from this study to a database of legume sequences. This database included sequences of *Lotus japonicus *(gene index release 3.0); *Medicago trunculata *(gene index release 8.0); *Glycine max *(gene index release 12.0) and *Phaseolus vulgaris *(gene index release 1.0) from The Institute for genomic Research (TIGR; Quackenbush et al, 2001) and *Arachis hypogea, Cajanus cajan, Pisum sativum *and *Robinia pseudoacacia *available from NCBI taxonomy browser . The following criteria were used in stand-alone BLASTN comparison: (1) exact match bp = 11; (2) e-value ≤ 10^-5 ^and (3) DNA identity ≥ 80% and 90%. Further, TBLASTX (with e-value cutoff of 10^-15^) was used for comparing chickpea sequences to GenBank's EST_others database for the identification of chickpea specific sequences. Sequences were additionally functionally characterized in the context with the Gene Ontology (GO), Metacyc and COG. Cross species comparison of stress responsive transcriptome was carried out by searching each gene of unigene set against all classes of the stress responsive gene entries in the GO database . Genes which had a match in more than one group of stress responsive genes were categorized as ubiquitous and those which were earlier known to be involved in *Fusarium *stress as found from the fungal responsive group in the GO database were categorised as canonical. Remaining of the genes which did not show any match in any of the stress responsive gene groups in the GO database were classified as non canonical.

### Identification of gene families using single-linkage clustering

In order to identify gene families, the chickpea contigs and singletons were combined into a single dataset. TBLASTX with e-value cutoff of 10^-15 ^was used to compare the dataset against itself. Sequences with at least one sequence in common in their BLAST reports were combined into a putative gene family as described. [109].

### Identification of SNPs

For the identification of SNPs, the ace file output of CAP3 programme was used as input to the PolyBayes SNP detection program along with the base values assigned by Phred for each of the contigged sequences. Perl scripts were used to parse the PolyBayes output file. The cutoff value for the probability of SNP was put at 0.99. Only base change mutations were considered in order to avoid any discrepancy in the results due to any error in the alignment. The SNPs were further divided into high-quality if minimum of two sequences from each genotype showed the same base change and low-quality if minimum of two sequences from one genotype and one from the other showed the same base change.

### Construction of cDNA microarray

The cDNA microarray setup consisted of 8098 probes corresponding to 2013 unigenes. The cDNA clones from both susceptible and resistant libraries were amplified by performing colony PCR (1× PCR buffer, 1.5 mM MgCl_2_, 5 units Taq DNA polymerase [Applied biosystems, USA], 0.5 mM dNTPs [Fermentas, Canada], 0.2 μM M13 primer [Sigma], 20 ng of template DNA) in 96 well format. The PCR amplified products were purified using Perfectprep PCR clean up kit as per manufacturer's instructions (Eppendorf, Germany). The quality of the PCR products was checked on 1.0% agarose gels. Five microlitres of the PCR products were reorganized on the 384-well printing plates with 5 μl of 100% DMSO and printed in duplicates on the poly-L-lysine coated slides (Sigma) using a high throughput arrayer (Arrayer ESI, SDDC2) followed by UV cross linking. The spiking and other controls such as DMSO, SSC, water and previously cloned chickpea genes like 18S rRNA and actin were also printed at different locations in the chickpea microarray which were used as negative and positive controls.

### Sample preparation, labeling and microarray hybridization

In order to find out the differential gene expression during chickpea-*Fusarium *incompatible interaction, root tissue sample were collected from *Fusarium *infected WR 315 seedlings after 24 h of infection. Water treated WR 315 plants were taken as control. Two biological replicates were done. Total RNA was isolated using Trizol reagent (Invitrogen, CA). 6 μg of the total RNA was used for cDNA synthesis using indirect TSA labeling and detection kit Micromax (PerkinElmer, Boston, MA). The RNA isolated from uninfected and infected tissue was labeled with flourscein and biotin, respectively. The labeled cDNA was purified using microcon YM 100 columns (Millipore, Bedford, MA). The purified flourscein and biotin labeled cDNAs were mixed and hybridized to the microarray slides in hybridization chambers (Corning, USA) at 65°C water bath for 16 hours. The slides were washed for 10 minutes in 0.5× SSC and 0.01%SDS, 10 minutes in 0.06× SSC and 0.01%SDS and for 5 minutes in 0.06× SSC. The slides were subsequently processed for replacement of flourscein with Cy3 and biotin with Cy5 as per manufacturer's instructions. The experiment was carried out in duplicate.

### Scanning and data analysis

Micraoarrays were scanned using Scan array 5000 scanner (PerkinElmer, MA) to produce two separate tiff images. Spot finding and quantification of the spots were done by using Scan array express software (PerkinElmer, MA). Spots appearing bad due to poor morphology, high local background and bubbles were flagged off and were excluded from further analysis. Spots with both channel intensities less than 500 were also filtered out. Spots were quantified using an adaptive method. Avadis software (PerkinElmer, MA) was used for further data transformation which consisted of background correction and normalization. For background correction, local background intensity of each spot was subtracted from its foreground intensity value. Due to non linearity of the data, intensity dependent Lowess normalization was applied. Cy5/Cy3 signal ratio was also calculated. Although, indirect TSA labeling was performed to generate the flourscent labeled probe that obviates the need for control dye swap experiments, initially dye swap experiment was performed for checking the reciprocity of the gene signals that showed high correlation between the original and dye swap slides. Cross slide one class t-test with Benjamini and Horchberg FDR multiple correction was performed on the four replicate data points for each clone. P-value of 0.05 and fold induction of 2.5 was used as a limit for statistically significant differences in the expression. The resulting differentially expressed gene list was uploaded for hierarchical clustering.

### RNA gel blots

Validation of the microarray results was done by northern analysis of a few transcripts. For this, total RNA was extracted from root tissue of water treated and *Fusarium *challenged plants collected at 24 hours after treatment using trizol reagent (Invitrogen). 20 μg of RNA was denatured in 50% formamide, 17% formaldehyde, and 10% MOPS buffer (200 mM MOPS, pH7.0, 50 mM sodium acetate and 1 mM EDTA) at 65°C for 5 min and was separated on 1.2% agarose gel containing 22 M formaldehyde in MOPS buffer, transferred to GenScreen plus membrane (Hybond-N, Amersham Biosciences) by downward capillary blotting in 20× SSC. The DNA probes were prepared by random primer labeling and purified by passing through Sephadex G-50 column as described in Sambrook et al., 2001 [105]. After 2 hrs of prehybridization in 0.3 M Nacl, 5% formamide, 10% dextran sulfate and 1% SDS, at 42°C, the radiolabeled probe was added and hybridization was carried out at the same temperature for 16-18 h. After stringent washing, the blot was exposed to X ray film (Kodak, Rochester, NY).

## List of abbreviations

EST: (Expressed sequence tags); SSR: (simple sequence repeats); SNP: (single nucleotide polymorphism); BLAST: (basic local alignment and search tool); KOG: (eukaryotic orthologous groups); TCA: (tricarboxylic acid cycle); ADH: (alcohol dehydrogenase); GAPDH: (glyceraldehyde3-phosphate dehydrogenase); PEP: (phosphoenol pyruvate); PGIP: (Polygalactouronase inhibiting proteins); PR: (pathogenesis related); NDK: (nucleoside diphosphate kinase); ROS: (reactive oxygen species); PAL: (phenyl ammonia lyase); SAUR: (small auxin up RNA); DRRG: (disease response regulated gene); (DHS): deoxyhypusine synthase; FDR: (false discovery rate); LOWESS: (locally weighted scatterplot smoothing).

## Authors' contributions

NA, DG, AD, NC and SC designed the research. NA carried out plasmid isolation, sequencing, microarray experiments and all analysis. DG constructed the subtracted libraries, performed sequencing and RNA gel blot. PB and SB performed all *in silico *analysis. NA, PB and SB developed the *Ca*EST, *Ca*Unigene and *Ca*SNP databases. GN and MKM carried out plasmid isolation and sequencing. GN, NA and SC designed the microarray. NA, PB and SC analyzed the data. SC conceived the study and coordinated. NA, NC and SC wrote the manuscript. All authors have read and approved the final manuscript.

## Supplementary Material

Additional file 1 **Annotation of *Ca*EST contigs**. Table showing summary of *Ca*EST contigs including contig length, total and genotype specific ESTs in each contig and functional annotation based on BLASTX and BLASTN. Click here for file

Additional file 2 **Annotation of *Ca*EST singletons**. Table showing Summary of *Ca*EST singletons including their length and functional annotation based on BLASTX and BLASTN. Click here for file

Additional file 3 **Functional class annotation of *Ca*Unigenes**. Table listing functional annotation and assignment of *Ca*Unigenes into different functional classes using BLAST, GO, MetaCyc and COG. Click here for file

Additional file 4 **Gene family identification**. Table listing chickpea gene families identified by single linkage clustering. Click here for file

Additional file 5 **Genotype dependent *Ca*SNP identification**. Table listing identification of single nucleotide polymorphisms (SNPs) from chickpea genotypes. Click here for file

Additional file 6 **Microarray analysis of *Ca*ESTs in resistant genotype**. Table showing list of chickpea genes showing differential expression (upregulated and downregulated) in response to *Fusarium *infection at 24 hrs post inoculation during incompatible interaction. Click here for file
