# Supplementary material for: Comparative analyses of genotype dependent expressed sequence tags and stress-responsive transcriptome of chickpea wilt illustrate predicted and unexpected genes and novel regulators of plant immunity
Source: BMC Genomics. 2009 Sep 5;10:415. doi: 10.1186/1471-2164-10-415 (PMC2755012; doi:10.1186/1471-2164-10-415)
Supplement: Additional file 1 — Annotation of CaEST contigs. Table showing summary of CaEST contigs including contig length, total and genotype specific ESTs in each contig and functional annotation based on BLASTX and BLASTN. [file 1471-2164-10-415-S1.pdf]

Additional file 1- Summary of *Ca* EST contigs including contig lenth, total and genotype specific ESTs in each contig and functional annotation based on BLASTX and BLASTN.

Contig Number	Contig Length (bp)	Contig Length New (bp)	Total Number of ESTs in Contig	Number of ESTs from JG-62 Genotype[a]	Number of ESTs from WR-315 Genotype[b]	Accession number[c]	Functional Annotation[d]	E-value[e]
Contig1	376	329	3	3	0	gb|AC171534.4|	Medicago truncatula chromosome 7 clone mth2-90a20, complete sequence	3E-26
Contig2	463	437	7	7	0	gb|ABE86405.1|	hypothetical protein MtrDRAFT_AC156827g15v2 [Medicago truncatula]	3E-49
Contig3	248	224	3	3	0	gb|ABE93138.1|	Amino acid/polyamine transporter II [Medicago truncatula]	5E-28
Contig4	455	429	2	2	0	gb|ABP03302.1|	Zinc finger, C2H2-type [Medicago truncatula]	2E-73
Contig5	295	237	3	3	0	dbj|BAA02115.1|	GTP-binding protein [Pisum sativum] prf||2001457G GTP-binding protein	3E-14
Contig6	546	520	8	7	1	sp|P62302|RS13_SOYBN	40S ribosomal protein S13 gb|AAS47510.1| ribosomal protein S13 [Glycine max]	7E-79
Contig7	400	355	2	2	0	dbj|AP006137.1|	Lotus japonicus genomic DNA, chromosome 5, clone:LjT37K17, TM0239, complete sequence	1E-10
Contig8	319	295	3	3	0	ref|XP_380605.1|	hypothetical protein FG00429.1 [Gibberella zeae PH-1]	6E-16
Contig9	330	285	3	2	1	gb|ABE80121.1|	Pyruvate kinase [Medicago truncatula] gb|ABE89087.1| Pyruvate kinase [Medicago truncatula]	8E-41
Contig10	584	554	25	21	4	emb|CAA09040.1|	glyceraldehyde 3-phosphate dehydrogenase, cytosolic [Cicer arietinum]	3E-90
Contig11	416	390	2	2	0	gb|ABE79089.2|	Protein of unknown function DUF676, hydrolase-like [Medicago truncatula]	3E-47
Contig12	515	476	4	3	1	emb|CAN79806.1|	hypothetical protein [Vitis vinifera]	4E-37
Contig13	456	430	4	2	2	gb|ABE88889.1|	Protein of unknown function DUF567 [Medicago truncatula]	6E-47
Contig14	382	335	8	6	2	gb|AAT01416.1|	translation initiation factor 5A [Tamarix androssowii]	1E-58
Contig15	389	342	3	3	0	gb|AF537102.1|	Plasmodiophora brassicae 16S ribosomal RNA gene, partial sequence; mitochondrial gene for mitochondrial product	0.00003
Contig16	388	341	3	3	0	gb|ABO82817.1|	Protein of unknown function DUF668 [Medicago truncatula]	9E-24
Contig17	454	430	4	4	0	gb|ABE93398.1|	RNA-binding region RNP-1 (RNA recognition motif); HMG-I and HMG-Y, DNA-binding [Medicago truncatula]	2E-11
Contig18	460	436	2	2	0	emb|CAN70526.1|	hypothetical protein [Vitis vinifera]	2E-69
Contig19	385	338	5	4	1	emb|CAN82003.1|	hypothetical protein [Vitis vinifera]	3E-35
Contig20	521	465	5	5	0	dbj|BAE71262.1|	putative protein kinase APK1A [Trifolium pratense]	5E-49
Contig21	477	453	3	3	0	gb|AAR30118.1|	putative histidine phosphotransferase HPT1p [Gibberella moniliformis]	5E-36
Contig22	335	335	2	2	0	gb|ABE83191.2|	Zinc finger, CCHC-type [Medicago truncatula]	7E-32

Contig23	452	428	3	3	0	gb|AAC28907.1|	phaseolin G-box binding protein PG2 [Phaseolus vulgaris]	4E-37
Contig24	263	235	2	2	0	gb|ABO80586.1|	Amidase [Medicago truncatula]	3E-22
Contig25	486	460	10	8	2	gb|AAC49902.1|	diadenosine 5',5'''-P1,P4-tetraphosphate hydrolase [Lupinus angustifolius]	2E-63
Contig26	179	153	2	2	0	ref|XP_389462.1|	hypothetical protein FG09286.1 [Gibberella zeae PH-1]	9E-22
Contig27	338	314	5	3	2	emb|CAB61749.1|	putative water channel protein [Cicer arietinum]	1E-29
Contig28	359	335	2	2	0	gb|AAT58365.1|	GMPase [Medicago sativa]	8E-31
Contig29	402	376	2	2	0	gb|DQ251457.1|	Siniperca chuatsi transposase mRNA, partial cds	0.00003
Contig30	705	658	8	3	5	sp|Q03460|GLSN_MEDSA	Glutamate synthase [NADH], chloroplast precursor (NADH-GOGAT) gb|AAB46617.1| NADH-glutamate synthase [Medicago sativa]	7E-61
Contig31	461	435	4	4	0	ref|NP_565451.1|	nucleic acid binding / zinc ion binding [Arabidopsis thaliana] gb|AAM14872.1| Expressed protein [Arabidopsis thaliana] gb|ABD19677.1| At2g19385 [Arabidopsis thaliana]	1E-43
Contig32	359	335	9	8	1	ref|NP_566599.1|	unknown protein [Arabidopsis thaliana] gb|AAK93587.1| unknown protein [Arabidopsis thaliana] gb|AAM14351.1| unknown protein [Arabidopsis thaliana] gb|AAM65658.1| unknown [Arabidopsis thaliana]	2E-24
Contig33	384	340	2	2	0	gb|EAY73868.1|	hypothetical protein OsI_001715 [Oryza sativa (indica cultivar-group)]	3E-30
Contig34	462	436	3	3	0	emb|CAE01583.2|	OSJNBa0068L06.9 [Oryza sativa (japonica cultivar-group)] emb|CAH65755.1| OSIGBa0123D13.4 [Oryza sativa (indica cultivar-group)] gb|EAY92749.1| hypothetical protein OsI_013982 [Oryza sativa (indica cultivar-group)] gb|EAZ29424.1| hypothetical protein OsJ_012907 [Oryza sativa (japonica cultivar-group)]	6E-42
Contig35	464	438	4	4	0	emb|AM706411.1|	Eristalis tenax partial mRNA for hypothetical protein (ORF1), isolate 3	0.0001
Contig36	647	606	7	7	0	gb|ABE89691.1|	Pectinesterase [Medicago truncatula]	1E-28
Contig37	444	420	3	3	0	ref|XP_382177.1|	hypothetical protein FG02001.1 [Gibberella zeae PH-1]	1E-73
Contig38	462	438	3	3	0	sp|P51062|CAPP_PEA	Phosphoenolpyruvate carboxylase (PEPCase) (PEPC) dbj|BAA10902.1| phosphoenolpyruvate carboxylase [Pisum sativum]	1E-69
Contig39	371	324	7	6	1	gb|AAS67005.1|	Phosphoenolpyruvate carboxylase [Glycine max]	3E-31
Contig40	458	396	11	5	6	emb|CAI48073.1|	60S ribosomal protein L37a [Capsicum chinense]	3E-46
Contig41	179	156	2	2	0	emb|AM422870.1|	Fagopyrum esculentum gFeAP9 gene for aspartic proteinase, exons 1-13	0.0007
Contig42	456	432	4	4	0	emb|CAJ13713.1|	hypothetical protein [Capsicum chinense]	3E-21
Contig43	421	379	7	6	1	emb|CAA62082.1|	cytochrome p450 [Arabidopsis thaliana]	4E-47

Contig44	376	352	2	2	0	emb|AJ749797.1|	Photobacterium damselae subsp. piscicida trpB gene for putative transposase, clone pRDA16	0.0001
Contig45	404	383	2	2	0	emb|CAN77029.1|	hypothetical protein [Vitis vinifera]	2E-55
Contig46	304	278	2	2	0	ref|XP_386464.1|	hypothetical protein FG06288.1 [Gibberella zeae PH-1]	9E-40
Contig47	459	435	4	4	0	gb|AAN74635.1|	DEAD box RNA helicase [Pisum sativum] gb|AAR97917.1| DEAD box RNA helicase [Pisum sativum]	4E-64
Contig48	460	434	3	3	0	gb|EAZ32237.1|	hypothetical protein OsJ_015720 [Oryza sativa (japonica cultivar-group)]	4E-32
Contig49	339	292	8	8	0	emb|CAD70620.1|	branched-chain amino acid aminotransferase-like protein [Cicer arietinum]	2E-37
Contig50	544	520	5	2	3	sp|Q9M5L0|RL35_EUPES	60S ribosomal protein L35 gb|AAF34800.1|AF227980_1 60S ribosomal protein L35 [Euphorbia esula]	5E-52
Contig51	343	317	2	1	1	ref|XP_385657.1|	hypothetical protein FG05481.1 [Gibberella zeae PH-1]	2E-18
Contig52	484	460	10	10	0	gb|AAD32141.1|AF123503_1	Nt-gh3 deduced protein [Nicotiana tabacum]	3E-72
Contig53	637	595	21	18	3	ref|NP_173390.1|	pfkB-type carbohydrate kinase family protein [Arabidopsis thaliana] gb|AAF79436.1|AC025808_18 F18O14.35 [Arabidopsis thaliana] gb|AAF98405.1|AC024609_6 Unknown protein [Arabidopsis thaliana] gb|AAO44087.1| At1g19600 [Arabidopsis thaliana] dbj|BAE99744.1| putative ribokinase [Arabidopsis thaliana]	3E-87
Contig54	384	358	4	4	0	dbj|BAA13032.1|	phosphoribosylanthranilate transferase [Pisum sativum]	1E-61
Contig55	491	447	9	9	0	emb|CAN80511.1|	hypothetical protein [Vitis vinifera]	3E-46
Contig56	412	388	3	2	1	gb|AAF08537.1|AF191098_1	nucleoside diphosphate kinase [Pisum sativum]	3E-64
Contig57	459	437	3	3	0	gb|ABO81056.1|	Ubiquitin-conjugating enzyme, E2 [Medicago truncatula]	3E-42
Contig58	429	382	2	2	0	emb|CAN71158.1|	hypothetical protein [Vitis vinifera]	8E-54
Contig59	431	384	3	3	0	emb|CAG28692.1|	hypothetical protein [Gibberella fujikuroi]	2E-27
Contig60	493	433	6	5	1	ref|XP_001257542.1|	conserved hypothetical protein [Neosartorya fischeri NRRL 181] gb|EAW15645.1| conserved hypothetical protein [Neosartorya fischeri NRRL 181]	4E-29
Contig61	634	588	8	8	0	emb|CAN81116.1|	hypothetical protein [Vitis vinifera]	1E-47
Contig62	456	425	2	2	0	ref|NP_564424.1|	PHD finger family protein [Arabidopsis thaliana] sp|Q9C810|Y1342_ARATH PHD finger protein At1g33420 gb|AAG51204.1|AC051630_1 hypothetical protein; 47104-44821 [Arabidopsis thaliana] gb|AAK59559.1| unknown protein [Arabidopsis thaliana] gb|AAK93738.1| unknown protein [Arabidopsis thaliana]	4E-37
Contig63	428	382	2	2	0	emb|CAN75568.1|	hypothetical protein [Vitis vinifera]	3E-40

Contig64	418	394	2	2	0	ref|NP_565528.1|	beta-hydroxyacyl-ACP dehydratase, putative [Arabidopsis thaliana] gb|AAD23619.2| putative beta-hydroxyacyl-ACP dehydratase [Arabidopsis thaliana] gb|AAM78110.1| At2g22230/T26C19.11 [Arabidopsis thaliana] gb|AAN72302.1| At2g22230/T26C19.11 [Arabidopsis thaliana]	4E-52
Contig65	572	548	8	6	2	emb|CAN69190.1|	hypothetical protein [Vitis vinifera]	1E-30
Contig66	621	579	18	10	8	sp|O81361|RS8_PRUAR	40S ribosomal protein S8 gb|AAC24583.1| 40S ribosomal protein S8 [Prunus armeniaca]	8E-91
Contig67	199	199	2	2	0	gb|ABN08080.1|	ATA15 protein, putative [Medicago truncatula]	2E-11
Contig68	592	571	7	7	0	emb|CAN73756.1|	hypothetical protein [Vitis vinifera]	3E-47
Contig69	325	293	2	2	0	gb|ABD33305.1|	hypothetical protein MtrDRAFT_AC158502g14v2 [Medicago truncatula]	2E-27
Contig70	654	574	16	6	10	emb|AJ608703.3|	Fusarium oxysporum f. sp. lycopersici six1 gene, fot5 gene, six2 gene, shh1 gene and ORF2 (partial)	1E-109
Contig71	467	425	9	9	0	emb|CAN72193.1|	hypothetical protein [Vitis vinifera]	3E-43
Contig72	233	189	5	5	0	ref|XP_382177.1|	hypothetical protein FG02001.1 [Gibberella zeae PH-1]	2E-27
Contig73	420	318	19	19	0	emb|AJ608703.3|	Fusarium oxysporum f. sp. lycopersici six1 gene, fot5 gene, six2 gene, shh1 gene and ORF2 (partial)	1E-96
Contig74	335	275	3	3	0	gb|AC195567.2|	Medicago truncatula chromosome 2 BAC clone mth2-22c17, complete sequence	0.022
Contig75	457	431	3	3	0	gb|DQ251457.1|	Siniperca chuatsi transposase mRNA, partial cds	0.00003
Contig76	400	347	2	2	0	gb|ABE78629.2|	Phosphoglucose isomerase (PGI) [Medicago truncatula]	3E-28
Contig77	509	459	28	17	11	emb|CAN78091.1|	hypothetical protein [Vitis vinifera]	1E-34
Contig78	484	460	3	3	0	gb|ABP02285.1|	hypothetical protein MtrDRAFT_AC126789g51v2 [Medicago truncatula]	2E-54
Contig79	424	398	3	3	0	emb|CAN59845.1|	hypothetical protein [Vitis vinifera]	3E-39
Contig80	377	353	8	8	0	gb|ABB29934.1|	acidic ribosomal protein P1a-like [Solanum tuberosum]	1E-18
Contig81	709	649	12	7	5	emb|CAA10284.1|	chlorophyll a/b binding protein [Cicer arietinum]	1E-113
Contig82	488	488	4	2	2	gb|EDN28454.1|	conserved hypothetical protein [Botryotinia fuckeliana B05.10]	1E-41
Contig83	473	447	4	2	2	gb|AAZ14831.1|	putative AP2-binding protein [Jatropha curcas]	5E-23
Contig84	656	632	14	6	8	dbj|BAD93605.1|	hypothetical protein [Cucumis melo]	1E-103
Contig85	596	554	9	5	4	dbj|BAE98181.1|	putative pyruvate decarboxylase [Fusarium oxysporum f. sp. lycopersici]	8E-63
Contig86	486	486	3	3	0	gb|ABE83899.1|	Serine/threonine protein kinase, active site [Medicago truncatula] gb|ABE89881.1| Serine/threonine protein kinase, active site [Medicago truncatula]	2E-86
Contig87	410	386	4	3	1	gb|ABE91660.2|	hypothetical protein MtrDRAFT_AC145219g51v2 [Medicago truncatula]	9E-54

Contig88	456	430	7	7	0	ref|XP_380875.1|	hypothetical protein FG00699.1 [Gibberella zeae PH-1]	2E-42
Contig89	318	318	3	3	0	emb|CAC36428.1|	mitogen activated protein kinase [Gibberella fujikuroi]	2E-50
Contig90	311	288	6	2	4	gb|ABE79479.1|	Ribosomal protein L10E [Medicago truncatula]	8E-44
Contig91	427	365	6	4	2	dbj|BAF31848.1|	nitrite reductase [Fusarium oxysporum]	3E-40
Contig92	314	296	2	2	0	emb|CU302348.1|	Medicago truncatula chromosome 5 clone mth2-45a24, COMPLETE SEQUENCE	1E-46
Contig93	233	196	6	4	2	emb|AJ534351.1|ABI534351	Agaricus bisporus partial mRNA for putative myosin heavy chain kinase (mhck gene), clone pm31	0.004
Contig94	423	388	3	3	0	ref|NP_001067438.1|	Os11g0199700 [Oryza sativa (japonica cultivar-group)] gb|ABA91945.2| VHS domain containing protein, expressed [Oryza sativa (japonica cultivar-group)] dbj|BAF27801.1| Os11g0199700 [Oryza sativa (japonica cultivar-group)]	2E-17
Contig95	228	207	2	1	1	dbj|BAA76430.1|	fructose-bisphosphate aldolase [Cicer arietinum]	2E-14
Contig96	239	215	2	2	0	emb|AJ749794.1|	Photobacterium damselae subsp. piscicida trpA gene for putative transposase and partial ORF1 DNA for hypothetical protein, clone pRDA13	0.000004
Contig97	528	528	6	6	0	emb|CAB76914.1|	60S ribosomal protein L6 [Cicer arietinum]	4E-75
Contig98	477	435	11	10	1	ref|NP_563815.1|	PSAO (photosystem I subunit O) [Arabidopsis thaliana] gb|AAK93637.1| unknown protein [Arabidopsis thaliana] gb|AAM14284.1| unknown protein [Arabidopsis thaliana] gb|AAM64918.1| putative 16kDa membrane protein [Arabidopsis thaliana] emb|CAD37939.1| photosystem I subunit O [Arabidopsis thaliana]	1E-53
Contig99	539	492	4	4	0	gb|AAK95391.1|	ribosomal protein L2 [Gossypium arboreum]	4E-31
Contig100	309	286	4	2	2	gb|EDN11149.1|	hypothetical protein HCAG_07602 [Ajellomyces capsulatus NAm1]	1E-16
Contig101	400	400	2	1	1	emb|CR954185.3|	Medicago truncatula chromosome 5 clone mth4-20m5, COMPLETE SEQUENCE	0.0001
Contig102	578	534	5	4	1	emb|CAN63147.1|	hypothetical protein [Vitis vinifera]	3E-60
Contig103	615	506	14	1	13	gb|AAQ18141.1|	poly(A)-binding protein C-terminal interacting protein 6 [Cucumis sativus]	6E-22
Contig104	447	447	4	3	1	gb|ABC68399.1|	cytochrome P450 monooxygenase CYP76O2 [Glycine max]	3E-53
Contig105	276	250	2	1	1	gb|ABE92718.1|	Proteasome component region PCI [Medicago truncatula] gb|ABO84469.1| Proteasome component region PCI [Medicago truncatula]	2E-36
Contig106	224	200	2	1	1	emb|CAN64127.1|	hypothetical protein [Vitis vinifera]	1E-17
Contig107	473	447	2	1	1	emb|CAN78160.1|	hypothetical protein [Vitis vinifera]	1E-35
Contig108	238	214	3	2	1	emb|CAN66103.1|	hypothetical protein [Vitis vinifera]	4E-16
Contig109	385	346	3	1	2	gb|ABP02189.1|	CBS [Medicago truncatula]	2E-22
Contig110	429	408	3	3	0	gb|AAZ32857.1|	unknown [Medicago sativa]	1E-43

Contig111	233	209	3	2	1	gb|ABE84970.1|	Pathogenesis-related transcriptional factor and ERF [Medicago truncatula]	6E-26
Contig112	393	375	4	4	0	gb|ABD32712.1|	Response regulator receiver; CCT [Medicago truncatula]	7E-29
Contig113	481	457	10	10	0	gb|DQ465767.1|	Sesbania drummondii clone SSH-14_01_F04_T3 mRNA sequence	0.008
Contig114	231	184	7	7	0	gb|ABE94388.1|	WD-40 repeat [Medicago truncatula] gb|ABO84189.1| WD40-like [Medicago truncatula]	4E-18
Contig115	416	390	2	1	1	gb|DQ459385.1|	Nicotiana tabacum serine/threonine kinase mRNA, partial cds	0.0005
Contig116	630	577	8	8	0	gb|AAL32037.2|AF439278_1	ethylene-responsive transciptional coactivator-like protein [Retama raetam]	5E-42
Contig117	319	295	3	3	0	ref|XP_958142.1|	hypothetical protein [Neurospora crassa OR74A] gb|EAA28906.1| hypothetical protein [Neurospora crassa]	8E-28
Contig118	391	366	3	1	2	emb|CAD33928.1|	tonoplast intrinsic protein [Cicer arietinum]	1E-31
Contig119	589	547	6	2	4	gb|AAT08648.1|	ADP-ribosylation factor [Hyacinthus orientalis]	2E-76
Contig120	287	261	2	2	0	gb|ABE91787.1|	Protein of unknown function DUF707 [Medicago truncatula]	3E-48
Contig121	491	450	4	1	3	emb|AM748509.1|	Vigna unguiculata partial mRNA for putative single-stranded nucleic acid binding R3H (MtrDRAFT_AC183371g11v1 gene), clone 54	0.00003
Contig122	327	280	11	7	4	gb|DQ406807.1|	Ceratitis capitata clone 17a mRNA sequence	0.000006
Contig123	762	712	25	18	7	emb|AJ608703.3|	Fusarium oxysporum f. sp. lycopersici six1 gene, fot5 gene, six2 gene, shh1 gene and ORF2 (partial)	1E-168
Contig124	482	436	9	6	3	emb|AJ250814.1|FOX250814	Fusarium oxysporum f. sp. lycopersici insertion sequence Foxy	0
Contig125	275	251	2	2	0	gb|AAL36394.1|	unknown protein [Arabidopsis thaliana] gb|AAQ89668.1| At1g77230 [Arabidopsis thaliana] dbj|BAD43411.1| hypothetical protein [Arabidopsis thaliana] dbj|BAD43428.1| hypothetical protein [Arabidopsis thaliana]	5E-32
Contig126	486	447	4	1	3	ref|XP_386197.1|	ADT_NEUCR ADP,ATP CARRIER PROTEIN (ADP/ATP TRANSLOCASE) (ADENINE NUCLEOTIDE TRANSLOCATOR) (ANT) [Gibberella zeae PH-1]	3E-22
Contig127	395	371	5	3	2	gb|AAA34366.1|	ribosomal protein L41	8E-46
Contig128	345	321	2	2	0	gb|EAZ27896.1|	hypothetical protein OsJ_011379 [Oryza sativa (japonica cultivar-group)]	9E-48
Contig129	309	271	2	2	0	emb|CAC43238.1|	calcium binding protein [Sesbania rostrata]	4E-12
Contig130	478	436	3	3	0	gb|ABO82668.1|	Proteinase inhibitor I25, cystatin [Medicago truncatula]	3E-32
Contig131	312	288	2	2	0	emb|CAN72414.1|	hypothetical protein [Vitis vinifera]	5E-22
Contig132	271	229	3	3	0	gb|AC136840.24|	Medicago truncatula clone mth2-33n3, complete sequence	1E-12
Contig133	406	385	2	2	0	gb|AAF64040.1|AF228501_1	14-3-3-like protein [Glycine max]	5E-65
Contig134	423	349	8	3	5	gb|AAS18240.1|	enolase [Glycine max]	8E-58

Contig135	459	433	5	5	0	gb|ABE77912.1|	SAM (and some other nucleotide) binding motif [Medicago truncatula] gb|ABE79986.1| SAM (and some other nucleotide) binding motif [Medicago truncatula] gb|ABE83018.1| SAM (and some other nucleotide) binding motif [Medicago truncatula]	4E-70
Contig136	230	204	2	2	0	emb|CAN62771.1|	hypothetical protein [Vitis vinifera]	5E-25
Contig137	178	152	2	2	0	gb|BT009458.1|	Triticum aestivum clone wlsu2.pk0001.h3:fis, full insert mRNA sequence	0.0002
Contig138	209	185	3	2	1	emb|AM490066.1|	Dicentrarchus labrax mRNA for dicentrarchus labrax CC chemokine 2 (cc2 gene)	0.053
Contig139	385	361	3	2	1	gb|ABO80202.1|	hypothetical protein MtrDRAFT_AC139707g22v2 [Medicago truncatula]	1E-22
Contig140	474	432	4	3	1	emb|CAN69887.1|	hypothetical protein [Vitis vinifera]	7E-46
Contig141	459	435	2	2	0	gb|ABE77505.1|	DECOY (exp=-1; , putative [Medicago truncatula]	9E-53
Contig142	449	423	2	1	1	emb|CAN62146.1|	hypothetical protein [Vitis vinifera]	5E-32
Contig143	426	379	5	3	2	dbj|BAC81649.1|	glutathione S-transferase [Pisum sativum]	1E-52
Contig144	556	508	6	6	0	emb|CAA93759.1|	putative transposase [Tolypocladium inflatum]	6E-11
Contig145	451	427	3	2	1	emb|CAN80034.1|	hypothetical protein [Vitis vinifera]	2E-11
Contig146	126	102	2	1	1	gb|AY972077.1|	Synthetic construct RLS (RLS) gene, complete cds	0.0001
Contig147	459	435	3	3	0	gb|ABE83604.1|	PUG; Zinc finger, C2H2-type; UBA-like [Medicago truncatula] gb|ABE91517.1| PUG; Zinc finger, C2H2-type; UBA-like [Medicago truncatula]	2E-68
Contig148	455	381	2	1	1	gb|ABE81243.1|	Malic oxidoreductase [Medicago truncatula]	1E-61
Contig149	175	175	3	2	1	emb|CR954193.3|	Medicago truncatula chromosome 5 clone mth2-27a1, COMPLETE SEQUENCE	8E-10
Contig150	459	433	5	5	0	gb|AAM22748.1|	polyubiquitin 2 [Deschampsia antarctica]	3E-54
Contig151	578	500	13	13	0	ref|XP_388835.1|	hypothetical protein FG08659.1 [Gibberella zeae PH-1]	1E-18
Contig152	631	583	5	5	0	gb|AAS46232.1|	methionine sulfoxide reductase A [Populus trichocarpa x Populus deltoides]	8E-81
Contig153	187	145	3	3	0	emb|CAA10287.2|	glucan-endo-1,3-beta-glucosidase [Cicer arietinum]	2E-22
Contig154	448	424	5	2	3	gb|AAL11502.1|AF367442_1	NAD-dependent malate dehydrogenase [Prunus persica]	2E-63
Contig155	456	432	4	4	0	emb|CAN62476.1|	hypothetical protein [Vitis vinifera]	3E-51
Contig156	459	433	2	2	0	gb|ABE78557.1|	Oligosaccharyl transferase, STT3 subunit [Medicago truncatula] gb|ABO83793.1| Oligosaccharyl transferase, STT3 subunit [Medicago truncatula]	2E-48
Contig157	453	410	4	4	0	ref|NP_001045159.1|	Os01g0911200 [Oryza sativa (japonica cultivar-group)] dbj|BAD82429.1| putative ribophorin II precursor [Oryza sativa (japonica cultivar-group)] dbj|BAF07073.1| Os01g0911200 [Oryza sativa (japonica cultivar-group)]	8E-42

Contig158	357	336	2	2	0	emb|AM748481.1|	Vigna unguiculata partial mRNA for putative ATP synthase CF1 alpha subunit (atpA gene), clone 26	0.002
Contig159	446	422	3	3	0	gb|ABE86430.2|	Glycosyl transferase, family 20; Trehalose-phosphatase [Medicago truncatula]	1E-61
Contig160	454	428	4	4	0	dbj|BAE71188.1|	BEL1-like homeodomain transcription factor [Trifolium pratense]	1E-64
Contig161	452	426	2	2	0	gb|ABE86996.1|	Protein kinase [Medicago truncatula]	5E-65
Contig162	421	356	9	4	5	gb|ABE92863.1|	F-actin capping protein, alpha subunit [Medicago truncatula]	5E-23
Contig163	451	427	3	3	0	gb|ABE89232.1|	Inositol polyphosphate related phosphatase [Medicago truncatula]	2E-60
Contig164	450	423	2	2	0	ref|NP_566081.1|	pyridoxine 5'-phosphate oxidase-related [Arabidopsis thaliana] gb|AAD20168.2| expressed protein [Arabidopsis thaliana] gb|AAM63414.1| unknown [Arabidopsis thaliana] gb|ABD94073.1| At2g46580 [Arabidopsis thaliana]	1E-45
Contig165	575	548	9	6	3	gb|ABP02830.1|	H+-transporting two-sector ATPase, alpha/beta subunit, central region; H+-transporting two-sector ATPase, alpha/beta subunit, C-terminal [Medicago truncatula]	1E-48
Contig166	501	464	12	11	1	emb|CAN79713.1|	hypothetical protein [Vitis vinifera]	3E-38
Contig167	415	377	3	3	0	gb|AAL06496.1|AF412043_1	AT5g04420/T32M21_20 [Arabidopsis thaliana]	4E-17
Contig168	348	320	8	7	1	ref|XP_390479.1|	hypothetical protein FG10303.1 [Gibberella zeae PH-1]	3E-30
Contig169	453	429	4	3	1	emb|CR962130.3|	Medicago truncatula chromosome 5 clone mte1-63h20, COMPLETE SEQUENCE	2E-43
Contig170	322	278	6	5	1	ref|XP_387180.1|	hypothetical protein FG07004.1 [Gibberella zeae PH-1]	8E-18
Contig171	463	431	4	4	0	gb|AAC64109.1|	signal recognition particle 54 kDa subunit precursor [Pisum sativum]	1E-63
Contig172	452	405	2	2	0	gb|DQ251457.1|	Siniperca chuatsi transposase mRNA, partial cds	0.00003
Contig173	375	351	4	4	0	gb|ABP02356.1|	hypothetical protein MtrDRAFT_AC130802g5v1 [Medicago truncatula]	9E-56
Contig174	472	448	10	10	0	gb|ABE84246.1|	Chlorophyll A-B binding protein [Medicago truncatula] gb|ABP02413.1| Chlorophyll A-B binding protein [Medicago truncatula]	4E-66
Contig175	462	390	2	2	0	gb|ABE93060.2|	CD9/CD37/CD63 antigen [Medicago truncatula]	1E-13
Contig176	342	298	5	3	2	ref|NP_851141.1|	RNA recognition motif (RRM)-containing protein [Arabidopsis thaliana] gb|AAK91419.1| AT5g46250/MPL12_3 [Arabidopsis thaliana] gb|AAN18157.1| At5g46250/MPL12_3 [Arabidopsis thaliana]	6E-20
Contig177	453	427	6	6	0	emb|CAN82780.1|	hypothetical protein [Vitis vinifera]	8E-65
Contig178	294	252	4	4	0	gb|ABC94943.1|	squalene epoxidase [Medicago sativa]	2E-44
Contig179	465	439	2	2	0	gb|ABE93770.1|	Adenylosuccinate synthetase [Medicago truncatula]	6E-78
Contig180	662	612	5	5	0	gb|ABP49577.1|	oleate desaturase [Caragana korshinskii var. intermedia]	3E-98

Contig181	333	309	3	3	0	dbj|BAE61155.1|	unnamed protein product [Aspergillus oryzae]	2E-42
Contig182	386	362	2	2	0	ref|NP_193961.2|	carboxylic ester hydrolase [Arabidopsis thaliana]	9E-51
Contig183	440	416	2	2	0	gb|EAZ27173.1|	hypothetical protein OsJ_010656 [Oryza sativa (japonica cultivar-group)]	2E-38
Contig184	463	437	2	2	0	ref|XP_380754.1|	hypothetical protein FG00578.1 [Gibberella zeae PH-1]	2E-78
Contig185	462	436	2	2	0	gb|AC134322.25|	Medicago truncatula clone mth2-17i21, complete sequence	7E-28
Contig186	461	435	5	4	1	gb|ABC87913.1|	polygalacturonase precursor [Glycine max]	1E-75
Contig187	161	143	2	2	0	emb|CT029789.1|	Poplar cDNA sequences	0.62
Contig188	359	312	2	2	0	ref|XP_961514.1|	hypothetical protein [Neurospora crassa OR74A] sp|Q9C2P2|RS29_NEUCR 40S ribosomal protein S29 emb|CAC28832.1| probable ribosomal protein S29.e.A, cytosolic [Neurospora crassa] gb|EAA32278.1| hypothetical protein [Neurospora crassa]	7E-21
Contig189	360	313	3	3	0	gb|AAT58770.1|	putative polyprotein [Oryza sativa (japonica cultivar-group)]	1E-24
Contig190	531	507	6	1	5	emb|CAB50768.1|	cytochrome P450 [Cicer arietinum]	2E-71
Contig191	301	258	2	2	0	gb|AAT08753.1|	LRR [Hyacinthus orientalis]	3E-35
Contig192	462	436	2	2	0	ref|NP_568756.2|	ceramide kinase-related [Arabidopsis thaliana] gb|AAQ62904.1| ceramide kinase [Arabidopsis thaliana]	2E-41
Contig193	431	407	4	4	0	gb|ABN09112.1|	Haem peroxidase, plant/fungal/bacterial [Medicago truncatula]	3E-35
Contig194	431	405	2	2	0	gb|AAK11734.1|	serine/threonine/tyrosine kinase [Arachis hypogaea]	8E-65
Contig195	388	341	2	2	0	ref|XP_389229.1|	hypothetical protein FG09053.1 [Gibberella zeae PH-1]	9E-62
Contig196	408	384	2	2	0	ref|NP_568725.1|	ELO3 (ELONGATA 3); N-acetyltransferase/ catalytic/ hydrogen ion transporting ATP synthase, rotational mechanism / hydrogen ion transporting ATPase, rotational mechanism / iron ion binding [Arabidopsis thaliana] sp|Q93ZR1|ELP3_ARATH Elongator complex protein 3 (Protein ELONGATA 3) (Elongator component 3) gb|AAL07172.1| putative histone acetyltransferase [Arabidopsis thaliana] emb|CAI79647.1| elongator component [Arabidopsis thaliana]	2E-67
Contig197	342	301	5	5	0	emb|CAB50768.1|	cytochrome P450 [Cicer arietinum]	6E-40
Contig198	448	422	3	3	0	gb|ABE80069.1|	Root cap [Medicago truncatula]	2E-69
Contig199	451	427	5	4	1	gb|AAM64316.1|	multicatalytic endopeptidase complex, proteasome precursor, beta subunit [Arabidopsis thaliana]	1E-36
Contig200	434	410	2	2	0	gb|AY972077.1|	Synthetic construct RLS (RLS) gene, complete cds	0.0005
Contig201	453	429	3	3	0	emb|CAN62762.1|	hypothetical protein [Vitis vinifera]	2E-50
Contig202	391	365	2	2	0	gb|ABE79078.1|	Mitochondrial carrier protein [Medicago truncatula]	9E-61

Contig203	304	265	2	2	0	gb|AAV63565.1|	auxin-induced putative aldo/keto reductase family protein [Arachis hypogaea]	1E-17
Contig204	457	433	7	7	0	gb|ABE78019.1|	Concanavalin A-like lectin/glucanase [Medicago truncatula]	5E-44
Contig205	355	332	2	2	0	ref|NP_001042188.1|	Os01g0178000 [Oryza sativa (japonica cultivar-group)] dbj|BAB63467.1| putative aspartate aminotransferase [Oryza sativa (japonica cultivar-group)] dbj|BAF04102.1| Os01g0178000 [Oryza sativa (japonica cultivar-group)] gb|EAY72764.1| hypothetical protein OsI_000611 [Oryza sativa (indica cultivar-group)] gb|EAZ10762.1| hypothetical protein OsJ_000587 [Oryza sativa (japonica cultivar-group)]	8E-42
Contig206	665	617	81	19	62	gb|ABE82737.1|	RNA-binding region RNP-1 (RNA recognition motif) [Medicago truncatula]	4E-33
Contig207	280	254	3	3	0	ref|XP_391558.1|	hypothetical protein FG11382.1 [Gibberella zeae PH-1]	3E-26
Contig208	371	347	2	2	0	emb|CAM35498.1|	spermine synthase [Lotus japonicus]	1E-57
Contig209	313	271	4	4	0	emb|CAN79638.1|	hypothetical protein [Vitis vinifera]	2E-40
Contig210	457	431	3	3	0	ref|XP_384070.1|	hypothetical protein FG03894.1 [Gibberella zeae PH-1]	3E-55
Contig211	460	434	4	4	0	emb|AM706411.1|	Eristalis tenax partial mRNA for hypothetical protein (ORF1), isolate 3	0.0001
Contig212	459	433	2	2	0	sp|Q8LJS2|HDT1_SOYBN	Histone deacetylase HDT1 (Histone deacetylase 2a) (HD2a) (Nucleolar histone deacetylase HD2-p39) gb|AAN03465.1| nucleolar histone deacetylase HD2-P39 [Glycine max]	3E-31
Contig213	453	429	5	5	0	emb|CAI91291.1|	deoxyhypusine synthase [Crotalaria juncea]	5E-68
Contig214	464	438	3	3	0	gb|ABO79980.1|	hypothetical protein MtrDRAFT_AC138448g30v2 [Medicago truncatula]	2E-43
Contig215	129	106	2	2	0	gb|L46857.1|ALFCAD1B	Medicago sativa cinnamyl-alcohol dehydrogenase (cad1) mRNA, partial cds	1E-32
Contig216	425	399	2	2	0	gb|AAL06505.1|AF412052_1	At2g43970/F6E13.10 [Arabidopsis thaliana]	4E-29
Contig217	360	334	5	5	0	ref|NP_974856.1|	zinc finger (ZPR1-type) family protein [Arabidopsis thaliana]	3E-31
Contig218	666	619	12	10	2	gb|ABO78621.1|	Translation factor [Medicago truncatula]	3E-91
Contig219	440	414	3	2	1	gb|ABP02565.1|	General substrate transporter [Medicago truncatula]	5E-56
Contig220	363	337	2	2	0	ref|XP_389399.1|	hypothetical protein FG09223.1 [Gibberella zeae PH-1]	2E-34
Contig221	443	419	2	2	0	emb|CAN61355.1|	hypothetical protein [Vitis vinifera]	4E-41
Contig222	465	433	5	4	1	gb|EAZ22851.1|	hypothetical protein OsJ_006334 [Oryza sativa (japonica cultivar-group)]	3E-46
Contig223	448	422	2	2	0	gb|ABE93791.1|	NOT2/NOT3/NOT5 [Medicago truncatula]	3E-55
Contig224	302	302	5	5	0	sp|O65735|ALF_CICAR	Fructose-bisphosphate aldolase, cytoplasmic isozyme emb|CAA06308.1| cytosolic fructose-1,6 bisphosphate aldolase [Cicer arietinum]	1E-37

Contig225	213	192	2	2	0	gb|ABE87946.1|	CAP protein [Medicago truncatula]	9E-11
Contig226	554	507	10	8	2	gb|AAC49013.1|	polyubiquitin containing 7 ubiquitin monomers	7E-80
Contig227	346	307	2	2	0	emb|AJ404642.1|CAR 404642	Cicer arietinum partial ORF for NAD-dependent malic enzyme (malate oxidoreductase), exons 1-4	1E-172
Contig228	439	393	3	3	0	dbj|BAE71265.1|	putative serine/threonine protein kinase-like protein [Trifolium pratense] dbj|BAE71272.1| putative serine/threonine protein kinase-like protein [Trifolium pratense]	2E-45
Contig229	437	414	3	2	1	ref|XP_384347.1|	hypothetical protein FG04171.1 [Gibberella zeae PH-1]	4E-18
Contig230	531	469	22	7	15	gb|ABO78621.1|	Translation factor [Medicago truncatula]	3E-52
Contig231	349	326	5	5	0	dbj|BAE71236.1|	putative ADP,ATP carrier-like protein [Trifolium pratense]	9E-48
Contig232	447	421	2	2	0	gb|ABO93014.1|	putative sterol desaturase [Solanum tuberosum]	4E-30
Contig233	457	430	3	3	0	ref|NP_850433.2|	unknown protein [Arabidopsis thaliana]	9E-24
Contig234	459	435	2	2	0	gb|ABP02851.1|	Calcium-binding EF-hand [Medicago truncatula]	5E-47
Contig235	267	241	2	2	0	gb|AAN74635.1|	DEAD box RNA helicase [Pisum sativum] gb|AAR97917.1| DEAD box RNA helicase [Pisum sativum]	1E-35
Contig236	360	334	2	2	0	gb|ABD32320.1|	DNA-directed RNA polymerase, subunit C11/M/9 [Medicago truncatula]	1E-20
Contig237	469	442	6	6	0	ref|NW_001594396.1|	Aspergillus niger CBS 513.88 contig An17c0060, complete genome emb|AM270388.1| Aspergillus niger contig An17c0060, complete genome	2E-15
Contig238	407	351	6	5	1	gb|AC166543.2|	Nectria haematococca clone JGIAYWI-5I13, complete sequence	0.000007
Contig239	459	433	2	2	0	gb|ABE83505.2|	TPP-binding enzymes [Medicago truncatula]	6E-68
Contig240	297	271	2	2	0	gb|AAM65588.1|	putative auxin-induced protein, IAA12 [Arabidopsis thaliana]	4E-18
Contig241	629	605	3	1	2	emb|CAA54168.1|	HMG 1 protein [Pisum sativum]	3E-16
Contig242	456	430	4	2	2	emb|CAN65596.1|	hypothetical protein [Vitis vinifera]	5E-44
Contig243	458	434	5	5	0	emb|CAH05011.1|	alpha-dioxygenase [Pisum sativum]	7E-70
Contig244	439	399	3	3	0	gb|ABE84531.1|	Polyadenylate binding protein, human types 1, 2, 3, 4 [Medicago truncatula]	4E-50
Contig245	460	436	2	2	0	gb|ABO79784.1|	hypothetical protein MtrDRAFT_AC137825g21v2 [Medicago truncatula]	4E-12
Contig246	388	353	2	2	0	ref|NP_563993.1|	unknown protein [Arabidopsis thaliana] gb|AAL59995.1| unknown protein [Arabidopsis thaliana] gb|AAM61212.1| unknown [Arabidopsis thaliana] gb|AAM67462.1| unknown protein [Arabidopsis thaliana] dbj|BAE99103.1| hypothetical protein [Arabidopsis thaliana]	2E-28
Contig247	427	380	2	2	0	gb|ABD32869.1|	Zinc finger, RING-type; RINGv [Medicago truncatula] gb|ABE79868.2| Zinc finger, RING-type [Medicago truncatula]	4E-11

Contig248	209	191	2	2	0	emb|CT029313.1|	Poplar cDNA sequences	0.003
Contig249	457	431	4	4	0	gb|AAZ20293.1|	isomerase-like protein [Arachis hypogaea]	7E-69
Contig250	550	505	7	7	0	gb|ABO84301.1|	Alpha-isopropylmalate/homocitrate synthase [Medicago truncatula]	1E-76
Contig251	148	122	2	2	0	gb|AF537102.1|	Plasmodiophora brassicae 16S ribosomal RNA gene, partial sequence; mitochondrial gene for mitochondrial product	0.000009
Contig252	447	421	2	2	0	gb|ABE88265.1|	Generic methyltransferase [Medicago truncatula] gb|ABN08826.1| Generic methyltransferase [Medicago truncatula]	7E-70
Contig253	418	392	9	3	6	gb|BT013115.1|	Lycopersicon esculentum clone 114402R, mRNA sequence	4E-23
Contig254	447	423	3	3	0	ref|NP_567919.1|	peroxidase, putative [Arabidopsis thaliana]	1E-39
Contig255	459	433	2	2	0	sp|Q43070|GALE1_PEA	UDP-glucose 4-epimerase (Galactowaldenase) (UDP-galactose 4-epimerase) gb|AAA86532.1| UDP-galactose-4-epimerase	7E-72
Contig256	343	272	2	2	0	emb|AM706411.1|	Eristalis tenax partial mRNA for hypothetical protein (ORF1), isolate 3	0.0004
Contig257	448	319	3	3	0	gb|BT009458.1|	Triticum aestivum clone wlsu2.pk0001.h3:fis, full insert mRNA sequence	9E-12
Contig258	344	297	4	3	1	ref|XP_001220685.1|	hypothetical protein CHGG_01464 [Chaetomium globosum CBS 148.51] gb|EAQ93229.1| hypothetical protein CHGG_01464 [Chaetomium globosum CBS 148.51]	3E-18
Contig259	452	428	2	2	0	gb|ABA40435.1|	unknown [Solanum tuberosum]	8E-44
Contig260	928	910	16	3	13	emb|CAA06245.1|	elongation factor 1-alpha (EF1-a) [Cicer arietinum]	1E-125
Contig261	459	433	3	3	0	emb|CAC84710.1|	aux/IAA protein [Populus tremula x Populus tremuloides]	1E-24
Contig262	323	297	2	2	0	emb|AM706411.1|	Eristalis tenax partial mRNA for hypothetical protein (ORF1), isolate 3	0.00002
Contig263	464	438	4	3	1	gb|ABE82022.1|	Adenylate kinase [Medicago truncatula]	7E-72
Contig264	435	417	4	2	2	gb|AAC49370.1|	non-specific lipid transfer-like protein	3E-25
Contig265	451	427	4	4	0	gb|ABE82974.1|	Pyridoxal-5-phosphate-dependent enzyme, beta subunit [Medicago truncatula]	7E-70
Contig266	460	434	3	3	0	emb|CAD31716.1|	putative ripening related protein [Cicer arietinum]	4E-29
Contig267	666	644	4	1	3	gb|AAO72533.1|	pyruvate decarboxylase 1 [Lotus corniculatus]	1E-118
Contig268	373	326	2	2	0	ref|XP_386905.1|	hypothetical protein FG06729.1 [Gibberella zeae PH-1]	5E-45
Contig269	431	407	2	2	0	ref|XP_383344.1|	hypothetical protein FG03168.1 [Gibberella zeae PH-1]	7E-67
Contig270	349	302	3	3	0	gb|ABE85490.2|	Response regulator receiver; CCT [Medicago truncatula]	4E-36
Contig271	592	557	11	11	0	emb|CAN73396.1|	hypothetical protein [Vitis vinifera] emb|CAN65595.1| hypothetical protein [Vitis vinifera]	5E-18
Contig272	445	421	14	3	11	gb|ABA46758.1|	unknown [Solanum tuberosum] gb|ABB02647.1| unknown [Solanum tuberosum]	2E-61

Contig273	642	593	58	55	3	emb|CAN75753.1|	hypothetical protein [Vitis vinifera]	2E-69
Contig274	574	498	5	5	0	ref|XP_386196.1|	conserved hypothetical protein [Gibberella zeae PH-1]	6E-33
Contig275	478	428	11	5	6	emb|CAD31838.1|	putative quinone oxidoreductase [Cicer arietinum]	3E-67
Contig276	168	103	3	3	0	emb|AJ608703.3|	Fusarium oxysporum f. sp. lycopersici six1 gene, fot5 gene, six2 gene, shh1 gene and ORF2 (partial)	3E-24
Contig277	168	147	2	2	0	gb|AC145222.18|	Medicago truncatula clone mth2-29a15, complete sequence	3E-12
Contig278	444	420	2	1	1	gb|ABE77501.1|	Glutathione S-transferase, C-terminal-like; Thioredoxin fold [Medicago truncatula]	5E-60
Contig279	350	324	6	4	2	dbj|AK247755.1|	Solanum lycopersicum cDNA, clone: LEFL2005N13, HTC in fruit	8E-24
Contig280	329	288	5	1	4	gb|ABO78021.1|	Curculin-like (mannose-binding) lectin [Medicago truncatula]	1E-41
Contig281	408	384	2	2	0	gb|ABO81258.1|	Targeting for Xklp2 [Medicago truncatula]	2E-25
Contig282	461	434	3	1	2	emb|CAB87834.1|	putative kinetochore protein [Vicia faba var. minor]	8E-57
Contig283	276	276	2	1	1	emb|CAN62725.1|	hypothetical protein [Vitis vinifera]	2E-18
Contig284	452	428	3	1	2	emb|CAN65016.1|	hypothetical protein [Vitis vinifera]	1E-37
Contig285	454	428	3	1	2	gb|ABE81204.1|	Ribosomal protein S13 [Medicago truncatula] gb|ABE81211.1| Ribosomal protein S13 [Medicago truncatula]	2E-58
Contig286	457	431	7	6	1	sp|P12886|ADH1_PEA	Alcohol dehydrogenase 1 emb|CAA29609.1| alcohol dehydrogenase [Pisum sativum]	2E-61
Contig287	456	430	4	1	3	emb|CAA98163.1|	RAB1X [Lotus japonicus]	3E-44
Contig288	453	427	3	1	2	ref|XP_380574.1|	hypothetical protein FG00398.1 [Gibberella zeae PH-1] sp|Q4IQW0|NOP12_GIBZE Nucleolar protein 12	1E-17
Contig289	188	162	2	1	1	gb|ABE94198.1|	NAD-dependent epimerase/dehydratase [Medicago truncatula]	6E-20
Contig290	277	256	2	2	0	dbj|BAA02118.1|	GTP-binding protein [Pisum sativum] emb|CAA82707.1| guanine nucleotide regulatory protein [Vicia faba] prf||2001457K GTP-binding protein prf||2115367A small GTP-binding protein	1E-40
Contig291	428	404	2	2	0	gb|ABE77464.1|	hypothetical protein MtrDRAFT_AC146865g22v1 [Medicago truncatula]	2E-14
Contig292	423	397	5	1	4	emb|CAG28693.1|	hypothetical protein [Gibberella fujikuroi]	6E-36
Contig293	471	471	4	1	3	gb|AAK84887.1|AF402606_1	homeodomain leucine zipper protein HDZ3 [Phaseolus vulgaris]	2E-56
Contig294	303	279	11	11	0	emb|AM697674.1|	Platynereis dumerilii mRNA for hypothetical protein (ORF1), isolate 2	0.001
Contig295	374	348	2	1	1	gb|EAY87990.1|	hypothetical protein OsI_009223 [Oryza sativa (indica cultivar-group)]	1E-11
Contig296	427	372	8	1	7	gb|AAD55298.1|AC008263_29	F25A4.24 [Arabidopsis thaliana]	2E-52
Contig297	406	380	5	2	3	emb|CAN67631.1|	hypothetical protein [Vitis vinifera]	2E-56

Contig298	426	354	5	5	0	ref|XP_380857.1|	hypothetical protein FG00681.1 [Gibberella zeae PH-1]	6E-12
Contig299	576	531	12	1	11	gb|AAD37427.1|AF149277_1	peroxidase 1 precursor [Phaseolus vulgaris]	5E-43
Contig300	391	367	2	1	1	gb|EF416175.1|	Sesbania rostrata cDNA-AFLP fragment 043BT43M21-641.8 genomic sequence	6E-25
Contig301	424	398	2	1	1	gb|ABO79664.1|	hypothetical protein MtrDRAFT_AC137701g32v2 [Medicago truncatula]	8E-66
Contig302	465	441	3	3	0	ref|NP_201198.1|	leucine-rich repeat transmembrane protein kinase, putative [Arabidopsis thaliana] dbj|BAA96896.1| receptor-like protein kinase [Arabidopsis thaliana]	6E-30
Contig303	345	321	2	1	1	ref|XP_384358.1|	hypothetical protein FG04182.1 [Gibberella zeae PH-1]	2E-51
Contig304	453	427	5	4	1	gb|AAC50039.1|	polynucleotide phosphorylase [Pisum sativum]	2E-52
Contig305	455	429	3	2	1	gb|ABO82182.1|	Zinc finger, Sec23/Sec24-type; Sec23/Sec24 trunk region; Sec23/Sec24 helical region; Gelsolin region; Sec23/Sec24 beta-sandwich [Medicago truncatula]	3E-40
Contig306	367	293	2	2	0	gb|AF075691.1|	Crassostrea gigas BAT1 homolog mRNA, complete cds	8E-18
Contig307	454	430	3	2	1	emb|CR932967.2|	Medicago truncatula chromosome 5 clone mth2-49e21, COMPLETE SEQUENCE	4E-60
Contig308	461	435	3	2	1	sp|P21727|TPT_PEA	Triose phosphate/phosphate translocator, chloroplast precursor (cTPT) (p36) (E30) emb|CAA38451.1| chloroplast import receptor p36 [Pisum sativum] emb|CAA48210.1| phosphate translocator [Pisum sativum] prf||1805409A phosphate translocator	1E-75
Contig309	564	538	11	2	9	gb|BT009458.1|	Triticum aestivum clone wlsu2.pk0001.h3:fis, full insert mRNA sequence	0.0002
Contig310	448	424	3	2	1	gb|AAK54302.1|AC034258_20	putative helicase [Oryza sativa (japonica cultivar-group)] gb|AAP54108.1| AT hook motif-containing protein, putative [Oryza sativa (japonica cultivar-group)]	4E-16
Contig311	374	349	4	3	1	emb|CAN77754.1|	hypothetical protein [Vitis vinifera] emb|CAN81281.1| hypothetical protein [Vitis vinifera]	1E-64
Contig312	261	237	3	2	1	gb|AAM61409.1|	unknown [Arabidopsis thaliana]	4E-23
Contig313	313	289	5	3	2	emb|CAB56741.1|	cytochrome P450 monooxygenase [Cicer arietinum]	2E-18
Contig314	322	275	3	2	1	emb|CU302335.1|	Medicago truncatula chromosome 5 clone mth2-155g22, COMPLETE SEQUENCE	1E-19
Contig315	592	545	9	2	7	emb|CAB50768.1|	cytochrome P450 [Cicer arietinum]	2E-78
Contig316	346	307	4	3	1	ref|NP_196487.1|	KIWI; DNA binding / transcription coactivator [Arabidopsis thaliana] sp|O65154|KIWI_ARATH RNA polymerase II transcriptional coactivator KIWI gb|AAC08574.1| putative transcriptional co-activator [Arabidopsis thaliana] emb|CAC05451.1| putative transcriptional co-activator (KIWI) [Arabidopsis thaliana] gb|AAO44011.1| At5g09250 [Arabidopsis thaliana] dbj|BAF00036.1| putative transcriptional co-activator [Arabidopsis thaliana]	4E-28
Contig317	449	423	3	1	2	gb|ABC59084.1|	cytochrome P450 monooxygenase CYP83G1 [Medicago truncatula]	1E-40

Contig318	745	626	204	127	77	gb|AY232720.1|	Fusarium oxysporum f. sp. vasinfectum strain X515-II Foxy transposable element, partial sequence	0
Contig319	244	219	2	2	0	emb|CAG14983.1|	putative universal stress protein [Cicer arietinum]	3E-23
Contig320	153	130	2	2	0	ref|XP_001242204.1|	hypothetical protein CIMG_06100 [Coccidioides immitis RS] gb|EAS30621.1| hypothetical protein CIMG_06100 [Coccidioides immitis RS]	2E-11
Contig321	352	308	2	2	0	gb|ABE90931.1|	Sm-like protein [imported] - Arabidopsis thaliana [Medicago truncatula]	4E-43
Contig322	236	192	3	3	0	ref|XP_382064.1|	hypothetical protein FG01888.1 [Gibberella zeae PH-1]	3E-18
Contig323	654	628	7	3	4	gb|AAZ29733.1|	phenylalanine ammonia lyase [Trifolium pratense]	1E-111
Contig324	280	256	2	2	0	emb|CAN78902.1|	hypothetical protein [Vitis vinifera]	1E-36
Contig325	408	384	2	2	0	gb|ABE77684.1|	PpiC-type peptidyl-prolyl cis-trans isomerase; Rhodanese-like [Medicago truncatula] gb|ABE82359.1| PpiC-type peptidyl-prolyl cis-trans isomerase; Rhodanese-like [Medicago truncatula]	4E-41
Contig326	401	369	2	2	0	gb|EAY79580.1|	hypothetical protein OsI_033539 [Oryza sativa (indica cultivar-group)]	7E-53
Contig327	323	281	5	5	0	gb|ABN08532.1|	Prephenate dehydratase with ACT region [Medicago truncatula]	2E-44
Contig328	408	375	3	3	0	emb|CAA05979.1|	adenine nucleotide translocator [Lupinus albus]	4E-59
Contig329	151	130	2	2	0	gb|AC140546.12|	Medicago truncatula clone mth2-35l19, complete sequence	3E-15
Contig330	367	320	3	3	0	emb|CAN69269.1|	hypothetical protein [Vitis vinifera]	8E-52
Contig331	312	288	2	2	0	gb|AAM91533.1|	membrane related protein CP5, putative [Arabidopsis thaliana] gb|AAN15331.1| membrane related protein CP5, putative [Arabidopsis thaliana]	6E-29
Contig332	309	248	4	4	0	sp|Q53IP3|MBF1_GIBFU	Multiprotein-bridging factor 1 emb|CAG28684.1| multiprotein bridging factor [Gibberella fujikuroi]	2E-11
Contig333	400	376	3	3	0	gb|DQ068118.1|	Brassica napus isolate mutant Cr3529 clone Bncr10 unknown mRNA	0.007
Contig334	335	309	2	2	0	emb|CAE55867.1|	Fot5 transposase [Fusarium oxysporum f. sp. lycopersici]	2E-41
Contig335	515	473	5	4	1	ref|XP_386920.1|	hypothetical protein FG06744.1 [Gibberella zeae PH-1]	1E-59
Contig336	558	511	4	4	0	ref|NP_001048662.1|	Os03g0102400 [Oryza sativa (japonica cultivar-group)] dbj|BAF10576.1| Os03g0102400 [Oryza sativa (japonica cultivar-group)]	2E-29
Contig337	331	292	3	3	0	ref|XP_387704.1|	KPYK_TRIRE Pyruvate kinase [Gibberella zeae PH-1]	2E-36
Contig338	460	442	4	4	0	gb|EAZ25517.1|	hypothetical protein OsJ_009000 [Oryza sativa (japonica cultivar-group)]	3E-18
Contig339	288	237	2	2	0	gb|ABD33414.1|	Pectinesterase; Pectinesterase inhibitor [Medicago truncatula]	5E-41

Contig340	282	282	2	2	0	ref|XP_721448.1|	hypothetical protein CaO19_2949 [Candida albicans SC5314] ref|XP_721176.1| hypothetical protein CaO19_10466 [Candida albicans SC5314] gb|EAL02368.1| hypothetical protein CaO19.10466 [Candida albicans SC5314] gb|EAL02649.1| hypothetical protein CaO19.2949 [Candida albicans SC5314]	8E-37
Contig341	765	747	6	6	0	emb|CAN65177.1|	hypothetical protein [Vitis vinifera]	5E-28
Contig342	374	330	2	2	0	emb|CAN74679.1|	hypothetical protein [Vitis vinifera]	7E-27
Contig343	314	291	3	3	0	ref|XP_390948.1|	hypothetical protein FG10772.1 [Gibberella zeae PH-1]	4E-18
Contig344	299	275	3	2	1	emb|CAA51821.1|	ubiquitin conjugating enzyme E2 [Solanum lycopersicum] gb|ABB02644.1| ubiquitin conjugating enzyme E2-like [Solanum tuberosum]	1E-49
Contig345	541	541	3	1	2	ref|NP_566991.2|	universal stress protein (USP) family protein [Arabidopsis thaliana] gb|AAG40033.1|AF324682_1 AT3g53990 [Arabidopsis thaliana] gb|AAG40390.1|AF325038_1 AT3g53990 [Arabidopsis thaliana] gb|AAG41484.1|AF326902_1 unknown protein [Arabidopsis thaliana] gb|AAK00403.1|AF339721_1 unknown protein [Arabidopsis thaliana] gb|AAK32867.1|AF361855_1 AT3g53990/F5K20_290 [Arabidopsis thaliana] emb|CAB88361.1| hypothetical protein [Arabidopsis thaliana] gb|AAK96518.1| AT3g53990/F5K20_290 [Arabidopsis thaliana] gb|AAL31227.1| AT3g53990/F5K20_290 [Arabidopsis thaliana] gb|AAL49942.1| AT3g53990/F5K20_290 [Arabidopsis thaliana] dbj|BAD94963.1| hypothetical protein [Arabidopsis thaliana]	4E-49
Contig346	334	334	2	2	0	gb|AAL85977.1|	putative uridine kinase [Arabidopsis thaliana]	2E-48
Contig347	379	350	2	2	0	gb|ABE77539.1|	hypothetical protein MtrDRAFT_AC146866g9v2 [Medicago truncatula]	3E-21
Contig348	752	734	7	7	0	ref|NP_180560.1|	NDA2 (ALTERNATIVE NAD(P)H DEHYDROGENASE 2); NADH dehydrogenase [Arabidopsis thaliana] gb|AAC31853.1| putative NADH dehydrogenase (ubiquinone oxidoreductase) [Arabidopsis thaliana]	3E-52
Contig349	448	372	16	14	2	gb|AY232723.1|	Fusarium oxysporum f. sp. vasinfectum strain Ag149-I Foxy transposable element, partial sequence	0
Contig350	637	616	19	11	8	sp|Q39458|MT1_CICAR	Metallothionein-like protein 1 (MT-1) emb|CAA65008.1| metallothionein [Cicer arietinum]	2E-23
Contig351	239	188	25	22	3	gb|AY232720.1|	Fusarium oxysporum f. sp. vasinfectum strain X515-II Foxy transposable element, partial sequence	1E-101
Contig352	431	367	6	5	1	gb|ABG73467.1|	6-phosphogluconolactonase [Oryza brachyantha]	1E-22
Contig353	374	327	3	3	0	emb|CAB43932.1|	putative serine/threonine-specific receptor protein kinase [Arabidopsis thaliana] emb|CAB79676.1| putative serine/threonine-specific receptor protein kinase [Arabidopsis thaliana]	2E-31
Contig354	313	287	2	2	0	gb|DQ251457.1|	Siniperca chuatsi transposase mRNA, partial cds	0.00002

Contig355	358	334	2	2	0	gb|ABE80340.1|	TonB box, N-terminal; Tetratricopeptide-like helical [Medicago truncatula]	4E-14
Contig356	471	445	9	2	7	gb|AAY56795.1|	vacuolar H+-ATPase subunit A [Vigna unguiculata]	2E-79
Contig357	300	276	3	3	0	dbj|BAE71282.1|	putative receptor-like GPI-anchored protein 2 [Trifolium pratense]	2E-22
Contig358	459	433	2	2	0	gb|ABE77790.2|	Patatin [Medicago truncatula]	2E-56
Contig359	466	434	11	10	1	ref|XP_382134.1|	hypothetical protein FG01958.1 [Gibberella zeae PH-1]	7E-38
Contig360	443	419	4	4	0	gb|ABE84268.2|	Zinc finger, CCHC-type; Putative 5-3 exonuclease [Medicago truncatula]	8E-68
Contig361	461	414	12	8	4	emb|AJ608703.3|	Fusarium oxysporum f. sp. lycopersici six1 gene, fot5 gene, six2 gene, shh1 gene and ORF2 (partial)	1E-97
Contig362	452	399	2	2	0	ref|NP_198099.1|	seryl-tRNA synthetase / serine--tRNA ligase [Arabidopsis thaliana] sp|Q39230|SYS_ARATH Seryl-tRNA synthetase (Seryl-tRNA(Ser/Sec) synthetase) (Serine--tRNA ligase) (SerRS) gb|AAK28648.1|AF360352_1 putative seryl-tRNA synthetase [Arabidopsis thaliana] emb|CAA94388.1| seryl-tRNA Synthetase [Arabidopsis thaliana] gb|AAK93731.1| putative seryl-tRNA synthetase [Arabidopsis thaliana]	2E-15
Contig363	501	501	17	15	2	gb|AAZ98791.1|	cystatin [Medicago sativa]	4E-43
Contig364	484	460	5	5	0	ref|XP_387328.1|	hypothetical protein FG07152.1 [Gibberella zeae PH-1]	6E-29
Contig365	524	524	5	1	4	gb|ABC94943.1|	squalene epoxidase [Medicago sativa]	7E-54
Contig366	300	277	2	2	0	emb|CAN62209.1|	hypothetical protein [Vitis vinifera]	6E-26
Contig367	442	386	2	2	0	gb|ABO80441.1|	EPS15 homology (EH) [Medicago truncatula]	7E-24
Contig368	338	291	11	10	1	gb|AC145156.61|	Medicago truncatula clone mth2-7h6, complete sequence	6E-74
Contig369	111	111	2	2	0	gb|AC174370.14|	Medicago truncatula chromosome 8 clone mth2-50f20, complete sequence	8E-18
Contig370	344	305	4	4	0	ref|NP_001054396.1|	Os05g0103600 [Oryza sativa (japonica cultivar-group)] gb|AAS88829.1| putative ankyrin protein [Oryza sativa (japonica cultivar-group)] dbj|BAF16310.1| Os05g0103600 [Oryza sativa (japonica cultivar-group)]	4E-24
Contig371	420	420	2	2	0	ref|XP_001273877.1|	conserved hypothetical protein [Aspergillus clavatus NRRL 1] gb|EAW12451.1| conserved hypothetical protein [Aspergillus clavatus NRRL 1]	7E-22
Contig372	452	426	3	3	0	sp|P04353|CALM_SPIOL	Calmodulin (CaM)	2E-43
Contig373	454	428	6	6	0	ref|NP_189052.2|	unknown protein [Arabidopsis thaliana] gb|AAK44146.1|AF370331_1 unknown protein [Arabidopsis thaliana] gb|AAN13152.1| unknown protein [Arabidopsis thaliana]	3E-12

Contig374	397	376	2	2	0	ref|NP_200914.2|	HDA05 (HISTONE DEACETYLASE5); histone deacetylase [Arabidopsis thaliana] sp|Q8RX28|HDA5_ARATH Histone deacetylase 5 gb|AAM13986.1| putative histone deacetylase [Arabidopsis thaliana] gb|AAM53342.1| histone deacetylase-like protein [Arabidopsis thaliana] gb|AAN15392.1| histone deacetylase-like protein [Arabidopsis thaliana]	2E-24
Contig375	379	355	2	1	1	gb|AY972077.1|	Synthetic construct RLS (RLS) gene, complete cds	0.0004
Contig376	482	456	7	6	1	ref|XP_001227483.1|	hypothetical protein CHGG_09556 [Chaetomium globosum CBS 148.51] gb|EAQ85542.1| hypothetical protein CHGG_09556 [Chaetomium globosum CBS 148.51]	5E-39
Contig377	446	423	2	2	0	gb|AAW31901.1|	calcium-dependent/calmodulin-independent protein kinase isoform 3 [Cicer arietinum]	4E-71
Contig378	421	352	4	4	0	emb|CAA66478.1|	transcription factor [Vicia faba var. minor]	2E-44
Contig379	461	437	2	2	0	sp|P32110|GSTX6_SOYBN	Probable glutathione S-transferase (Heat shock protein 26A) (G2-4) gb|AAA33973.1| Gmhsp26-A	5E-48
Contig380	248	222	2	2	0	ref|NP_567434.1|	unknown protein [Arabidopsis thaliana] sp|Q8VXX9|BETL1_ARATH Bet1-like protein At4g14600 gb|AAL67066.1| unknown protein [Arabidopsis thaliana] gb|AAM14339.1| unknown protein [Arabidopsis thaliana] gb|AAM63165.1| unknown [Arabidopsis thaliana] dbj|BAC43249.1| unknown protein [Arabidopsis thaliana]	9E-22
Contig381	547	513	5	2	3	emb|CAA08855.1|	copper amine oxidase [Cicer arietinum]	4E-97
Contig382	296	282	2	2	0	sp|Q9AT63|PDX1_GINBI	Probable pyridoxal biosynthesis protein PDX1 (Sor-like protein) gb|AAK18310.1|AF344827_1 Sor-like protein [Ginkgo biloba]	5E-15
Contig383	469	391	7	7	0	emb|CAA71881.1|	Tyrosyl-tRNA synthetase [Nicotiana tabacum]	2E-45
Contig384	639	591	5	5	0	emb|AM497810.1|	Nidula niveotomentosa partial mRNA for glucose-6-phosphate-1-dehydrogenase (g6pd gene)	0.00005
Contig385	309	240	11	11	0	gb|BT009458.1|	Triticum aestivum clone wlsu2.pk0001.h3:fis, full insert mRNA sequence	2E-17
Contig386	407	390	2	2	0	gb|AC140850.20|	Medicago truncatula clone mth2-11i23, complete sequence	5E-47
Contig387	452	428	2	2	0	gb|ABO84100.1|	Something about silencing protein 10 , related [Medicago truncatula]	9E-38
Contig388	256	230	2	2	0	gb|ABE89195.1|	hypothetical protein MtrDRAFT_AC146775g29v2 [Medicago truncatula]	1E-35
Contig389	279	255	2	2	0	gb|AF493232.1|	Lycopersicon pimpinellifolium cysteine protease (Rcr3) gene, complete cds	0.018
Contig390	372	346	3	1	2	gb|ABD32881.1|	Nascent polypeptide-associated complex NAC; UBA-like [Medicago truncatula]	1E-33
Contig391	461	435	4	4	0	gb|ABE94111.1|	DNA repair protein RadA; Peptidase M41, FtsH [Medicago truncatula]	2E-50
Contig392	500	477	4	4	0	emb|CAA83548.1|	PsHSC71.0 [Pisum sativum]	1E-42
Contig393	431	407	14	14	0	gb|ABC94943.1|	squalene epoxidase [Medicago sativa]	8E-60

Contig394	157	133	2	2	0	emb|AJ749801.1|	Photobacterium damselae subsp. piscicida partial trpA gene for putative transposase, clone pRDA20	0.00004
Contig395	466	392	9	9	0	gb|ABE85988.1|	Zinc finger, RING-type; Zinc finger, RanBP2-type; Zinc finger, C6HC-type [Medicago truncatula]	2E-43
Contig396	256	230	3	3	0	sp|P15001|PHYA_PEA	Phytochrome A gb|AAA33682.1| phytochrome [Pisum sativum] emb|CAA32242.1| phytochrome apoprotein [Pisum sativum] gb|AAT97643.1| phytochrome A apoprotein [Pisum sativum] prf||1604466A phytochrome	4E-32
Contig397	311	285	2	2	0	ref|NP_199928.1|	unknown protein [Arabidopsis thaliana] dbj|BAA97377.1| unnamed protein product [Arabidopsis thaliana] gb|AAK76512.1| unknown protein [Arabidopsis thaliana] gb|AAM14313.1| unknown protein [Arabidopsis thaliana]	7E-32
Contig398	449	419	3	1	2	gb|ABE88379.1|	Nascent polypeptide-associated complex NAC [Medicago truncatula]	1E-61
Contig399	188	145	2	2	0	gb|ABE82627.1|	Argonaute and Dicer protein, PAZ; Stem cell self-renewal protein Piwi [Medicago truncatula] gb|ABE93839.1| Argonaute and Dicer protein, PAZ; Stem cell self-renewal protein Piwi [Medicago truncatula]	8E-12
Contig400	188	167	2	2	0	gb|EF672342.1|	Hypocrea sp. Z28 18S ribosomal RNA gene, partial sequence	8E-81
Contig401	178	133	2	2	0	gb|AF426840.1|	Trifolium repens early nodulin enod40-3 mRNA, complete cds	6E-20
Contig402	551	509	11	10	1	emb|AM706411.1|	Eristalis tenax partial mRNA for hypothetical protein (ORF1), isolate 3	0.0002
Contig403	424	398	2	2	0	gb|ABD33005.1|	Membrane bound O-acyl transferase, MBOAT [Medicago truncatula]	3E-58
Contig404	346	320	2	2	0	gb|AC182813.8|	Medicago truncatula clone mth2-69k1, complete sequence	2E-18
Contig405	326	299	2	2	0	gb|AAK68074.1|AF384970_1	somatic embryogenesis receptor-like kinase 3 [Arabidopsis thaliana]	4E-45
Contig406	461	436	2	2	0	ref|NP_200932.2|	protein binding [Arabidopsis thaliana] dbj|BAB08479.1| leucine-rich repeat disease resistance protein-like [Arabidopsis thaliana] gb|AAM91553.1| Cf-5 disease resistance protein-like [Arabidopsis thaliana] gb|AAN15323.1| Cf-5 disease resistance protein-like [Arabidopsis thaliana]	1E-59
Contig407	545	503	15	1	14	gb|AC140914.20|	Medicago truncatula clone mth2-18h17, complete sequence	1E-45
Contig408	311	290	6	3	3	ref|XM_381275.1|	Gibberella zeae PH-1 chromosome 1 RAN_BRUMA GTP-binding nuclear protein RAN/TC4 (FG01099.1) partial mRNA	7E-30
Contig409	137	114	2	1	1	ref|XP_389229.1|	hypothetical protein FG09053.1 [Gibberella zeae PH-1]	9E-14
Contig410	178	155	2	2	0	gb|EAZ07839.1|	hypothetical protein OsI_029071 [Oryza sativa (indica cultivar-group)]	7E-21
Contig411	434	413	5	3	2	emb|CAA10189.1|	class I chitinase [Cicer arietinum]	2E-57
Contig412	381	381	2	2	0	emb|AJ577394.1|CAR577394	Cicer arietinum 18S rRNA gene, 5.8S rRNA gene, IGS, ITS1 and ITS2, clone CanrDNA	0
Contig413	220	220	2	2	0	gb|AAZ94896.1|	actin-like protein [Catharanthus roseus]	3E-34
Contig414	200	146	5	1	4	emb|AM706411.1|	Eristalis tenax partial mRNA for hypothetical protein (ORF1), isolate 3	0.00005

Contig415	462	409	6	5	1	emb|CAN80597.1|	hypothetical protein [Vitis vinifera]	4E-66
Contig416	423	380	2	0	2	dbj|BAB40710.1|	BY-2 kinesin-like protein 10 [Nicotiana tabacum]	3E-33
Contig417	525	503	5	0	5	gb|ABE79354.1|	Embryo-specific 3 [Medicago truncatula]	2E-13
Contig418	453	429	4	0	4	emb|AJ749794.1|	Photobacterium damselae subsp. piscicida trpA gene for putative transposase and partial ORF1 DNA for hypothetical protein, clone pRDA13	0.0001
Contig419	425	407	3	0	3	ref|NP_850995.1|	binding [Arabidopsis thaliana] gb|AAG51417.1|AC009465_17 unknown protein; 78656-75813 [Arabidopsis thaliana] gb|AAM65399.1| contains similarity to O-linked GlcNAc transferases [Arabidopsis thaliana] gb|AAN72000.1| expressed protein [Arabidopsis thaliana]	6E-61
Contig420	349	326	2	0	2	emb|CR940305.16|	M.truncatula DNA sequence from clone MTH2-28N4 on chromosome 3, complete sequence	4E-35
Contig421	558	519	7	0	7	gb|ABE85685.1|	Cyclin-like F-box [Medicago truncatula]	2E-54
Contig422	333	303	2	0	2	gb|AC126019.16|	Medicago truncatula clone mth2-22p22, complete sequence	1E-34
Contig423	430	409	2	0	2	emb|Y15372.1|MTY15372	Medicago truncatula mRNA for MtN4 gene, partial	1E-10
Contig424	280	256	3	0	3	gb|ABR25719.1|	ADP-ribosylation factor 1 [Oryza sativa (indica cultivar-group)]	7E-43
Contig425	596	572	12	2	10	sp|P42654|1433B_VICFA	14-3-3-like protein B (VFA-1433B) emb|CAA88416.1| 14-3-3 brain protein homolog [Vicia faba]	8E-77
Contig426	421	379	2	0	2	dbj|BAB16429.1|	NtEIG-A1 [Nicotiana tabacum]	4E-13
Contig427	255	229	2	0	2	emb|CAN79067.1|	hypothetical protein [Vitis vinifera]	2E-21
Contig428	308	268	3	0	3	dbj|BAF45465.1|	hypothetical protein [Nicotiana tabacum]	1E-30
Contig429	920	873	9	1	8	sp|O81928|TCMO_CICAR	Trans-cinnamate 4-monooxygenase (Cinnamic acid 4-hydroxylase) (CA4H) (C4H) (P450C4H) (Cytochrome P450 73) emb|CAA07519.2| trans-cinnamic 4-monooxygenase [Cicer arietinum]	1E-159
Contig430	335	309	3	0	3	dbj|BAB43909.1|	phosphoenolpyruvate carboxykinase [Flaveria pringlei]	3E-27
Contig431	539	515	8	1	7	sp|Q9FY14|TIP1_MEDTR	Probable aquaporin TIP-type (MtAQP1) emb|CAC01618.1| aquaporin [Medicago truncatula]	3E-64
Contig432	312	289	3	0	3	ref|XM_458371.1|	Debaryomyces hansenii CBS767 hypothetical protein (DEHA0C16984g) partial mRNA	0.021
Contig433	363	299	4	2	2	ref|XP_381155.1|	conserved hypothetical protein [Gibberella zeae PH-1]	2E-49
Contig434	445	421	2	0	2	gb|AAZ38969.1|	GAMYB-binding protein [Glycine max]	9E-45
Contig435	922	858	84	67	17	sp|P12886|ADH1_PEA	Alcohol dehydrogenase 1 emb|CAA29609.1| alcohol dehydrogenase [Pisum sativum]	3E-36
Contig436	399	399	2	1	1	gb|AAB86380.1|	aquaporin-like transmembrane channel protein [Medicago sativa]	2E-67
Contig437	305	273	4	0	4	gb|AAB29959.2|	pathogen- and wound-inducible antifungal protein CBP20 precursor [Nicotiana tabacum]	1E-27

Contig438	459	435	2	0	2	ref|NM_105819.3|	Arabidopsis thaliana Rieske (2Fe-2S) domain-containing protein (AT1G71500) mRNA, complete cds	2E-09
Contig439	367	339	2	0	2	gb|AC155885.2|	Medicago truncatula chromosome 7 clone mth2-10p23, complete sequence	1E-47
Contig440	427	380	3	0	3	gb|ABE84165.2|	5-methyltetrahydropteroyltriglutamate--homocysteine S-methyltransferase; Prismane-like [Medicago truncatula] gb|ABE81639.2| 5-methyltetrahydropteroyltriglutamate--homocysteine S-methyltransferase; Prismane-like [Medicago truncatula]	2E-60
Contig441	375	351	5	1	4	emb|CAC67501.1|	selenium binding protein [Medicago sativa]	2E-57
Contig442	234	210	2	0	2	emb|CAN66493.1|	hypothetical protein [Vitis vinifera]	2E-26
Contig443	440	358	9	0	9	emb|CAN65399.1|	hypothetical protein [Vitis vinifera]	7E-18
Contig444	164	120	3	0	3	emb|AM158278.1|	Phaseolus vulgaris partial mRNA for proline-rich protein (prp3 gene), variety the Prince	8E-16
Contig445	513	471	12	0	12	ref|XP_712836.1|	hypothetical protein CaO19.14167 [Candida albicans SC5314] ref|XP_712807.1| hypothetical protein CaO19.6878 [Candida albicans SC5314] gb|EAK93638.1| questionable orf [Candida albicans SC5314] gb|EAK93667.1| questionable orf [Candida albicans SC5314]	4E-12
Contig446	599	114	3	0	3	gb|AC174296.26|	Medicago truncatula clone mth2-72l21, complete sequence	0.0005
Contig447	140	371	2	0	2	gb|DQ251457.1|	Siniperca chuatsi transposase mRNA, partial cds	0.000002
Contig448	400	345	2	0	2	emb|AM706411.1|	Eristalis tenax partial mRNA for hypothetical protein (ORF1), isolate 3	0.0001
Contig449	566	519	7	0	7	ref|NP_683525.2|	unknown protein [Arabidopsis thaliana] sp|Q6ID70|Y3377_ARATH Protein At3g03773 gb|AAT41786.1| At3g03773 [Arabidopsis thaliana] gb|AAT68743.1| hypothetical protein At3g03773 [Arabidopsis thaliana] gb|AAT70471.1| At3g03773 [Arabidopsis thaliana] gb|AAX55169.1| hypothetical protein At3g03773 [Arabidopsis thaliana]	8E-51
Contig450	459	433	4	0	4	gb|AAM65998.1|	putative dTDP-glucose 4-6-dehydratase [Arabidopsis thaliana]	2E-78
Contig451	442	400	2	0	2	dbj|BAF45465.1|	hypothetical protein [Nicotiana tabacum]	1E-44
Contig452	461	437	5	0	5	gb|AAK84885.1|AF402604_1	homeodomain leucine zipper protein HDZ1 [Phaseolus vulgaris]	1E-27
Contig453	587	563	6	1	5	emb|CAC06095.1|	ferredoxin-nitrite reductase [Lotus japonicus]	9E-91
Contig454	456	432	4	0	4	emb|CAK54360.1|	putative desaturase-like protein [Trifolium repens]	4E-80
Contig455	427	375	8	0	8	dbj|D14411.1|VIRARG2	Vigna radiata arg2 mRNA	3E-11
Contig456	247	223	4	2	2	emb|AM478166.2|	Vitis vinifera contig VV78X136477.5, whole genome shotgun sequence	2E-11
Contig457	205	169	3	1	2	gb|AAP80667.1|AF479048_1	ribosomal Pr 117 [Triticum aestivum]	1E-21
Contig458	691	667	5	0	5	gb|ABP03503.1|	Pathogenesis-related transcriptional factor and ERF [Medicago truncatula]	8E-70

Contig459	448	396	2	0	2	gb|ABB69781.1|	beta-glucan-binding protein 1 [Medicago truncatula]	8E-50
Contig460	306	247	5	0	5	gb|AF075691.1|	Crassostrea gigas BAT1 homolog mRNA, complete cds	9E-14
Contig461	525	483	5	0	5	gb|ABK06441.1|	flag-tagged protein kinase domain of putative mitogen-activated protein kinase kinase kinase [synthetic construct]	4E-14
Contig462	439	418	2	0	2	gb|ABB89773.1|	At3g12550-like protein [Boechera stricta]	6E-41
Contig463	340	297	2	0	2	gb|ABO79380.1|	Protein of unknown function DUF676, hydrolase-like [Medicago truncatula] gb|ABE80808.2| Protein of unknown function DUF676, hydrolase-like [Medicago truncatula]	6E-26
Contig464	264	190	4	0	4	emb|CR962134.2|	Medicago truncatula chromosome 5 clone mte1-40n1, COMPLETE SEQUENCE	8E-11
Contig465	448	324	2	0	2	gb|EAZ07445.1|	hypothetical protein OsI_028677 [Oryza sativa	2E-17
Contig466	254	195	3	0	3	emb|CAN76898.1|	hypothetical protein [Vitis vinifera]	2E-24
Contig467	137	120	2	0	2	gb|AC121233.16|	Medicago truncatula clone mth2-14g13, complete sequence	2E-16
Contig468	259	217	2	0	2	gb|AC148528.15|	Medicago truncatula clone mth2-53h4, complete sequence	3E-41
Contig469	414	393	2	0	2	emb|AM450436.2|	Vitis vinifera contig VV78X174412.6, whole genome shotgun sequence	0.0000001
Contig470	298	277	2	0	2	pdb|2IX6|A	Chain A, Short Chain Specific Acyl-Coa Oxidase From Arabidopsis Thaliana, Acx4 pdb|2IX6|B Chain B, Short Chain Specific Acyl-Coa Oxidase From Arabidopsis Thaliana, Acx4 pdb|2IX6|C Chain C, Short Chain Specific Acyl-Coa Oxidase From Arabidopsis Thaliana, Acx4 pdb|2IX6|D Chain D, Short Chain Specific Acyl-Coa Oxidase From Arabidopsis Thaliana, Acx4 pdb|2IX6|E Chain E, Short Chain Specific Acyl-Coa Oxidase From Arabidopsis Thaliana, Acx4 pdb|2IX6|F Chain F, Short Chain Specific Acyl-Coa Oxidase From Arabidopsis Thaliana, Acx4	6E-33
Contig471	455	431	5	0	5	gb|AY972077.1|	Synthetic construct RLS (RLS) gene, complete cds	0.0001
Contig472	396	349	3	0	3	ref|NP_190919.1|	ABC transporter family protein [Arabidopsis thaliana] sp|Q9LFG8|WBC20_ARATH Probable white-brown complex homolog protein 20 emb|CAB67658.1| ABC transporter-like protein [Arabidopsis thaliana]	2E-11
Contig473	355	312	3	0	3	gb|EAZ04460.1|	hypothetical protein OsI_025692 [Oryza sativa (indica cultivar-group)]	1E-12
Contig474	425	359	4	0	4	dbj|BAF00195.1|	hypothetical protein [Arabidopsis thaliana]	8E-15
Contig475	422	401	2	0	2	emb|CAA17544.1|	hypothetical protein [Arabidopsis thaliana] emb|CAB79125.1| hypothetical protein [Arabidopsis thaliana]	1E-26
Contig476	463	437	2	1	1	emb|CT028615.1|	Poplar cDNA sequences	0.000002
Contig477	440	416	2	0	2	ref|NP_196899.2|	unknown protein [Arabidopsis thaliana] ref|NP_001078580.1| unknown protein [Arabidopsis thaliana]	7E-14
Contig478	366	340	2	0	2	gb|AAC78102.1|	60S ribosomal protein L21 [Oryza sativa]	7E-27

Contig479	417	391	6	2	4	gb|AC169178.2|	Medicago truncatula chromosome 7 BAC clone mth2-2o21, complete sequence	1E-16
Contig480	352	328	2	0	2	gb|ABE90681.1|	Protein kinase; GroEL-like chaperone, ATPase [Medicago truncatula]	2E-55
Contig481	224	197	3	0	3	gb|ABD32628.1|	Granulin; Peptidase C1A, papain [Medicago truncatula]	1E-29
Contig482	1006	982	17	1	16	sp|Q9SML4|CHS1_CICAR	Chalcone synthase 1 (Naringenin-chalcone synthase 1) emb|CAA10190.1| chalcone synthase [Cicer arietinum]	1E-157
Contig483	452	431	5	3	2	sp|Q96423|TCMO_GLYEC	Trans-cinnamate 4-monooxygenase (Cinnamic acid 4-hydroxylase) (CA4H) (C4H) (P450C4H) (Cytochrome P450 73) dbj|BAA13414.1| cytochrome P450 (CYP73A14) [Glycyrrhiza echinata]	2E-66
Contig484	487	463	4	0	4	gb|ABN08635.1|	Mitochondrial import inner membrane translocase, subunit Tim17/22 [Medicago truncatula]	2E-60
Contig485	464	446	7	0	7	emb|CAN63150.1|	hypothetical protein [Vitis vinifera]	3E-58
Contig486	430	393	4	0	4	emb|CAA10067.1|	cytochrome P450 [Cicer arietinum]	1E-51
Contig487	202	202	3	1	2	dbj|BAB43813.1|	CaNAG2 [Candida albicans] dbj|BAB43820.1| CaNAG2 [Candida albicans]	2E-31
Contig488	532	471	17	13	4	sp|P13603|ADH1_TRIRP	Alcohol dehydrogenase 1 emb|CAA32934.1| unnamed protein product [Trifolium repens]	1E-80
Contig489	344	320	3	0	3	gb|AY972077.1|	Synthetic construct RLS (RLS) gene, complete cds	0.0004
Contig490	141	114	2	0	2	gb|AAT66941.1|	CesA2 [Acacia mangium]	1E-14
Contig491	432	409	5	0	5	ref|NP_563657.1|	CLPP5 (NUCLEAR ENCODED CLP PROTEASE 1); endopeptidase Clp [Arabidopsis thaliana] gb|AAG10637.1|AC022521_15 ATP-dependent Clp protease subunit ClpP [Arabidopsis thaliana] emb|CAB43488.1| ATP-dependent Clp protease subunit ClpP [Arabidopsis thaliana] dbj|BAA82065.1| nClpP1 [Arabidopsis thaliana] gb|AAM60971.1| ATP-dependent Clp protease proteolytic subunit ClpP5 [Arabidopsis thaliana] gb|ABD65590.1| At1g02560 [Arabidopsis thaliana]	9E-70
Contig492	297	255	3	0	3	emb|CAB71133.1|	hypothetical protein [Cicer arietinum]	4E-25
Contig493	229	166	2	0	2	gb|AC119419.9|	Medicago truncatula clone mth2-6b12, complete sequence	1E-24
Contig494	342	316	2	0	2	gb|ABE90057.1|	Cell division protein FtsZ [Medicago truncatula]	3E-48
Contig495	432	408	8	0	8	sp|O65735|ALF_CICAR	Fructose-bisphosphate aldolase, cytoplasmic isozyme emb|CAA06308.1| cytosolic fructose-1,6 bisphosphate aldolase [Cicer arietinum]	3E-24
Contig496	733	691	6	0	6	gb|ABE82951.1|	Translation protein SH3-like [Medicago truncatula]	1E-127
Contig497	280	256	2	0	2	gb|AAO46881.1|	60S ribosomal protein [Medicago sativa]	2E-39
Contig498	340	299	5	0	5	dbj|AB182104.1|	Silene latifolia SlSS mRNA for strictosidine synthase family protein, partial cds	0.00002
Contig499	460	436	2	0	2	gb|AAQ72787.1|	putative GTP-binding protein [Cucumis sativus]	2E-46

Contig500	448	422	3	0	3	dbj|AK226272.1|	Arabidopsis thaliana mRNA for ribosomal protein S30 homolog, complete cds, clone: RAFL05-08-E11	4E-29
Contig501	332	293	3	0	3	emb|CT029336.1|	Poplar cDNA sequences	0.022
Contig502	452	426	4	0	4	gb|AAX47170.1|	SHORT VEGETATIVE PHASE [Pisum sativum]	8E-52
Contig503	208	184	2	0	2	gb|ABO83561.1|	RabGAP/TBC [Medicago truncatula] gb|ABO83596.1| RabGAP/TBC [Medicago truncatula]	1E-27
Contig504	225	185	2	0	2	gb|DQ251457.1|	Siniperca chuatsi transposase mRNA, partial cds	0.000004
Contig505	454	454	14	12	2	ref|XP_720740.1|	hypothetical protein CaO19_11781 [Candida albicans SC5314] ref|XP_720612.1| hypothetical protein CaO19_4305 [Candida albicans SC5314] gb|EAL01777.1| hypothetical protein CaO19.4305 [Candida albicans SC5314] gb|EAL01911.1| hypothetical protein CaO19.11781 [Candida albicans SC5314]	8E-47
Contig506	407	370	3	1	2	gb|ABE79095.2|	Sec61beta [Medicago truncatula]	5E-27
Contig507	368	344	3	0	3	gb|EAZ42794.1|	hypothetical protein OsJ_026277 [Oryza sativa (japonica cultivar-group)]	6E-20
Contig508	217	193	2	0	2	gb|ABE88015.1|	hypothetical protein MtrDRAFT_AC146791g14v2 [Medicago truncatula]	6E-23
Contig509	615	591	8	0	8	gb|ABE93756.1|	Longin-like [Medicago truncatula]	4E-74
Contig510	437	419	7	0	7	emb|CAA06853.1|	26S protease regulatory subunit 6 [Cicer arietinum]	2E-71
Contig511	469	445	5	0	5	emb|CAN79836.1|	hypothetical protein [Vitis vinifera]	2E-42
Contig512	366	324	3	0	3	emb|AM484893.2|	Vitis vinifera contig VV78X135458.6, whole genome shotgun sequence	0.0001
Contig513	268	247	3	0	3	ref|NP_564656.1|	LEM3 (ligand-effect modulator 3) family protein / CDC50 family protein [Arabidopsis thaliana] gb|AAD25612.1|AC005287_14 Unknown protein [Arabidopsis thaliana] gb|AAL38602.1|AF446869_1 At1g54320/F20D21_50 [Arabidopsis thaliana] gb|AAK74030.1| At1g54320/F20D21_50 [Arabidopsis thaliana] gb|AAK96636.1| At1g54320/F20D21_50 [Arabidopsis thaliana]	1E-19
Contig514	318	234	4	0	4	emb|CT028662.1|	Poplar cDNA sequences	0.00009
Contig515	549	487	8	4	4	gb|AAD19957.1|	thiosulfate sulfurtransferase [Datisca glomerata]	2E-19
Contig516	428	410	2	0	2	ref|XP_001234258.1|	PREDICTED: similar to bacterial IS-element [Gallus gallus]	7E-46
Contig517	492	468	9	1	8	sp|O81361|RS8_PRUAR	40S ribosomal protein S8 gb|AAC24583.1| 40S ribosomal protein S8 [Prunus armeniaca]	3E-58
Contig518	454	405	2	0	2	gb|AAC16330.1|	SAR DNA-binding protein-1 [Pisum sativum]	3E-66
Contig519	140	114	2	0	2	gb|BT009458.1|	Triticum aestivum clone wlsu2.pk0001.h3:fis, full insert mRNA sequence	0.0001
Contig520	264	240	2	0	2	emb|AM697674.1|	Platynereis dumerilii mRNA for hypothetical protein (ORF1), isolate 2	0.001
Contig521	722	698	9	0	9	gb|ABO84376.1|	cAMP response element binding (CREB) protein [Medicago truncatula]	2E-22

Contig522	445	421	3	0	3	gb|AAM62626.1|	nodulin protein, putative [Arabidopsis thaliana]	1E-43
Contig523	455	429	5	0	5	ref|XM_383891.1|	Gibberella zeae PH-1 chromosome 2 hypothetical protein (FG03715.1) partial mRNA	6E-13
Contig524	422	373	3	0	3	gb|ABE93901.1|	Nonaspanin (TM9SF) [Medicago truncatula]	2E-43
Contig525	327	300	2	0	2	dbj|BAA25187.1|	ARG10 [Vigna radiata]	6E-15
Contig526	218	192	2	0	2	gb|ABE85161.1|	Nascent polypeptide-associated complex NAC; UBA-like [Medicago truncatula]	4E-12
Contig527	206	188	2	1	1	gb|ABQ95992.1|	14-3-3-like protein [Cicer arietinum] gb|ABQ95994.1| 14-3-3-like protein [Cicer arietinum]	3E-29
Contig528	460	436	3	0	3	gb|AC124218.18|	Medicago truncatula clone mth2-30b20, complete sequence	2E-46
Contig529	286	262	2	0	2	gb|EAZ25517.1|	hypothetical protein OsJ_009000 [Oryza sativa (japonica cultivar-group)]	1E-16
Contig530	408	384	3	0	3	emb|CAB71135.1|	putative imbibition protein [Cicer arietinum]	4E-69
Contig531	711	664	7	1	6	gb|ABB89021.1|	CXE carboxylesterase [Actinidia deliciosa]	4E-52
Contig532	492	468	22	0	22	sp|P08688|ALB2_PEA	Albumin-2 (PA2) gb|AAA02981.1| albumin 2 gb|AAA33641.1| major seed albumin prf||1314296A albumin	6E-26
Contig533	482	454	5	0	5	gb|ABO80199.1|	Ribosomal L23 protein; Ribosomal protein L23, N-terminal [Medicago truncatula]	4E-43
Contig534	462	436	9	0	9	dbj|BAE71244.1|	putative DNA binding protein [Trifolium pratense]	9E-75
Contig535	423	399	2	1	1	emb|AJ749797.1|	Photobacterium damselae subsp. piscicida trpB gene for putative transposase, clone pRDA16	0.0005
Contig536	321	297	2	0	2	emb|CAI56440.1|	S-adenosyl-L-homocysteine hydrolase [Cicer arietinum]	2E-48
Contig537	312	286	2	0	2	gb|ABN08656.1|	Ribosomal protein S10, eukaryotic and archaeal form [Medicago truncatula]	4E-35
Contig538	445	421	2	0	2	gb|AAF68120.1|AC010793_15	F20B17.14 [Arabidopsis thaliana] gb|AAG52249.1|AC011717_17 putative aspartyl protease; 105611-106921 [Arabidopsis thaliana]	3E-26
Contig539	465	439	5	0	5	gb|ABE89020.1|	Adenosine kinase [Medicago truncatula]	3E-76
Contig540	229	203	2	0	2	gb|ABE77438.1|	Ribosomal protein L24/L26 [Medicago truncatula]	7E-14
Contig541	421	400	4	1	3	gb|AC134242.17|	Medicago truncatula clone mth2-10p20, complete sequence	0.00003
Contig542	326	302	4	0	4	gb|AY972077.1|	Synthetic construct RLS (RLS) gene, complete cds	0.0004
Contig543	552	527	9	2	7	gb|AAP33475.1|	polygalacturonase-like protein [Fragaria x ananassa]	7E-87
Contig544	338	314	2	0	2	gb|AAL29212.1|AF354454_1	putative acyl-CoA synthetase [Capsicum annuum]	7E-26
Contig545	456	430	6	0	6	gb|ABN08040.1|	Acyl-coA-binding protein, ACBP; Serine/threonine protein phosphatase, BSU1 [Medicago truncatula]	6E-74
Contig546	452	428	6	0	6	emb|CAN66217.1|	hypothetical protein [Vitis vinifera]	2E-24

Contig547	234	216	3	0	3	gb|AC139745.35|	Medicago truncatula clone mth2-17d15, complete sequence	2E-23
Contig548	491	446	7	2	5	emb|CR936368.12|	M.truncatula DNA sequence from clone MTH2-4O11 on chromosome 3, complete sequence	5E-32
Contig549	141	120	2	0	2	gb|DQ072007.1|	Streptococcus mutans clone D7, genomic sequence	0.0005
Contig550	292	268	3	0	3	gb|AAM65672.1|	4-coumarate-CoA ligase-like protein [Arabidopsis thaliana]	7E-35
Contig551	255	231	2	0	2	gb|AY972077.1|	Synthetic construct RLS (RLS) gene, complete cds	0.004
Contig552	337	310	4	1	3	gb|EAZ16684.1|	hypothetical protein OsJ_030893 [Oryza sativa (japonica cultivar-group)]	2E-19
Contig553	224	203	2	0	2	gb|AAR29343.1|	allantoinase [Robinia pseudoacacia]	3E-30
Contig554	421	400	2	0	2	ref|XP_384449.1|	hypothetical protein FG04273.1 [Gibberella zeae PH-1]	6E-52
Contig555	685	645	5	1	4	gb|AAS46231.1|	methionine sulfoxide reductase A [Populus trichocarpa x Populus deltoides]	7E-86
Contig556	424	347	3	0	3	gb|ABA40437.1|	40S ribosomal protein S7-like protein [Solanum tuberosum] gb|ABA46775.1| unknown [Solanum tuberosum] gb|ABB17004.1| ribosomal protein S7-like protein [Solanum tuberosum] gb|ABB87101.1| 40S ribosomal protein S7-like protein-like [Solanum tuberosum]	1E-49
Contig557	376	337	4	0	4	gb|ABD28715.1|	Peptidase S24, S26A and S26B [Medicago truncatula]	3E-40
Contig558	692	645	7	0	7	gb|AAZ66745.1|	coronatine-insensitive 1 [Glycine max]	1E-104
Contig559	743	693	117	18	99	sp|P27047|DRR4_PEA	Disease resistance response protein DRRG49-C gb|AAA33663.1| disease resistance response protein (DRRG49-c)	1E-71
Contig560	451	425	3	0	3	emb|AM706411.1|	Eristalis tenax partial mRNA for hypothetical protein (ORF1), isolate 3	0.0001
Contig561	447	424	3	0	3	emb|CAN77401.1|	hypothetical protein [Vitis vinifera]	4E-12
Contig562	449	423	2	0	2	ref|NP_176514.1|	DEAD box RNA helicase, putative [Arabidopsis thaliana] sp|Q9C8S9|RH48_ARATH Probable DEAD-box ATP-dependent RNA helicase 48 gb|AAG52143.1|AC022355_4 putative RNA helicase; 42376-45543 [Arabidopsis thaliana]	3E-21
Contig563	434	410	3	0	3	emb|CAA63093.1|	alcohol dehydrogenase [Solanum tuberosum]	2E-33
Contig564	328	281	6	0	6	gb|AAY34909.1|	enolase [Prunus armeniaca]	6E-47
Contig565	340	316	3	0	3	gb|AC134822.19|	Medicago truncatula clone mth2-15j20, complete sequence	1E-22
Contig566	467	443	4	0	4	dbj|BAB86847.1|	elongation factor EF-2 [Pisum sativum]	2E-81
Contig567	464	436	2	0	2	gb|ABP87900.1|	ethylene receptor [Glycine max]	6E-46
Contig568	457	431	4	0	4	dbj|BAB10271.1|	ankyrin-like protein [Arabidopsis thaliana]	1E-67
Contig569	322	296	3	0	3	emb|CAA09589.1|	pepc2 [Vicia faba]	7E-35

Contig570	225	199	2	0	2	ref|NM_113389.1|	Arabidopsis thaliana CLE41 (CLAVATA3/ESR-RELATED 41); receptor binding (CLE41) mRNA, complete cds	0.0000002
Contig571	420	402	3	2	1	ref|XP_391802.1|	H2B_NEUCR Histone H2B [Gibberella zeae PH-1] sp|Q4HTT2|H2B_GIBZE Histone H2B	6E-33
Contig572	420	373	2	0	2	sp|Q9SQF4|SUI1_BRAOL	Protein translation factor SUI1 homolog (Translation initiation factor nps45) gb|AAF04624.1|AF098672_1 translation initiation factor nps45 [Brassica oleracea]	2E-32
Contig573	320	296	2	0	2	ref|NP_973636.1|	unknown protein [Arabidopsis thaliana]	4E-28
Contig574	582	532	10	3	7	sp|O24301|SUS2_PEA	Sucrose synthase 2 (Sucrose-UDP glucosyltransferase 2) emb|CAA04512.1| second sucrose synthase [Pisum sativum]	8E-92
Contig575	380	293	84	7	77	dbj|BAB33421.1|	putative senescence-associated protein [Pisum sativum]	4E-45
Contig576	661	661	14	3	11	emb|CAN63462.1|	hypothetical protein [Vitis vinifera]	4E-40
Contig577	659	638	3	2	1	gb|AAB19212.1|	polygalacturonase-inhibiting protein [Malus x domestica] gb|ABA26937.1| polygalacturonase-inhibiting protein [Malus x domestica]	1E-84
Contig578	431	411	2	0	2	emb|CAN69512.1|	hypothetical protein [Vitis vinifera]	6E-68
Contig579	326	302	3	0	3	gb|DQ068118.1|	Brassica napus isolate mutant Cr3529 clone Bncr10 unknown mRNA	0.006
Contig580	462	441	6	0	6	gb|ABC69764.1|	unknown [Vitis pseudoreticulata]	5E-24
Contig581	457	433	6	1	5	emb|CAA66108.1|	specific tissue protein 1 [Cicer arietinum]	7E-62
Contig582	406	386	3	0	3	sp|P47922|NDK1_PEA	Nucleoside diphosphate kinase 1 (Nucleoside diphosphate kinase I) (NDK I) (NDP kinase I) (NDPK I) emb|CAA50511.1| nucleoside-diphosphate kinase [Pisum sativum]	5E-55
Contig583	467	417	6	0	6	gb|AC137701.25|	Medicago truncatula clone mth2-35h1, complete sequence	6E-16
Contig584	288	240	4	0	4	emb|AM748403.1|	Vigna unguiculata partial mRNA for putative rubisco activase (Rca gene), clone 17	0.0003
Contig585	619	619	5	2	3	emb|CAN74145.1|	hypothetical protein [Vitis vinifera]	2E-83
Contig586	731	689	4	1	3	gb|ABH02865.1|	MYB transcription factor MYB176 [Glycine max]	5E-33
Contig587	714	688	8	0	8	dbj|BAA19156.1|	HMG-1 [Canavalia gladiata]	1E-45
Contig588	427	354	20	4	16	gb|AAG17879.1|AF293406_1	60S ribosomal protein L10A [Phaseolus coccineus]	1E-40
Contig589	457	431	7	0	7	gb|ABE82951.1|	Translation protein SH3-like [Medicago truncatula]	3E-66
Contig590	457	433	2	0	2	ref|NP_974254.1|	unknown protein [Arabidopsis thaliana] gb|AAG50831.1|AC074395_5 unknown protein, 5' partial [Arabidopsis thaliana]	2E-52
Contig591	420	394	2	0	2	gb|AAQ20041.1|	isoflavone 3'-hydroxylase [Medicago truncatula]	9E-56
Contig592	352	328	3	0	3	gb|AAG50672.1|AC079829_5	hypothetical protein [Arabidopsis thaliana]	2E-12
Contig593	590	510	4	0	4	gb|AC137703.42|	Medicago truncatula clone mth2-11d24, complete sequence	3E-15

Contig594	595	577	7	3	4	gb|AAZ32865.1|	thioredoxin h [Medicago sativa]	7E-52
Contig595	362	336	4	3	1	gb|ABE80304.1|	Extradiol ring-cleavage dioxygenase, class III enzyme, subunit B [Medicago truncatula]	2E-50
Contig596	664	638	5	0	5	dbj|BAB86539.1|	putative aspartate aminotransferase [Oryza sativa (japonica cultivar-group)]	2E-81
Contig597	482	433	6	2	4	ref|NP_563985.1|	GOS11 (GOLGI SNARE 11); SNARE binding [Arabidopsis thaliana] sp|Q9LMP7|GOS11_ARATH Golgi SNARE 11 protein (AtGOS11) (Golgi SNAP receptor complex member 1-1) gb|AAF82157.1|AC034256_21 Contains similarity to GOS28/P28 protein from Homo sapiens gb|AF047438. ESTs gb|F14225, gb|AA395297, gb|BE038320 come from this gene. [Arabidopsis thaliana] gb|AAG48789.1|AF332426_1 unknown protein [Arabidopsis thaliana] gb|AAK48904.1|AF357528_1 Golgi SNARE 11 protein [Arabidopsis thaliana] gb|AAK95249.1|AF410263_1 At1g15880/F7H2_20 [Arabidopsis thaliana] gb|AAM10347.1| At1g15880/F7H2_20 [Arabidopsis thaliana]	1E-49
Contig598	282	235	2	0	2	emb|CAB50768.1|	cytochrome P450 [Cicer arietinum]	1E-39
Contig599	288	261	2	0	2	gb|AAB33256.1|	Clostridium pasteurianum ferredoxin homolog [Solanum tuberosum]	7E-21
Contig600	489	429	5	3	2	gb|AAZ32910.1|	polygalacturonase-like protein [Medicago sativa]	3E-69
Contig601	418	394	2	0	2	gb|AAD25952.1|AF085717_1	putative callose synthase catalytic subunit [Gossypium hirsutum]	1E-40
Contig602	451	427	3	0	3	emb|CAN59927.1|	hypothetical protein [Vitis vinifera]	3E-53
Contig603	440	414	3	0	3	emb|CAA10287.2|	glucan-endo-1,3-beta-glucosidase [Cicer arietinum]	2E-29
Contig604	466	440	4	0	4	gb|AC166897.12|	Medicago truncatula clone mth2-64l14, complete sequence	1E-60
Contig605	567	513	9	8	1	gb|ABO82441.1|	Auxin responsive SAUR protein [Medicago truncatula]	2E-40
Contig606	287	269	5	1	4	gb|AAG17666.1|	S-adenosylmethionine synthetase [Brassica juncea]	4E-43
Contig607	184	161	2	0	2	ref|NM_123209.3|	Arabidopsis thaliana GRF3 (GENERAL REGULATORY FACTOR 3); protein phosphorylated amino acid binding (GRF3) mRNA, complete cds	2E-22
Contig608	453	429	5	0	5	gb|ABE92923.1|	C2; Peptidase, cysteine peptidase active site [Medicago truncatula]	8E-73
Contig609	591	565	11	1	10	emb|CAD56219.1|	ribosomal protein S3a [Cicer arietinum]	1E-102
Contig610	457	396	2	0	2	gb|AC123976.23|	Medicago truncatula clone mth2-30j23, complete sequence	5E-32
Contig611	337	311	2	0	2	gb|ABE80756.1|	DNA-binding WRKY [Medicago truncatula] gb|ABO80762.1| DNA-binding WRKY [Medicago truncatula]	7E-21
Contig612	264	238	2	0	2	gb|ABE86055.1|	Amino acid/polyamine transporter II [Medicago truncatula]	8E-34
Contig613	572	527	16	7	9	emb|CAA10067.1|	cytochrome P450 [Cicer arietinum]	1E-98

Contig614	448	409	2	0	2	gb|AC140104.22|	Medicago truncatula clone mth2-10g22, complete sequence	6E-13
Contig615	460	436	2	0	2	gb|ABO79609.1|	Ataxin-2, N-terminal; Like-Sm ribonucleoprotein-related, core [Medicago truncatula] gb|ABO81296.1| Ataxin-2, N-terminal; Like-Sm ribonucleoprotein-related, core [Medicago truncatula]	1E-13
Contig616	418	400	4	2	2	gb|AAZ29733.1|	phenylalanine ammonia lyase [Trifolium pratense]	9E-61
Contig617	462	436	3	0	3	emb|CAB16844.1|	serine C-palmitoyltransferase like protein [Arabidopsis thaliana] emb|CAB80314.1| serine C-palmitoyltransferase like protein [Arabidopsis thaliana]	6E-66
Contig618	455	432	4	0	4	gb|ABE79860.1|	Peptidase T1A, proteasome beta-subunit [Medicago truncatula]	1E-67
Contig619	463	437	2	0	2	emb|CAA04447.1|	DnaJ-like protein [Medicago sativa] gb|AAC19391.1| DnaJ-like protein MsJ1 [Medicago sativa]	6E-49
Contig620	264	240	2	0	2	gb|AY972077.1|	Synthetic construct RLS (RLS) gene, complete cds	0.00007
Contig621	359	335	2	0	2	gb|AY972077.1|	Synthetic construct RLS (RLS) gene, complete cds	0.0001
Contig622	464	438	3	0	3	emb|CAA10129.1|	hypothetical protein [Cicer arietinum]	2E-32
Contig623	464	438	3	0	3	gb|ABE77463.1|	conserved hypothetical protein [Medicago truncatula]	2E-49
Contig624	447	423	2	0	2	gb|ABF59516.1|	putative spindle disassembly related protein CDC48 [Nicotiana tabacum]	2E-34
Contig625	481	434	4	0	4	gb|ABH10138.1|	NADP-thioredoxin reductase A [Medicago truncatula]	1E-51
Contig626	460	434	3	0	3	sp|Q41009|TOC34_PEA	Translocase of chloroplast 34 (34 kDa chloroplast outer envelope protein) (GTP-binding protein OEP34) (GTP-binding protein IAP34) emb|CAA82196.1| chloroplast outer envelope protein 34 [Pisum sativum] gb|AAC25785.1| GTP-binding protein [Pisum sativum]	3E-71
Contig627	459	435	5	0	5	gb|AAM94349.1|	pyruvate kinase [Glycine max]	1E-74
Contig628	452	426	2	0	2	emb|CAC35070.1|	VIP3 protein [Zea mays]	3E-51
Contig629	454	430	2	1	1	emb|CAN63142.1|	hypothetical protein [Vitis vinifera]	3E-41
Contig630	323	281	2	0	2	ref|XP_386626.1|	hypothetical protein FG06450.1 [Gibberella zeae PH-1]	8E-47
Contig631	459	435	2	0	2	gb|ABA12218.1|	translation elongation factor 1A-2 [Gossypium hirsutum]	7E-77
Contig632	486	460	3	0	3	gb|ABP02563.1|	AMP-dependent synthetase and ligase [Medicago truncatula]	1E-76
Contig633	488	442	4	0	4	ref|NP_001047515.1|	Os02g0634800 [Oryza sativa (japonica cultivar-group)] dbj|BAD25096.1| putative ubiquitin-conjugating enzyme E2 [Oryza sativa (japonica cultivar-group)] dbj|BAD25314.1| putative ubiquitin-conjugating enzyme E2 [Oryza sativa (japonica cultivar-group)] dbj|BAF09429.1| Os02g0634800 [Oryza sativa (japonica cultivar-group)]	9E-75
Contig634	481	455	3	0	3	dbj|BAA25755.1|	vcCyP [Vicia faba]	2E-66

Contig635	151	127	2	0	2	emb|CAN68644.1|	hypothetical protein [Vitis vinifera]	3E-14
Contig636	525	467	15	12	3	emb|AJ250814.1|FOX250814	Fusarium oxysporum f. sp. lycopersici insertion sequence Foxy	0
Contig637	455	431	2	0	2	gb|AAD37428.1|AF149278_1	peroxidase 3 precursor [Phaseolus vulgaris]	6E-37
Contig638	191	167	2	0	2	emb|AJ749797.1|	Photobacterium damselae subsp. piscicida trpB gene for putative transposase, clone pRDA16	0.0002
Contig639	379	347	3	1	2	ref|NP_001060360.1|	Os07g0631100 [Oryza sativa (japonica cultivar-group)] sp|Q8LHP0|ELOF1_ORYSJ Transcription elongation factor 1 homolog dbj|BAC10134.1| unknown protein [Oryza sativa (japonica cultivar-group)] dbj|BAF22274.1| Os07g0631100 [Oryza sativa (japonica cultivar-group)] gb|EAY85182.1| hypothetical protein OsI_006415 [Oryza sativa (indica cultivar-group)] gb|EAZ40760.1| hypothetical protein OsJ_024243 [Oryza sativa (japonica cultivar-group)]	3E-33
Contig640	713	676	7	2	5	emb|CAB71135.1|	putative imbibition protein [Cicer arietinum]	1E-100
Contig641	447	421	2	0	2	ref|NP_191771.1|	tRNA synthetase class II (G, H, P and S) family protein [Arabidopsis thaliana] ref|NP_850736.1| tRNA synthetase class II (G, H, P and S) family protein [Arabidopsis thaliana] emb|CAB71872.1| multifunctional aminoacyl-tRNA ligase-like protein [Arabidopsis thaliana] gb|AAL24294.1| multifunctional aminoacyl-tRNA ligase-like protein [Arabidopsis thaliana] gb|AAM91120.1| multifunctional aminoacyl-tRNA ligase-like protein [Arabidopsis thaliana]	2E-59
Contig642	208	182	2	0	2	gb|AF537102.1|	Plasmodiophora brassicae 16S ribosomal RNA gene, partial sequence; mitochondrial gene for mitochondrial product	0.00001
Contig643	417	375	2	0	2	emb|CAG34224.1|	putative Bet v I family protein [Cicer arietinum]	8E-47
Contig644	193	172	2	0	2	gb|ABD33216.1|	Peptidase A1, pepsin [Medicago truncatula]	3E-21
Contig645	427	427	3	0	3	gb|ABE81462.1|	Protein kinase [Medicago truncatula]	1E-27
Contig646	442	416	4	2	2	emb|CAN80447.1|	hypothetical protein [Vitis vinifera]	1E-32
Contig647	419	378	4	2	2	gb|ABE87918.1|	Cupin region [Medicago truncatula]	8E-30
Contig648	416	398	2	0	2	gb|ABC01895.1|	transporter-like protein [Solanum tuberosum]	6E-62
Contig649	490	457	24	2	22	emb|AJ299396.1|CAR299396	Cicer arietinum partial mRNA for putative extensin (ORF), clone CanEXT-1	1E-81
Contig650	436	401	3	0	3	gb|ABO81948.2|	Zinc finger, GATA-type [Medicago truncatula]	6E-26
Contig651	458	434	2	1	1	gb|AAY44152.1|	ATP synthase beta subunit [Ecballium elaterium]	3E-66
Contig652	368	344	2	0	2	gb|ABE84302.1|	hypothetical protein MtrDRAFT_AC146552g12v2 [Medicago truncatula]	3E-12
Contig653	315	297	2	0	2	dbj|AB307742.1|	Antheraea yamamai ap mRNA for aminopeptidase N, partial cds	0.005
Contig654	454	454	5	0	5	emb|CAD31716.1|	putative ripening related protein [Cicer arietinum]	2E-41

Contig655	561	475	7	0	7	gb|AAY54007.1|	subtilisin-like protease [Arachis hypogaea]	3E-26
Contig656	642	554	10	0	10	gb|AAM65274.1|	NADH dehydrogenase [Arabidopsis thaliana]	1E-33
Contig657	446	420	2	0	2	gb|AAD45425.1|	gibberellin 2-oxidase [Pisum sativum]	4E-62
Contig658	148	122	2	0	2	gb|AC136472.40|	Medicago truncatula clone mth2-24f21, complete sequence	1E-11
Contig659	701	659	6	0	6	emb|CAN63851.1|	hypothetical protein [Vitis vinifera]	4E-56
Contig660	208	182	2	0	2	gb|ABE94198.1|	NAD-dependent epimerase/dehydratase [Medicago truncatula]	3E-27
Contig661	314	267	2	0	2	gb|DQ251457.1|	Siniperca chuatsi transposase mRNA, partial cds	0.00002
Contig662	444	420	2	0	2	gb|ABE93914.1|	Protein phosphatase 2C [Medicago truncatula]	1E-68
Contig663	295	269	7	1	6	gb|ABE78148.1|	Heat shock protein DnaJ [Medicago truncatula] gb|ABE83817.1| Heat shock protein DnaJ [Medicago truncatula] gb|ABP02364.1| Heat shock protein DnaJ [Medicago truncatula]	8E-41
Contig664	280	260	2	0	2	gb|ABE78148.1|	Heat shock protein DnaJ [Medicago truncatula] gb|ABE83817.1| Heat shock protein DnaJ [Medicago truncatula] gb|ABP02364.1| Heat shock protein DnaJ [Medicago truncatula]	2E-35
Contig665	622	601	8	0	8	emb|CAH55772.1|	putative His-Asp phosphotransfer protein [Pisum sativum]	5E-55
Contig666	205	205	2	0	2	emb|AM473965.2|	Vitis vinifera contig VV78X014869.2, whole genome shotgun sequence	9E-13
Contig667	424	396	2	0	2	ref|NP_567984.1|	pantothenate kinase family protein [Arabidopsis thaliana] gb|AAL32984.1| unknown protein [Arabidopsis thaliana] gb|AAN28872.1| At4g35360/F23E12_80 [Arabidopsis thaliana]	4E-38
Contig668	416	351	8	2	6	gb|AAK11734.1|	serine/threonine/tyrosine kinase [Arachis hypogaea]	5E-56
Contig669	152	128	2	0	2	emb|CAB50768.1|	cytochrome P450 [Cicer arietinum]	1E-13
Contig670	415	391	3	0	3	gb|AAL17948.1|	phosphoinositide-specific phospholipase C [Medicago truncatula] gb|ABE91697.1| Phosphoinositide-specific phospholipase C (PLC) [Medicago truncatula]	4E-71
Contig671	320	254	4	0	4	ref|XM_381601.1|	Gibberella zeae PH-1 chromosome 1 PMA1_NEUCR Plasma membrane ATPase (Proton pump) (FG01425.1) partial mRNA	2E-33
Contig672	381	342	2	0	2	gb|ABO82672.1|	Protein of unknown function DUF568 [Medicago truncatula]	1E-59
Contig673	633	406	4	0	4	sp|Q00016|	IFR_CICAR Isoflavone reductase (IFR) (2'-hydroxyisoflavone reductase)	7E-35
Contig674	461	437	6	5	1	gb|AAD37376.1|AF145350_1	peroxidase [Glycine max]	3E-58
Contig675	383	344	2	0	2	emb|CAA07232.1|	putative Pi starvation-induced protein [Cicer arietinum]	1E-30
Contig676	483	457	4	0	4	emb|CAA72315.1|	putative 21kD protein precursor [Medicago sativa subsp. x varia]	1E-59

Contig677	418	400	4	0	4	gb|ABC69764.1|	unknown [Vitis pseudoreticulata]	1E-34
Contig678	441	420	5	4	1	dbj|BAF31848.1|	nitrite reductase [Fusarium oxysporum]	6E-78
Contig679	384	358	3	0	3	ref|XP_390294.1|	ILV5_NEUCR Ketol-acid reductoisomerase, mitochondrial precursor (Acetohydroxy-acid reductoisomerase) (Alpha-keto-beta-hydroxylacil reductoisomerase) [Gibberella zeae PH-1]	2E-39
Contig680	1009	985	133	18	115	dbj|BAF01964.1|	hypothetical protein [Arabidopsis thaliana]	3E-68
Contig681	111	111	5	0	5	gb|AF075691.1|	gigas BAT1 homolog mRNA, complete cds	3E-08
Contig682	346	346	2	0	2	sp|Q5W915|USP_PEA	UDP-sugar pyrophospharylase (PsUSP) dbj|BAD66876.1| UDP-sugar pyrophospharylase [Pisum sativum]	6E-18
Contig683	760	712	4	0	4	gb|AAL17949.1|	type IIB calcium ATPase [Medicago truncatula]	9E-83
Contig684	471	445	2	0	2	gb|AF537102.1|	Plasmodiophora brassicae 16S ribosomal RNA gene, partial sequence; mitochondrial gene for mitochondrial product	0.00003
Contig685	426	402	3	0	3	emb|CAN62058.1|	hypothetical protein [Vitis vinifera]	7E-19
Contig686	455	429	2	0	2	dbj|BAA13683.1|	O-methyltransferase [Glycyrrhiza echinata]	9E-40
Contig687	282	238	2	0	2	gb|ABE89792.1|	Peptidase S10, serine carboxypeptidase [Medicago truncatula]	2E-23
Contig688	473	447	8	1	7	gb|AAQ09002.1|	hypothetical protein [Phaseolus vulgaris]	2E-17
Contig689	493	467	2	1	1	emb|CAN78056.1|	hypothetical protein [Vitis vinifera]	2E-27
Contig690	843	798	6	0	6	emb|CAA78515.1|	dehydrin-cognate [Pisum sativum]	1E-13
Contig691	787	719	8	2	6	gb|ABE84071.1|	Nucleic acid-binding, OB-fold, subgroup [Medicago truncatula]	1E-77
Contig692	456	430	5	0	5	gb|DQ459385.1|	Nicotiana tabacum serine/threonine kinase mRNA, partial cds	0.0005
Contig693	449	449	2	0	2	gb|ABK42077.1|	ubiquitin extension protein [Capsicum annuum]	4E-54
Contig694	751	727	5	0	5	gb|AAP33475.1|	polygalacturonase-like protein [Fragaria x ananassa]	1E-119
Contig695	316	292	3	0	3	emb|CR954195.3|	Medicago truncatula chromosome 5 clone mth2-16p13, COMPLETE SEQUENCE	9E-11
Contig696	311	288	4	0	4	sp|O65751|RSSA_CICAR	40S ribosomal protein SA (p40) emb|CAA07226.1| ribosome-associated protein p40 [Cicer arietinum]	3E-51
Contig697	457	431	4	0	4	gb|ABE83264.1|	Isocitrate dehydrogenase NADP-dependent, plant [Medicago truncatula]	4E-76
Contig698	602	560	4	0	4	emb|CAN81488.1|	hypothetical protein [Vitis vinifera]	2E-48
Contig699	223	197	2	0	2	gb|DQ251457.1|	Siniperca chuatsi transposase mRNA, partial cds	0.000001
Contig700	320	268	2	1	1	gb|DQ459385.1|	Nicotiana tabacum serine/threonine kinase mRNA, partial cds	0.000001
Contig701	293	272	2	1	1	ref|XM_390948.1|	Gibberella zeae PH-1 chromosome 3 hypothetical protein (FG10772.1) partial mRNA	0.00008

Contig702	677	635	5	1	4	gb|ABE86297.1|	Ubiquitin; Apoptosis regulator Bcl-2 protein, BAG [Medicago truncatula]	2E-75
Contig703	415	389	4	0	4	dbj|BAD95044.1|	serine/threonine kinase [Arabidopsis thaliana]	4E-22
Contig704	608	563	11	8	3	gb|ABA86966.1|	triosephosphate isomerase [Glycine max]	3E-72
Contig705	176	158	2	0	2	emb|CT028654.1|	Poplar cDNA sequences	0.044
Contig706	338	317	2	0	2	gb|ABE94198.1|	NAD-dependent epimerase/dehydratase [Medicago truncatula]	9E-56
Contig707	567	546	6	1	5	gb|AF155232.1|AF155232	Pisum sativum extensin (Ext) mRNA, complete cds	3E-46
Contig708	816	792	5	0	5	emb|CAA92583.1|	cysteine protease [Pisum sativum]	1E-129
Contig709	384	360	2	0	2	gb|AAZ23107.1|	plastid ATP/ADP transport protein 2 [Manihot esculenta]	3E-62
Contig710	538	491	7	2	5	gb|ABE89853.1|	Thioredoxin-related; Thioredoxin domain 2 [Medicago truncatula]	2E-62
Contig711	407	353	3	0	3	ref|NP_190005.1|	pyridine nucleotide-disulphide oxidoreductase family protein [Arabidopsis thaliana] emb|CAB88427.1| putative protein [Arabidopsis thaliana] gb|AAM61214.1| unknown [Arabidopsis thaliana] gb|AAN72037.1| putative protein [Arabidopsis thaliana] gb|AAP37810.1| At3g44190 [Arabidopsis thaliana]	1E-34
Contig712	256	232	2	0	2	emb|CAA10134.1|	basic blue copper protein [Cicer arietinum]	1E-30
Contig713	345	319	2	0	2	sp|Q8LJS2|HDT1_SOYBN	Histone deacetylase HDT1 (Histone deacetylase 2a) (HD2a) (Nucleolar histone deacetylase HD2-p39) gb|AAN03465.1| nucleolar histone deacetylase HD2-P39 [Glycine max]	1E-16
Contig714	627	603	5	0	5	sp|P49613|METL_PEA	S-adenosylmethionine synthetase 2 (Methionine adenosyltransferase 2) (AdoMet synthetase 2) emb|CAA57581.1| methionine adenosyltransferase [Pisum sativum] gb|AAA58773.1| S-adenosylmethionine synthase	1E-111
Contig715	747	702	7	3	4	gb|AAS47511.1|	ribosomal protein S6 [Glycine max]	8E-92
Contig716	460	436	3	0	3	emb|CU075908.9|	M.truncatula DNA sequence from clone MTH2-31B23 on chromosome 3, complete sequence	2E-28
Contig717	343	317	2	1	1	gb|ABE77920.1|	Rhodanese-like [Medicago truncatula] gb|ABE92275.1| Rhodanese-like [Medicago truncatula]	4E-37
Contig718	330	286	2	0	2	sp|Q42908|PMGI_MESCR	2,3-bisphosphoglycerate-independent phosphoglycerate mutase (Phosphoglyceromutase) (BPG-independent PGAM) (PGAM-I) gb|AAA86979.1| phosphoglyceromutase	1E-42
Contig719	568	542	8	3	5	gb|AAQ20041.1|	isoflavone 3'-hydroxylase [Medicago truncatula]	2E-62
Contig720	526	502	10	4	6	gb|ABE84254.1|	Universal stress protein (Usp) [Medicago truncatula] gb|ABE88281.1| Universal stress protein (Usp) [Medicago truncatula]	2E-72
Contig721	513	466	8	0	8	emb|CAA04447.1|	DnaJ-like protein [Medicago sativa] gb|AAC19391.1| DnaJ-like protein MsJ1 [Medicago sativa]	3E-69
Contig722	535	488	230	151	79	emb|AJ608703.3|	Fusarium oxysporum f. sp. lycopersici six1 gene, fot5 gene, six2 gene, shh1 gene and ORF2 (partial)	1E-117

Contig723	294	270	3	0	3	emb|CAA62227.1|	peroxidase1C [Medicago sativa]	5E-40
Contig724	706	685	6	3	3	gb|AAB99755.1|	malate dehydrogenase precursor [Medicago sativa]	3E-68
Contig725	342	316	2	1	1	gb|AAP37978.1|	class 10 pathogenesis-related protein [Lupinus luteus]	2E-22
Contig726	448	424	2	0	2	gb|ABE87796.1|	Zinc finger, RING-type; RINGv [Medicago truncatula]	8E-66
Contig727	926	902	8	0	8	emb|CAN73348.1|	hypothetical protein [Vitis vinifera]	1E-160
Contig728	454	454	2	0	2	gb|ABE84969.1|	Ribosomal protein L19e [Medicago truncatula]	1E-17
Contig729	228	204	2	0	2	emb|CAN67946.1|	hypothetical protein [Vitis vinifera]	2E-25
Contig730	448	422	2	0	2	gb|ABE93325.2|	Peptidase S10, serine carboxypeptidase [Medicago truncatula]	6E-30
Contig731	326	308	2	0	2	emb|CAN77737.1|	hypothetical protein [Vitis vinifera]	4E-25
Contig732	321	300	3	1	2	dbj|BAD97439.1|	peroxidase [Pisum sativum]	3E-36
Contig733	451	431	5	2	3	gb|ABO84368.1|	Cell division protein FtsZ [Medicago truncatula]	3E-66
Contig734	453	427	8	0	8	gb|AAL16064.1|AF420238_1	S-adenosyl-L-methionine synthetase [Dendrobium crumenatum]	4E-66
Contig735	1336	1303	20	4	16	emb|CAA48140.1|	ubiquitin [Antirrhinum majus]	1E-136
Contig736	757	739	7	3	4	dbj|BAC65212.1|	phosphate permease [Fusarium oxysporum]	8E-71
Contig737	112	112	2	0	2	gb|AC149135.2|	Medicago truncatula chromosome 2 clone mth2-31g23, complete sequence	1E-16
Contig738	237	216	5	1	4	dbj|BAA76417.1|	chalcone reductase [Cicer arietinum]	8E-24
Contig739	458	438	12	5	7	ref|XP_384488.1|	ATPB_NEUCR ATP synthase beta chain, mitochondrial precursor [Gibberella zeae PH-1]	2E-47
Contig740	467	446	3	0	3	gb|ABO15567.1|	cysteine synthase [Glycine max]	2E-43
Contig741	672	658	18	0	18	gb|ABE84165.2|	5-methyltetrahydropteroyltriglutamate--homocysteine S-methyltransferase; Prismane-like [Medicago truncatula] gb|ABE81639.2| 5-methyltetrahydropteroyltriglutamate--homocysteine S-methyltransferase; Prismane-like [Medicago truncatula]	1E-111
Contig742	348	304	2	0	2	gb|AAF26124.1|AC012328_27	unknown protein [Arabidopsis thaliana]	4E-17
Contig743	427	377	3	0	3	gb|AAB94584.1|	CYP71A10 [Glycine max]	8E-28
Contig744	387	363	5	0	5	gb|ABP03363.1|	RNA-binding region RNP-1 (RNA recognition motif) [Medicago truncatula]	2E-58
Contig745	723	699	8	0	8	gb|AAK66766.1|AF386739_1	aquaporin protein PIP1;1 [Medicago truncatula]	3E-94
Contig746	269	243	2	0	2	gb|ABE92907.2|	Peptidase S24, S26A and S26B [Medicago truncatula]	2E-12

Contig747	160	134	2	0	2	emb|CAB52812.1|	Ribosomal protein L7Ae-like (fragment) [Arabidopsis thaliana] emb|CAB79193.1| Ribosomal protein L7Ae-like (fragment) [Arabidopsis thaliana]	4E-14
Contig748	483	457	2	0	2	ref|NP_191771.1|	tRNA synthetase class II (G, H, P and S) family protein [Arabidopsis thaliana] ref|NP_850736.1| tRNA synthetase class II (G, H, P and S) family protein [Arabidopsis thaliana] emb|CAB71872.1| multifunctional aminoacyl-tRNA ligase-like protein [Arabidopsis thaliana] gb|AAL24294.1| multifunctional aminoacyl-tRNA ligase-like protein [Arabidopsis thaliana] gb|AAM91120.1| multifunctional aminoacyl-tRNA ligase-like protein [Arabidopsis thaliana]	2E-82
Contig749	727	678	3	0	3	gb|AAO39834.1|	ferrous ion membrane transport protein DMT1 [Glycine max]	1E-67
Contig750	392	365	70	62	8	ref|XP_381334.1|	hypothetical protein FG01158.1 [Gibberella zeae PH-1]	2E-22
Contig751	419	393	4	1	3	gb|AAF65513.1|	GTP-binding protein [Capsicum annuum]	3E-63
Contig752	461	436	2	0	2	ref|NP_568466.1|	CIPK25 (CBL-INTERACTING PROTEIN KINASE 25); kinase [Arabidopsis thaliana] gb|AAL41008.1|AF448226_1 CBL-interacting protein kinase CIPK25 [Arabidopsis thaliana]	2E-64
Contig753	453	427	5	0	5	gb|AC187294.1|	Glycine max clone gmw1-105h23, complete sequence	2E-21
Contig754	694	670	3	0	3	dbj|BAE71301.1|	putative arginine decarboxylase [Trifolium pratense]	1E-104
Contig755	189	165	2	0	2	gb|ABN07990.1|	SAM (and some other nucleotide) binding motif [Medicago truncatula]	8E-20
Contig756	293	269	2	0	2	emb|AJ006767.1|CAR6767	Cicer arietinum mRNA for histone H1	1E-137
Contig757	446	412	5	2	3	emb|CAN73350.1|	hypothetical protein [Vitis vinifera]	6E-68
Contig758	264	240	2	0	2	sp|P35694|BRU1_SOYBN	Brassinosteroid-regulated protein BRU1 precursor gb|AAA81350.1| brassinosteroid-regulated protein	8E-31
Contig759	257	236	2	0	2	gb|AAQ21120.1|	early light inducible protein [Trifolium pratense]	6E-37
Contig760	333	312	2	0	2	emb|CAN80774.1|	hypothetical protein [Vitis vinifera]	1E-14
Contig761	112	112	2	0	2	gb|DQ990796.1|	Hetreulophus sp. CD021 enolase mRNA, partial cds	0.0001
Contig762	384	358	2	0	2	emb|AJ608703.3|	Fusarium oxysporum f. sp. lycopersici six1 gene, fot5 gene, six2 gene, shh1 gene and ORF2 (partial)	3E-91
Contig763	596	596	7	2	5	emb|CAN68131.1|	hypothetical protein [Vitis vinifera]	1E-10
Contig764	568	523	7	0	7	emb|CAB71128.2|	cationic peroxidase [Cicer arietinum]	1E-84
Contig765	291	222	4	1	3	gb|CP000302.1|	Shewanella denitrificans OS217, complete genome	0.3
Contig766	208	184	2	0	2	emb|CT029636.1|	Poplar cDNA sequences	0.053
Contig767	351	325	3	0	3	gb|ABD32718.1|	O-methyltransferase, family 2; Dimerisation [Medicago truncatula]	8E-28

Contig768	387	363	2	0	2	dbj|BAE71240.1|	putative cytoplasmic aconitate hydratase [Trifolium pratense]	4E-60
Contig769	415	389	3	0	3	ref|XP_382875.1|	hypothetical protein FG02699.1 [Gibberella zeae PH-1]	1E-29
Contig770	192	171	5	3	2	emb|AM748504.1|	Vigna unguiculata partial mRNA for putative carbamoyl-phosphate synthase, GATase region (MtrDRAFT_AC135505g12v2 gene), clone 49	0.0008
Contig771	383	357	2	0	2	gb|ABA29157.1|	unknown [Pisum sativum]	7E-35
Contig772	342	318	2	0	2	emb|Y15372.1|MTY15372	Medicago truncatula mRNA for MtN4 gene, partial	1E-28
Contig773	462	436	6	2	4	gb|ABE84488.1|	Plant lipid transfer/seed storage/trypsin-alpha amylase inhibitor [Medicago truncatula]	1E-40
Contig774	406	363	3	0	3	gb|AC121245.17|	Medicago truncatula clone mth2-33g3, complete sequence	1E-19
Contig775	489	467	3	0	3	emb|CAN61836.1|	hypothetical protein [Vitis vinifera]	2E-56
Contig776	119	119	2	0	2	gb|AF020273.1|AF020273	Medicago sativa nodule-enhanced malate dehydrogenase precursor (nemdh) mRNA, complete cds	1E-29
Contig777	202	176	2	1	1	ref|XP_389679.1|	hypothetical protein FG09503.1 [Gibberella zeae PH-1]	2E-24
Contig778	455	429	3	0	3	gb|EAZ24782.1|	hypothetical protein OsJ_008265 [Oryza sativa (japonica cultivar-group)]	1E-16
Contig779	563	539	4	0	4	gb|AAN74636.1|	DEAD box RNA helicase [Pisum sativum]	1E-89
Contig780	455	431	3	1	2	emb|CAA10131.1|	chalcone synthase [Cicer arietinum]	3E-51
Contig781	336	310	2	0	2	emb|CAN69812.1|	hypothetical protein [Vitis vinifera]	1E-19
Contig782	202	176	2	1	1	gb|BT009458.1|	Triticum aestivum clone wlsu2.pk0001.h3:fis, full insert mRNA sequence	0.0002
Contig783	510	467	4	0	4	gb|ABE81662.1|	serine C-palmitoyltransferase like protein [imported] - Arabidopsis thaliana-related [Medicago truncatula]	8E-40
Contig784	456	430	2	0	2	emb|CAN67205.1|	hypothetical protein [Vitis vinifera]	3E-49
Contig785	318	297	2	0	2	gb|ABO87610.1|	chloroplast ferredoxin-NADP+ reductase [Pisum sativum]	2E-51
Contig786	459	435	4	0	4	gb|ABE79257.1|	MIR [Medicago truncatula]	4E-79
Contig787	458	434	2	1	1	gb|ABE86786.1|	hypothetical protein MtrDRAFT_AC124956g13v2 [Medicago truncatula]	4E-66
Contig788	447	421	2	0	2	emb|CAN70143.1|	hypothetical protein [Vitis vinifera]	1E-20
Contig789	373	329	2	0	2	emb|AJ749795.1|	Photobacterium damselae subsp. piscicida partial ORF1 DNA for hypothetical protein, clone pRDA14	0.0004
Contig790	487	487	3	2	1	gb|ABE91892.1|	Ras small GTPase, Ras type; Small GTP-binding protein domain [Medicago truncatula]	4E-72
Contig791	106	106	2	0	2	gb|AC152347.14|	Medicago truncatula clone mth2-28c12, complete sequence	0.0004

Contig792	416	398	2	0	2	ref|NP_001062502.1|	Os08g0559400 [Oryza sativa (japonica cultivar-group)] dbj|BAB62328.1| cyclophilin [Oryza sativa (japonica cultivar-group)] dbj|BAB62329.1| cyclophilin [Oryza sativa (japonica cultivar-group)] dbj|BAD13138.1| cyclophilin [Oryza sativa (japonica cultivar-group)] dbj|BAF24416.1| Os08g0559400 [Oryza sativa (japonica cultivar-group)] gb|EAZ43652.1| hypothetical protein OsJ_027135 [Oryza sativa (japonica cultivar-group)]	2E-45
Contig793	375	351	3	0	3	gb|ABE84165.2|	5-methyltetrahydropteroyltriglutamate--homocysteine S-methyltransferase; Prismane-like [Medicago truncatula] gb|ABE81639.2| 5-methyltetrahydropteroyltriglutamate--homocysteine S-methyltransferase; Prismane-like [Medicago truncatula]	2E-43
Contig794	460	436	3	0	3	gb|ABE91564.1|	Ubiquitin-conjugating enzyme, E2 [Medicago truncatula]	6E-26
Contig795	459	433	3	0	3	gb|ABE82378.1|	Aldehyde dehydrogenase [Medicago truncatula] gb|ABE91820.1| Aldehyde dehydrogenase [Medicago truncatula]	2E-77
Contig796	304	304	2	1	1	gb|ABE84969.1|	Ribosomal protein L19e [Medicago truncatula]	8E-39
Contig797	444	420	2	0	2	emb|CAN64127.1|	hypothetical protein [Vitis vinifera]	2E-18
Contig798	424	403	4	0	4	gb|AAU44342.1|	monodehydroascorbate reductase II [Pisum sativum]	5E-57
Contig799	516	489	3	0	3	dbj|BAE71208.1|	hypothetical protein [Trifolium pratense] dbj|BAE71210.1| hypothetical protein [Trifolium pratense]	3E-65
Contig800	453	418	3	0	3	gb|ABO78563.1|	Glycine cleavage system P-protein [Medicago truncatula]	7E-69
Contig801	167	149	2	0	2	emb|CU013517.1|	Medicago truncatula chromosome 5 clone mth2-76o9, COMPLETE SEQUENCE	0.000003
Contig802	412	394	13	0	13	gb|ABO79321.1|	Ribosomal protein S24e [Medicago truncatula]	3E-18
Contig803	340	298	5	0	5	gb|ABO78644.1|	Protein of unknown function DUF543 [Medicago truncatula]	1E-21
Contig804	186	144	2	0	2	gb|ABE81243.1|	Malic oxidoreductase [Medicago truncatula]	2E-19
Contig805	457	431	3	0	3	gb|AY874423.1|	Fusarium oxysporum voucher VPRI 19292 mitochondrion, partial genome	0
Contig806	187	169	2	0	2	ref|XP_381601.1|	PMA1_NEUCR Plasma membrane ATPase (Proton pump) [Gibberella zeae PH-1]	1E-24
Contig807	360	311	2	0	2	emb|X93220.2|CACG2	Cicer arietinum partial mRNA for cysteine proteinase (cacG2 gene)	1E-110
Contig808	326	302	2	0	2	emb|AJ749797.1|	Photobacterium damselae subsp. piscicida trpB gene for putative transposase, clone pRDA16	0.00009
Contig809	463	439	4	0	4	gb|AC139708.15|	Medicago truncatula clone mth2-9f16, complete sequence	6E-16
Contig810	313	266	3	0	3	gb|ABO81713.1|	Ribosomal protein L36E [Medicago truncatula]	6E-42
Contig811	459	433	2	0	2	gb|AAQ74889.1|	Al-induced protein [Gossypium hirsutum]	1E-60
Contig812	359	312	2	0	2	gb|DQ251457.1|	Siniperca chuatsi transposase mRNA, partial cds	0.000006
Contig813	374	342	2	0	2	emb|CAD29733.1|	pectin methylesterase [Sesbania rostrata]	4E-54

Contig814	428	404	2	1	1	gb|AAT94362.1|	putative chalcone isomerase 4 [Glycine max]	7E-54
Contig815	448	328	4	0	4	gb|ABE90033.1|	Protein kinase [Medicago truncatula]	4E-13
Contig816	423	352	4	0	4	gb|ABE85164.1|	hypothetical protein MtrDRAFT_AC119415g11v2 [Medicago truncatula]	5E-19
Contig817	224	203	2	0	2	sp|Q42800|DAPA_SOYBN	Dihydrodipicolinate synthase, chloroplast precursor (DHDPS) gb|AAA73555.1| dihydrodipicolinate synthase	3E-24
Contig818	427	403	2	0	2	gb|ABI34274.1|	IS10 transposase, putative [Lycopersicon esculentum]	1E-27
Contig819	358	315	2	0	2	gb|ABO84303.1|	LOC548366 protein, related [Medicago truncatula]	1E-50
Contig820	160	135	2	0	2	emb|CT027663.2|	Medicago truncatula chromosome 5 clone mth2-139g23, COMPLETE SEQUENCE	3E-46
Contig821	477	456	3	0	3	gb|DQ383816.1|	Lactuca sativa cultivar Salinas chloroplast, complete genome	5E-38
Contig822	467	443	4	0	4	ref|NP_001065513.1|	Os10g0580400 [Oryza sativa (japonica cultivar-group)] gb|AAG46170.1|AC018727_22 putative urea active transport protein [Oryza sativa] gb|AAP55189.1| SSS sodium solute transporter superfamily protein, expressed [Oryza sativa (japonica cultivar-group)] gb|AAR27948.1| DUR3 [Oryza sativa (japonica cultivar-group)] dbj|BAF27350.1| Os10g0580400 [Oryza sativa (japonica cultivar-group)] gb|EAZ17111.1| hypothetical protein OsJ_031320 [Oryza sativa (japonica cultivar-group)]	2E-50
Contig823	451	451	11	0	11	gb|AAY26520.1|	secretory peroxidase [Catharanthus roseus]	6E-49
Contig824	474	474	5	0	5	gb|AAZ50401.1|	Sus [Agrobacterium tumefaciens]	3E-34
Contig825	289	268	2	0	2	gb|DQ485193.1|	Nerium oleander microsatellite CATR25 sequence	3E-63
Contig826	251	230	2	0	2	dbj|AB262513.1|	Pseudomonas aeruginosa gene for 16S rRNA, partial sequence, strain: Hg2	9E-29
Contig827	654	600	9	2	7	gb|ABE86679.1|	Intracellular chloride channel [Medicago truncatula]	4E-77
Contig828	406	380	5	2	3	gb|ABM69111.1|	phosphate transporter 5 [Medicago truncatula]	4E-45
Contig829	410	336	3	0	3	gb|AAC32158.1|	hypothetical protein [Picea mariana]	1E-13
Contig830	383	345	3	0	3	emb|AJ487465.1|CAR487465	Cicer arietinum mRNA for putative quinone oxidoreductase (qor gene)	1E-119
Contig831	902	837	10	0	10	gb|AAT45084.1|	proline dehydrogenase [Medicago sativa] gb|AAT45085.1| proline dehydrogenase [Medicago sativa]	1E-137
Contig832	391	367	3	0	3	emb|CAN81471.1|	hypothetical protein [Vitis vinifera]	8E-28
Contig833	746	732	6	4	2	dbj|BAB32793.1|	110 kDa 4SNc-Tudor domain protein [Pisum sativum]	8E-68
Contig834	386	313	7	0	7	emb|CAA56142.1|	pathogenesis related protein [Cicer arietinum]	4E-20
Contig835	227	201	2	0	2	gb|AC174308.7|	Medicago truncatula clone mth2-139i23, complete sequence	0.00006

Contig836	479	479	3	0	3	gb|AAD39439.1|AF132001_1	PHAP2A protein [Petunia x hybrida]	9E-33
Contig837	461	461	10	0	10	gb|AAY26520.1|	secretory peroxidase [Catharanthus roseus]	3E-80
Contig838	201	179	3	0	3	gb|AY099112.1|	Rattus norvegicus obese protein gene, 5' flanking region and partial cds	6E-11
Contig839	840	840	6	0	6	gb|AAD33922.1|AF143954_1	agglutinin [Amaranthus hypochondriacus] emb|CAA77664.1| seed specific protein of balanced nutritional quality [Amaranthus hypochondriacus]	1E-163
Contig840	576	531	11	4	7	gb|ABE88774.1|	Translation factor; Elongation factor G, III and V [Medicago truncatula]	6E-97
Contig841	450	424	2	0	2	gb|DQ251457.1|	Siniperca chuatsi transposase mRNA, partial cds	0.000008
Contig842	489	489	3	0	3	gb|ABE78306.1|	IQ calmodulin-binding region [Medicago truncatula]	2E-59
Contig843	313	266	2	0	2	gb|AAL86349.1|	unknown protein [Arabidopsis thaliana]	2E-31
Contig844	868	824	32	1	31	sp|P34921|G3PC_DIACA	Glyceraldehyde-3-phosphate dehydrogenase, cytosolic	1E-137
Contig845	464	440	2	0	2	gb|AAO61674.1|	AKIN gamma [Medicago truncatula]	4E-72
Contig846	372	339	3	0	3	dbj|BAB88648.1|	microtubule bundling polypeptide TMBP200 [Nicotiana tabacum]	7E-40
Contig847	445	379	7	1	6	gb|ABE85391.1|	S25 ribosomal protein [Medicago truncatula] gb|ABE85967.1| S25 ribosomal protein [Medicago truncatula]	1E-29
Contig848	391	367	2	0	2	gb|AC139854.21|	Medicago truncatula clone mth2-16e16, complete sequence	7E-43
Contig849	703	627	10	4	6	gb|ABE82912.1|	Ribosomal protein S4, bacterial and organelle form [Medicago truncatula]	1E-64
Contig850	361	337	2	1	1	gb|ABE85139.1|	TPR repeat [Medicago truncatula]	7E-21
Contig851	322	283	3	0	3	gb|ABE90991.1|	Heat shock protein DnaJ, N-terminal; Tetratricopeptide-like helical [Medicago truncatula]	4E-18
Contig852	595	553	4	0	4	ref|NP_200679.1|	ROC7 (rotamase CyP 7); peptidyl-prolyl cis-trans isomerase [Arabidopsis thaliana] sp|Q9SP02|CP20A_ARATH Peptidyl-prolyl cis-trans isomerase CYP20-1 precursor (PPIase CYP20-1) (Rotamase cyclophilin-7) (Cyclophilin of 20 kDa 1) gb|AAF05760.1|AF192490_1 cyclophilin [Arabidopsis thaliana] dbj|BAA97339.1| cyclophilin [Arabidopsis thaliana] gb|AAK82490.1| AT5g58710/mzn1_160 [Arabidopsis thaliana] gb|AAM16173.1| AT5g58710/mzn1_160 [Arabidopsis thaliana] gb|AAM63473.1| cyclophilin ROC7 [Arabidopsis thaliana]	5E-78
Contig853	275	226	2	0	2	emb|CAN82123.1|	hypothetical protein [Vitis vinifera]	3E-30
Contig854	571	547	8	0	8	emb|CAA10132.1|	superoxide dismutase [Cicer arietinum] emb|CAA10160.1| superoxide dismutase [Cicer arietinum]	4E-76
Contig855	477	430	5	0	5	gb|ABE90729.1|	Myosin II heavy chain-like [Medicago truncatula] gb|ABO80686.1| Myosin II heavy chain-like [Medicago truncatula]	7E-58
Contig856	314	296	2	1	1	emb|CT029620.1|	Poplar cDNA sequences	0.021

Contig857	332	308	8	0	8	dbj|BAC22609.1|	41 kD chloroplast nucleoid DNA binding protein (CND41) [Nicotiana sylvestris]	3E-30
Contig858	349	307	2	0	2	sp|Q9M4D8|DCAM_VICFA	S-adenosylmethionine decarboxylase proenzyme (AdoMetDC) (SamDC) [Contains: S-adenosylmethionine decarboxylase alpha chain; S-adenosylmethionine decarboxylase beta chain] emb|CAB76966.1| S-adenosylmethionine decarboxylase [Vicia faba]	2E-39
Contig859	333	309	2	0	2	ref|NP_564405.1|	LOL1 (LSD ONE LIKE 1) [Arabidopsis thaliana] gb|AAL15306.1| At1g32540/T9G5_1 [Arabidopsis thaliana] gb|AAM51585.1| At1g32540/T9G5_1 [Arabidopsis thaliana] gb|AAQ55219.1| LSD1-like [Arabidopsis thaliana]	6E-20
Contig860	433	433	3	0	3	emb|CU406989.5|	Mouse DNA sequence from clone CH29-63I6 on chromosome 11, complete sequence	1.8
Contig861	230	204	2	0	2	gb|EAY96565.1|	hypothetical protein OsI_017798 [Oryza sativa (indica cultivar-group)] gb|EAZ32893.1| hypothetical protein OsJ_016376 [Oryza sativa (japonica cultivar-group)]	7E-19
Contig862	487	487	5	0	5	ref|NP_564367.1|	integral membrane HRF1 family protein [Arabidopsis thaliana] ref|NP_001077633.1| integral membrane HRF1 family protein [Arabidopsis thaliana] gb|AAK64057.1| unknown protein [Arabidopsis thaliana] gb|AAM44952.1| unknown protein [Arabidopsis thaliana] gb|AAM63447.1| unknown [Arabidopsis thaliana]	1E-45
Contig863	448	417	2	0	2	gb|AAP03880.2|	Avr9/Cf-9 induced kinase 1 [Nicotiana tabacum]	2E-40
Contig864	840	814	4	0	4	gb|ABO77440.1|	S-adenosylmethionine decarboxylase [Medicago sativa subsp. falcata]	1E-114
Contig865	448	422	2	0	2	ref|NP_568117.1|	phosphoadenosine phosphosulfate (PAPS) reductase family protein [Arabidopsis thaliana] emb|CAB83302.1| putative protein [Arabidopsis thaliana]	4E-45
Contig866	408	390	6	0	6	emb|CAA80983.1|	narbonin [Vicia narbonensis]	7E-37
Contig867	341	315	2	0	2	gb|AAG23130.1|	diacylglycerol kinase variant A [Lycopersicon esculentum]	2E-19
Contig868	192	163	2	0	2	gb|ABC59084.1|	cytochrome P450 monooxygenase CYP83G1 [Medicago truncatula]	2E-15
Contig869	449	449	2	0	2	emb|CAN67313.1|	hypothetical protein [Vitis vinifera]	6E-29
Contig870	437	413	2	0	2	gb|ABE85996.1|	Glycoside hydrolase, family 1 [Medicago truncatula]	8E-52
Contig871	380	354	2	0	2	gb|ABE78982.1|	Ras small GTPase, Rab type [Medicago truncatula]	2E-58
Contig872	307	281	2	0	2	emb|AM422095.1|	Danio rerio partial mRNA for vox protein (vox gene)	0.00002
Contig873	623	574	7	0	7	emb|CAN71478.1|	hypothetical protein [Vitis vinifera]	1E-55
Contig874	300	276	2	0	2	gb|ABD32628.1|	Granulin; Peptidase C1A, papain [Medicago truncatula]	8E-31
Contig875	446	420	2	0	2	gb|ABH02852.1|	MYB transcription factor MYB112 [Glycine max]	7E-13
Contig876	580	539	7	1	6	gb|ABO84368.1|	Cell division protein FtsZ [Medicago truncatula]	7E-85

Contig877	288	260	2	0	2	ref|NP_850271.1|	ATGSL08 (GLUCAN SYNTHASE-LIKE 8); 1,3 beta-glucan synthase/ transferase, transferring glycosyl groups [Arabidopsis thaliana]	2E-32
Contig878	469	431	3	1	2	gb|AAM08880.1|AC113339_26	Hypothetical protein [Oryza sativa]	7E-16
Contig879	404	404	2	0	2	gb|ABE82897.1|	Acetohydroxy acid isomeroreductase [Medicago truncatula]	3E-61
Contig880	419	401	3	0	3	gb|AAP83926.1|	transaldolase [Lycopersicon esculentum]	4E-21
Contig881	306	279	2	0	2	emb|CT029528.1|	Poplar cDNA sequences	0.08
Contig882	521	500	6	5	1	gb|ABE84580.1|	fiber protein Fb15 [Medicago truncatula]	7E-38
Contig883	352	326	2	0	2	gb|ABE82737.1|	RNA-binding region RNP-1 (RNA recognition motif) [Medicago truncatula]	1E-36
Contig884	227	203	2	0	2	sp|O23948|VATE_GOSHI	Vacuolar ATP synthase subunit E (V-ATPase subunit E) (Vacuolar proton pump subunit E) gb|AAB72177.1| vacuolar H+-ATPase subunit E [Gossypium hirsutum]	2E-24
Contig885	400	354	2	0	2	gb|AAL49957.1|	GTP cyclohydrolase I [Lycopersicon esculentum]	3E-34
Contig886	224	224	2	0	2	emb|AJ006760.1|CAR6760	Cicer arietinum mRNA for hypothetical protein, clone Can107	1E-107
Contig887	454	428	2	0	2	emb|CAN81061.1|	hypothetical protein [Vitis vinifera]	2E-33
Contig888	125	101	2	0	2	emb|AM422119.2|	Danio rerio ca7 mRNA, 3' UTR	0.0001
Contig889	269	222	4	0	4	gb|ABE90867.1|	Ribosomal L18ae protein [Medicago truncatula]	2E-36
Contig890	466	440	2	0	2	gb|AAL01888.1|AF404404_1	acyl-CoA oxidase [Glycine max]	4E-60
Contig891	263	221	2	0	2	gb|ABE86663.2|	Zinc finger, RING-type [Medicago truncatula]	7E-32
Contig892	238	196	3	0	3	gb|AAV92899.1|	Avr9/Cf-9 rapidly elicited protein 140 [Nicotiana tabacum]	9E-16
Contig893	553	509	5	0	5	gb|AY874423.1|	Fusarium oxysporum voucher VPRI 19292 mitochondrion, partial genome	0
Contig894	403	366	2	0	2	dbj|BAE99924.1|	hypothetical protein [Arabidopsis thaliana]	5E-30
Contig895	457	431	2	0	2	gb|AAO72990.1|	cyclin D [Populus alba]	9E-56
Contig896	451	405	3	0	3	gb|AAT37529.1|	purple acid phosphatase 1 [Solanum tuberosum]	9E-32
Contig897	212	173	3	0	3	gb|AAR14273.1|	predicted protein [Populus alba x Populus tremula]	9E-27
Contig898	367	294	3	0	3	gb|DQ507301.1|	Belgica antarctica clone Ba-U40 CG32816-like mRNA, partial cds	0.000002

Contig899	453	427	2	0	2	ref|NP_187341.1|	DIN3/LTA1 (DARK INDUCIBLE 3); alpha-ketoacid dehydrogenase [Arabidopsis thaliana] ref|NP_850527.1| DIN3/LTA1 (DARK INDUCIBLE 3); alpha-ketoacid dehydrogenase [Arabidopsis thaliana] gb|AAF63813.1| branched chain alpha-keto acid dehydrogenase E2 subunit [Arabidopsis thaliana] gb|AAM63444.1| branched chain alpha-keto acid dehydrogenase E2 subunit [Arabidopsis thaliana]	2E-60
Contig900	455	431	2	1	1	gb|ABE78689.2|	AMP-dependent synthetase and ligase [Medicago truncatula]	3E-74
Contig901	418	400	3	0	3	ref|XM_001011326.2|	Tetrahymena thermophila SB210 hypothetical protein (TTHERM_00430020) mRNA, complete cds	0.028
Contig902	404	383	4	0	4	ref|XP_001257448.1|	cupin domain protein [Neosartorya fischeri NRRL 181] gb|EAW15551.1| cupin domain protein [Neosartorya fischeri NRRL 181]	1E-14
Contig903	344	297	3	0	3	gb|ABD28527.1|	Protein kinase [Medicago truncatula] gb|ABE81653.1| Protein kinase [Medicago truncatula]	1E-45
Contig904	449	422	3	0	3	dbj|BAE71227.1|	putative rubisco subunit binding-protein alpha subunit [Trifolium pratense]	3E-61
Contig905	445	423	5	0	5	sp|Q9ZSW9|TCTP_HEVBR	Translationally-controlled tumor protein homolog (TCTP) gb|AAD10032.1| translationally controlled tumor protein [Hevea brasiliensis]	3E-53
Contig906	570	544	6	2	4	gb|ABE87516.2|	Ribosomal protein L7Ae/L30e/S12e/Gadd45 [Medicago truncatula]	6E-58
Contig907	309	288	2	0	2	gb|ABE78365.1|	Metallophosphoesterase; Purple acid phosphatase, N-terminal [Medicago truncatula] gb|ABE86342.1| Metallophosphoesterase; Purple acid phosphatase, N-terminal [Medicago truncatula]	3E-47
Contig908	415	394	7	4	3	gb|ABE85050.1|	Universal stress protein (Usp) [Medicago truncatula]	3E-49
Contig909	406	380	2	0	2	gb|ABC59078.1|	cytochrome P450 monooxygenase CYP72A59 [Medicago truncatula]	3E-57
Contig910	422	401	3	0	3	gb|ABO79239.1|	KOB1 , putative [Medicago truncatula]	5E-20
Contig911	710	661	5	1	4	pdb|2P4H|X	Chain X, Crystal Structure Of Vestitone Reductase From Alfalfa (Medicago Sativa L.)	1E-110
Contig912	412	342	3	0	3	emb|X68649.1|PSACTG	P.sativum mRNA for actin	3E-45
Contig913	470	470	44	41	3	ref|XP_720736.1|	hypothetical protein CaO19_11777 [Candida albicans SC5314] ref|XP_720608.1| hypothetical protein CaO19_4301 [Candida albicans SC5314] gb|EAL01773.1| hypothetical protein CaO19.4301 [Candida albicans SC5314] gb|EAL01907.1| hypothetical protein CaO19.11777 [Candida albicans SC5314]	6E-76
Contig914	427	368	3	0	3	emb|CAN61918.1|	hypothetical protein [Vitis vinifera]	2E-12
Contig915	180	159	5	0	5	emb|AM422093.1|	Danio rerio partial mRNA for lecithin retinol acetyltransferase (lrat gene)	0.003
Contig916	365	323	6	4	2	gb|ABE80903.2|	Light chain 3 (LC3) [Medicago truncatula]	3E-33
Contig917	454	428	3	0	3	gb|ABE92135.1|	Glutathione S-transferase, C-terminal-like; Thioredoxin fold [Medicago truncatula]	3E-61
Contig918	406	360	2	0	2	gb|ABE81243.1|	Malic oxidoreductase [Medicago truncatula]	3E-22

Contig919	228	205	2	0	2	gb|AC149636.14|	Medicago truncatula clone mth2-180a12, complete sequence	0.0000002
Contig920	460	436	2	0	2	emb|CAN75020.1|	hypothetical protein [Vitis vinifera]	2E-29
Contig921	354	328	4	0	4	gb|ABE88774.1|	Translation factor; Elongation factor G, III and V [Medicago truncatula]	4E-38
Contig922	248	227	2	1	1	emb|CT009479.4|	M.truncatula DNA sequence from clone MTH2-164E3 on chromosome 3, complete sequence	2E-23
Contig923	426	405	2	0	2	gb|ABB72820.1|	oligouridylate binding protein-like protein [Solanum tuberosum]	5E-64
Contig924	424	398	3	0	3	gb|AC150244.2|	Medicago truncatula chromosome 7 clone mth2-180p14, complete sequence	0.000002
Contig925	674	632	4	0	4	dbj|BAD24713.1|	protein disufide isomerase-like protein [Glycine max]	3E-79
Contig926	323	296	8	0	8	gb|ABE92804.1|	Curculin-like (mannose-binding) lectin [Medicago truncatula]	2E-47
Contig927	425	404	2	0	2	gb|ABO80948.1|	S-adenosylmethionine synthetase [Medicago truncatula]	2E-73
Contig928	408	384	2	0	2	ref|NP_565408.1|	protein kinase, putative [Arabidopsis thaliana] gb|AAK43904.1|AF370585_1 putative protein kinase [Arabidopsis thaliana] gb|AAK83605.1| At2g17220/T23A1.8 [Arabidopsis thaliana] gb|AAD25140.2| putative protein kinase [Arabidopsis thaliana] gb|AAN31120.1| At2g17220/T23A1.8 [Arabidopsis thaliana]	5E-57
Contig929	503	503	3	0	3	gb|ABD33016.1|	Transcription Factor IIF, Rap30/Rap74, interaction [Medicago truncatula]	3E-68
Contig930	273	241	3	0	3	emb|CAN82178.1|	hypothetical protein [Vitis vinifera]	3E-37
Contig931	1249	1249	11	2	9	emb|CAA08906.1|	cysteine proteinase [Cicer arietinum]	0
Contig932	350	306	5	0	5	gb|EAZ28486.1|	hypothetical protein OsJ_011969 [Oryza sativa (japonica cultivar-group)]	9E-46
Contig933	417	391	2	1	1	gb|ABE89160.1|	O-methyltransferase, family 2; Dimerisation [Medicago truncatula]	1E-46
Contig934	351	333	2	1	1	ref|NP_181843.2|	DNA binding / transcription factor [Arabidopsis thaliana]	5E-24
Contig935	251	204	3	0	3	gb|ABA03227.1|	glyceraldehyde-3-phosphate dehydrogenase [Populus maximowiczii x Populus nigra]	4E-12
Contig936	488	440	6	1	5	sp|Q9FVL0|HBL1_MEDSA	Non-symbiotic hemoglobin 1 (MEDsa GLB1) gb|AAG29748.1|AF172172_1 non-symbiotic hemoglobin [Medicago sativa]	4E-59
Contig937	401	380	2	0	2	emb|CAN73618.1|	hypothetical protein [Vitis vinifera]	4E-24
Contig938	494	435	4	0	4	gb|ABE93220.1|	General substrate transporter [Medicago truncatula]	2E-67
Contig939	453	393	3	0	3	ref|XP_001216717.1|	predicted protein [Aspergillus terreus NIH2624] gb|EAU31269.1| predicted protein [Aspergillus terreus NIH2624]	1E-30
Contig940	506	480	4	1	3	gb|BT009458.1|	Triticum aestivum clone wlsu2.pk0001.h3:fis, full insert mRNA sequence	0.0006
Contig941	671	631	6	2	4	gb|ABE79318.1|	Plant lipid transfer/seed storage/trypsin-alpha amylase inhibitor [Medicago truncatula]	4E-33
Contig942	454	428	2	0	2	gb|AAC77929.1|	similar to Nicotiana HR lesion-inducing ORF [Medicago sativa]	4E-57

Contig943	216	190	2	0	2	sp|P49163|RK22_MEDSA	50S ribosomal protein L22, chloroplast precursor (CL22) gb|AAB46612.1| ribosomal protein CL22 [Medicago sativa]	1E-25
Contig944	375	270	2	0	2	gb|DQ459385.1|	Nicotiana tabacum serine/threonine kinase mRNA, partial cds	2E-15
Contig945	285	259	4	0	4	emb|AJ006770.1|CAR6770	Cicer arietinum mRNA for extensin, partial	1E-136
Contig946	409	388	2	0	2	gb|ABE92854.1|	Ribosomal L22e protein [Medicago truncatula]	3E-37
Contig947	517	478	4	0	4	gb|ABQ95992.1|	14-3-3-like protein [Cicer arietinum] gb|ABQ95994.1| 14-3-3-like protein [Cicer arietinum]	4E-64
Contig948	450	424	2	0	2	emb|CAB76913.1|	hypothetical protein [Cicer arietinum]	4E-74
Contig949	276	232	2	0	2	ref|XM_001309854.1|	Trichomonas vaginalis G3 variable membrane protein precursor, putative (TVAG_087500) mRNA, complete cds	0.28
Contig950	426	379	3	0	3	emb|AM706411.1|	Eristalis tenax partial mRNA for hypothetical protein (ORF1), isolate 3	0.0001
Contig951	439	406	3	0	3	emb|CAD91338.1|	beta-fructofuranosidase [Glycine max]	4E-61
Contig952	454	434	3	0	3	ref|NP_565024.1|	unknown protein [Arabidopsis thaliana]	5E-19
Contig953	425	374	2	0	2	sp|P22196|PER2_ARAHY	Cationic peroxidase 2 precursor (PNPC2) gb|AAA32676.1| cationic peroxidase	8E-26
Contig954	418	373	2	0	2	gb|AC138452.10|	Medicago truncatula clone mth2-17p11, complete sequence	0.11
Contig955	694	670	7	0	7	gb|AAA33358.1|	3-hydroxy-3-methylglutaryl-coenzyme A reductase	2E-30
Contig956	426	405	3	0	3	gb|AC174326.12|	Medicago truncatula chromosome 8 clone mth2-107i14, complete sequence	1E-38
Contig957	757	690	13	7	6	sp|P28012|CFI1_MEDSA	Chalcone--flavonone isomerase 1 (Chalcone isomerase 1) pdb|1EYP|A Chain A, Chalcone Isomerase pdb|1EYP|B Chain B, Chalcone Isomerase pdb|1EYQ|A Chain A, Chalcone Isomerase And Naringenin pdb|1EYQ|B Chain B, Chalcone Isomerase And Naringenin pdb|1JEP|A Chain A, Chalcone Isomerase Complexed With 4'-Hydroxyflavanone pdb|1JEP|B Chain B, Chalcone Isomerase Complexed With 4'-Hydroxyflavanone pdb|1FM8|A Chain A, Chalcone Isomerase Complexed With 5,4'-Dideoxyflavanone pdb|1FM8|B Chain B, Chalcone Isomerase Complexed With 5,4'-Dideoxyflavanone pdb|1FM7|A Chain A, Chalcone Isomerase Complexed With 5-Deoxyflavanone pdb|1FM7|B Chain B, Chalcone Isomerase Complexed With 5-Deoxyflavanone gb|AAB41524.1| chalcone isomerase [Medicago sativa]	1E-103
Contig958	421	394	2	0	2	gb|AAX18706.1|	cold-related protein Cor413 [Gossypium barbadense] gb|ABI97481.1| COR413-like protein [Gossypium barbadense]	7E-13
Contig959	424	403	2	0	2	emb|CAC08564.1|	wound-induced GSK-3-like protein [Medicago sativa subsp. x varia]	5E-25
Contig960	426	405	2	0	2	emb|CAN73778.1|	hypothetical protein [Vitis vinifera]	2E-47
Contig961	426	375	3	0	3	gb|AAD32880.1|AC005489_18	F14N23.18 [Arabidopsis thaliana]	3E-17
Contig962	321	273	26	1	25	dbj|BAB33421.1|	putative senescence-associated protein [Pisum sativum]	4E-19

Contig963	421	393	2	0	2	gb|EAZ38613.1|	hypothetical protein OsJ_022096 [Oryza sativa (japonica cultivar-group)]	1E-21
Contig964	422	404	2	0	2	emb|CAN63456.1|	hypothetical protein [Vitis vinifera]	2E-63
Contig965	754	733	4	0	4	gb|ABF66654.1|	EBP1 [Ammopiptanthus mongolicus]	1E-100
Contig966	367	343	2	0	2	ref|NP_001078516.1|	histone H3.2 [Arabidopsis thaliana]	9E-53
Contig967	177	159	2	0	2	gb|AAU07997.1|	phosphoenolpyruvate carboxylase 2; LaPEPC2 [Lupinus albus]	1E-21
Contig968	349	326	4	1	3	sp|Q41649|FKB15_VICFA	FK506-binding protein 2 precursor (Peptidyl-prolyl cis-trans isomerase) (PPIase) (Rotamase) (15 kDa FKBP) (FKBP-15) gb|AAC49392.1| immunophilin precursor	2E-36
Contig969	274	253	2	1	1	gb|ABE87707.1|	hypothetical protein MtrDRAFT_AC155898g5v1 [Medicago truncatula] gb|ABO83845.1| mitochondrial ATP synthase precursor, putative [Medicago truncatula]	1E-35
Contig970	428	407	2	1	1	gb|ABD32383.1|	Zinc finger, RING-type [Medicago truncatula]	6E-25
Contig971	301	262	2	0	2	gb|AAT74554.1|	QM family protein [Caragana jubata]	1E-44
Contig972	410	364	7	0	7	gb|ABO82310.1|	Dimerisation [Medicago truncatula]	3E-41
Contig973	494	452	6	0	6	emb|CAA52800.1|	T-protein of the glycine decarboxylase complex [Pisum sativum]	3E-80

a. These ESTs come from JG-62 genotype.

b. These ESTs come from WR-315 genotpe.

c. Accession number shown refers to the NCBI database.

d. The possible function of the chickpea contigs was assigned by performing BLASTX and BLASTN against nonredundant protein and nucleotide database in NCBI.

e. For BLASTX the E-value cutoff used was 10 and for BLASTN the cutoff used wad 10-20.
