# Supplementary material for: Comparative analyses of genotype dependent expressed sequence tags and stress-responsive transcriptome of chickpea wilt illustrate predicted and unexpected genes and novel regulators of plant immunity
Source: BMC Genomics. 2009 Sep 5;10:415. doi: 10.1186/1471-2164-10-415 (PMC2755012; doi:10.1186/1471-2164-10-415)
Supplement: Additional file 2 — Annotation of CaEST singletons. Table showing Summary of CaEST singletons including their length and functional annotation based on BLASTX and BLASTN. [file 1471-2164-10-415-S2.pdf]

Additional file 2 - Summary of *Ca* EST singletons including their length and functional annotation based on BLASTX and BLASTN

Clone Id[a]	Length (bp)	Accession number[b]	Functional annotation[c]	E-value[d]
CaF1_JIE_01_A_03	312	emb|AM497806.1|	Nidula niveotomentosa partial mRNA for putative betatubulin (btt gene)	0.00002
CaF1_JIE_01_C_01	240	gb|ABD32214.1|	Ribosomal protein L34e [Medicago truncatula] gb|ABN08919.1| Ribosomal protein L34e [Medicago truncatula]	6E-29
CaF1_JIE_01_C_09	376	gb|ABE87035.1|	Orn/DAP/Arg decarboxylase 2; Protease-associated PA; Proteinase inhibitor I9, subtilisin propeptide [Medicago truncatula]	8E-57
CaF1_JIE_01_D_02	461	emb|CAC10208.1|	cytosolic malate dehydrogenase [Cicer arietinum]	2E-72
CaF1_JIE_01_D_06	152	gb|EAZ40434.1|	hypothetical protein OsJ_023917 [Oryza sativa (japonica cultivar-group)]	9E-22
CaF1_JIE_01_H_08	200	gb|AY232724.1|	Fusarium oxysporum f. sp. vasinfectum strain Ag149-III Foxy transposable element, partial sequence	2E-87
CaF1_JIE_02_A_02	196	emb|AM706411.1|	Eristalis tenax partial mRNA for hypothetical protein (ORF1), isolate 3	0.0008
CaF1_JIE_02_C_04	214	emb|CAB75818.1|	putative protein [Arabidopsis thaliana]	3E-32
CaF1_JIE_02_G_01	449	gb|ABE91098.1|	Ribosomal protein S4E; RNA-binding S4; KOW [Medicago truncatula]	1E-61
CaF1_JIE_03_A_05	470	gb|ABE88922.1|	Serine/threonine protein kinase, active site [Medicago truncatula]	1E-71
CaF1_JIE_03_A_08	267	emb|CAN77471.1|	hypothetical protein [Vitis vinifera]	2E-22
CaF1_JIE_03_B_09	351	gb|ABE83254.1|	Peptidase S8 and S53, subtilisin, kexin, sedolisin; Integrase, catalytic region; Zinc finger, CCHC-type; Peptidase aspartic, catalytic [Medicago truncatula]	3E-16
CaF1_JIE_03_B_10	318	gb|ABE88922.1|	Serine/threonine protein kinase, active site [Medicago truncatula]	1E-47
CaF1_JIE_03_C_03	240	gb|ABD33394.2|	FAR1; Polynucleotidyl transferase, Ribonuclease H fold [Medicago truncatula]	3E-29
CaF1_JIE_03_C_04	336	gb|EDN17888.1|	eukaryotic initiation factor 4A [Botryotinia fuckeliana B05.10]	1E-11
CaF1_JIE_03_C_09	383	emb|CAN66051.1|	hypothetical protein [Vitis vinifera]	6E-28
CaF1_JIE_03_E_09	491	emb|CAA62226.1|	peroxidase1B [Medicago sativa]	1E-68
CaF1_JIE_03_G_01	463	gb|ABE84189.1|	SYNC1 protein, related [Medicago truncatula]	3E-75
CaF1_JIE_03_G_05	458	gb|AAW78864.1|	respiratory burst oxidase 2 [Medicago truncatula] gb|ABN08032.1| Calcium-binding EF-hand; Ferric reductase-like transmembrane component [Medicago truncatula]	8E-79
CaF1_JIE_03_G_09	486	emb|CAN84007.1|	hypothetical protein [Vitis vinifera]	4E-13
CaF1_JIE_03_H_11	457	ref|XP_001223273.1|	mannitol-1-phosphate dehydrogenase [Chaetomium globosum CBS 148.51] gb|EAQ87440.1| mannitol-1-phosphate dehydrogenase [Chaetomium globosum CBS 148.51]	1E-17
CaF1_JIE_04_A_03	311	ref|XP_388769.1|	hypothetical protein FG08593.1 [Gibberella zeae PH-1]	4E-25
CaF1_JIE_04_B_09	392	ref|NP_566858.1|	unknown protein [Arabidopsis thaliana] dbj|BAB02262.1| unnamed protein product [Arabidopsis thaliana] gb|AAK92723.1| unknown protein [Arabidopsis thaliana] gb|AAM45104.1| unknown protein [Arabidopsis thaliana]	1E-25
CaF1_JIE_04_C_01	460	ref|XP_381616.1|	hypothetical protein FG01440.1 [Gibberella zeae PH-1]	9E-29
CaF1_JIE_04_D_05	389	gb|ABD32692.1|	2OG-Fe(II) oxygenase [Medicago truncatula]	2E-35
CaF1_JIE_04_H_03	456	gb|AAL86291.1|	unknown protein [Arabidopsis thaliana]	5E-22
CaF1_JIE_04_H_04	167	ref|XP_381334.1|	hypothetical protein FG01158.1 [Gibberella zeae PH-1]	6E-12
CaF1_JIE_04_H_05	462	sp|P81406|GAPN_PEA	NADP-dependent glyceraldehyde-3-phosphate dehydrogenase (Non-phosphorylating glyceraldehyde 3-phosphate dehydrogenase) (Glyceraldehyde-3-phosphate dehydrogenase [NADP+]) (Triosephosphate dehydrogenase) emb|CAA53076.1| glyceraldehyde-3-phosphate dehydrogenase (nonphosphorylating,NADP+) [Pisum sativum] gb|AAO38512.1| non-phosphorylating glyceraldehyde-3-phosphate dehydrogenase [Pisum sativum]	5E-77
CaF1_JIE_05_B_01	217	gb|AAB05992.1|	SDL	2E-25
CaF1_JIE_05_B_05	379	ref|NP_189124.1|	PRT1 (PROTEOLYSIS 1); ubiquitin-protein ligase [Arabidopsis thaliana] sp|Q8LBL5|PRT1_ARATH E3 ubiquitin-protein ligase PRT1 (Proteolysis 1 protein) emb|CAA11891.1| PRT1 [Arabidopsis thaliana] emb|CAA11892.1| PRT1 [Arabidopsis thaliana] dbj|BAB02890.1| PRT1 protein [Arabidopsis thaliana] gb|AAL87280.1| putative PRT1 protein [Arabidopsis thaliana] gb|AAM45125.1| putative PRT1 protein [Arabidopsis thaliana]	3E-21
CaF1_JIE_05_B_08	460	gb|EAZ41147.1|	hypothetical protein OsJ_024630 [Oryza sativa (japonica cultivar-group)]	9E-75
CaF1_JIE_05_D_06	426	gb|DQ459385.1|	Nicotiana tabacum serine/threonine kinase mRNA, partial cds	1E-13
CaF1_JIE_05_E_01	428	gb|ABE80212.1|	Protein of unknown function DUF339 [Medicago truncatula]	7E-32
CaF1_JIE_05_E_02	424	emb|AJ538370.1|NTA538370	Nicotiana tabacum cDNA-AFLP-fragment BT1-M24-018	0.000000002
CaF1_JIE_05_E_05	260	gb|EAZ03897.1|	hypothetical protein OsI_025129 [Oryza sativa (indica cultivar-group)]	3E-25
CaF1_JIE_05_E_06	434	dbj|BAD90801.1|	histone 3 [Conocephalum conicum]	5E-27

CaF1_JIE_05_F_10	244	emb|X56240.1|VFUSP	V.faba USP gene for an unknown seed protein	3E-16
CaF1_JIE_05_F_11	400	gb|ABE79940.1|	Aldo/keto reductase [Medicago truncatula]	1E-51
CaF1_JIE_05_G_09	414	gb|DQ465789.1|	Sesbania drummondii clone SSH-36_01_A09_T3 mRNA sequence	0.0005
CaF1_JIE_05_H_07	235	gb|AY232720.1|	Fusarium oxysporum f. sp. vasinfectum strain X515-II Foxy transposable element, partial sequence	3E-99
CaF1_JIE_06_A_10	221	gb|AC123882.6|	Mus musculus chromosome 1, clone RP23-447L15, complete sequence	0.88
CaF1_JIE_06_C_04	427	dbj|BAE71261.1|	hypothetical protein [Trifolium pratense]	2E-52
CaF1_JIE_06_C_10	463	ref|NP_001050135.1|	Os03g0355600 [Oryza sativa (japonica cultivar-group)] dbj|BAF12049.1| Os03g0355600 [Oryza sativa (japonica cultivar-group)]	2E-77
CaF1_JIE_06_D_02	461	gb|ABO81176.1|	Protein of unknown function DUF26 [Medicago truncatula]	4E-62
CaF1_JIE_06_D_08	302	gb|AC152184.1|	Medicago truncatula chromosome 7 clone mte1-61c3, complete sequence	0.00000009
CaF1_JIE_06_E_05	428	emb|CAG28693.1|	hypothetical protein [Gibberella fujikuroi]	1E-19
CaF1_JIE_06_E_10	239	gb|AY232720.1|	Fusarium oxysporum f. sp. vasinfectum strain X515-II Foxy transposable element, partial sequence	1E-107
CaF1_JIE_07_B_04	431	emb|CAN77652.1|	hypothetical protein [Vitis vinifera]	3E-67
CaF1_JIE_07_B_10	389	ref|XP_390378.1|	hypothetical protein FG10202.1 [Gibberella zeae PH-1]	9E-19
CaF1_JIE_07_C_06	166	gb|AC186678.3|	Medicago truncatula chromosome 7 BAC clone mth2-51a12, complete sequence	2E-44
CaF1_JIE_07_C_07	460	emb|CAN78114.1|	hypothetical protein [Vitis vinifera]	2E-22
CaF1_JIE_07_D_04	161	gb|AY461597.1|	Synthetic construct arsenic-like protein gene, complete cds	0.00001
CaF1_JIE_07_D_11	389	emb|AL592227.4|	Human DNA sequence from clone RP11-32D4 on chromosome 9 Contains a CpG island, complete sequence	0.41
CaF1_JIE_07_E_03	423	gb|ABN08957.1|	H+-transporting two-sector ATPase, C (AC39) subunit [Medicago truncatula]	2E-72
CaF1_JIE_07_E_05	437	gb|ABE80517.1|	Autophagy-related protein 2, related [Medicago truncatula]	2E-33
CaF1_JIE_07_F_03	430	gb|AC146549.8|	Medicago truncatula clone mth2-7p18, complete sequence	5E-16
CaF1_JIE_07_F_10	194	gb|AC140550.31|	Medicago truncatula clone mth2-54a24, complete sequence	6E-23
CaF1_JIE_07_G_02	272	gb|ABD28486.1|	Protein of unknown function UPF0041 [Medicago truncatula]	2E-30
CaF1_JIE_07_G_06	462	sp|P22196|PER2_ARAHY	Cationic peroxidase 2 precursor (PNPC2) gb|AAA32676.1| cationic peroxidase	1E-22
CaF1_JIE_07_G_11	368	gb|AAG28426.1|AF194945_1	cytosolic aconitase [Nicotiana tabacum]	6E-52
CaF1_JIE_07_H_03	180	gb|ABE82281.1|	Ribosomal protein S9 [Medicago truncatula] gb|ABO82751.1| Ribosomal protein S9 [Medicago truncatula]	3E-14
CaF1_JIE_07_H_04	365	gb|ABN09189.1|	cAMP response element binding (CREB) protein [Medicago truncatula]	4E-22
CaF1_JIE_07_H_11	467	gb|ABO79438.1|	Peptidase aspartic, active site [Medicago truncatula]	2E-40
CaF1_JIE_08_A_08	200	emb|AM422099.2|	Danio rerio tf2a mRNA, 3'UTR	0.0002
CaF1_JIE_08_D_09	483	emb|CAN65776.1|	hypothetical protein [Vitis vinifera]	7E-61
CaF1_JIE_08_D_11	456	ref|XP_383193.1|	hypothetical protein FG03017.1 [Gibberella zeae PH-1]	6E-41
CaF1_JIE_08_E_06	306	gb|AAV92900.1|	Avr9/Cf-9 rapidly elicited protein 150 [Nicotiana tabacum]	2E-11
CaF1_JIE_08_F_02	463	gb|ABA54868.1|	putative shikimate kinase [Fagus sylvatica]	6E-31
CaF1_JIE_08_F_08	461	gb|ABE93079.1|	hypothetical protein MtrDRAFT_AC144517g10v2 [Medicago truncatula]	2E-63
CaF1_JIE_08_G_07	458	ref|NP_566028.1|	unknown protein [Arabidopsis thaliana] gb|AAK48959.1|AF370532_1 Unknown protein [Arabidopsis thaliana] gb|AAL66926.1| unknown protein [Arabidopsis thaliana] gb|AAC31837.2| expressed protein [Arabidopsis thaliana]	2E-11
CaF1_JIE_08_G_10	290	dbj|BAD24657.1|	xylogen protein 1 [Zinnia elegans]	2E-17
CaF1_JIE_09_A_03	261	gb|AC125476.30|	Medicago truncatula clone mth2-10e13, complete sequence	8E-20
CaF1_JIE_09_C_10	455	gb|AAN03470.1|	RING-H2 finger protein [Glycine max]	5E-51
CaF1_JIE_09_D_03	152	gb|ABE87516.2|	Ribosomal protein L7Ae/L30e/S12e/Gadd45 [Medicago truncatula]	2E-11
CaF1_JIE_09_D_06	353	ref|XP_381184.1|	hypothetical protein FG01008.1 [Gibberella zeae PH-1]	2E-44
CaF1_JIE_09_E_02	459	gb|ABD33160.1|	Protein of unknown function DUF716 [Medicago truncatula]	6E-36
CaF1_JIE_09_E_03	425	gb|AAD52015.1|AF082862_1	unknown [Pisum sativum]	2E-51
CaF1_JIE_09_E_06	266	ref|XM_711830.1|	Candida albicans SC5314 hypothetical protein (CaO19_12635), mRNA	0.017
CaF1_JIE_09_F_08	328	emb|CAN78632.1|	hypothetical protein [Vitis vinifera]	5E-41
CaF1_JIE_09_G_04	458	gb|EAY90304.1|	hypothetical protein OsI_011537 [Oryza sativa (indica cultivar-group)]	3E-33
CaF1_JIE_09_G_05	458	emb|CAN80281.1|	hypothetical protein [Vitis vinifera]	3E-46
CaF1_JIE_09_H_01	310	gb|AAX86048.1|	tubulin B4 [Glycine max]	2E-36

CaF1_JIE_09_H_09	448	ref|NP_194420.1|	AT-HF (Arabidopsis thaliana HisF protein) sp|Q9SZ30|HIS5_ARATH Imidazole glycerol phosphate synthase hisHF, chloroplast precursor (IGP synthase) (ImGP synthase) (IGPS) [Includes: Glutamine amidotransferase ; Cyclase] emb|CAB36536.1| glutamine amidotransferase/cyclase [Arabidopsis thaliana] emb|CAB79545.1| glutamine amidotransferase/cyclase [Arabidopsis thaliana]	2E-49
CaF1_JIE_10_B_07	458	gb|ABE85391.1|	S25 ribosomal protein [Medicago truncatula] gb|ABE85967.1| S25 ribosomal protein [Medicago truncatula]	8E-31
CaF1_JIE_10_C_01	272	emb|AM706411.1|	Eristalis tenax partial mRNA for hypothetical protein (ORF1), isolate 3	0.00007
CaF1_JIE_10_D_09	220	emb|CAN59899.1|	hypothetical protein [Vitis vinifera]	2E-16
CaF1_JIE_10_F_02	422	emb|CAN82378.1|	hypothetical protein [Vitis vinifera]	1E-51
CaF1_JIE_10_G_06	370	gb|AE013599.4|	Drosophila melanogaster chromosome 2R, complete sequence	1.5
CaF1_JIE_10_H_07	338	gb|EAZ43296.1|	hypothetical protein OsJ_026779 [Oryza sativa (japonica cultivar-group)]	7E-14
CaF1_JIE_11_E_07	458	gb|ABE88774.1|	Translation factor; Elongation factor G, III and V [Medicago truncatula]	2E-57
CaF1_JIE_11_E_08	240	gb|DQ459385.1|	Nicotiana tabacum serine/threonine kinase mRNA, partial cds	0.004
CaF1_JIE_12_C_01	189	emb|AM706411.1|	Eristalis tenax partial mRNA for hypothetical protein (ORF1), isolate 3	0.00000001
CaF1_JIE_12_D_06	454	gb|ABE79750.1|	AAA ATPase, central region; DEAD/DEAH box helicase, N-terminal [Medicago truncatula]	3E-45
CaF1_JIE_12_E_05	375	dbj|BAB09853.1|	ER66 protein-like [Arabidopsis thaliana]	9E-35
CaF1_JIE_12_E_08	375	dbj|BAC07504.2|	receptor-like protein kinase [Nicotiana tabacum]	2E-50
CaF1_JIE_12_E_10	286	ref|XP_001219290.1|	hypothetical protein CHGG_00069 [Chaetomium globosum CBS 148.51] gb|EAQ91834.1| hypothetical protein CHGG_00069 [Chaetomium globosum CBS 148.51]	3E-19
CaF1_JIE_12_E_11	456	emb|CAN80884.1|	hypothetical protein [Vitis vinifera]	3E-42
CaF1_JIE_12_H_08	450	dbj|BAD94487.1|	phosphoenolpyruvate carboxykinase-like protein [Arabidopsis thaliana]	4E-39
CaF1_JIE_13_C_01	274	gb|ABD32291.1|	Uncharacterized Cys-rich domain [Medicago truncatula]	1E-27
CaF1_JIE_13_C_11	429	ref|XP_001224160.1|	hypothetical protein CHGG_04946 [Chaetomium globosum CBS 148.51] gb|EAQ88327.1| hypothetical protein CHGG_04946 [Chaetomium globosum CBS 148.51]	4E-17
CaF1_JIE_13_E_03	379	ref|NP_194214.2|	arginosuccinate synthase family [Arabidopsis thaliana] sp|Q9SZX3|ASSY_ARATH Argininosuccinate synthase, chloroplast precursor (Citrulline--aspartate ligase) gb|AAL38728.1| putative argininosuccinate synthase [Arabidopsis thaliana] gb|AAM14258.1| putative argininosuccinate synthase [Arabidopsis thaliana]	1E-55
CaF1_JIE_14_A_04	463	gb|AAT68475.1|	calcium/calmodulin-regulated receptor-like kinase [Medicago sativa]	3E-12
CaF1_JIE_14_B_09	423	gb|AAL74418.2|AF452454_1	ATP sulfurylase [Glycine max]	2E-74
CaF1_JIE_14_C_09	427	gb|ABO84327.1|	Putative non-LTR retroelement reverse transcriptase, related [Medicago truncatula]	4E-19
CaF1_JIE_14_D_03	463	ref|NP_197749.2|	DNAJ heat shock N-terminal domain-containing protein [Arabidopsis thaliana] ref|NP_001031930.1| heat shock protein binding / nucleotide binding / unfolded protein binding [Arabidopsis thaliana] gb|AAM91576.1| putative protein [Arabidopsis thaliana] gb|AAP13437.1| At5g23590 [Arabidopsis thaliana]	1E-40
CaF1_JIE_14_H_09	462	ref|NP_200706.1|	ceramidase family protein [Arabidopsis thaliana]	1E-47
CaF1_JIE_15_A_03	384	gb|ABO83982.1|	protein binding , related [Medicago truncatula]	4E-59
CaF1_JIE_15_A_06	221	gb|EAY88020.1|	hypothetical protein OsI_009253 [Oryza sativa (indica cultivar-group)]	3E-23
CaF1_JIE_15_A_09	461	gb|EAY96947.1|	hypothetical protein OsI_018180 [Oryza sativa (indica cultivar-group)]	3E-26
CaF1_JIE_15_A_10	461	ref|XP_389647.1|	GR78_NEUCR 78 KDA GLUCOSE-REGULATED PROTEIN HOMOLOG PRECURSOR (GRP 78) (IMMUNOGLOBULIN HEAVY CHAIN BINDING PROTEIN HOMOLOG) (BIP) [Gibberella zeae PH-1]	2E-40
CaF1_JIE_15_B_02	460	gb|ABE81529.1|	Pyridoxal-5-phosphate-dependent enzyme, beta subunit [Medicago truncatula] gb|ABE91493.1| Pyridoxal-5-phosphate-dependent enzyme, beta subunit [Medicago truncatula]	3E-74
CaF1_JIE_15_B_11	379	gb|ABE92129.1|	Protein kinase [Medicago truncatula]	8E-57
CaF1_JIE_15_C_05	183	gb|AY327035.1|	Ixodes ricinus cytochrome oxidase subunit I mRNA, partial cds; mitochondrial gene for mitochondrial product	0.0002
CaF1_JIE_15_D_07	467	gb|AAU05467.1|	At5g22850 [Arabidopsis thaliana] gb|AAV59285.1| At5g22850 [Arabidopsis thaliana]	1E-61
CaF1_JIE_15_E_02	237	gb|BT009458.1|	Triticum aestivum clone wlsu2.pk0001.h3:fis, full insert mRNA sequence	0.0002
CaF1_JIE_15_G_09	463	gb|AAA66200.1|	signal recognition particle 54 kDa subunit	6E-28

CaF1_JIE_15_H_01	351	gb|ABE78289.1|	UDP-glucuronosyl/UDP-glucosyltransferase [Medicago truncatula]	1E-48
CaF1_JIE_16_B_05	193	sp|P12886|ADH1_PEA	Alcohol dehydrogenase 1 emb|CAA29609.1| alcohol dehydrogenase [Pisum sativum]	2E-22
CaF1_JIE_16_C_06	259	emb|AM697674.1|	Platynereis dumerilii mRNA for hypothetical protein (ORF1), isolate 2	0.001
CaF1_JIE_16_G_09	415	ref|XP_380571.1|	RS15_PODAN 40S RIBOSOMAL PROTEIN S15 (S12) [Gibberella zeae PH-1]	8E-41
CaF1_JIE_16_H_01	440	emb|CAN76661.1|	hypothetical protein [Vitis vinifera]	5E-43
CaF1_JIE_16_H_02	467	dbj|BAD94495.1|	sigma-like factor [Arabidopsis thaliana]	8E-12
CaF1_JIE_16_H_10	450	gb|AC166093.12|	Medicago truncatula clone mth2-17k6, complete sequence	2E-40
CaF1_JIE_17_B_10	280	ref|XP_381443.1|	hypothetical protein FG01267.1 [Gibberella zeae PH-1]	7E-34
CaF1_JIE_17_C_11	151	gb|AC146573.21|	Medicago truncatula clone mth2-145j1, complete sequence	5E-23
CaF1_JIE_17_E_07	120	emb|AJ749800.1|	Photobacterium damselae subsp. piscicida partial coi genes for putative cytochrome C oxidase proteins, clone pRDA19	0.0000005
CaF1_JIE_17_F_04	494	gb|ABD28734.1|	UDP-N-acetylglucosamine transferase subunit ALG14, related [Medicago truncatula] gb|ABE92926.1| UDP-N-acetylglucosamine transferase subunit ALG14, related [Medicago truncatula]	1E-13
CaF1_JIE_17_G_04	174	dbj|BAF44219.1|	polyketide reductase [Lotus japonicus]	9E-11
CaF1_JIE_18_B_01	360	gb|EAZ41723.1|	hypothetical protein OsJ_025206 [Oryza sativa (japonica cultivar-group)]	3E-16
CaF1_JIE_18_C_07	393	ref|XP_460488.1|	hypothetical protein DEHA0F03157g [Debaryomyces hansenii CBS767] emb|CAG88798.1| unnamed protein product [Debaryomyces hansenii CBS767]	1E-52
CaF1_JIE_18_C_10	339	ref|NP_172147.2|	2-oxoglutarate-dependent dioxygenase, putative [Arabidopsis thaliana] sp|Q84MB3|ACCH1_ARATH 1-aminocyclopropane-1-carboxylate oxidase homolog 1 gb|AAP21238.1| At1g06620 [Arabidopsis thaliana] dbj|BAE99663.1| oxidoreductase like protein [Arabidopsis thaliana]	2E-21
CaF1_JIE_18_D_08	452	gb|AAV66464.1|	drought responsive element binding protein [Glycine soja]	6E-36
CaF1_JIE_18_E_07	219	emb|AJ749800.1|	Photobacterium damselae subsp. piscicida partial coi genes for putative cytochrome C oxidase proteins, clone pRDA19	0.00000006
CaF1_JIE_18_F_02	456	sp|O04866|ARGD_ALNGL	Acetylornithine aminotransferase, mitochondrial precursor (ACOAT) (Acetylornithine transaminase) (AOTA) emb|CAA69936.1| acetylornithine aminotransferase [Alnus glutinosa]	2E-44
CaF1_JIE_18_H_08	450	ref|XP_388653.1|	hypothetical protein FG08477.1 [Gibberella zeae PH-1]	1E-47
CaF1_JIE_19_A_01	351	gb|ABG21940.1|	AGR_C_5039p, putative, expressed [Oryza sativa (japonica cultivar-group)] gb|ABG21941.1| AGR_C_5039p, putative, expressed [Oryza sativa (japonica cultivar-group)]	4E-31
CaF1_JIE_19_A_02	461	gb|AAQ87023.1|	VDAC3.1 [Lotus corniculatus var. japonicus]	7E-27
CaF1_JIE_19_A_09	446	emb|AM706411.1|	Eristalis tenax partial mRNA for hypothetical protein (ORF1), isolate 3	0.00000003
CaF1_JIE_19_C_02	450	ref|XP_387328.1|	hypothetical protein FG07152.1 [Gibberella zeae PH-1]	2E-28
CaF1_JIE_19_E_08	135	ref|XM_381728.1|	Gibberella zeae PH-1 chromosome 1 hypothetical protein (FG01552.1) partial mRNA	2E-12
CaF1_JIE_19_F_04	152	gb|AC151956.5|	Medicago truncatula clone mth2-52p17, complete sequence	9E-34
CaF1_JIE_19_F_10	461	gb|ABO81298.1|	Protein kinase; TonB box, N-terminal [Medicago truncatula]	3E-44
CaF1_JIE_19_F_11	460	sp|P51850|PDC1_PEA	Pyruvate decarboxylase isozyme 1 (PDC) emb|CAA91444.1| pyruvate decarboxylase [Pisum sativum]	1E-69
CaF1_JIE_19_G_03	447	emb|CAN72598.1|	hypothetical protein [Vitis vinifera]	1E-68
CaF1_JIE_19_G_04	456	gb|ABE79642.1|	Cellular retinaldehyde-binding/triple function, N-terminal [Medicago truncatula]	2E-62
CaF1_JIE_19_G_10	368	emb|AM706411.1|	Eristalis tenax partial mRNA for hypothetical protein (ORF1), isolate 3	3E-17
CaF1_JIE_19_H_01	460	emb|CAN68819.1|	hypothetical protein [Vitis vinifera]	3E-48
CaF1_JIE_20_A_01	462	gb|AF082024.1|AF082024	Pimpinella brachycarpa Phyb1 mRNA, complete cds	0.000000009
CaF1_JIE_20_A_02	296	emb|CAA10189.1|	class I chitinase [Cicer arietinum]	3E-21
CaF1_JIE_20_A_07	398	emb|AJ749797.1|	Photobacterium damselae subsp. piscicida trpB gene for putative transposase, clone pRDA16	0.0004
CaF1_JIE_20_A_11	490	ref|NP_195483.1|	HHP4 (heptahelical protein 4); receptor [Arabidopsis thaliana] gb|AAK25883.1|AF360173_1 unknown protein [Arabidopsis thaliana] emb|CAB38307.1| putative protein [Arabidopsis thaliana] emb|CAB80433.1| putative protein [Arabidopsis thaliana] gb|AAL07197.1| unknown protein [Arabidopsis thaliana]	1E-63
CaF1_JIE_20_B_11	175	gb|AC148406.12|	Medicago truncatula clone mth2-46e9, complete sequence	8E-10
CaF1_JIE_20_C_03	453	gb|EDN04544.1|	actin [Ajellomyces capsulatus NAm1]	4E-76
CaF1_JIE_20_C_08	460	emb|CAJ13711.1|	putative ethylene response protein [Capsicum chinense]	9E-56
CaF1_JIE_20_D_03	407	gb|ABE82132.1|	Protein of unknown function DUF239, plant [Medicago truncatula]	2E-64
CaF1_JIE_20_D_05	154	gb|AC147430.9|	Medicago truncatula clone mth2-71e6, complete sequence	1E-20
CaF1_JIE_20_D_06	461	dbj|BAE99337.1|	putative beta-amylase [Arabidopsis thaliana]	4E-60
CaF1_JIE_20_D_10	462	emb|CAN72566.1|	hypothetical protein [Vitis vinifera]	2E-40
CaF1_JIE_20_G_10	275	ref|XP_384352.1|	hypothetical protein FG04176.1 [Gibberella zeae PH-1]	2E-26

CaF1_JIE_21_B_05	462	gb|AAK92832.1|	putative glycyl tRNA synthetase [Arabidopsis thaliana]	2E-29
CaF1_JIE_22_A_04	375	gb|EAT90924.1|	hypothetical protein SNOG_01275 [Phaeosphaeria nodorum SN15]	9E-33
CaF1_JIE_22_A_06	460	gb|AAF70823.1|AF154422_1	beta-galactosidase [Lycopersicon esculentum]	3E-34
CaF1_JIE_22_A_09	340	emb|CAK54360.1|	putative desaturase-like protein [Trifolium repens]	2E-51
CaF1_JIE_22_B_02	463	emb|CAN61038.1|	hypothetical protein [Vitis vinifera]	1E-17
CaF1_JIE_22_B_08	459	gb|DQ251457.1|	Siniperca chuatsi transposase mRNA, partial cds	0.00003
CaF1_JIE_22_B_11	459	ref|NP_196799.1|	unknown protein [Arabidopsis thaliana] emb|CAB88259.1| putative protein [Arabidopsis thaliana] dbj|BAC42665.1| unknown protein [Arabidopsis thaliana]	8E-60
CaF1_JIE_22_D_10	458	emb|AM706411.1|	Eristalis tenax partial mRNA for hypothetical protein (ORF1), isolate 3	0.0001
CaF1_JIE_22_D_11	381	ref|XM_381680.1|	Gibberella zeae PH-1 chromosome 1 conserved hypothetical protein (FG01504.1) partial mRNA	5E-13
CaF1_JIE_22_E_06	461	gb|AAT40482.1|	hypothetical protein [Solanum demissum]	2E-45
CaF1_JIE_22_E_07	462	gb|AAK82520.1|	AT5g24810/F6A4_20 [Arabidopsis thaliana]	6E-60
CaF1_JIE_22_G_08	461	gb|ABE86660.1|	Zinc finger, C2H2-type [Medicago truncatula]	3E-14
CaF1_JIE_23_A_03	462	sp|Q850K7|EXLB1_ORYSJ	Expansin-like B1 precursor (OsEXLB1) (Expensin-related 1) (OsEXPR1) (OsaEXPb3.1) gb|EAZ03949.1| hypothetical protein OsI_025181 [Oryza sativa (indica cultivar-group)] gb|EAZ39893.1| hypothetical protein OsJ_023376 [Oryza sativa (japonica cultivar-group)]	5E-27
CaF1_JIE_23_C_09	432	emb|CAB41490.1|	cytochrome P450 monooxygenase [Cicer arietinum]	6E-71
CaF1_JIE_23_F_05	461	emb|AM748481.1|	Vigna unguiculata partial mRNA for putative ATP synthase CF1 alpha subunit (atpA gene), clone 26	0.002
CaF1_JIE_23_H_02	460	emb|CAN71304.1|	hypothetical protein [Vitis vinifera]	1E-46
CaF1_JIE_24_A_06	437	emb|AM422119.2|	Danio rerio ca7 mRNA, 3' UTR	0.0005
CaF1_JIE_24_D_02	450	gb|ABE93515.2|	Cytochrome b5 [Medicago truncatula]	2E-28
CaF1_JIE_24_E_01	224	gb|AY972077.1|	Synthetic construct RLS (RLS) gene, complete cds	0.004
CaF1_JIE_24_F_04	319	emb|AJ243804.1|CAR243804	Cicer arietinum mRNA for cytochrome P450 (cyp93C3 gene)	9E-75
CaF1_JIE_24_F_11	240	ref|NP_179680.1|	secretory carrier membrane protein (SCAMP) family protein [Arabidopsis thaliana] gb|AAD20911.1| putative secretory carrier-associated membrane protein [Arabidopsis thaliana] gb|AAT06461.1| At2g20840 [Arabidopsis thaliana] dbj|BAD93720.1| putative secretory carrier-associated membrane protein [Arabidopsis thaliana]	1E-32
CaF1_JIE_25_A_02	431	gb|AAB84193.1|	dormancy-associated protein [Pisum sativum]	9E-38
CaF1_JIE_25_A_06	223	gb|ABE79228.1|	2OG-Fe(II) oxygenase [Medicago truncatula]	4E-27
CaF1_JIE_25_B_07	303	gb|AAP03880.2|	Avr9/Cf-9 induced kinase 1 [Nicotiana tabacum]	9E-45
CaF1_JIE_25_C_01	384	gb|ABE94142.2|	Translation initiation factor IF5 [Medicago truncatula]	4E-38
CaF1_JIE_25_D_02	166	emb|AM706411.1|	Eristalis tenax partial mRNA for hypothetical protein (ORF1), isolate 3	0.00000001
CaF1_JIE_25_E_09	400	emb|CAN68798.1|	hypothetical protein [Vitis vinifera]	7E-43
CaF1_JIE_25_E_11	428	emb|CAN80955.1|	hypothetical protein [Vitis vinifera]	5E-20
CaF1_JIE_25_F_03	290	gb|DQ459385.1|	Nicotiana tabacum serine/threonine kinase mRNA, partial cds	0.000001
CaF1_JIE_25_G_08	463	emb|AJ749797.1|	Photobacterium damselae subsp. piscicida trpB gene for putative transposase, clone pRDA16	0.0005
CaF1_JIE_25_G_10	283	emb|CT029282.1|	Poplar cDNA sequences	0.019
CaF1_JIE_25_H_03	224	emb|CU424495.1|	Medicago truncatula chromosome 5 clone mte1-17l21, COMPLETE SEQUENCE	1E-18
CaF1_JIE_25_H_11	229	ref|XP_383636.1|	hypothetical protein FG03460.1 [Gibberella zeae PH-1]	7E-29
CaF1_JIE_26_B_04	126	emb|AJ250814.1|FOX250814	Fusarium oxysporum f. sp. lycopersici insertion sequence Foxy	0.0000001
CaF1_JIE_26_C_07	243	gb|ABE91874.1|	SAM (and some other nucleotide) binding motif [Medicago truncatula]	6E-31
CaF1_JIE_26_E_04	232	emb|AJ411814.1|CAR411814	Cicer arietinum Ty3-gypsy like Retrotransposon CaRep and partial pol gene for polyprotein including RNAse and Integrase, PPT and 3'LTR, clone pCaEr915	1E-58
CaF1_JIE_26_F_03	199	gb|AF271892.1|AF271892	Pisum sativum DEAD box protein P68 (P68) mRNA, complete cds	2E-20
CaF1_JIE_26_G_07	429	dbj|BAF49052.1|	phytoene synthase [Prunus mume]	1E-69
CaF1_JIE_26_G_09	462	sp|Q41009|TOC34_PEA	Translocase of chloroplast 34 (34 kDa chloroplast outer envelope protein) (GTP-binding protein OEP34) (GTP-binding protein IAP34) emb|CAA82196.1| chloroplast outer envelope protein 34 [Pisum sativum] gb|AAC25785.1| GTP-binding protein [Pisum sativum]	1E-54
CaF1_JIE_27_B_02	406	gb|AAN31890.1|	putative sterol-C-methyltransferase [Arabidopsis thaliana]	3E-58
CaF1_JIE_27_C_04	404	gb|AAC32610.1|	ras-like small monomeric GTP-binding protein [Avena fatua]	9E-51
CaF1_JIE_27_D_01	461	emb|CT028832.1|	Poplar cDNA sequences	0.0004
CaF1_JIE_27_D_10	193	ref|NP_563662.1|	pectinesterase family protein [Arabidopsis thaliana] gb|AAF02886.1|AC009525_20 Similar to pectinesterases [Arabidopsis thaliana] gb|ABO38784.1| At1g02810 [Arabidopsis thaliana]	2E-19
CaF1_JIE_27_E_08	314	emb|CAJ29291.1|	putative polyol transporter protein 4 [Lotus japonicus]	2E-32

CaF1_JIE_28_A_04	444	gb|EAZ17668.1|	hypothetical protein OsJ_031877 [Oryza sativa (japonica cultivar-group)]	1E-34
CaF1_JIE_28_B_05	269	gb|ABD28403.1|	Formylmethionine deformylase [Medicago truncatula]	3E-37
CaF1_JIE_28_D_10	193	ref|NP_563662.1|	pectinesterase family protein [Arabidopsis thaliana] gb|AAF02886.1|AC009525_20 Similar to pectinesterases [Arabidopsis thaliana] gb|ABO38784.1| At1g02810 [Arabidopsis thaliana]	8E-18
CaF1_JIE_28_F_10	342	gb|AAF75791.1|AF271892_1	DEAD box protein P68 [Pisum sativum]	3E-29
CaF1_JIE_29_A_01	170	gb|U10046.1|PSU10046	Pisum sativum ribosomal protein L27 homolog (RPL27-5) mRNA, complete cds	5E-11
CaF1_JIE_29_A_06	416	emb|CAN81370.1|	hypothetical protein [Vitis vinifera]	8E-20
CaF1_JIE_29_A_08	150	gb|AY887902.1|	Homo sapiens mutant GSTP1 (GSTP1) mRNA, complete cds	0.00002
CaF1_JIE_29_D_09	253	emb|AJ749803.1|	Photobacterium damselae subsp. piscicida partial ORF1 DNA for hypothetical protein, clone pRDA24	0.000004
CaF1_JIE_29_E_03	254	gb|AAG26305.1|	photosystem II CP47 protein [Trochodendron aralioides]	7E-35
CaF1_JIE_29_E_08	159	emb|AL445196.7|	Human DNA sequence from clone RP11-31K13 on chromosome 6 Contains a heterogeneous nuclear ribonucleoprotein A1 (HNRPA1) pseudogene, the 5' end of the VMP gene for vesicular membrane protein p24 and one CpG island, complete sequence	0.006
CaF1_JIE_29_E_10	335	ref|XP_390958.1|	hypothetical protein FG10782.1 [Gibberella zeae PH-1]	6E-23
CaF1_JIE_29_F_07	157	gb|ABO78866.1|	WD40-like [Medicago truncatula]	1E-12
CaF1_JIE_29_F_09	272	gb|AC157490.18|	Medicago truncatula clone mth2-123f23, complete sequence	2E-60
CaF1_JIE_29_F_11	253	gb|EAZ25322.1|	hypothetical protein OsJ_008805 [Oryza sativa (japonica cultivar-group)]	8E-17
CaF1_JIE_29_G_02	329	gb|AF537102.1|	Plasmodiophora brassicae 16S ribosomal RNA gene, partial sequence; mitochondrial gene for mitochondrial product	0.00002
CaF1_JIE_29_G_07	392	gb|ABN09164.1|	Protein phosphatase 2C-like [Medicago truncatula]	5E-36
CaF1_JIE_29_H_02	368	gb|ABD28395.1|	Nucleoporin interacting component; Protein prenyltransferase [Medicago truncatula]	5E-54
CaF1_JIE_29_H_07	344	gb|EAY72790.1|	hypothetical protein OsI_000637 [Oryza sativa (indica cultivar-group)]	7E-34
CaF1_JIE_30_A_02	231	gb|EAZ35224.1|	hypothetical protein OsJ_018707 [Oryza sativa (japonica cultivar-group)]	3E-12
CaF1_JIE_30_B_06	382	emb|CAN79350.1|	hypothetical protein [Vitis vinifera]	5E-22
CaF1_JIE_30_D_06	231	gb|ABE77505.1|	DECOY (exp=-1; , putative [Medicago truncatula]	4E-18
CaF1_JIE_30_E_06	461	emb|AM748481.1|	Vigna unguiculata partial mRNA for putative ATP synthase CF1 alpha subunit (atpA gene), clone 26	0.002
CaF1_JIE_30_F_04	331	dbj|AB286673.1|	Lethenteron japonicum LjHox10s gene for LjHox10s homeobox, parital cds	0.0004
CaF1_JIE_30_H_05	101	emb|AM497808.1|	Nidula niveotomentosa partial mRNA for putative thiolase c (thio c gene)	0.00009
CaF1_JIE_31_A_04	396	gb|AAT36331.1|	nitrilase 4A [Lupinus angustifolius] gb|ABB51979.1| nitrilase 4A [Lupinus angustifolius]	1E-54
CaF1_JIE_31_D_02	296	ref|XP_381197.1|	conserved hypothetical protein [Gibberella zeae PH-1] sp|Q8L805|RL35_WHEAT 60S ribosomal protein L35 pdb|2GO5|5 Chain 5, Structure Of Signal Recognition Particle Receptor (Sr) In Complex With Signal Recognition Particle (Srp) And Ribosome Nascent Chain Complex pdb|2J37|5 Chain 5, Model Of Mammalian Srp Bound To 80s Rncs gb|AAM92709.1| putative ribosomal protein L35 [Triticum aestivum]	1E-36
CaF1_JIE_31_F_03	455	gb|AC167403.2|	Medicago truncatula chromosome 7 BAC clone mte1-14f12, complete sequence	3E-27
CaF1_JIE_31_F_11	223	gb|AAM49801.1|	GFA2 [Arabidopsis thaliana]	1E-18
CaF1_JIE_32_A_06	410	emb|CAN68264.1|	hypothetical protein [Vitis vinifera]	7E-61
CaF1_JIE_32_B_05	194	gb|AC137822.30|	Medicago truncatula clone mth2-31e20, complete sequence	3E-22
CaF1_JIE_32_E_06	249	ref|XP_380676.1|	conserved hypothetical protein [Gibberella zeae PH-1]	3E-30
CaF1_JIE_32_E_09	176	gb|ABE92104.1|	hypothetical protein MtrDRAFT_AC144760g11v2 [Medicago truncatula]	2E-18
CaF1_JIE_32_E_10	340	emb|AJ293848.1|KPN293848	Klebsiella pneumoniae contig region pSL022	0.00009
CaF1_JIE_32_G_03	223	emb|CAA06731.1|	GDP dissociation inhibitor [Cicer arietinum]	2E-31
CaF1_JIE_32_G_08	122	gb|AY232722.1|	Fusarium oxysporum f. sp. vasinfectum strain Ag149 Foxy transposable element, partial sequence	7E-40
CaF1_JIE_32_G_09	406	emb|AJ749800.1|	Photobacterium damselae subsp. piscicida partial coi genes for putative cytochrome C oxidase proteins, clone pRDA19	0.0001
CaF1_JIE_32_H_02	107	gb|AC148397.13|	Medicago truncatula clone mth2-22h4, complete sequence	3E-26
CaF1_JIE_33_A_07	472	gb|AAF78397.1|AC009273_3	Contains similarity to a putative protein T2J13.100 gi|6522560 from Arabidopsis thaliana BAC T2J13 gb|AL132967	3E-24
CaF1_JIE_33_C_09	458	gb|ABO84551.1|	Protein of unknown function DUF506, plant [Medicago truncatula]	4E-48

CaF1_JIE_33_D_01	462	gb|ABE93018.1|	cAMP response element binding (CREB) protein; Prefoldin [Medicago truncatula]	2E-34
CaF1_JIE_33_D_08	448	emb|CAN78553.1|	hypothetical protein [Vitis vinifera]	3E-66
CaF1_JIE_34_B_03	312	emb|AJ749800.1|	Photobacterium damselae subsp. piscicida partial coi genes for putative cytochrome C oxidase proteins, clone pRDA19	0.0000003
CaF1_JIE_34_B_06	263	gb|AC151621.20|	Medicago truncatula clone mth2-14p3, complete sequence	1E-24
CaF1_JIE_34_B_07	304	gb|K03313.1|RIATL	Integrated Ri plasmid agropine (A. rhizogenes strain A4) complete TL-DNA and flanking plant (Convolvulus arvensis) DNA	1E-157
CaF1_JIE_34_B_11	326	emb|CAN80974.1|	hypothetical protein [Vitis vinifera]	8E-44
CaF1_JIE_34_D_01	448	gb|ABN08040.1|	Acyl-coA-binding protein, ACBP; Serine/threonine protein phosphatase, BSU1 [Medicago truncatula]	2E-41
CaF1_JIE_34_E_01	458	ref|XP_384354.1|	hypothetical protein FG04178.1 [Gibberella zeae PH-1]	3E-52
CaF1_JIE_34_G_01	232	gb|DQ251457.1|	Siniperca chuatsi transposase mRNA, partial cds	0.00002
CaF1_JIE_34_H_11	350	gb|ABE77486.1|	Protein kinase [Medicago truncatula]	2E-22
CaF1_JIE_35_B_03	307	gb|ABL59986.1|	brittle stalk-2-like protein 6 [Zea mays]	6E-15
CaF1_JIE_35_C_04	350	ref|NM_145102.2|	Homo sapiens zinc finger with KRAB and SCAN domains 5 (ZKSCAN5), transcript variant 2, mRNA	5.7
CaF1_JIE_35_D_08	438	gb|ABG22120.1|	polyprotein [Cynara scolymus]	4E-42
CaF1_JIE_35_E_02	322	gb|ABN08649.1|	C2 [Medicago truncatula]	9E-32
CaF1_JIE_35_F_10	168	gb|AC175685.3|	Medicago truncatula chromosome 2 BAC clone mte1-55k6, complete sequence	6E-26
CaF1_JIE_35_H_02	209	emb|AM706411.1|	Eristalis tenax partial mRNA for hypothetical protein (ORF1), isolate 3	0.00000001
CaF1_JIE_35_H_10	346	ref|XP_361955.2|	hypothetical protein MGG_04400 [Magnaporthe grisea 70-15] gb|EDJ96104.1| hypothetical protein MGG_04400 [Magnaporthe grisea 70-15]	4E-40
CaF1_JIE_36_A_06	294	emb|AJ749794.1|	Photobacterium damselae subsp. piscicida trpA gene for putative transposase and partial ORF1 DNA for hypothetical protein, clone pRDA13	0.00002
CaF1_JIE_36_B_01	463	sp|P06452|ATPI_PEA	Chloroplast ATP synthase a chain precursor (ATPase subunit IV) emb|CAA29349.1| atpI protein [Pisum sativum] emb|CAA27255.1| unnamed protein product [Pisum sativum] prf||1204179A synthase a,ATP	2E-79
CaF1_JIE_36_B_02	463	gb|AAW33880.1|	RING-H2 subgroup RHE protein [Populus alba x Populus tremula]	8E-15
CaF1_JIE_36_B_11	357	ref|NP_194186.1|	clathrin adaptor complexes medium subunit family protein [Arabidopsis thaliana] emb|CAA23008.1| clathrin coat assembly like protein [Arabidopsis thaliana] emb|CAB79365.1| clathrin coat assembly like protein [Arabidopsis thaliana] gb|AAL59993.1| putative clathrin coat assembly protein [Arabidopsis thaliana]	3E-39
CaF1_JIE_36_D_11	439	emb|CAN64650.1|	hypothetical protein [Vitis vinifera]	6E-31
CaF1_JIE_36_E_08	304	dbj|BAB86895.1|	syringolide-induced protein B15-3-5 [Glycine max]	2E-38
CaF1_JIE_36_F_10	432	ref|NP_181934.1|	late embryogenesis abundant family protein / LEA family protein [Arabidopsis thaliana] ref|NP_850408.1| late embryogenesis abundant family protein / LEA family protein [Arabidopsis thaliana] gb|AAC23428.1| similar to late embryogenesis abundant proteins [Arabidopsis thaliana] gb|ABD59061.1| At2g44060 [Arabidopsis thaliana]	6E-50
CaF1_JIE_36_H_10	397	gb|ABN05714.1|	metal ion transporter , putative [Medicago truncatula] gb|ABE84684.2| metal ion transporter , putative [Medicago truncatula]	2E-21
CaF1_JIE_36_H_11	448	gb|ABE83633.1|	Ribosomal protein L10; Ribosomal protein 60S [Medicago truncatula]	2E-58
CaF1_JIE_37_A_07	214	gb|AC146746.15|	Medicago truncatula clone mth2-108p9, complete sequence	9E-38
CaF1_JIE_37_B_02	373	gb|AAX63898.1|	geranylgeranyl reductase [Medicago truncatula]	2E-63
CaF1_JIE_37_C_10	370	gb|ABN08360.1|	Cation transporting ATPase, C-terminal [Medicago truncatula]	3E-51
CaF1_JIE_37_E_05	200	gb|AC147009.5|	Medicago truncatula clone mth2-139j3, complete sequence	6E-17
CaF1_JIE_37_F_08	344	emb|CAA10131.1|	chalcone synthase [Cicer arietinum]	1E-48
CaF1_JIE_37_G_05	165	ref|XM_381579.1|	Gibberella zeae PH-1 chromosome 1 hypothetical protein (FG01403.1) partial mRNA	1E-11
CaF1_JIE_37_H_06	251	gb|AF537102.1|	Plasmodiophora brassicae 16S ribosomal RNA gene, partial sequence; mitochondrial gene for mitochondrial product	0.00002
CaF1_JIE_38_A_05	208	ref|XP_389789.1|	conserved hypothetical protein [Gibberella zeae PH-1]	3E-28
CaF1_JIE_38_B_08	315	dbj|BAD95892.1|	Ser/Thr protein kinase [Lotus japonicus]	5E-45
CaF1_JIE_38_C_11	423	gb|AAY86360.1|	cinnamoyl-CoA reductase [Acacia mangium x Acacia auriculiformis]	3E-43
CaF1_JIE_38_E_01	151	gb|AC104660.4|	Homo sapiens BAC clone RP11-614H7 from 4, complete sequence	0.57
CaF1_JIE_38_E_11	421	gb|AF075691.1|	Crassostrea gigas BAT1 homolog mRNA, complete cds	0.0000001
CaF1_JIE_38_F_06	186	gb|AAK13318.1|AF290958_1	ATP:citrate lyase [Capsicum annuum]	7E-14
CaF1_JIE_38_H_02	444	ref|XP_388777.1|	hypothetical protein FG08601.1 [Gibberella zeae PH-1]	2E-43
CaF1_JIE_39_C_03	454	gb|ABE77854.2|	Phospholipid/glycerol acyltransferase [Medicago truncatula]	2E-40
CaF1_JIE_39_G_10	205	emb|AL929433.10|	Mouse DNA sequence from clone RP23-193M23 on chromosome 4, complete sequence	0.21

CaF1_JIE_39_H_08	377	emb|CAA10289.1|	hypothetical protein [Cicer arietinum]	5E-51
CaF1_JIE_40_B_07	398	sp|P28551|TBB3_SOYBN	Tubulin beta chain (Beta tubulin) emb|CAA42777.1| beta-tubulin [Glycine max]	6E-63
CaF1_JIE_40_B_11	463	emb|CAN73178.1|	hypothetical protein [Vitis vinifera]	1E-19
CaF1_JIE_40_C_08	368	emb|AM706411.1|	Eristalis tenax partial mRNA for hypothetical protein (ORF1), isolate 3	1E-13
CaF1_JIE_40_D_01	188	gb|AY461597.1|	Synthetic construct arsenic-like protein gene, complete cds	0.003
CaF1_JIE_40_D_03	392	emb|CAA10289.1|	hypothetical protein [Cicer arietinum]	2E-37
CaF1_JIE_40_E_01	220	gb|AAM19795.1|	At2g04030/F3C11.14 [Arabidopsis thaliana]	5E-28
CaF1_JIE_40_E_05	462	dbj|BAD97435.1|	peroxidase [Pisum sativum]	1E-58
CaF1_JIE_40_F_07	250	emb|CAN82032.1|	hypothetical protein [Vitis vinifera]	1E-25
CaF1_JIE_40_F_10	298	gb|ABE85501.1|	Protein of unknown function DUF810 [Medicago truncatula]	1E-29
CaF1_JIE_40_G_04	296	gb|AC186194.28|	Medicago truncatula chromosome 6 clone mth2-77b24, complete sequence	0.000001
CaF1_JIE_40_H_02	139	emb|AM706411.1|	Eristalis tenax partial mRNA for hypothetical protein (ORF1), isolate 3	0.00003
CaF1_JIE_40_H_08	372	gb|ABE88390.1|	Pre-mRNA processing ribonucleoprotein, binding region; NOSIC [Medicago truncatula]	3E-56
CaF1_JIE_41_A_04	447	emb|CAN74802.1|	hypothetical protein [Vitis vinifera]	4E-60
CaF1_JIE_41_A_06	447	ref|XP_965630.1|	hypothetical protein [Neurospora crassa OR74A] gb|EAA36394.1| hypothetical protein [Neurospora crassa]	3E-11
CaF1_JIE_41_A_10	288	emb|AJ749800.1|	Photobacterium damselae subsp. piscicida partial coi genes for putative cytochrome C oxidase proteins, clone pRDA19	0.0000003
CaF1_JIE_41_B_07	191	sp|P12886|ADH1_PEA	Alcohol dehydrogenase 1 emb|CAA29609.1| alcohol dehydrogenase [Pisum sativum]	2E-22
CaF1_JIE_41_C_07	307	gb|AF537102.1|	Plasmodiophora brassicae 16S ribosomal RNA gene, partial sequence; mitochondrial gene for mitochondrial product	0.00002
CaF1_JIE_41_D_01	376	ref|XP_391654.1|	hypothetical protein FG11478.1 [Gibberella zeae PH-1]	8E-20
CaF1_JIE_41_D_04	459	emb|CAA87075.1|	heat shock transcription factor 29 [Glycine max]	7E-24
CaF1_JIE_41_E_01	447	gb|ABE89691.1|	Pectinesterase [Medicago truncatula]	8E-33
CaF1_JIE_41_E_08	411	emb|AM706411.1|	Eristalis tenax partial mRNA for hypothetical protein (ORF1), isolate 3	0.002
CaF1_JIE_41_G_02	458	gb|ABP03273.1|	Uncharacterized Cys-rich domain [Medicago truncatula]	1E-69
CaF1_JIE_41_G_04	461	pir||S47243	starch phosphorylase (EC 2.4.1.1) isoform L precursor, chloroplast - fava bean	2E-80
CaF1_JIE_41_H_04	222	ref|XP_383642.1|	hypothetical protein FG03466.1 [Gibberella zeae PH-1]	5E-26
CaF1_JIE_42_A_01	374	ref|NP_181224.1|	xyloglucan:xyloglucosyl transferase, putative / xyloglucan endotransglycosylase, putative / endo-xyloglucan transferase, putative [Arabidopsis thaliana] sp|Q9SJL9|XTH32_ARATH Probable xyloglucan endotransglucosylase/hydrolase protein 32 precursor (At-XTH32) (XTH-32) gb|AAD31572.1| xyloglucan endotransglycosylase, putative [Arabidopsis thaliana] gb|AAK76514.1| putative xyloglucan endo-transglycosylase [Arabidopsis thaliana] gb|AAM66089.1| putative xyloglucan endo-transglycosylase [Arabidopsis thaliana] gb|AAM91780.1| putative xyloglucan endo-transglycosylase [Arabidopsis thaliana]	6E-49
CaF1_JIE_42_A_04	410	sp|P08688|ALB2_PEA	Albumin-2 (PA2) gb|AAA02981.1| albumin 2 gb|AAA33641.1| major seed albumin prf||1314296A albumin	2E-56
CaF1_JIE_42_G_01	316	gb|AAT38758.1|	Putative gag-pol polyprotein, identical [Solanum demissum]	9E-22
CaF1_JIE_42_G_06	282	sp|Q00016|IFR_CICAR	Isoflavone reductase (IFR) (2'-hydroxyisoflavone reductase) (NADPH:isoflavone oxidoreductase) emb|CAA43167.1| NADPH:isoflavone oxidoreductase [Cicer arietinum]	1E-45
CaF1_WIE_01_A_01	240	emb|CAI56440.1|	S-adenosyl-L-homocysteine hydrolase [Cicer arietinum]	8E-34
CaF1_WIE_01_A_05	304	emb|AL672270.12|	Mouse DNA sequence from clone RP23-351A10 on chromosome X Contains the Rbm41 gene for RNA binding motif protein 41, the gene for a novel Nsp1-like C-terminal region containing protein, a novel gene (E230019M04Rik), a similar to glyceraldehyde-3-phosphate	0.005
CaF1_WIE_01_A_11	460	sp|P52780|SYQ_LUPLU	Glutaminyl-tRNA synthetase (Glutamine--tRNA ligase) (GlnRS) emb|CAA62901.1| tRNA-glutamine synthetase [Lupinus luteus]	5E-68
CaF1_WIE_01_B_04	174	emb|AJ404640.1|CAR404640	Cicer arietinum mRNA for for hypothetical protein, clone Can47	2E-69
CaF1_WIE_01_B_06	336	gb|AY972077.1|	Synthetic construct RLS (RLS) gene, complete cds	0.0004
CaF1_WIE_01_B_07	167	gb|DQ459385.1|	Nicotiana tabacum serine/threonine kinase mRNA, partial cds	0.0000002
CaF1_WIE_01_C_02	229	emb|CAA19877.1|	protein kinase-like protein [Arabidopsis thaliana] emb|CAB80112.1| protein kinase-like protein [Arabidopsis thaliana]	1E-28
CaF1_WIE_01_C_05	426	gb|AC150843.16|	Medicago truncatula clone mth2-103p8, complete sequence	1E-23
CaF1_WIE_01_C_07	290	gb|AC020551.5|	Homo sapiens BAC clone RP11-404J23 from 4, complete sequence	0.3
CaF1_WIE_01_C_08	331	emb|CAA10189.1|	class I chitinase [Cicer arietinum]	6E-57
CaF1_WIE_01_D_01	301	gb|AC126786.22|	Medicago truncatula clone mth2-8c2, complete sequence	1E-43
CaF1_WIE_01_D_04	357	emb|AM431760.2|	Vitis vinifera contig VV78X085461.2, whole genome shotgun sequence	0.095
CaF1_WIE_01_D_09	360	gb|ABO82002.1|	AIG1 [Medicago truncatula]	3E-29
CaF1_WIE_01_E_03	461	gb|AAY43802.1|	Fb2 [Gossypium hirsutum]	5E-39

CaF1_WIE_01_G_06	450	ref|NP_563771.1|	GAUT6 (Galacturonosyltransferase 6); polygalacturonate 4-alpha-galacturonosyltransferase/ transferase, transferring glycosyl groups [Arabidopsis thaliana] gb|AAF63140.1|AC011001_10 Unknown protein [Arabidopsis thaliana] gb|AAK76574.1| unknown protein [Arabidopsis thaliana] gb|AAM14391.1| unknown protein [Arabidopsis thaliana]	6E-49
CaF1_WIE_01_H_08	240	emb|CT028787.1|	Poplar cDNA sequences	0.000001
CaF1_WIE_01_H_09	458	ref|NP_564118.1|	dolichyl-phosphate beta-D-mannosyltransferase, putative / dolichol-phosphate mannosyltransferase, putative / mannose-P-dolichol synthase, putative [Arabidopsis thaliana] gb|AAF80640.1|AC069251_33 F2D10.6 [Arabidopsis thaliana] gb|AAO64810.1| At1g20575 [Arabidopsis thaliana] dbj|BAD43322.1| hypothetical protein [Arabidopsis thaliana] dbj|BAE99453.1| hypothetical protein [Arabidopsis thaliana]	7E-56
CaF1_WIE_02_A_06	208	emb|AM461517.2|	Vitis vinifera contig VV78X154143.3, whole genome shotgun sequence	0.013
CaF1_WIE_02_B_04	400	emb|CAN62584.1|	hypothetical protein [Vitis vinifera]	5E-32
CaF1_WIE_02_B_09	457	ref|NP_567210.1|	unknown protein [Arabidopsis thaliana] sp|O04616|Y4115_ARATH Protein At4g01150, chloroplast precursor gb|AAK63864.1|AF389292_1 AT4g01150/F2N1_18 [Arabidopsis thaliana] gb|AAB61025.1| A_IG002N01.18 gene product [Arabidopsis thaliana] emb|CAB80924.1| hypothetical protein [Arabidopsis thaliana] gb|AAM10278.1| AT4g01150/F2N1_18 [Arabidopsis thaliana]	2E-25
CaF1_WIE_02_C_03	461	emb|CAN63456.1|	hypothetical protein [Vitis vinifera]	9E-51
CaF1_WIE_02_C_06	465	dbj|BAD91082.1|	beta-D-galactosidase [Pyrus pyrifolia]	6E-49
CaF1_WIE_02_D_09	440	sp|P48488|PP1_MEDVA	Serine/threonine-protein phosphatase PP1 emb|CAA56766.1| potentially catalitic subunit of the ser /thr protein phosphatase 1 [Medicago sativa subsp. x varia]	2E-23
CaF1_WIE_02_E_05	463	emb|CAN65557.1|	hypothetical protein [Vitis vinifera]	8E-15
CaF1_WIE_02_E_09	344	sp|P48488|PP1_MEDVA	Serine/threonine-protein phosphatase PP1 emb|CAA56766.1| potentially catalitic subunit of the ser /thr protein phosphatase 1 [Medicago sativa subsp. x varia]	6E-57
CaF1_WIE_02_G_07	453	gb|ABD96879.1|	hypothetical protein [Cleome spinosa]	2E-40
CaF1_WIE_02_H_03	325	emb|X95708.1|CANMT1MET	C.arietinum mRNA for metallothionein (clone: CanMT-1)	2E-33
CaF1_WIE_02_H_08	352	ref|NP_175380.2|	peroxidase, putative [Arabidopsis thaliana] sp|Q9FX85|PER10_ARATH Peroxidase 10 precursor (Atperox P10) (ATP5a) gb|AAG13043.1|AC011807_2 peroxidase ATP5a [Arabidopsis thaliana] dbj|BAC43700.1| putative peroxidase [Arabidopsis thaliana] gb|AAP12891.1| At1g49570 [Arabidopsis thaliana]	2E-36
CaF1_WIE_02_H_11	316	emb|AJ534351.1|ABI534351	Agaricus bisporus partial mRNA for putative myosin heavy chain kinase (mhck gene), clone pm31	0.001
CaF1_WIE_03_A_06	240	gb|AAV85853.1|	AT-rich element binding factor 3 [Pisum sativum]	2E-34
CaF1_WIE_03_A_11	424	dbj|BAF01953.1|	hypothetical protein [Arabidopsis thaliana]	2E-64
CaF1_WIE_03_B_07	339	gb|AAP68983.1|	alternative oxidase 2b [Glycine max]	6E-31
CaF1_WIE_03_C_03	426	sp|P31023|DLDH_PEA	Dihydrolipoyl dehydrogenase, mitochondrial precursor (Dihydrolipoamide dehydrogenase) (Pyruvate dehydrogenase complex E3 subunit) (PDC-E3) (E3) (Glycine cleavage system L protein) emb|CAA44729.1| lipoamide dehydrogenase [Pisum sativum] emb|CAA45066.2| dihydrolipoamide dehydrogenase [Pisum sativum]	3E-69
CaF1_WIE_03_D_05	344	emb|CAN73725.1|	hypothetical protein [Vitis vinifera]	1E-38
CaF1_WIE_03_E_02	431	gb|ABE93328.1|	Peptidase S10, serine carboxypeptidase [Medicago truncatula]	6E-36
CaF1_WIE_03_F_01	459	gb|ABN09112.1|	Haem peroxidase, plant/fungal/bacterial [Medicago truncatula]	1E-37
CaF1_WIE_03_F_02	284	gb|DQ459385.1|	Nicotiana tabacum serine/threonine kinase mRNA, partial cds	0.000000005
CaF1_WIE_03_F_11	423	dbj|BAB43814.1|	CaNAG3 [Candida albicans] dbj|BAB43819.1| CaNAG3 [Candida albicans]	3E-14
CaF1_WIE_03_H_01	337	emb|CT971488.3|	M.truncatula DNA sequence from clone MTH2-154I11 on chromosome 3, complete sequence	6E-74
CaF1_WIE_03_H_03	353	sp|P31023|DLDH_PEA	Dihydrolipoyl dehydrogenase, mitochondrial precursor (Dihydrolipoamide dehydrogenase) (Pyruvate dehydrogenase complex E3 subunit) (PDC-E3) (E3) (Glycine cleavage system L protein) emb|CAA44729.1| lipoamide dehydrogenase [Pisum sativum] emb|CAA45066.2| dihydrolipoamide dehydrogenase [Pisum sativum]	8E-50
CaF1_WIE_03_H_07	156	gb|DQ306770.1|	Hevea brasiliensis isolate SSH41 mRNA sequence	0.002
CaF1_WIE_03_H_08	143	emb|AJ250814.1|FOX250814	Fusarium oxysporum f. sp. lycopersici insertion sequence Foxy	9E-40
CaF1_WIE_03_H_10	430	emb|CAA10287.2|	glucan-endo-1,3-beta-glucosidase [Cicer arietinum]	5E-67
CaF1_WIE_03_H_11	461	emb|CAN78123.1|	hypothetical protein [Vitis vinifera]	4E-30
CaF1_WIE_04_A_09	453	emb|CAA08855.1|	copper amine oxidase [Cicer arietinum]	4E-74

CaF1_WIE_04_B_03	459	ref|NP_179333.2|	ARID/BRIGHT DNA-binding domain-containing protein [Arabidopsis thaliana] gb|AAK96550.1| At2g17400 [Arabidopsis thaliana] gb|AAO11549.1| At2g17400/At2g17400 [Arabidopsis thaliana] dbj|BAF02083.1| hypothetical protein [Arabidopsis thaliana]	2E-44
CaF1_WIE_04_C_07	460	emb|AJ749800.1|	Photobacterium damselae subsp. piscicida partial coi genes for putative cytochrome C oxidase proteins, clone pRDA19	1E-16
CaF1_WIE_04_C_09	328	sp|P29450|TRXF_PEA	Thioredoxin F-type, chloroplast precursor (TRX-F) emb|CAA45098.1| thioredoxin F [Pisum sativum] gb|AAC49357.1| thioredoxin f	2E-42
CaF1_WIE_04_D_11	334	emb|CAN68264.1|	hypothetical protein [Vitis vinifera]	5E-43
CaF1_WIE_04_E_07	448	ref|NP_199258.3|	unknown protein [Arabidopsis thaliana]	1E-51
CaF1_WIE_04_E_08	446	gb|AAW31666.1|	putative late-embryogenesis protein-like protein [Ammopiptanthus mongolicus]	4E-29
CaF1_WIE_04_F_04	382	gb|AC140026.11|	Medicago truncatula clone mth2-36j24, complete sequence	7E-12
CaF1_WIE_04_F_06	180	gb|AC124216.27|	Medicago truncatula clone mth2-34o22, complete sequence	0.003
CaF1_WIE_04_G_10	304	gb|AC174142.11|	Medicago truncatula clone mth2-69j4, complete sequence	8E-42
CaF1_WIE_05_A_10	327	gb|AAK62821.1|AF332960_1	auxin-regulated dual specificity cytosolic kinase [Lycopersicon esculentum]	3E-29
CaF1_WIE_05_A_11	245	gb|ABD96861.1|	hypothetical protein [Cleome spinosa]	5E-24
CaF1_WIE_05_B_06	425	gb|ABN08784.1|	Hypothetical 214.8 kDa protein ycf1, related [Medicago truncatula]	6E-55
CaF1_WIE_05_B_07	464	gb|AAM63442.1|	PSI type III chlorophyll a/b-binding protein, putative [Arabidopsis thaliana]	1E-23
CaF1_WIE_05_B_10	264	emb|AJ749800.1|	Photobacterium damselae subsp. piscicida partial coi genes for putative cytochrome C oxidase proteins, clone pRDA19	0.00007
CaF1_WIE_05_C_05	270	emb|AJ006024.1|CAR6024	Cicer arietinum mRNA for cysteine synthase, partial	1E-104
CaF1_WIE_05_C_07	360	emb|CAN75603.1|	hypothetical protein [Vitis vinifera]	2E-20
CaF1_WIE_05_D_08	462	gb|AAF33784.1|AF220456_1	cold acclimation responsive protein BudCAR4 [Medicago sativa] gb|AAF33786.1|AF220458_1 cold acclimation responsive protein BudCAR6 [Medicago sativa] gb|AAA16926.1| CAS15	2E-18
CaF1_WIE_05_E_03	247	emb|CAB88266.1|	putative protein [Arabidopsis thaliana]	5E-28
CaF1_WIE_05_E_04	344	gb|AC174142.11|	Medicago truncatula clone mth2-69j4, complete sequence	8E-61
CaF1_WIE_05_E_05	181	gb|ABE82023.1|	Protein kinase [Medicago truncatula]	6E-20
CaF1_WIE_05_E_09	277	emb|CAB56743.1|	cytochrome P450 monooxygenase [Cicer arietinum]	2E-11
CaF1_WIE_05_F_01	453	gb|AAO24648.1|	unknown protein [Phytophthora sojae]	1E-43
CaF1_WIE_05_F_04	452	gb|ABE77517.1|	HSF/ETS, DNA-binding [Medicago truncatula]	7E-59
CaF1_WIE_05_G_03	386	ref|XP_453838.1|	unnamed protein product [Kluyveromyces lactis] ref|XP_453844.1| unnamed protein product [Kluyveromyces lactis] emb|CAH00934.1| unnamed protein product [Kluyveromyces lactis NRRL Y-1140] emb|CAH00940.1| unnamed protein product [Kluyveromyces lactis NRRL Y-1140]	4E-12
CaF1_WIE_05_G_05	216	gb|EAY96947.1|	hypothetical protein OsI_018180 [Oryza sativa (indica cultivar-group)]	3E-21
CaF1_WIE_05_G_07	151	emb|AJ749797.1|	Photobacterium damselae subsp. piscicida trpB gene for putative transposase, clone pRDA16	0.00001
CaF1_WIE_05_H_02	192	gb|AAM63510.1|	Actin-depolymerizing factor ADF-6 [Arabidopsis thaliana]	2E-12
CaF1_WIE_05_H_05	356	emb|CAN60304.1|	hypothetical protein [Vitis vinifera]	4E-11
CaF1_WIE_06_A_01	313	gb|ABO83962.1|	Glycoside hydrolase, family 1 [Medicago truncatula]	2E-38
CaF1_WIE_06_A_08	450	emb|AJ299396.1|CAR299396	Cicer arietinum partial mRNA for putative extensin (ORF), clone CanEXT-1	7E-34
CaF1_WIE_06_B_01	428	gb|DQ465791.1|	Sesbania drummondii clone SSH-38_01_F05_T3 mRNA sequence	0.007
CaF1_WIE_06_C_04	302	gb|ABE77841.1|	PEBP [Medicago truncatula]	8E-12
CaF1_WIE_06_F_11	457	dbj|BAE71282.1|	putative receptor-like GPI-anchored protein 2 [Trifolium pratense]	4E-44
CaF1_WIE_06_H_07	146	gb|AAU04405.1|	pyruvate kinase [Citrus limon]	9E-17
CaF1_WIE_06_H_09	382	ref|XM_001068112.1|	PREDICTED: Rattus norvegicus hypothetical protein LOC688741 (LOC688741), mRNA	0.0001
CaF1_WIE_07_A_03	222	emb|AM748481.1|	Vigna unguiculata partial mRNA for putative ATP synthase CF1 alpha subunit (atpA gene), clone 26	0.004
CaF1_WIE_07_A_05	244	gb|AC091047.10|	Homo sapiens chromosome 8, clone RP11-102F4, complete sequence	0.004
CaF1_WIE_07_A_06	215	emb|CAN77792.1|	hypothetical protein [Vitis vinifera]	1E-13
CaF1_WIE_07_B_07	464	ref|NP_567972.1|	SLP2 (subtilisin-like serine protease 2); subtilase [Arabidopsis thaliana] emb|CAA17763.1| subtilisin proteinase-like [Arabidopsis thaliana] emb|CAB80215.1| subtilisin proteinase-like [Arabidopsis thaliana] gb|AAL67071.1| putative subtilisin serine protease [Arabidopsis thaliana] gb|AAM19998.1| putative subtilisin serine proteinase [Arabidopsis thaliana]	1E-58
CaF1_WIE_07_D_07	365	gb|ABN09825.1|	Thioredoxin domain 2; Thioredoxin fold [Medicago truncatula]	7E-26
CaF1_WIE_07_E_03	426	sp|P29828|PDI_MEDSA	Protein disulfide-isomerase precursor (PDI) emb|CAA77575.1| protein disulfide isomerase [Medicago sativa]	2E-62

CaF1_WIE_07_E_04	207	emb|AJ250814.1|FOX250814	Fusarium oxysporum f. sp. lycopersici insertion sequence Foxy	3E-31
CaF1_WIE_07_E_10	419	emb|Z31720.1|NTL19RIB	N.tabacum (cv.Samsun NN) L19 mRNA for ribosomal protein L19	0.0000005
CaF1_WIE_07_F_04	179	gb|AC144477.16|	Medicago truncatula clone mth2-6k4, complete sequence	6E-20
CaF1_WIE_07_G_02	416	gb|ABM53472.1|	eIF5A [Rosa chinensis]	5E-43
CaF1_WIE_07_G_04	321	gb|DQ251457.1|	Siniperca chuatsi transposase mRNA, partial cds	0.00002
CaF1_WIE_08_A_11	296	gb|AAP69821.1|	ARF [Oryza sativa (japonica cultivar-group)]	2E-34
CaF1_WIE_08_B_04	370	gb|ABE90187.1|	Splicing factor 3B subunit 10 [Medicago truncatula]	5E-44
CaF1_WIE_08_E_09	423	gb|AC173474.18|	Medicago truncatula clone mth2-64j6, complete sequence	2E-12
CaF1_WIE_08_F_06	409	ref|NP_566383.1|	protein phosphatase-related [Arabidopsis thaliana] gb|AAF01527.1|AC009991_23 unknown protein [Arabidopsis thaliana] gb|ABD19691.1| At3g10940 [Arabidopsis thaliana] dbj|BAE98389.1| hypothetical protein [Arabidopsis thaliana]	8E-60
CaF1_WIE_08_F_10	422	emb|AJ293392.1|HSA293392	Homo sapiens mRNA differentially expressed in malignant melanoma, clone MM K2	0.0005
CaF1_WIE_08_H_06	421	emb|CAN63226.1|	hypothetical protein [Vitis vinifera]	4E-25
CaF1_WIE_08_H_07	100	dbj|AB019573.1|	Homo sapiens mRNA expressed only in placental villi, clone SMAP31	0.0003
CaF1_WIE_08_H_08	424	emb|AJ293850.1|KPN293850	Klebsiella pneumoniae partial EVGA gene for putative positive transcription regulator EVGA, contig region pSL042	0.0001
CaF1_WIE_09_A_05	295	gb|AY147012.1|	Medicago truncatula type IIB calcium ATPase (MCA6) mRNA, partial cds	3E-38
CaF1_WIE_09_C_10	301	gb|L47967.1|PEARPL41A	Pisum sativum (clone PsRCI35-2) ribosomal protein L41 mRNA, complete cds	7E-30
CaF1_WIE_09_C_11	372	gb|ABO81477.1|	GB|AAB61107.1|2194132|F20P5 , putative [Medicago truncatula]	6E-44
CaF1_WIE_09_D_10	111	gb|AY245442.1|	Pisum sativum ent-kaurene oxidase (LH) mRNA, complete cds	3E-14
CaF1_WIE_09_E_10	454	emb|CAI56441.1|	germin-like protein [Cicer arietinum]	5E-63
CaF1_WIE_09_E_11	420	gb|ABE79363.1|	Protein kinase [Medicago truncatula]	2E-47
CaF1_WIE_09_F_01	486	gb|ABE84859.1|	Phosphofructokinase [Medicago truncatula]	2E-45
CaF1_WIE_09_F_03	402	emb|CAB95831.1|	hypothetical protein [Cicer arietinum]	2E-17
CaF1_WIE_09_F_07	120	gb|AC167711.1|	Medicago truncatula chromosome 7 clone mth2-167p21, complete sequence	5E-44
CaF1_WIE_09_F_10	255	emb|AM422099.2|	Danio rerio tf2a mRNA, 3'UTR	0.0003
CaF1_WIE_09_G_04	212	ref|NP_568818.1|	eukaryotic translation initiation factor SUI1, putative [Arabidopsis thaliana] ref|NP_851192.1| eukaryotic translation initiation factor SUI1, putative [Arabidopsis thaliana] gb|AAK60326.1|AF385736_1 AT5g54940/MBG8_21 [Arabidopsis thaliana] dbj|BAB08773.1| translation initiation factor-like protein [Arabidopsis thaliana] gb|AAM64690.1| translation initiation factor-like protein [Arabidopsis thaliana] gb|AAM91507.1| AT5g54940/MBG8_21 [Arabidopsis thaliana]	4E-22
CaF1_WIE_09_H_01	272	gb|ABE84165.2|	5-methyltetrahydropteroyltriglutamate--homocysteine S-methyltransferase; Prismane-like [Medicago truncatula] gb|ABE81639.2| 5-methyltetrahydropteroyltriglutamate--homocysteine S-methyltransferase; Prismane-like [Medicago truncatula]	1E-27
CaF1_WIE_09_H_07	380	gb|ABD96913.1|	hypothetical protein [Cleome spinosa]	4E-37
CaF1_WIE_10_B_01	485	ref|XP_380839.1|	hypothetical protein FG00663.1 [Gibberella zeae PH-1]	1E-35
CaF1_WIE_10_B_02	482	emb|CAA55865.1|	Rab [Medicago sativa]	2E-44
CaF1_WIE_10_B_03	459	gb|ABE85045.1|	Cyclic peptide transporter [Medicago truncatula]	2E-67
CaF1_WIE_10_C_04	454	gb|ABE89041.1|	RNA-binding region RNP-1 (RNA recognition motif) [Medicago truncatula]	2E-33
CaF1_WIE_10_C_07	248	emb|CR954185.3|	Medicago truncatula chromosome 5 clone mth4-20m5, COMPLETE SEQUENCE	4E-80
CaF1_WIE_10_D_09	204	dbj|AK246309.1|	Solanum lycopersicum cDNA, clone: FC06CB10, HTC in fruit	4E-21
CaF1_WIE_10_E_02	488	gb|ABE94678.1|	hypothetical protein MtrDRAFT_AC126784g9v2 [Medicago truncatula]	1E-13
CaF1_WIE_10_F_11	467	gb|AC126784.11|	Medicago truncatula clone mth2-36b12, complete sequence	0.0000005
CaF1_WIE_10_G_11	397	gb|ABO84625.1|	Inositol phosphatase/fructose-1,6-bisphosphatase; Inositol monophosphatase [Medicago truncatula]	1E-16
CaF1_WIE_10_H_06	456	emb|CAN81048.1|	hypothetical protein [Vitis vinifera]	1E-14
CaF1_WIE_10_H_08	407	ref|XP_381235.1|	hypothetical protein FG01059.1 [Gibberella zeae PH-1] sp|Q4INZ9|FKBP4_GIBZE FK506-binding protein 4 (Peptidyl-prolyl cis-trans isomerase) (PPIase) (Rotamase)	3E-25
CaF1_WIE_10_H_10	184	gb|AAB32504.1|	root hair protein RH2 [Pisum sativum]	1E-14
CaF1_WIE_11_B_03	483	gb|ABE89815.1|	hypothetical protein MtrDRAFT_AC148171g28v2 [Medicago truncatula]	2E-47
CaF1_WIE_11_B_09	320	gb|AAC49183.1|	SDL5A	2E-46
CaF1_WIE_11_C_02	307	gb|ABR04092.1|	cytochrome b5 [Malus x domestica]	4E-11
CaF1_WIE_11_C_08	265	gb|AF172172.1|AF172172	Medicago sativa non-symbiotic hemoglobin (MHB1) mRNA, complete cds	2E-27
CaF1_WIE_11_E_06	280	gb|AC146819.19|	Medicago truncatula clone mth2-176k6, complete sequence	5E-12

CaF1_WIE_11_H_10	276	sp|Q9XF97|RL4_PRUAR	60S ribosomal protein L4 (L1) gb|AAD32206.1|AF134732_1 60S ribosomal protein L1 [Prunus armeniaca]	7E-40
CaF1_WIE_12_A_11	390	ref|NP_171699.1|	pentatricopeptide (PPR) repeat-containing protein [Arabidopsis thaliana] gb|AAF76475.1|AC020622_9 Contains similarity to an unknown protein gi|AAD26479 from Arabidopsis thaliana BAC gb|AC007169 and contains multiple PPR PF|01535 repeats gb|AAQ56795.1| At1g01970 [Arabidopsis thaliana] dbj|BAE99830.1| hypothetical protein [Arabidopsis thaliana]	5E-43
CaF1_WIE_12_B_08	476	gb|AAF43953.1|AC012188_30	Strong similarity to an unknown protein from Arabidopsis thaliana gb|AL049171.1	3E-20
CaF1_WIE_12_C_02	194	gb|AY232722.1|	Fusarium oxysporum f. sp. vasinfectum strain Ag149 Foxy transposable element, partial sequence	4E-64
CaF1_WIE_12_C_04	475	emb|CT963109.2|	Medicago truncatula chromosome 5 clone mth2-64i23, COMPLETE SEQUENCE	4E-14
CaF1_WIE_12_C_06	427	ref|NP_565111.1|	nodulin MtN21 family protein [Arabidopsis thaliana] gb|AAK76570.1| putative nodulin protein [Arabidopsis thaliana] gb|AAM14389.1| putative nodulin protein [Arabidopsis thaliana] gb|AAN31815.1| putative nodulin [Arabidopsis thaliana] dbj|BAE98595.1| nodulin-like protein [Arabidopsis thaliana]	3E-46
CaF1_WIE_12_C_07	450	emb|CAN77946.1|	hypothetical protein [Vitis vinifera]	1E-67
CaF1_WIE_12_C_11	282	emb|AJ279846.1|MMU279846	Mus musculus partial mRNA for hypothetical protein, clone mvx2002	0.000005
CaF1_WIE_12_D_04	464	gb|AAM63110.1|	F-box protein AtFBL5 [Arabidopsis thaliana]	1E-59
CaF1_WIE_12_D_07	180	gb|ABR80314.1|	putative transposase [Klebsiella pneumoniae subsp. pneumoniae MGH 78578] gb|ABR80449.1| mercuric resistance protein [Klebsiella pneumoniae subsp. pneumoniae MGH 78578]	6E-20
CaF1_WIE_12_E_04	424	gb|ABE80121.1|	Pyruvate kinase [Medicago truncatula] gb|ABE89087.1| Pyruvate kinase [Medicago truncatula]	4E-65
CaF1_WIE_12_F_01	483	gb|ABQ10186.1|	succinyl-CoA ligase beta subunit [Caragana jubata]	7E-77
CaF1_WIE_12_F_07	396	ref|NP_001050458.1|	Os03g0440900 [Oryza sativa (japonica cultivar-group)] gb|AAO23085.1| putative leucine-rich repeat protein [Oryza sativa (japonica cultivar-group)] gb|ABF96847.1| BRASSINOSTEROID INSENSITIVE 1-associated receptor kinase 1 precursor, putative, expressed [Oryza sativa (japonica cultivar-group)] dbj|BAF12372.1| Os03g0440900 [Oryza sativa (japonica cultivar-group)] gb|EAY90604.1| hypothetical protein OsI_011837 [Oryza sativa (indica cultivar-group)] gb|EAZ27458.1| hypothetical protein OsJ_010941 [Oryza sativa (japonica cultivar-group)]	7E-19
CaF1_WIE_12_F_08	140	gb|AF033850.1|AF033850	Homo sapiens phospholipase D2 (PLD2) mRNA, complete cds	0.002
CaF1_WIE_12_G_03	280	gb|AAS38575.1|	short-chain dehydrogenase Tic32 [Pisum sativum]	6E-25
CaF1_WIE_12_G_04	190	gb|ABO78621.1|	Translation factor [Medicago truncatula]	6E-21
CaF1_WIE_12_G_07	248	emb|CAC86996.1|	ATP citrate lyase b-subunit [Lupinus albus]	2E-22
CaF1_WIE_12_G_10	471	gb|ABE80179.1|	hypothetical protein MtrDRAFT_AC139601g19v2 [Medicago truncatula]	2E-53
CaF1_WIE_12_H_05	359	gb|ABE80160.2|	Prefoldin [Medicago truncatula]	6E-45
CaF1_WIE_12_H_10	238	emb|AJ749794.1|	Photobacterium damselae subsp. piscicida trpA gene for putative transposase and partial ORF1 DNA for hypothetical protein, clone pRDA13	0.00002
CaF1_WIE_13_A_05	464	gb|AAV50005.1|	14-3-3 family protein [Malus x domestica]	6E-55
CaF1_WIE_13_B_06	221	emb|AJ278505.1|CAR278505	Cicer arietinum mRNA for putative 14-kDa proline-rich protein (ORF1)	3E-71
CaF1_WIE_13_C_02	393	gb|ABO83636.1|	Glycoside hydrolase, clan GH-D; Raffinose synthase [Medicago truncatula]	2E-24
CaF1_WIE_13_C_10	461	dbj|BAE99246.1|	hypothetical protein [Arabidopsis thaliana]	2E-54
CaF1_WIE_13_C_11	454	gb|AAM34784.1|AF510671_1	HDA2 [Arabidopsis thaliana]	3E-65
CaF1_WIE_13_D_02	359	gb|ABE79269.1|	Regulator of chromosome condensation/beta-lactamase-inhibitor protein II [Medicago truncatula]	4E-40
CaF1_WIE_13_D_07	364	emb|CAN78893.1|	hypothetical protein [Vitis vinifera]	7E-35
CaF1_WIE_13_E_08	131	emb|AM422097.2|	Danio rerio sox3 mRNA, 3'UTR	0.000008
CaF1_WIE_13_F_03	197	gb|AY232724.1|	Fusarium oxysporum f. sp. vasinfectum strain Ag149-III Foxy transposable element, partial sequence	4E-52
CaF1_WIE_13_G_01	455	gb|AAD41024.1|	sucrose transport protein SUT1 [Pisum sativum]	1E-11
CaF1_WIE_13_H_04	144	emb|AM706411.1|	Eristalis tenax partial mRNA for hypothetical protein (ORF1), isolate 3	0.009
CaF1_WIE_14_C_06	429	ref|NP_001031840.1|	ATDFB (A. THALIANA DHFS-FPGS HOMOLOG B); tetrahydrofolylpolyglutamate synthase [Arabidopsis thaliana]	1E-30
CaF1_WIE_14_C_09	320	emb|AJ635223.1|	Pisum sativum mRNA for ftsH-like protease (ftsh4 gene)	2E-24

CaF1_WIE_14_C_11	206	dbj|AP004547.1|	Lotus japonicus genomic DNA, chromosome 6, clone:LjT31L24, TM0228a, complete sequence	2E-17
CaF1_WIE_14_D_10	399	emb|CAA67728.1|	pectinacetylesterase precursor [Vigna radiata var. radiata]	7E-38
CaF1_WIE_14_E_03	352	gb|AAZ79356.1|	aldehyde dehydrogenase [Vitis pseudoreticulata]	1E-46
CaF1_WIE_14_E_11	446	gb|AC141115.22|	Medicago truncatula clone mth2-16b23, complete sequence	1E-103
CaF1_WIE_14_G_01	256	emb|AJ404848.1|GMA404848	Glycine max mRNA for ribosomal protein L2 (rpL2 gene)	4E-37
CaF1_WIE_14_G_02	369	emb|CAJ91151.1|	AMP-binding protein [Platanus x acerifolia]	2E-45
CaF1_WIE_14_G_09	336	gb|ABN09080.1|	Heat shock protein DnaJ [Medicago truncatula]	2E-51
CaF1_WIE_14_H_02	381	emb|CAN62265.1|	hypothetical protein [Vitis vinifera]	1E-13
CaF1_WIE_15_A_02	486	emb|CAC10212.1|	putative mitochondrial glyoxalase II [Cicer arietinum]	1E-66
CaF1_WIE_15_A_04	337	sp|P51850|PDC1_PEA	Pyruvate decarboxylase isozyme 1 (PDC) emb|CAA91444.1| pyruvate decarboxylase [Pisum sativum]	8E-17
CaF1_WIE_15_B_02	446	emb|CAB83305.1|	putative protein [Arabidopsis thaliana]	1E-49
CaF1_WIE_15_F_08	459	gb|ABE91043.2|	Peptidase C13, legumain [Medicago truncatula] gb|ABP03557.1| Peptidase C13, legumain [Medicago truncatula]	6E-65
CaF1_WIE_15_G_06	445	gb|ABE83515.1|	hypothetical protein MtrDRAFT_AC129091g11v2 [Medicago truncatula]	4E-41
CaF1_WIE_15_H_09	450	dbj|BAF45465.1|	hypothetical protein [Nicotiana tabacum]	4E-43
CaF1_WIE_15_H_10	449	dbj|BAE62261.1|	unnamed protein product [Aspergillus oryzae]	6E-47
CaF1_WIE_16_A_11	454	gb|ABA81885.1|	profilin-like [Solanum tuberosum]	5E-56
CaF1_WIE_16_B_07	381	ref|NP_187764.1|	unknown protein [Arabidopsis thaliana] gb|AAG51447.1|AC008153_20 hypothetical protein; 89863-88075 [Arabidopsis thaliana]	1E-11
CaF1_WIE_16_B_10	454	emb|CAN78555.1|	hypothetical protein [Vitis vinifera]	2E-32
CaF1_WIE_16_C_03	282	dbj|BAD05166.1|	acid phosphatase [Phaseolus vulgaris] dbj|BAD05167.1| acid phosphatase [Phaseolus vulgaris]	4E-36
CaF1_WIE_16_C_09	266	emb|AJ749800.1|	Photobacterium damselae subsp. piscicida partial coi genes for putative cytochrome C oxidase proteins, clone pRDA19	0.00007
CaF1_WIE_16_C_10	452	emb|CAA10125.1|	40S ribosomal protein S19 [Cicer arietinum]	5E-44
CaF1_WIE_16_F_05	233	gb|AC159805.23|	Glycine max clone gmw2-173d12, complete sequence	5E-21
CaF1_WIE_16_G_04	454	emb|CAC10208.1|	cytosolic malate dehydrogenase [Cicer arietinum]	5E-23
CaF1_WIE_16_H_04	320	sp|P32289|GLNA_VIGAC	Glutamine synthetase nodule isozyme (Glutamate--ammonia ligase) (GS) gb|AAA34239.1| glutamine synthetase prf||2106409A Gln synthetase	7E-37
CaF1_WIE_16_H_06	288	emb|CAN70439.1|	hypothetical protein [Vitis vinifera]	3E-26
CaF1_WIE_17_A_10	467	gb|AY972077.1|	Synthetic construct RLS (RLS) gene, complete cds	0.0001
CaF1_WIE_17_A_11	466	gb|ABO77634.1|	peroxidase [Medicago truncatula]	1E-41
CaF1_WIE_17_B_03	400	emb|CT963114.2|	Medicago truncatula chromosome 5 clone mte1-26c9, COMPLETE SEQUENCE	5E-10
CaF1_WIE_17_E_01	459	gb|EAZ28165.1|	hypothetical protein OsJ_011648 [Oryza sativa (japonica cultivar-group)]	5E-32
CaF1_WIE_17_E_05	468	gb|ABE85045.1|	Cyclic peptide transporter [Medicago truncatula]	3E-63
CaF1_WIE_17_E_08	462	ref|NP_201196.1|	unknown protein [Arabidopsis thaliana] sp|P57681|PCYOX_ARATH Probable prenylcysteine oxidase precursor dbj|BAB11039.1| unnamed protein product [Arabidopsis thaliana] gb|AAL91144.1| unknown protein [Arabidopsis thaliana] gb|AAM91116.1| unknown protein [Arabidopsis thaliana]	9E-51
CaF1_WIE_17_F_09	460	emb|CAN65847.1|	hypothetical protein [Vitis vinifera]	1E-62
CaF1_WIE_17_G_10	314	emb|CAA10123.1|	hypothetical protein [Cicer arietinum]	1E-43
CaF1_WIE_18_A_09	458	emb|CT967318.5|	M.truncatula DNA sequence from clone MTH2-3P14 on chromosome 3, complete sequence	1E-20
CaF1_WIE_18_C_09	453	gb|ABI31652.1|	26S proteasome regulatory particle non-ATPase subunit 12 [Camellia sinensis]	5E-54
CaF1_WIE_18_D_06	167	gb|ABN08458.1|	Cytochrome b561 / ferric reductase transmembrane [Medicago truncatula]	1E-14
CaF1_WIE_18_D_11	336	gb|ABN05716.1|	Chaperone DnaK [Medicago truncatula]	6E-23
CaF1_WIE_18_F_03	485	gb|ABQ32305.1|	putative ubiquitin-conjugating enzyme [Artemisia annua]	3E-60
CaF1_WIE_18_F_05	395	emb|CAN69723.1|	hypothetical protein [Vitis vinifera]	3E-32
CaF1_WIE_18_F_06	486	emb|CAI84657.1|	hypothetical protein [Nicotiana tabacum]	1E-61
CaF1_WIE_18_F_11	463	gb|AAL23710.2|	cellulose synthase [Populus tremuloides]	2E-62
CaF1_WIE_18_H_02	192	gb|ABO80203.1|	Homeodomain-related [Medicago truncatula]	1E-19
CaF1_WIE_18_H_03	458	emb|CAJ38371.1|	HMG-protein [Plantago major]	7E-21
CaF1_WIE_19_A_01	288	gb|EAZ39797.1|	hypothetical protein OsJ_023280 [Oryza sativa (japonica cultivar-group)]	4E-29
CaF1_WIE_19_A_04	272	gb|BT009458.1|	Triticum aestivum clone wlsu2.pk0001.h3:fis, full insert mRNA sequence	0.0003
CaF1_WIE_19_A_07	443	gb|ABE84066.1|	SecY protein; ABC transporter related [Medicago truncatula]	3E-75

CaF1_WIE_19_A_10	307	ref|NP_195500.1|	phosphoenolpyruvate carboxykinase (ATP), putative / PEP carboxykinase, putative / PEPCK, putative [Arabidopsis thaliana] sp|Q9T074|PEPCK_ARATH Phosphoenolpyruvate carboxykinase [ATP] (PEP carboxykinase) (Phosphoenolpyruvate carboxylase) (PEPCK) gb|AAK50062.1|AF372922_1 AT4g37870/T28I19_150 [Arabidopsis thaliana] emb|CAB38935.1| phosphoenolpyruvate carboxykinase (ATP)-like protein [Arabidopsis thaliana] emb|CAB80452.1| phosphoenolpyruvate carboxykinase (ATP)-like protein [Arabidopsis thaliana] gb|AAL77736.1| AT4g37870/T28I19_150 [Arabidopsis thaliana] dbj|BAE98480.1| phosphoenolpyruvate carboxykinase (ATP) -like protein [Arabidopsis thaliana]	2E-41
CaF1_WIE_19_B_03	124	ref|XP_386857.1|	hypothetical protein FG06681.1 [Gibberella zeae PH-1]	6E-12
CaF1_WIE_19_B_07	152	dbj|BAB85760.1|	putative mitochondrial carrier protein [Fusarium oxysporum]	9E-17
CaF1_WIE_19_B_08	255	gb|ABC75374.1|	SGS; HSP20-like chaperone [Medicago truncatula]	9E-35
CaF1_WIE_19_C_04	184	ref|XP_386326.1|	hypothetical protein FG06150.1 [Gibberella zeae PH-1]	1E-11
CaF1_WIE_19_C_10	200	ref|NP_567126.1|	glycoside hydrolase family 28 protein / polygalacturonase (pectinase) family protein [Arabidopsis thaliana] gb|AAG40344.1|AF324992_1 AT3g62110 [Arabidopsis thaliana] gb|AAN31866.1| unknown protein [Arabidopsis thaliana]	3E-20
CaF1_WIE_19_E_01	128	emb|CAB56744.1|	cytochrome P450 monooxygenase [Cicer arietinum]	7E-13
CaF1_WIE_19_G_01	429	sp|Q01289|POR_PEA	Protochlorophyllide reductase, chloroplast precursor (PCR) (NADPH-protochlorophyllide oxidoreductase) (POR) emb|CAA44786.1| protochlorophyllide reductase [Pisum sativum]	8E-28
CaF1_WIE_19_G_11	457	emb|CAB96990.1|	putative 14-kDa proline-rich protein [Cicer arietinum]	6E-42
CaF1_WIE_19_H_10	462	emb|CAG14979.1|	non-cyanogenic beta-glucosidase [Cicer arietinum]	8E-63
CaF1_WIE_20_A_06	462	emb|CAN82256.1|	hypothetical protein [Vitis vinifera]	4E-51
CaF1_WIE_20_B_08	341	sp|O82043|ILV5_PEA	Ketol-acid reductoisomerase, chloroplast precursor (Acetohydroxy-acid reductoisomerase) (Alpha-keto-beta-hydroxylacil reductoisomerase) emb|CAA76854.1| ketol-acid reductoisomerase [Pisum sativum]	4E-48
CaF1_WIE_20_C_01	216	gb|AC137838.40|	Medicago truncatula clone mth2-34l12, complete sequence	9E-38
CaF1_WIE_20_C_09	368	gb|EAY92246.1|	hypothetical protein OsI_013479 [Oryza sativa (indica cultivar-group)] gb|EAZ28970.1| hypothetical protein OsJ_012453 [Oryza sativa (japonica cultivar-group)]	9E-48
CaF1_WIE_20_D_05	426	dbj|BAC58013.1|	S-adenosyl-L-methionine: 2,7,4'-trihydroxyisoflavanone 4'-O-methyltransferase [Lotus japonicus]	6E-21
CaF1_WIE_20_E_02	339	gb|ABO84368.1|	Cell division protein FtsZ [Medicago truncatula]	4E-41
CaF1_WIE_20_E_03	380	ref|NP_175129.3|	unknown protein [Arabidopsis thaliana] gb|AAP37679.1| At1g45150 [Arabidopsis thaliana] dbj|BAE99332.1| hypothetical protein [Arabidopsis thaliana]	2E-44
CaF1_WIE_20_E_10	127	gb|AC110027.5|	Homo sapiens chromosome 15, clone RP11-452K20, complete sequence	0.03
CaF1_WIE_20_F_02	356	gb|AAT46998.1|	triosephosphate isomerase [Glycine max]	7E-35
CaF1_WIE_20_F_04	336	ref|XM_955692.1|	Neurospora crassa OR74A hypothetical protein (NCU08963.1) partial mRNA	3E-14
CaF1_WIE_20_F_05	454	gb|ABE81707.1|	Esterase/lipase/thioesterase; Peptidase S9B, dipeptidylpeptidase IV N-terminal [Medicago truncatula]	3E-65
CaF1_WIE_20_G_01	183	emb|CR962121.2|	Medicago truncatula chromosome 5 clone mte1-77f5, COMPLETE SEQUENCE	8E-44
CaF1_WIE_20_G_06	209	emb|CU179904.1|	Medicago truncatula chromosome 5 clone mth2-104i2, COMPLETE SEQUENCE	3E-25
CaF1_WIE_20_G_11	256	gb|EAZ20254.1|	hypothetical protein OsJ_034463 [Oryza sativa (japonica cultivar-group)]	2E-31
CaF1_WIE_20_H_06	121	gb|DQ455594.1|	Phaseolus vulgaris PP2c mRNA, partial cds	6E-19
CaF1_WIE_20_H_07	419	emb|AM486371.2|	Vitis vinifera contig VV78X233435.4, whole genome shotgun sequence	0.11
CaF1_WIE_21_B_06	424	sp|Q96551|METK_CATRO	S-adenosylmethionine synthetase 1 (Methionine adenosyltransferase 1) (AdoMet synthetase 1) emb|CAA95856.1| S-adenosyl-L-methionine synthetase 1 [Catharanthus roseus]	4E-52
CaF1_WIE_21_B_10	334	emb|CU405944.1|	Oryza rufipogon (W1943) cDNA clone: ORW1943C006H23, full insert sequence	1E-127
CaF1_WIE_21_C_04	229	emb|X95708.1|CANMT1MET	C.arietinum mRNA for metallothionein (clone: CanMT-1)	1E-95
CaF1_WIE_21_C_09	229	emb|AJ299066.1|CAR299066	Cicer arietinum partial mRNA for ubiquitin-conjugating enzyme E2 (ORF1), clone CanUBC-2	3E-93
CaF1_WIE_21_E_04	369	gb|AC144731.15|	Medicago truncatula clone mth2-5g18, complete sequence	3E-11
CaF1_WIE_21_E_07	460	gb|AAG51802.1|AC067754_18	phosphoglycerate dehydrogenase, putative; 33424-31403 [Arabidopsis thaliana]	2E-24
CaF1_WIE_21_E_10	314	gb|AC144930.26|	Medicago truncatula clone mth2-15e11, complete sequence	2E-36
CaF1_WIE_21_F_02	205	gb|ABO81708.1|	2-oxoglutarate dehydrogenase, E1 component [Medicago truncatula]	2E-27

CaF1_WIE_21_F_10	398	emb|CT967319.2|	M.truncatula DNA sequence from clone MTH2-59J17 on chromosome 3, complete sequence	4E-32
CaF1_WIE_21_G_11	128	gb|AF089724.1|AF089724	Pisum sativum signal recognition particle 54 kDa subunit precursor (Ffc) mRNA, nuclear gene encoding chloroplast protein, partial cds	1E-29
CaF1_WIE_21_H_09	435	gb|ABF13308.1|	PP2c [Phaseolus vulgaris]	2E-60
CaF1_WIE_22_C_02	281	gb|AY327035.1|	Ixodes ricinus cytochrome oxidase subunit I mRNA, partial cds; mitochondrial gene for mitochondrial product	0.0003
CaF1_WIE_22_H_07	417	gb|ABN08340.1|	ZIM [Medicago truncatula]	3E-16
CaF1_WIE_23_A_03	370	emb|CAA79177.1|	Tumor protein [Arabidopsis thaliana]	3E-12
CaF1_WIE_23_A_04	455	gb|AAS18240.1|	enolase [Glycine max]	1E-69
CaF1_WIE_23_B_04	419	gb|DQ008803.1|	Takhtajania perrieri large subunit ribosomal RNA gene, partial sequence; mitochondrial	0
CaF1_WIE_23_B_07	436	gb|ABE85435.1|	Protein kinase; NAF [Medicago truncatula] gb|ABO83915.1| Protein kinase; NAF [Medicago truncatula] gb|ABO84280.1| Protein kinase; NAF [Medicago truncatula]	2E-56
CaF1_WIE_23_C_03	310	emb|CAN69767.1|	hypothetical protein [Vitis vinifera]	2E-13
CaF1_WIE_23_D_03	274	gb|AC148237.7|	Medicago truncatula clone mth2-1o14, complete sequence	1E-15
CaF1_WIE_23_D_07	368	gb|AAA74456.1|	nitrite reductase	3E-57
CaF1_WIE_23_E_11	421	emb|CAN71280.1|	hypothetical protein [Vitis vinifera]	1E-52
CaF1_WIE_23_F_11	455	gb|ABE78146.1|	E1 protein and Def2/Der2 allergen [Medicago truncatula] gb|ABE83819.1| E1 protein and Def2/Der2 allergen [Medicago truncatula]	1E-32
CaF1_WIE_23_G_08	396	emb|CAA98170.1|	RAB7C [Lotus japonicus]	4E-58
CaF1_WIE_24_A_06	485	dbj|BAB02526.1|	unnamed protein product [Arabidopsis thaliana]	5E-38
CaF1_WIE_24_A_11	226	gb|AC165430.3|	Medicago truncatula chromosome 2 BAC clone mth2-67a21, complete sequence	5E-24
CaF1_WIE_24_B_01	368	ref|NP_564660.1|	unknown protein [Arabidopsis thaliana] gb|AAM13069.1| unknown protein [Arabidopsis thaliana] gb|AAM61748.1| unknown [Arabidopsis thaliana]	2E-31
CaF1_WIE_24_B_07	385	gb|ABE88784.1|	SH3 [Medicago truncatula]	6E-49
CaF1_WIE_24_B_08	416	emb|AM486183.1|	Vitis vinifera, whole genome shotgun sequence, contig VV78X166878.4, clone ENTAV 115	5E-16
CaF1_WIE_24_C_10	369	ref|NP_200750.1|	oxysterol-binding family protein [Arabidopsis thaliana] gb|AAK96664.1| oxysterol-binding protein [Arabidopsis thaliana] gb|AAN15434.1| oxysterol-binding protein [Arabidopsis thaliana]	1E-32
CaF1_WIE_24_D_04	364	gb|AAV69019.1|	NADH:cytochrome b5 reductase [Vernicia fordii] gb|AAV69021.1| NADH:cytochrome b5 reductase [Vernicia fordii]	4E-54
CaF1_WIE_24_E_10	232	gb|ABB16967.1|	unknown [Solanum tuberosum]	2E-31
CaF1_WIE_24_F_02	100	emb|AM466081.2|	Vitis vinifera contig VV78X185514.6, whole genome shotgun sequence	0.0000001
CaF1_WIE_24_F_03	411	gb|ABO84518.1|	WD40-like [Medicago truncatula]	3E-27
CaF1_WIE_24_F_04	156	emb|AM706411.1|	Eristalis tenax partial mRNA for hypothetical protein (ORF1), isolate 3	0.00004
CaF1_WIE_24_F_06	411	gb|AAK11734.1|	serine/threonine/tyrosine kinase [Arachis hypogaea]	5E-56
CaF1_WIE_24_G_08	385	emb|CAN83013.1|	hypothetical protein [Vitis vinifera]	4E-38
CaF1_WIE_24_G_09	168	gb|AC187645.7|	Glycine max clone gmp1-49d18, complete sequence	2E-16
CaF1_WIE_24_H_04	336	dbj|BAF42040.1|	pectin methylesterase 3 [Pyrus communis]	2E-40
CaF1_WIE_24_H_10	222	ref|XP_381029.1|	hypothetical protein FG00853.1 [Gibberella zeae PH-1]	1E-25
CaF1_WIE_25_A_01	465	ref|NP_564656.1|	LEM3 (ligand-effect modulator 3) family protein / CDC50 family protein [Arabidopsis thaliana] gb|AAD25612.1|AC005287_14 Unknown protein [Arabidopsis thaliana] gb|AAL38602.1|AF446869_1 At1g54320/F20D21_50 [Arabidopsis thaliana] gb|AAK74030.1| At1g54320/F20D21_50 [Arabidopsis thaliana] gb|AAK96636.1| At1g54320/F20D21_50 [Arabidopsis thaliana]	8E-46
CaF1_WIE_25_A_02	190	emb|CAA71762.1|	Ubiquitin activating enzyme E1 [Nicotiana tabacum]	4E-20
CaF1_WIE_25_B_07	271	gb|AAM21172.1|AF305635_1	serine/threonine protein phosphatase 2A [Pisum sativum]	1E-28
CaF1_WIE_25_C_10	448	gb|ABP03222.1|	Zinc finger, FYVE/PHD-type [Medicago truncatula]	4E-30
CaF1_WIE_25_D_05	448	sp|Q42908|PMGI_MESCR	2,3-bisphosphoglycerate-independent phosphoglycerate mutase (Phosphoglyceromutase) (BPG-independent PGAM) (PGAM-I) gb|AAA86979.1| phosphoglyceromutase	2E-49
CaF1_WIE_25_D_06	446	emb|CAN63304.1|	hypothetical protein [Vitis vinifera]	2E-27
CaF1_WIE_25_D_07	388	gb|AC139745.35|	Medicago truncatula clone mth2-17d15, complete sequence	3E-23
CaF1_WIE_25_E_01	157	gb|ABE91842.1|	Gonadotropin, beta chain; Gibberellin regulated protein [Medicago truncatula]	9E-11
CaF1_WIE_25_F_09	429	emb|AM706411.1|	Eristalis tenax partial mRNA for hypothetical protein (ORF1), isolate 3	0.0005
CaF1_WIE_25_G_04	424	gb|M31921.1|VVCH2AB	V.carteri histone H2A-III and H2B-III genes, complete cds	0.0005
CaF1_WIE_26_C_01	493	gb|AAO46881.1|	60S ribosomal protein [Medicago sativa]	9E-73
CaF1_WIE_26_C_06	190	ref|XM_846823.1|	PREDICTED: Canis familiaris similar to ribosomal protein L31 (LOC609548), mRNA	0.19

CaF1_WIE_26_D_06	215	gb|ABO84434.1|	Esterase/lipase/thioesterase [Medicago truncatula]	1E-16
CaF1_WIE_26_F_05	301	gb|AC155894.5|	Medicago truncatula chromosome 7 BAC clone mth2-67b7, complete sequence	2E-58
CaF1_WIE_26_F_11	192	gb|AAX85979.1|	NAC2 protein [Glycine max] gb|AAY46122.1| NAC domain protein NAC2 [Glycine max]	3E-29
CaF1_WIE_26_G_02	494	gb|AAB46611.1|	aspartate aminotransferase [Medicago sativa]	2E-52
CaF1_WIE_26_G_07	343	emb|CAN74446.1|	hypothetical protein [Vitis vinifera]	8E-31
CaF1_WIE_26_G_10	260	emb|CAC32462.1|	sucrose synthase isoform 3 [Pisum sativum]	3E-41
CaF1_WIE_26_H_02	360	emb|CT573077.3|	Medicago truncatula chromosome 5 clone mth2-43j18, COMPLETE SEQUENCE	0.00000003
CaF1_WIE_26_H_04	495	dbj|BAD94972.1|	putative protein [Arabidopsis thaliana]	1E-52
CaF1_WIE_26_H_06	317	gb|AC122458.3|	Mus musculus BAC clone RP24-270D10 from chromosome 8, complete sequence	0.083
CaF1_WIE_26_H_08	208	emb|CAN79702.1|	hypothetical protein [Vitis vinifera]	1E-20
CaF1_WIE_27_A_03	297	gb|ABN09177.1|	Single-stranded nucleic acid binding R3H [Medicago truncatula]	1E-20
CaF1_WIE_27_A_05	285	emb|AM706414.1|	Eristalis tenax partial mRNA for hypothetical protein (ORF1), isolate 6	0.0003
CaF1_WIE_27_A_06	418	gb|AAN38066.1|	oligomycin sensitivity conferring protein [Silene latifolia]	9E-14
CaF1_WIE_27_B_10	159	gb|AY972077.1|	Synthetic construct RLS (RLS) gene, complete cds	0.039
CaF1_WIE_27_C_03	212	ref|XP_381601.1|	PMA1_NEUCR Plasma membrane ATPase (Proton pump) [Gibberella zeae PH-1]	1E-20
CaF1_WIE_27_C_10	195	gb|AAZ67970.1|	At1g63940 [Arabidopsis thaliana] gb|AAZ67971.1| At1g63940 [Arabidopsis thaliana] gb|AAZ67972.1| At1g63940 [Arabidopsis thaliana] gb|AAZ67973.1| At1g63940 [Arabidopsis thaliana] gb|AAZ67974.1| At1g63940 [Arabidopsis thaliana] gb|AAZ67975.1| At1g63940 [Arabidopsis thaliana] gb|AAZ67976.1| At1g63940 [Arabidopsis thaliana] gb|AAZ67977.1| At1g63940 [Arabidopsis thaliana] gb|AAZ67978.1| At1g63940 [Arabidopsis thaliana] gb|AAZ67979.1| At1g63940 [Arabidopsis thaliana] gb|AAZ67980.1| At1g63940 [Arabidopsis thaliana] gb|AAZ67981.1| At1g63940 [Arabidopsis thaliana] gb|AAZ67982.1| At1g63940 [Arabidopsis thaliana] gb|AAZ67983.1| At1g63940 [Arabidopsis thaliana] gb|AAZ67984.1| At1g63940 [Arabidopsis thaliana] gb|AAZ67985.1| At1g63940-like protein [Arabidopsis lyrata]	1E-22
CaF1_WIE_27_D_06	449	gb|ABE87365.2|	Ubiquitin [Medicago truncatula]	9E-62
CaF1_WIE_27_D_07	208	emb|CU075768.4|	Zebrafish DNA sequence from clone DKEY-177P5 in linkage group 14, complete sequence	0.21
CaF1_WIE_27_E_01	497	sp|P39869|NIA_LOTJA	Nitrate reductase [NADH] (NR) emb|CAA56696.1| nitrate reductase (NADH) [Lotus japonicus]	2E-61
CaF1_WIE_27_E_02	241	gb|DQ251457.1|	Siniperca chuatsi transposase mRNA, partial cds	0.000001
CaF1_WIE_27_E_06	414	ref|XP_380918.1|	hypothetical protein FG00742.1 [Gibberella zeae PH-1]	3E-70
CaF1_WIE_27_F_01	450	gb|L10211.1|ALFIOM	Medicago sativa isoliquiritigenin 2'-O-methyltransferase mRNA, complete cds	0.002
CaF1_WIE_27_F_02	143	emb|CT573504.2|	Medicago truncatula chromosome 5 clone mth2-178o19, COMPLETE SEQUENCE	0.0000001
CaF1_WIE_27_G_09	351	gb|ABE92592.1|	RHO protein GDP dissociation inhibitor [Medicago truncatula]	4E-48
CaF1_WIE_27_G_10	418	gb|ABP03363.1|	RNA-binding region RNP-1 (RNA recognition motif) [Medicago truncatula]	1E-37
CaF1_WIE_27_H_05	478	sp|P22778|ATPO_IPOBA	ATP synthase delta chain, mitochondrial precursor (Oligomycin sensitivity conferral protein) (OSCP) gb|AAA33388.1| F-1-ATPase delta subunit precursor (EC 3.6.1.3)	4E-32
CaF1_WIE_27_H_10	386	gb|ABO82746.1|	RNA-binding region RNP-1 (RNA recognition motif) [Medicago truncatula]	3E-25
CaF1_WIE_28_A_05	413	gb|AAM61490.1|	60S ribosomal protein L13, BBC1 protein [Arabidopsis thaliana]	3E-54
CaF1_WIE_28_C_03	271	gb|AC121235.20|	Medicago truncatula clone mth2-21k24, complete sequence	4E-65
CaF1_WIE_28_D_11	229	emb|CAN83660.1|	hypothetical protein [Vitis vinifera]	1E-11
CaF1_WIE_28_E_01	452	emb|CAA72183.1|	annexin-like protein [Medicago sativa]	1E-58
CaF1_WIE_28_E_06	425	gb|ABP03118.1|	Rh-like protein/ammonium transporter [Medicago truncatula]	1E-27
CaF1_WIE_28_E_07	319	emb|CAD31714.1|	fructokinase-like protein [Cicer arietinum]	5E-46
CaF1_WIE_28_F_06	237	emb|AM464167.1|	Vitis vinifera contig VV78X141930.41, whole genome shotgun sequence	0.015
CaF1_WIE_28_F_08	422	gb|ABC86745.1|	pollen-specific protein [Vitis pseudoreticulata]	1E-17
CaF1_WIE_28_F_11	344	gb|ABE81122.1|	Cytochrome c, monohaem [Medicago truncatula] gb|ABO84810.1| Cytochrome c, monohaem [Medicago truncatula]	8E-55
CaF1_WIE_28_G_09	280	gb|ABE82748.1|	CCT [Medicago truncatula]	4E-29
CaF1_WIE_28_G_11	327	gb|ABD28560.1|	Alpha-1,4-glucan-protein synthase (UDP-forming) [Medicago truncatula] gb|ABO78407.1| Alpha-1,4-glucan-protein synthase (UDP-forming) [Medicago truncatula]	1E-49
CaF1_WIE_28_H_03	472	ref|XP_383076.1|	hypothetical protein FG02900.1 [Gibberella zeae PH-1]	2E-25

CaF1_WIE_28_H_05	343	gb|ABE92593.1|	Membrane attack complex component/perforin/complement C9 [Medicago truncatula]	6E-31
CaF1_WIE_29_B_02	482	gb|ABE78980.1|	Ras small GTPase, Rab type [Medicago truncatula]	2E-21
CaF1_WIE_29_B_07	464	ref|NP_001048802.1|	Os03g0123100 [Oryza sativa (japonica cultivar-group)] gb|AAN74837.1| Putative ubiquitin-conjugating enzyme [Oryza sativa (japonica cultivar-group)] gb|ABF93717.1| Ubiquitin-conjugating enzyme E2 I, putative, expressed [Oryza sativa (japonica cultivar-group)] dbj|BAF10716.1| Os03g0123100 [Oryza sativa (japonica cultivar-group)] gb|EAY88329.1| hypothetical protein OsI_009562 [Oryza sativa (indica cultivar-group)] gb|EAZ25409.1| hypothetical protein OsJ_008892 [Oryza sativa (japonica cultivar-group)]	4E-83
CaF1_WIE_29_B_08	463	gb|EAZ06542.1|	hypothetical protein OsI_027774 [Oryza sativa (indica cultivar-group)]	1E-14
CaF1_WIE_29_C_03	485	emb|CAN63881.1|	hypothetical protein [Vitis vinifera]	1E-51
CaF1_WIE_29_C_09	432	emb|CAN78769.1|	hypothetical protein [Vitis vinifera]	1E-21
CaF1_WIE_29_E_06	486	emb|CAA16672.1|	predicted protein [Arabidopsis thaliana]	1E-17
CaF1_WIE_29_E_09	463	emb|CAN67310.1|	hypothetical protein [Vitis vinifera]	3E-23
CaF1_WIE_29_E_10	312	gb|AC150981.14|	Medicago truncatula clone mth2-166m22, complete sequence	2E-18
CaF1_WIE_29_F_02	486	ref|XP_383424.1|	hypothetical protein FG03248.1 [Gibberella zeae PH-1]	1E-70
CaF1_WIE_29_F_08	426	dbj|AB182103.1|	Silene latifolia SlAPG mRNA for anther-specific proline-rich protein APG precursor, partial cds	0.0001
CaF1_WIE_29_H_10	361	emb|CT573421.5|	M.truncatula DNA sequence from clone MTH2-27B10 on chromosome 3, complete sequence	2E-12
CaF1_WIE_29_H_11	277	gb|ABE91475.2|	Glycine-rich protein, putative [Medicago truncatula]	1E-14
CaF1_WIE_30_A_11	468	gb|EAZ24579.1|	hypothetical protein OsJ_008062 [Oryza sativa (japonica cultivar-group)]	4E-18
CaF1_WIE_30_B_03	305	dbj|AP004505.1|	Lotus japonicus genomic DNA, chromosome 3, clone:LjT10E18, TM0035, complete sequence	2E-24
CaF1_WIE_30_B_09	210	gb|ABE68721.1|	putative IN2-1 protein [Arachis hypogaea]	1E-20
CaF1_WIE_30_D_03	358	gb|ABN08660.1|	hypothetical protein MtrDRAFT_AC157891g33v2 [Medicago truncatula] gb|ABE94640.2| hypothetical protein MtrDRAFT_AC141114g34v2 [Medicago truncatula]	7E-35
CaF1_WIE_30_D_06	459	ref|XP_388371.1|	hypothetical protein FG08195.1 [Gibberella zeae PH-1]	6E-70
CaF1_WIE_30_D_11	265	emb|CU207236.5|	M.truncatula DNA sequence from clone MTH2-56B11 on chromosome 3, complete sequence	2E-45
CaF1_WIE_30_E_04	312	gb|ABO81167.1|	3-isopropylmalate dehydratase large subunit [Medicago truncatula]	3E-25
CaF1_WIE_30_E_05	418	emb|CAE12168.2|	formate dehydrogenase [Quercus robur]	2E-65
CaF1_WIE_30_E_07	223	gb|BT009458.1|	Triticum aestivum clone wlsu2.pk0001.h3:fis, full insert mRNA sequence	0.00006
CaF1_WIE_30_E_09	287	gb|AAM63313.1|	Contains similarity to bHLH transcription factor GBOF-1 from Tulipa gesneriana gb|AF185269 [Arabidopsis thaliana]	6E-13
CaF1_WIE_30_F_11	456	gb|ABO82384.1|	Cytochrome b5; Armadillo-like helical [Medicago truncatula]	1E-72
CaF1_WIE_30_H_09	485	sp|Q42877|RPB2_SOLLC	DNA-directed RNA polymerase II subunit RPB2 (RNA polymerase II subunit B2) (RNA polymerase II subunit 2) (DNA-directed RNA polymerase II 135 kDa polypeptide) gb|AAC49273.1| RNA polymerase II subunit 2	4E-83
CaF1_WIE_31_D_09	382	gb|ABP02096.1|	Thaumatin, pathogenesis-related [Medicago truncatula]	1E-49
CaF1_WIE_31_D_10	194	dbj|BAF01964.1|	hypothetical protein [Arabidopsis thaliana]	4E-17
CaF1_WIE_31_E_05	363	emb|CU302248.6|	M.truncatula DNA sequence from clone MTH2-33P23 on chromosome 3, complete sequence	0.000006
CaF1_WIE_31_E_09	103	emb|AM748415.1|	Vigna unguiculata partial mRNA for putative proton-dependent oligopeptide transport (POT) family protein (AT3G54140 gene), clone 29	0.36
CaF1_WIE_31_F_11	453	gb|ABN07918.1|	Zinc finger, ZZ-type; Zinc finger, C2H2-type [Medicago truncatula]	1E-54
CaF1_WIE_31_G_06	298	emb|CAC08564.1|	wound-induced GSK-3-like protein [Medicago sativa subsp. x varia]	3E-38
CaF1_WIE_31_H_11	422	ref|XM_384325.1|	Gibberella zeae PH-1 chromosome 2 hypothetical protein (FG04149.1) partial mRNA	8E-15
CaF1_WIE_32_A_02	500	gb|AAN86061.1|	geranylgeranyl diphosphate synthase [Citrus unshiu]	4E-63
CaF1_WIE_32_A_04	432	gb|ABE81754.1|	Ctr copper transporter [Medicago truncatula]	1E-53
CaF1_WIE_32_B_02	320	gb|ABE83853.1|	Concanavalin A-like lectin/glucanase [Medicago truncatula]	2E-17
CaF1_WIE_32_B_07	297	emb|CAN65606.1|	hypothetical protein [Vitis vinifera]	2E-14
CaF1_WIE_32_C_08	223	emb|X14826.1|TRADH1	Trifolium repens Adh1 mRNA for alcohol dehydrogenase 1	1E-24
CaF1_WIE_32_C_09	350	ref|XP_380654.1|	conserved hypothetical protein [Gibberella zeae PH-1]	2E-50
CaF1_WIE_32_E_07	224	emb|CT028787.1|	Poplar cDNA sequences	0.000004
CaF1_WIE_32_E_09	451	gb|L34658.1|FSOGAGPOL	Fusarium oxysporum gag polyprotein (gag) gene, complete cds; pol polyprotein (pol) gene, complete cds	0.0000005
CaF1_WIE_32_E_11	282	gb|AF075691.1|	Crassostrea gigas BAT1 homolog mRNA, complete cds	0.00000008
CaF1_WIE_32_F_07	356	gb|DQ507301.1|	Belgica antarctica clone Ba-U40 CG32816-like mRNA, partial cds	0.0004

CaF1_WIE_32_G_04	406	ref|NP_683481.1|	unknown protein [Arabidopsis thaliana] gb|AAL38694.1| unknown protein [Arabidopsis thaliana] gb|AAM20199.1| unknown protein [Arabidopsis thaliana] gb|AAM67283.1| unknown [Arabidopsis thaliana] dbj|BAC42463.1| unknown protein [Arabidopsis thaliana]	8E-17
CaF1_WIE_32_G_05	194	gb|AY232722.1|	Fusarium oxysporum f. sp. vasinfectum strain Ag149 Foxy transposable element, partial sequence	2E-60
CaF1_WIE_32_G_11	322	gb|ABL98074.1|	chitinase-related agglutinin [Robinia pseudoacacia]	3E-21
CaF1_WIE_32_H_04	193	ref|NP_563662.1|	pectinesterase family protein [Arabidopsis thaliana] gb|AAF02886.1|AC009525_20 Similar to pectinesterases [Arabidopsis thaliana] gb|ABO38784.1| At1g02810 [Arabidopsis thaliana]	2E-19
CaF1_WIE_32_H_08	352	ref|NP_191627.1|	unknown protein [Arabidopsis thaliana] emb|CAB82672.1| putative protein [Arabidopsis thaliana] dbj|BAC41904.1| unknown protein [Arabidopsis thaliana] gb|AAO64825.1| At3g60680 [Arabidopsis thaliana]	5E-33
CaF1_WIE_32_H_09	240	emb|AJ749803.1|	Photobacterium damselae subsp. piscicida partial ORF1 DNA for hypothetical protein, clone pRDA24	0.00006
CaF1_WIE_32_H_10	239	gb|ABP03659.1|	Haem peroxidase, plant/fungal/bacterial [Medicago truncatula]	3E-21
CaF1_WIE_33_A_02	344	gb|AAM62609.1|	unknown [Arabidopsis thaliana]	2E-33
CaF1_WIE_33_A_03	378	ref|NP_182310.1|	zinc finger (B-box type) family protein [Arabidopsis thaliana] sp|O82256|COL13_ARATH Zinc finger protein CONSTANS-LIKE 13 gb|AAC63643.1| putative zinc-finger protein (B-box zinc finger domain) [Arabidopsis thaliana] gb|AAM15120.1| putative zinc-finger protein (B-box zinc finger domain) [Arabidopsis thaliana] gb|AAY56404.1| At2g47890 [Arabidopsis thaliana]	7E-11
CaF1_WIE_33_A_05	459	gb|AAL87150.1|AF480496_4	putative histidinol phosphate aminotransferase [Oryza sativa (japonica cultivar-group)]	2E-23
CaF1_WIE_33_B_02	461	gb|AC146705.11|	Medicago truncatula clone mth2-101f3, complete sequence	2E-18
CaF1_WIE_33_B_05	427	ref|NP_567575.1|	ERD3 (EARLY-RESPONSIVE TO DEHYDRATION 3) [Arabidopsis thaliana] ref|NP_849408.1| ERD3 (EARLY-RESPONSIVE TO DEHYDRATION 3) [Arabidopsis thaliana] dbj|BAB63914.1| ERD3 protein [Arabidopsis thaliana]	3E-51
CaF1_WIE_33_C_08	427	dbj|AB182972.1|	Rana pirica mRNA for trypsinogen, partial sequence, clone:No 304	0.0001
CaF1_WIE_33_D_10	324	ref|XP_001245473.1|	hypothetical protein CIMG_04914 [Coccidioides immitis RS] gb|EAS33890.1| hypothetical protein CIMG_04914 [Coccidioides immitis RS]	9E-16
CaF1_WIE_33_E_02	460	ref|NP_186958.1|	NADP-dependent oxidoreductase, putative [Arabidopsis thaliana] gb|AAF26116.1|AC012328_19 putative NADP-dependent oxidoreductase [Arabidopsis thaliana]	5E-56
CaF1_WIE_33_F_09	200	emb|Z23097.1|PSMETALLP	P.sativum gene for metallothionein-like protein	0.00000005
CaF1_WIE_33_G_10	200	dbj|BAA92699.1|	type 2A protein phosphatase-3 [Vicia faba]	1E-25
CaF1_WIE_33_H_06	229	sp|P25317|GSTXA_TOBAC	Probable glutathione S-transferase parA (Auxin-regulated protein parA) (STR246C protein) gb|AAA67894.1| par peptide emb|CAA56790.1| STR246C [Nicotiana tabacum]	8E-12
CaF1_WIE_34_A_03	149	emb|AJ299396.1|CAR299396	Cicer arietinum partial mRNA for putative extensin (ORF), clone CanEXT-1	4E-11
CaF1_WIE_34_A_04	464	gb|ABE81765.1|	Translation initiation factor eIF-3b [Medicago truncatula]	1E-78
CaF1_WIE_34_B_08	457	sp|P52904|ODPB_PEA	Pyruvate dehydrogenase E1 component subunit beta, mitochondrial precursor (PDHE1-B) gb|AAB01223.1| pyruvate dehydrogenase E1beta	1E-63
CaF1_WIE_34_C_05	446	gb|ABK15530.1|	guanylyl cyclase [Glycine max]	3E-53
CaF1_WIE_34_C_06	249	gb|EAZ14922.1|	hypothetical protein OsJ_004747 [Oryza sativa (japonica cultivar-group)]	2E-19
CaF1_WIE_34_C_09	226	emb|CAA47810.1|	pectinesterase [Pisum sativum]	1E-19
CaF1_WIE_34_D_02	250	ref|XM_387051.1|	Gibberella zeae PH-1 chromosome 4 hypothetical protein (FG06875.1) partial mRNA	5E-18
CaF1_WIE_34_E_07	209	emb|CAN65460.1|	hypothetical protein [Vitis vinifera]	4E-17
CaF1_WIE_34_E_11	423	emb|CAD33928.1|	tonoplast intrinsic protein [Cicer arietinum]	1E-31
CaF1_WIE_34_F_01	264	gb|ABI34093.1|	homocysteine S-methyltransferase [Medicago sativa]	5E-33
CaF1_WIE_34_F_10	416	gb|ABE86426.2|	Heat shock protein 101. (exp=-1; wgp=0; cg=-1; geno, related [Medicago truncatula]	2E-47
CaF1_WIE_34_F_11	295	gb|ABE93168.1|	Prefoldin; Helix-loop-helix DNA-binding [Medicago truncatula]	8E-20
CaF1_WIE_34_G_01	454	emb|CAN70797.1|	hypothetical protein [Vitis vinifera]	2E-62
CaF1_WIE_34_G_06	415	ref|NP_001056664.1|	Os06g0127500 [Oryza sativa (japonica cultivar-group)] dbj|BAC24834.1| putative RNA-binding protein [Oryza sativa (japonica cultivar-group)] dbj|BAD67747.1| putative RNA-binding protein [Oryza sativa (japonica cultivar-group)] dbj|BAF18578.1| Os06g0127500 [Oryza sativa (japonica cultivar-group)] gb|EAZ35678.1| hypothetical protein OsJ_019161 [Oryza sativa (japonica cultivar-group)]	1E-14

CaF1_WIE_34_H_03	387	ref|NP_174119.1|	pyridoxal-dependent decarboxylase family protein [Arabidopsis thaliana] sp|Q9C509|SGPL_ARATH Sphingosine-1-phosphate lyase (SP-lyase) (SPL) (Sphingosine-1-phosphate aldolase) gb|AAG51494.1|AC069471_25 sphingosine-1-phosphate lyase, putative [Arabidopsis thaliana] gb|AAK25876.1|AF360166_1 putative sphingosine-1-phosphate lyase [Arabidopsis thaliana] gb|AAM44962.1| putative sphingosine-1-phosphate lyase [Arabidopsis thaliana] dbj|BAD13416.1| sphingosine-1-phosphate lyase [Arabidopsis thaliana]	2E-51
CaF1_WIE_34_H_09	460	emb|CAB95829.1|	hypothetical protein [Cicer arietinum]	1E-77
CaF1_WIE_34_H_11	464	gb|ABB02640.1|	unknown [Solanum tuberosum]	4E-67
CaF1_WIE_35_C_09	371	emb|CAN83232.1|	hypothetical protein [Vitis vinifera]	1E-13
CaF1_WIE_35_D_02	197	ref|NP_195785.1|	macrophage migration inhibitory factor family protein / MIF family protein [Arabidopsis thaliana] emb|CAB82281.1| light-inducible protein ATLS1 [Arabidopsis thaliana] gb|AAL32937.1| light-inducible protein ATLS1 [Arabidopsis thaliana] gb|AAM10137.1| light-inducible protein ATLS1 [Arabidopsis thaliana]	9E-14
CaF1_WIE_35_E_06	296	emb|AM706411.1|	Eristalis tenax partial mRNA for hypothetical protein (ORF1), isolate 3	0.00008
CaF1_WIE_35_E_08	466	gb|AAN77150.1|	fiber protein Fb11 [Gossypium barbadense]	2E-19
CaF1_WIE_35_F_01	460	dbj|BAE71236.1|	putative ADP,ATP carrier-like protein [Trifolium pratense]	3E-58
CaF1_WIE_35_F_08	451	emb|CAN82979.1|	hypothetical protein [Vitis vinifera]	2E-42
CaF1_WIE_35_F_11	189	dbj|AP004971.1|	Lotus japonicus genomic DNA, chromosome 5, clone:LjT45G21, TM0151, complete sequence	0.0000002
CaF1_WIE_35_G_02	181	emb|CU302347.1|	Medicago truncatula chromosome 5 clone mth2-5p5, COMPLETE SEQUENCE	1E-24
CaF1_WIE_35_G_07	463	gb|ABE93021.1|	Protein prenyltransferase [Medicago truncatula]	1E-65
CaF1_WIE_36_A_03	454	ref|NP_189150.1|	QUA1 (QUASIMODO1); polygalacturonate 4-alpha-galacturonosyltransferase/ transferase, transferring glycosyl groups / transferase, transferring hexosyl groups [Arabidopsis thaliana] sp|Q9LSG3|QUA1_ARATH Glycosyltransferase QUASIMODO1 dbj|BAB02072.1| unnamed protein product [Arabidopsis thaliana] gb|AAM20426.1| glycosyl transferase, putative [Arabidopsis thaliana] gb|AAQ56836.1| At3g25140 [Arabidopsis thaliana]	2E-76
CaF1_WIE_36_A_05	175	emb|CAN65024.1|	hypothetical protein [Vitis vinifera]	3E-12
CaF1_WIE_36_A_08	304	gb|AC152818.17|	Medicago truncatula clone mth2-85g12, complete sequence	2E-15
CaF1_WIE_36_A_09	377	emb|CAA06731.1|	GDP dissociation inhibitor [Cicer arietinum]	1E-44
CaF1_WIE_36_B_06	239	dbj|AP004940.1|	Lotus japonicus genomic DNA, chromosome 3, clone:LjT48I11, TM0106, complete sequence	3E-16
CaF1_WIE_36_B_08	390	gb|AAF25357.1|AF210061_1	dirigent protein [Forsythia x intermedia]	8E-25
CaF1_WIE_36_B_10	170	emb|CT028832.1|	Poplar cDNA sequences	0.0007
CaF1_WIE_36_B_11	433	ref|NP_200619.1|	unknown protein [Arabidopsis thaliana] gb|AAL58914.1|AF462824_1 AT5g58110/k21l19_90 [Arabidopsis thaliana] dbj|BAB11003.1| unnamed protein product [Arabidopsis thaliana] gb|AAM19978.1| AT5g58110/k21l19_90 [Arabidopsis thaliana]	6E-44
CaF1_WIE_36_C_09	451	gb|AAP72282.2|	calcium-dependent calmodulin-independent protein kinase isoform 2 [Cicer arietinum]	1E-76
CaF1_WIE_36_D_01	340	gb|ABE78703.1|	Heat shock protein DnaJ [Medicago truncatula]	2E-22
CaF1_WIE_36_D_07	261	emb|AJ534351.1|ABI534351	Agaricus bisporus partial mRNA for putative myosin heavy chain kinase (mhck gene), clone pm31	0.000004
CaF1_WIE_36_D_10	112	gb|AC174299.9|	Medicago truncatula clone mth2-80e19, complete sequence	5E-13
CaF1_WIE_36_E_03	225	gb|DQ465789.1|	Sesbania drummondii clone SSH-36_01_A09_T3 mRNA sequence	0.000004
CaF1_WIE_36_E_04	155	gb|AF075691.1|	Crassostrea gigas BAT1 homolog mRNA, complete cds	0.0002
CaF1_WIE_36_E_07	285	gb|AC146649.17|	Medicago truncatula clone mth2-10d6, complete sequence	1E-22
CaF1_WIE_36_E_09	142	gb|DQ445143.1|	Beta vulgaris chromosome 9 clone BAC123 genomic sequence	0.009
CaF1_WIE_36_F_01	256	emb|CAE76635.1|	cyclophilin-type peptidyl-prolyl cis-trans isomerase [Cicer arietinum]	5E-12
CaF1_WIE_36_G_06	104	gb|AC124963.32|	Medicago truncatula clone mth2-24f5, complete sequence	7E-15
CaF1_WIE_36_H_04	183	gb|ABE79819.2|	GroEL-like chaperone, ATPase [Medicago truncatula]	3E-22
CaF1_WIE_36_H_07	320	emb|CAN81538.1|	hypothetical protein [Vitis vinifera]	1E-20
CaF1_WIE_37_A_10	284	gb|AAK84885.1|AF402604_1	homeodomain leucine zipper protein HDZ1 [Phaseolus vulgaris]	3E-31
CaF1_WIE_37_C_10	135	emb|AM706411.1|	Eristalis tenax partial mRNA for hypothetical protein (ORF1), isolate 3	0.0001
CaF1_WIE_37_E_04	195	emb|AM748410.1|	Vigna unguiculata partial mRNA for putative CBL-interacting protein kinase 12 (CIPK12 gene), clone 24	0.0000008
CaF1_WIE_37_E_06	452	gb|ABG45901.1|	actin [Cryptosporidium sp. Czech B1] gb|ABG45905.1| actin [Cryptosporidium sp. BB23] gb|ABG45906.1| actin [Cryptosporidium sp. BP1]	1E-12
CaF1_WIE_37_E_07	461	gb|ABE85250.1|	3-phosphoshikimate 1-carboxyvinyltransferase [Medicago truncatula]	5E-60
CaF1_WIE_37_E_11	291	emb|CU062643.7|	M.truncatula DNA sequence from clone MTH2-144G2 on chromosome 3, complete sequence	4E-19

CaF1_WIE_37_G_01	220	dbj|AP004973.1|	Lotus japonicus genomic DNA, chromosome 3, clone:LjT41A07, TM0155b, complete sequence	5E-24
CaF1_WIE_37_G_02	446	gb|ABE93744.2|	FAR1; Heavy metal transport/detoxification protein; Zinc finger, SWIM-type [Medicago truncatula]	1E-66
CaF1_WIE_37_G_08	457	gb|ABE85053.1|	Universal stress protein (Usp) [Medicago truncatula]	1E-19
CaF1_WIE_37_G_09	253	gb|AC140914.20|	Medicago truncatula clone mth2-18h17, complete sequence	2E-39
CaF1_WIE_37_H_02	462	dbj|BAA76420.1|	multidrug resistance protein [Cicer arietinum]	3E-20
CaF1_WIE_37_H_04	182	dbj|BAD46202.1|	hypothetical protein [Oryza sativa (japonica cultivar-group)]	4E-18
CaF1_WIE_38_A_05	204	gb|AY099112.1|	Rattus norvegicus obese protein gene, 5' flanking region and partial cds	6E-11
CaF1_WIE_38_A_08	219	dbj|AB262513.1|	Pseudomonas aeruginosa gene for 16S rRNA, partial sequence, strain: Hg2	8E-29
CaF1_WIE_38_B_11	235	gb|DQ485185.1|	Catharanthus trichophyllus genotype CtN58 microsatellite CATR10 sequence	2E-82
CaF1_WIE_38_C_05	204	gb|AY099112.1|	Rattus norvegicus obese protein gene, 5' flanking region and partial cds	6E-11
CaF1_WIE_38_C_09	152	gb|DQ485192.1|	Thevetia peruviana microsatellite CATR25 sequence	2E-41
CaF1_WIE_38_C_11	228	gb|DQ485185.1|	Catharanthus trichophyllus genotype CtN58 microsatellite CATR10 sequence	2E-82
CaF1_WIE_38_D_08	280	dbj|AB262513.1|	Pseudomonas aeruginosa gene for 16S rRNA, partial sequence, strain: Hg2	1E-28
CaF1_WIE_38_D_10	273	gb|DQ485193.1|	Nerium oleander microsatellite CATR25 sequence	1E-28
CaF1_WIE_38_E_07	251	gb|DQ485193.1|	Nerium oleander microsatellite CATR25 sequence	4E-31
CaF1_WIE_38_F_09	428	dbj|AB262513.1|	Pseudomonas aeruginosa gene for 16S rRNA, partial sequence, strain: Hg2	2E-28
CaF1_WIE_38_F_10	276	gb|DQ485193.1|	Nerium oleander microsatellite CATR25 sequence	7E-33
CaF1_WIE_38_G_10	258	dbj|AB262513.1|	Pseudomonas aeruginosa gene for 16S rRNA, partial sequence, strain: Hg2	9E-29
CaF1_WIE_38_G_11	240	gb|DQ485185.1|	Catharanthus trichophyllus genotype CtN58 microsatellite CATR10 sequence	2E-82
CaF1_WIE_38_H_04	204	gb|AY099112.1|	Rattus norvegicus obese protein gene, 5' flanking region and partial cds	6E-11
CaF1_WIE_38_H_06	447	gb|AC159145.4|	Medicago truncatula chromosome 2 BAC clone mth2-67e10, complete sequence	6E-25
CaF1_WIE_38_H_11	255	gb|DQ485185.1|	Catharanthus trichophyllus genotype CtN58 microsatellite CATR10 sequence	2E-82
CaF1_WIE_39_A_08	480	gb|ABE84767.2|	ABC-2; AAA ATPase [Medicago truncatula]	5E-73
CaF1_WIE_39_A_11	404	emb|CAB65893.1|	hypotethical protein, homologous to ORF8 of pRiA4 [Agrobacterium rhizogenes]	4E-13
CaF1_WIE_39_B_02	459	ref|XM_001524953.1|	Lodderomyces elongisporus NRRL YB-4239 hypothetical protein (LELG_04035) mRNA, complete cds	0.49
CaF1_WIE_39_B_11	453	ref|NP_565409.1|	phosphate-responsive 1 family protein [Arabidopsis thaliana] gb|AAD25141.1| expressed protein [Arabidopsis thaliana] gb|AAL24171.1| At2g17230/T23A1.9 [Arabidopsis thaliana] gb|AAL90964.1| At2g17230/T23A1.9 [Arabidopsis thaliana]	3E-61
CaF1_WIE_39_C_09	455	ref|NP_196765.2|	carbon-nitrogen hydrolase family protein [Arabidopsis thaliana] gb|AAL91613.1| AT5g12040/F14F18_210 [Arabidopsis thaliana] gb|AAM10335.1| AT5g12040/F14F18_210 [Arabidopsis thaliana]	6E-52
CaF1_WIE_39_C_10	420	gb|AY847700.1|	Catharanthus roseus clone CrP15 T-DNA sequence	9E-98
CaF1_WIE_39_F_09	420	gb|AY847700.1|	Catharanthus roseus clone CrP15 T-DNA sequence	1E-96
CaF1_WIE_39_G_09	456	gb|ABE80590.2|	hypothetical protein MtrDRAFT_AC148398g42v2 [Medicago truncatula]	2E-39
CaF1_WIE_39_H_04	451	gb|ABB47998.1|	Zinc finger, C3HC4 type family protein, expressed [Oryza sativa (japonica cultivar-group)]	1E-33
CaF1_WIE_39_H_06	357	sp|Q9SC12|IF5A_SENVE	Eukaryotic translation initiation factor 5A (eIF-5A) emb|CAB65463.1| translation initiation factor 5A precursor protein (eIF-5A) [Senecio vernalis]	6E-18
CaF1_WIE_39_H_07	215	gb|ABE81390.1|	N-6 Adenine-specific DNA methylase [Medicago truncatula]	9E-19
CaF1_WIE_39_H_08	195	emb|AM748410.1|	Vigna unguiculata partial mRNA for putative CBL-interacting protein kinase 12 (CIPK12 gene), clone 24	0.0000008
CaF1_WIE_40_A_05	453	emb|CAN64202.1|	hypothetical protein [Vitis vinifera]	2E-36
CaF1_WIE_40_A_06	223	gb|ABE79560.1|	Chaperone DnaK [Medicago truncatula]	3E-13
CaF1_WIE_40_A_07	461	gb|ABE80525.1|	Pentatricopeptide repeat [Medicago truncatula]	3E-68
CaF1_WIE_40_C_03	293	gb|AY461597.1|	Synthetic construct arsenic-like protein gene, complete cds	0.005
CaF1_WIE_40_C_10	200	ref|NP_001049320.1|	Os03g0206600 [Oryza sativa (japonica cultivar-group)] gb|ABF94553.1| integral membrane protein, putative, expressed [Oryza sativa (japonica cultivar-group)] dbj|BAF11234.1| Os03g0206600 [Oryza sativa (japonica cultivar-group)]	5E-14
CaF1_WIE_40_D_06	214	gb|AAC28536.1|	putative beta-amylase [Arabidopsis thaliana]	2E-27
CaF1_WIE_40_E_04	448	gb|ABN05791.1|	TrkA-N [Medicago truncatula] gb|ABP02181.1| TrkA-N [Medicago truncatula]	3E-45

CaF1_WIE_40_F_01	455	ref|NP_564506.1|	unknown protein [Arabidopsis thaliana] sp|Q9SX77|UMP6_ARATH Unknown protein At1g47420, mitochondrial precursor gb|AAD46040.1|AC007519_25 ESTs gb|H36253 and gb|AA04251 come from this gene. [Arabidopsis thaliana] gb|AAK06877.1|AF344326_1 unknown protein [Arabidopsis thaliana] gb|AAK59453.1| unknown protein [Arabidopsis thaliana] gb|AAK96754.1| Unknown protein [Arabidopsis thaliana] gb|AAL34164.1| unknown protein [Arabidopsis thaliana] gb|AAM60959.1| unknown [Arabidopsis thaliana] gb|AAN15664.1| Unknown protein [Arabidopsis thaliana]	6E-21
CaF1_WIE_40_F_08	461	gb|ABB55398.1|	40S ribosomal protein S10-like [Solanum tuberosum]	1E-46
CaF1_WIE_40_G_02	170	gb|AF075691.1|	Crassostrea gigas BAT1 homolog mRNA, complete cds	0.00000001
CaF1_WIE_40_G_07	199	gb|AY972077.1|	Synthetic construct RLS (RLS) gene, complete cds	0.0002
CaF1_WIE_40_G_08	279	gb|DQ459385.1|	Nicotiana tabacum serine/threonine kinase mRNA, partial cds	0.00008
CaF1_WIE_40_H_09	314	gb|ABE89800.2|	eIF4-gamma/eIF5/eIF2-epsilon [Medicago truncatula]	2E-29
CaF1_WIE_41_A_05	279	gb|ABE85043.1|	HSP20-like chaperone [Medicago truncatula]	2E-23
CaF1_WIE_41_A_06	461	emb|CAN81423.1|	hypothetical protein [Vitis vinifera]	2E-51
CaF1_WIE_41_B_09	384	gb|ABO84487.1|	hypothetical protein MtrDRAFT_AC174293g6v2 [Medicago truncatula]	3E-18
CaF1_WIE_41_C_08	458	gb|ABE93295.1|	Leucine-rich repeat; Leucine-rich repeat, cysteine-containing subtype [Medicago truncatula]	1E-56
CaF1_WIE_41_D_01	430	gb|ABF06706.1|	UP-9A [Nicotiana tabacum]	1E-11
CaF1_WIE_41_E_01	464	gb|ABO84738.1|	Harpin-induced 1 [Medicago truncatula]	9E-35
CaF1_WIE_41_E_04	462	sp|Q39445|TBB_CICAR	Tubulin beta chain (Beta tubulin) emb|CAA67056.1| beta-tubulin [Cicer arietinum]	2E-38
CaF1_WIE_41_G_03	463	gb|ABK06434.1|	flag-tagged protein kinase domain of putative mitogen-activated protein kinase kinase kinase [synthetic construct]	3E-54
CaF1_WIE_41_G_08	463	ref|NP_569023.1|	unknown protein [Arabidopsis thaliana] gb|AAL38822.1| unknown protein [Arabidopsis thaliana] gb|AAM51275.1| unknown protein [Arabidopsis thaliana]	3E-50
CaF1_WIE_41_H_01	455	emb|Y17329.1|PSA17329	Pisum sativum mRNA for calnexin	3E-39
CaF1_WIE_41_H_04	345	dbj|AP004505.1|	Lotus japonicus genomic DNA, chromosome 3, clone:LjT10E18, TM0035, complete sequence	4E-38
CaF1_WIE_41_H_09	462	gb|ABP02866.1|	Ribosomal protein L30e [Medicago truncatula]	3E-58
CaF1_WIE_42_A_02	282	gb|ABE84980.1|	NAD-binding site; Nucleotide sugar epimerase [Medicago truncatula]	2E-42
CaF1_WIE_42_E_04	405	gb|AAB40396.1|	glycolate oxidase [Mesembryanthemum crystallinum]	5E-49
CaF1_WIE_42_E_09	334	emb|CR626927.1|	Bacteroides fragilis NCTC 9343, complete genome	0.088
CaF1_WIE_42_F_08	276	emb|CAN83660.1|	hypothetical protein [Vitis vinifera]	8E-20
CaF1_WIE_42_G_01	419	emb|AJ299396.1|CAR299396	Cicer arietinum partial mRNA for putative extensin (ORF), clone CanEXT-1	1E-38
CaF1_WIE_42_H_11	442	gb|ABE81079.1|	PpiC-type peptidyl-prolyl cis-trans isomerase [Medicago truncatula] gb|ABE89370.1| peptidyl-prolyl cis-trans isomerase 1 (ec 5.2.1.8) (rotamase pin1)(ppiase pin1) (mdpin1) [Medicago truncatula]	1E-46
CaF1_WIE_43_C_10	457	emb|CAC43237.1|	lipoxygenase [Sesbania rostrata]	1E-43
CaF1_WIE_43_D_10	188	emb|CAN69766.1|	hypothetical protein [Vitis vinifera]	1E-10
CaF1_WIE_43_D_11	460	gb|AAT94364.1|	chalcone isomerase 1B2 [Glycine max]	4E-53
CaF1_WIE_43_F_02	419	emb|CT028787.1|	Poplar cDNA sequences	0.00003
CaF1_WIE_43_F_10	259	emb|Y16672.1|MSY16672	Medicago sativa mRNA for putative arginine/serine-rich splicing factor, (sclp gene)	7E-39
CaF1_WIE_43_G_02	450	ref|XP_359800.2|	ribosomal protein L39 [Magnaporthe grisea 70-15] gb|EDJ95441.1| ribosomal protein L39 [Magnaporthe grisea 70-15] gb|EDN06404.1| ribosomal protein L39 [Ajellomyces capsulatus NAm1]	6E-23
CaF1_WIE_43_G_05	120	emb|Z93765.1|MDZ93765	M.domestica mRNA for lignostilbene dioxygenase-like protein	0.11
CaF1_WIE_43_H_02	411	gb|ABE93021.1|	Protein prenyltransferase [Medicago truncatula]	7E-37
CaF1_WIE_43_H_03	444	emb|CAH66506.1|	OSIGBa0111I14.1 [Oryza sativa (indica cultivar-group)]	7E-42
CaF1_WIE_43_H_07	454	dbj|BAD06518.1|	hypothetical protein [Pisum sativum] dbj|BAD12184.1| 12-oxophytodienoic acid 10, 11-reductase [Pisum sativum] gb|AAX54688.1| 12-oxophytodienoic acid 10,10-reductase [Pisum sativum]	3E-67
CaF1_WIE_44_B_06	341	gb|EAY85769.1|	hypothetical protein OsI_007002 [Oryza sativa (indica cultivar-group)]	1E-29
CaF1_WIE_44_C_04	373	ref|NP_001050957.1|	Os03g0691800 [Oryza sativa (japonica cultivar-group)] gb|AAT76985.1| putative HIPL1 protein [Oryza sativa (japonica cultivar-group)] gb|ABF98298.1| HIPL1 protein precursor, putative, expressed [Oryza sativa (japonica cultivar-group)] dbj|BAF12871.1| Os03g0691800 [Oryza sativa (japonica cultivar-group)] gb|EAZ28206.1| hypothetical protein OsJ_011689 [Oryza sativa (japonica cultivar-group)]	1E-51
CaF1_WIE_44_C_11	130	dbj|AB182973.1|	Rana pirica mRNA for trypsinogen, partial sequence, clone:No 316	0.0000005
CaF1_WIE_44_D_02	478	dbj|BAE93460.1|	diacylglycerolacyltransferase-1a [Glycine max]	5E-71

CaF1_WIE_44_D_03	435	gb|ABP02242.1|	Cyclin-like F-box [Medicago truncatula]	3E-71
CaF1_WIE_44_D_07	192	emb|AJ009878.1|CAR9878	Cicer arietinum mRNA for cysteine proteinase	1E-91
CaF1_WIE_44_F_11	262	ref|XP_381439.1|	hypothetical protein FG01263.1 [Gibberella zeae PH-1]	1E-13
CaF1_WIE_44_G_03	271	emb|CAN68337.1|	hypothetical protein [Vitis vinifera]	2E-39
CaF1_WIE_44_H_04	124	emb|AJ534345.1|ABI534345	Agaricus bisporus partial mRNA for putative glyoxylate pathway regulator (gpr gene), clone pm166	0.0005
CaF1_WIE_45_A_04	419	gb|AAF22842.1|AF209910_1	vacuolar sorting receptor protein [Prunus dulcis]	3E-50
CaF1_WIE_45_B_03	273	gb|ABE84233.1|	Arf GTPase activating protein [Medicago truncatula]	2E-13
CaF1_WIE_45_B_10	248	emb|CT573508.2|	Medicago truncatula chromosome 5 clone mth2-29p15, COMPLETE SEQUENCE	0.0000003
CaF1_WIE_45_C_07	404	gb|AY032742.1|	Fusarium sporotrichioides guanine nucleotide-binding protein mRNA, complete cds	5E-93
CaF1_WIE_45_C_09	200	gb|AAL66290.1|AF452450_1	adenosine 5'-phosphosulfate reductase [Glycine max]	1E-14
CaF1_WIE_45_E_07	338	gb|ABH02845.1|	MYB transcription factor MYB93 [Glycine max]	5E-25
CaF1_WIE_45_E_10	424	ref|NP_001077888.1|	ALDH6B2 (Aldehyde dehydrogenase 6B2) [Arabidopsis thaliana]	2E-53
CaF1_WIE_45_H_01	327	gb|EF025129.1|	Medicago truncatula PHD6 mRNA, complete cds	3E-23
CaF1_WIE_46_A_04	461	emb|CAN72806.1|	hypothetical protein [Vitis vinifera]	7E-19
CaF1_WIE_46_A_06	342	gb|ABQ11262.1|	mago nashi-like protein 1 [Physalis pubescens] gb|ABQ11264.1| mago nashi-like protein 1 [Physalis pubescens]	4E-27
CaF1_WIE_46_A_09	418	gb|AC150798.3|	Medicago truncatula chromosome 2 clone mth2-33b11, complete sequence	3E-76
CaF1_WIE_46_A_10	206	emb|AM452705.1|	Vitis vinifera, whole genome shotgun sequence, contig VV78X225485.15, clone ENTAV 115	0.000000004
CaF1_WIE_46_B_03	340	gb|ABE80335.1|	Kunitz inhibitor ST1-like [Medicago truncatula]	2E-27
CaF1_WIE_46_B_07	279	emb|CAN75568.1|	hypothetical protein [Vitis vinifera]	9E-38
CaF1_WIE_46_B_08	284	ref|NP_191085.1|	unknown protein [Arabidopsis thaliana] emb|CAB75759.1| putative protein [Arabidopsis thaliana] gb|AAM20644.1| putative protein [Arabidopsis thaliana] gb|AAM91309.1| putative protein [Arabidopsis thaliana] gb|AAO43936.1| putative calcium homeostasis regulator [Arabidopsis thaliana]	2E-19
CaF1_WIE_46_C_03	204	gb|EAZ30583.1|	hypothetical protein OsJ_014066 [Oryza sativa (japonica cultivar-group)]	5E-27
CaF1_WIE_46_C_07	392	gb|AF537102.1|	Plasmodiophora brassicae 16S ribosomal RNA gene, partial sequence; mitochondrial gene for mitochondrial product	0.00003
CaF1_WIE_46_D_04	197	gb|ABI51282.1|	actin [Hibiscus cannabinus]	5E-22
CaF1_WIE_46_D_05	462	gb|ABE92992.1|	Exostosin-like [Medicago truncatula]	6E-47
CaF1_WIE_46_D_06	425	gb|ABP03326.1|	Calcium-binding EF-hand; Ras small GTPase, Rho type [Medicago truncatula]	6E-63
CaF1_WIE_46_D_09	183	dbj|AP006094.1|	Lotus japonicus genomic DNA, chromosome 4, clone:LjT39H01, TM0172, complete sequence	0.000000003
CaF1_WIE_46_D_11	390	sp|O24301|SUS2_PEA	Sucrose synthase 2 (Sucrose-UDP glucosyltransferase 2) emb|CAA04512.1| second sucrose synthase [Pisum sativum]	1E-24
CaF1_WIE_46_E_03	147	emb|AM706411.1|	Eristalis tenax partial mRNA for hypothetical protein (ORF1), isolate 3	0.00004
CaF1_WIE_46_F_04	161	gb|AC135231.23|	Medicago truncatula clone mth2-28m10, complete sequence	5E-17
CaF1_WIE_46_F_09	448	gb|ABK55756.1|	P450 monooxygenase-like protein [Stylosanthes guianensis]	9E-51
CaF1_WIE_46_G_07	211	emb|AJ489609.1|CAR489609	Cicer arietinum mRNA for alpha-expansin 4 (expa4 gene)	2E-88
CaF1_WIE_46_G_10	378	emb|AM706411.1|	Eristalis tenax partial mRNA for hypothetical protein (ORF1), isolate 3	0.0001
CaF1_WIE_46_H_04	463	gb|ABO78866.1|	WD40-like [Medicago truncatula]	5E-16
CaF1_WIE_47_A_02	307	gb|AC148171.16|	Medicago truncatula clone mth2-30a2, complete sequence	4E-22
CaF1_WIE_47_A_05	407	gb|ABO81445.1|	Glycoside hydrolase, family 1 [Medicago truncatula]	3E-22
CaF1_WIE_47_A_06	287	gb|AY972077.1|	Synthetic construct RLS (RLS) gene, complete cds	0.075
CaF1_WIE_47_A_07	196	emb|CU137664.2|	Medicago truncatula chromosome 5 clone mth2-69n5, COMPLETE SEQUENCE	2E-20
CaF1_WIE_47_A_08	357	emb|CAN63025.1|	hypothetical protein [Vitis vinifera]	4E-16
CaF1_WIE_47_A_11	423	gb|ABE78454.1|	Phosphoesterase, DHHA1 [Medicago truncatula]	3E-55
CaF1_WIE_47_B_06	151	emb|AL392048.9|	Human DNA sequence from clone RP11-305D15 on chromosome 13 Contains the 5' end of the RB1 gene for retinoblastoma 1 (including osteosarcoma), two novel genes, the gene for purinergic receptor (family A group 5) (P2Y5), a PEST-containing nuclear protein (P	2.3
CaF1_WIE_47_B_07	204	gb|AC152818.17|	Medicago truncatula clone mth2-85g12, complete sequence	6E-20
CaF1_WIE_47_B_08	462	gb|ABO83980.1|	Thioredoxin domain 2; Thioredoxin fold [Medicago truncatula]	8E-39
CaF1_WIE_47_C_02	462	emb|CAN66493.1|	hypothetical protein [Vitis vinifera]	2E-20
CaF1_WIE_47_C_03	453	gb|ABE93337.1|	Protein kinase [Medicago truncatula]	7E-77
CaF1_WIE_47_C_04	360	gb|ABE82410.1|	SBP [Medicago truncatula] gb|ABE92194.1| SBP [Medicago truncatula]	2E-41

CaF1_WIE_47_C_09	484	emb|CAN75662.1|	hypothetical protein [Vitis vinifera]	3E-34
CaF1_WIE_47_C_11	407	emb|CAN65403.1|	hypothetical protein [Vitis vinifera]	3E-33
CaF1_WIE_47_D_01	365	gb|ABP03277.1|	UBX; PUG; Zinc finger, C2H2-type [Medicago truncatula]	1E-35
CaF1_WIE_47_D_03	461	gb|AAY22204.1|	putative aquaporin [Phaseolus vulgaris]	5E-54
CaF1_WIE_47_D_07	460	ref|NP_199656.1|	SKS3 (SKU5 Similar 3); copper ion binding [Arabidopsis thaliana]	5E-19
CaF1_WIE_47_D_08	164	emb|AM706411.1|	Eristalis tenax partial mRNA for hypothetical protein (ORF1), isolate 3	0.00004
CaF1_WIE_47_E_01	113	emb|AJ608703.3|	Fusarium oxysporum f. sp. lycopersici six1 gene, fot5 gene, six2 gene, shh1 gene and ORF2 (partial)	5E-19
CaF1_WIE_47_E_02	155	gb|DQ465754.1|	Sesbania drummondii clone SSH-1_01_F12_T3 mRNA sequence	0.00001
CaF1_WIE_47_E_07	462	gb|AC149579.11|	Medicago truncatula clone mth2-99d8, complete sequence	2E-25
CaF1_WIE_47_F_03	444	ref|NP_174675.2|	STT3B (STAUROSPORIN AND TEMPERATURE SENSITIVE 3-LIKE B); oligosaccharyl transferase [Arabidopsis thaliana] gb|AAG12524.1|AC015446_5 Putative integral membrane protein [Arabidopsis thaliana]	3E-74
CaF1_WIE_47_H_01	140	ref|XM_001349534.1|	Plasmodium falciparum 3D7 DNA repair endonuclease, putative (PFB0265c) mRNA, complete cds	0.13
CaF1_WIE_47_H_03	462	gb|ABE85038.2|	Peptidase S1 and S6, chymotrypsin/Hap; Immunoglobulin/major histocompatibility complex; AAA ATPase, central region; SMAD/FHA [Medicago truncatula]	3E-30
CaF1_WIE_47_H_06	329	emb|CT029405.1|	Poplar cDNA sequences	0.022
CaF1_WIE_48_A_04	182	ref|NP_196983.1|	binding [Arabidopsis thaliana] emb|CAC01878.1| putative protein [Arabidopsis thaliana] gb|AAM13237.1| putative protein [Arabidopsis thaliana] gb|AAM67181.1| unknown [Arabidopsis thaliana] gb|AAN65128.1| putative protein [Arabidopsis thaliana] dbj|BAD93942.1| hypothetical protein [Arabidopsis thaliana]	1E-19
CaF1_WIE_48_B_10	341	gb|ABE93939.2|	Nuclear pore complex protein Nup205 , related [Medicago truncatula]	7E-34
CaF1_WIE_48_B_11	377	ref|XP_382956.1|	conserved hypothetical protein [Gibberella zeae PH-1]	3E-36
CaF1_WIE_48_C_01	159	emb|CT029389.1|	Poplar cDNA sequences	0.039
CaF1_WIE_48_C_04	208	dbj|BAA36291.1|	HMG-CoA reductase [Cucumis melo]	3E-22
CaF1_WIE_48_C_05	384	emb|CR931811.1|	Medicago truncatula chromosome 5 clone mte1-56a16, COMPLETE SEQUENCE	0.00000003
CaF1_WIE_48_D_02	208	dbj|BAA36291.1|	HMG-CoA reductase [Cucumis melo]	6E-21
CaF1_WIE_48_D_03	256	emb|CT028615.1|	Poplar cDNA sequences	0.00002
CaF1_WIE_48_D_08	195	emb|AJ749797.1|	Photobacterium damselae subsp. piscicida trpB gene for putative transposase, clone pRDA16	0.0002
CaF1_WIE_48_D_09	199	gb|DQ251457.1|	Siniperca chuatsi transposase mRNA, partial cds	0.00001
CaF1_WIE_48_D_10	223	gb|AC148237.7|	Medicago truncatula clone mth2-1o14, complete sequence	2E-29
CaF1_WIE_48_E_02	424	dbj|AK224696.2|	Solanum lycopersicum cDNA, clone: FC08DG08, HTC in fruit	0.0000005
CaF1_WIE_48_E_03	301	gb|DQ251457.1|	Siniperca chuatsi transposase mRNA, partial cds	0.00002
CaF1_WIE_48_E_04	235	emb|CAI48073.1|	60S ribosomal protein L37a [Capsicum chinense]	4E-27
CaF1_WIE_48_E_07	263	emb|CAN77483.1|	hypothetical protein [Vitis vinifera]	2E-30
CaF1_WIE_48_E_08	420	sp|Q8SAG3|ADF_VITVI	Actin-depolymerizing factor (ADF) gb|AAL79826.1|AF440310_1 actin depolymerizing factor [Vitis vinifera]	5E-46
CaF1_WIE_48_F_01	444	gb|EAZ32228.1|	hypothetical protein OsJ_015711 [Oryza sativa (japonica cultivar-group)]	2E-19
CaF1_WIE_48_F_04	325	emb|AM231535.1|	Photorhabdus asymbiotica subsp. asymbiotica partial tra for ArsR family/rhodanese-like protein, clone 83	0.086
CaF1_WIE_48_F_05	383	gb|ABD32921.2|	F5O11.19, related [Medicago truncatula]	7E-61
CaF1_WIE_48_F_09	456	gb|ABE90758.1|	Annexin [Medicago truncatula]	3E-56
CaF1_WIE_48_F_10	219	gb|ABL98074.1|	chitinase-related agglutinin [Robinia pseudoacacia]	7E-22
CaF1_WIE_48_G_01	256	emb|AM697674.1|	Platynereis dumerilii mRNA for hypothetical protein (ORF1), isolate 2	0.001
CaF1_WIE_48_G_04	143	gb|AC174142.11|	Medicago truncatula clone mth2-69j4, complete sequence	0.000000009
CaF1_WIE_48_H_01	104	emb|AJ249801.1|CAR249801	Cicer arietinum partial mRNA for cytochrome P450 monooxygenase (cyp81E4 gene)	3E-17
CaF1_WIE_48_H_02	485	emb|CAN65408.1|	hypothetical protein [Vitis vinifera]	3E-39
CaF1_WIE_48_H_11	459	gb|ABE89510.1|	U2 auxiliary factor small subunit [Medicago truncatula]	1E-72
CaF1_WIE_49_A_02	219	emb|AJ293849.1|KPN293849	Klebsiella pneumoniae contig region pSL029	0.00006
CaF1_WIE_49_A_06	444	emb|AJ749797.1|	Photobacterium damselae subsp. piscicida trpB gene for putative transposase, clone pRDA16	0.0005
CaF1_WIE_49_A_10	456	gb|AF461200.1|	Medicago truncatula nodule-enhanced malate dehydrogenase gene, exons 1 and 2 and partial cds	2E-46
CaF1_WIE_49_B_02	320	emb|CT954252.6|	M.truncatula DNA sequence from clone MTH2-60M21 on chromosome 3, complete sequence	1E-46
CaF1_WIE_49_B_04	208	sp|Q9MAV7|RL31_PANGI	60S ribosomal protein L31 dbj|BAA96368.1| ribosomal protein L31 [Panax ginseng]	4E-24
CaF1_WIE_49_B_05	421	gb|ABG73621.1|	leucine-rich repeat receptor-like kinase [Populus tomentosa]	5E-45

CaF1_WIE_49_B_06	486	gb|EAZ03842.1|	hypothetical protein OsI_025074 [Oryza sativa (indica cultivar-group)] gb|EAZ39784.1| hypothetical protein OsJ_023267 [Oryza sativa (japonica cultivar-group)]	3E-38
CaF1_WIE_49_B_08	408	emb|CAA09588.1|	phosphoenolpyruvate-carboxylase [Vicia faba]	6E-65
CaF1_WIE_49_B_11	218	dbj|AP006667.1|	Lotus japonicus genomic DNA, chromosome 5, clone:LjT31L14, TM0366, complete sequence	0.00000002
CaF1_WIE_49_C_02	361	ref|NP_173826.1|	unknown protein [Arabidopsis thaliana] gb|AAC00576.1| Unknown protein [Arabidopsis thaliana]	2E-17
CaF1_WIE_49_C_04	485	gb|EAZ11192.1|	hypothetical protein OsJ_001017 [Oryza sativa (japonica cultivar-group)]	3E-20
CaF1_WIE_49_C_09	272	gb|ABE91004.1|	Zinc finger, CCCH-type; Zinc finger, RING-type [Medicago truncatula]	2E-31
CaF1_WIE_49_D_04	266	ref|NP_851260.1|	ATHDH (HISTIDINOL DEHYDROGENASE) [Arabidopsis thaliana]	1E-14
CaF1_WIE_49_D_06	424	emb|CR962137.2|	Medicago truncatula chromosome 5 clone mte1-10p15, COMPLETE SEQUENCE	5E-41
CaF1_WIE_49_D_07	289	gb|ABE93358.1|	Protein of unknown function DUF630 [Medicago truncatula]	6E-13
CaF1_WIE_49_D_09	123	gb|DQ465754.1|	Sesbania drummondii clone SSH-1_01_F12_T3 mRNA sequence	0.0001
CaF1_WIE_49_D_11	172	ref|NP_178980.1|	malate oxidoreductase, putative [Arabidopsis thaliana] gb|AAD22679.1| malate oxidoreductase (malic enzyme) [Arabidopsis thaliana] gb|AAM14058.1| putative malate oxidoreductase (malic enzyme) [Arabidopsis thaliana] gb|AAN41396.1| putative malate oxidoreductase (malic enzyme) [Arabidopsis thaliana]	7E-21
CaF1_WIE_49_F_03	280	sp|Q9SXU1|PSA7_CICAR	Proteasome subunit alpha type 7 (20S proteasome alpha subunit D) (20S proteasome subunit alpha-4) dbj|BAA76428.1| multicatalytic endopeptidase complex [Cicer arietinum]	5E-39
CaF1_WIE_49_F_10	448	emb|CAN65965.1|	hypothetical protein [Vitis vinifera]	6E-61
CaF1_WIE_49_G_02	422	ref|XM_382053.1|	Gibberella zeae PH-1 chromosome 1 hypothetical protein (FG01877.1) partial mRNA	1E-10
CaF1_WIE_49_G_05	457	gb|AC144340.30|	Medicago truncatula clone mth2-7k2, complete sequence	3E-30
CaF1_WIE_49_G_08	153	emb|AJ276466.1|KPN276466	Klebsiella pneumoniae contig region pSL004	0.00004
CaF1_WIE_49_G_11	426	emb|AM447853.2|	Vitis vinifera contig VV78X083454.9, whole genome shotgun sequence	0.0005
CaF1_WIE_49_H_04	185	emb|AM425323.1|	Vitis vinifera contig VV78X242376.24, whole genome shotgun sequence	1E-11
CaF1_WIE_49_H_08	223	dbj|AP004534.1|	Lotus japonicus genomic DNA, chromosome 4, clone:LjT14P20, TM0087, complete sequence	5E-27
CaF1_WIE_50_B_03	454	gb|AAO49473.1|	putative serine/threonine kinase [Vitis vinifera]	5E-11
CaF1_WIE_50_B_08	163	gb|ABG90381.1|	glutathione S-transferase [Caragana korshinskii]	4E-13
CaF1_WIE_50_C_09	460	gb|ABE87035.1|	Orn/DAP/Arg decarboxylase 2; Protease-associated PA; Proteinase inhibitor I9, subtilisin propeptide [Medicago truncatula]	8E-73
CaF1_WIE_50_D_06	461	emb|AM706411.1|	Eristalis tenax partial mRNA for hypothetical protein (ORF1), isolate 3	0.0001
CaF1_WIE_50_D_11	384	sp|P52904|ODPB_PEA	Pyruvate dehydrogenase E1 component subunit beta, mitochondrial precursor (PDHE1-B) gb|AAB01223.1| pyruvate dehydrogenase E1beta	9E-56
CaF1_WIE_50_E_02	392	ref|XP_364191.1|	hypothetical protein MGG_09036 [Magnaporthe grisea 70-15] ref|XP_001522086.1| hypothetical protein MGCH7_ch7g203 [Magnaporthe grisea 70-15] gb|EAQ70796.1| hypothetical protein MGCH7_ch7g203 [Magnaporthe grisea 70-15] gb|EDK02087.1| hypothetical protein MGG_09036 [Magnaporthe grisea 70-15]	1E-25
CaF1_WIE_50_E_03	344	gb|AAS47511.1|	ribosomal protein S6 [Glycine max]	1E-30
CaF1_WIE_50_E_05	385	emb|CAC67501.1|	selenium binding protein [Medicago sativa]	1E-61
CaF1_WIE_50_E_10	448	gb|ABP65665.1|	VTC2-like protein [Actinidia chinensis]	1E-59
CaF1_WIE_50_G_05	452	gb|ABO84404.1|	SAE2 , putative [Medicago truncatula]	2E-47
CaF1_WIE_50_G_06	432	ref|XP_001267009.1|	calmodulin [Neosartorya fischeri NRRL 181] gb|EAW25112.1| calmodulin [Neosartorya fischeri NRRL 181]	1E-16
CaF1_WIE_50_G_07	460	gb|ABE92424.1|	hypothetical protein MtrDRAFT_AC137986g22v2 [Medicago truncatula] gb|ABN08461.1| hypothetical protein MtrDRAFT_AC157472g30v2 [Medicago truncatula]	8E-15
CaF1_WIE_51_A_02	451	gb|ABE91787.1|	Protein of unknown function DUF707 [Medicago truncatula]	2E-61
CaF1_WIE_51_A_06	457	emb|CAN60708.1|	hypothetical protein [Vitis vinifera]	8E-20
CaF1_WIE_51_A_10	213	gb|BT009458.1|	Triticum aestivum clone wlsu2.pk0001.h3:fis, full insert mRNA sequence	0.00006
CaF1_WIE_51_B_08	455	sp|Q39366|LGUL_BRAOG	Putative lactoylglutathione lyase (Methylglyoxalase) (Aldoketomutase) (Glyoxalase I) (Glx I) (Ketone-aldehyde mutase) (S-D-lactoylglutathione methylglyoxal lyase) emb|CAA99248.1| unknown [Brassica oleracea var. gemmifera]	4E-27
CaF1_WIE_51_B_10	270	emb|CAA63598.1|	glyoxysomal beta-ketoacyl-thiolase [Brassica napus]	4E-31
CaF1_WIE_51_C_04	448	dbj|BAE71253.1|	hypothetical protein [Trifolium pratense]	
CaF1_WIE_51_C_06	360	gb|DQ117568.1|	Phaseolus vulgaris clone PvD34, mRNA sequence	3E-29

CaF1_WIE_51_C_09	235	sp|Q945F4|IF5A2_MEDSA	Eukaryotic translation initiation factor 5A-2 (eIF-5A-2) gb|AAL10404.1|AF416338_1 eukaryotic translation initiation factor 5A-2 [Medicago sativa] gb|ABE85424.1| NusG [Medicago truncatula] gb|ABE88714.1| NusG [Medicago truncatula]	5E-30
CaF1_WIE_51_C_10	155	dbj|AP006431.1|	Lotus japonicus genomic DNA, chromosome 5, clone:LjT38F20, TM0328, complete sequence	0.0006
CaF1_WIE_51_C_11	454	gb|ABE78903.1|	Protein kinase [Medicago truncatula] gb|ABE81974.1| Protein kinase [Medicago truncatula]	7E-64
CaF1_WIE_51_D_06	298	gb|AAB71830.1|	annexin [Lavatera thuringiaca]	6E-26
CaF1_WIE_51_D_09	269	emb|AJ534347.1|ABI534347	Agaricus bisporus partial mRNA for putative hexose transporter protein (htp gene)	0.28
CaF1_WIE_51_E_07	368	gb|ABD32889.1|	AAA ATPase; 26S proteasome subunit P45 [Medicago truncatula] gb|ABN08912.1| AAA ATPase; 26S proteasome subunit P45 [Medicago truncatula]	8E-57
CaF1_WIE_51_F_04	462	gb|ABN08184.1|	General substrate transporter [Medicago truncatula] gb|ABN09010.1| General substrate transporter [Medicago truncatula]	2E-65
CaF1_WIE_51_F_07	414	gb|ABM91070.1|	xyloglucan endotransglycosylase/hydrolase precursor XTH-3 [Populus tremula x Populus tremuloides]	1E-44
CaF1_WIE_51_G_06	379	gb|AY461597.1|	Synthetic construct arsenic-like protein gene, complete cds	0.0001
CaF1_WIE_51_H_05	220	gb|AF071889.1|AF071889	Prunus armeniaca 40S ribosomal protein S8 (RPS8) mRNA, complete cds	2E-29
CaF1_WIE_52_A_07	334	gb|AAP80667.1|AF479048_1	ribosomal Pr 117 [Triticum aestivum]	5E-49
CaF1_WIE_52_A_11	459	dbj|AP006629.1|	Lotus japonicus genomic DNA, chromosome 2, clone:LjT10B11, TM0008, complete sequence	7E-34
CaF1_WIE_52_B_01	488	gb|ABP02258.1|	Glycoside hydrolase, family 3, N-terminal; Glycoside hydrolase, family 3, C-terminal [Medicago truncatula]	5E-55
CaF1_WIE_52_B_07	369	gb|AF397032.2|	Pisum sativum clone PsEXT3.28 root nodule extensin mRNA, partial cds	1E-25
CaF1_WIE_52_B_09	458	gb|ABD32628.1|	Granulin; Peptidase C1A, papain [Medicago truncatula]	3E-76
CaF1_WIE_52_C_05	475	gb|ABP03389.1|	20S proteasome, A and B subunits [Medicago truncatula]	4E-74
CaF1_WIE_52_C_06	460	gb|ABE89416.1|	TGS; Small GTP-binding protein domain [Medicago truncatula]	4E-79
CaF1_WIE_52_E_05	401	ref|NP_181843.2|	DNA binding / transcription factor [Arabidopsis thaliana]	4E-23
CaF1_WIE_52_F_01	454	gb|EAZ36326.1|	hypothetical protein OsJ_019809 [Oryza sativa (japonica cultivar-group)]	3E-13
CaF1_WIE_52_F_05	448	emb|CR940305.16|	M.truncatula DNA sequence from clone MTH2-28N4 on chromosome 3, complete sequence	5E-41
CaF1_WIE_52_G_04	356	ref|NP_197500.1|	RPT6A (regulatory particle triple-A 6A); ATPase [Arabidopsis thaliana] gb|AAK64142.1| putative 26S proteasome AAA-ATPase subunit RPT6a [Arabidopsis thaliana] gb|AAL85134.1| putative 26S proteasome AAA-ATPase subunit RPT6a [Arabidopsis thaliana] gb|AAM65046.1| 26S proteasome AAA-ATPase subunit RPT6a-like protein [Arabidopsis thaliana] dbj|BAE98371.1| 26S proteasome AAA-ATPase subunit RPT6a - like protein [Arabidopsis thaliana]	5E-47
CaF1_WIE_53_A_01	336	gb|ABR15094.1|	ATP citrate lyase alpha subunit [Glycyrrhiza uralensis]	3E-29
CaF1_WIE_53_A_02	462	gb|AAG29593.1|AF196286_1	Ser/Thr specific protein phosphatase 2A A regulatory subunit alpha isoform [Medicago sativa subsp. x varia]	3E-39
CaF1_WIE_53_A_10	384	sp|O65731|RS5_CICAR	40S ribosomal protein S5 emb|CAA06491.1| 40S ribosomal protein S5 [Cicer arietinum]	5E-19
CaF1_WIE_53_B_09	429	dbj|BAF34844.1|	pterocarpan reductase [Lotus japonicus]	5E-60
CaF1_WIE_53_C_10	454	sp|P31239|ACCO_PEA	1-aminocyclopropane-1-carboxylate oxidase (ACC oxidase) (Ethylene-forming enzyme) (EFE) gb|AAA33644.1| 1-aminocyclopropane-1-carboxylate oxidase	3E-18
CaF1_WIE_53_D_06	449	gb|AAM61146.1|	unknown [Arabidopsis thaliana]	1E-49
CaF1_WIE_53_D_07	455	ref|NP_001077535.1|	UBX domain-containing protein [Arabidopsis thaliana] ref|NP_001077536.1| UBX domain-containing protein [Arabidopsis thaliana]	5E-27
CaF1_WIE_53_E_03	416	dbj|AB049721.2|	Pisum sativum ssa-11 mRNA for putative senescence-associated protein, complete cds	5E-78
CaF1_WIE_53_E_05	458	gb|ABF59516.1|	putative spindle disassembly related protein CDC48 [Nicotiana tabacum]	4E-77
CaF1_WIE_53_F_03	455	gb|ABG90380.1|	bZIP transcription factor [Caragana korshinskii]	4E-46
CaF1_WIE_53_G_02	456	sp|P55844|RL14_PEA	Probable 60 ribosomal protein L14 (Hydroxyproline-rich glycoprotein HRGP1)	9E-56
CaF1_WIE_53_G_06	461	ref|XP_386883.1|	hypothetical protein FG06707.1 [Gibberella zeae PH-1]	3E-54
CaF1_WIE_53_H_05	459	gb|ABE84183.2|	Protein kinase [Medicago truncatula]	4E-74
CaF1_WIE_53_H_09	455	gb|EAY91540.1|	hypothetical protein OsI_012773 [Oryza sativa (indica cultivar-group)]	1E-11

CaF1_WIE_54_A_01	330	dbj|BAB32793.1|	110 kDa 4SNc-Tudor domain protein [Pisum sativum]	6E-44
CaF1_WIE_54_A_05	424	gb|AAG31076.1|AF283566_1	sucrose-phosphatase [Medicago truncatula]	1E-20
CaF1_WIE_54_B_01	387	emb|CAN76851.1|	hypothetical protein [Vitis vinifera]	6E-34
CaF1_WIE_54_B_02	200	emb|CAA10290.1|	ribulose 1,5-bisphosphate carboxylase small subunit [Cicer arietinum]	7E-27
CaF1_WIE_54_B_06	459	emb|CAB36704.1|	putative protein [Arabidopsis thaliana] emb|CAB80144.1| putative protein [Arabidopsis thaliana]	7E-50
CaF1_WIE_54_C_09	424	ref|NP_568217.1|	BolA-like family protein [Arabidopsis thaliana] dbj|BAB09404.1| unnamed protein product [Arabidopsis thaliana] gb|AAM65194.1| unknown [Arabidopsis thaliana] gb|AAO24583.1| At5g09830 [Arabidopsis thaliana] dbj|BAF00125.1| hypothetical protein [Arabidopsis thaliana]	6E-30
CaF1_WIE_54_C_10	307	emb|CAJ15149.1|	sialyltransferase-like protein [Medicago truncatula] gb|ABE93190.1| Glycosyl transferase, family 29; Immunoglobulin/major histocompatibility complex [Medicago truncatula]	2E-40
CaF1_WIE_54_C_11	246	emb|CAN74631.1|	hypothetical protein [Vitis vinifera]	1E-19
CaF1_WIE_54_D_03	450	dbj|BAB86847.1|	elongation factor EF-2 [Pisum sativum]	2E-62
CaF1_WIE_54_D_07	430	gb|ABE81376.1|	Cell division protein FtsZ [Medicago truncatula]	6E-69
CaF1_WIE_54_D_10	457	emb|AM697674.1|	Platynereis dumerilii mRNA for hypothetical protein (ORF1), isolate 2	0.000008
CaF1_WIE_54_D_11	222	gb|AY972077.1|	Synthetic construct RLS (RLS) gene, complete cds	0.001
CaF1_WIE_54_E_01	353	emb|CAA65982.1|	cdc2MsF [Medicago sativa]	4E-20
CaF1_WIE_54_E_05	305	sp|O24301|SUS2_PEA	Sucrose synthase 2 (Sucrose-UDP glucosyltransferase 2) emb|CAA04512.1| second sucrose synthase [Pisum sativum]	5E-43
CaF1_WIE_54_E_06	110	gb|AY972077.1|	Synthetic construct RLS (RLS) gene, complete cds	0.0001
CaF1_WIE_54_E_07	244	emb|CAD31718.1|	putative cytochrome P450 monooxygenase [Cicer arietinum]	7E-13
CaF1_WIE_54_E_09	216	emb|AM422122.1|	Danio rerio tcamp mRNA, 3' UTR	0.00001
CaF1_WIE_54_F_05	271	emb|CAJ31277.1|	autophagy protein 5 [Glycine max]	3E-15
CaF1_WIE_54_F_06	118	emb|AM697674.1|	Platynereis dumerilii mRNA for hypothetical protein (ORF1), isolate 2	0.0004
CaF1_WIE_54_F_07	282	gb|AAM65872.1|	ferritin subunit, putative [Arabidopsis thaliana]	3E-15
CaF1_WIE_54_G_08	427	dbj|BAD94926.1|	hypothetical protein [Arabidopsis thaliana]	1E-17
CaF1_WIE_55_B_04	423	emb|CR962124.2|	Medicago truncatula chromosome 5 clone mth2-44c15, COMPLETE SEQUENCE	2E-43
CaF1_WIE_55_B_09	422	gb|AAP22955.1|	Potyvirus VPg interacting protein [Pisum sativum]	4E-62
CaF1_WIE_55_C_01	337	gb|AF520576.1|	Glycine max extensin-like protein gene, promoter region and complete cds	0.000001
CaF1_WIE_55_C_03	390	gb|EAZ03281.1|	hypothetical protein OsI_024513 [Oryza sativa (indica cultivar-group)]	6E-29
CaF1_WIE_55_C_06	436	emb|CU019601.8|	M.truncatula DNA sequence from clone MTH2-12A22 on chromosome 3, complete sequence	1E-16
CaF1_WIE_55_C_08	295	gb|AY972077.1|	Synthetic construct RLS (RLS) gene, complete cds	0.0003
CaF1_WIE_55_D_03	290	gb|ABE78927.1|	Peptidase T1A, proteasome beta-subunit [Medicago truncatula]	4E-45
CaF1_WIE_55_E_03	302	gb|AY972077.1|	Synthetic construct RLS (RLS) gene, complete cds	0.0003
CaF1_WIE_55_E_04	421	ref|XM_415370.2|	PREDICTED: Gallus gallus similar to class I alpha chain (LOC417083), mRNA	0.000000008
CaF1_WIE_55_E_09	458	emb|CAN74784.1|	hypothetical protein [Vitis vinifera]	4E-45
CaF1_WIE_55_E_10	295	gb|DQ073809.1|	Trifolium pratense phenylalanine ammonia lyase (PAL1) mRNA, complete cds	9E-11
CaF1_WIE_55_F_07	302	gb|ABN08775.1|	Glycoside hydrolase, family 19 [Medicago truncatula]	2E-30
CaF1_WIE_55_G_01	368	gb|ABP02712.1|	14-3-3 protein [Medicago truncatula]	1E-53
CaF1_WIE_55_G_06	395	gb|ABE90826.1|	von Willebrand factor, type C; Heavy metal transport/detoxification protein [Medicago truncatula]	7E-29
CaF1_WIE_55_G_09	456	emb|CAJ38375.1|	nucleoside-diphopshate-sugar dehydratase [Plantago major]	4E-60
CaF1_WIE_55_H_01	345	gb|AY972077.1|	Synthetic construct RLS (RLS) gene, complete cds	0.023
CaF1_WIE_55_H_06	433	gb|AY273895.1|	Brugia malayi transglutaminase mRNA, partial cds	0.000008
CaF1_WIE_55_H_09	429	gb|ABO81708.1|	2-oxoglutarate dehydrogenase, E1 component [Medicago truncatula]	2E-68
CaF1_WIE_56_A_10	423	emb|CAN61192.1|	hypothetical protein [Vitis vinifera]	3E-38
CaF1_WIE_56_B_03	405	ref|NP_001046176.1|	Os02g0194200 [Oryza sativa (japonica cultivar-group)] dbj|BAD15406.1| KH domain-containing protein-like [Oryza sativa (japonica cultivar-group)] dbj|BAF08090.1| Os02g0194200 [Oryza sativa (japonica cultivar-group)] gb|EAY84850.1| hypothetical protein OsI_006083 [Oryza sativa (indica cultivar-group)] gb|EAZ22072.1| hypothetical protein OsJ_005555 [Oryza sativa (japonica cultivar-group)]	7E-27

CaF1_WIE_56_D_10	308	ref|NP_001058575.1|	Os06g0714500 [Oryza sativa (japonica cultivar-group)] dbj|BAD53565.1| putative spastin protein [Oryza sativa (japonica cultivar-group)] dbj|BAF20489.1| Os06g0714500 [Oryza sativa (japonica cultivar-group)] gb|EAZ02349.1| hypothetical protein OsI_023581 [Oryza sativa (indica cultivar-group)] gb|EAZ38273.1| hypothetical protein OsJ_021756 [Oryza sativa (japonica cultivar-group)]	8E-44
CaF1_WIE_56_D_11	455	gb|ABE82917.1|	Aldo/keto reductase [Medicago truncatula]	3E-67
CaF1_WIE_56_E_08	424	gb|AAC32158.1|	hypothetical protein [Picea mariana]	1E-13
CaF1_WIE_56_F_04	422	dbj|BAE71253.1|	hypothetical protein [Trifolium pratense]	6E-42
CaF1_WIE_56_F_05	425	gb|ABE86452.1|	Aminotransferase, class I and II [Medicago truncatula] gb|ABE87161.1| 1-aminocyclopropane-1-carboxylate synthase [Medicago truncatula]	6E-65
CaF1_WIE_56_G_06	405	gb|ABI34092.1|	cystathionine gamma-synthase [Medicago sativa]	5E-59
CaF1_WIE_56_G_07	396	emb|CAC67501.1|	selenium binding protein [Medicago sativa]	7E-30
CaF1_WIE_56_H_11	283	gb|AAD47346.1|AF112440_1	ribosomal protein S26 [Pisum sativum]	2E-23

a. In the clone ID, the first two letters (Ca) signify the source plant, *Cicer arietinum* , the third letter (F) designates the pathogen name, *Fusarium* , and the numeral 1 designates race 1 of *Fusarium* . In the three letter abbreviation JIE and WIE, the first letter J and W refers to the JG-62 and WR-315 genotypes of chickpea, followed by (IE) signifying *Fusarium* induced library and the early time points taken for the tissue collection. For additional details, see Materials and Methods.

b. Accession number shown refers to the NCBI database.

c. The possible function of the chickpea contigs was assigned by performing BLASTX and BLASTN against nonredundant protein and nucleotide database in NCBI.

d. For BLASTX the E-value cutoff used was 10^{-15} and for BLASTN the cutoff used wad 10^{-20}
