# Supplementary material for: Comparative analyses of genotype dependent expressed sequence tags and stress-responsive transcriptome of chickpea wilt illustrate predicted and unexpected genes and novel regulators of plant immunity
Source: BMC Genomics. 2009 Sep 5;10:415. doi: 10.1186/1471-2164-10-415 (PMC2755012; doi:10.1186/1471-2164-10-415)
Supplement: Additional file 3 — Functional class annotation of CaUnigenes. Table listing functional annotation and assignment of CaUnigenes into different functional classes using BLAST, GO, MetaCyc and COG. [file 1471-2164-10-415-S3.pdf]

Additional file 3 - Functional annotation and assignment of *Ca* Unigenes into different functional classes.

Functional class	Contig/Clone ID[a]	Accession number[b]	Functional annotation[c]	E-value[d]
Cell cycle control and cell division	Contig965	gb|ABF66654.1|	EBP1 [Ammopiptanthus mongolicus]	1E-100
	Contig876	gb|ABO84368.1|	Cell division protein FtsZ [Medicago truncatula]	7E-85
	CaF1_WIE_54_D_07	gb|ABE81376.1|	Cell division protein FtsZ [Medicago truncatula]	6E-69
	Contig733	gb|ABO84368.1|	Cell division protein FtsZ [Medicago truncatula]	3E-66
	Contig282	emb|CAB87834.1|	putative kinetochore protein [Vicia faba var. minor]	8E-57
	Contig895	gb|AAO72990.1|	cyclin D [Populus alba]	9E-56
	Contig494	gb|ABE90057.1|	Cell division protein FtsZ [Medicago truncatula]	3E-48
	CaF1_WIE_11_B_09	gb|AAC49183.1|	SDL5A	2E-46
	CaF1_WIE_20_E_02	gb|ABO84368.1|	Cell division protein FtsZ [Medicago truncatula]	4E-41
	Contig281	gb|ABO81258.1|	Targeting for Xklp2 [Medicago truncatula]	2E-25
	CaF1_JIE_05_B_01	gb|AAB05992.1|	SDL	2E-25
	CaF1_WIE_54_E_01	emb|CAA65982.1|	cdc2MsF [Medicago sativa]	4E-20
Cellular redox state	Contig275	emb|CAD31838.1|	putative quinone oxidoreductase [Cicer arietinum]	3E-67
	Contig868	gb|ABC59084.1|	cytochrome P450 monooxygenase CYP83G1 [Medicago truncatula]	2E-15
	CaF1_WIE_18_D_06	gb|ABN08458.1|	Cytochrome b561 / ferric reductase transmembrane [Medicago truncatula]	1E-14
	CaF1_WIE_50_B_08	gb|ABG90381.1|	glutathione S-transferase [Caragana korshinskii]	4E-13
	CaF1_WIE_33_H_06	sp|P25317|GSTXA_TOBAC	Probable glutathione S-transferase parA (Auxin-regulated protein parA) (STR246C protein) gb|AAA67894.1| par peptide emb|CAA56790.1| STR246C [Nicotiana tabacum]	8E-12
	CaF1_WIE_11_C_02	gb|ABR04092.1|	cytochrome b5 [Malus x domestica]	4E-11
	Contig830	emb|AJ487465.1|CAR487465	Cicer arietinum mRNA for putative quinone oxidoreductase (qor gene)	1E-119
	Contig613	emb|CAA10067.1|	cytochrome P450 [Cicer arietinum]	1E-98
	Contig837	gb|AAY26520.1|	secretory peroxidase [Catharanthus roseus]	3E-80
	CaF1_JIE_03_G_05	gb|AAW78864.1|	respiratory burst oxidase 2 [Medicago truncatula] gb|ABN08032.1| Calcium binding EF-hand; Ferric reductase-like transmembrane component [Medicago truncatula]	8E-79
	Contig315	emb|CAB50768.1|	cytochrome P450 [Cicer arietinum]	2E-78
	Contig854	emb|CAA10132.1|	superoxide dismutase [Cicer arietinum] emb|CAA10160.1| superoxide dismutase [Cicer arietinum]	4E-76
	CaF1_JIE_24_F_04	emb|AJ243804.1|CAR243804	Cicer arietinum mRNA for cytochrome P450 (cyp93C3 gene)	9E-75
	CaF1_WIE_30_F_11	gb|ABO82384.1|	Cytochrome b5; Armadillo-like helical [Medicago truncatula]	1E-72
	Contig190	emb|CAB50768.1|	cytochrome P450 [Cicer arietinum]	2E-71
	CaF1_JIE_23_C_09	emb|CAB41490.1|	cytochrome P450 monooxygenase [Cicer arietinum]	6E-71
	CaF1_JIE_03_E_09	emb|CAA62226.1|	peroxidase1B [Medicago sativa]	1E-68
	Contig710	gb|ABE89853.1|	Thioredoxin-related; Thioredoxin domain 2 [Medicago truncatula]	2E-62
	Contig917	gb|ABE92135.1|	Glutathione S-transferase, C-terminal-like; Thioredoxin fold [Medicago truncatula]	3E-61
	Contig278	gb|ABE77501.1|	Glutathione S-transferase, C-terminal-like; Thioredoxin fold [Medicago truncatula]	5E-60
	CaF1_JIE_40_E_05	dbj|BAD97435.1|	peroxidase [Pisum sativum]	1E-58
	Contig674	gb|AAD37376.1|AF145350_1	peroxidase [Glycine max]	3E-58
	Contig909	gb|ABC59078.1|	cytochrome P450 monooxygenase CYP72A59 [Medicago truncatula]	3E-57
	CaF1_WIE_33_E_02	ref|NP_186958.1|	NADP-dependent oxidoreductase, putative [Arabidopsis thaliana] gb|AAF26116.1|AC012328_19 putative NADP-dependent oxidoreductase [Arabidopsis thaliana]	5E-56
	CaF1_WIE_28_F_11	gb|ABE81122.1|	Cytochrome c, monohaem [Medicago truncatula] gb|ABO84810.1| Cytochrome c, monohaem [Medicago truncatula]	8E-55
	CaF1_WIE_24_D_04	gb|AAV69019.1|	NADH:cytochrome b5 reductase [Vernicia fordii] gb|AAV69021.1| NADH:cytochrome b5 reductase [Vernicia fordii]	4E-54
	Contig104	gb|ABC68399.1|	cytochrome P450 monooxygenase CYP76O2 [Glycine max]	3E-53
	Contig143	dbj|BAC81649.1|	glutathione S-transferase [Pisum sativum]	1E-52
	Contig594	gb|AAZ32865.1|	thioredoxin h [Medicago sativa]	7E-52
	Contig486	emb|CAA10067.1|	cytochrome P450 [Cicer arietinum]	1E-51
	Contig625	gb|ABH10138.1|	NADP-thioredoxin reductase A [Medicago truncatula]	1E-51
	Contig823	gb|AAY26520.1|	secretory peroxidase [Catharanthus roseus]	6E-49
	Contig379	sp|P32110|GSTX6_SOYBN	Probable glutathione S-transferase (Heat shock protein 26A) (G2-4) gb|AAA33973.1| Gmhsp26-A	5E-48
	Contig43	emb|CAA62082.1|	cytochrome p450 [Arabidopsis thaliana]	4E-47
	Contig299	gb|AAD37427.1|AF149277_1	peroxidase 1 precursor [Phaseolus vulgaris]	5E-43
	CaF1_WIE_04_C_09	sp|P29450|TRXF_PEA	Thioredoxin F-type, chloroplast precursor (TRX-F) emb|CAA45098.1| thioredoxin F [Pisum sativum] gb|AAC49357.1| thioredoxin f	2E-42
	CaF1_WIE_17_A_11	gb|ABO77634.1|	peroxidase [Medicago truncatula]	1E-41

	Contig317	gb|ABC59084.1|	cytochrome P450 monooxygenase CYP83G1 [Medicago truncatula]	1E-40
	Contig723	emb|CAA62227.1|	peroxidase1C [Medicago sativa]	5E-40
	Contig197	emb|CAB50768.1|	cytochrome P450 [Cicer arietinum]	6E-40
	Contig254	ref|NP_567919.1|	peroxidase, putative [Arabidopsis thaliana]	1E-39
	Contig598	emb|CAB50768.1|	cytochrome P450 [Cicer arietinum]	1E-39
	CaF1_WIE_47_B_08	gb|ABO83980.1|	Thioredoxin domain 2; Thioredoxin fold [Medicago truncatula]	8E-39
	CaF1_WIE_03_F_01	gb|ABN09112.1|	Haem peroxidase, plant/fungal/bacterial [Medicago truncatula]	1E-37
	Contig637	gb|AAD37428.1|AF149278_1	peroxidase 3 precursor [Phaseolus vulgaris]	6E-37
	CaF1_WIE_02_H_08	ref|NP_175380.2|	peroxidase, putative [Arabidopsis thaliana] sp|Q9FX85|PER10_ARATH Peroxidase 10 precursor (Atperox P10) (ATP5a) gb|AAG13043.1|AC011807_2 peroxidase ATP5a [Arabidopsis thaliana] dbj|BAC43700.1| putative peroxidase [Arabidopsis thaliana] gb|AAP12891.1| At1g49570 [Arabidopsis thaliana]	2E-36
	Contig732	dbj|BAD97439.1|	peroxidase [Pisum sativum]	3E-36
	CaF1_JIE_04_D_05	gb|ABD32692.1|	2OG-Fe(II) oxygenase [Medicago truncatula]	2E-35
	Contig193	gb|ABN09112.1|	Haem peroxidase, plant/fungal/bacterial [Medicago truncatula]	3E-35
	CaF1_JIE_24_D_02	gb|ABE93515.2|	Cytochrome b5 [Medicago truncatula]	2E-28
	CaF1_JIE_25_A_06	gb|ABE79228.1|	2OG-Fe(II) oxygenase [Medicago truncatula]	4E-27
	CaF1_WIE_07_D_07	gb|ABN09825.1|	Thioredoxin domain 2; Thioredoxin fold [Medicago truncatula]	7E-26
	CaF1_WIE_32_H_10	gb|ABP03659.1|	Haem peroxidase, plant/fungal/bacterial [Medicago truncatula]	3E-21
	CaF1_WIE_30_B_09	gb|ABE68721.1|	putative IN2-1 protein [Arachis hypogaea]	1E-20
	Contig313	emb|CAB56741.1|	cytochrome P450 monooxygenase [Cicer arietinum]	2E-18
	Contig669	emb|CAB50768.1|	cytochrome P450 [Cicer arietinum]	1E-13
	CaF1_WIE_19_E_01	emb|CAB56744.1|	cytochrome P450 monooxygenase [Cicer arietinum]	7E-13
	CaF1_WIE_54_E_07	emb|CAD31718.1|	putative cytochrome P450 monooxygenase [Cicer arietinum]	7E-13
	CaF1_WIE_05_E_09	emb|CAB56743.1|	cytochrome P450 monooxygenase [Cicer arietinum]	2E-11
Cellular Transport/inorganic ion transport and metabolism	Contig683	gb|AAL17949.1|	type IIB calcium ATPase [Medicago truncatula]	9E-83
	Contig827	gb|ABE86679.1|	Intracellular chloride channel [Medicago truncatula]	4E-77
	CaF1_WIE_19_A_07	gb|ABE84066.1|	SecY protein; ABC transporter related [Medicago truncatula]	3E-75
	Contig736	dbj|BAC65212.1|	phosphate permease [Fusarium oxysporum]	8E-71
	Contig749	gb|AAO39834.1|	ferrous ion membrane transport protein DMT1 [Glycine max]	1E-67
	Contig938	gb|ABE93220.1|	General substrate transporter [Medicago truncatula]	2E-67
	CaF1_WIE_51_F_04	gb|ABN08184.1|	General substrate transporter [Medicago truncatula] gb|ABN09010.1| General substrate transporter [Medicago truncatula]	2E-65
	Contig171	gb|AAC64109.1|	signal recognition particle 54 kDa subunit precursor [Pisum sativum]	1E-63
	Contig648	gb|ABC01895.1|	transporter-like protein [Solanum tuberosum]	6E-62
	CaF1_WIE_48_F_05	gb|ABD32921.2|	F5O11.19, related [Medicago truncatula]	7E-61
	Contig202	gb|ABE79078.1|	Mitochondrial carrier protein [Medicago truncatula]	9E-61
	Contig484	gb|ABN08635.1|	Mitochondrial import inner membrane translocase, subunit Tim17/22 [Medicago truncatula]	2E-60
	Contig219	gb|ABP02565.1|	General substrate transporter [Medicago truncatula]	5E-56
	CaF1_WIE_45_A_04	gb|AAF22842.1|AF209910_1	vacuolar sorting receptor protein [Prunus dulcis]	3E-50
	Contig597	ref|NP_563985.1|	GOS11 (GOLGI SNARE 11); SNARE binding [Arabidopsis thaliana] sp|Q9LMP7|GOS11_ARATH Golgi SNARE 11 protein (AtGOS11) (Golgi SNAP receptor complex member 1-1) gb|AAF82157.1|AC034256_21 Contains similarity to GOS28/P28 protein from Homo sapiens gb|AF047438. ESTs gb|F14225, gb|AA395297, gb|BE038320 come from this gene. [Arabidopsis thaliana] gb|AAG48789.1|AF332426_1 unknown protein [Arabidopsis thaliana] gb|AAK48904.1|AF357528_1 Golgi SNARE 11 protein [Arabidopsis thaliana] gb|AAK95249.1|AF410263_1 At1g15880/F7H2_20 [Arabidopsis thaliana] gb|AAM10347.1| At1g15880/F7H2_20 [Arabidopsis thaliana]	1E-49
	Contig862	ref|NP_564367.1|	integral membrane HRF1 family protein [Arabidopsis thaliana] ref|NP_001077633.1| integral membrane HRF1 family protein [Arabidopsis thaliana] gb|AAK64057.1| unknown protein [Arabidopsis thaliana] gb|AAM44952.1| unknown protein [Arabidopsis thaliana] gb|AAM63447.1| unknown [Arabidopsis thaliana]	1E-45
	CaF1_WIE_40_E_04	gb|ABN05791.1|	TrkA-N [Medicago truncatula] gb|ABP02181.1| TrkA-N [Medicago truncatula]	3E-45
	Contig828	gb|ABM69111.1|	phosphate transporter 5 [Medicago truncatula]	4E-45
	Contig305	gb|ABO82182.1|	Zinc finger, Sec23/Sec24-type; Sec23/Sec24 trunk region; Sec23/Sec24 helical region; Gelsolin region; Sec23/Sec24 beta-sandwich [Medicago truncatula]	3E-40

	CaF1_JIE_36_B_11	ref|NP_194186.1|	clathrin adaptor complexes medium subunit family protein [Arabidopsis thaliana] emb|CAA23008.1| clathrin coat assembly like protein [Arabidopsis thaliana] emb|CAB79365.1| clathrin coat assembly like protein [Arabidopsis thaliana] gb|AAL59993.1| putative clathrin coat assembly protein [Arabidopsis thaliana]	3E-39
	CaF1_WIE_09_A_05	gb|AY147012.1|	Medicago truncatula type IIB calcium ATPase (MCA6) mRNA, partial cds	3E-38
	CaF1_JIE_24_F_11	ref|NP_179680.1|	secretory carrier membrane protein (SCAMP) family protein [Arabidopsis thaliana] gb|AAD20911.1| putative secretory carrier-associated membrane protein [Arabidopsis thaliana] gb|AAT06461.1| At2g20840 [Arabidopsis thaliana] dbj|BAD93720.1| putative secretory carrier-associated membrane protein [Arabidopsis thaliana]	1E-32
	CaF1_JIE_27_E_08	emb|CAJ29291.1|	putative polyol transporter protein 4 [Lotus japonicus]	2E-32
	CaF1_WIE_21_G_11	gb|AF089724.1|AF089724	Pisum sativum signal recognition particle 54 kDa subunit precursor (Ffc) mRNA, nuclear gene encoding chloroplast protein, partial cds	1E-29
	Contig331	gb|AAM91533.1|	membrane related protein CP5, putative [Arabidopsis thaliana] gb|AAN15331.1| membrane related protein CP5, putative [Arabidopsis thaliana]	6E-29
	CaF1_WIE_55_G_06	gb|ABE90826.1|	von Willebrand factor, type C; Heavy metal transport/detoxification protein [Medicago truncatula]	7E-29
	CaF1_JIE_15_G_09	gb|AAA66200.1|	signal recognition particle 54 kDa subunit	6E-28
	CaF1_JIE_19_A_02	gb|AAQ87023.1|	VDAC3.1 [Lotus corniculatus var. japonicus]	7E-27
	CaF1_WIE_37_H_02	dbj|BAA76420.1|	multidrug resistance protein [Cicer arietinum]	3E-20
	CaF1_WIE_19_B_07	dbj|BAB85760.1|	putative mitochondrial carrier protein [Fusarium oxysporum]	9E-17
	CaF1_JIE_37_C_10	gb|ABN08360.1|	Cation transporting ATPase, C-terminal [Medicago truncatula]	3E-51
	Contig626	sp|Q41009|TOC34_PEA	Translocase of chloroplast 34 (34 kDa chloroplast outer envelope protein) (GTP-binding protein OEP34) (GTP-binding protein IAP34) emb|CAA82196.1| chloroplast outer envelope protein 34 [Pisum sativum] gb|AAC25785.1| GTP-binding protein [Pisum sativum]	3E-71
	CaF1_JIE_26_G_09	sp|Q41009|TOC34_PEA	Translocase of chloroplast 34 (34 kDa chloroplast outer envelope protein) (GTP-binding protein OEP34) (GTP-binding protein IAP34) emb|CAA82196.1| chloroplast outer envelope protein 34 [Pisum sativum] gb|AAC25785.1| GTP-binding protein [Pisum sativum]	1E-54
	CaF1_WIE_48_B_10	gb|ABE93939.2|	Nuclear pore complex protein Nup205 , related [Medicago truncatula]	7E-34
	Contig118	emb|CAD33928.1|	tonoplast intrinsic protein [Cicer arietinum]	1E-31
	CaF1_WIE_34_E_11	emb|CAD33928.1|	tonoplast intrinsic protein [Cicer arietinum]	1E-31
	Contig472	ref|NP_190919.1|	ABC transporter family protein [Arabidopsis thaliana] sp|Q9LFG8|WBC20_ARATH Probable white-brown complex homolog protein 20 emb|CAB67658.1| ABC transporter-like protein [Arabidopsis thaliana]	2E-11
	CaF1_WIE_37_G_02	gb|ABE93744.2|	FAR1; Heavy metal transport/detoxification protein; Zinc finger, SWIM-type [Medicago truncatula]	1E-66
	CaF1_WIE_32_A_04	gb|ABE81754.1|	Ctr copper transporter [Medicago truncatula]	1E-53
	CaF1_JIE_36_H_10	gb|ABN05714.1|	metal ion transporter , putative [Medicago truncatula] gb|ABE84684.2| metal ion transporter , putative [Medicago truncatula]	2E-21
	CaF1_WIE_50_E_05	emb|CAC67501.1|	selenium binding protein [Medicago sativa]	1E-61
	Contig441	emb|CAC67501.1|	selenium binding protein [Medicago sativa]	2E-57
	CaF1_WIE_47_C_04	gb|ABE82410.1|	SBP [Medicago truncatula] gb|ABE92194.1| SBP [Medicago truncatula]	2E-41
	Contig712	emb|CAA10134.1|	basic blue copper protein [Cicer arietinum]	1E-30
	CaF1_WIE_56_G_07	emb|CAC67501.1|	selenium binding protein [Medicago sativa]	7E-30
	CaF1_WIE_47_D_07	ref|NP_199656.1|	SKS3 (SKU5 Similar 3); copper ion binding [Arabidopsis thaliana]	5E-19
Cytoskeleton	CaF1_WIE_53_E_05	gb|ABF59516.1|	putative spindle disassembly related protein CDC48 [Nicotiana tabacum]	4E-77
	CaF1_JIE_20_C_03	gb|EDN04544.1|	actin [Ajellomyces capsulatus NAm1]	4E-76
	CaF1_JIE_40_B_07	sp|P28551|TBB3_SOYBN	Tubulin beta chain (Beta tubulin) emb|CAA42777.1| beta-tubulin [Glycine max]	6E-63
	Contig855	gb|ABE90729.1|	Myosin II heavy chain-like [Medicago truncatula] gb|ABO80686.1| Myosin II heavy chain-like [Medicago truncatula]	7E-58
	CaF1_WIE_16_A_11	gb|ABA81885.1|	profilin-like [Solanum tuberosum]	5E-56
	CaF1_WIE_48_E_08	sp|Q8SAG3|ADF_VITVI	Actin-depolymerizing factor (ADF) gb|AAL79826.1|AF440310_1 actin depolymerizing factor [Vitis vinifera]	5E-46
	Contig912	emb|X68649.1|PSACTG	P.sativum mRNA for actin	3E-45
	Contig846	dbj|BAB88648.1|	microtubule bundling polypeptide TMBP200 [Nicotiana tabacum]	7E-40
	CaF1_WIE_41_E_04	sp|Q39445|TBB_CICAR	Tubulin beta chain (Beta tubulin) emb|CAA67056.1| beta-tubulin [Cicer arietinum]	2E-38
	CaF1_JIE_09_H_01	gb|AAX86048.1|	tubulin B4 [Glycine max]	2E-36
	Contig624	gb|ABF59516.1|	putative spindle disassembly related protein CDC48 [Nicotiana tabacum]	2E-34

	Contig413	gb|AAZ94896.1|	actin-like protein [Catharanthus roseus]	3E-34
	Contig416	dbj|BAB40710.1|	BY-2 kinesin-like protein 10 [Nicotiana tabacum]	3E-33
	CaF1_WIE_46_D_04	gb|ABI51282.1|	actin [Hibiscus cannabinus]	5E-22
	CaF1_WIE_37_E_06	gb|ABG45901.1|	actin [Cryptosporidium sp. Czech B1] gb|ABG45905.1| actin [Cryptosporidium sp. BB23] gb|ABG45906.1| actin [Cryptosporidium sp. BP1]	1E-12
	CaF1_WIE_05_H_02	gb|AAM63510.1|	Actin-depolymerizing factor ADF-6 [Arabidopsis thaliana]	2E-12
Defense mechanism	Contig892	gb|AAV92899.1|	Avr9/Cf-9 rapidly elicited protein 140 [Nicotiana tabacum]	9E-16
	CaF1_JIE_08_E_06	gb|AAV92900.1|	Avr9/Cf-9 rapidly elicited protein 150 [Nicotiana tabacum]	2E-11
	CaF1_WIE_41_E_01	gb|ABO84738.1|	Harpin-induced 1 [Medicago truncatula]	9E-35
	Contig764	emb|CAB71128.2|	cationic peroxidase [Cicer arietinum]	1E-84
	Contig559	sp|P27047|DRR4_PEA	Disease resistance response protein DRRG49-C gb|AAA33663.1| disease resistance response protein (DRRG49-c)	1E-71
	Contig411	emb|CAA10189.1|	class I chitinase [Cicer arietinum]	2E-57
	Contig942	gb|AAC77929.1|	similar to Nicotiana HR lesion-inducing ORF [Medicago sativa]	4E-57
	CaF1_WIE_01_C_08	emb|CAA10189.1|	class I chitinase [Cicer arietinum]	6E-57
	CaF1_WIE_31_D_09	gb|ABP02096.1|	Thaumatin, pathogenesis-related [Medicago truncatula]	1E-49
	Contig643	emb|CAG34224.1|	putative Bet v I family protein [Cicer arietinum]	8E-47
	CaF1_JIE_25_B_07	gb|AAP03880.2|	Avr9/Cf-9 induced kinase 1 [Nicotiana tabacum]	9E-45
	Contig773	gb|ABE84488.1|	Plant lipid transfer/seed storage/trypsin-alpha amylase inhibitor [Medicago truncatula]	1E-40
	Contig863	gb|AAP03880.2|	Avr9/Cf-9 induced kinase 1 [Nicotiana tabacum]	2E-40
	Contig941	gb|ABE79318.1|	Plant lipid transfer/seed storage/trypsin-alpha amylase inhibitor [Medicago truncatula]	4E-33
	Contig437	gb|AAB29959.2|	pathogen- and wound-inducible antifungal protein CBP20 precursor [Nicotiana tabacum]	1E-27
	Contig953	sp|P22196|PER2_ARAHY	Cationic peroxidase 2 precursor (PNPC2) gb|AAA32676.1| cationic peroxidase	8E-26
	Contig264	gb|AAC49370.1|	non-specific lipid transfer-like protein	3E-25
	CaF1_WIE_36_B_08	gb|AAF25357.1|AF210061_1	dirigent protein [Forsythia x intermedia]	8E-25
	CaF1_JIE_07_G_06	sp|P22196|PER2_ARAHY	Cationic peroxidase 2 precursor (PNPC2) gb|AAA32676.1| cationic peroxidase	1E-22
	Contig725	gb|AAP37978.1|	class 10 pathogenesis-related protein [Lupinus luteus]	2E-22
	CaF1_WIE_48_F_10	gb|ABL98074.1|	chitinase-related agglutinin [Robinia pseudoacacia]	7E-22
	CaF1_JIE_20_A_02	emb|CAA10189.1|	class I chitinase [Cicer arietinum]	3E-21
	CaF1_WIE_32_G_11	gb|ABL98074.1|	chitinase-related agglutinin [Robinia pseudoacacia]	3E-21
	Contig834	emb|CAA56142.1|	pathogenesis related protein [Cicer arietinum]	4E-20
Development/storage/dormancy and senescence	Contig654	emb|CAD31716.1|	putative ripening related protein [Cicer arietinum]	2E-41
	Contig266	emb|CAD31716.1|	putative ripening related protein [Cicer arietinum]	4E-29
	CaF1_JIE_25_A_02	gb|AAB84193.1|	dormancy-associated protein [Pisum sativum]	9E-38
	CaF1_WIE_53_E_03	dbj|AB049721.2|	Pisum sativum ssa-11 mRNA for putative senescence-associated protein, complete cds	5E-78
	Contig575	dbj|BAB33421.1|	putative senescence-associated protein [Pisum sativum]	4E-45
	Contig962	dbj|BAB33421.1|	putative senescence-associated protein [Pisum sativum]	4E-19
	Contig839	gb|AAD33922.1|AF143954_1	agglutinin [Amaranthus hypochondriacus] emb|CAA77664.1| seed specific protein of balanced nutritional quality [Amaranthus hypochondriacus]	1E-163
	CaF1_WIE_09_E_10	emb|CAI56441.1|	germin-like protein [Cicer arietinum]	5E-63
	CaF1_JIE_42_A_04	sp|P08688|ALB2_PEA	Albumin-2 (PA2) gb|AAA02981.1| albumin 2 gb|AAA33641.1| major seed albumin prf||1314296A albumin	2E-56
	Contig532	sp|P08688|ALB2_PEA	Albumin-2 (PA2) gb|AAA02981.1| albumin 2 gb|AAA33641.1| major seed albumin prf||1314296A albumin	6E-26
Energy production and conversion	Contig709	gb|AAZ23107.1|	plastid ATP/ADP transport protein 2 [Manihot esculenta]	3E-62
	Contig356	gb|AAY56795.1|	vacuolar H+-ATPase subunit A [Vigna unguiculata]	2E-79
	CaF1_WIE_39_A_08	gb|ABE84767.2|	ABC-2; AAA ATPase [Medicago truncatula]	5E-73
	CaF1_JIE_07_E_03	gb|ABN08957.1|	H+-transporting two-sector ATPase, C (AC39) subunit [Medicago truncatula]	2E-72
	Contig656	gb|AAM65274.1|	NADH dehydrogenase [Arabidopsis thaliana]	1E-33
	Contig651	gb|AAY44152.1|	ATP synthase beta subunit [Ecballium elaterium]	3E-66
	CaF1_WIE_35_F_01	dbj|BAE71236.1|	putative ADP,ATP carrier-like protein [Trifolium pratense]	3E-58
	Contig348	ref|NP_180560.1|	NDA2 (ALTERNATIVE NAD(P)H DEHYDROGENASE 2); NADH dehydrogenase [Arabidopsis thaliana] gb|AAC31853.1| putative NADH dehydrogenase (ubiquinone oxidoreductase) [Arabidopsis thaliana]	3E-52
	Contig165	gb|ABP02830.1|	H+-transporting two-sector ATPase, alpha/beta subunit, central region; H+-transporting two-sector ATPase, alpha/beta subunit, C-terminal [Medicago truncatula]	1E-48

	Contig231	dbj|BAE71236.1|	putative ADP,ATP carrier-like protein [Trifolium pratense]	9E-48
	Contig739	ref|XP_384488.1|	ATPB_NEUCR ATP synthase beta chain, mitochondrial precursor [Gibberella zeae PH-1]	2E-47
	CaF1_WIE_27_H_05	sp|P22778|ATPO_IPOBA	ATP synthase delta chain, mitochondrial precursor (Oligomycin sensitivity conferral protein) (OSCP) gb|AAA33388.1| F-1-ATPase delta subunit precursor (EC 3.6.1.3)	4E-32
	CaF1_WIE_03_B_07	gb|AAP68983.1|	alternative oxidase 2b [Glycine max]	6E-31
	Contig806	ref|XP_381601.1|	PMA1_NEUCR Plasma membrane ATPase (Proton pump) [Gibberella zeae PH-1]	1E-24
	Contig884	sp|O23948|VATE_GOSHI	Vacuolar ATP synthase subunit E (V-ATPase subunit E) (Vacuolar proton pump subunit E) gb|AAB72177.1| vacuolar H+-ATPase subunit E [Gossypium hirsutum]	2E-24
	Contig126	ref|XP_386197.1|	ADT_NEUCR ADP,ATP CARRIER PROTEIN (ADP/ATP TRANSLOCASE) (ADENINE NUCLEOTIDE TRANSLOCATOR) (ANT) [Gibberella zeae PH-1]	3E-22
	CaF1_WIE_27_C_03	ref|XP_381601.1|	PMA1_NEUCR Plasma membrane ATPase (Proton pump) [Gibberella zeae PH-1]	1E-20
Hormone responsive	Contig203	gb|AAV63565.1|	auxin-induced putative aldo/keto reductase family protein [Arachis hypogaea]	1E-17
	Contig558	gb|AAZ66745.1|	coronatine-insensitive 1 [Glycine max]	1E-104
	CaF1_JIE_20_C_08	emb|CAJ13711.1|	putative ethylene response protein [Capsicum chinense]	9E-56
	Contig567	gb|ABP87900.1|	ethylene receptor [Glycine max]	6E-46
	Contig116	gb|AAL32037.2|AF439278_1	ethylene-responsive transciptional coactivator-like protein [Retama raetam]	5E-42
	Contig605	gb|ABO82441.1|	Auxin responsive SAUR protein [Medicago truncatula]	2E-40
	Contig758	sp|P35694|BRU1_SOYBN	Brassinosteroid-regulated protein BRU1 precursor gb|AAA81350.1| brassinosteroid-regulated protein	8E-31
	CaF1_WIE_05_A_10	gb|AAK62821.1|AF332960_1	auxin-regulated dual specificity cytosolic kinase [Lycopersicon esculentum]	3E-29
	Contig261	emb|CAC84710.1|	aux/IAA protein [Populus tremula x Populus tremuloides]	1E-24
	Contig240	gb|AAM65588.1|	putative auxin-induced protein, IAA12 [Arabidopsis thaliana]	4E-18
Hypothetical protein	CaF1_WIE_48_F_01	gb|EAZ32228.1|	hypothetical protein OsJ_015711 [Oryza sativa (japonica cultivar-group)]	2E-19
	Contig727	emb|CAN73348.1|	hypothetical protein [Vitis vinifera]	1E-160
	Contig886	emb|AJ006760.1|CAR6760	Cicer arietinum mRNA for hypothetical protein, clone Can107	1E-107
	Contig84	dbj|BAD93605.1|	hypothetical protein [Cucumis melo]	1E-103
	Contig585	emb|CAN74145.1|	hypothetical protein [Vitis vinifera]	2E-83
	Contig184	ref|XP_380754.1|	hypothetical protein FG00578.1 [Gibberella zeae PH-1]	2E-78
	CaF1_WIE_34_H_09	emb|CAB95829.1|	hypothetical protein [Cicer arietinum]	1E-77
	Contig913	ref|XP_720736.1|	hypothetical protein CaO19_11777 [Candida albicans SC5314] ref|XP_720608.1| hypothetical protein CaO19_4301 [Candida albicans SC5314] gb|EAL01773.1| hypothetical protein CaO19.4301 [Candida albicans SC5314] gb|EAL01907.1| hypothetical protein CaO19.11777 [Candida albicans SC5314]	6E-76
	CaF1_JIE_05_B_08	gb|EAZ41147.1|	hypothetical protein OsJ_024630 [Oryza sativa (japonica cultivar-group)]	9E-75
	Contig948	emb|CAB76913.1|	hypothetical protein [Cicer arietinum]	4E-74
	Contig37	ref|XP_382177.1|	hypothetical protein FG02001.1 [Gibberella zeae PH-1]	1E-73
	CaF1_WIE_29_F_02	ref|XP_383424.1|	hypothetical protein FG03248.1 [Gibberella zeae PH-1]	1E-70
	CaF1_WIE_27_E_06	ref|XP_380918.1|	hypothetical protein FG00742.1 [Gibberella zeae PH-1]	3E-70
	CaF1_WIE_30_D_06	ref|XP_388371.1|	hypothetical protein FG08195.1 [Gibberella zeae PH-1]	6E-70
	Contig18	emb|CAN70526.1|	hypothetical protein [Vitis vinifera]	2E-69
	Contig273	emb|CAN75753.1|	hypothetical protein [Vitis vinifera]	2E-69
	CaF1_WIE_01_B_04	emb|AJ404640.1|CAR404640	Cicer arietinum mRNA for for hypothetical protein, clone Can47	2E-69
	CaF1_JIE_19_G_03	emb|CAN72598.1|	hypothetical protein [Vitis vinifera]	1E-68
	Contig680	dbj|BAF01964.1|	hypothetical protein [Arabidopsis thaliana]	3E-68
	Contig578	emb|CAN69512.1|	hypothetical protein [Vitis vinifera]	6E-68
	Contig757	emb|CAN73350.1|	hypothetical protein [Vitis vinifera]	6E-68
	CaF1_WIE_12_C_07	emb|CAN77946.1|	hypothetical protein [Vitis vinifera]	1E-67
	CaF1_JIE_07_B_04	emb|CAN77652.1|	hypothetical protein [Vitis vinifera]	3E-67
	CaF1_WIE_43_H_07	dbj|BAD06518.1|	hypothetical protein [Pisum sativum] dbj|BAD12184.1| 12-oxophytodienoic acid 10, 11-reductase [Pisum sativum] gb|AAX54688.1| 12-oxophytodienoic acid 10,10-reductase [Pisum sativum]	3E-67
	Contig269	ref|XP_383344.1|	hypothetical protein FG03168.1 [Gibberella zeae PH-1]	7E-67
	CaF1_JIE_33_D_08	emb|CAN78553.1|	hypothetical protein [Vitis vinifera]	3E-66
	Contig415	emb|CAN80597.1|	hypothetical protein [Vitis vinifera]	4E-66
	Contig787	gb|ABE86786.1|	hypothetical protein MtrDRAFT_AC124956g13v2 [Medicago truncatula]	4E-66
	Contig301	gb|ABO79664.1|	hypothetical protein MtrDRAFT_AC137701g32v2 [Medicago truncatula]	8E-66

	Contig799	dbj|BAE71208.1|	hypothetical protein [Trifolium pratense] dbj|BAE71210.1| hypothetical protein [Trifolium pratense]	3E-65
	Contig177	emb|CAN82780.1|	hypothetical protein [Vitis vinifera]	8E-65
	Contig311	emb|CAN77754.1|	hypothetical protein [Vitis vinifera] emb|CAN81281.1| hypothetical protein [Vitis vinifera]	1E-64
	CaF1_WIE_03_A_11	dbj|BAF01953.1|	hypothetical protein [Arabidopsis thaliana]	2E-64
	Contig964	emb|CAN63456.1|	hypothetical protein [Vitis vinifera]	2E-63
	CaF1_JIE_08_F_08	gb|ABE93079.1|	hypothetical protein MtrDRAFT_AC144517g10v2 [Medicago truncatula]	2E-63
	CaF1_WIE_17_F_09	emb|CAN65847.1|	hypothetical protein [Vitis vinifera]	1E-62
	CaF1_WIE_34_G_01	emb|CAN70797.1|	hypothetical protein [Vitis vinifera]	2E-62
	Contig195	ref|XP_389229.1|	hypothetical protein FG09053.1 [Gibberella zeae PH-1]	9E-62
	CaF1_WIE_18_F_06	emb|CAI84657.1|	hypothetical protein [Nicotiana tabacum]	1E-61
	CaF1_WIE_49_F_10	emb|CAN65965.1|	hypothetical protein [Vitis vinifera]	6E-61
	CaF1_JIE_08_D_09	emb|CAN65776.1|	hypothetical protein [Vitis vinifera]	7E-61
	CaF1_JIE_32_A_06	emb|CAN68264.1|	hypothetical protein [Vitis vinifera]	7E-61
	Contig102	emb|CAN63147.1|	hypothetical protein [Vitis vinifera]	3E-60
	CaF1_JIE_41_A_04	emb|CAN74802.1|	hypothetical protein [Vitis vinifera]	4E-60
	Contig335	ref|XP_386920.1|	hypothetical protein FG06744.1 [Gibberella zeae PH-1]	1E-59
	Contig485	emb|CAN63150.1|	hypothetical protein [Vitis vinifera]	3E-58
	Contig297	emb|CAN67631.1|	hypothetical protein [Vitis vinifera]	2E-56
	Contig775	emb|CAN61836.1|	hypothetical protein [Vitis vinifera]	2E-56
	Contig659	emb|CAN63851.1|	hypothetical protein [Vitis vinifera]	4E-56
	Contig173	gb|ABP02356.1|	hypothetical protein MtrDRAFT_AC130802g5v1 [Medicago truncatula]	9E-56
	Contig873	emb|CAN71478.1|	hypothetical protein [Vitis vinifera]	1E-55
	Contig445	ref|XP_712836.1|	hypothetical protein CaO19.14167 [Candida albicans SC5314] ref|XP_712807.1| hypothetical protein CaO19.6878 [Candida albicans SC5314] gb|EAK93638.1| questionable orf [Candida albicans SC5314] gb|EAK93667.1| questionable orf [Candida albicans SC5314]	4E-12
	Contig45	emb|CAN77029.1|	hypothetical protein [Vitis vinifera]	2E-55
	Contig210	ref|XP_384070.1|	hypothetical protein FG03894.1 [Gibberella zeae PH-1]	3E-55
	CaF1_WIE_05_B_06	gb|ABN08784.1|	Hypothetical 214.8 kDa protein ycf1, related [Medicago truncatula]	6E-55
	Contig78	gb|ABP02285.1|	hypothetical protein MtrDRAFT_AC126789g51v2 [Medicago truncatula]	2E-54
	CaF1_WIE_13_C_10	dbj|BAE99246.1|	hypothetical protein [Arabidopsis thaliana]	2E-54
	CaF1_WIE_53_G_06	ref|XP_386883.1|	hypothetical protein FG06707.1 [Gibberella zeae PH-1]	3E-54
	Contig58	emb|CAN71158.1|	hypothetical protein [Vitis vinifera]	8E-54
	Contig87	gb|ABE91660.2|	hypothetical protein MtrDRAFT_AC145219g51v2 [Medicago truncatula]	9E-54
	CaF1_WIE_12_G_10	gb|ABE80179.1|	hypothetical protein MtrDRAFT_AC139601g19v2 [Medicago truncatula]	2E-53
	Contig602	emb|CAN59927.1|	hypothetical protein [Vitis vinifera]	3E-53
	Contig326	gb|EAY79580.1|	hypothetical protein OsI_033539 [Oryza sativa (indica cultivar-group)]	7E-53
	CaF1_JIE_18_C_07	ref|XP_460488.1|	hypothetical protein DEHA0F03157g [Debaryomyces hansenii CBS767] emb|CAG88798.1| unnamed protein product [Debaryomyces hansenii CBS767]	1E-52
	CaF1_WIE_23_E_11	emb|CAN71280.1|	hypothetical protein [Vitis vinifera]	1E-52
	CaF1_JIE_06_C_04	dbj|BAE71261.1|	hypothetical protein [Trifolium pratense]	2E-52
	CaF1_JIE_34_E_01	ref|XP_384354.1|	hypothetical protein FG04178.1 [Gibberella zeae PH-1]	3E-52
	Contig554	ref|XP_384449.1|	hypothetical protein FG04273.1 [Gibberella zeae PH-1]	6E-52
	Contig330	emb|CAN69269.1|	hypothetical protein [Vitis vinifera]	8E-52
	CaF1_JIE_10_F_02	emb|CAN82378.1|	hypothetical protein [Vitis vinifera]	1E-51
	CaF1_WIE_29_C_03	emb|CAN63881.1|	hypothetical protein [Vitis vinifera]	1E-51
	Contig303	ref|XP_384358.1|	hypothetical protein FG04182.1 [Gibberella zeae PH-1]	2E-51
	CaF1_WIE_41_A_06	emb|CAN81423.1|	hypothetical protein [Vitis vinifera]	2E-51
	Contig155	emb|CAN62476.1|	hypothetical protein [Vitis vinifera]	3E-51
	CaF1_WIE_20_A_06	emb|CAN82256.1|	hypothetical protein [Vitis vinifera]	4E-51
	CaF1_JIE_39_H_08	emb|CAA10289.1|	hypothetical protein [Cicer arietinum]	5E-51
	CaF1_WIE_02_C_03	emb|CAN63456.1|	hypothetical protein [Vitis vinifera]	9E-51
	Contig201	emb|CAN62762.1|	hypothetical protein [Vitis vinifera]	2E-50
	CaF1_WIE_32_C_09	ref|XP_380654.1|	conserved hypothetical protein [Gibberella zeae PH-1]	2E-50
	Contig433	ref|XP_381155.1|	conserved hypothetical protein [Gibberella zeae PH-1]	2E-49
	Contig623	gb|ABE77463.1|	conserved hypothetical protein [Medicago truncatula]	2E-49
	Contig2	gb|ABE86405.1|	hypothetical protein MtrDRAFT_AC156827g15v2 [Medicago truncatula]	3E-49
	Contig784	emb|CAN67205.1|	hypothetical protein [Vitis vinifera]	3E-49
	Contig698	emb|CAN81488.1|	hypothetical protein [Vitis vinifera]	2E-48
	CaF1_JIE_19_H_01	emb|CAN68819.1|	hypothetical protein [Vitis vinifera]	3E-48
	Contig128	gb|EAZ27896.1|	hypothetical protein OsJ_011379 [Oryza sativa (japonica cultivar-group)]	9E-48

	CaF1_WIE_20_C_09	gb|EAY92246.1|	hypothetical protein OsI_013479 [Oryza sativa (indica cultivar-group)] gb|EAZ28970.1| hypothetical protein OsJ_012453 [Oryza sativa (japonica cultivar-group)]	9E-48
	Contig61	emb|CAN81116.1|	hypothetical protein [Vitis vinifera]	1E-47
	CaF1_JIE_18_H_08	ref|XP_388653.1|	hypothetical protein FG08477.1 [Gibberella zeae PH-1]	1E-47
	Contig960	emb|CAN73778.1|	hypothetical protein [Vitis vinifera]	2E-47
	CaF1_WIE_11_B_03	gb|ABE89815.1|	hypothetical protein MtrDRAFT_AC148171g28v2 [Medicago truncatula]	2E-47
	Contig68	emb|CAN73756.1|	hypothetical protein [Vitis vinifera]	3E-47
	Contig505	ref|XP_720740.1|	hypothetical protein CaO19_11781 [Candida albicans SC5314] ref|XP_720612.1| hypothetical protein CaO19_4305 [Candida albicans SC5314] gb|EAL01777.1| hypothetical protein CaO19.4305 [Candida albicans SC5314] gb|EAL01911.1| hypothetical protein CaO19.11781 [Candida albicans SC5314]	8E-47
	Contig630	ref|XP_386626.1|	hypothetical protein FG06450.1 [Gibberella zeae PH-1]	8E-47
	CaF1_JIE_23_H_02	emb|CAN71304.1|	hypothetical protein [Vitis vinifera]	1E-46
	Contig55	emb|CAN80511.1|	hypothetical protein [Vitis vinifera]	3E-46
	Contig222	gb|EAZ22851.1|	hypothetical protein OsJ_006334 [Oryza sativa (japonica cultivar-group)]	3E-46
	CaF1_JIE_09_G_05	emb|CAN80281.1|	hypothetical protein [Vitis vinifera]	3E-46
	Contig140	emb|CAN69887.1|	hypothetical protein [Vitis vinifera]	7E-46
	Contig932	gb|EAZ28486.1|	hypothetical protein OsJ_011969 [Oryza sativa (japonica cultivar-group)]	9E-46
	CaF1_JIE_22_E_06	gb|AAT40482.1|	hypothetical protein [Solanum demissum]	2E-45
	CaF1_WIE_55_E_09	emb|CAN74784.1|	hypothetical protein [Vitis vinifera]	4E-45
	Contig268	ref|XP_386905.1|	hypothetical protein FG06729.1 [Gibberella zeae PH-1]	5E-45
	Contig451	dbj|BAF45465.1|	hypothetical protein [Nicotiana tabacum]	1E-44
	CaF1_JIE_09_D_06	ref|XP_381184.1|	hypothetical protein FG01008.1 [Gibberella zeae PH-1]	2E-44
	Contig242	emb|CAN65596.1|	hypothetical protein [Vitis vinifera]	5E-44
	CaF1_JIE_34_B_11	emb|CAN80974.1|	hypothetical protein [Vitis vinifera]	8E-44
	CaF1_WIE_17_G_10	emb|CAA10123.1|	hypothetical protein [Cicer arietinum]	1E-43
	Contig214	gb|ABO79980.1|	hypothetical protein MtrDRAFT_AC138448g30v2 [Medicago truncatula]	2E-43
	CaF1_JIE_38_H_02	ref|XP_388777.1|	hypothetical protein FG08601.1 [Gibberella zeae PH-1]	2E-43
	Contig71	emb|CAN72193.1|	hypothetical protein [Vitis vinifera]	3E-43
	CaF1_WIE_15_H_09	dbj|BAF45465.1|	hypothetical protein [Nicotiana tabacum]	4E-43
	CaF1_JIE_16_H_01	emb|CAN76661.1|	hypothetical protein [Vitis vinifera]	5E-43
	CaF1_WIE_04_D_11	emb|CAN68264.1|	hypothetical protein [Vitis vinifera]	5E-43
	CaF1_JIE_25_E_09	emb|CAN68798.1|	hypothetical protein [Vitis vinifera]	7E-43
	Contig88	ref|XP_380875.1|	hypothetical protein FG00699.1 [Gibberella zeae PH-1]	2E-42
	Contig511	emb|CAN79836.1|	hypothetical protein [Vitis vinifera]	2E-42
	CaF1_WIE_35_F_08	emb|CAN82979.1|	hypothetical protein [Vitis vinifera]	2E-42
	CaF1_JIE_12_E_11	emb|CAN80884.1|	hypothetical protein [Vitis vinifera]	3E-42
	CaF1_WIE_56_F_04	dbj|BAE71253.1|	hypothetical protein [Trifolium pratense]	6E-42
	Contig82	gb|EDN28454.1|	conserved hypothetical protein [Botryotinia fuckeliana B05.10]	1E-41
	Contig629	emb|CAN63142.1|	hypothetical protein [Vitis vinifera]	3E-41
	Contig221	emb|CAN61355.1|	hypothetical protein [Vitis vinifera]	4E-41
	CaF1_WIE_15_G_06	gb|ABE83515.1|	hypothetical protein MtrDRAFT_AC129091g11v2 [Medicago truncatula]	4E-41
	CaF1_JIE_09_F_08	emb|CAN78632.1|	hypothetical protein [Vitis vinifera]	5E-41
	CaF1_JIE_08_D_11	ref|XP_383193.1|	hypothetical protein FG03017.1 [Gibberella zeae PH-1]	6E-41
	Contig209	emb|CAN79638.1|	hypothetical protein [Vitis vinifera]	2E-40
	CaF1_JIE_20_D_10	emb|CAN72566.1|	hypothetical protein [Vitis vinifera]	2E-40
	CaF1_WIE_02_G_07	gb|ABD96879.1|	hypothetical protein [Cleome spinosa]	2E-40
	Contig63	emb|CAN75568.1|	hypothetical protein [Vitis vinifera]	3E-40
	Contig576	emb|CAN63462.1|	hypothetical protein [Vitis vinifera]	4E-40
	CaF1_JIE_35_H_10	ref|XP_361955.2|	hypothetical protein MGG_04400 [Magnaporthe grisea 70-15] gb|EDJ96104.1| hypothetical protein MGG_04400 [Magnaporthe grisea 70-15]	4E-40
	Contig46	ref|XP_386464.1|	hypothetical protein FG06288.1 [Gibberella zeae PH-1]	9E-40
	CaF1_WIE_39_G_09	gb|ABE80590.2|	hypothetical protein MtrDRAFT_AC148398g42v2 [Medicago truncatula]	2E-39
	CaF1_WIE_44_G_03	emb|CAN68337.1|	hypothetical protein [Vitis vinifera]	2E-39
	Contig79	emb|CAN59845.1|	hypothetical protein [Vitis vinifera]	3E-39
	CaF1_WIE_48_H_02	emb|CAN65408.1|	hypothetical protein [Vitis vinifera]	3E-39
	Contig376	ref|XP_001227483.1|	hypothetical protein CHGG_09556 [Chaetomium globosum CBS 148.51] gb|EAQ85542.1| hypothetical protein CHGG_09556 [Chaetomium globosum CBS 148.51]	5E-39
	CaF1_WIE_03_D_05	emb|CAN73725.1|	hypothetical protein [Vitis vinifera]	1E-38
	Contig183	gb|EAZ27173.1|	hypothetical protein OsJ_010656 [Oryza sativa (japonica cultivar-group)]	2E-38
	Contig166	emb|CAN79713.1|	hypothetical protein [Vitis vinifera]	3E-38

	CaF1_WIE_49_B_06	gb|EAZ03842.1|	hypothetical protein OsI_025074 [Oryza sativa (indica cultivar-group)] gb|EAZ39784.1| hypothetical protein OsJ_023267 [Oryza sativa (japonica cultivar-group)]	3E-38
	CaF1_WIE_56_A_10	emb|CAN61192.1|	hypothetical protein [Vitis vinifera]	3E-38
	CaF1_WIE_24_G_08	emb|CAN83013.1|	hypothetical protein [Vitis vinifera]	4E-38
	Contig359	ref|XP_382134.1|	hypothetical protein FG01958.1 [Gibberella zeae PH-1]	7E-38
	CaF1_WIE_46_B_07	emb|CAN75568.1|	hypothetical protein [Vitis vinifera]	9E-38
	Contig284	emb|CAN65016.1|	hypothetical protein [Vitis vinifera]	1E-37
	CaF1_JIE_40_D_03	emb|CAA10289.1|	hypothetical protein [Cicer arietinum]	2E-37
	Contig930	emb|CAN82178.1|	hypothetical protein [Vitis vinifera]	3E-37
	Contig12	emb|CAN79806.1|	hypothetical protein [Vitis vinifera]	4E-37
	CaF1_WIE_09_H_07	gb|ABD96913.1|	hypothetical protein [Cleome spinosa]	4E-37
	Contig340	ref|XP_721448.1|	hypothetical protein CaO19_2949 [Candida albicans SC5314] ref|XP_721176.1| hypothetical protein CaO19_10466 [Candida albicans SC5314] gb|EAL02368.1| hypothetical protein CaO19.10466 [Candida albicans SC5314] gb|EAL02649.1| hypothetical protein CaO19.2949 [Candida albicans SC5314]	8E-37
	Contig324	emb|CAN78902.1|	hypothetical protein [Vitis vinifera]	1E-36
	CaF1_JIE_31_D_02	ref|XP_381197.1|	conserved hypothetical protein [Gibberella zeae PH-1] sp|Q8L805|RL35_WHEAT 60S ribosomal protein L35 pdb|2GO5|5 Chain 5, Structure Of Signal Recognition Particle Receptor (Sr) In Complex With Signal Recognition Particle (Srp) And Ribosome Nascent Chain Complex pdb|2J37|5 Chain 5, Model Of Mammalian Srp Bound To 80s Rncs gb|AAM92709.1| putative ribosomal protein L35 [Triticum aestivum]	1E-36
	CaF1_WIE_40_A_05	emb|CAN64202.1|	hypothetical protein [Vitis vinifera]	2E-36
	CaF1_WIE_48_B_11	ref|XP_382956.1|	conserved hypothetical protein [Gibberella zeae PH-1]	3E-36
	Contig292	emb|CAG28693.1|	hypothetical protein [Gibberella fujikuroi]	6E-36
	Contig107	emb|CAN78160.1|	hypothetical protein [Vitis vinifera]	1E-35
	Contig388	gb|ABE89195.1|	hypothetical protein MtrDRAFT_AC146775g29v2 [Medicago truncatula]	1E-35
	Contig969	gb|ABE87707.1|	hypothetical protein MtrDRAFT_AC155898g5v1 [Medicago truncatula] gb|ABO83845.1| mitochondrial ATP synthase precursor, putative [Medicago truncatula]	1E-35
	CaF1_WIE_10_B_01	ref|XP_380839.1|	hypothetical protein FG00663.1 [Gibberella zeae PH-1]	1E-35
	Contig19	emb|CAN82003.1|	hypothetical protein [Vitis vinifera]	3E-35
	CaF1_WIE_13_D_07	emb|CAN78893.1|	hypothetical protein [Vitis vinifera]	7E-35
	CaF1_WIE_30_D_03	gb|ABN08660.1|	hypothetical protein MtrDRAFT_AC157891g33v2 [Medicago truncatula] gb|ABE94640.2| hypothetical protein MtrDRAFT_AC141114g34v2 [Medicago truncatula]	7E-35
	Contig77	emb|CAN78091.1|	hypothetical protein [Vitis vinifera]	1E-34
	CaF1_JIE_28_A_04	gb|EAZ17668.1|	hypothetical protein OsJ_031877 [Oryza sativa (japonica cultivar-group)]	1E-34
	Contig220	ref|XP_389399.1|	hypothetical protein FG09223.1 [Gibberella zeae PH-1]	2E-34
	CaF1_WIE_47_C_09	emb|CAN75662.1|	hypothetical protein [Vitis vinifera]	3E-34
	CaF1_WIE_54_B_01	emb|CAN76851.1|	hypothetical protein [Vitis vinifera]	6E-34
	CaF1_JIE_17_B_10	ref|XP_381443.1|	hypothetical protein FG01267.1 [Gibberella zeae PH-1]	7E-34
	CaF1_JIE_29_H_07	gb|EAY72790.1|	hypothetical protein OsI_000637 [Oryza sativa (indica cultivar-group)]	7E-34
	Contig887	emb|CAN81061.1|	hypothetical protein [Vitis vinifera]	2E-33
	CaF1_JIE_09_G_04	gb|EAY90304.1|	hypothetical protein OsI_011537 [Oryza sativa (indica cultivar-group)]	3E-33
	CaF1_WIE_47_C_11	emb|CAN65403.1|	hypothetical protein [Vitis vinifera]	3E-33
	Contig274	ref|XP_386196.1|	conserved hypothetical protein [Gibberella zeae PH-1]	6E-33
	CaF1_JIE_22_A_04	gb|EAT90924.1|	hypothetical protein SNOG_01275 [Phaeosphaeria nodorum SN15]	9E-33
	Contig646	emb|CAN80447.1|	hypothetical protein [Vitis vinifera]	1E-32
	Contig622	emb|CAA10129.1|	hypothetical protein [Cicer arietinum]	2E-32
	CaF1_WIE_16_B_10	emb|CAN78555.1|	hypothetical protein [Vitis vinifera]	2E-32
	CaF1_WIE_18_F_05	emb|CAN69723.1|	hypothetical protein [Vitis vinifera]	3E-32
	Contig48	gb|EAZ32237.1|	hypothetical protein OsJ_015720 [Oryza sativa (japonica cultivar-group)]	4E-32
	Contig142	emb|CAN62146.1|	hypothetical protein [Vitis vinifera]	5E-32
	CaF1_WIE_02_B_04	emb|CAN62584.1|	hypothetical protein [Vitis vinifera]	5E-32
	CaF1_WIE_17_E_01	gb|EAZ28165.1|	hypothetical protein OsJ_011648 [Oryza sativa (japonica cultivar-group)]	5E-32
	CaF1_WIE_20_G_11	gb|EAZ20254.1|	hypothetical protein OsJ_034463 [Oryza sativa (japonica cultivar-group)]	2E-31
	CaF1_JIE_36_D_11	emb|CAN64650.1|	hypothetical protein [Vitis vinifera]	6E-31
	CaF1_WIE_26_G_07	emb|CAN74446.1|	hypothetical protein [Vitis vinifera]	8E-31
	Contig65	emb|CAN69190.1|	hypothetical protein [Vitis vinifera]	1E-30
	Contig428	dbj|BAF45465.1|	hypothetical protein [Nicotiana tabacum]	1E-30
	CaF1_WIE_48_E_07	emb|CAN77483.1|	hypothetical protein [Vitis vinifera]	2E-30

	Contig33	gb|EAY73868.1|	hypothetical protein OsI_001715 [Oryza sativa (indica cultivar-group)]	3E-30
	Contig168	ref|XP_390479.1|	hypothetical protein FG10303.1 [Gibberella zeae PH-1]	3E-30
	Contig853	emb|CAN82123.1|	hypothetical protein [Vitis vinifera]	3E-30
	CaF1_JIE_32_E_06	ref|XP_380676.1|	conserved hypothetical protein [Gibberella zeae PH-1]	3E-30
	CaF1_WIE_03_H_11	emb|CAN78123.1|	hypothetical protein [Vitis vinifera]	4E-30
	Contig894	dbj|BAE99924.1|	hypothetical protein [Arabidopsis thaliana]	5E-30
	Contig769	ref|XP_382875.1|	hypothetical protein FG02699.1 [Gibberella zeae PH-1]	1E-29
	CaF1_WIE_44_B_06	gb|EAY85769.1|	hypothetical protein OsI_007002 [Oryza sativa (indica cultivar-group)]	1E-29
	Contig920	emb|CAN75020.1|	hypothetical protein [Vitis vinifera]	2E-29
	Contig60	ref|XP_001257542.1|	conserved hypothetical protein [Neosartorya fischeri NRRL 181] gb|EAW15645.1| conserved hypothetical protein [Neosartorya fischeri NRRL 181]	4E-29
	CaF1_WIE_19_A_01	gb|EAZ39797.1|	hypothetical protein OsJ_023280 [Oryza sativa (japonica cultivar-group)]	4E-29
	Contig364	ref|XP_387328.1|	hypothetical protein FG07152.1 [Gibberella zeae PH-1]	6E-29
	Contig869	emb|CAN67313.1|	hypothetical protein [Vitis vinifera]	6E-29
	CaF1_WIE_55_C_03	gb|EAZ03281.1|	hypothetical protein OsI_024513 [Oryza sativa (indica cultivar-group)]	6E-29
	CaF1_JIE_25_H_11	ref|XP_383636.1|	hypothetical protein FG03460.1 [Gibberella zeae PH-1]	7E-29
	CaF1_JIE_04_C_01	ref|XP_381616.1|	hypothetical protein FG01440.1 [Gibberella zeae PH-1]	9E-29
	CaF1_JIE_19_C_02	ref|XP_387328.1|	hypothetical protein FG07152.1 [Gibberella zeae PH-1]	2E-28
	CaF1_JIE_38_A_05	ref|XP_389789.1|	conserved hypothetical protein [Gibberella zeae PH-1]	3E-28
	Contig341	emb|CAN65177.1|	hypothetical protein [Vitis vinifera]	5E-28
	CaF1_WIE_31_D_10	dbj|BAF01964.1|	hypothetical protein [Arabidopsis thaliana]	4E-17
	CaF1_JIE_03_C_09	emb|CAN66051.1|	hypothetical protein [Vitis vinifera]	6E-28
	Contig117	ref|XP_958142.1|	hypothetical protein [Neurospora crassa OR74A] gb|EAA28906.1| hypothetical protein [Neurospora crassa]	8E-28
	Contig832	emb|CAN81471.1|	hypothetical protein [Vitis vinifera]	8E-28
	Contig59	emb|CAG28692.1|	hypothetical protein [Gibberella fujikuroi]	2E-27
	Contig69	gb|ABD33305.1|	hypothetical protein MtrDRAFT_AC158502g14v2 [Medicago truncatula]	2E-27
	Contig72	ref|XP_382177.1|	hypothetical protein FG02001.1 [Gibberella zeae PH-1]	2E-27
	Contig689	emb|CAN78056.1|	hypothetical protein [Vitis vinifera]	2E-27
	CaF1_WIE_25_D_06	emb|CAN63304.1|	hypothetical protein [Vitis vinifera]	2E-27
	CaF1_WIE_46_C_03	gb|EAZ30583.1|	hypothetical protein OsJ_014066 [Oryza sativa (japonica cultivar-group)]	5E-27
	Contig342	emb|CAN74679.1|	hypothetical protein [Vitis vinifera]	7E-27
	Contig475	emb|CAA17544.1|	hypothetical protein [Arabidopsis thaliana] emb|CAB79125.1| hypothetical protein [Arabidopsis thaliana]	1E-26
	Contig442	emb|CAN66493.1|	hypothetical protein [Vitis vinifera]	2E-26
	CaF1_JIE_20_G_10	ref|XP_384352.1|	hypothetical protein FG04176.1 [Gibberella zeae PH-1]	2E-26
	Contig207	ref|XP_391558.1|	hypothetical protein FG11382.1 [Gibberella zeae PH-1]	3E-26
	CaF1_JIE_15_A_09	gb|EAY96947.1|	hypothetical protein OsI_018180 [Oryza sativa (indica cultivar-group)]	3E-26
	CaF1_WIE_16_H_06	emb|CAN70439.1|	hypothetical protein [Vitis vinifera]	3E-26
	CaF1_JIE_41_H_04	ref|XP_383642.1|	hypothetical protein FG03466.1 [Gibberella zeae PH-1]	5E-26
	Contig366	emb|CAN62209.1|	hypothetical protein [Vitis vinifera]	6E-26
	CaF1_JIE_40_F_07	emb|CAN82032.1|	hypothetical protein [Vitis vinifera]	1E-25
	CaF1_WIE_24_H_10	ref|XP_381029.1|	hypothetical protein FG00853.1 [Gibberella zeae PH-1]	1E-25
	CaF1_WIE_50_E_02	ref|XP_364191.1|	hypothetical protein MGG_09036 [Magnaporthe grisea 70-15] ref|XP_001522086.1| hypothetical protein MGCH7_ch7g203 [Magnaporthe grisea 70-15] gb|EAQ70796.1| hypothetical protein MGCH7_ch7g203 [Magnaporthe grisea 70-15] gb|EDK02087.1| hypothetical protein MGG_09036 [Magnaporthe grisea 70-15]	1E-25
	Contig729	emb|CAN67946.1|	hypothetical protein [Vitis vinifera]	2E-25
	CaF1_WIE_28_H_03	ref|XP_383076.1|	hypothetical protein FG02900.1 [Gibberella zeae PH-1]	2E-25
	CaF1_JIE_05_E_05	gb|EAZ03897.1|	hypothetical protein OsI_025129 [Oryza sativa (indica cultivar-group)]	3E-25
	CaF1_WIE_10_H_08	ref|XP_381235.1|	hypothetical protein FG01059.1 [Gibberella zeae PH-1] sp|Q4INZ9|FKBP4_GIBZE FK506-binding protein 4 (Peptidyl-prolyl cis-trans isomerase) (PPIase) (Rotamase)	3E-25
	Contig492	emb|CAB71133.1|	hypothetical protein [Cicer arietinum]	4E-25
	Contig731	emb|CAN77737.1|	hypothetical protein [Vitis vinifera]	4E-25
	CaF1_JIE_04_A_03	ref|XP_388769.1|	hypothetical protein FG08593.1 [Gibberella zeae PH-1]	4E-25
	CaF1_WIE_08_H_06	emb|CAN63226.1|	hypothetical protein [Vitis vinifera]	4E-25
	Contig136	emb|CAN62771.1|	hypothetical protein [Vitis vinifera]	5E-25
	Contig466	emb|CAN76898.1|	hypothetical protein [Vitis vinifera]	2E-24
	Contig546	emb|CAN66217.1|	hypothetical protein [Vitis vinifera]	2E-24
	Contig777	ref|XP_389679.1|	hypothetical protein FG09503.1 [Gibberella zeae PH-1]	2E-24

	Contig937	emb|CAN73618.1|	hypothetical protein [Vitis vinifera]	4E-24
	CaF1_WIE_05_A_11	gb|ABD96861.1|	hypothetical protein [Cleome spinosa]	5E-24
	CaF1_JIE_15_A_06	gb|EAY88020.1|	hypothetical protein OsI_009253 [Oryza sativa (indica cultivar-group)]	3E-23
	CaF1_WIE_29_E_09	emb|CAN67310.1|	hypothetical protein [Vitis vinifera]	3E-23
	Contig508	gb|ABE88015.1|	hypothetical protein MtrDRAFT_AC146791g14v2 [Medicago truncatula]	6E-23
	CaF1_JIE_29_E_10	ref|XP_390958.1|	hypothetical protein FG10782.1 [Gibberella zeae PH-1]	6E-23
	Contig139	gb|ABO80202.1|	hypothetical protein MtrDRAFT_AC139707g22v2 [Medicago truncatula]	1E-22
	Contig750	ref|XP_381334.1|	hypothetical protein FG01158.1 [Gibberella zeae PH-1]	2E-22
	CaF1_JIE_03_A_08	emb|CAN77471.1|	hypothetical protein [Vitis vinifera]	2E-22
	CaF1_JIE_07_C_07	emb|CAN78114.1|	hypothetical protein [Vitis vinifera]	2E-22
	Contig131	emb|CAN72414.1|	hypothetical protein [Vitis vinifera]	5E-22
	CaF1_JIE_30_B_06	emb|CAN79350.1|	hypothetical protein [Vitis vinifera]	5E-22
	Contig371	ref|XP_001273877.1|	conserved hypothetical protein [Aspergillus clavatus NRRL 1] gb|EAW12451.1| conserved hypothetical protein [Aspergillus clavatus NRRL 1]	7E-22
	Contig26	ref|XP_389462.1|	hypothetical protein FG09286.1 [Gibberella zeae PH-1]	9E-22
	CaF1_JIE_01_D_06	gb|EAZ40434.1|	hypothetical protein OsJ_023917 [Oryza sativa (japonica cultivar-group)]	9E-22
	Contig963	gb|EAZ38613.1|	hypothetical protein OsJ_022096 [Oryza sativa (japonica cultivar-group)]	1E-21
	CaF1_WIE_29_C_09	emb|CAN78769.1|	hypothetical protein [Vitis vinifera]	1E-21
	Contig427	emb|CAN79067.1|	hypothetical protein [Vitis vinifera]	2E-21
	Contig42	emb|CAJ13713.1|	hypothetical protein [Capsicum chinense]	3E-21
	Contig347	gb|ABE77539.1|	hypothetical protein MtrDRAFT_AC146866g9v2 [Medicago truncatula]	3E-21
	CaF1_WIE_05_G_05	gb|EAY96947.1|	hypothetical protein OsI_018180 [Oryza sativa (indica cultivar-group)]	3E-21
	Contig188	ref|XP_961514.1|	hypothetical protein [Neurospora crassa OR74A] sp|Q9C2P2|RS29_NEUCR 40S ribosomal protein S29 emb|CAC28832.1| probable ribosomal protein S29.e.A, cytosolic [Neurospora crassa] gb|EAA32278.1| hypothetical protein [Neurospora crassa]	7E-21
	Contig410	gb|EAZ07839.1|	hypothetical protein OsI_029071 [Oryza sativa (indica cultivar-group)]	7E-21
	Contig788	emb|CAN70143.1|	hypothetical protein [Vitis vinifera]	1E-20
	CaF1_WIE_26_H_08	emb|CAN79702.1|	hypothetical protein [Vitis vinifera]	1E-20
	CaF1_WIE_36_H_07	emb|CAN81538.1|	hypothetical protein [Vitis vinifera]	1E-20
	CaF1_JIE_29_A_06	emb|CAN81370.1|	hypothetical protein [Vitis vinifera]	8E-20
	CaF1_JIE_06_E_05	emb|CAG28693.1|	hypothetical protein [Gibberella fujikuroi]	1E-19
	Contig322	ref|XP_382064.1|	hypothetical protein FG01888.1 [Gibberella zeae PH-1]	3E-18
	Contig229	ref|XP_384347.1|	hypothetical protein FG04171.1 [Gibberella zeae PH-1]	4E-18
	Contig343	ref|XP_390948.1|	hypothetical protein FG10772.1 [Gibberella zeae PH-1]	4E-18
	Contig688	gb|AAQ09002.1|	hypothetical protein [Phaseolus vulgaris]	2E-17
	CaF1_WIE_09_F_03	emb|CAB95831.1|	hypothetical protein [Cicer arietinum]	2E-17
	Contig529	gb|EAZ25517.1|	hypothetical protein OsJ_009000 [Oryza sativa (japonica cultivar-group)]	1E-16
	Contig8	ref|XP_380605.1|	hypothetical protein FG00429.1 [Gibberella zeae PH-1]	6E-16
	CaF1_WIE_52_F_01	gb|EAZ36326.1|	hypothetical protein OsJ_019809 [Oryza sativa (japonica cultivar-group)]	3E-13
	Contig298	ref|XP_380857.1|	hypothetical protein FG00681.1 [Gibberella zeae PH-1]	6E-12
	CaF1_JIE_04_H_04	ref|XP_381334.1|	hypothetical protein FG01158.1 [Gibberella zeae PH-1]	6E-12
	CaF1_WIE_19_C_04	ref|XP_386326.1|	hypothetical protein FG06150.1 [Gibberella zeae PH-1]	1E-11
	CaF1_JIE_41_A_06	ref|XP_965630.1|	hypothetical protein [Neurospora crassa OR74A] gb|EAA36394.1| hypothetical protein [Neurospora crassa]	3E-11
	CaF1_WIE_51_C_04	dbj|BAE71253.1|	hypothetical protein [Trifolium pratense]	
	CaF1_WIE_49_C_04	gb|EAZ11192.1|	hypothetical protein OsJ_001017 [Oryza sativa (japonica cultivar-group)]	3E-20
	CaF1_JIE_25_E_11	emb|CAN80955.1|	hypothetical protein [Vitis vinifera]	5E-20
	CaF1_WIE_42_F_08	emb|CAN83660.1|	hypothetical protein [Vitis vinifera]	8E-20
	CaF1_JIE_40_B_11	emb|CAN73178.1|	hypothetical protein [Vitis vinifera]	1E-19
	CaF1_WIE_54_C_11	emb|CAN74631.1|	hypothetical protein [Vitis vinifera]	1E-19
	Contig816	gb|ABE85164.1|	hypothetical protein MtrDRAFT_AC119415g11v2 [Medicago truncatula]	5E-19
	Contig861	gb|EAY96565.1|	hypothetical protein OsI_017798 [Oryza sativa (indica cultivar-group)] gb|EAZ32893.1| hypothetical protein OsJ_016376 [Oryza sativa (japonica cultivar-group)]	7E-19
	CaF1_JIE_07_B_10	ref|XP_390378.1|	hypothetical protein FG10202.1 [Gibberella zeae PH-1]	9E-19
	Contig283	emb|CAN62725.1|	hypothetical protein [Vitis vinifera]	2E-18
	CaF1_JIE_32_E_09	gb|ABE92104.1|	hypothetical protein MtrDRAFT_AC144760g11v2 [Medicago truncatula]	2E-18

	CaF1_WIE_41_B_09	gb|ABO84487.1|	hypothetical protein MtrDRAFT_AC174293g6v2 [Medicago truncatula]	3E-18
	CaF1_WIE_37_H_04	dbj|BAD46202.1|	hypothetical protein [Oryza sativa (japonica cultivar-group)]	4E-18
	Contig170	ref|XP_387180.1|	hypothetical protein FG07004.1 [Gibberella zeae PH-1]	8E-18
	CaF1_WIE_54_G_08	dbj|BAD94926.1|	hypothetical protein [Arabidopsis thaliana]	1E-17
	CaF1_WIE_34_E_07	emb|CAN65460.1|	hypothetical protein [Vitis vinifera]	4E-17
	Contig778	gb|EAZ24782.1|	hypothetical protein OsJ_008265 [Oryza sativa (japonica cultivar-group)]	1E-16
	CaF1_JIE_18_B_01	gb|EAZ41723.1|	hypothetical protein OsJ_025206 [Oryza sativa (japonica cultivar-group)]	3E-16
	Contig760	emb|CAN80774.1|	hypothetical protein [Vitis vinifera]	1E-14
	CaF1_WIE_29_B_08	gb|EAZ06542.1|	hypothetical protein OsI_027774 [Oryza sativa (indica cultivar-group)]	1E-14
	Contig291	gb|ABE77464.1|	hypothetical protein MtrDRAFT_AC146865g22v1 [Medicago truncatula]	2E-14
	CaF1_JIE_10_H_07	gb|EAZ43296.1|	hypothetical protein OsJ_026779 [Oryza sativa (japonica cultivar-group)]	7E-14
	Contig829	gb|AAC32158.1|	hypothetical protein [Picea mariana]	1E-13
	CaF1_WIE_56_E_08	gb|AAC32158.1|	hypothetical protein [Picea mariana]	1E-13
	CaF1_JIE_03_G_09	emb|CAN84007.1|	hypothetical protein [Vitis vinifera]	4E-13
	CaF1_WIE_39_A_11	emb|CAB65893.1|	hypotethical protein, homologous to ORF8 of pRiA4 [Agrobacterium rhizogenes]	4E-13
	Contig592	gb|AAG50672.1|AC079829_5	hypothetical protein [Arabidopsis thaliana]	2E-12
	Contig652	gb|ABE84302.1|	hypothetical protein MtrDRAFT_AC146552g12v2 [Medicago truncatula]	3E-12
	Contig245	gb|ABO79784.1|	hypothetical protein MtrDRAFT_AC137825g21v2 [Medicago truncatula]	4E-12
	Contig561	emb|CAN77401.1|	hypothetical protein [Vitis vinifera]	4E-12
	CaF1_WIE_53_H_09	gb|EAY91540.1|	hypothetical protein OsI_012773 [Oryza sativa (indica cultivar-group)]	1E-11
	Contig145	emb|CAN80034.1|	hypothetical protein [Vitis vinifera]	2E-11
	Contig797	emb|CAN64127.1|	hypothetical protein [Vitis vinifera]	2E-18
	Contig106	emb|CAN64127.1|	hypothetical protein [Vitis vinifera]	1E-17
	CaF1_JIE_22_B_02	emb|CAN61038.1|	hypothetical protein [Vitis vinifera]	1E-17
	Contig258	ref|XP_001220685.1|	hypothetical protein CHGG_01464 [Chaetomium globosum CBS 148.51] gb|EAQ93229.1| hypothetical protein CHGG_01464 [Chaetomium globosum CBS 148.51]	3E-18
	CaF1_WIE_05_C_07	emb|CAN75603.1|	hypothetical protein [Vitis vinifera]	2E-20
	CaF1_WIE_47_C_02	emb|CAN66493.1|	hypothetical protein [Vitis vinifera]	2E-20
	Contig507	gb|EAZ42794.1|	hypothetical protein OsJ_026277 [Oryza sativa (japonica cultivar-group)]	6E-20
	CaF1_JIE_41_D_01	ref|XP_391654.1|	hypothetical protein FG11478.1 [Gibberella zeae PH-1]	8E-20
	CaF1_WIE_51_A_06	emb|CAN60708.1|	hypothetical protein [Vitis vinifera]	8E-20
	Contig781	emb|CAN69812.1|	hypothetical protein [Vitis vinifera]	1E-19
	Contig552	gb|EAZ16684.1|	hypothetical protein OsJ_030893 [Oryza sativa (japonica cultivar-group)]	2E-19
	CaF1_WIE_34_C_06	gb|EAZ14922.1|	hypothetical protein OsJ_004747 [Oryza sativa (japonica cultivar-group)]	2E-19
	CaF1_JIE_12_E_10	ref|XP_001219290.1|	hypothetical protein CHGG_00069 [Chaetomium globosum CBS 148.51] gb|EAQ91834.1| hypothetical protein CHGG_00069 [Chaetomium globosum CBS 148.51]	3E-19
	CaF1_WIE_46_A_04	emb|CAN72806.1|	hypothetical protein [Vitis vinifera]	7E-19
	Contig151	ref|XP_388835.1|	hypothetical protein FG08659.1 [Gibberella zeae PH-1]	1E-18
	Contig51	ref|XP_385657.1|	hypothetical protein FG05481.1 [Gibberella zeae PH-1]	2E-18
	CaF1_WIE_30_A_11	gb|EAZ24579.1|	hypothetical protein OsJ_008062 [Oryza sativa (japonica cultivar-group)]	4E-18
	Contig271	emb|CAN73396.1|	hypothetical protein [Vitis vinifera] emb|CAN65595.1| hypothetical protein [Vitis vinifera]	5E-18
	Contig443	emb|CAN65399.1|	hypothetical protein [Vitis vinifera]	7E-18
	Contig288	ref|XP_380574.1|	hypothetical protein FG00398.1 [Gibberella zeae PH-1] sp|Q4IQW0|NOP12_GIBZE Nucleolar protein 12	1E-17
	Contig465	gb|EAZ07445.1|	hypothetical protein OsI_028677 [Oryza sativa	2E-17
	CaF1_JIE_13_C_11	ref|XP_001224160.1|	hypothetical protein CHGG_04946 [Chaetomium globosum CBS 148.51] gb|EAQ88327.1| hypothetical protein CHGG_04946 [Chaetomium globosum CBS 148.51]	4E-17
	CaF1_JIE_29_F_11	gb|EAZ25322.1|	hypothetical protein OsJ_008805 [Oryza sativa (japonica cultivar-group)]	8E-17
	Contig100	gb|EDN11149.1|	hypothetical protein HCAG_07602 [Ajellomyces capsulatus NAm1]	1E-16
	CaF1_JIE_10_D_09	emb|CAN59899.1|	hypothetical protein [Vitis vinifera]	2E-16
	Contig108	emb|CAN66103.1|	hypothetical protein [Vitis vinifera]	4E-16
	CaF1_WIE_47_A_08	emb|CAN63025.1|	hypothetical protein [Vitis vinifera]	4E-16
	Contig878	gb|AAM08880.1|AC113339_26	Hypothetical protein [Oryza sativa]	7E-16

	CaF1_WIE_33_D_10	ref|XP_001245473.1|	hypothetical protein CIMG_04914 [Coccidioides immitis RS] gb|EAS33890.1| hypothetical protein CIMG_04914 [Coccidioides immitis RS]	9E-16
	Contig474	dbj|BAF00195.1|	hypothetical protein [Arabidopsis thaliana]	8E-15
	CaF1_WIE_02_E_05	emb|CAN65557.1|	hypothetical protein [Vitis vinifera]	8E-15
	CaF1_WIE_50_G_07	gb|ABE92424.1|	hypothetical protein MtrDRAFT_AC137986g22v2 [Medicago truncatula] gb|ABN08461.1| hypothetical protein MtrDRAFT_AC157472g30v2 [Medicago truncatula]	8E-15
	CaF1_WIE_10_H_06	emb|CAN81048.1|	hypothetical protein [Vitis vinifera]	1E-14
	CaF1_WIE_32_B_07	emb|CAN65606.1|	hypothetical protein [Vitis vinifera]	2E-14
	Contig635	emb|CAN68644.1|	hypothetical protein [Vitis vinifera]	3E-14
	Contig409	ref|XP_389229.1|	hypothetical protein FG09053.1 [Gibberella zeae PH-1]	9E-14
	CaF1_WIE_07_A_06	emb|CAN77792.1|	hypothetical protein [Vitis vinifera]	1E-13
	CaF1_WIE_10_E_02	gb|ABE94678.1|	hypothetical protein MtrDRAFT_AC126784g9v2 [Medicago truncatula]	1E-13
	CaF1_WIE_14_H_02	emb|CAN62265.1|	hypothetical protein [Vitis vinifera]	1E-13
	CaF1_WIE_35_C_09	emb|CAN83232.1|	hypothetical protein [Vitis vinifera]	1E-13
	CaF1_WIE_44_F_11	ref|XP_381439.1|	hypothetical protein FG01263.1 [Gibberella zeae PH-1]	1E-13
	CaF1_WIE_23_C_03	emb|CAN69767.1|	hypothetical protein [Vitis vinifera]	2E-13
	Contig473	gb|EAZ04460.1|	hypothetical protein OsI_025692 [Oryza sativa (indica cultivar-group)]	1E-12
	Contig914	emb|CAN61918.1|	hypothetical protein [Vitis vinifera]	2E-12
	CaF1_JIE_30_A_02	gb|EAZ35224.1|	hypothetical protein OsJ_018707 [Oryza sativa (japonica cultivar-group)]	3E-12
	CaF1_WIE_36_A_05	emb|CAN65024.1|	hypothetical protein [Vitis vinifera]	3E-12
	CaF1_WIE_19_B_03	ref|XP_386857.1|	hypothetical protein FG06681.1 [Gibberella zeae PH-1]	6E-12
	Contig295	gb|EAY87990.1|	hypothetical protein OsI_009223 [Oryza sativa (indica cultivar-group)]	1E-11
	CaF1_WIE_28_D_11	emb|CAN83660.1|	hypothetical protein [Vitis vinifera]	1E-11
	Contig320	ref|XP_001242204.1|	hypothetical protein CIMG_06100 [Coccidioides immitis RS] gb|EAS30621.1| hypothetical protein CIMG_06100 [Coccidioides immitis RS]	2E-11
	CaF1_WIE_05_H_05	emb|CAN60304.1|	hypothetical protein [Vitis vinifera]	4E-11
	Contig763	emb|CAN68131.1|	hypothetical protein [Vitis vinifera]	1E-10
	CaF1_WIE_43_D_10	emb|CAN69766.1|	hypothetical protein [Vitis vinifera]	1E-10
	Contig685	emb|CAN62058.1|	hypothetical protein [Vitis vinifera]	7E-19
	Contig338	gb|EAZ25517.1|	hypothetical protein OsJ_009000 [Oryza sativa (japonica cultivar-group)]	3E-18
Metabolism	CaF1_WIE_10_B_03	gb|ABE85045.1|	Cyclic peptide transporter [Medicago truncatula]	2E-67
	CaF1_WIE_17_E_05	gb|ABE85045.1|	Cyclic peptide transporter [Medicago truncatula]	3E-63
	CaF1_WIE_28_E_06	gb|ABP03118.1|	Rh-like protein/ammonium transporter [Medicago truncatula]	1E-27
	CaF1_WIE_13_G_01	gb|AAD41024.1|	sucrose transport protein SUT1 [Pisum sativum]	1E-11
	Contig831	gb|AAT45084.1|	proline dehydrogenase [Medicago sativa] gb|AAT45085.1| proline dehydrogenase [Medicago sativa]	1E-137
	Contig844	sp|P34921|G3PC_DIACA	Glyceraldehyde-3-phosphate dehydrogenase, cytosolic	1E-137
	Contig694	gb|AAP33475.1|	polygalacturonase-like protein [Fragaria x ananassa]	1E-119
	Contig267	gb|AAO72533.1|	pyruvate decarboxylase 1 [Lotus corniculatus]	1E-118
	Contig864	gb|ABO77440.1|	S-adenosylmethionine decarboxylase [Medicago sativa subsp. falcata]	1E-114
	Contig714	sp|P49613|METL_PEA	S-adenosylmethionine synthetase 2 (Methionine adenosyltransferase 2) (AdoMet synthetase 2) emb|CAA57581.1| methionine adenosyltransferase [Pisum sativum] gb|AAA58773.1| S-adenosylmethionine synthase	1E-111
	Contig741	gb|ABE84165.2|	5-methyltetrahydropteroyltriglutamate--homocysteine S-methyltransferase; Prismane-like [Medicago truncatula] gb|ABE81639.2| 5-methyltetrahydropteroyltriglutamate--homocysteine S-methyltransferase; Prismane-like [Medicago truncatula]	1E-111
	Contig911	pdb|2P4H|X	Chain X, Crystal Structure Of Vestitone Reductase From Alfalfa (Medicago Sativa L.)	1E-110
	Contig754	dbj|BAE71301.1|	putative arginine decarboxylase [Trifolium pratense]	1E-104
	CaF1_WIE_05_C_05	emb|AJ006024.1|CAR6024	Cicer arietinum mRNA for cysteine synthase, partial	1E-104
	Contig180	gb|ABP49577.1|	oleate desaturase [Caragana korshinskii var. intermedia]	3E-98
	Contig381	emb|CAA08855.1|	copper amine oxidase [Cicer arietinum]	4E-97
	Contig574	sp|O24301|SUS2_PEA	Sucrose synthase 2 (Sucrose-UDP glucosyltransferase 2) emb|CAA04512.1 second sucrose synthase [Pisum sativum]	8E-92
	Contig453	emb|CAC06095.1|	ferredoxin-nitrite reductase [Lotus japonicus]	9E-91
	Contig10	emb|CAA09040.1|	glyceraldehyde 3-phosphate dehydrogenase, cytosolic [Cicer arietinum]	3E-90

	Contig53	ref|NP_173390.1|	pfkB-type carbohydrate kinase family protein [Arabidopsis thaliana] gb|AAF79436.1|AC025808_18 F18O14.35 [Arabidopsis thaliana] gb|AAF98405.1|AC024609_6 Unknown protein [Arabidopsis thaliana] gb|AAO44087.1| At1g19600 [Arabidopsis thaliana] dbj|BAE99744.1| putative ribokinase [Arabidopsis thaliana]	3E-87
	Contig543	gb|AAP33475.1|	polygalacturonase-like protein [Fragaria x ananassa]	7E-87
	Contig555	gb|AAS46231.1|	methionine sulfoxide reductase A [Populus trichocarpa x Populus deltoides]	7E-86
	Contig577	gb|AAB19212.1|	polygalacturonase-inhibiting protein [Malus x domestica] gb|ABA26937.1| polygalacturonase-inhibiting protein [Malus x domestica]	1E-84
	Contig596	dbj|BAB86539.1|	putative aspartate aminotransferase [Oryza sativa (japonica cultivar-group)]	2E-81
	Contig152	gb|AAS46232.1|	methionine sulfoxide reductase A [Populus trichocarpa x Populus deltoides]	8E-81
	Contig488	sp|P13603|ADH1_TRIRP	Alcohol dehydrogenase 1 emb|CAA32934.1| unnamed protein product [Trifolium repens]	1E-80
	CaF1_JIE_41_G_04	pir||S47243	starch phosphorylase (EC 2.4.1.1) isoform L precursor, chloroplast - fava bean	2E-80
	Contig973	emb|CAA52800.1|	T-protein of the glycine decarboxylase complex [Pisum sativum]	3E-80
	Contig454	emb|CAK54360.1|	putative desaturase-like protein [Trifolium repens]	4E-80
	Contig450	gb|AAM65998.1|	putative dTDP-glucose 4-6-dehydratase [Arabidopsis thaliana]	2E-78
	Contig179	gb|ABE93770.1|	Adenylosuccinate synthetase [Medicago truncatula]	6E-78
	Contig678	dbj|BAF31848.1|	nitrite reductase [Fusarium oxysporum]	6E-78
	Contig795	gb|ABE82378.1|	Aldehyde dehydrogenase [Medicago truncatula] gb|ABE91820.1| Aldehyde dehydrogenase [Medicago truncatula]	2E-77
	CaF1_JIE_04_H_05	sp|P81406|GAPN_PEA	NADP-dependent glyceraldehyde-3-phosphate dehydrogenase (Non-phosphorylating glyceraldehyde 3-phosphate dehydrogenase) (Glyceraldehyde-3-phosphate dehydrogenase [NADP+]) (Triosephosphate dehydrogenase) emb|CAA53076.1| glyceraldehyde-3-phosphate dehydrogenase (nonphosphorylating,NADP+) [Pisum sativum] gb|AAO38512.1| non-phosphorylating glyceraldehyde-3-phosphate dehydrogenase [Pisum sativum]	5E-77
	CaF1_WIE_12_F_01	gb|ABQ10186.1|	succinyl-CoA ligase beta subunit [Caragana jubata]	7E-77
	Contig250	gb|ABO84301.1|	Alpha-isopropylmalate/homocitrate synthase [Medicago truncatula]	1E-76
	Contig632	gb|ABP02563.1|	AMP-dependent synthetase and ligase [Medicago truncatula]	1E-76
	CaF1_WIE_36_A_03	ref|NP_189150.1|	QUA1 (QUASIMODO1); polygalacturonate 4-alpha-galacturonosyltransferase/ transferase, transferring glycosyl groups / transferase, transferring hexosyl groups [Arabidopsis thaliana] sp|Q9LSG3|QUA1_ARATH Glycosyltransferase QUASIMODO1 dbj|BAB02072.1| unnamed protein product [Arabidopsis thaliana] gb|AAM20426.1| glycosyl transferase, putative [Arabidopsis thaliana] gb|AAQ56836.1| At3g25140 [Arabidopsis thaliana]	2E-76
	Contig539	gb|ABE89020.1|	Adenosine kinase [Medicago truncatula]	3E-76
	Contig697	gb|ABE83264.1|	Isocitrate dehydrogenase NADP-dependent, plant [Medicago truncatula]	4E-76
	Contig186	gb|ABC87913.1|	polygalacturonase precursor [Glycine max]	1E-75
	Contig308	sp|P21727|TPT_PEA	Triose phosphate/phosphate translocator, chloroplast precursor (cTPT) (p36) (E30) emb|CAA38451.1| chloroplast import receptor p36 [Pisum sativum] emb|CAA48210.1| phosphate translocator [Pisum sativum] prf||1805409A phosphate translocator	1E-75
	Contig627	gb|AAM94349.1|	pyruvate kinase [Glycine max]	1E-74
	CaF1_JIE_14_B_09	gb|AAL74418.2|AF452454_1	ATP sulfurylase [Glycine max]	2E-74
	Contig900	gb|ABE78689.2|	AMP-dependent synthetase and ligase [Medicago truncatula]	3E-74
	CaF1_JIE_15_B_02	gb|ABE81529.1|	Pyridoxal-5-phosphate-dependent enzyme, beta subunit [Medicago truncatula] gb|ABE91493.1| Pyridoxal-5-phosphate-dependent enzyme, beta subunit [Medicago truncatula]	3E-74
	CaF1_WIE_04_A_09	emb|CAA08855.1|	copper amine oxidase [Cicer arietinum]	4E-74
	Contig545	gb|ABN08040.1|	Acyl-coA-binding protein, ACBP; Serine/threonine protein phosphatase, BSU1 [Medicago truncatula]	6E-74
	Contig927	gb|ABO80948.1|	S-adenosylmethionine synthetase [Medicago truncatula]	2E-73
	Contig608	gb|ABE92923.1|	C2; Peptidase, cysteine peptidase active site [Medicago truncatula]	8E-73
	CaF1_WIE_50_C_09	gb|ABE87035.1|	Orn/DAP/Arg decarboxylase 2; Protease-associated PA; Proteinase inhibitor I9, subtilisin propeptide [Medicago truncatula]	8E-73
	CaF1_JIE_01_D_02	emb|CAC10208.1|	cytosolic malate dehydrogenase [Cicer arietinum]	2E-72
	Contig704	gb|ABA86966.1|	triosephosphate isomerase [Glycine max]	3E-72
	Contig255	sp|Q43070|GALE1_PEA	UDP-glucose 4-epimerase (Galactowaldenase) (UDP-galactose 4-epimerase) gb|AAA86532.1| UDP-galactose-4-epimerase	7E-72
	Contig263	gb|ABE82022.1|	Adenylate kinase [Medicago truncatula]	7E-72
	CaF1_WIE_44_D_02	dbj|BAE93460.1|	diacylglycerolacyltransferase-1a [Glycine max]	5E-71

	Contig135	gb|ABE77912.1|	SAM (and some other nucleotide) binding motif [Medicago truncatula] gb|ABE79986.1| SAM (and some other nucleotide) binding motif [Medicago truncatula] gb|ABE83018.1| SAM (and some other nucleotide) binding motif [Medicago truncatula]	4E-70
	Contig755	gb|ABN07990.1|	SAM (and some other nucleotide) binding motif [Medicago truncatula]	8E-20
	Contig243	emb|CAH05011.1|	alpha-dioxygenase [Pisum sativum]	7E-70
	Contig252	gb|ABE88265.1|	Generic methyltransferase [Medicago truncatula] gb|ABN08826.1| Generic methyltransferase [Medicago truncatula]	7E-70
	Contig265	gb|ABE82974.1|	Pyridoxal-5-phosphate-dependent enzyme, beta subunit [Medicago truncatula]	7E-70
	Contig38	sp|P51062|CAPP_PEA	Phosphoenolpyruvate carboxylase (PEPCase) (PEPC) dbj|BAA10902.1| phosphoenolpyruvate carboxylase [Pisum sativum]	1E-69
	CaF1_JIE_19_F_11	sp|P51850|PDC1_PEA	Pyruvate decarboxylase isozyme 1 (PDC) emb|CAA91444.1| pyruvate decarboxylase [Pisum sativum]	1E-69
	CaF1_JIE_26_G_07	dbj|BAF49052.1|	phytoene synthase [Prunus mume]	1E-69
	CaF1_WIE_23_A_04	gb|AAS18240.1|	enolase [Glycine max]	1E-69
	Contig600	gb|AAZ32910.1|	polygalacturonase-like protein [Medicago sativa]	3E-69
	CaF1_WIE_03_C_03	sp|P31023|DLDH_PEA	Dihydrolipoyl dehydrogenase, mitochondrial precursor (Dihydrolipoamide dehydrogenase) (Pyruvate dehydrogenase complex E3 subunit) (PDC-E3) (E3) (Glycine cleavage system L protein) emb|CAA44729.1| lipoamide dehydrogenase [Pisum sativum] emb|CAA45066.2| dihydrolipoamide dehydrogenase [Pisum sativum]	3E-69
	Contig740	gb|ABO15567.1|	cysteine synthase [Glycine max]	2E-43
	Contig249	gb|AAZ20293.1|	isomerase-like protein [Arachis hypogaea]	7E-69
	Contig800	gb|ABO78563.1|	Glycine cleavage system P-protein [Medicago truncatula]	7E-69
	CaF1_WIE_55_H_09	gb|ABO81708.1|	2-oxoglutarate dehydrogenase, E1 component [Medicago truncatula]	2E-68
	Contig724	gb|AAB99755.1|	malate dehydrogenase precursor [Medicago sativa]	3E-68
	Contig213	emb|CAI91291.1|	deoxyhypusine synthase [Crotalaria juncea]	5E-68
	CaF1_WIE_01_A_11	sp|P52780|SYQ_LUPLU	Glutaminyl-tRNA synthetase (Glutamine--tRNA ligase) (GlnRS) emb|CAA62901.1| tRNA-glutamine synthetase [Lupinus luteus]	5E-68
	Contig239	gb|ABE83505.2|	TPP-binding enzymes [Medicago truncatula]	6E-68
	CaF1_WIE_56_D_11	gb|ABE82917.1|	Aldo/keto reductase [Medicago truncatula]	3E-67
	CaF1_WIE_03_H_10	emb|CAA10287.2|	glucan-endo-1,3-beta-glucosidase [Cicer arietinum]	5E-67
	CaF1_WIE_15_A_02	emb|CAC10212.1|	putative mitochondrial glyoxalase II [Cicer arietinum]	1E-66
	Contig734	gb|AAL16064.1|AF420238_1	S-adenosyl-L-methionine synthetase [Dendrobium crumenatum]	4E-66
	Contig617	emb|CAB16844.1|	serine C-palmitoyltransferase like protein [Arabidopsis thaliana] emb|CAB80314.1| serine C-palmitoyltransferase like protein [Arabidopsis thaliana]	6E-66
	CaF1_WIE_35_G_07	gb|ABE93021.1|	Protein prenyltransferase [Medicago truncatula]	1E-65
	CaF1_WIE_30_E_05	emb|CAE12168.2|	formate dehydrogenase [Quercus robur]	2E-65
	CaF1_WIE_12_E_04	gb|ABE80121.1|	Pyruvate kinase [Medicago truncatula] gb|ABE89087.1| Pyruvate kinase [Medicago truncatula]	4E-65
	CaF1_WIE_49_B_08	emb|CAA09588.1|	phosphoenolpyruvate-carboxylase [Vicia faba]	6E-65
	CaF1_WIE_56_F_05	gb|ABE86452.1|	Aminotransferase, class I and II [Medicago truncatula] gb|ABE87161.1| 1-aminocyclopropane-1-carboxylate synthase [Medicago truncatula]	6E-65
	Contig56	gb|AAF08537.1|AF191098_1	nucleoside diphosphate kinase [Pisum sativum]	3E-64
	Contig923	gb|ABB72820.1|	oligouridylate binding protein-like protein [Solanum tuberosum]	5E-64
	CaF1_WIE_34_B_08	sp|P52904|ODPB_PEA	Pyruvate dehydrogenase E1 component subunit beta, mitochondrial precursor (PDHE1-B) gb|AAB01223.1| pyruvate dehydrogenase E1beta	1E-63
	Contig25	gb|AAC49902.1|	diadenosine 5',5'''-P1,P4-tetraphosphate hydrolase [Lupinus angustifolius]	2E-63
	Contig154	gb|AAL11502.1|AF367442_1	NAD-dependent malate dehydrogenase [Prunus persica]	2E-63
	CaF1_JIE_37_B_02	gb|AAX63898.1|	geranylgeranyl reductase [Medicago truncatula]	2E-63
	CaF1_WIE_32_A_02	gb|AAN86061.1|	geranylgeranyl diphosphate synthase [Citrus unshiu]	4E-63
	Contig85	dbj|BAE98181.1|	putative pyruvate decarboxylase [Fusarium oxysporum f. sp. lycopersici]	8E-63
	CaF1_WIE_19_H_10	emb|CAG14979.1|	non-cyanogenic beta-glucosidase [Cicer arietinum]	8E-63
	CaF1_WIE_18_F_11	gb|AAL23710.2|	cellulose synthase [Populus tremuloides]	2E-62
	Contig657	gb|AAD45425.1|	gibberellin 2-oxidase [Pisum sativum]	4E-62
	Contig54	dbj|BAA13032.1|	phosphoribosylanthranilate transferase [Pisum sativum]	1E-61
	Contig148	gb|ABE81243.1|	Malic oxidoreductase [Medicago truncatula]	1E-61
	Contig159	gb|ABE86430.2|	Glycosyl transferase, family 20; Trehalose-phosphatase [Medicago truncatula]	1E-61
	Contig286	sp|P12886|ADH1_PEA	Alcohol dehydrogenase 1 emb|CAA29609.1| alcohol dehydrogenase [Pisum sativum]	2E-61
	CaF1_WIE_27_E_01	sp|P39869|NIA_LOTJA	Nitrate reductase [NADH] (NR) emb|CAA56696.1| nitrate reductase (NADH) [Lotus japonicus]	2E-61
	Contig879	gb|ABE82897.1|	Acetohydroxy acid isomeroreductase [Medicago truncatula]	3E-61
	Contig951	emb|CAD91338.1|	beta-fructofuranosidase [Glycine max]	4E-61

	Contig30	sp|Q03460|GLSN_MEDSA	Glutamate synthase [NADH], chloroplast precursor (NADH-GOGAT) gb|AAB46617.1| NADH-glutamate synthase [Medicago sativa]	7E-61
	Contig163	gb|ABE89232.1|	Inositol polyphosphate related phosphatase [Medicago truncatula]	2E-60
	Contig440	gb|ABE84165.2|	5-methyltetrahydropteroyltriglutamate--homocysteine S-methyltransferase; Prismane-like [Medicago truncatula] gb|ABE81639.2| 5-methyltetrahydropteroyltriglutamate--homocysteine S-methyltransferase; Prismane-like [Medicago truncatula]	2E-60
	Contig899	ref|NP_187341.1|	DIN3/LTA1 (DARK INDUCIBLE 3); alpha-ketoacid dehydrogenase [Arabidopsis thaliana] ref|NP_850527.1| DIN3/LTA1 (DARK INDUCIBLE 3); alpha-ketoacid dehydrogenase [Arabidopsis thaliana] gb|AAF63813.1| branched chain alpha-keto acid dehydrogenase E2 subunit [Arabidopsis thaliana] gb|AAM63444.1| branched chain alpha-keto acid dehydrogenase E2 subunit [Arabidopsis thaliana]	2E-60
	Contig768	dbj|BAE71240.1|	putative cytoplasmic aconitate hydratase [Trifolium pratense]	4E-60
	Contig890	gb|AAL01888.1|AF404404_1	acyl-CoA oxidase [Glycine max]	4E-60
	CaF1_JIE_20_D_06	dbj|BAE99337.1|	putative beta-amylase [Arabidopsis thaliana]	4E-60
	CaF1_WIE_55_G_09	emb|CAJ38375.1|	nucleoside-diphopshate-sugar dehydratase [Plantago major]	4E-60
	CaF1_WIE_37_E_07	gb|ABE85250.1|	3-phosphoshikimate 1-carboxyvinyltransferase [Medicago truncatula]	5E-60
	CaF1_WIE_50_E_10	gb|ABP65665.1|	VTC2-like protein [Actinidia chinensis]	1E-59
	Contig328	emb|CAA05979.1|	adenine nucleotide translocator [Lupinus albus]	4E-59
	Contig936	sp|Q9FVL0|HBL1_MEDSA	Non-symbiotic hemoglobin 1 (MEDsa GLB1) gb|AAG29748.1|AF172172_1 non-symbiotic hemoglobin [Medicago sativa]	4E-59
	Contig403	gb|ABD33005.1|	Membrane bound O-acyl transferase, MBOAT [Medicago truncatula]	3E-58
	Contig134	gb|AAS18240.1|	enolase [Glycine max]	8E-58
	Contig208	emb|CAM35498.1|	spermine synthase [Lotus japonicus]	1E-57
	CaF1_WIE_23_D_07	gb|AAA74456.1|	nitrite reductase	3E-57
	Contig798	gb|AAU44342.1|	monodehydroascorbate reductase II [Pisum sativum]	5E-57
	CaF1_JIE_01_C_09	gb|ABE87035.1|	Orn/DAP/Arg decarboxylase 2; Protease-associated PA; Proteinase inhibitor I9, subtilisin propeptide [Medicago truncatula]	8E-57
	Contig706	gb|ABE94198.1|	NAD-dependent epimerase/dehydratase [Medicago truncatula]	9E-56
	CaF1_WIE_50_D_11	sp|P52904|ODPB_PEA	Pyruvate dehydrogenase E1 component subunit beta, mitochondrial precursor (PDHE1-B) gb|AAB01223.1| pyruvate dehydrogenase E1beta	9E-56
	CaF1_JIE_13_E_03	ref|NP_194214.2|	arginosuccinate synthase family [Arabidopsis thaliana] sp|Q9SZX3|ASSY_ARATH Argininosuccinate synthase, chloroplast precursor (Citrulline--aspartate ligase) gb|AAL38728.1| putative argininosuccinate synthase [Arabidopsis thaliana] gb|AAM14258.1| putative argininosuccinate synthase [Arabidopsis thaliana]	1E-55
	CaF1_WIE_47_A_11	gb|ABE78454.1|	Phosphoesterase, DHHA1 [Medicago truncatula]	3E-55
	Contig582	sp|P47922|NDK1_PEA	Nucleoside diphosphate kinase 1 (Nucleoside diphosphate kinase I) (NDK I) (NDP kinase I) (NDPK I) emb|CAA50511.1| nucleoside-diphosphate kinase [Pisum sativum]	5E-55
	Contig665	emb|CAH55772.1|	putative His-Asp phosphotransfer protein [Pisum sativum]	5E-55
	CaF1_WIE_52_B_01	gb|ABP02258.1|	Glycoside hydrolase, family 3, N-terminal; Glycoside hydrolase, family 3, C terminal [Medicago truncatula]	5E-55
	CaF1_JIE_31_A_04	gb|AAT36331.1|	nitrilase 4A [Lupinus angustifolius] gb|ABB51979.1| nitrilase 4A [Lupinus angustifolius]	1E-54
	Contig813	emb|CAD29733.1|	pectin methylesterase [Sesbania rostrata]	4E-54
	CaF1_WIE_45_E_10	ref|NP_001077888.1|	ALDH6B2 (Aldehyde dehydrogenase 6B2) [Arabidopsis thaliana]	2E-53
	Contig296	gb|AAD55298.1|AC008263_29	F25A4.24 [Arabidopsis thaliana]	2E-52
	Contig304	gb|AAC50039.1|	polynucleotide phosphorylase [Pisum sativum]	2E-52
	CaF1_WIE_26_G_02	gb|AAB46611.1|	aspartate aminotransferase [Medicago sativa]	2E-52
	Contig64	ref|NP_565528.1|	beta-hydroxyacyl-ACP dehydratase, putative [Arabidopsis thaliana] gb|AAD23619.2| putative beta-hydroxyacyl-ACP dehydratase [Arabidopsis thaliana] gb|AAM78110.1| At2g22230/T26C19.11 [Arabidopsis thaliana] gb|AAN72302.1| At2g22230/T26C19.11 [Arabidopsis thaliana]	4E-52
	Contig531	gb|ABB89021.1|	CXE carboxylesterase [Actinidia deliciosa]	4E-52
	CaF1_WIE_21_B_06	sp|Q96551|METK_CATRO	S-adenosylmethionine synthetase 1 (Methionine adenosyltransferase 1) (AdoMet synthetase 1) emb|CAA95856.1| S-adenosyl-L-methionine synthetase 1 [Catharanthus roseus]	4E-52
	CaF1_JIE_07_G_11	gb|AAG28426.1|AF194945_1	cytosolic aconitase [Nicotiana tabacum]	6E-52
	CaF1_WIE_39_C_09	ref|NP_196765.2|	carbon-nitrogen hydrolase family protein [Arabidopsis thaliana] gb|AAL91613.1| AT5g12040/F14F18_210 [Arabidopsis thaliana] gb|AAM10335.1| AT5g12040/F14F18_210 [Arabidopsis thaliana]	6E-52
	Contig870	gb|ABE85996.1|	Glycoside hydrolase, family 1 [Medicago truncatula]	8E-52
	CaF1_JIE_05_F_11	gb|ABE79940.1|	Aldo/keto reductase [Medicago truncatula]	1E-51
	CaF1_JIE_22_A_09	emb|CAK54360.1|	putative desaturase-like protein [Trifolium repens]	2E-51

	CaF1_WIE_34_H_03	ref|NP_174119.1|	pyridoxal-dependent decarboxylase family protein [Arabidopsis thaliana] sp|Q9C509|SGPL_ARATH Sphingosine-1-phosphate lyase (SP-lyase) (SPL) (Sphingosine-1-phosphate aldolase) gb|AAG51494.1|AC069471_25 sphingosine-1-phosphate lyase, putative [Arabidopsis thaliana] gb|AAK25876.1|AF360166_1 putative sphingosine-1-phosphate lyase [Arabidopsis thaliana] gb|AAM44962.1| putative sphingosine-1-phosphate lyase [Arabidopsis thaliana] dbj|BAD13416.1| sphingosine-1-phosphate lyase [Arabidopsis thaliana]	2E-51
	Contig182	ref|NP_193961.2|	carboxylic ester hydrolase [Arabidopsis thaliana]	9E-51
	CaF1_WIE_46_F_09	gb|ABK55756.1|	P450 monooxygenase-like protein [Stylosanthes guianensis]	9E-51
	Contig595	gb|ABE80304.1|	Extradiol ring-cleavage dioxygenase, class III enzyme, subunit B [Medicago truncatula]	2E-50
	Contig459	gb|ABB69781.1|	beta-glucan-binding protein 1 [Medicago truncatula]	8E-50
	CaF1_WIE_03_H_03	sp|P31023|DLDH_PEA	Dihydrolipoyl dehydrogenase, mitochondrial precursor (Dihydrolipoamide dehydrogenase) (Pyruvate dehydrogenase complex E3 subunit) (PDC-E3) (E3) (Glycine cleavage system L protein) emb|CAA44729.1| lipoamide dehydrogenase [Pisum sativum] emb|CAA45066.2| dihydrolipoamide dehydrogenase [Pisum sativum]	8E-50
	CaF1_WIE_28_G_11	gb|ABD28560.1|	Alpha-1,4-glucan-protein synthase (UDP-forming) [Medicago truncatula] gb|ABO78407.1| Alpha-1,4-glucan-protein synthase (UDP-forming) [Medicago truncatula]	1E-49
	CaF1_WIE_25_D_05	sp|Q42908|PMGI_MESCR	2,3-bisphosphoglycerate-independent phosphoglycerate mutase (Phosphoglyceromutase) (BPG-independent PGAM) (PGAM-I) gb|AAA86979.1| phosphoglyceromutase	2E-49
	CaF1_WIE_42_E_04	gb|AAB40396.1|	glycolate oxidase [Mesembryanthemum crystallinum]	5E-49
	CaF1_JIE_42_A_01	ref|NP_181224.1|	xyloglucan:xyloglucosyl transferase, putative / xyloglucan endotransglycosylase, putative / endo-xyloglucan transferase, putative [Arabidopsis thaliana] sp|Q9SJL9|XTH32_ARATH Probable xyloglucan endotransglucosylase/hydrolase protein 32 precursor (At-XTH32) (XTH-32) gb|AAD31572.1| xyloglucan endotransglycosylase, putative [Arabidopsis thaliana] gb|AAK76514.1| putative xyloglucan endo-transglycosylase [Arabidopsis thaliana] gb|AAM66089.1| putative xyloglucan endo-transglycosylase [Arabidopsis thaliana] gb|AAM91780.1| putative xyloglucan endo-transglycosylase [Arabidopsis thaliana]	6E-49
	CaF1_WIE_01_G_06	ref|NP_563771.1|	GAUT6 (Galacturonosyltransferase 6); polygalacturonate 4-alpha-galacturonosyltransferase/ transferase, transferring glycosyl groups [Arabidopsis thaliana] gb|AAF63140.1|AC011001_10 Unknown protein [Arabidopsis thaliana] gb|AAK76574.1| unknown protein [Arabidopsis thaliana] gb|AAM14391.1| unknown protein [Arabidopsis thaliana]	6E-49
	CaF1_WIE_02_C_06	dbj|BAD91082.1|	beta-D-galactosidase [Pyrus pyrifolia]	6E-49
	CaF1_JIE_15_H_01	gb|ABE78289.1|	UDP-glucuronosyl/UDP-glucosyltransferase [Medicago truncatula]	1E-48
	Contig346	gb|AAL85977.1|	putative uridine kinase [Arabidopsis thaliana]	2E-48
	Contig536	emb|CAI56440.1|	S-adenosyl-L-homocysteine hydrolase [Cicer arietinum]	2E-48
	CaF1_WIE_20_B_08	sp|O82043|ILV5_PEA	Ketol-acid reductoisomerase, chloroplast precursor (Acetohydroxy-acid reductoisomerase) (Alpha-keto-beta-hydroxylacil reductoisomerase) emb|CAA76854.1| ketol-acid reductoisomerase [Pisum sativum]	4E-48
	CaF1_JIE_14_H_09	ref|NP_200706.1|	ceramidase family protein [Arabidopsis thaliana]	1E-47
	CaF1_WIE_50_G_05	gb|ABO84404.1|	SAE2 , putative [Medicago truncatula]	2E-47
	Contig907	gb|ABE78365.1|	Metallophosphoesterase; Purple acid phosphatase, N-terminal [Medicago truncatula] gb|ABE86342.1| Metallophosphoesterase; Purple acid phosphatase, N-terminal [Medicago truncatula]	3E-47
	Contig564	gb|AAY34909.1|	enolase [Prunus armeniaca]	6E-47
	Contig933	gb|ABE89160.1|	O-methyltransferase, family 2; Dimerisation [Medicago truncatula]	1E-46
	CaF1_WIE_14_E_03	gb|AAZ79356.1|	aldehyde dehydrogenase [Vitis pseudoreticulata]	1E-46
	CaF1_WIE_12_C_06	ref|NP_565111.1|	nodulin MtN21 family protein [Arabidopsis thaliana] gb|AAK76570.1| putative nodulin protein [Arabidopsis thaliana] gb|AAM14389.1| putative nodulin protein [Arabidopsis thaliana] gb|AAN31815.1| putative nodulin [Arabidopsis thaliana] dbj|BAE98595.1| nodulin-like protein [Arabidopsis thaliana]	3E-46
	CaF1_WIE_28_E_07	emb|CAD31714.1|	fructokinase-like protein [Cicer arietinum]	5E-46
	Contig164	ref|NP_566081.1|	pyridoxine 5'-phosphate oxidase-related [Arabidopsis thaliana] gb|AAD20168.2| expressed protein [Arabidopsis thaliana] gb|AAM63414.1 unknown [Arabidopsis thaliana] gb|ABD94073.1| At2g46580 [Arabidopsis thaliana]	1E-45
	CaF1_WIE_09_F_01	gb|ABE84859.1|	Phosphofructokinase [Medicago truncatula]	2E-45
	Contig865	ref|NP_568117.1|	phosphoadenosine phosphosulfate (PAPS) reductase family protein [Arabidopsis thaliana] emb|CAB83302.1| putative protein [Arabidopsis thaliana]	4E-45

	CaF1_WIE_51_F_07	gb|ABM91070.1|	xyloglucan endotransglycosylase/hydrolase precursor XTH-3 [Populus tremula x Populus tremuloides]	1E-44
	Contig327	gb|ABN08532.1|	Prephenate dehydratase with ACT region [Medicago truncatula]	2E-44
	CaF1_JIE_18_F_02	sp|O04866|ARGD_ALNGL	Acetylornithine aminotransferase, mitochondrial precursor (ACOAT) (Acetylornithine transaminase) (AOTA) emb|CAA69936.1| acetylornithine aminotransferase [Alnus glutinosa]	2E-44
	Contig522	gb|AAM62626.1|	nodulin protein, putative [Arabidopsis thaliana]	1E-43
	CaF1_WIE_43_C_10	emb|CAC43237.1|	lipoxygenase [Sesbania rostrata]	1E-43
	Contig793	gb|ABE84165.2|	5-methyltetrahydropteroyltriglutamate--homocysteine S-methyltransferase; Prismane-like [Medicago truncatula] gb|ABE81639.2| 5-methyltetrahydropteroyltriglutamate--homocysteine S-methyltransferase; Prismane-like [Medicago truncatula]	2E-43
	Contig606	gb|AAG17666.1|	S-adenosylmethionine synthetase [Brassica juncea]	4E-43
	CaF1_WIE_54_E_05	sp|O24301|SUS2_PEA	Sucrose synthase 2 (Sucrose-UDP glucosyltransferase 2) emb|CAA04512.1 second sucrose synthase [Pisum sativum]	5E-43
	Contig718	sp|Q42908|PMGI_MESCR	2,3-bisphosphoglycerate-independent phosphoglycerate mutase (Phosphoglyceromutase) (BPG-independent PGAM) (PGAM-I) gb|AAA86979.1| phosphoglyceromutase	1E-42
	CaF1_WIE_42_A_02	gb|ABE84980.1|	NAD-binding site; Nucleotide sugar epimerase [Medicago truncatula]	2E-42
	Contig192	ref|NP_568756.2|	ceramide kinase-related [Arabidopsis thaliana] gb|AAQ62904.1| ceramide kinase [Arabidopsis thaliana]	2E-41
	CaF1_JIE_34_D_01	gb|ABN08040.1|	Acyl-coA-binding protein, ACBP; Serine/threonine protein phosphatase, BSU1 [Medicago truncatula]	2E-41
	CaF1_WIE_19_A_10	ref|NP_195500.1|	phosphoenolpyruvate carboxykinase (ATP), putative / PEP carboxykinase, putative / PEPCK, putative [Arabidopsis thaliana] sp|Q9T074|PEPCK_ARATH Phosphoenolpyruvate carboxykinase [ATP] (PEP carboxykinase) (Phosphoenolpyruvate carboxylase) (PEPCK) gb|AAK50062.1|AF372922_1 AT4g37870/T28I19_150 [Arabidopsis thaliana] emb|CAB38935.1| phosphoenolpyruvate carboxykinase (ATP)-like protein [Arabidopsis thaliana] emb|CAB80452.1| phosphoenolpyruvate carboxykinase (ATP)-like protein [Arabidopsis thaliana] gb|AAL77736.1| AT4g37870/T28I19_150 [Arabidopsis thaliana] dbj|BAE98480.1| phosphoenolpyruvate carboxykinase (ATP) -like protein [Arabidopsis thaliana]	2E-41
	CaF1_WIE_26_G_10	emb|CAC32462.1|	sucrose synthase isoform 3 [Pisum sativum]	3E-41
	Contig339	gb|ABD33414.1|	Pectinesterase; Pectinesterase inhibitor [Medicago truncatula]	5E-41
	Contig9	gb|ABE80121.1|	Pyruvate kinase [Medicago truncatula] gb|ABE89087.1| Pyruvate kinase [Medicago truncatula]	8E-41
	Contig601	gb|AAD25952.1|AF085717_1	putative callose synthase catalytic subunit [Gossypium hirsutum]	1E-40
	CaF1_JIE_15_A_10	ref|XP_389647.1|	GR78_NEUCR 78 KDA GLUCOSE-REGULATED PROTEIN HOMOLOG PRECURSOR (GRP 78) (IMMUNOGLOBULIN HEAVY CHAIN BINDING PROTEIN HOMOLOG) (BIP) [Gibberella zeae PH-1]	2E-40
	CaF1_JIE_39_C_03	gb|ABE77854.2|	Phospholipid/glycerol acyltransferase [Medicago truncatula]	2E-40
	CaF1_WIE_24_H_04	dbj|BAF42040.1|	pectin methylesterase 3 [Pyrus communis]	2E-40
	CaF1_WIE_54_C_10	emb|CAJ15149.1|	sialyltransferase-like protein [Medicago truncatula] gb|ABE93190.1| Glycosyl transferase, family 29; Immunoglobulin/major histocompatibility complex [Medicago truncatula]	2E-40
	Contig91	dbj|BAF31848.1|	nitrite reductase [Fusarium oxysporum]	3E-40
	Contig783	gb|ABE81662.1|	serine C-palmitoyltransferase like protein [imported] - Arabidopsis thaliana-related [Medicago truncatula]	8E-40
	Contig686	dbj|BAA13683.1|	O-methyltransferase [Glycyrrhiza echinata]	9E-40
	Contig679	ref|XP_390294.1|	ILV5_NEUCR Ketol-acid reductoisomerase, mitochondrial precursor (Acetohydroxy-acid reductoisomerase) (Alpha-keto-beta-hydroxylacil reductoisomerase) [Gibberella zeae PH-1]	2E-39
	Contig858	sp|Q9M4D8|DCAM_VICFA	S-adenosylmethionine decarboxylase proenzyme (AdoMetDC) (SamDC) [Contains: S-adenosylmethionine decarboxylase alpha chain; S-adenosylmethionine decarboxylase beta chain] emb|CAB76966.1| S-adenosylmethionine decarboxylase [Vicia faba]	2E-39
	CaF1_JIE_12_H_08	dbj|BAD94487.1|	phosphoenolpyruvate carboxykinase-like protein [Arabidopsis thaliana]	4E-39
	CaF1_WIE_06_A_01	gb|ABO83962.1|	Glycoside hydrolase, family 1 [Medicago truncatula]	2E-38
	Contig667	ref|NP_567984.1|	pantothenate kinase family protein [Arabidopsis thaliana] gb|AAL32984.1| unknown protein [Arabidopsis thaliana] gb|AAN28872.1| At4g35360/F23E12_80 [Arabidopsis thaliana]	4E-38
	CaF1_WIE_14_D_10	emb|CAA67728.1|	pectinacetylesterase precursor [Vigna radiata var. radiata]	7E-38
	Contig224	sp|O65735|ALF_CICAR	Fructose-bisphosphate aldolase, cytoplasmic isozyme emb|CAA06308.1| cytosolic fructose-1,6-bisphosphate aldolase [Cicer arietinum]	1E-37

	Contig49	emb|CAD70620.1|	branched-chain amino acid aminotransferase-like protein [Cicer arietinum]	2E-37
	CaF1_JIE_28_B_05	gb|ABD28403.1|	Formylmethionine deformylase [Medicago truncatula]	3E-37
	CaF1_WIE_16_H_04	sp|P32289|GLNA_VIGAC	Glutamine synthetase nodule isozyme (Glutamate--ammonia ligase) (GS) gb|AAA34239.1| glutamine synthetase prf||2106409A Gln synthetase	7E-37
	CaF1_WIE_43_H_02	gb|ABE93021.1|	Protein prenyltransferase [Medicago truncatula]	7E-37
	Contig337	ref|XP_387704.1|	KPYK_TRIRE Pyruvate kinase [Gibberella zeae PH-1]	2E-36
	Contig435	sp|P12886|ADH1_PEA	Alcohol dehydrogenase 1 emb|CAA29609.1| alcohol dehydrogenase [Pisum sativum]	3E-36
	CaF1_WIE_16_C_03	dbj|BAD05166.1|	acid phosphatase [Phaseolus vulgaris] dbj|BAD05167.1| acid phosphatase [Phaseolus vulgaris]	4E-36
	Contig21	gb|AAR30118.1|	putative histidine phosphotransferase HPT1p [Gibberella moniliformis]	5E-36
	CaF1_WIE_20_F_02	gb|AAT46998.1|	triosephosphate isomerase [Glycine max]	7E-35
	Contig711	ref|NP_190005.1|	pyridine nucleotide-disulphide oxidoreductase family protein [Arabidopsis thaliana] emb|CAB88427.1| putative protein [Arabidopsis thaliana] gb|AAM61214.1| unknown [Arabidopsis thaliana] gb|AAN72037.1| putative protein [Arabidopsis thaliana] gb|AAP37810.1| At3g44190 [Arabidopsis thaliana]	1E-34
	CaF1_JIE_22_A_06	gb|AAF70823.1|AF154422_1	beta-galactosidase [Lycopersicon esculentum]	3E-34
	Contig612	gb|ABE86055.1|	Amino acid/polyamine transporter II [Medicago truncatula]	8E-34
	CaF1_WIE_01_A_01	emb|CAI56440.1|	S-adenosyl-L-homocysteine hydrolase [Cicer arietinum]	8E-34
	Contig563	emb|CAA63093.1|	alcohol dehydrogenase [Solanum tuberosum]	2E-33
	CaF1_WIE_34_F_01	gb|ABI34093.1|	homocysteine S-methyltransferase [Medicago sativa]	5E-33
	Contig470	pdb|2IX6|A	Chain A, Short Chain Specific Acyl-Coa Oxidase From Arabidopsis Thaliana, Acx4 pdb|2IX6|B Chain B, Short Chain Specific Acyl-Coa Oxidase From Arabidopsis Thaliana, Acx4 pdb|2IX6|C Chain C, Short Chain Specific Acyl-Coa Oxidase From Arabidopsis Thaliana, Acx4 pdb|2IX6|D Chain D, Short Chain Specific Acyl-Coa Oxidase From Arabidopsis Thaliana, Acx4 pdb|2IX6|E Chain E, Short Chain Specific Acy Coa Oxidase From Arabidopsis Thaliana, Acx4 pdb|2IX6|F Chain F, Short Chain Specific Acyl-Coa Oxidase From Arabidopsis Thaliana, Acx4	6E-33
	CaF1_JIE_41_E_01	gb|ABE89691.1|	Pectinesterase [Medicago truncatula]	8E-33
	Contig877	ref|NP_850271.1|	ATGSL08 (GLUCAN SYNTHASE-LIKE 8); 1,3-beta-glucan synthase/ transferase, transferring glycosyl groups [Arabidopsis thaliana]	2E-32
	Contig39	gb|AAS67005.1|	Phosphoenolpyruvate carboxylase [Glycine max]	3E-31
	CaF1_WIE_51_B_10	emb|CAA63598.1|	glyoxysomal beta-ketoacyl-thiolase [Brassica napus]	4E-31
	CaF1_JIE_08_F_02	gb|ABA54868.1|	putative shikimate kinase [Fagus sylvatica]	6E-31
	CaF1_JIE_26_C_07	gb|ABE91874.1|	SAM (and some other nucleotide) binding motif [Medicago truncatula]	6E-31
	CaF1_WIE_14_C_06	ref|NP_001031840.1|	ATDFB (A. THALIANA DHFS-FPGS HOMOLOG B); tetrahydrofolylpolyglutamate synthase [Arabidopsis thaliana]	1E-30
	Contig955	gb|AAA33358.1|	3-hydroxy-3-methylglutaryl-coenzyme A reductase	2E-30
	CaF1_WIE_55_F_07	gb|ABN08775.1|	Glycoside hydrolase, family 19 [Medicago truncatula]	2E-30
	Contig553	gb|AAR29343.1|	allantoinase [Robinia pseudoacacia]	3E-30
	Contig776	gb|AF020273.1|AF020273	Medicago sativa nodule-enhanced malate dehydrogenase precursor (nemdh) mRNA, complete cds	1E-29
	Contig603	emb|CAA10287.2|	glucan-endo-1,3-beta-glucosidase [Cicer arietinum]	2E-29
	CaF1_WIE_53_A_01	gb|ABR15094.1|	ATP citrate lyase alpha subunit [Glycyrrhiza uralensis]	3E-29
	Contig36	gb|ABE89691.1|	Pectinesterase [Medicago truncatula]	1E-28
	Contig76	gb|ABE78629.2|	Phosphoglucose isomerase (PGI) [Medicago truncatula]	3E-28
	Contig3	gb|ABE93138.1|	Amino acid/polyamine transporter II [Medicago truncatula]	5E-28
	Contig767	gb|ABD32718.1|	O-methyltransferase, family 2; Dimerisation [Medicago truncatula]	8E-28
	CaF1_WIE_19_G_01	sp|Q01289|POR_PEA	Protochlorophyllide reductase, chloroplast precursor (PCR) (NADPH-protochlorophyllide oxidoreductase) (POR) emb|CAA44786.1| protochlorophyllide reductase [Pisum sativum]	8E-28
	CaF1_WIE_09_H_01	gb|ABE84165.2|	5-methyltetrahydropteroyltriglutamate--homocysteine S-methyltransferase; Prismane-like [Medicago truncatula] gb|ABE81639.2| 5-methyltetrahydropteroyltriglutamate--homocysteine S-methyltransferase; Prismane-like [Medicago truncatula]	1E-27
	CaF1_WIE_11_C_08	gb|AF172172.1|AF172172	Medicago sativa non-symbiotic hemoglobin (MHB1) mRNA, complete cds	2E-27
	CaF1_WIE_21_F_02	gb|ABO81708.1|	2-oxoglutarate dehydrogenase, E1 component [Medicago truncatula]	2E-27
	CaF1_WIE_40_D_06	gb|AAC28536.1|	putative beta-amylase [Arabidopsis thaliana]	2E-27
	Contig430	dbj|BAB43909.1|	phosphoenolpyruvate carboxykinase [Flaveria pringlei]	3E-27
	Contig660	gb|ABE94198.1|	NAD-dependent epimerase/dehydratase [Medicago truncatula]	3E-27

	CaF1_WIE_51_B_08	sp|Q39366|LGUL_BRAOG	Putative lactoylglutathione lyase (Methylglyoxalase) (Aldoketomutase) (Glyoxalase I) (Glx I) (Ketone-aldehyde mutase) (S-D-lactoylglutathione methylglyoxal lyase) emb|CAA99248.1| unknown [Brassica oleracea var. gemmifera]	4E-27
	Contig544	gb|AAL29212.1|AF354454_1	putative acyl-CoA synthetase [Capsicum annuum]	7E-26
	CaF1_WIE_30_E_04	gb|ABO81167.1|	3-isopropylmalate dehydratase large subunit [Medicago truncatula]	3E-25
	CaF1_WIE_32_C_08	emb|X14826.1|TRADH1	Trifolium repens Adh1 mRNA for alcohol dehydrogenase 1	1E-24
	CaF1_WIE_46_D_11	sp|O24301|SUS2_PEA	Sucrose synthase 2 (Sucrose-UDP glucosyltransferase 2) emb|CAA04512.1 second sucrose synthase [Pisum sativum]	1E-24
	CaF1_WIE_13_C_02	gb|ABO83636.1|	Glycoside hydrolase, clan GH-D; Raffinose synthase [Medicago truncatula]	2E-24
	CaF1_WIE_21_E_07	gb|AAG51802.1|AC067754_18	phosphoglycerate dehydrogenase, putative; 33424-31403 [Arabidopsis thaliana]	2E-24
	Contig495	sp|O65735|ALF_CICAR	Fructose-bisphosphate aldolase, cytoplasmic isozyme emb|CAA06308.1| cytosolic fructose-1,6-bisphosphate aldolase [Cicer arietinum]	3E-24
	Contig817	sp|Q42800|DAPA_SOYBN	Dihydrodipicolinate synthase, chloroplast precursor (DHDPS) gb|AAA73555.1| dihydrodipicolinate synthase	3E-24
	CaF1_WIE_33_A_05	gb|AAL87150.1|AF480496_4	putative histidinol phosphate aminotransferase [Oryza sativa (japonica cultivar-group)]	2E-23
	CaF1_WIE_16_G_04	emb|CAC10208.1|	cytosolic malate dehydrogenase [Cicer arietinum]	5E-23
	Contig352	gb|ABG73467.1|	6-phosphogluconolactonase [Oryza brachyantha]	1E-22
	Contig153	emb|CAA10287.2|	glucan-endo-1,3-beta-glucosidase [Cicer arietinum]	2E-22
	CaF1_JIE_16_B_05	sp|P12886|ADH1_PEA	Alcohol dehydrogenase 1 emb|CAA29609.1| alcohol dehydrogenase [Pisum sativum]	2E-22
	CaF1_JIE_41_B_07	sp|P12886|ADH1_PEA	Alcohol dehydrogenase 1 emb|CAA29609.1| alcohol dehydrogenase [Pisum sativum]	2E-22
	CaF1_WIE_12_G_07	emb|CAC86996.1|	ATP citrate lyase b-subunit [Lupinus albus]	2E-22
	Contig24	gb|ABO80586.1|	Amidase [Medicago truncatula]	3E-22
	Contig918	gb|ABE81243.1|	Malic oxidoreductase [Medicago truncatula]	3E-22
	CaF1_WIE_47_A_05	gb|ABO81445.1|	Glycoside hydrolase, family 1 [Medicago truncatula]	3E-22
	CaF1_WIE_48_C_04	dbj|BAA36291.1|	HMG-CoA reductase [Cucumis melo]	3E-22
	Contig967	gb|AAU07997.1|	phosphoenolpyruvate carboxylase 2; LaPEPC2 [Lupinus albus]	1E-21
	CaF1_JIE_18_C_10	ref|NP_172147.2|	2-oxoglutarate-dependent dioxygenase, putative [Arabidopsis thaliana] sp|Q84MB3|ACCH1_ARATH 1-aminocyclopropane-1-carboxylate oxidase homolog 1 gb|AAP21238.1| At1g06620 [Arabidopsis thaliana] dbj|BAE99663.1| oxidoreductase like protein [Arabidopsis thaliana]	2E-21
	Contig880	gb|AAP83926.1|	transaldolase [Lycopersicon esculentum]	4E-21
	CaF1_WIE_20_D_05	dbj|BAC58013.1|	S-adenosyl-L-methionine: 2,7,4'-trihydroxyisoflavanone 4'-O-methyltransferase [Lotus japonicus]	6E-21
	CaF1_WIE_48_D_02	dbj|BAA36291.1|	HMG-CoA reductase [Cucumis melo]	6E-21
	Contig599	gb|AAB33256.1|	Clostridium pasteurianum ferredoxin homolog [Solanum tuberosum]	7E-21
	CaF1_WIE_49_D_11	ref|NP_178980.1|	malate oxidoreductase, putative [Arabidopsis thaliana] gb|AAD22679.1| malate oxidoreductase (malic enzyme) [Arabidopsis thaliana] gb|AAM14058.1| putative malate oxidoreductase (malic enzyme) [Arabidopsis thaliana] gb|AAN41396.1| putative malate oxidoreductase (malic enzyme) [Arabidopsis thaliana]	7E-21
	CaF1_WIE_54_A_05	gb|AAG31076.1|AF283566_1	sucrose-phosphatase [Medicago truncatula]	1E-20
	Contig682	sp|Q5W915|USP_PEA	UDP-sugar pyrophospharylase (PsUSP) dbj|BAD66876.1| UDP-sugar pyrophospharylase [Pisum sativum]	6E-18
	CaF1_WIE_15_A_04	sp|P51850|PDC1_PEA	Pyruvate decarboxylase isozyme 1 (PDC) emb|CAA91444.1| pyruvate decarboxylase [Pisum sativum]	8E-17
	CaF1_WIE_06_H_07	gb|AAU04405.1|	pyruvate kinase [Citrus limon]	9E-17
	CaF1_WIE_45_C_09	gb|AAL66290.1|AF452450_1	adenosine 5'-phosphosulfate reductase [Glycine max]	1E-14
	Contig95	dbj|BAA76430.1|	fructose-bisphosphate aldolase [Cicer arietinum]	2E-14
	CaF1_JIE_38_F_06	gb|AAK13318.1|AF290958_1	ATP:citrate lyase [Capsicum annuum]	7E-14
	Contig935	gb|ABA03227.1|	glyceraldehyde-3-phosphate dehydrogenase [Populus maximowiczii x Populus nigra]	4E-12
	CaF1_WIE_49_A_10	gb|AF461200.1|	Medicago truncatula nodule-enhanced malate dehydrogenase gene, exons 1 and 2 and partial cds	2E-46
	CaF1_WIE_19_C_10	ref|NP_567126.1|	glycoside hydrolase family 28 protein / polygalacturonase (pectinase) family protein [Arabidopsis thaliana] gb|AAG40344.1|AF324992_1 AT3g62110 [Arabidopsis thaliana] gb|AAN31866.1| unknown protein [Arabidopsis thaliana]	3E-20
	Contig289	gb|ABE94198.1|	NAD-dependent epimerase/dehydratase [Medicago truncatula]	6E-20
	CaF1_WIE_34_C_09	emb|CAA47810.1|	pectinesterase [Pisum sativum]	1E-19
	CaF1_JIE_27_D_10	ref|NP_563662.1|	pectinesterase family protein [Arabidopsis thaliana] gb|AAF02886.1|AC009525_20 Similar to pectinesterases [Arabidopsis thaliana] gb|ABO38784.1| At1g02810 [Arabidopsis thaliana]	2E-19

	CaF1_WIE_32_H_04	ref|NP_563662.1|	pectinesterase family protein [Arabidopsis thaliana] gb|AAF02886.1|AC009525_20 Similar to pectinesterases [Arabidopsis thaliana] gb|ABO38784.1| At1g02810 [Arabidopsis thaliana]	2E-19
	CaF1_WIE_53_C_10	sp|P31239|ACCO_PEA	1-aminocyclopropane-1-carboxylate oxidase (ACC oxidase) (Ethylene-forming enzyme) (EFE) gb|AAA33644.1| 1-aminocyclopropane-1-carboxylate oxidase	3E-18
	CaF1_WIE_10_G_11	gb|ABO84625.1|	Inositol phosphatase/fructose-1,6-bisphosphatase; Inositol monophosphatase [Medicago truncatula]	1E-16
	CaF1_WIE_26_D_06	gb|ABO84434.1|	Esterase/lipase/thioesterase [Medicago truncatula]	1E-16
	Contig382	sp|Q9AT63|PDX1_GINBI	Probable pyridoxal biosynthesis protein PDX1 (Sor-like protein) gb|AAK18310.1|AF344827_1 Sor-like protein [Ginkgo biloba]	5E-15
	CaF1_JIE_17_F_04	gb|ABD28734.1|	UDP-N-acetylglucosamine transferase subunit ALG14, related [Medicago truncatula] gb|ABE92926.1| UDP-N-acetylglucosamine transferase subunit ALG14, related [Medicago truncatula]	1E-13
	CaF1_WIE_56_G_06	gb|ABI34092.1|	cystathionine gamma-synthase [Medicago sativa]	5E-59
	CaF1_JIE_03_H_11	ref|XP_001223273.1|	mannitol-1-phosphate dehydrogenase [Chaetomium globosum CBS 148.51] gb|EAQ87440.1| mannitol-1-phosphate dehydrogenase [Chaetomium globosum CBS 148.51]	1E-17
	Contig515	gb|AAD19957.1|	thiosulfate sulfurtransferase [Datisca glomerata]	2E-19
	Contig804	gb|ABE81243.1|	Malic oxidoreductase [Medicago truncatula]	2E-19
	Contig867	gb|AAG23130.1|	diacylglycerol kinase variant A [Lycopersicon esculentum]	2E-19
	CaF1_JIE_28_D_10	ref|NP_563662.1|	pectinesterase family protein [Arabidopsis thaliana] gb|AAF02886.1|AC009525_20 Similar to pectinesterases [Arabidopsis thaliana] gb|ABO38784.1| At1g02810 [Arabidopsis thaliana]	8E-18
	CaF1_JIE_17_G_04	dbj|BAF44219.1|	polyketide reductase [Lotus japonicus]	9E-11
	Contig746	gb|ABE92907.2|	Peptidase S24, S26A and S26B [Medicago truncatula]	2E-12
Nucleotide binding proteins	CaF1_WIE_52_C_06	gb|ABE89416.1|	TGS; Small GTP-binding protein domain [Medicago truncatula]	4E-79
	Contig790	gb|ABE91892.1|	Ras small GTPase, Ras type; Small GTP-binding protein domain [Medicago truncatula]	4E-72
	Contig751	gb|AAF65513.1|	GTP-binding protein [Capsicum annuum]	3E-63
	CaF1_WIE_46_D_06	gb|ABP03326.1|	Calcium-binding EF-hand; Ras small GTPase, Rho type [Medicago truncatula]	6E-63
	Contig871	gb|ABE78982.1|	Ras small GTPase, Rab type [Medicago truncatula]	2E-58
	CaF1_WIE_23_G_08	emb|CAA98170.1|	RAB7C [Lotus japonicus]	4E-58
	CaF1_JIE_27_C_04	gb|AAC32610.1|	ras-like small monomeric GTP-binding protein [Avena fatua]	9E-51
	Contig499	gb|AAQ72787.1|	putative GTP-binding protein [Cucumis sativus]	2E-46
	CaF1_WIE_14_G_02	emb|CAJ91151.1|	AMP-binding protein [Platanus x acerifolia]	2E-45
	CaF1_WIE_36_A_09	emb|CAA06731.1|	GDP dissociation inhibitor [Cicer arietinum]	1E-44
	CaF1_WIE_10_B_02	emb|CAA55865.1|	Rab [Medicago sativa]	2E-44
	Contig287	emb|CAA98163.1|	RAB1X [Lotus japonicus]	3E-44
	Contig290	dbj|BAA02118.1|	GTP-binding protein [Pisum sativum] emb|CAA82707.1| guanine nucleotide regulatory protein [Vicia faba] prf||2001457K GTP-binding protein prf||2115367A small GTP-binding protein	1E-40
	Contig885	gb|AAL49957.1|	GTP cyclohydrolase I [Lycopersicon esculentum]	3E-34
	CaF1_JIE_32_G_03	emb|CAA06731.1|	GDP dissociation inhibitor [Cicer arietinum]	2E-31
	Contig28	gb|AAT58365.1|	GMPase [Medicago sativa]	8E-31
	Contig503	gb|ABO83561.1|	RabGAP/TBC [Medicago truncatula] gb|ABO83596.1| RabGAP/TBC [Medicago truncatula]	1E-27
	CaF1_WIE_29_B_02	gb|ABE78980.1|	Ras small GTPase, Rab type [Medicago truncatula]	2E-21
	Contig5	dbj|BAA02115.1|	GTP-binding protein [Pisum sativum] prf||2001457G GTP-binding protein	3E-14
Photosynthesis	Contig857	dbj|BAC22609.1|	41 kD chloroplast nucleoid DNA binding protein (CND41) [Nicotiana sylvestris]	3E-30
	Contig81	emb|CAA10284.1|	chlorophyll a/b binding protein [Cicer arietinum]	1E-113
	CaF1_JIE_36_B_01	sp|P06452|ATPI_PEA	Chloroplast ATP synthase a chain precursor (ATPase subunit IV) emb|CAA29349.1| atpI protein [Pisum sativum] emb|CAA27255.1| unnamed protein product [Pisum sativum] prf||1204179A synthase a,ATP	2E-79
	Contig174	gb|ABE84246.1|	Chlorophyll A-B binding protein [Medicago truncatula] gb|ABP02413.1| Chlorophyll A-B binding protein [Medicago truncatula]	4E-66
	Contig904	dbj|BAE71227.1|	putative rubisco subunit binding-protein alpha subunit [Trifolium pratense]	3E-61

	Contig98	ref|NP_563815.1|	PSAO (photosystem I subunit O) [Arabidopsis thaliana] gb|AAK93637.1| unknown protein [Arabidopsis thaliana] gb|AAM14284.1| unknown protein [Arabidopsis thaliana] gb|AAM64918.1| putative 16kDa membrane protein [Arabidopsis thaliana] emb|CAD37939.1| photosystem I subunit O [Arabidopsis thaliana]	1E-53
	Contig785	gb|ABO87610.1|	chloroplast ferredoxin-NADP+ reductase [Pisum sativum]	2E-51
	CaF1_JIE_29_E_03	gb|AAG26305.1|	photosystem II CP47 protein [Trochodendron aralioides]	7E-35
	Contig916	gb|ABE80903.2|	Light chain 3 (LC3) [Medicago truncatula]	3E-33
	Contig227	emb|AJ404642.1|CAR404642	Cicer arietinum partial ORF for NAD-dependent malic enzyme (malate oxidoreductase), exons 1-4	1E-172
	CaF1_WIE_05_B_07	gb|AAM63442.1|	PSI type III chlorophyll a/b-binding protein, putative [Arabidopsis thaliana]	1E-23
	CaF1_WIE_54_B_02	emb|CAA10290.1|	ribulose 1,5-bisphosphate carboxylase small subunit [Cicer arietinum]	7E-27
Post translational modification, protein turn over and chaperons	CaF1_JIE_03_B_09	gb|ABE83254.1|	Peptidase S8 and S53, subtilisin, kexin, sedolisin; Integrase, catalytic region; Zinc finger, CCHC-type; Peptidase aspartic, catalytic [Medicago truncatula]	3E-16
	CaF1_WIE_25_A_02	emb|CAA71762.1|	Ubiquitin activating enzyme E1 [Nicotiana tabacum]	4E-20
	CaF1_WIE_36_F_01	emb|CAE76635.1|	cyclophilin-type peptidyl-prolyl cis-trans isomerase [Cicer arietinum]	5E-12
	Contig931	emb|CAA08906.1|	cysteine proteinase [Cicer arietinum]	0
	Contig735	emb|CAA48140.1|	ubiquitin [Antirrhinum majus]	1E-136
	Contig708	emb|CAA92583.1|	cysteine protease [Pisum sativum]	1E-129
	Contig807	emb|X93220.2|CACG2	Cicer arietinum partial mRNA for cysteine proteinase (cacG2 gene)	1E-110
	CaF1_WIE_21_C_09	emb|AJ299066.1|CAR299066	Cicer arietinum partial mRNA for ubiquitin-conjugating enzyme E2 (ORF1), clone CanUBC-2	3E-93
	CaF1_WIE_44_D_07	emb|AJ009878.1|CAR9878	Cicer arietinum mRNA for cysteine proteinase	1E-91
	Contig226	gb|AAC49013.1|	polyubiquitin containing 7 ubiquitin monomers	7E-80
	Contig925	dbj|BAD24713.1|	protein disufide isomerase-like protein [Glycine max]	3E-79
	Contig786	gb|ABE79257.1|	MIR [Medicago truncatula]	4E-79
	Contig852	ref|NP_200679.1|	ROC7 (rotamase CyP 7); peptidyl-prolyl cis-trans isomerase [Arabidopsis thaliana] sp|Q9SP02|CP20A_ARATH Peptidyl-prolyl cis-trans isomerase CYP20-1 precursor (PPIase CYP20-1) (Rotamase cyclophilin-7) (Cyclophilin of 20 kDa 1) gb|AAF05760.1|AF192490_1 cyclophilin [Arabidopsis thaliana] dbj|BAA97339.1| cyclophilin [Arabidopsis thaliana] gb|AAK82490.1| AT5g58710/mzn1_160 [Arabidopsis thaliana] gb|AAM16173.1| AT5g58710/mzn1_160 [Arabidopsis thaliana] gb|AAM63473.1| cyclophilin ROC7 [Arabidopsis thaliana]	5E-78
	CaF1_WIE_52_B_09	gb|ABD32628.1|	Granulin; Peptidase C1A, papain [Medicago truncatula]	3E-76
	Contig702	gb|ABE86297.1|	Ubiquitin; Apoptosis regulator Bcl-2 protein, BAG [Medicago truncatula]	2E-75
	CaF1_WIE_47_F_03	ref|NP_174675.2|	STT3B (STAUROSPORIN AND TEMPERATURE SENSITIVE 3-LIKE B); oligosaccharyl transferase [Arabidopsis thaliana] gb|AAG12524.1|AC015446_5 Putative integral membrane protein [Arabidopsis thaliana]	3E-74
	CaF1_WIE_52_C_05	gb|ABP03389.1|	20S proteasome, A and B subunits [Medicago truncatula]	4E-74
	Contig510	emb|CAA06853.1|	26S protease regulatory subunit 6 [Cicer arietinum]	2E-71
	CaF1_WIE_44_D_03	gb|ABP02242.1|	Cyclin-like F-box [Medicago truncatula]	3E-71
	Contig491	ref|NP_563657.1|	CLPP5 (NUCLEAR ENCODED CLP PROTEASE 1); endopeptidase Clp [Arabidopsis thaliana] gb|AAG10637.1|AC022521_15 ATP-dependent Clp protease subunit ClpP [Arabidopsis thaliana] emb|CAB43488.1| ATP-dependent Clp protease subunit ClpP [Arabidopsis thaliana] dbj|BAA82065.1| nClpP1 [Arabidopsis thaliana] gb|AAM60971.1| ATP-dependent Clp protease proteolytic subunit ClpP5 [Arabidopsis thaliana] gb|ABD65590.1| At1g02560 [Arabidopsis thaliana]	9E-70
	Contig721	emb|CAA04447.1|	DnaJ-like protein [Medicago sativa] gb|AAC19391.1| DnaJ-like protein MsJ1 [Medicago sativa]	3E-69
	CaF1_JIE_29_H_02	gb|ABD28395.1|	Nucleoporin interacting component; Protein prenyltransferase [Medicago truncatula]	5E-54
	Contig618	gb|ABE79860.1|	Peptidase T1A, proteasome beta-subunit [Medicago truncatula]	1E-67
	Contig634	dbj|BAA25755.1|	vcCyP [Vicia faba]	2E-66
	CaF1_WIE_20_F_05	gb|ABE81707.1|	Esterase/lipase/thioesterase; Peptidase S9B, dipeptidylpeptidase IV N-terminal [Medicago truncatula]	3E-65
	CaF1_WIE_15_F_08	gb|ABE91043.2|	Peptidase C13, legumain [Medicago truncatula] gb|ABP03557.1| Peptidase C13, legumain [Medicago truncatula]	6E-65
	CaF1_WIE_07_E_03	sp|P29828|PDI_MEDSA	Protein disulfide-isomerase precursor (PDI) emb|CAA77575.1| protein disulfide isomerase [Medicago sativa]	2E-62
	CaF1_WIE_27_D_06	gb|ABE87365.2|	Ubiquitin [Medicago truncatula]	9E-62

	CaF1_WIE_18_F_03	gb|ABQ32305.1|	putative ubiquitin-conjugating enzyme [Artemisia annua]	3E-60
	CaF1_WIE_12_D_04	gb|AAM63110.1|	F-box protein AtFBL5 [Arabidopsis thaliana]	1E-59
	CaF1_JIE_15_A_03	gb|ABO83982.1|	protein binding , related [Medicago truncatula]	4E-59
	CaF1_WIE_07_B_07	ref|NP_567972.1|	SLP2 (subtilisin-like serine protease 2); subtilase [Arabidopsis thaliana] emb|CAA17763.1| subtilisin proteinase-like [Arabidopsis thaliana] emb|CAB80215.1| subtilisin proteinase-like [Arabidopsis thaliana] gb|AAL67071.1| putative subtilisin serine protease [Arabidopsis thaliana] gb|AAM19998.1| putative subtilisin serine proteinase [Arabidopsis thaliana]	1E-58
	CaF1_WIE_51_E_07	gb|ABD32889.1|	AAA ATPase; 26S proteasome subunit P45 [Medicago truncatula] gb|ABN08912.1| AAA ATPase; 26S proteasome subunit P45 [Medicago truncatula]	8E-57
	CaF1_WIE_01_H_09	ref|NP_564118.1|	dolichyl-phosphate beta-D-mannosyltransferase, putative / dolichol-phosphate mannosyltransferase, putative / mannose-P-dolichol synthase, putative [Arabidopsis thaliana] gb|AAF80640.1|AC069251_33 F2D10.6 [Arabidopsis thaliana] gb|AAO64810.1| At1g20575 [Arabidopsis thaliana] dbj|BAD43322.1| hypothetical protein [Arabidopsis thaliana] dbj|BAE99453.1| hypothetical protein [Arabidopsis thaliana]	7E-56
	Contig421	gb|ABE85685.1|	Cyclin-like F-box [Medicago truncatula]	2E-54
	Contig150	gb|AAM22748.1|	polyubiquitin 2 [Deschampsia antarctica]	3E-54
	Contig693	gb|ABK42077.1|	ubiquitin extension protein [Capsicum annuum]	4E-54
	CaF1_WIE_18_C_09	gb|ABI31652.1|	26S proteasome regulatory particle non-ATPase subunit 12 [Camellia sinensis]	5E-54
	CaF1_WIE_14_G_09	gb|ABN09080.1|	Heat shock protein DnaJ [Medicago truncatula]	2E-51
	Contig244	gb|ABE84531.1|	Polyadenylate binding protein, human types 1, 2, 3, 4 [Medicago truncatula]	4E-50
	Contig344	emb|CAA51821.1|	ubiquitin conjugating enzyme E2 [Solanum lycopersicum] gb|ABB02644.1| ubiquitin conjugating enzyme E2-like [Solanum tuberosum]	1E-49
	Contig619	emb|CAA04447.1|	DnaJ-like protein [Medicago sativa] gb|AAC19391.1| DnaJ-like protein MsJ1 [Medicago sativa]	6E-49
	Contig156	gb|ABE78557.1|	Oligosaccharyl transferase, STT3 subunit [Medicago truncatula] gb|ABO83793.1| Oligosaccharyl transferase, STT3 subunit [Medicago truncatula]	2E-48
	CaF1_WIE_34_F_10	gb|ABE86426.2|	Heat shock protein 101. (exp=-1; wgp=0; cg=-1; geno, related [Medicago truncatula]	2E-47
	CaF1_WIE_52_G_04	ref|NP_197500.1|	RPT6A (regulatory particle triple-A 6A); ATPase [Arabidopsis thaliana] gb|AAK64142.1| putative 26S proteasome AAA-ATPase subunit RPT6a [Arabidopsis thaliana] gb|AAL85134.1| putative 26S proteasome AAA-ATPase subunit RPT6a [Arabidopsis thaliana] gb|AAM65046.1| 26S proteasome AAA-ATPase subunit RPT6a-like protein [Arabidopsis thaliana] dbj|BAE98371.1| 26S proteasome AAA-ATPase subunit RPT6a -like protein [Arabidopsis thaliana]	5E-47
	CaF1_WIE_42_H_11	gb|ABE81079.1|	PpiC-type peptidyl-prolyl cis-trans isomerase [Medicago truncatula] gb|ABE89370.1| peptidyl-prolyl cis-trans isomerase 1 (ec 5.2.1.8) (rotamase pin1)(ppiase pin1) (mdpin1) [Medicago truncatula]	1E-46
	CaF1_WIE_55_D_03	gb|ABE78927.1|	Peptidase T1A, proteasome beta-subunit [Medicago truncatula]	4E-45
	Contig971	gb|AAT74554.1|	QM family protein [Caragana jubata]	1E-44
	Contig851	gb|ABE90991.1|	Heat shock protein DnaJ, N-terminal; Tetratricopeptide-like helical [Medicago truncatula]	4E-18
	Contig363	gb|AAZ98791.1|	cystatin [Medicago sativa]	4E-43
	Contig392	emb|CAA83548.1|	PsHSC71.0 [Pisum sativum]	1E-42
	Contig57	gb|ABO81056.1|	Ubiquitin-conjugating enzyme, E2 [Medicago truncatula]	3E-42
	CaF1_JIE_35_D_08	gb|ABG22120.1|	polyprotein [Cynara scolymus]	4E-42
	Contig325	gb|ABE77684.1|	PpiC-type peptidyl-prolyl cis-trans isomerase; Rhodanese-like [Medicago truncatula] gb|ABE82359.1| PpiC-type peptidyl-prolyl cis-trans isomerase; Rhodanese-like [Medicago truncatula]	4E-41
	Contig663	gb|ABE78148.1|	Heat shock protein DnaJ [Medicago truncatula] gb|ABE83817.1| Heat shock protein DnaJ [Medicago truncatula] gb|ABP02364.1| Heat shock protein DnaJ [Medicago truncatula]	8E-41
	CaF1_JIE_14_D_03	ref|NP_197749.2|	DNAJ heat shock N-terminal domain-containing protein [Arabidopsis thaliana] ref|NP_001031930.1| heat shock protein binding / nucleotide binding / unfolded protein binding [Arabidopsis thaliana] gb|AAM91576.1| putative protein [Arabidopsis thaliana] gb|AAP13437.1| At5g23590 [Arabidopsis thaliana]	1E-40
	CaF1_JIE_07_H_11	gb|ABO79438.1|	Peptidase aspartic, active site [Medicago truncatula]	2E-40
	Contig557	gb|ABD28715.1|	Peptidase S24, S26A and S26B [Medicago truncatula]	3E-40
	CaF1_WIE_49_F_03	sp|Q9SXU1|PSA7_CICAR	Proteasome subunit alpha type 7 (20S proteasome alpha subunit D) (20S proteasome subunit alpha-4) dbj|BAA76428.1| multicatalytic endopeptidase complex [Cicer arietinum]	5E-39
	Contig199	gb|AAM64316.1|	multicatalytic endopeptidase complex, proteasome precursor, beta subunit [Arabidopsis thaliana]	1E-36

	Contig105	gb|ABE92718.1|	Proteasome component region PCI [Medicago truncatula] gb|ABO84469.1| Proteasome component region PCI [Medicago truncatula]	2E-36
	Contig968	sp|Q41649|FKB15_VICFA	FK506-binding protein 2 precursor (Peptidyl-prolyl cis-trans isomerase) (PPIase) (Rotamase) (15 kDa FKBP) (FKBP-15) gb|AAC49392.1| immunophilin precursor	2E-36
	CaF1_WIE_03_E_02	gb|ABE93328.1|	Peptidase S10, serine carboxypeptidase [Medicago truncatula]	6E-36
	Contig664	gb|ABE78148.1|	Heat shock protein DnaJ [Medicago truncatula] gb|ABE83817.1| Heat shock protein DnaJ [Medicago truncatula] gb|ABP02364.1| Heat shock protein DnaJ [Medicago truncatula]	2E-35
	CaF1_WIE_19_B_08	gb|ABC75374.1|	SGS; HSP20-like chaperone [Medicago truncatula]	9E-35
	Contig130	gb|ABO82668.1|	Proteinase inhibitor I25, cystatin [Medicago truncatula]	3E-32
	Contig874	gb|ABD32628.1|	Granulin; Peptidase C1A, papain [Medicago truncatula]	8E-31
	CaF1_WIE_47_H_03	gb|ABE85038.2|	Peptidase S1 and S6, chymotrypsin/Hap; Immunoglobulin/major histocompatibility complex; AAA ATPase, central region; SMAD/FHA [Medicago truncatula]	3E-30
	Contig730	gb|ABE93325.2|	Peptidase S10, serine carboxypeptidase [Medicago truncatula]	6E-30
	Contig481	gb|ABD32628.1|	Granulin; Peptidase C1A, papain [Medicago truncatula]	1E-29
	CaF1_WIE_46_B_03	gb|ABE80335.1|	Kunitz inhibitor ST1-like [Medicago truncatula]	2E-27
	Contig506	gb|ABE79095.2|	Sec61beta [Medicago truncatula]	5E-27
	CaF1_WIE_53_D_07	ref|NP_001077535.1|	UBX domain-containing protein [Arabidopsis thaliana] ref|NP_001077536.1| UBX domain-containing protein [Arabidopsis thaliana]	5E-27
	Contig538	gb|AAF68120.1|AC010793_15	F20B17.14 [Arabidopsis thaliana] gb|AAG52249.1|AC011717_17 putative aspartyl protease; 105611-106921 [Arabidopsis thaliana]	3E-26
	Contig655	gb|AAY54007.1|	subtilisin-like protease [Arachis hypogaea]	3E-26
	Contig794	gb|ABE91564.1|	Ubiquitin-conjugating enzyme, E2 [Medicago truncatula]	6E-26
	CaF1_WIE_14_C_09	emb|AJ635223.1|	Pisum sativum mRNA for ftsH-like protease (ftsh4 gene)	2E-24
	Contig687	gb|ABE89792.1|	Peptidase S10, serine carboxypeptidase [Medicago truncatula]	2E-23
	CaF1_WIE_41_A_05	gb|ABE85043.1|	HSP20-like chaperone [Medicago truncatula]	2E-23
	CaF1_WIE_18_D_11	gb|ABN05716.1|	Chaperone DnaK [Medicago truncatula]	6E-23
	CaF1_WIE_36_D_01	gb|ABE78703.1|	Heat shock protein DnaJ [Medicago truncatula]	2E-22
	CaF1_WIE_36_H_04	gb|ABE79819.2|	GroEL-like chaperone, ATPase [Medicago truncatula]	3E-22
	Contig103	gb|AAQ18141.1|	poly(A)-binding protein C-terminal interacting protein 6 [Cucumis sativus]	6E-22
	Contig644	gb|ABD33216.1|	Peptidase A1, pepsin [Medicago truncatula]	3E-21
	CaF1_JIE_05_B_05	ref|NP_189124.1|	PRT1 (PROTEOLYSIS 1); ubiquitin-protein ligase [Arabidopsis thaliana] sp|Q8LBL5|PRT1_ARATH E3 ubiquitin-protein ligase PRT1 (Proteolysis 1 protein) emb|CAA11891.1| PRT1 [Arabidopsis thaliana] emb|CAA11892.1| PRT1 [Arabidopsis thaliana] dbj|BAB02890.1| PRT1 protein [Arabidopsis thaliana] gb|AAL87280.1| putative PRT1 protein [Arabidopsis thaliana] gb|AAM45125.1| putative PRT1 protein [Arabidopsis thaliana]	3E-21
Replication, recombination and repair	Contig391	gb|ABE94111.1|	DNA repair protein RadA; Peptidase M41, FtsH [Medicago truncatula]	2E-50
	Contig189	gb|AAT58770.1|	putative polyprotein [Oryza sativa (japonica cultivar-group)]	1E-24
	CaF1_JIE_42_G_01	gb|AAT38758.1|	Putative gag-pol polyprotein, identical [Solanum demissum]	9E-22
	Contig310	gb|AAK54302.1|AC034258_20	putative helicase [Oryza sativa (japonica cultivar-group)] gb|AAP54108.1| AT hook motif-containing protein, putative [Oryza sativa (japonica cultivar-group)]	4E-16
RNA processing and modification	Contig176	ref|NP_851141.1|	RNA recognition motif (RRM)-containing protein [Arabidopsis thaliana] gb|AAK91419.1| AT5g46250/MPL12_3 [Arabidopsis thaliana] gb|AAN18157.1| At5g46250/MPL12_3 [Arabidopsis thaliana]	6E-20
	CaF1_WIE_34_G_06	ref|NP_001056664.1|	Os06g0127500 [Oryza sativa (japonica cultivar-group)] dbj|BAC24834.1| putative RNA-binding protein [Oryza sativa (japonica cultivar-group)] dbj|BAD67747.1| putative RNA-binding protein [Oryza sativa (japonica cultivar-group)] dbj|BAF18578.1| Os06g0127500 [Oryza sativa (japonica cultivar-group)] gb|EAZ35678.1| hypothetical protein OsJ_019161 [Oryza sativa (japonica cultivar-group)]	1E-14
	Contig615	gb|ABO79609.1|	Ataxin-2, N-terminal; Like-Sm ribonucleoprotein-related, core [Medicago truncatula] gb|ABO81296.1| Ataxin-2, N-terminal; Like-Sm ribonucleoprotein-related, core [Medicago truncatula]	1E-13
	Contig779	gb|AAN74636.1|	DEAD box RNA helicase [Pisum sativum]	1E-89
	CaF1_WIE_48_H_11	gb|ABE89510.1|	U2 auxiliary factor small subunit [Medicago truncatula]	1E-72

	Contig47	gb|AAN74635.1|	DEAD box RNA helicase [Pisum sativum] gb|AAR97917.1| DEAD box RNA helicase [Pisum sativum]	4E-64
	Contig744	gb|ABP03363.1|	RNA-binding region RNP-1 (RNA recognition motif) [Medicago truncatula]	2E-58
	CaF1_JIE_40_H_08	gb|ABE88390.1|	Pre-mRNA processing ribonucleoprotein, binding region; NOSIC [Medicago truncatula]	3E-56
	CaF1_JIE_12_D_06	gb|ABE79750.1|	AAA ATPase, central region; DEAD/DEAH box helicase, N-terminal [Medicago truncatula]	3E-45
	CaF1_WIE_08_B_04	gb|ABE90187.1|	Splicing factor 3B subunit 10 [Medicago truncatula]	5E-44
	Contig321	gb|ABE90931.1|	Sm-like protein [imported] - Arabidopsis thaliana [Medicago truncatula]	4E-43
	CaF1_WIE_43_F_10	emb|Y16672.1|MSY16672	Medicago sativa mRNA for putative arginine/serine-rich splicing factor, (sclp gene)	7E-39
	CaF1_WIE_27_G_10	gb|ABP03363.1|	RNA-binding region RNP-1 (RNA recognition motif) [Medicago truncatula]	1E-37
	Contig883	gb|ABE82737.1|	RNA-binding region RNP-1 (RNA recognition motif) [Medicago truncatula]	1E-36
	Contig235	gb|AAN74635.1|	DEAD box RNA helicase [Pisum sativum] gb|AAR97917.1| DEAD box RNA helicase [Pisum sativum]	1E-35
	CaF1_WIE_10_C_04	gb|ABE89041.1|	RNA-binding region RNP-1 (RNA recognition motif) [Medicago truncatula]	2E-33
	Contig206	gb|ABE82737.1|	RNA-binding region RNP-1 (RNA recognition motif) [Medicago truncatula]	4E-33
	CaF1_JIE_03_C_03	gb|ABD33394.2|	FAR1; Polynucleotidyl transferase, Ribonuclease H fold [Medicago truncatula]	3E-29
	CaF1_JIE_28_F_10	gb|AAF75791.1|AF271892_1	DEAD box protein P68 [Pisum sativum]	3E-29
	CaF1_WIE_27_H_10	gb|ABO82746.1|	RNA-binding region RNP-1 (RNA recognition motif) [Medicago truncatula]	3E-25
	Contig562	ref|NP_176514.1|	DEAD box RNA helicase, putative [Arabidopsis thaliana] sp|Q9C8S9|RH48_ARATH Probable DEAD-box ATP-dependent RNA helicase 48 gb|AAG52143.1|AC022355_4 putative RNA helicase; 42376-45543 [Arabidopsis thaliana]	3E-21
	Contig17	gb|ABE93398.1|	RNA-binding region RNP-1 (RNA recognition motif); HMG-I and HMG-Y, DNA-binding [Medicago truncatula]	2E-11
Secondary metabolism	Contig482	sp|Q9SML4|CHS1_CICAR	Chalcone synthase 1 (Naringenin-chalcone synthase 1) emb|CAA10190.1| chalcone synthase [Cicer arietinum]	1E-157
	Contig957	sp|P28012|CFI1_MEDSA	Chalcone--flavonone isomerase 1 (Chalcone isomerase 1) pdb|1EYP|A Chain A, Chalcone Isomerase pdb|1EYP|B Chain B, Chalcone Isomerase pdb|1EYQ|A Chain A, Chalcone Isomerase And Naringenin pdb|1EYQ|B Chain B, Chalcone Isomerase And Naringenin pdb|1JEP|A Chain A, Chalcone Isomerase Complexed With 4'-Hydroxyflavanone pdb|1JEP|B Chain B, Chalcone Isomerase Complexed With 4'-Hydroxyflavanone pdb|1FM8|A Chain A, Chalcone Isomerase Complexed With 5,4'-Dideoxyflavanone pdb|1FM8|B Chain B, Chalcone Isomerase Complexed With 5,4'-Dideoxyflavanone pdb|1FM7|A Chain A, Chalcone Isomerase Complexed With 5-Deoxyflavanone pdb|1FM7|B Chain B, Chalcone Isomerase Complexed With 5-Deoxyflavanone gb|AAB41524.1| chalcone isomerase [Medicago sativa]	1E-103
	CaF1_WIE_43_D_11	gb|AAT94364.1|	chalcone isomerase 1B2 [Glycine max]	4E-53
	Contig780	emb|CAA10131.1|	chalcone synthase [Cicer arietinum]	3E-51
	CaF1_JIE_37_F_08	emb|CAA10131.1|	chalcone synthase [Cicer arietinum]	1E-48
	Contig738	dbj|BAA76417.1|	chalcone reductase [Cicer arietinum]	8E-24
	Contig429	sp|O81928|TCMO_CICAR	Trans-cinnamate 4-monooxygenase (Cinnamic acid 4-hydroxylase) (CA4H) (C4H) (P450C4H) (Cytochrome P450 73) emb|CAA07519.2| trans-cinnamic 4-monooxygenase [Cicer arietinum]	1E-159
	Contig323	gb|AAZ29733.1|	phenylalanine ammonia lyase [Trifolium pratense]	1E-111
	Contig483	sp|Q96423|TCMO_GLYEC	Trans-cinnamate 4-monooxygenase (Cinnamic acid 4-hydroxylase) (CA4H) (C4H) (P450C4H) (Cytochrome P450 73) dbj|BAA13414.1| cytochrome P450 (CYP73A14) [Glycyrrhiza echinata]	2E-66
	Contig719	gb|AAQ20041.1|	isoflavone 3'-hydroxylase [Medicago truncatula]	2E-62
	Contig616	gb|AAZ29733.1|	phenylalanine ammonia lyase [Trifolium pratense]	9E-61
	CaF1_WIE_53_B_09	dbj|BAF34844.1|	pterocarpan reductase [Lotus japonicus]	5E-60
	Contig393	gb|ABC94943.1|	squalene epoxidase [Medicago sativa]	8E-60
	CaF1_JIE_27_B_02	gb|AAN31890.1|	putative sterol-C-methyltransferase [Arabidopsis thaliana]	3E-58
	Contig591	gb|AAQ20041.1|	isoflavone 3'-hydroxylase [Medicago truncatula]	9E-56
	Contig365	gb|ABC94943.1|	squalene epoxidase [Medicago sativa]	7E-54
	Contig814	gb|AAT94362.1|	putative chalcone isomerase 4 [Glycine max]	7E-54
	CaF1_JIE_42_G_06	sp|Q00016|IFR_CICAR	Isoflavone reductase (IFR) (2'-hydroxyisoflavone reductase) (NADPH:isoflavone oxidoreductase) emb|CAA43167.1| NADPH:isoflavone oxidoreductase [Cicer arietinum]	1E-45

	Contig178	gb|ABC94943.1|	squalene epoxidase [Medicago sativa]	2E-44
	CaF1_JIE_38_C_11	gb|AAY86360.1|	cinnamoyl-CoA reductase [Acacia mangium x Acacia auriculiformis]	3E-43
	Contig550	gb|AAM65672.1|	4-coumarate-CoA ligase-like protein [Arabidopsis thaliana]	7E-35
	Contig215	gb|L46857.1|ALFCAD1B	Medicago sativa cinnamyl-alcohol dehydrogenase (cad1) mRNA, partial cds	1E-32
	CaF1_WIE_24_C_10	ref|NP_200750.1|	oxysterol-binding family protein [Arabidopsis thaliana] gb|AAK96664.1| oxysterol-binding protein [Arabidopsis thaliana] gb|AAN15434.1| oxysterol binding protein [Arabidopsis thaliana]	1E-32
	Contig232	gb|ABO93014.1|	putative sterol desaturase [Solanum tuberosum]	4E-30
	Contig743	gb|AAB94584.1|	CYP71A10 [Glycine max]	8E-28
	Contig673	sp|Q00016|	IFR_CICAR Isoflavone reductase (IFR) (2'-hydroxyisoflavone reductase)	7E-35
Signaling	Contig425	sp|P42654|1433B_VICFA	14-3-3-like protein B (VFA-1433B) emb|CAA88416.1| 14-3-3 brain protein homolog [Vicia faba]	8E-77
	Contig568	dbj|BAB10271.1|	ankyrin-like protein [Arabidopsis thaliana]	1E-67
	Contig133	gb|AAF64040.1|AF228501_1	14-3-3-like protein [Glycine max]	5E-65
	Contig947	gb|ABQ95992.1|	14-3-3-like protein [Cicer arietinum] gb|ABQ95994.1| 14-3-3-like protein [Cicer arietinum]	4E-64
	CaF1_WIE_13_A_05	gb|AAV50005.1|	14-3-3 family protein [Malus x domestica]	6E-55
	CaF1_WIE_55_G_01	gb|ABP02712.1|	14-3-3 protein [Medicago truncatula]	1E-53
	Contig527	gb|ABQ95992.1|	14-3-3-like protein [Cicer arietinum] gb|ABQ95994.1| 14-3-3-like protein [Cicer arietinum]	3E-29
	CaF1_WIE_05_E_05	gb|ABE82023.1|	Protein kinase [Medicago truncatula]	6E-20
	CaF1_WIE_50_G_06	ref|XP_001267009.1|	calmodulin [Neosartorya fischeri NRRL 181] gb|EAW25112.1| calmodulin [Neosartorya fischeri NRRL 181]	1E-16
	CaF1_WIE_46_H_04	gb|ABO78866.1|	WD40-like [Medicago truncatula]	5E-16
	Contig815	gb|ABE90033.1|	Protein kinase [Medicago truncatula]	4E-13
	CaF1_JIE_29_F_07	gb|ABO78866.1|	WD40-like [Medicago truncatula]	1E-12
	Contig332	sp|Q53IP3|MBF1_GIBFU	Multiprotein-bridging factor 1 emb|CAG28684.1| multiprotein bridging factor [Gibberella fujikuroi]	2E-11
	Contig86	gb|ABE83899.1|	Serine/threonine protein kinase, active site [Medicago truncatula] gb|ABE89881.1| Serine/threonine protein kinase, active site [Medicago truncatula]	2E-86
	CaF1_WIE_47_C_03	gb|ABE93337.1|	Protein kinase [Medicago truncatula]	7E-77
	CaF1_WIE_36_C_09	gb|AAP72282.2|	calcium-dependent calmodulin-independent protein kinase isoform 2 [Cicer arietinum]	1E-76
	CaF1_WIE_53_H_05	gb|ABE84183.2|	Protein kinase [Medicago truncatula]	4E-74
	CaF1_JIE_03_A_05	gb|ABE88922.1|	Serine/threonine protein kinase, active site [Medicago truncatula]	1E-71
	Contig377	gb|AAW31901.1|	calcium-dependent/calmodulin-independent protein kinase isoform 3 [Cicer arietinum]	4E-71
	Contig670	gb|AAL17948.1|	phosphoinositide-specific phospholipase C [Medicago truncatula] gb|ABE91697.1| Phosphoinositide-specific phospholipase C (PLC) [Medicago truncatula]	4E-71
	Contig662	gb|ABE93914.1|	Protein phosphatase 2C [Medicago truncatula]	1E-68
	Contig161	gb|ABE86996.1|	Protein kinase [Medicago truncatula]	5E-65
	Contig194	gb|AAK11734.1|	serine/threonine/tyrosine kinase [Arachis hypogaea]	8E-65
	Contig752	ref|NP_568466.1|	CIPK25 (CBL-INTERACTING PROTEIN KINASE 25); kinase [Arabidopsis thaliana] gb|AAL41008.1|AF448226_1 CBL-interacting protein kinase CIPK25 [Arabidopsis thaliana]	2E-64
	CaF1_WIE_51_C_11	gb|ABE78903.1|	Protein kinase [Medicago truncatula] gb|ABE81974.1| Protein kinase [Medicago truncatula]	7E-64
	CaF1_JIE_20_A_11	ref|NP_195483.1|	HHP4 (heptahelical protein 4); receptor [Arabidopsis thaliana] gb|AAK25883.1|AF360173_1 unknown protein [Arabidopsis thaliana] emb|CAB38307.1| putative protein [Arabidopsis thaliana] emb|CAB80433.1| putative protein [Arabidopsis thaliana] gb|AAL07197.1| unknown protein [Arabidopsis thaliana]	1E-63
	CaF1_WIE_21_H_09	gb|ABF13308.1|	PP2c [Phaseolus vulgaris]	2E-60
	CaF1_WIE_08_F_06	ref|NP_566383.1|	protein phosphatase-related [Arabidopsis thaliana] gb|AAF01527.1|AC009991_23 unknown protein [Arabidopsis thaliana] gb|ABD19691.1| At3g10940 [Arabidopsis thaliana] dbj|BAE98389.1| hypothetical protein [Arabidopsis thaliana]	8E-60
	Contig842	gb|ABE78306.1|	IQ calmodulin-binding region [Medicago truncatula]	2E-59
	Contig928	ref|NP_565408.1|	protein kinase, putative [Arabidopsis thaliana] gb|AAK43904.1|AF370585_1 putative protein kinase [Arabidopsis thaliana] gb|AAK83605.1| At2g17220/T23A1.8 [Arabidopsis thaliana] gb|AAD25140.2| putative protein kinase [Arabidopsis thaliana] gb|AAN31120.1| At2g17220/T23A1.8 [Arabidopsis thaliana]	5E-57
	CaF1_WIE_02_E_09	sp|P48488|PP1_MEDVA	Serine/threonine-protein phosphatase PP1 emb|CAA56766.1| potentially catalitic subunit of the ser /thr protein phosphatase 1 [Medicago sativa subsp. x varia]	6E-57
	CaF1_JIE_15_B_11	gb|ABE92129.1|	Protein kinase [Medicago truncatula]	8E-57

	CaF1_WIE_41_C_08	gb|ABE93295.1|	Leucine-rich repeat; Leucine-rich repeat, cysteine-containing subtype [Medicago truncatula]	1E-56
	Contig406	ref|NP_200932.2|	protein binding [Arabidopsis thaliana] dbj|BAB08479.1| leucine-rich repeat disease resistance protein-like [Arabidopsis thaliana] gb|AAM91553.1| Cf-5 disease resistance protein-like [Arabidopsis thaliana] gb|AAN15323.1| Cf-5 disease resistance protein-like [Arabidopsis thaliana]	1E-59
	CaF1_WIE_23_B_07	gb|ABE85435.1|	Protein kinase; NAF [Medicago truncatula] gb|ABO83915.1| Protein kinase; NAF [Medicago truncatula] gb|ABO84280.1| Protein kinase; NAF [Medicago truncatula]	2E-56
	Contig668	gb|AAK11734.1|	serine/threonine/tyrosine kinase [Arachis hypogaea]	5E-56
	CaF1_WIE_24_F_06	gb|AAK11734.1|	serine/threonine/tyrosine kinase [Arachis hypogaea]	5E-56
	Contig480	gb|ABE90681.1|	Protein kinase; GroEL-like chaperone, ATPase [Medicago truncatula]	2E-55
	CaF1_WIE_41_G_03	gb|ABK06434.1|	flag-tagged protein kinase domain of putative mitogen-activated protein kinase kinase kinase [synthetic construct]	3E-54
	CaF1_JIE_09_C_10	gb|AAN03470.1|	RING-H2 finger protein [Glycine max]	5E-51
	Contig89	emb|CAC36428.1|	mitogen activated protein kinase [Gibberella fujikuroi]	2E-50
	CaF1_JIE_12_E_08	dbj|BAC07504.2|	receptor-like protein kinase [Nicotiana tabacum]	2E-50
	Contig20	dbj|BAE71262.1|	putative protein kinase APK1A [Trifolium pratense]	5E-49
	CaF1_JIE_03_B_10	gb|ABE88922.1|	Serine/threonine protein kinase, active site [Medicago truncatula]	1E-47
	Contig926	gb|ABE92804.1|	Curculin-like (mannose-binding) lectin [Medicago truncatula]	2E-47
	CaF1_WIE_09_E_11	gb|ABE79363.1|	Protein kinase [Medicago truncatula]	2E-47
	Contig234	gb|ABP02851.1|	Calcium-binding EF-hand [Medicago truncatula]	5E-47
	Contig903	gb|ABD28527.1|	Protein kinase [Medicago truncatula] gb|ABE81653.1| Protein kinase [Medicago truncatula]	1E-45
	Contig228	dbj|BAE71265.1|	putative serine/threonine protein kinase-like protein [Trifolium pratense] dbj|BAE71272.1| putative serine/threonine protein kinase-like protein [Trifolium pratense]	2E-45
	Contig405	gb|AAK68074.1|AF384970_1	somatic embryogenesis receptor-like kinase 3 [Arabidopsis thaliana]	4E-45
	CaF1_JIE_38_B_08	dbj|BAD95892.1|	Ser/Thr protein kinase [Lotus japonicus]	5E-45
	CaF1_WIE_49_B_05	gb|ABG73621.1|	leucine-rich repeat receptor-like kinase [Populus tomentosa]	5E-45
	CaF1_JIE_19_F_10	gb|ABO81298.1|	Protein kinase; TonB box, N-terminal [Medicago truncatula]	3E-44
	Contig372	sp|P04353|CALM_SPIOL	Calmodulin (CaM)	2E-43
	Contig280	gb|ABO78021.1|	Curculin-like (mannose-binding) lectin [Medicago truncatula]	1E-41
	CaF1_WIE_41_H_01	emb|Y17329.1|PSA17329	Pisum sativum mRNA for calnexin	3E-39
	CaF1_WIE_53_A_02	gb|AAG29593.1|AF196286_1	Ser/Thr specific protein phosphatase 2A A regulatory subunit alpha isoform [Medicago sativa subsp. x varia]	3E-39
	Contig270	gb|ABE85490.2|	Response regulator receiver; CCT [Medicago truncatula]	4E-36
	CaF1_JIE_29_G_07	gb|ABN09164.1|	Protein phosphatase 2C-like [Medicago truncatula]	5E-36
	Contig191	gb|AAT08753.1|	LRR [Hyacinthus orientalis]	3E-35
	CaF1_JIE_12_E_05	dbj|BAB09853.1|	ER66 protein-like [Arabidopsis thaliana]	9E-35
	CaF1_JIE_33_D_01	gb|ABE93018.1|	cAMP response element binding (CREB) protein; Prefoldin [Medicago truncatula]	2E-34
	Contig396	sp|P15001|PHYA_PEA	Phytochrome A gb|AAA33682.1| phytochrome [Pisum sativum] emb|CAA32242.1| phytochrome apoprotein [Pisum sativum] gb|AAT97643.1| phytochrome A apoprotein [Pisum sativum] prf||1604466A phytochrome	4E-32
	Contig896	gb|AAT37529.1|	purple acid phosphatase 1 [Solanum tuberosum]	9E-32
	CaF1_JIE_35_E_02	gb|ABN08649.1|	C2 [Medicago truncatula]	9E-32
	Contig353	emb|CAB43932.1|	putative serine/threonine-specific receptor protein kinase [Arabidopsis thaliana] emb|CAB79676.1| putative serine/threonine-specific receptor protein kinase [Arabidopsis thaliana]	2E-31
	Contig302	ref|NP_201198.1|	leucine-rich repeat transmembrane protein kinase, putative [Arabidopsis thaliana] dbj|BAA96896.1| receptor-like protein kinase [Arabidopsis	6E-30
	CaF1_WIE_54_C_09	ref|NP_568217.1|	BolA-like family protein [Arabidopsis thaliana] dbj|BAB09404.1| unnamed protein product [Arabidopsis thaliana] gb|AAM65194.1| unknown [Arabidopsis thaliana] gb|AAO24583.1| At5g09830 [Arabidopsis thaliana] dbj|BAF00125.1| hypothetical protein [Arabidopsis thaliana]	6E-30
	Contig112	gb|ABD32712.1|	Response regulator receiver; CCT [Medicago truncatula]	7E-29
	CaF1_WIE_01_C_02	emb|CAA19877.1|	protein kinase-like protein [Arabidopsis thaliana] emb|CAB80112.1| protein kinase-like protein [Arabidopsis thaliana]	1E-28
	CaF1_WIE_25_B_07	gb|AAM21172.1|AF305635_1	serine/threonine protein phosphatase 2A [Pisum sativum]	1E-28
	Contig645	gb|ABE81462.1|	Protein kinase [Medicago truncatula]	1E-27
	CaF1_WIE_24_F_03	gb|ABO84518.1|	WD40-like [Medicago truncatula]	3E-27
	CaF1_WIE_33_G_10	dbj|BAA92699.1|	type 2A protein phosphatase-3 [Vicia faba]	1E-25
	CaF1_JIE_33_A_07	gb|AAF78397.1|AC009273_3	Contains similarity to a putative protein T2J13.100 gi|6522560 from Arabidopsis thaliana BAC T2J13 gb|AL132967	3E-24

	Contig367	gb|ABO80441.1|	EPS15 homology (EH) [Medicago truncatula]	7E-24
	CaF1_WIE_02_D_09	sp|P48488|PP1_MEDVA	Serine/threonine-protein phosphatase PP1 emb|CAA56766.1| potentially catalitic subunit of the ser /thr protein phosphatase 1 [Medicago sativa subsp. x varia]	2E-23
	Contig521	gb|ABO84376.1|	cAMP response element binding (CREB) protein [Medicago truncatula]	2E-22
	CaF1_JIE_34_H_11	gb|ABE77486.1|	Protein kinase [Medicago truncatula]	2E-22
	Contig703	dbj|BAD95044.1|	serine/threonine kinase [Arabidopsis thaliana]	4E-22
	CaF1_JIE_07_H_04	gb|ABN09189.1|	cAMP response element binding (CREB) protein [Medicago truncatula]	4E-22
	Contig114	gb|ABE94388.1|	WD-40 repeat [Medicago truncatula] gb|ABO84189.1| WD40-like [Medicago truncatula]	4E-18
	Contig461	gb|ABK06441.1|	flag-tagged protein kinase domain of putative mitogen-activated protein kinase kinase kinase [synthetic construct]	4E-14
	CaF1_JIE_14_A_04	gb|AAT68475.1|	calcium/calmodulin-regulated receptor-like kinase [Medicago sativa]	3E-12
	CaF1_WIE_50_B_03	gb|AAO49473.1|	putative serine/threonine kinase [Vitis vinifera]	5E-11
Stress	CaF1_WIE_37_G_08	gb|ABE85053.1|	Universal stress protein (Usp) [Medicago truncatula]	1E-19
	Contig945	emb|AJ006770.1|CAR6770	Cicer arietinum mRNA for extensin, partial	1E-136
	Contig640	emb|CAB71135.1|	putative imbibition protein [Cicer arietinum]	1E-100
	CaF1_WIE_21_C_04	emb|X95708.1|CANMT1MET	C.arietinum mRNA for metallothionein (clone: CanMT-1)	1E-95
	Contig350	sp|Q39458|MT1_CICAR	Metallothionein-like protein 1 (MT-1) emb|CAA65008.1| metallothionein [Cicer arietinum]	2E-23
	Contig745	gb|AAK66766.1|AF386739_1	aquaporin protein PIP1;1 [Medicago truncatula]	3E-94
	Contig649	emb|AJ299396.1|CAR299396	Cicer arietinum partial mRNA for putative extensin (ORF), clone CanEXT-1	1E-81
	Contig720	gb|ABE84254.1|	Universal stress protein (Usp) [Medicago truncatula] gb|ABE88281.1| Universal stress protein (Usp) [Medicago truncatula]	2E-72
	Contig530	emb|CAB71135.1|	putative imbibition protein [Cicer arietinum]	4E-69
	Contig436	gb|AAB86380.1|	aquaporin-like transmembrane channel protein [Medicago sativa]	2E-67
	Contig431	sp|Q9FY14|TIP1_MEDTR	Probable aquaporin TIP-type (MtAQP1) emb|CAC01618.1| aquaporin [Medicago truncatula]	3E-64
	Contig811	gb|AAQ74889.1|	Al-induced protein [Gossypium hirsutum]	1E-60
	CaF1_WIE_28_E_01	emb|CAA72183.1|	annexin-like protein [Medicago sativa]	1E-58
	CaF1_WIE_48_F_09	gb|ABE90758.1|	Annexin [Medicago truncatula]	3E-56
	CaF1_WIE_47_D_03	gb|AAY22204.1|	putative aquaporin [Phaseolus vulgaris]	5E-54
	CaF1_WIE_33_B_05	ref|NP_567575.1|	ERD3 (EARLY-RESPONSIVE TO DEHYDRATION 3) [Arabidopsis thaliana] ref|NP_849408.1| ERD3 (EARLY-RESPONSIVE TO DEHYDRATION 3) [Arabidopsis thaliana] dbj|BAB63914.1| ERD3 protein [Arabidopsis thaliana]	3E-51
	CaF1_JIE_36_F_10	ref|NP_181934.1|	late embryogenesis abundant family protein / LEA family protein [Arabidopsis thaliana] ref|NP_850408.1| late embryogenesis abundant family protein / LEA family protein [Arabidopsis thaliana] gb|AAC23428.1| similar to late embryogenesis abundant proteins [Arabidopsis thaliana] gb|ABD59061.1| At2g44060 [Arabidopsis thaliana]	6E-50
	CaF1_WIE_04_E_08	gb|AAW31666.1|	putative late-embryogenesis protein-like protein [Ammopiptanthus mongolicus]	4E-29
	Contig908	gb|ABE85050.1|	Universal stress protein (Usp) [Medicago truncatula]	3E-49
	Contig345	ref|NP_566991.2|	universal stress protein (USP) family protein [Arabidopsis thaliana] gb|AAG40033.1|AF324682_1 AT3g53990 [Arabidopsis thaliana] gb|AAG40390.1|AF325038_1 AT3g53990 [Arabidopsis thaliana] gb|AAG41484.1|AF326902_1 unknown protein [Arabidopsis thaliana] gb|AAK00403.1|AF339721_1 unknown protein [Arabidopsis thaliana] gb|AAK32867.1|AF361855_1 AT3g53990/F5K20_290 [Arabidopsis thaliana] emb|CAB88361.1| hypothetical protein [Arabidopsis thaliana] gb|AAK96518.1| AT3g53990/F5K20_290 [Arabidopsis thaliana] gb|AAL31227.1| AT3g53990/F5K20_290 [Arabidopsis thaliana] gb|AAL49942.1| AT3g53990/F5K20_290 [Arabidopsis thaliana] dbj|BAD94963.1| hypothetical protein [Arabidopsis thaliana]	4E-49
	Contig707	gb|AF155232.1|AF155232	Pisum sativum extensin (Ext) mRNA, complete cds	3E-46
	CaF1_WIE_01_E_03	gb|AAY43802.1|	Fb2 [Gossypium hirsutum]	5E-39
	CaF1_WIE_42_G_01	emb|AJ299396.1|CAR299396	Cicer arietinum partial mRNA for putative extensin (ORF), clone CanEXT-1	1E-38
	CaF1_WIE_31_G_06	emb|CAC08564.1|	wound-induced GSK-3-like protein [Medicago sativa subsp. x varia]	3E-38
	Contig759	gb|AAQ21120.1|	early light inducible protein [Trifolium pratense]	6E-37
	CaF1_JIE_18_D_08	gb|AAV66464.1|	drought responsive element binding protein [Glycine soja]	6E-36
	CaF1_WIE_06_A_08	emb|AJ299396.1|CAR299396	Cicer arietinum partial mRNA for putative extensin (ORF), clone CanEXT-1	7E-34
	CaF1_WIE_02_H_03	emb|X95708.1|CANMT1MET	C.arietinum mRNA for metallothionein (clone: CanMT-1)	2E-33
	Contig675	emb|CAA07232.1|	putative Pi starvation-induced protein [Cicer arietinum]	1E-30
	Contig27	emb|CAB61749.1|	putative water channel protein [Cicer arietinum]	1E-29

	CaF1_WIE_51_D_06	gb|AAB71830.1|	annexin [Lavatera thuringiaca]	6E-26
	CaF1_WIE_52_B_07	gb|AF397032.2|	Pisum sativum clone PsEXT3.28 root nodule extensin mRNA, partial cds	1E-25
	Contig959	emb|CAC08564.1|	wound-induced GSK-3-like protein [Medicago sativa subsp. x varia]	5E-25
	Contig319	emb|CAG14983.1|	putative universal stress protein [Cicer arietinum]	3E-23
	CaF1_WIE_40_A_06	gb|ABE79560.1|	Chaperone DnaK [Medicago truncatula]	3E-13
	CaF1_WIE_05_D_08	gb|AAF33784.1|AF220456_1	cold acclimation responsive protein BudCAR4 [Medicago sativa] gb|AAF33786.1|AF220458_1 cold acclimation responsive protein BudCAR6 [Medicago sativa] gb|AAA16926.1| CAS15	2E-18
	Contig958	gb|AAX18706.1|	cold-related protein Cor413 [Gossypium barbadense] gb|ABI97481.1| COR413-like protein [Gossypium barbadense]	7E-13
Transcription	CaF1_WIE_30_H_09	sp|Q42877|RPB2_SOLLC	DNA-directed RNA polymerase II subunit RPB2 (RNA polymerase II subunit B2) (RNA polymerase II subunit 2) (DNA-directed RNA polymerase II 135 kDa polypeptide) gb|AAC49273.1| RNA polymerase II subunit 2	4E-83
	Contig458	gb|ABP03503.1|	Pathogenesis-related transcriptional factor and ERF [Medicago truncatula]	8E-70
	Contig534	dbj|BAE71244.1|	putative DNA binding protein [Trifolium pratense]	9E-75
	Contig4	gb|ABP03302.1|	Zinc finger, C2H2-type [Medicago truncatula]	2E-73
	Contig147	gb|ABE83604.1|	PUG; Zinc finger, C2H2-type; UBA-like [Medicago truncatula] gb|ABE91517.1| PUG; Zinc finger, C2H2-type; UBA-like [Medicago truncatula]	2E-68
	Contig929	gb|ABD33016.1|	Transcription Factor IIF, Rap30/Rap74, interaction [Medicago truncatula]	3E-68
	CaF1_WIE_08_A_11	gb|AAP69821.1|	ARF [Oryza sativa (japonica cultivar-group)]	2E-34
	Contig360	gb|ABE84268.2|	Zinc finger, CCHC-type; Putative 5-3 exonuclease [Medicago truncatula]	8E-68
	Contig518	gb|AAC16330.1|	SAR DNA-binding protein-1 [Pisum sativum]	3E-66
	Contig726	gb|ABE87796.1|	Zinc finger, RING-type; RINGv [Medicago truncatula]	8E-66
	Contig160	dbj|BAE71188.1|	BEL1-like homeodomain transcription factor [Trifolium pratense]	1E-64
	Contig293	gb|AAK84887.1|AF402606_1	homeodomain leucine zipper protein HDZ3 [Phaseolus vulgaris]	2E-56
	Contig223	gb|ABE93791.1|	NOT2/NOT3/NOT5 [Medicago truncatula]	3E-55
	CaF1_WIE_31_F_11	gb|ABN07918.1|	Zinc finger, ZZ-type; Zinc finger, C2H2-type [Medicago truncatula]	1E-54
	Contig502	gb|AAX47170.1|	SHORT VEGETATIVE PHASE [Pisum sativum]	8E-52
	CaF1_WIE_53_F_03	gb|ABG90380.1|	bZIP transcription factor [Caragana korshinskii]	4E-46
	Contig434	gb|AAZ38969.1|	GAMYB-binding protein [Glycine max]	9E-45
	Contig111	gb|ABE84970.1|	Pathogenesis-related transcriptional factor and ERF [Medicago truncatula]	6E-26
	Contig378	emb|CAA66478.1|	transcription factor [Vicia faba var. minor]	2E-44
	CaF1_WIE_04_B_03	ref|NP_179333.2|	ARID/BRIGHT DNA-binding domain-containing protein [Arabidopsis thaliana] gb|AAK96550.1| At2g17400 [Arabidopsis thaliana] gb|AAO11549.1| At2g17400/At2g17400 [Arabidopsis thaliana] dbj|BAF02083.1| hypothetical protein [Arabidopsis thaliana]	2E-44
	Contig395	gb|ABE85988.1|	Zinc finger, RING-type; Zinc finger, RanBP2-type; Zinc finger, C6HC-type [Medicago truncatula]	2E-43
	Contig23	gb|AAC28907.1|	phaseolin G-box binding protein PG2 [Phaseolus vulgaris]	4E-37
	Contig62	ref|NP_564424.1|	PHD finger family protein [Arabidopsis thaliana] sp|Q9C810|Y1342_ARATH PHD finger protein At1g33420 gb|AAG51204.1|AC051630_1 hypothetical protein; 47104-44821 [Arabidopsis thaliana] gb|AAK59559.1| unknown protein [Arabidopsis thaliana] gb|AAK93738.1| unknown protein [Arabidopsis thaliana]	4E-37
	CaF1_WIE_03_A_06	gb|AAV85853.1|	AT-rich element binding factor 3 [Pisum sativum]	2E-34
	CaF1_WIE_39_H_04	gb|ABB47998.1|	Zinc finger, C3HC4 type family protein, expressed [Oryza sativa (japonica cultivar-group)]	1E-33
	Contig586	gb|ABH02865.1|	MYB transcription factor MYB176 [Glycine max]	5E-33
	Contig22	gb|ABE83191.2|	Zinc finger, CCHC-type [Medicago truncatula]	7E-32
	Contig891	gb|ABE86663.2|	Zinc finger, RING-type [Medicago truncatula]	7E-32
	CaF1_WIE_49_C_09	gb|ABE91004.1|	Zinc finger, CCCH-type; Zinc finger, RING-type [Medicago truncatula]	2E-31
	Contig217	ref|NP_974856.1|	zinc finger (ZPR1-type) family protein [Arabidopsis thaliana]	3E-31
	CaF1_WIE_37_A_10	gb|AAK84885.1|AF402604_1	homeodomain leucine zipper protein HDZ1 [Phaseolus vulgaris]	3E-31
	CaF1_WIE_25_C_10	gb|ABP03222.1|	Zinc finger, FYVE/PHD-type [Medicago truncatula]	4E-30
	Contig316	ref|NP_196487.1|	KIWI; DNA binding / transcription coactivator [Arabidopsis thaliana] sp|O65154|KIWI_ARATH RNA polymerase II transcriptional coactivator KIWI gb|AAC08574.1| putative transcriptional co-activator [Arabidopsis thaliana] emb|CAC05451.1| putative transcriptional co-activator (KIWI) [Arabidopsis thaliana] gb|AAO44011.1| At5g09250 [Arabidopsis thaliana] dbj|BAF00036.1| putative transcriptional co-activator [Arabidopsis thaliana]	4E-28
	Contig452	gb|AAK84885.1|AF402604_1	homeodomain leucine zipper protein HDZ1 [Phaseolus vulgaris]	1E-27
	Contig650	gb|ABO81948.2|	Zinc finger, GATA-type [Medicago truncatula]	6E-26

	CaF1_WIE_45_E_07	gb|ABH02845.1|	MYB transcription factor MYB93 [Glycine max]	5E-25
	Contig970	gb|ABD32383.1|	Zinc finger, RING-type [Medicago truncatula]	6E-25
	Contig934	ref|NP_181843.2|	DNA binding / transcription factor [Arabidopsis thaliana]	5E-24
	CaF1_JIE_41_D_04	emb|CAA87075.1|	heat shock transcription factor 29 [Glycine max]	7E-24
	CaF1_WIE_52_E_05	ref|NP_181843.2|	DNA binding / transcription factor [Arabidopsis thaliana]	4E-23
	Contig83	gb|AAZ14831.1|	putative AP2-binding protein [Jatropha curcas]	5E-23
	Contig611	gb|ABE80756.1|	DNA-binding WRKY [Medicago truncatula] gb|ABO80762.1| DNA-binding WRKY [Medicago truncatula]	7E-21
	Contig236	gb|ABD32320.1|	DNA-directed RNA polymerase, subunit C11/M/9 [Medicago truncatula]	1E-20
	Contig241	emb|CAA54168.1|	HMG 1 protein [Pisum sativum]	3E-16
	CaF1_WIE_30_E_09	gb|AAM63313.1|	Contains similarity to bHLH transcription factor GBOF-1 from Tulipa gesneriana gb|AF185269 [Arabidopsis thaliana]	6E-13
	Contig875	gb|ABH02852.1|	MYB transcription factor MYB112 [Glycine max]	7E-13
	Contig362	ref|NP_198099.1|	seryl-tRNA synthetase / serine--tRNA ligase [Arabidopsis thaliana] sp|Q39230|SYS_ARATH Seryl-tRNA synthetase (Seryl-tRNA(Ser/Sec) synthetase) (Serine--tRNA ligase) (SerRS) gb|AAK28648.1|AF360352_1 putative seryl-tRNA synthetase [Arabidopsis thaliana] emb|CAA94388.1| seryl-tRNA Synthetase [Arabidopsis thaliana] gb|AAK93731.1| putative seryl-tRNA synthetase [Arabidopsis thaliana]	2E-15
	CaF1_JIE_22_G_08	gb|ABE86660.1|	Zinc finger, C2H2-type [Medicago truncatula]	3E-14
	Contig247	gb|ABD32869.1|	Zinc finger, RING-type; RINGv [Medicago truncatula] gb|ABE79868.2| Zinc finger, RING-type [Medicago truncatula]	4E-11
	CaF1_WIE_33_A_03	ref|NP_182310.1|	zinc finger (B-box type) family protein [Arabidopsis thaliana] sp|O82256|COL13_ARATH Zinc finger protein CONSTANS-LIKE 13 gb|AAC63643.1| putative zinc-finger protein (B-box zinc finger domain) [Arabidopsis thaliana] gb|AAM15120.1| putative zinc-finger protein (B-box zinc finger domain) [Arabidopsis thaliana] gb|AAY56404.1| At2g47890 [Arabidopsis thaliana]	7E-11
	Contig691	gb|ABE84071.1|	Nucleic acid-binding, OB-fold, subgroup [Medicago truncatula]	1E-77
	Contig833	dbj|BAB32793.1|	110 kDa 4SNc-Tudor domain protein [Pisum sativum]	8E-68
	CaF1_WIE_05_F_04	gb|ABE77517.1|	HSF/ETS, DNA-binding [Medicago truncatula]	7E-59
	CaF1_WIE_54_A_01	dbj|BAB32793.1|	110 kDa 4SNc-Tudor domain protein [Pisum sativum]	6E-44
	Contig31	ref|NP_565451.1|	nucleic acid binding / zinc ion binding [Arabidopsis thaliana] gb|AAM14872.1| Expressed protein [Arabidopsis thaliana] gb|ABD19677.1| At2g19385 [Arabidopsis thaliana]	1E-43
	Contig836	gb|AAD39439.1|AF132001_1	PHAP2A protein [Petunia x hybrida]	9E-33
	CaF1_WIE_27_A_03	gb|ABN09177.1|	Single-stranded nucleic acid binding R3H [Medicago truncatula]	1E-20
	CaF1_WIE_34_F_11	gb|ABE93168.1|	Prefoldin; Helix-loop-helix DNA-binding [Medicago truncatula]	8E-20
	CaF1_WIE_12_H_05	gb|ABE80160.2|	Prefoldin [Medicago truncatula]	6E-45
	Contig756	emb|AJ006767.1|CAR6767	Cicer arietinum mRNA for histone H1	1E-137
	Contig196	ref|NP_568725.1|	ELO3 (ELONGATA 3); N-acetyltransferase/ catalytic/ hydrogen ion transporting ATP synthase, rotational mechanism / hydrogen ion transporting ATPase, rotational mechanism / iron ion binding [Arabidopsis thaliana] sp|Q93ZR1|ELP3_ARATH Elongator complex protein 3 (Protein ELONGATA 3) (Elongator component 3) gb|AAL07172.1| putative histone acetyltransferase [Arabidopsis thaliana] emb|CAI79647.1| elongator component [Arabidopsis thaliana]	2E-67
	CaF1_WIE_13_C_11	gb|AAM34784.1|AF510671_1	HDA2 [Arabidopsis thaliana]	3E-65
	Contig966	ref|NP_001078516.1|	histone H3.2 [Arabidopsis thaliana]	9E-53
	Contig587	dbj|BAA19156.1|	HMG-1 [Canavalia gladiata]	1E-45
	CaF1_WIE_09_C_11	gb|ABO81477.1|	GB|AAB61107.1|2194132|F20P5 , putative [Medicago truncatula]	6E-44
	CaF1_WIE_13_D_02	gb|ABE79269.1|	Regulator of chromosome condensation/beta-lactamase-inhibitor protein II [Medicago truncatula]	4E-40
	Contig387	gb|ABO84100.1|	Something about silencing protein 10 , related [Medicago truncatula]	9E-38
	Contig571	ref|XP_391802.1|	H2B_NEUCR Histone H2B [Gibberella zeae PH-1] sp|Q4HTT2|H2B_GIBZE Histone H2B	6E-33
	Contig212	sp|Q8LJS2|HDT1_SOYBN	Histone deacetylase HDT1 (Histone deacetylase 2a) (HD2a) (Nucleolar histone deacetylase HD2-p39) gb|AAN03465.1| nucleolar histone deacetylase HD2-P39 [Glycine max]	3E-31
	CaF1_JIE_05_E_06	dbj|BAD90801.1|	histone 3 [Conocephalum conicum]	5E-27

	Contig374	ref|NP_200914.2|	HDA05 (HISTONE DEACETYLASE5); histone deacetylase [Arabidopsis thaliana] sp|Q8RX28|HDA5_ARATH Histone deacetylase 5 gb|AAM13986.1| putative histone deacetylase [Arabidopsis thaliana] gb|AAM53342.1| histone deacetylase-like protein [Arabidopsis thaliana] gb|AAN15392.1| histone deacetylase-like protein [Arabidopsis thaliana]	2E-24
	CaF1_WIE_18_H_03	emb|CAJ38371.1|	HMG-protein [Plantago major]	7E-21
	CaF1_WIE_39_H_07	gb|ABE81390.1|	N-6 Adenine-specific DNA methylase [Medicago truncatula]	9E-19
	Contig713	sp|Q8LJS2|HDT1_SOYBN	Histone deacetylase HDT1 (Histone deacetylase 2a) (HD2a) (Nucleolar histone deacetylase HD2-p39) gb|AAN03465.1| nucleolar histone deacetylase HD2-P39 [Glycine max]	1E-16
Translation, ribosomal structure and biogenesis	CaF1_JIE_07_H_03	gb|ABE82281.1|	Ribosomal protein S9 [Medicago truncatula] gb|ABO82751.1| Ribosomal protein S9 [Medicago truncatula]	3E-14
	Contig540	gb|ABE77438.1|	Ribosomal protein L24/L26 [Medicago truncatula]	7E-14
	Contig747	emb|CAB52812.1|	Ribosomal protein L7Ae-like (fragment) [Arabidopsis thaliana] emb|CAB79193.1| Ribosomal protein L7Ae-like (fragment) [Arabidopsis thaliana]	4E-14
	CaF1_JIE_03_C_04	gb|EDN17888.1|	eukaryotic initiation factor 4A [Botryotinia fuckeliana B05.10]	1E-11
	CaF1_JIE_09_D_03	gb|ABE87516.2|	Ribosomal protein L7Ae/L30e/S12e/Gadd45 [Medicago truncatula]	2E-11
	CaF1_WIE_07_G_02	gb|ABM53472.1|	eIF5A [Rosa chinensis]	5E-43
	Contig572	sp|Q9SQF4|SUI1_BRAOL	Protein translation factor SUI1 homolog (Translation initiation factor nps45) gb|AAF04624.1|AF098672_1 translation initiation factor nps45 [Brassica oleracea]	2E-32
	CaF1_JIE_21_B_05	gb|AAK92832.1|	putative glycyl tRNA synthetase [Arabidopsis thaliana]	2E-29
	CaF1_WIE_40_H_09	gb|ABE89800.2|	eIF4-gamma/eIF5/eIF2-epsilon [Medicago truncatula]	2E-29
	Contig412	emb|AJ577394.1|CAR577394	Cicer arietinum 18S rRNA gene, 5.8S rRNA gene, IGS, ITS1 and ITS2, clone CanrDNA	0
	Contig496	gb|ABE82951.1|	Translation protein SH3-like [Medicago truncatula]	1E-127
	Contig260	emb|CAA06245.1|	elongation factor 1-alpha (EF1-a) [Cicer arietinum]	1E-125
	Contig609	emb|CAD56219.1|	ribosomal protein S3a [Cicer arietinum]	1E-102
	Contig840	gb|ABE88774.1|	Translation factor; Elongation factor G, III and V [Medicago truncatula]	6E-97
	Contig715	gb|AAS47511.1|	ribosomal protein S6 [Glycine max]	8E-92
	Contig218	gb|ABO78621.1|	Translation factor [Medicago truncatula]	3E-91
	Contig66	sp|O81361|RS8_PRUAR	40S ribosomal protein S8 gb|AAC24583.1| 40S ribosomal protein S8 [Prunus armeniaca]	8E-91
	Contig748	ref|NP_191771.1|	tRNA synthetase class II (G, H, P and S) family protein [Arabidopsis thaliana] ref|NP_850736.1| tRNA synthetase class II (G, H, P and S) family protein [Arabidopsis thaliana] emb|CAB71872.1| multifunctional aminoacyl tRNA ligase-like protein [Arabidopsis thaliana] gb|AAL24294.1| multifunctional aminoacyl-tRNA ligase-like protein [Arabidopsis thaliana] gb|AAM91120.1| multifunctional aminoacyl-tRNA ligase-like protein [Arabidopsis thaliana]	2E-82
	Contig566	dbj|BAB86847.1|	elongation factor EF-2 [Pisum sativum]	2E-81
	Contig400	gb|EF672342.1|	Hypocrea sp. Z28 18S ribosomal RNA gene, partial sequence	8E-81
	Contig6	sp|P62302|RS13_SOYBN	40S ribosomal protein S13 gb|AAS47510.1| ribosomal protein S13 [Glycine max]	7E-79
	CaF1_WIE_34_A_04	gb|ABE81765.1|	Translation initiation factor eIF-3b [Medicago truncatula]	1E-78
	Contig631	gb|ABA12218.1|	translation elongation factor 1A-2 [Gossypium hirsutum]	7E-77
	Contig97	emb|CAB76914.1|	60S ribosomal protein L6 [Cicer arietinum]	4E-75
	CaF1_WIE_26_C_01	gb|AAO46881.1|	60S ribosomal protein [Medicago sativa]	9E-73
	Contig589	gb|ABE82951.1|	Translation protein SH3-like [Medicago truncatula]	3E-66
	Contig849	gb|ABE82912.1|	Ribosomal protein S4, bacterial and organelle form [Medicago truncatula]	1E-64
	CaF1_WIE_54_D_03	dbj|BAB86847.1|	elongation factor EF-2 [Pisum sativum]	2E-62
	CaF1_JIE_02_G_01	gb|ABE91098.1|	Ribosomal protein S4E; RNA-binding S4; KOW [Medicago truncatula]	1E-61
	Contig641	ref|NP_191771.1|	tRNA synthetase class II (G, H, P and S) family protein [Arabidopsis thaliana] ref|NP_850736.1| tRNA synthetase class II (G, H, P and S) family protein [Arabidopsis thaliana] emb|CAB71872.1| multifunctional aminoacyl tRNA ligase-like protein [Arabidopsis thaliana] gb|AAL24294.1| multifunctional aminoacyl-tRNA ligase-like protein [Arabidopsis thaliana] gb|AAM91120.1| multifunctional aminoacyl-tRNA ligase-like protein [Arabidopsis thaliana]	2E-59
	Contig14	gb|AAT01416.1|	translation initiation factor 5A [Tamarix androssowii]	1E-58
	Contig285	gb|ABE81204.1|	Ribosomal protein S13 [Medicago truncatula] gb|ABE81211.1| Ribosomal protein S13 [Medicago truncatula]	2E-58
	CaF1_JIE_36_H_11	gb|ABE83633.1|	Ribosomal protein L10; Ribosomal protein 60S [Medicago truncatula]	2E-58

	Contig517	sp|O81361|RS8_PRUAR	40S ribosomal protein S8 gb|AAC24583.1| 40S ribosomal protein S8 [Prunus armeniaca]	3E-58
	CaF1_WIE_41_H_09	gb|ABP02866.1|	Ribosomal protein L30e [Medicago truncatula]	3E-58
	Contig906	gb|ABE87516.2|	Ribosomal protein L7Ae/L30e/S12e/Gadd45 [Medicago truncatula]	6E-58
	CaF1_JIE_11_E_07	gb|ABE88774.1|	Translation factor; Elongation factor G, III and V [Medicago truncatula]	2E-57
	CaF1_WIE_53_G_02	sp|P55844|RL14_PEA	Probable 60 ribosomal protein L14 (Hydroxyproline-rich glycoprotein HRGP1)	9E-56
	CaF1_WIE_28_A_05	gb|AAM61490.1|	60S ribosomal protein L13, BBC1 protein [Arabidopsis thaliana]	3E-54
	Contig230	gb|ABO78621.1|	Translation factor [Medicago truncatula]	3E-52
	Contig50	sp|Q9M5L0|RL35_EUPES	60S ribosomal protein L35 gb|AAF34800.1|AF227980_1 60S ribosomal protein L35 [Euphorbia esula]	5E-52
	Contig696	sp|O65751|RSSA_CICAR	40S ribosomal protein SA (p40) emb|CAA07226.1| ribosome-associated protein p40 [Cicer arietinum]	3E-51
	Contig556	gb|ABA40437.1|	40S ribosomal protein S7-like protein [Solanum tuberosum] gb|ABA46775.1| unknown [Solanum tuberosum] gb|ABB17004.1| ribosomal protein S7-like protein [Solanum tuberosum] gb|ABB87101.1| 40S ribosomal protein S7-like protein-like [Solanum tuberosum]	1E-49
	CaF1_WIE_52_A_07	gb|AAP80667.1|AF479048_1	ribosomal Pr 117 [Triticum aestivum]	5E-49
	CaF1_WIE_40_F_08	gb|ABB55398.1|	40S ribosomal protein S10-like [Solanum tuberosum]	1E-46
	Contig40	emb|CAI48073.1|	60S ribosomal protein L37a [Capsicum chinense]	3E-46
	Contig127	gb|AAA34366.1|	ribosomal protein L41	8E-46
	Contig383	emb|CAA71881.1|	Tyrosyl-tRNA synthetase [Nicotiana tabacum]	2E-45
	CaF1_WIE_16_C_10	emb|CAA10125.1|	40S ribosomal protein S19 [Cicer arietinum]	5E-44
	Contig90	gb|ABE79479.1|	Ribosomal protein L10E [Medicago truncatula]	8E-44
	Contig533	gb|ABO80199.1|	Ribosomal L23 protein; Ribosomal protein L23, N-terminal [Medicago truncatula]	4E-43
	Contig810	gb|ABO81713.1|	Ribosomal protein L36E [Medicago truncatula]	6E-42
	CaF1_JIE_16_G_09	ref|XP_380571.1|	RS15_PODAN 40S RIBOSOMAL PROTEIN S15 (S12) [Gibberella zeae PH-1]	8E-41
	Contig588	gb|AAG17879.1|AF293406_1	60S ribosomal protein L10A [Phaseolus coccineus]	1E-40
	CaF1_WIE_11_H_10	sp|Q9XF97|RL4_PRUAR	60S ribosomal protein L4 (L1) gb|AAD32206.1|AF134732_1 60S ribosomal protein L1 [Prunus armeniaca]	7E-40
	Contig497	gb|AAO46881.1|	60S ribosomal protein [Medicago sativa]	2E-39
	Contig796	gb|ABE84969.1|	Ribosomal protein L19e [Medicago truncatula]	8E-39
	Contig921	gb|ABE88774.1|	Translation factor; Elongation factor G, III and V [Medicago truncatula]	4E-38
	CaF1_JIE_25_C_01	gb|ABE94142.2|	Translation initiation factor IF5 [Medicago truncatula]	4E-38
	Contig946	gb|ABE92854.1|	Ribosomal L22e protein [Medicago truncatula]	3E-37
	CaF1_WIE_14_G_01	emb|AJ404848.1|GMA404848	Glycine max mRNA for ribosomal protein L2 (rpL2 gene)	4E-37
	Contig889	gb|ABE90867.1|	Ribosomal L18ae protein [Medicago truncatula]	2E-36
	Contig537	gb|ABN08656.1|	Ribosomal protein S10, eukaryotic and archaeal form [Medicago truncatula]	4E-35
	Contig99	gb|AAK95391.1|	ribosomal protein L2 [Gossypium arboreum]	4E-31
	CaF1_JIE_10_B_07	gb|ABE85391.1|	S25 ribosomal protein [Medicago truncatula] gb|ABE85967.1| S25 ribosomal protein [Medicago truncatula]	8E-31
	CaF1_WIE_50_E_03	gb|AAS47511.1|	ribosomal protein S6 [Glycine max]	1E-30
	CaF1_WIE_51_C_09	sp|Q945F4|IF5A2_MEDSA	Eukaryotic translation initiation factor 5A-2 (eIF-5A-2) gb|AAL10404.1|AF416338_1 eukaryotic translation initiation factor 5A-2 [Medicago sativa] gb|ABE85424.1| NusG [Medicago truncatula] gb|ABE88714.1| NusG [Medicago truncatula]	5E-30
	CaF1_WIE_09_C_10	gb|L47967.1|PEARPL41A	Pisum sativum (clone PsRCI35-2) ribosomal protein L41 mRNA, complete cds	7E-30
	Contig847	gb|ABE85391.1|	S25 ribosomal protein [Medicago truncatula] gb|ABE85967.1| S25 ribosomal protein [Medicago truncatula]	1E-29
	CaF1_WIE_51_H_05	gb|AF071889.1|AF071889	Prunus armeniaca 40S ribosomal protein S8 (RPS8) mRNA, complete cds	2E-29
	Contig500	dbj|AK226272.1|	Arabidopsis thaliana mRNA for ribosomal protein S30 homolog, complete cds, clone: RAFL05-08-E11	4E-29
	CaF1_JIE_01_C_01	gb|ABD32214.1|	Ribosomal protein L34e [Medicago truncatula] gb|ABN08919.1| Ribosomal protein L34e [Medicago truncatula]	6E-29
	CaF1_WIE_38_A_08	dbj|AB262513.1|	Pseudomonas aeruginosa gene for 16S rRNA, partial sequence, strain: Hg2	8E-29
	Contig826	dbj|AB262513.1|	Pseudomonas aeruginosa gene for 16S rRNA, partial sequence, strain: Hg2	9E-29
	CaF1_WIE_38_G_10	dbj|AB262513.1|	Pseudomonas aeruginosa gene for 16S rRNA, partial sequence, strain: Hg2	9E-29
	CaF1_WIE_38_D_08	dbj|AB262513.1|	Pseudomonas aeruginosa gene for 16S rRNA, partial sequence, strain: Hg2	1E-28
	CaF1_WIE_38_F_09	dbj|AB262513.1|	Pseudomonas aeruginosa gene for 16S rRNA, partial sequence, strain: Hg2	2E-28
	CaF1_WIE_48_E_04	emb|CAI48073.1|	60S ribosomal protein L37a [Capsicum chinense]	4E-27

	Contig478	gb|AAC78102.1|	60S ribosomal protein L21 [Oryza sativa]	7E-27
	Contig943	sp|P49163|RK22_MEDSA	50S ribosomal protein L22, chloroplast precursor (CL22) gb|AAB46612.1| ribosomal protein CL22 [Medicago sativa]	1E-25
	CaF1_WIE_49_B_04	sp|Q9MAV7|RL31_PANGI	60S ribosomal protein L31 dbj|BAA96368.1| ribosomal protein L31 [Panax ginseng]	4E-24
	CaF1_WIE_56_H_11	gb|AAD47346.1|AF112440_1	ribosomal protein S26 [Pisum sativum]	2E-23
	CaF1_WIE_43_G_02	ref|XP_359800.2|	ribosomal protein L39 [Magnaporthe grisea 70-15] gb|EDJ95441.1| ribosomal protein L39 [Magnaporthe grisea 70-15] gb|EDN06404.1| ribosomal protein L39 [Ajellomyces capsulatus NAm1]	6E-23
	CaF1_WIE_09_G_04	ref|NP_568818.1|	eukaryotic translation initiation factor SUI1, putative [Arabidopsis thaliana] ref|NP_851192.1| eukaryotic translation initiation factor SUI1, putative [Arabidopsis thaliana] gb|AAK60326.1|AF385736_1 AT5g54940/MBG8_21 [Arabidopsis thaliana] dbj|BAB08773.1| translation initiation factor-like protein [Arabidopsis thaliana] gb|AAM64690.1| translation initiation factor-like protein [Arabidopsis thaliana] gb|AAM91507.1| AT5g54940/MBG8_21 [Arabidopsis thaliana]	4E-22
	Contig457	gb|AAP80667.1|AF479048_1	ribosomal Pr 117 [Triticum aestivum]	1E-21
	CaF1_WIE_12_G_04	gb|ABO78621.1|	Translation factor [Medicago truncatula]	6E-21
	CaF1_WIE_53_A_10	sp|O65731|RS5_CICAR	40S ribosomal protein S5 emb|CAA06491.1| 40S ribosomal protein S5 [Cicer arietinum]	5E-19
	Contig802	gb|ABO79321.1|	Ribosomal protein S24e [Medicago truncatula]	3E-18
	Contig80	gb|ABB29934.1|	acidic ribosomal protein P1a-like [Solanum tuberosum]	1E-18
	CaF1_WIE_39_H_06	sp|Q9SC12|IF5A_SENVE	Eukaryotic translation initiation factor 5A (eIF-5A) emb|CAB65463.1| translation initiation factor 5A precursor protein (eIF-5A) [Senecio vernalis]	6E-18
	Contig728	gb|ABE84969.1|	Ribosomal protein L19e [Medicago truncatula]	1E-17
Unknown function	CaF1_WIE_16_B_07	ref|NP_187764.1|	unknown protein [Arabidopsis thaliana] gb|AAG51447.1|AC008153_20 hypothetical protein; 89863-88075 [Arabidopsis thaliana]	1E-11
	Contig373	ref|NP_189052.2|	unknown protein [Arabidopsis thaliana] gb|AAK44146.1|AF370331_1 unknown protein [Arabidopsis thaliana] gb|AAN13152.1| unknown protein [Arabidopsis thaliana]	3E-12
	CaF1_WIE_12_B_08	gb|AAF43953.1|AC012188_30	Strong similarity to an unknown protein from Arabidopsis thaliana gb|AL049171.1	3E-20
	CaF1_WIE_49_C_02	ref|NP_173826.1|	unknown protein [Arabidopsis thaliana] gb|AAC00576.1| Unknown protein [Arabidopsis thaliana]	2E-17
	Contig742	gb|AAF26124.1|AC012328_27	unknown protein [Arabidopsis thaliana]	4E-17
	CaF1_WIE_49_D_07	gb|ABE93358.1|	Protein of unknown function DUF630 [Medicago truncatula]	6E-13
	CaF1_WIE_34_H_11	gb|ABB02640.1|	unknown [Solanum tuberosum]	4E-67
	CaF1_JIE_20_D_03	gb|ABE82132.1|	Protein of unknown function DUF239, plant [Medicago truncatula]	2E-64
	CaF1_JIE_06_D_02	gb|ABO81176.1|	Protein of unknown function DUF26 [Medicago truncatula]	4E-62
	Contig272	gb|ABA46758.1|	unknown [Solanum tuberosum] gb|ABB02647.1| unknown [Solanum tuberosum]	2E-61
	CaF1_WIE_51_A_02	gb|ABE91787.1|	Protein of unknown function DUF707 [Medicago truncatula]	2E-61
	CaF1_JIE_22_B_11	ref|NP_196799.1|	unknown protein [Arabidopsis thaliana] emb|CAB88259.1| putative protein [Arabidopsis thaliana] dbj|BAC42665.1| unknown protein [Arabidopsis thaliana]	8E-60
	Contig672	gb|ABO82672.1|	Protein of unknown function DUF568 [Medicago truncatula]	1E-59
	CaF1_WIE_26_H_04	dbj|BAD94972.1|	putative protein [Arabidopsis thaliana]	1E-52
	Contig590	ref|NP_974254.1|	unknown protein [Arabidopsis thaliana] gb|AAG50831.1|AC074395_5 unknown protein, 5' partial [Arabidopsis thaliana]	2E-52
	CaF1_WIE_04_E_07	ref|NP_199258.3|	unknown protein [Arabidopsis thaliana]	1E-51
	CaF1_JIE_09_E_03	gb|AAD52015.1|AF082862_1	unknown [Pisum sativum]	2E-51
	Contig449	ref|NP_683525.2|	unknown protein [Arabidopsis thaliana] sp|Q6ID70|Y3377_ARATH Protein At3g03773 gb|AAT41786.1| At3g03773 [Arabidopsis thaliana] gb|AAT68743.1| hypothetical protein At3g03773 [Arabidopsis thaliana] gb|AAT70471.1| At3g03773 [Arabidopsis thaliana] gb|AAX55169.1| hypothetical protein At3g03773 [Arabidopsis thaliana]	8E-51
	CaF1_WIE_17_E_08	ref|NP_201196.1|	unknown protein [Arabidopsis thaliana] sp|P57681|PCYOX_ARATH Probable prenylcysteine oxidase precursor dbj|BAB11039.1| unnamed protein product [Arabidopsis thaliana] gb|AAL91144.1| unknown protein [Arabidopsis thaliana] gb|AAM91116.1| unknown protein [Arabidopsis thaliana]	9E-51
	CaF1_WIE_41_G_08	ref|NP_569023.1|	unknown protein [Arabidopsis thaliana] gb|AAL38822.1| unknown protein [Arabidopsis thaliana] gb|AAM51275.1| unknown protein [Arabidopsis thaliana]	3E-50
	CaF1_WIE_54_B_06	emb|CAB36704.1|	putative protein [Arabidopsis thaliana] emb|CAB80144.1| putative protein [Arabidopsis thaliana]	7E-50
	CaF1_WIE_15_B_02	emb|CAB83305.1|	putative protein [Arabidopsis thaliana]	1E-49
	CaF1_WIE_53_D_06	gb|AAM61146.1|	unknown [Arabidopsis thaliana]	1E-49

	Contig120	gb|ABE91787.1|	Protein of unknown function DUF707 [Medicago truncatula]	3E-48
	CaF1_JIE_33_C_09	gb|ABO84551.1|	Protein of unknown function DUF506, plant [Medicago truncatula]	4E-48
	Contig11	gb|ABE79089.2|	Protein of unknown function DUF676, hydrolase-like [Medicago truncatula]	3E-47
	Contig13	gb|ABE88889.1|	Protein of unknown function DUF567 [Medicago truncatula]	6E-47
	CaF1_WIE_15_H_10	dbj|BAE62261.1|	unnamed protein product [Aspergillus oryzae]	6E-47
	CaF1_WIE_20_E_03	ref|NP_175129.3|	unknown protein [Arabidopsis thaliana] gb|AAP37679.1| At1g45150 [Arabidopsis thaliana] dbj|BAE99332.1| hypothetical protein [Arabidopsis thaliana]	2E-44
	CaF1_WIE_36_B_11	ref|NP_200619.1|	unknown protein [Arabidopsis thaliana] gb|AAL58914.1|AF462824_1 AT5g58110/k21l19_90 [Arabidopsis thaliana] dbj|BAB11003.1| unnamed protein product [Arabidopsis thaliana] gb|AAM19978.1| AT5g58110/k21l19_90 [Arabidopsis thaliana]	6E-44
	Contig259	gb|ABA40435.1|	unknown [Solanum tuberosum]	8E-44
	Contig110	gb|AAZ32857.1|	unknown [Medicago sativa]	1E-43
	CaF1_WIE_05_F_01	gb|AAO24648.1|	unknown protein [Phytophthora sojae]	1E-43
	Contig181	dbj|BAE61155.1|	unnamed protein product [Aspergillus oryzae]	2E-42
	CaF1_WIE_24_A_06	dbj|BAB02526.1|	unnamed protein product [Arabidopsis thaliana]	5E-38
	CaF1_JIE_09_E_02	gb|ABD33160.1|	Protein of unknown function DUF716 [Medicago truncatula]	6E-36
	Contig771	gb|ABA29157.1|	unknown [Pisum sativum]	7E-35
	Contig677	gb|ABC69764.1|	unknown [Vitis pseudoreticulata]	1E-34
	CaF1_WIE_33_A_02	gb|AAM62609.1|	unknown [Arabidopsis thaliana]	2E-33
	CaF1_WIE_32_H_08	ref|NP_191627.1|	unknown protein [Arabidopsis thaliana] emb|CAB82672.1| putative protein [Arabidopsis thaliana] dbj|BAC41904.1| unknown protein [Arabidopsis thaliana] gb|AAO64825.1| At3g60680 [Arabidopsis thaliana]	5E-33
	CaF1_JIE_02_C_04	emb|CAB75818.1|	putative protein [Arabidopsis thaliana]	3E-32
	Contig125	gb|AAL36394.1|	unknown protein [Arabidopsis thaliana] gb|AAQ89668.1| At1g77230 [Arabidopsis thaliana] dbj|BAD43411.1| hypothetical protein [Arabidopsis thaliana] dbj|BAD43428.1| hypothetical protein [Arabidopsis thaliana]	5E-32
	Contig397	ref|NP_199928.1|	unknown protein [Arabidopsis thaliana] dbj|BAA97377.1| unnamed protein product [Arabidopsis thaliana] gb|AAK76512.1| unknown protein [Arabidopsis thaliana] gb|AAM14313.1| unknown protein [Arabidopsis thaliana]	7E-32
	CaF1_JIE_05_E_01	gb|ABE80212.1|	Protein of unknown function DUF339 [Medicago truncatula]	7E-32
	Contig843	gb|AAL86349.1|	unknown protein [Arabidopsis thaliana]	2E-31
	CaF1_WIE_24_B_01	ref|NP_564660.1|	unknown protein [Arabidopsis thaliana] gb|AAM13069.1| unknown protein [Arabidopsis thaliana] gb|AAM61748.1| unknown [Arabidopsis thaliana]	2E-31
	CaF1_WIE_24_E_10	gb|ABB16967.1|	unknown [Solanum tuberosum]	2E-31
	CaF1_JIE_07_G_02	gb|ABD28486.1|	Protein of unknown function UPF0041 [Medicago truncatula]	2E-30
	CaF1_JIE_40_F_10	gb|ABE85501.1|	Protein of unknown function DUF810 [Medicago truncatula]	1E-29
	Contig246	ref|NP_563993.1|	unknown protein [Arabidopsis thaliana] gb|AAL59995.1| unknown protein [Arabidopsis thaliana] gb|AAM61212.1| unknown [Arabidopsis thaliana] gb|AAM67462.1| unknown protein [Arabidopsis thaliana] dbj|BAE99103.1| hypothetical protein [Arabidopsis thaliana]	2E-28
	Contig573	ref|NP_973636.1|	unknown protein [Arabidopsis thaliana]	4E-28
	CaF1_WIE_05_E_03	emb|CAB88266.1|	putative protein [Arabidopsis thaliana]	5E-28
	Contig463	gb|ABO79380.1|	Protein of unknown function DUF676, hydrolase-like [Medicago truncatula] gb|ABE80808.2| Protein of unknown function DUF676, hydrolase-like [Medicago truncatula]	6E-26
	CaF1_JIE_04_B_09	ref|NP_566858.1|	unknown protein [Arabidopsis thaliana] dbj|BAB02262.1| unnamed protein product [Arabidopsis thaliana] gb|AAK92723.1| unknown protein [Arabidopsis thaliana] gb|AAM45104.1| unknown protein [Arabidopsis thaliana]	1E-25
	CaF1_WIE_02_B_09	ref|NP_567210.1|	unknown protein [Arabidopsis thaliana] sp|O04616|Y4115_ARATH Protein At4g01150, chloroplast precursor gb|AAK63864.1|AF389292_1 AT4g01150/F2N1_18 [Arabidopsis thaliana] gb|AAB61025.1| A_IG002N01.18 gene product [Arabidopsis thaliana] emb|CAB80924.1| hypothetical protein [Arabidopsis thaliana] gb|AAM10278.1| AT4g01150/F2N1_18 [Arabidopsis thaliana]	2E-25
	Contig32	ref|NP_566599.1|	unknown protein [Arabidopsis thaliana] gb|AAK93587.1| unknown protein [Arabidopsis thaliana] gb|AAM14351.1| unknown protein [Arabidopsis thaliana] gb|AAM65658.1| unknown [Arabidopsis thaliana]	2E-24
	Contig580	gb|ABC69764.1|	unknown [Vitis pseudoreticulata]	5E-24
	Contig16	gb|ABO82817.1|	Protein of unknown function DUF668 [Medicago truncatula]	9E-24
	Contig233	ref|NP_850433.2|	unknown protein [Arabidopsis thaliana]	9E-24
	Contig312	gb|AAM61409.1|	unknown [Arabidopsis thaliana]	4E-23

	CaF1_JIE_04_H_03	gb|AAL86291.1|	unknown protein [Arabidopsis thaliana]	5E-22
	Contig380	ref|NP_567434.1|	unknown protein [Arabidopsis thaliana] sp|Q8VXX9|BETL1_ARATH Bet1 like protein At4g14600 gb|AAL67066.1| unknown protein [Arabidopsis thaliana] gb|AAM14339.1| unknown protein [Arabidopsis thaliana] gb|AAM63165.1| unknown [Arabidopsis thaliana] dbj|BAC43249.1| unknown protein [Arabidopsis thaliana]	9E-22
	Contig803	gb|ABO78644.1|	Protein of unknown function DUF543 [Medicago truncatula]	1E-21
	CaF1_WIE_40_F_01	ref|NP_564506.1|	unknown protein [Arabidopsis thaliana] sp|Q9SX77|UMP6_ARATH Unknown protein At1g47420, mitochondrial precursor gb|AAD46040.1|AC007519_25 ESTs gb|H36253 and gb|AA04251 come from this gene. [Arabidopsis thaliana] gb|AAK06877.1|AF344326_1 unknown protein [Arabidopsis thaliana] gb|AAK59453.1| unknown protein [Arabidopsis thaliana] gb|AAK96754.1| Unknown protein [Arabidopsis thaliana] gb|AAL34164.1| unknown protein [Arabidopsis thaliana] gb|AAM60959.1| unknown [Arabidopsis thaliana] gb|AAN15664.1| Unknown protein [Arabidopsis thaliana]	6E-21
	CaF1_WIE_46_B_08	ref|NP_191085.1|	unknown protein [Arabidopsis thaliana] emb|CAB75759.1| putative protein [Arabidopsis thaliana] gb|AAM20644.1| putative protein [Arabidopsis thaliana] gb|AAM91309.1| putative protein [Arabidopsis thaliana] gb|AAO43936.1| putative calcium homeostasis regulator [Arabidopsis thaliana]	2E-19
	Contig952	ref|NP_565024.1|	unknown protein [Arabidopsis thaliana]	5E-19
	CaF1_WIE_32_G_04	ref|NP_683481.1|	unknown protein [Arabidopsis thaliana] gb|AAL38694.1| unknown protein [Arabidopsis thaliana] gb|AAM20199.1| unknown protein [Arabidopsis thaliana] gb|AAM67283.1| unknown [Arabidopsis thaliana] dbj|BAC42463.1| unknown protein [Arabidopsis thaliana]	8E-17
	Contig477	ref|NP_196899.2|	unknown protein [Arabidopsis thaliana] ref|NP_001078580.1| unknown protein [Arabidopsis thaliana]	7E-14
	CaF1_JIE_08_G_07	ref|NP_566028.1|	unknown protein [Arabidopsis thaliana] gb|AAK48959.1|AF370532_1 Unknown protein [Arabidopsis thaliana] gb|AAL66926.1| unknown protein [Arabidopsis thaliana] gb|AAC31837.2| expressed protein [Arabidopsis thaliana]	2E-11
Miscellaneous	Contig129	emb|CAC43238.1|	calcium binding protein [Sesbania rostrata]	4E-12
	CaF1_WIE_48_A_04	ref|NP_196983.1|	binding [Arabidopsis thaliana] emb|CAC01878.1| putative protein [Arabidopsis thaliana] gb|AAM13237.1| putative protein [Arabidopsis thaliana] gb|AAM67181.1| unknown [Arabidopsis thaliana] gb|AAN65128.1| putative protein [Arabidopsis thaliana] dbj|BAD93942.1| hypothetical protein [Arabidopsis thaliana]	1E-19
	CaF1_WIE_25_E_01	gb|ABE91842.1|	Gonadotropin, beta chain; Gibberellin regulated protein [Medicago truncatula]	9E-11
	CaF1_WIE_10_H_10	gb|AAB32504.1|	root hair protein RH2 [Pisum sativum]	1E-14
	Contig805	gb|AY874423.1|	Fusarium oxysporum voucher VPRI 19292 mitochondrion, partial genome	0
	Contig893	gb|AY874423.1|	Fusarium oxysporum voucher VPRI 19292 mitochondrion, partial genome	0
	CaF1_WIE_23_B_04	gb|DQ008803.1|	Takhtajania perrieri large subunit ribosomal RNA gene, partial sequence; mitochondrial	0
	CaF1_JIE_34_B_07	gb|K03313.1|RIATL	Integrated Ri plasmid agropine (A. rhizogenes strain A4) complete TL-DNA and flanking plant (Convolvulus arvensis) DNA	1E-157
	CaF1_WIE_21_B_10	emb|CU405944.1|	Oryza rufipogon (W1943) cDNA clone: ORW1943C006H23, full insert sequence	1E-127
	CaF1_WIE_14_E_11	gb|AC141115.22|	Medicago truncatula clone mth2-16b23, complete sequence	1E-103
	CaF1_WIE_39_C_10	gb|AY847700.1|	Catharanthus roseus clone CrP15 T-DNA sequence	9E-98
	CaF1_WIE_39_F_09	gb|AY847700.1|	Catharanthus roseus clone CrP15 T-DNA sequence	1E-96
	CaF1_WIE_45_C_07	gb|AY032742.1|	Fusarium sporotrichioides guanine nucleotide-binding protein mRNA, complete cds	5E-93
	CaF1_WIE_46_G_07	emb|AJ489609.1|CAR489609	Cicer arietinum mRNA for alpha-expansin 4 (expa4 gene)	2E-88
	CaF1_WIE_29_B_07	ref|NP_001048802.1|	Os03g0123100 [Oryza sativa (japonica cultivar-group)] gb|AAN74837.1| Putative ubiquitin-conjugating enzyme [Oryza sativa (japonica cultivar-group)] gb|ABF93717.1| Ubiquitin-conjugating enzyme E2 I, putative, expressed [Oryza sativa (japonica cultivar-group)] dbj|BAF10716.1| Os03g0123100 [Oryza sativa (japonica cultivar-group)] gb|EAY88329.1| hypothetical protein OsI_009562 [Oryza sativa (indica cultivar-group)] gb|EAZ25409.1| hypothetical protein OsJ_008892 [Oryza sativa (japonica cultivar-group)]	4E-83
	CaF1_WIE_38_B_11	gb|DQ485185.1|	Catharanthus trichophyllus genotype CtN58 microsatellite CATR10 sequence	2E-82
	CaF1_WIE_38_C_11	gb|DQ485185.1|	Catharanthus trichophyllus genotype CtN58 microsatellite CATR10 sequence	2E-82
	CaF1_WIE_38_G_11	gb|DQ485185.1|	Catharanthus trichophyllus genotype CtN58 microsatellite CATR10 sequence	2E-82

	CaF1_WIE_38_H_11	gb|DQ485185.1|	Catharanthus trichophyllus genotype CtN58 microsatellite CATR10 sequence	2E-82
	CaF1_WIE_10_C_07	emb|CR954185.3|	Medicago truncatula chromosome 5 clone mth4-20m5, COMPLETE SEQUENCE	4E-80
	CaF1_JIE_06_C_10	ref|NP_001050135.1|	Os03g0355600 [Oryza sativa (japonica cultivar-group)] dbj|BAF12049.1| Os03g0355600 [Oryza sativa (japonica cultivar-group)]	2E-77
	Contig119	gb|AAT08648.1|	ADP-ribosylation factor [Hyacinthus orientalis]	2E-76
	CaF1_WIE_46_A_09	gb|AC150798.3|	Medicago truncatula chromosome 2 clone mth2-33b11, complete sequence	3E-76
	CaF1_JIE_03_G_01	gb|ABE84189.1|	SYNC1 protein, related [Medicago truncatula]	3E-75
	Contig633	ref|NP_001047515.1|	Os02g0634800 [Oryza sativa (japonica cultivar-group)] dbj|BAD25096.1| putative ubiquitin-conjugating enzyme E2 [Oryza sativa (japonica cultivar-group)] dbj|BAD25314.1| putative ubiquitin-conjugating enzyme E2 [Oryza sativa (japonica cultivar-group)] dbj|BAF09429.1| Os02g0634800 [Oryza sativa (japonica cultivar-group)]	9E-75
	Contig509	gb|ABE93756.1|	Longin-like [Medicago truncatula]	4E-74
	Contig368	gb|AC145156.61|	Medicago truncatula clone mth2-7h6, complete sequence	6E-74
	CaF1_WIE_03_H_01	emb|CT971488.3|	M.truncatula DNA sequence from clone MTH2-154I11 on chromosome 3, complete sequence	6E-74
	Contig52	gb|AAD32141.1|AF123503_1	Nt-gh3 deduced protein [Nicotiana tabacum]	3E-72
	Contig845	gb|AAO61674.1|	AKIN gamma [Medicago truncatula]	4E-72
	CaF1_WIE_13_B_06	emb|AJ278505.1|CAR278505	Cicer arietinum mRNA for putative 14-kDa proline-rich protein (ORF1)	3E-71
	CaF1_JIE_41_G_02	gb|ABP03273.1|	Uncharacterized Cys-rich domain [Medicago truncatula]	1E-69
	Contig198	gb|ABE80069.1|	Root cap [Medicago truncatula]	2E-69
	CaF1_WIE_40_A_07	gb|ABE80525.1|	Pentatricopeptide repeat [Medicago truncatula]	3E-68
	CaF1_WIE_28_C_03	gb|AC121235.20|	Medicago truncatula clone mth2-21k24, complete sequence	4E-65
	Contig825	gb|DQ485193.1|	Nerium oleander microsatellite CATR25 sequence	3E-63
	CaF1_JIE_19_G_04	gb|ABE79642.1|	Cellular retinaldehyde-binding/triple function, N-terminal [Medicago truncatula]	2E-62
	Contig581	emb|CAA66108.1|	specific tissue protein 1 [Cicer arietinum]	7E-62
	Contig398	gb|ABE88379.1|	Nascent polypeptide-associated complex NAC [Medicago truncatula]	1E-61
	CaF1_JIE_15_D_07	gb|AAU05467.1|	At5g22850 [Arabidopsis thaliana] gb|AAV59285.1| At5g22850 [Arabidopsis thaliana]	1E-61
	CaF1_WIE_05_E_04	gb|AC174142.11|	Medicago truncatula clone mth2-69j4, complete sequence	8E-61
	Contig604	gb|AC166897.12|	Medicago truncatula clone mth2-64l14, complete sequence	1E-60
	CaF1_JIE_29_F_09	gb|AC157490.18|	Medicago truncatula clone mth2-123f23, complete sequence	2E-60
	Contig307	emb|CR932967.2|	Medicago truncatula chromosome 5 clone mth2-49e21, COMPLETE SEQUENCE	4E-60
	CaF1_JIE_22_E_07	gb|AAK82520.1|	AT5g24810/F6A4_20 [Arabidopsis thaliana]	6E-60
	Contig676	emb|CAA72315.1|	putative 21kD protein precursor [Medicago sativa subsp. x varia]	1E-59
	CaF1_WIE_26_F_05	gb|AC155894.5|	Medicago truncatula chromosome 7 BAC clone mth2-67b7, complete sequence	2E-58
	Contig358	gb|ABE77790.2|	Patatin [Medicago truncatula]	2E-56
	Contig905	sp|Q9ZSW9|TCTP_HEVBR	Translationally-controlled tumor protein homolog (TCTP) gb|AAD10032.1| translationally controlled tumor protein [Hevea brasiliensis]	3E-53
	CaF1_WIE_34_C_05	gb|ABK15530.1|	guanylyl cyclase [Glycine max]	3E-53
	Contig141	gb|ABE77505.1|	DECOY (exp=-1; , putative [Medicago truncatula]	9E-53
	CaF1_WIE_44_C_04	ref|NP_001050957.1|	Os03g0691800 [Oryza sativa (japonica cultivar-group)] gb|AAT76985.1| putative HIPL1 protein [Oryza sativa (japonica cultivar-group)] gb|ABF98298.1| HIPL1 protein precursor, putative, expressed [Oryza sativa (japonica cultivar-group)] dbj|BAF12871.1| Os03g0691800 [Oryza sativa (japonica cultivar-group)] gb|EAZ28206.1| hypothetical protein OsJ_011689 [Oryza sativa (japonica cultivar-group)]	1E-51
	Contig628	emb|CAC35070.1|	VIP3 protein [Zea mays]	3E-51
	Contig819	gb|ABO84303.1|	LOC548366 protein, related [Medicago truncatula]	1E-50
	Contig822	ref|NP_001065513.1|	Os10g0580400 [Oryza sativa (japonica cultivar-group)] gb|AAG46170.1|AC018727_22 putative urea active transport protein [Oryza sativa] gb|AAP55189.1| SSS sodium solute transporter superfamily protein, expressed [Oryza sativa (japonica cultivar-group)] gb|AAR27948.1 DUR3 [Oryza sativa (japonica cultivar-group)] dbj|BAF27350.1| Os10g0580400 [Oryza sativa (japonica cultivar-group)] gb|EAZ17111.1| hypothetical protein OsJ_031320 [Oryza sativa (japonica cultivar-group)]	2E-50

	CaF1_JIE_09_H_09	ref|NP_194420.1|	AT-HF (Arabidopsis thaliana HisF protein) sp|Q9SZ30|HIS5_ARATH Imidazole glycerol phosphate synthase hisHF, chloroplast precursor (IGP synthase) (ImGP synthase) (IGPS) [Includes: Glutamine amidotransferase ; Cyclase] emb|CAB36536.1| glutamine amidotransferase/cyclase [Arabidopsis thaliana] emb|CAB79545.1| glutamine amidotransferase/cyclase [Arabidopsis thaliana]	2E-49
	CaF1_WIE_24_B_07	gb|ABE88784.1|	SH3 [Medicago truncatula]	6E-49
	CaF1_WIE_27_G_09	gb|ABE92592.1|	RHO protein GDP dissociation inhibitor [Medicago truncatula]	4E-48
	Contig439	gb|AC155885.2|	Medicago truncatula chromosome 7 clone mth2-10p23, complete sequence	1E-47
	Contig386	gb|AC140850.20|	Medicago truncatula clone mth2-11i23, complete sequence	5E-47
	CaF1_WIE_46_D_05	gb|ABE92992.1|	Exostosin-like [Medicago truncatula]	6E-47
	Contig92	emb|CU302348.1|	Medicago truncatula chromosome 5 clone mth2-45a24, COMPLETE SEQUENCE	1E-46
	CaF1_WIE_49_B_02	emb|CT954252.6|	M.truncatula DNA sequence from clone MTH2-60M21 on chromosome 3, complete sequence	1E-46
	Contig528	gb|AC124218.18|	Medicago truncatula clone mth2-30b20, complete sequence	2E-46
	Contig820	emb|CT027663.2|	Medicago truncatula chromosome 5 clone mth2-139g23, COMPLETE SEQUENCE	3E-46
	Contig516	ref|XP_001234258.1|	PREDICTED: similar to bacterial IS-element [Gallus gallus]	7E-46
	CaF1_WIE_25_A_01	ref|NP_564656.1|	LEM3 (ligand-effect modulator 3) family protein / CDC50 family protein [Arabidopsis thaliana] gb|AAD25612.1|AC005287_14 Unknown protein [Arabidopsis thaliana] gb|AAL38602.1|AF446869_1 At1g54320/F20D21_50 [Arabidopsis thaliana] gb|AAK74030.1| At1g54320/F20D21_50 [Arabidopsis thaliana] gb|AAK96636.1| At1g54320/F20D21_50 [Arabidopsis thaliana]	8E-46
	Contig407	gb|AC140914.20|	Medicago truncatula clone mth2-18h17, complete sequence	1E-45
	Contig792	ref|NP_001062502.1|	Os08g0559400 [Oryza sativa (japonica cultivar-group)] dbj|BAB62328.1| cyclophilin [Oryza sativa (japonica cultivar-group)] dbj|BAB62329.1| cyclophilin [Oryza sativa (japonica cultivar-group)] dbj|BAD13138.1| cyclophilin [Oryza sativa (japonica cultivar-group)] dbj|BAF24416.1| Os08g0559400 [Oryza sativa (japonica cultivar-group)] gb|EAZ43652.1| hypothetical protein OsJ_027135 [Oryza sativa (japonica cultivar-group)]	2E-45
	CaF1_WIE_30_D_11	emb|CU207236.5|	M.truncatula DNA sequence from clone MTH2-56B11 on chromosome 3, complete sequence	2E-45
	CaF1_JIE_07_C_06	gb|AC186678.3|	Medicago truncatula chromosome 7 BAC clone mth2-51a12, complete sequence	2E-44
	CaF1_WIE_06_F_11	dbj|BAE71282.1|	putative receptor-like GPI-anchored protein 2 [Trifolium pratense]	4E-44
	Contig204	gb|ABE78019.1|	Concanavalin A-like lectin/glucanase [Medicago truncatula]	5E-44
	CaF1_WIE_09_F_07	gb|AC167711.1|	Medicago truncatula chromosome 7 clone mth2-167p21, complete sequence	5E-44
	CaF1_WIE_20_G_01	emb|CR962121.2|	Medicago truncatula chromosome 5 clone mte1-77f5, COMPLETE SEQUENCE	8E-44
	CaF1_WIE_56_D_10	ref|NP_001058575.1|	Os06g0714500 [Oryza sativa (japonica cultivar-group)] dbj|BAD53565.1| putative spastin protein [Oryza sativa (japonica cultivar-group)] dbj|BAF20489.1| Os06g0714500 [Oryza sativa (japonica cultivar-group)] gb|EAZ02349.1| hypothetical protein OsI_023581 [Oryza sativa (indica cultivar-group)] gb|EAZ38273.1| hypothetical protein OsJ_021756 [Oryza sativa (japonica cultivar-group)]	8E-44
	CaF1_WIE_01_D_01	gb|AC126786.22|	Medicago truncatula clone mth2-8c2, complete sequence	1E-43
	Contig169	emb|CR962130.3|	Medicago truncatula chromosome 5 clone mte1-63h20, COMPLETE SEQUENCE	2E-43
	Contig524	gb|ABE93901.1|	Nonaspanin (TM9SF) [Medicago truncatula]	2E-43
	CaF1_WIE_55_B_04	emb|CR962124.2|	Medicago truncatula chromosome 5 clone mth2-44c15, COMPLETE SEQUENCE	2E-43
	CaF1_WIE_12_A_11	ref|NP_171699.1|	pentatricopeptide (PPR) repeat-containing protein [Arabidopsis thaliana] gb|AAF76475.1|AC020622_9 Contains similarity to an unknown protein gi|AAD26479 from Arabidopsis thaliana BAC gb|AC007169 and contains multiple PPR PF|01535 repeats gb|AAQ56795.1| At1g01970 [Arabidopsis thaliana] dbj|BAE99830.1| hypothetical protein [Arabidopsis thaliana]	5E-43
	Contig424	gb|ABR25719.1|	ADP-ribosylation factor 1 [Oryza sativa (indica cultivar-group)]	7E-43
	Contig848	gb|AC139854.21|	Medicago truncatula clone mth2-16e16, complete sequence	7E-43
	Contig34	emb|CAE01583.2|	OSJNBa0068L06.9 [Oryza sativa (japonica cultivar-group)] emb|CAH65755.1| OSIGBa0123D13.4 [Oryza sativa (indica cultivar-group)] gb|EAY92749.1| hypothetical protein OsI_013982 [Oryza sativa (indica cultivar-group)] gb|EAZ29424.1| hypothetical protein OsJ_012907 [Oryza sativa (japonica cultivar-group)]	6E-42
	CaF1_WIE_19_G_11	emb|CAB96990.1|	putative 14-kDa proline-rich protein [Cicer arietinum]	6E-42

	CaF1_WIE_43_H_03	emb|CAH66506.1|	OSIGBa0111I14.1 [Oryza sativa (indica cultivar-group)]	7E-42
	Contig157	ref|NP_001045159.1|	Os01g0911200 [Oryza sativa (japonica cultivar-group)] dbj|BAD82429.1| putative ribophorin II precursor [Oryza sativa (japonica cultivar-group)] dbj|BAF07073.1| Os01g0911200 [Oryza sativa (japonica cultivar-group)]	8E-42
	Contig205	ref|NP_001042188.1|	Os01g0178000 [Oryza sativa (japonica cultivar-group)] dbj|BAB63467.1| putative aspartate aminotransferase [Oryza sativa (japonica cultivar-group)] dbj|BAF04102.1| Os01g0178000 [Oryza sativa (japonica cultivar-group)] gb|EAY72764.1| hypothetical protein OsI_000611 [Oryza sativa (indica cultivar-group)] gb|EAZ10762.1| hypothetical protein OsJ_000587 [Oryza sativa (japonica cultivar-group)]	8E-42
	CaF1_WIE_04_G_10	gb|AC174142.11|	Medicago truncatula clone mth2-69j4, complete sequence	8E-42
	CaF1_WIE_38_C_09	gb|DQ485192.1|	Thevetia peruviana microsatellite CATR25 sequence	2E-41
	Contig468	gb|AC148528.15|	Medicago truncatula clone mth2-53h4, complete sequence	3E-41
	Contig972	gb|ABO82310.1|	Dimerisation [Medicago truncatula]	3E-41
	CaF1_WIE_49_D_06	emb|CR962137.2|	Medicago truncatula chromosome 5 clone mte1-10p15, COMPLETE SEQUENCE	5E-41
	CaF1_WIE_52_F_05	emb|CR940305.16|	M.truncatula DNA sequence from clone MTH2-28N4 on chromosome 3, complete sequence	5E-41
	Contig462	gb|ABB89773.1|	At3g12550-like protein [Boechera stricta]	6E-41
	CaF1_JIE_16_H_10	gb|AC166093.12|	Medicago truncatula clone mth2-17k6, complete sequence	2E-40
	CaF1_WIE_37_G_09	gb|AC140914.20|	Medicago truncatula clone mth2-18h17, complete sequence	2E-39
	Contig956	gb|AC174326.12|	Medicago truncatula chromosome 8 clone mth2-107i14, complete sequence	1E-38
	CaF1_JIE_36_E_08	dbj|BAB86895.1|	syringolide-induced protein B15-3-5 [Glycine max]	2E-38
	CaF1_WIE_41_H_04	dbj|AP004505.1|	Lotus japonicus genomic DNA, chromosome 3, clone:LjT10E18, TM0035, complete sequence	4E-38
	Contig821	gb|DQ383816.1|	Lactuca sativa cultivar Salinas chloroplast, complete genome	5E-38
	Contig882	gb|ABE84580.1|	fiber protein Fb15 [Medicago truncatula]	7E-38
	CaF1_JIE_37_A_07	gb|AC146746.15|	Medicago truncatula clone mth2-108p9, complete sequence	9E-38
	CaF1_WIE_55_B_09	gb|AAP22955.1|	Potyvirus VPg interacting protein [Pisum sativum]	4E-62
	CaF1_WIE_20_C_01	gb|AC137838.40|	Medicago truncatula clone mth2-34l12, complete sequence	9E-38
	Contig717	gb|ABE77920.1|	Rhodanese-like [Medicago truncatula] gb|ABE92275.1| Rhodanese-like [Medicago truncatula]	4E-37
	Contig866	emb|CAA80983.1|	narbonin [Vicia narbonensis]	7E-37
	CaF1_WIE_21_E_10	gb|AC144930.26|	Medicago truncatula clone mth2-15e11, complete sequence	2E-36
	CaF1_WIE_47_D_01	gb|ABP03277.1|	UBX; PUG; Zinc finger, C2H2-type [Medicago truncatula]	1E-35
	Contig420	emb|CR940305.16|	M.truncatula DNA sequence from clone MTH2-28N4 on chromosome 3, complete sequence	4E-35
	Contig569	emb|CAA09589.1|	pepc2 [Vicia faba]	7E-35
	Contig422	gb|AC126019.16|	Medicago truncatula clone mth2-22p22, complete sequence	1E-34
	Contig824	gb|AAZ50401.1|	Sus [Agrobacterium tumefaciens]	3E-34
	CaF1_WIE_52_A_11	dbj|AP006629.1|	Lotus japonicus genomic DNA, chromosome 2, clone:LjT10B11, TM0008, complete sequence	7E-34
	CaF1_JIE_19_F_04	gb|AC151956.5|	Medicago truncatula clone mth2-52p17, complete sequence	9E-34
	Contig390	gb|ABD32881.1|	Nascent polypeptide-associated complex NAC; UBA-like [Medicago truncatula]	1E-33
	Contig671	ref|XM_381601.1|	Gibberella zeae PH-1 chromosome 1 PMA1_NEUCR Plasma membrane ATPase (Proton pump) (FG01425.1) partial mRNA	2E-33
	Contig639	ref|NP_001060360.1|	Os07g0631100 [Oryza sativa (japonica cultivar-group)] sp|Q8LHP0|ELOF1_ORYSJ Transcription elongation factor 1 homolog dbj|BAC10134.1| unknown protein [Oryza sativa (japonica cultivar-group)] dbj|BAF22274.1| Os07g0631100 [Oryza sativa (japonica cultivar-group)] gb|EAY85182.1| hypothetical protein OsI_006415 [Oryza sativa (indica cultivar-group)] gb|EAZ40760.1| hypothetical protein OsJ_024243 [Oryza sativa (japonica cultivar-group)]	3E-33
	CaF1_WIE_38_F_10	gb|DQ485193.1|	Nerium oleander microsatellite CATR25 sequence	7E-33
	CaF1_WIE_23_F_11	gb|ABE78146.1|	E1 protein and Def2/Der2 allergen [Medicago truncatula] gb|ABE83819.1| E1 protein and Def2/Der2 allergen [Medicago truncatula]	1E-32
	CaF1_WIE_21_F_10	emb|CT967319.2|	M.truncatula DNA sequence from clone MTH2-59J17 on chromosome 3, complete sequence	4E-32
	Contig548	emb|CR936368.12|	M.truncatula DNA sequence from clone MTH2-4O11 on chromosome 3, complete sequence	5E-32
	Contig610	gb|AC123976.23|	Medicago truncatula clone mth2-30j23, complete sequence	5E-32

	CaF1_JIE_19_A_01	gb|ABG21940.1|	AGR_C_5039p, putative, expressed [Oryza sativa (japonica cultivar-group)] gb|ABG21941.1| AGR_C_5039p, putative, expressed [Oryza sativa (japonica cultivar-group)]	4E-31
	CaF1_WIE_38_E_07	gb|DQ485193.1|	Nerium oleander microsatellite CATR25 sequence	4E-31
	CaF1_WIE_28_H_05	gb|ABE92593.1|	Membrane attack complex component/perforin/complement C9 [Medicago truncatula]	6E-31
	Contig939	ref|XP_001216717.1|	predicted protein [Aspergillus terreus NIH2624] gb|EAU31269.1| predicted protein [Aspergillus terreus NIH2624]	1E-30
	CaF1_WIE_49_G_05	gb|AC144340.30|	Medicago truncatula clone mth2-7k2, complete sequence	3E-30
	Contig408	ref|XM_381275.1|	Gibberella zeae PH-1 chromosome 1 RAN_BRUMA GTP-binding nuclear protein RAN/TC4 (FG01099.1) partial mRNA	7E-30
	Contig647	gb|ABE87918.1|	Cupin region [Medicago truncatula]	8E-30
	Contig336	ref|NP_001048662.1|	Os03g0102400 [Oryza sativa (japonica cultivar-group)] dbj|BAF10576.1| Os03g0102400 [Oryza sativa (japonica cultivar-group)]	2E-29
	CaF1_WIE_48_D_10	gb|AC148237.7|	Medicago truncatula clone mth2-1o14, complete sequence	2E-29
	CaF1_WIE_01_D_09	gb|ABO82002.1|	AIG1 [Medicago truncatula]	3E-29
	CaF1_WIE_26_F_11	gb|AAX85979.1|	NAC2 protein [Glycine max] gb|AAY46122.1| NAC domain protein NAC2 [Glycine max]	3E-29
	CaF1_WIE_51_C_06	gb|DQ117568.1|	Phaseolus vulgaris clone PvD34, mRNA sequence	3E-29
	Contig216	gb|AAL06505.1|AF412052_1	At2g43970/F6E13.10 [Arabidopsis thaliana]	4E-29
	CaF1_WIE_28_G_09	gb|ABE82748.1|	CCT [Medicago truncatula]	4E-29
	Contig772	emb|Y15372.1|MTY15372	Medicago truncatula mRNA for MtN4 gene, partial	1E-28
	CaF1_WIE_38_D_10	gb|DQ485193.1|	Nerium oleander microsatellite CATR25 sequence	1E-28
	Contig716	emb|CU075908.9|	M.truncatula DNA sequence from clone MTH2-31B23 on chromosome 3, complete sequence	2E-28
	CaF1_JIE_40_E_01	gb|AAM19795.1|	At2g04030/F3C11.14 [Arabidopsis thaliana]	5E-28
	Contig185	gb|AC134322.25|	Medicago truncatula clone mth2-17i21, complete sequence	7E-28
	CaF1_JIE_13_C_01	gb|ABD32291.1|	Uncharacterized Cys-rich domain [Medicago truncatula]	1E-27
	CaF1_JIE_31_F_03	gb|AC167403.2|	Medicago truncatula chromosome 7 BAC clone mte1-14f12, complete sequence	3E-27
	CaF1_WIE_46_A_06	gb|ABQ11262.1|	mago nashi-like protein 1 [Physalis pubescens] gb|ABQ11264.1| mago nashi-like protein 1 [Physalis pubescens]	4E-27
	CaF1_JIE_23_A_03	sp|Q850K7|EXLB1_ORYSJ	Expansin-like B1 precursor (OsEXLB1) (Expensin-related 1) (OsEXPR1) (OsaEXPb3.1) gb|EAZ03949.1| hypothetical protein OsI_025181 [Oryza sativa (indica cultivar-group)] gb|EAZ39893.1| hypothetical protein OsJ_023376 [Oryza sativa (japonica cultivar-group)]	5E-27
	CaF1_WIE_49_H_08	dbj|AP004534.1|	Lotus japonicus genomic DNA, chromosome 4, clone:LjT14P20, TM0087, complete sequence	5E-27
	CaF1_WIE_56_B_03	ref|NP_001046176.1|	Os02g0194200 [Oryza sativa (japonica cultivar-group)] dbj|BAD15406.1| KH domain-containing protein-like [Oryza sativa (japonica cultivar-group)] dbj|BAF08090.1| Os02g0194200 [Oryza sativa (japonica cultivar-group)] gb|EAY84850.1| hypothetical protein OsI_006083 [Oryza sativa (indica cultivar-group)] gb|EAZ22072.1| hypothetical protein OsJ_005555 [Oryza sativa (japonica cultivar-group)]	7E-27
	Contig897	gb|AAR14273.1|	predicted protein [Populus alba x Populus tremula]	9E-27
	Contig1	gb|AC171534.4|	Medicago truncatula chromosome 7 clone mth2-90a20, complete sequence	3E-26
	CaF1_JIE_32_H_02	gb|AC148397.13|	Medicago truncatula clone mth2-22h4, complete sequence	3E-26
	CaF1_JIE_35_F_10	gb|AC175685.3|	Medicago truncatula chromosome 2 BAC clone mte1-55k6, complete sequence	6E-26
	CaF1_WIE_47_E_07	gb|AC149579.11|	Medicago truncatula clone mth2-99d8, complete sequence	2E-25
	CaF1_WIE_20_G_06	emb|CU179904.1|	Medicago truncatula chromosome 5 clone mth2-104i2, COMPLETE SEQUENCE	3E-25
	Contig300	gb|EF416175.1|	Sesbania rostrata cDNA-AFLP fragment 043BT43M21-641.8 genomic sequence	6E-25
	CaF1_WIE_12_G_03	gb|AAS38575.1|	short-chain dehydrogenase Tic32 [Pisum sativum]	6E-25
	CaF1_WIE_38_H_06	gb|AC159145.4|	Medicago truncatula chromosome 2 BAC clone mth2-67e10, complete sequence	6E-25
	Contig493	gb|AC119419.9|	Medicago truncatula clone mth2-6b12, complete sequence	1E-24
	CaF1_JIE_34_B_06	gb|AC151621.20|	Medicago truncatula clone mth2-14p3, complete sequence	1E-24
	CaF1_WIE_35_G_02	emb|CU302347.1|	Medicago truncatula chromosome 5 clone mth2-5p5, COMPLETE SEQUENCE	1E-24
	CaF1_WIE_30_B_03	dbj|AP004505.1|	Lotus japonicus genomic DNA, chromosome 3, clone:LjT10E18, TM0035, complete sequence	2E-24
	Contig370	ref|NP_001054396.1|	Os05g0103600 [Oryza sativa (japonica cultivar-group)] gb|AAS88829.1| putative ankyrin protein [Oryza sativa (japonica cultivar-group)] dbj|BAF16310.1| Os05g0103600 [Oryza sativa (japonica cultivar-group)]	4E-24

	CaF1_WIE_24_A_11	gb|AC165430.3|	Medicago truncatula chromosome 2 BAC clone mth2-67a21, complete sequence	5E-24
	CaF1_WIE_37_G_01	dbj|AP004973.1|	Lotus japonicus genomic DNA, chromosome 3, clone:LjT41A07, TM0155b, complete sequence	5E-24
	Contig279	dbj|AK247755.1|	Solanum lycopersicum cDNA, clone: LEFL2005N13, HTC in fruit	8E-24
	CaF1_WIE_01_C_05_W1,g	gb|AC150843.16|	Medicago truncatula clone mth2-103p8, complete sequence	1E-23
	Contig547	gb|AC139745.35|	Medicago truncatula clone mth2-17d15, complete sequence	2E-23
	Contig922	emb|CT009479.4|	M.truncatula DNA sequence from clone MTH2-164E3 on chromosome 3, complete sequence	2E-23
	CaF1_WIE_25_D_07	gb|AC139745.35|	Medicago truncatula clone mth2-17d15, complete sequence	3E-23
	CaF1_WIE_45_H_01	gb|EF025129.1|	Medicago truncatula PHD6 mRNA, complete cds	3E-23
	Contig253	gb|BT013115.1|	Lycopersicon esculentum clone 114402R, mRNA sequence	4E-23
	Contig162	gb|ABE92863.1|	F-actin capping protein, alpha subunit [Medicago truncatula]	5E-23
	CaF1_JIE_17_C_11	gb|AC146573.21|	Medicago truncatula clone mth2-145j1, complete sequence	5E-23
	CaF1_JIE_07_F_10	gb|AC140550.31|	Medicago truncatula clone mth2-54a24, complete sequence	6E-23
	Contig565	gb|AC134822.19|	Medicago truncatula clone mth2-15j20, complete sequence	1E-22
	CaF1_WIE_27_C_10	gb|AAZ67970.1|	At1g63940 [Arabidopsis thaliana] gb|AAZ67971.1| At1g63940 [Arabidopsis thaliana] gb|AAZ67972.1| At1g63940 [Arabidopsis thaliana] gb|AAZ67973.1| At1g63940 [Arabidopsis thaliana] gb|AAZ67974.1| At1g63940 [Arabidopsis thaliana] gb|AAZ67975.1| At1g63940 [Arabidopsis thaliana] gb|AAZ67976.1| At1g63940 [Arabidopsis thaliana] gb|AAZ67977.1| At1g63940 [Arabidopsis thaliana] gb|AAZ67978.1| At1g63940 [Arabidopsis thaliana] gb|AAZ67979.1| At1g63940 [Arabidopsis thaliana] gb|AAZ67980.1| At1g63940 [Arabidopsis thaliana] gb|AAZ67981.1| At1g63940 [Arabidopsis thaliana] gb|AAZ67982.1| At1g63940 [Arabidopsis thaliana] gb|AAZ67983.1| At1g63940 [Arabidopsis thaliana] gb|AAZ67984.1| At1g63940 [Arabidopsis thaliana] gb|AAZ67985.1| At1g63940-like protein [Arabidopsis lyrata]	1E-22
	CaF1_WIE_36_E_07	gb|AC146649.17|	Medicago truncatula clone mth2-10d6, complete sequence	1E-22
	Contig109	gb|ABP02189.1|	CBS [Medicago truncatula]	2E-22
	Contig357	dbj|BAE71282.1|	putative receptor-like GPI-anchored protein 2 [Trifolium pratense]	2E-22
	Contig607	ref|NM_123209.3|	Arabidopsis thaliana GRF3 (GENERAL REGULATORY FACTOR 3); protein phosphorylated amino acid binding (GRF3) mRNA, complete cds	2E-22
	CaF1_JIE_32_B_05	gb|AC137822.30|	Medicago truncatula clone mth2-31e20, complete sequence	3E-22
	CaF1_WIE_47_A_02	gb|AC148171.16|	Medicago truncatula clone mth2-30a2, complete sequence	4E-22
	Contig753	gb|AC187294.1|	Glycine max clone gmw1-105h23, complete sequence	2E-21
	CaF1_WIE_10_D_09	dbj|AK246309.1|	Solanum lycopersicum cDNA, clone: FC06CB10, HTC in fruit	4E-21
	CaF1_WIE_16_F_05	gb|AC159805.23|	Glycine max clone gmw2-173d12, complete sequence	5E-21
	Contig850	gb|ABE85139.1|	TPR repeat [Medicago truncatula]	7E-21
	CaF1_JIE_20_D_05	gb|AC147430.9|	Medicago truncatula clone mth2-71e6, complete sequence	1E-20
	CaF1_WIE_18_A_09	emb|CT967318.5|	M.truncatula DNA sequence from clone MTH2-3P14 on chromosome 3, complete sequence	1E-20
	CaF1_WIE_12_D_07	gb|ABR80314.1|	putative transposase [Klebsiella pneumoniae subsp. pneumoniae MGH 78578] gb|ABR80449.1| mercuric resistance protein [Klebsiella pneumoniae subsp. pneumoniae MGH 78578]	6E-20
	Contig513	ref|NP_564656.1|	LEM3 (ligand-effect modulator 3) family protein / CDC50 family protein [Arabidopsis thaliana] gb|AAD25612.1|AC005287_14 Unknown protein [Arabidopsis thaliana] gb|AAL38602.1|AF446869_1 At1g54320/F20D21_50 [Arabidopsis thaliana] gb|AAK74030.1| At1g54320/F20D21_50 [Arabidopsis thaliana] gb|AAK96636.1| At1g54320/F20D21_50 [Arabidopsis thaliana]	1E-19
	CaF1_WIE_18_H_02	gb|ABO80203.1|	Homeodomain-related [Medicago truncatula]	1E-19
	CaF1_JIE_31_F_11	gb|AAM49801.1|	GFA2 [Arabidopsis thaliana]	1E-18
	CaF1_JIE_30_D_06	gb|ABE77505.1|	DECOY (exp=-1; , putative [Medicago truncatula]	4E-18
	CaF1_WIE_28_F_08	gb|ABC86745.1|	pollen-specific protein [Vitis pseudoreticulata]	1E-17
	CaF1_WIE_29_E_06	emb|CAA16672.1|	predicted protein [Arabidopsis thaliana]	1E-17
	CaF1_JIE_08_G_10	dbj|BAD24657.1|	xylogen protein 1 [Zinnia elegans]	2E-17
	CaF1_WIE_32_B_02	gb|ABE83853.1|	Concanavalin A-like lectin/glucanase [Medicago truncatula]	2E-17
	CaF1_WIE_54_F_05	emb|CAJ31277.1|	autophagy protein 5 [Glycine max]	3E-15
	Contig525	dbj|BAA25187.1|	ARG10 [Vigna radiata]	6E-15
	CaF1_WIE_29_H_11	gb|ABE91475.2|	Glycine-rich protein, putative [Medicago truncatula]	1E-14
	CaF1_WIE_49_D_04	ref|NP_851260.1|	ATHDH (HISTIDINOL DEHYDROGENASE) [Arabidopsis thaliana]	1E-14
	Contig355	gb|ABE80340.1|	TonB box, N-terminal; Tetratricopeptide-like helical [Medicago truncatula]	4E-14
	CaF1_WIE_35_D_02	ref|NP_195785.1|	macrophage migration inhibitory factor family protein / MIF family protein [Arabidopsis thaliana] emb|CAB82281.1| light-inducible protein ATLS1 [Arabidopsis thaliana] gb|AAL32937.1| light-inducible protein ATLS1 [Arabidopsis thaliana] gb|AAM10137.1| light-inducible protein ATLS1 [Arabidopsis thaliana]	9E-14
	Contig175	gb|ABE93060.2|	CD9/CD37/CD63 antigen [Medicago truncatula]	1E-13

	Contig690	emb|CAA78515.1|	dehydrin-cognate [Pisum sativum]	1E-13
	CaF1_WIE_23_A_03	emb|CAA79177.1|	Tumor protein [Arabidopsis thaliana]	3E-12
	Contig526	gb|ABE85161.1|	Nascent polypeptide-associated complex NAC; UBA-like [Medicago truncatula]	4E-12
	CaF1_WIE_05_G_03	ref|XP_453838.1|	unnamed protein product [Kluyveromyces lactis] ref|XP_453844.1| unnamed protein product [Kluyveromyces lactis] emb|CAH00934.1| unnamed protein product [Kluyveromyces lactis NRRL Y-1140] emb|CAH00940.1| unnamed protein product [Kluyveromyces lactis NRRL Y-1140]	4E-12
	CaF1_JIE_16_H_02	dbj|BAD94495.1|	sigma-like factor [Arabidopsis thaliana]	8E-12
	CaF1_WIE_06_C_04	gb|ABE77841.1|	PEBP [Medicago truncatula]	8E-12
	Contig124	emb|AJ250814.1|FOX250814	Fusarium oxysporum f. sp. lycopersici insertion sequence Foxy	0
	Contig318	gb|AY232720.1|	Fusarium oxysporum f. sp. vasinfectum strain X515-II Foxy transposable element, partial sequence	0
	Contig349	gb|AY232723.1|	Fusarium oxysporum f. sp. vasinfectum strain Ag149-I Foxy transposable element, partial sequence	0
	Contig636	emb|AJ250814.1|FOX250814	Fusarium oxysporum f. sp. lycopersici insertion sequence Foxy	0
	Contig123	emb|AJ608703.3|	Fusarium oxysporum f. sp. lycopersici six1 gene, fot5 gene, six2 gene, shh1 gene and ORF2 (partial)	1E-168
	Contig722	emb|AJ608703.3|	Fusarium oxysporum f. sp. lycopersici six1 gene, fot5 gene, six2 gene, shh1 gene and ORF2 (partial)	1E-117
	Contig70	emb|AJ608703.3|	Fusarium oxysporum f. sp. lycopersici six1 gene, fot5 gene, six2 gene, shh1 gene and ORF2 (partial)	1E-109
	CaF1_JIE_06_E_10	gb|AY232720.1|	Fusarium oxysporum f. sp. vasinfectum strain X515-II Foxy transposable element, partial sequence	1E-107
	Contig351	gb|AY232720.1|	Fusarium oxysporum f. sp. vasinfectum strain X515-II Foxy transposable element, partial sequence	1E-101
	CaF1_JIE_05_H_07	gb|AY232720.1|	Fusarium oxysporum f. sp. vasinfectum strain X515-II Foxy transposable element, partial sequence	3E-99
	Contig361	emb|AJ608703.3|	Fusarium oxysporum f. sp. lycopersici six1 gene, fot5 gene, six2 gene, shh1 gene and ORF2 (partial)	1E-97
	Contig73	emb|AJ608703.3|	Fusarium oxysporum f. sp. lycopersici six1 gene, fot5 gene, six2 gene, shh1 gene and ORF2 (partial)	1E-96
	Contig762	emb|AJ608703.3|	Fusarium oxysporum f. sp. lycopersici six1 gene, fot5 gene, six2 gene, shh1 gene and ORF2 (partial)	3E-91
	CaF1_JIE_01_H_08	gb|AY232724.1|	Fusarium oxysporum f. sp. vasinfectum strain Ag149-III Foxy transposable element, partial sequence	2E-87
	CaF1_WIE_12_C_02	gb|AY232722.1|	Fusarium oxysporum f. sp. vasinfectum strain Ag149 Foxy transposable element, partial sequence	4E-64
	CaF1_WIE_32_G_05	gb|AY232722.1|	Fusarium oxysporum f. sp. vasinfectum strain Ag149 Foxy transposable element, partial sequence	2E-60
	CaF1_JIE_26_E_04	emb|AJ411814.1|CAR411814	Cicer arietinum Ty3-gypsy like Retrotransposon CaRep and partial pol gene for polyprotein including RNAse and Integrase, PPT and 3'LTR, clone pCaEr915	1E-58
	CaF1_WIE_13_F_03	gb|AY232724.1|	Fusarium oxysporum f. sp. vasinfectum strain Ag149-III Foxy transposable element, partial sequence	4E-52
	Contig334	emb|CAE55867.1|	Fot5 transposase [Fusarium oxysporum f. sp. lycopersici]	2E-41
	CaF1_JIE_32_G_08	gb|AY232722.1|	Fusarium oxysporum f. sp. vasinfectum strain Ag149 Foxy transposable element, partial sequence	7E-40
	CaF1_WIE_03_H_08	emb|AJ250814.1|FOX250814	Fusarium oxysporum f. sp. lycopersici insertion sequence Foxy	9E-40
	CaF1_WIE_07_E_04	emb|AJ250814.1|FOX250814	Fusarium oxysporum f. sp. lycopersici insertion sequence Foxy	3E-31
	Contig818	gb|ABI34274.1|	IS10 transposase, putative [Lycopersicon esculentum]	1E-27
	Contig276	emb|AJ608703.3|	Fusarium oxysporum f. sp. lycopersici six1 gene, fot5 gene, six2 gene, shh1 gene and ORF2 (partial)	3E-24
	CaF1_WIE_39_B_11	ref|NP_565409.1|	phosphate-responsive 1 family protein [Arabidopsis thaliana] gb|AAD25141.1| expressed protein [Arabidopsis thaliana] gb|AAL24171.1| At2g17230/T23A1.9 [Arabidopsis thaliana] gb|AAL90964.1| At2g17230/T23A1.9 [Arabidopsis thaliana]	3E-61
	Contig419	ref|NP_850995.1|	binding [Arabidopsis thaliana] gb|AAG51417.1|AC009465_17 unknown protein; 78656-75813 [Arabidopsis thaliana] gb|AAM65399.1| contains similarity to O-linked GlcNAc transferases [Arabidopsis thaliana] gb|AAN72000.1| expressed protein [Arabidopsis thaliana]	6E-61
	CaF1_JIE_07_E_05	gb|ABE80517.1|	Autophagy-related protein 2, related [Medicago truncatula]	2E-33
	Contig487	dbj|BAB43813.1|	CaNAG2 [Candida albicans] dbj|BAB43820.1| CaNAG2 [Candida albicans]	2E-31
	Contig910	gb|ABO79239.1|	KOB1 , putative [Medicago truncatula]	5E-20
	Contig859	ref|NP_564405.1|	LOL1 (LSD ONE LIKE 1) [Arabidopsis thaliana] gb|AAL15306.1| At1g32540/T9G5_1 [Arabidopsis thaliana] gb|AAM51585.1| At1g32540/T9G5_1 [Arabidopsis thaliana] gb|AAQ55219.1| LSD1-like [Arabidopsis thaliana]	6E-20
	CaF1_WIE_35_E_08	gb|AAN77150.1|	fiber protein Fb11 [Gossypium barbadense]	2E-19

	CaF1_WIE_12_F_07	ref|NP_001050458.1|	Os03g0440900 [Oryza sativa (japonica cultivar-group)] gb|AAO23085.1| putative leucine-rich repeat protein [Oryza sativa (japonica cultivar-group)] gb|ABF96847.1| BRASSINOSTEROID INSENSITIVE 1-associated receptor kinase 1 precursor, putative, expressed [Oryza sativa (japonica cultivar-group)] dbj|BAF12372.1| Os03g0440900 [Oryza sativa (japonica cultivar-group)] gb|EAY90604.1| hypothetical protein OsI_011837 [Oryza sativa (indica cultivar-group)] gb|EAZ27458.1| hypothetical protein OsJ_010941 [Oryza sativa (japonica cultivar-group)]	7E-19
	Contig94	ref|NP_001067438.1|	Os11g0199700 [Oryza sativa (japonica cultivar-group)] gb|ABA91945.2| VHS domain containing protein, expressed [Oryza sativa (japonica cultivar-group)] dbj|BAF27801.1| Os11g0199700 [Oryza sativa (japonica cultivar-group)]	2E-17
	Contig961	gb|AAD32880.1|AC005489_18	F14N23.18 [Arabidopsis thaliana]	3E-17
	Contig167	gb|AAL06496.1|AF412043_1	AT5g04420/T32M21_20 [Arabidopsis thaliana]	4E-17
	CaF1_WIE_22_H_07	gb|ABN08340.1|	ZIM [Medicago truncatula]	3E-16
	CaF1_WIE_54_F_07	gb|AAM65872.1|	ferritin subunit, putative [Arabidopsis thaliana]	3E-15
	CaF1_JIE_35_B_03	gb|ABL59986.1|	brittle stalk-2-like protein 6 [Zea mays]	6E-15
	CaF1_JIE_36_B_02	gb|AAW33880.1|	RING-H2 subgroup RHE protein [Populus alba x Populus tremula]	8E-15
	Contig490	gb|AAT66941.1|	CesA2 [Acacia mangium]	1E-14
	Contig902	ref|XP_001257448.1|	cupin domain protein [Neosartorya fischeri NRRL 181] gb|EAW15551.1| cupin domain protein [Neosartorya fischeri NRRL 181]	1E-14
	CaF1_WIE_03_F_11	dbj|BAB43814.1|	CaNAG3 [Candida albicans] dbj|BAB43819.1| CaNAG3 [Candida albicans]	3E-14
	CaF1_WIE_40_C_10	ref|NP_001049320.1|	Os03g0206600 [Oryza sativa (japonica cultivar-group)] gb|ABF94553.1| integral membrane protein, putative, expressed [Oryza sativa (japonica cultivar-group)] dbj|BAF11234.1| Os03g0206600 [Oryza sativa (japonica cultivar-group)]	5E-14
	CaF1_WIE_27_A_06	gb|AAN38066.1|	oligomycin sensitivity conferring protein [Silene latifolia]	9E-14
	Contig417	gb|ABE79354.1|	Embryo-specific 3 [Medicago truncatula]	2E-13
	CaF1_WIE_45_B_03	gb|ABE84233.1|	Arf GTPase activating protein [Medicago truncatula]	2E-13
	Contig426	dbj|BAB16429.1|	NtEIG-A1 [Nicotiana tabacum]	4E-13
	Contig399	gb|ABE82627.1|	Argonaute and Dicer protein, PAZ; Stem cell self-renewal protein Piwi [Medicago truncatula] gb|ABE93839.1| Argonaute and Dicer protein, PAZ; Stem cell self-renewal protein Piwi [Medicago truncatula]	8E-12
	CaF1_WIE_41_D_01	gb|ABF06706.1|	UP-9A [Nicotiana tabacum]	1E-11
	Contig67	gb|ABN08080.1|	ATA15 protein, putative [Medicago truncatula]	2E-11
	Contig144	emb|CAA93759.1|	putative transposase [Tolypocladium inflatum]	6E-11
	Contig225	gb|ABE87946.1|	CAP protein [Medicago truncatula]	9E-11
	CaF1_JIE_14_C_09	gb|ABO84327.1|	Putative non-LTR retroelement reverse transcriptase, related [Medicago truncatula]	4E-19
No significant homology	CaF1_JIE_26_F_03	gb|AF271892.1|AF271892	Pisum sativum DEAD box protein P68 (P68) mRNA, complete cds	2E-20
	CaF1_WIE_47_A_07	emb|CU137664.2|	Medicago truncatula chromosome 5 clone mth2-69n5, COMPLETE SEQUENCE	2E-20
	Contig401	gb|AF426840.1|	Trifolium repens early nodulin enod40-3 mRNA, complete cds	6E-20
	CaF1_WIE_07_F_04	gb|AC144477.16|	Medicago truncatula clone mth2-6k4, complete sequence	6E-20
	CaF1_WIE_47_B_07	gb|AC152818.17|	Medicago truncatula clone mth2-85g12, complete sequence	6E-20
	CaF1_JIE_09_A_03	gb|AC125476.30|	Medicago truncatula clone mth2-10e13, complete sequence	8E-20
	Contig314	emb|CU302335.1|	Medicago truncatula chromosome 5 clone mth2-155g22, COMPLETE SEQUENCE	1E-19
	Contig774	gb|AC121245.17|	Medicago truncatula clone mth2-33g3, complete sequence	1E-19
	CaF1_WIE_37_E_11	emb|CU062643.7|	M.truncatula DNA sequence from clone MTH2-144G2 on chromosome 3, complete sequence	4E-19
	CaF1_WIE_47_E_01	emb|AJ608703.3|	Fusarium oxysporum f. sp. lycopersici six1 gene, fot5 gene, six2 gene, shh1 gene and ORF2 (partial)	5E-19
	CaF1_WIE_20_H_06	gb|DQ455594.1|	Phaseolus vulgaris PP2c mRNA, partial cds	6E-19
	CaF1_JIE_25_H_03	emb|CU424495.1|	Medicago truncatula chromosome 5 clone mte1-17l21, COMPLETE SEQUENCE	1E-18
	Contig404	gb|AC182813.8|	Medicago truncatula clone mth2-69k1, complete sequence	2E-18
	CaF1_WIE_29_E_10	gb|AC150981.14|	Medicago truncatula clone mth2-166m22, complete sequence	2E-18
	CaF1_WIE_33_B_02	gb|AC146705.11|	Medicago truncatula clone mth2-101f3, complete sequence	2E-18
	CaF1_WIE_34_D_02	ref|XM_387051.1|	Gibberella zeae PH-1 chromosome 4 hypothetical protein (FG06875.1) partial mRNA	5E-18
	Contig306	gb|AF075691.1|	Crassostrea gigas BAT1 homolog mRNA, complete cds	8E-18
	Contig369	gb|AC174370.14|	Medicago truncatula chromosome 8 clone mth2-50f20, complete sequence	8E-18
	Contig385	gb|BT009458.1|	Triticum aestivum clone wlsu2.pk0001.h3:fis, full insert mRNA sequence	2E-17

	CaF1_WIE_14_C_11	dbj|AP004547.1|	Lotus japonicus genomic DNA, chromosome 6, clone:LjT31L24, TM0228a, complete sequence	2E-17
	CaF1_JIE_19_G_10	emb|AM706411.1|	Eristalis tenax partial mRNA for hypothetical protein (ORF1), isolate 3	3E-17
	CaF1_WIE_48_H_01	emb|AJ249801.1|CAR249801	Cicer arietinum partial mRNA for cytochrome P450 monooxygenase (cyp81E4 gene)	3E-17
	CaF1_WIE_46_F_04	gb|AC135231.23|	Medicago truncatula clone mth2-28m10, complete sequence	5E-17
	CaF1_JIE_37_E_05	gb|AC147009.5|	Medicago truncatula clone mth2-139j3, complete sequence	6E-17
	Contig479	gb|AC169178.2|	Medicago truncatula chromosome 7 BAC clone mth2-2o21, complete sequence	1E-16
	Contig737	gb|AC149135.2|	Medicago truncatula chromosome 2 clone mth2-31g23, complete sequence	1E-16
	CaF1_WIE_04_C_07	emb|AJ749800.1|	Photobacterium damselae subsp. piscicida partial coi genes for putative cytochrome C oxidase proteins, clone pRDA19	1E-16
	CaF1_WIE_55_C_06	emb|CU019601.8|	M.truncatula DNA sequence from clone MTH2-12A22 on chromosome 3, complete sequence	1E-16
	Contig467	gb|AC121233.16|	Medicago truncatula clone mth2-14g13, complete sequence	2E-16
	CaF1_WIE_24_G_09	gb|AC187645.7|	Glycine max clone gmp1-49d18, complete sequence	2E-16
	CaF1_JIE_05_F_10	emb|X56240.1|VFUSP	V.faba USP gene for an unknown seed protein	3E-16
	CaF1_WIE_36_B_06	dbj|AP004940.1|	Lotus japonicus genomic DNA, chromosome 3, clone:LjT48I11, TM0106, complete sequence	3E-16
	CaF1_JIE_07_F_03	gb|AC146549.8|	Medicago truncatula clone mth2-7p18, complete sequence	5E-16
	CaF1_WIE_24_B_08	emb|AM486183.1|	Vitis vinifera, whole genome shotgun sequence, contig VV78X166878.4, clone ENTAV 115	5E-16
	Contig583	gb|AC137701.25|	Medicago truncatula clone mth2-35h1, complete sequence	6E-16
	Contig809	gb|AC139708.15|	Medicago truncatula clone mth2-9f16, complete sequence	6E-16
	Contig444	emb|AM158278.1|	Phaseolus vulgaris partial mRNA for proline-rich protein (prp3 gene), variety the Prince	8E-16
	CaF1_WIE_23_D_03	gb|AC148237.7|	Medicago truncatula clone mth2-1o14, complete sequence	1E-15
	Contig237	ref|NW_001594396.1|	Aspergillus niger CBS 513.88 contig An17c0060, complete genome emb|AM270388.1| Aspergillus niger contig An17c0060, complete genome	2E-15
	Contig944	gb|DQ459385.1|	Nicotiana tabacum serine/threonine kinase mRNA, partial cds	2E-15
	CaF1_WIE_36_A_08	gb|AC152818.17|	Medicago truncatula clone mth2-85g12, complete sequence	2E-15
	Contig329	gb|AC140546.12|	Medicago truncatula clone mth2-35l19, complete sequence	3E-15
	Contig593	gb|AC137703.42|	Medicago truncatula clone mth2-11d24, complete sequence	3E-15
	CaF1_WIE_36_G_06	gb|AC124963.32|	Medicago truncatula clone mth2-24f5, complete sequence	7E-15
	CaF1_WIE_31_H_11	ref|XM_384325.1|	Gibberella zeae PH-1 chromosome 2 hypothetical protein (FG04149.1) partial mRNA	8E-15
	Contig681	gb|AF075691.1|	gigas BAT1 homolog mRNA, complete cds	3E-08
	CaF1_WIE_09_D_10	gb|AY245442.1|	Pisum sativum ent-kaurene oxidase (LH) mRNA, complete cds	3E-14
	CaF1_WIE_20_F_04	ref|XM_955692.1|	Neurospora crassa OR74A hypothetical protein (NCU08963.1) partial mRNA	3E-14
	CaF1_WIE_12_C_04	emb|CT963109.2|	Medicago truncatula chromosome 5 clone mth2-64i23, COMPLETE SEQUENCE	4E-14
	Contig460	gb|AF075691.1|	Crassostrea gigas BAT1 homolog mRNA, complete cds	9E-14
	CaF1_JIE_05_D_06	gb|DQ459385.1|	Nicotiana tabacum serine/threonine kinase mRNA, partial cds	1E-13
	CaF1_JIE_40_C_08	emb|AM706411.1|	Eristalis tenax partial mRNA for hypothetical protein (ORF1), isolate 3	1E-13
	CaF1_JIE_22_D_11	ref|XM_381680.1|	Gibberella zeae PH-1 chromosome 1 conserved hypothetical protein (FG01504.1) partial mRNA	5E-13
	CaF1_WIE_36_D_10	gb|AC174299.9|	Medicago truncatula clone mth2-80e19, complete sequence	5E-13
	Contig523	ref|XM_383891.1|	Gibberella zeae PH-1 chromosome 2 hypothetical protein (FG03715.1) partial mRNA	6E-13
	Contig614	gb|AC140104.22|	Medicago truncatula clone mth2-10g22, complete sequence	6E-13
	Contig666	emb|AM473965.2|	Vitis vinifera contig VV78X014869.2, whole genome shotgun sequence	9E-13
	Contig132	gb|AC136840.24|	Medicago truncatula clone mth2-33n3, complete sequence	1E-12
	CaF1_JIE_19_E_08	ref|XM_381728.1|	Gibberella zeae PH-1 chromosome 1 hypothetical protein (FG01552.1) partial mRNA	2E-12
	CaF1_WIE_08_E_09	gb|AC173474.18|	Medicago truncatula clone mth2-64j6, complete sequence	2E-12
	CaF1_WIE_29_H_10	emb|CT573421.5|	M.truncatula DNA sequence from clone MTH2-27B10 on chromosome 3, complete sequence	2E-12
	Contig277	gb|AC145222.18|	Medicago truncatula clone mth2-29a15, complete sequence	3E-12
	CaF1_WIE_11_E_06	gb|AC146819.19|	Medicago truncatula clone mth2-176k6, complete sequence	5E-12
	CaF1_WIE_04_F_04	gb|AC140026.11|	Medicago truncatula clone mth2-36j24, complete sequence	7E-12
	Contig257	gb|BT009458.1|	Triticum aestivum clone wlsu2.pk0001.h3:fis, full insert mRNA sequence	9E-12
	Contig658	gb|AC136472.40|	Medicago truncatula clone mth2-24f21, complete sequence	1E-11
	CaF1_JIE_37_G_05	ref|XM_381579.1|	Gibberella zeae PH-1 chromosome 1 hypothetical protein (FG01403.1) partial mRNA	1E-11
	CaF1_WIE_49_H_04	emb|AM425323.1|	Vitis vinifera contig VV78X242376.24, whole genome shotgun sequence	1E-11

	Contig456	emb|AM478166.2|	Vitis vinifera contig VV78X136477.5, whole genome shotgun sequence	2E-11
	Contig455	dbj|D14411.1|VIRARG2	Vigna radiata arg2 mRNA	3E-11
	CaF1_WIE_21_E_04	gb|AC144731.15|	Medicago truncatula clone mth2-5g18, complete sequence	3E-11
	CaF1_WIE_34_A_03	emb|AJ299396.1|CAR299396	Cicer arietinum partial mRNA for putative extensin (ORF), clone CanEXT-1	4E-11
	CaF1_JIE_29_A_01	gb|U10046.1|PSU10046	Pisum sativum ribosomal protein L27 homolog (RPL27-5) mRNA, complete cds	5E-11
	Contig838	gb|AY099112.1|	Rattus norvegicus obese protein gene, 5' flanking region and partial cds	6E-11
	CaF1_WIE_38_A_05	gb|AY099112.1|	Rattus norvegicus obese protein gene, 5' flanking region and partial cds	6E-11
	CaF1_WIE_38_C_05	gb|AY099112.1|	Rattus norvegicus obese protein gene, 5' flanking region and partial cds	6E-11
	CaF1_WIE_38_H_04	gb|AY099112.1|	Rattus norvegicus obese protein gene, 5' flanking region and partial cds	6E-11
	Contig464	emb|CR962134.2|	Medicago truncatula chromosome 5 clone mte1-40n1, COMPLETE SEQUENCE	8E-11
	Contig695	emb|CR954195.3|	Medicago truncatula chromosome 5 clone mth2-16p13, COMPLETE SEQUENCE	9E-11
	CaF1_WIE_55_E_10	gb|DQ073809.1|	Trifolium pratense phenylalanine ammonia lyase (PAL1) mRNA, complete cds	9E-11
	Contig7	dbj|AP006137.1|	Lotus japonicus genomic DNA, chromosome 5, clone:LjT37K17, TM0239, complete sequence	1E-10
	Contig423	emb|Y15372.1|MTY15372	Medicago truncatula mRNA for MtN4 gene, partial	1E-10
	CaF1_WIE_49_G_02	ref|XM_382053.1|	Gibberella zeae PH-1 chromosome 1 hypothetical protein (FG01877.1) partial mRNA	1E-10
	CaF1_WIE_17_B_03	emb|CT963114.2|	Medicago truncatula chromosome 5 clone mte1-26c9, COMPLETE SEQUENCE	5E-10
	Contig149	emb|CR954193.3|	Medicago truncatula chromosome 5 clone mth2-27a1, COMPLETE SEQUENCE	8E-10
	CaF1_JIE_20_B_11	gb|AC148406.12|	Medicago truncatula clone mth2-46e9, complete sequence	8E-10
	Contig438	ref|NM_105819.3|	Arabidopsis thaliana Rieske (2Fe-2S) domain-containing protein (AT1G71500) mRNA, complete cds	2E-09
	CaF1_JIE_05_E_02	emb|AJ538370.1|NTA538370	Nicotiana tabacum cDNA-AFLP-fragment BT1-M24-018	2E-09
	CaF1_WIE_46_D_09	dbj|AP006094.1|	Lotus japonicus genomic DNA, chromosome 4, clone:LjT39H01, TM0172, complete sequence	3E-09
	CaF1_WIE_46_A_10	emb|AM452705.1|	Vitis vinifera, whole genome shotgun sequence, contig VV78X225485.15, clone ENTAV 115	4E-09
	CaF1_WIE_03_F_02	gb|DQ459385.1|	Nicotiana tabacum serine/threonine kinase mRNA, partial cds	5E-09
	CaF1_WIE_55_E_04	ref|XM_415370.2|	PREDICTED: Gallus gallus similar to class I alpha chain (LOC417083), mRNA	8E-09
	CaF1_JIE_20_A_01	gb|AF082024.1|AF082024	Pimpinella brachycarpa Phyb1 mRNA, complete cds	9E-09
	CaF1_WIE_48_G_04	gb|AC174142.11|	Medicago truncatula clone mth2-69j4, complete sequence	9E-09
	CaF1_JIE_12_C_01	emb|AM706411.1|	Eristalis tenax partial mRNA for hypothetical protein (ORF1), isolate 3	1E-08
	CaF1_JIE_25_D_02	emb|AM706411.1|	Eristalis tenax partial mRNA for hypothetical protein (ORF1), isolate 3	1E-08
	CaF1_JIE_35_H_02	emb|AM706411.1|	Eristalis tenax partial mRNA for hypothetical protein (ORF1), isolate 3	1E-08
	CaF1_WIE_40_G_02	gb|AF075691.1|	Crassostrea gigas BAT1 homolog mRNA, complete cds	1E-08
	CaF1_WIE_49_B_11	dbj|AP006667.1|	Lotus japonicus genomic DNA, chromosome 5, clone:LjT31L14, TM0366, complete sequence	2E-08
	CaF1_JIE_19_A_09	emb|AM706411.1|	Eristalis tenax partial mRNA for hypothetical protein (ORF1), isolate 3	3E-08
	CaF1_WIE_26_H_02	emb|CT573077.3|	Medicago truncatula chromosome 5 clone mth2-43j18, COMPLETE SEQUENCE	3E-08
	CaF1_WIE_48_C_05	emb|CR931811.1|	Medicago truncatula chromosome 5 clone mte1-56a16, COMPLETE SEQUENCE	3E-08
	CaF1_WIE_33_F_09	emb|Z23097.1|PSMETALLP	P.sativum gene for metallothionein-like protein	5E-08
	CaF1_JIE_18_E_07	emb|AJ749800.1|	Photobacterium damselae subsp. piscicida partial coi genes for putative cytochrome C oxidase proteins, clone pRDA19	6E-08
	CaF1_WIE_32_E_11	gb|AF075691.1|	Crassostrea gigas BAT1 homolog mRNA, complete cds	8E-08
	CaF1_JIE_06_D_08	gb|AC152184.1|	Medicago truncatula chromosome 7 clone mte1-61c3, complete sequence	9E-08
	Contig469	emb|AM450436.2|	Vitis vinifera contig VV78X174412.6, whole genome shotgun sequence	0.0000001
	CaF1_JIE_26_B_04	emb|AJ250814.1|FOX250814	Fusarium oxysporum f. sp. lycopersici insertion sequence Foxy	0.0000001
	CaF1_JIE_38_E_11	gb|AF075691.1|	Crassostrea gigas BAT1 homolog mRNA, complete cds	0.0000001
	CaF1_WIE_24_F_02	emb|AM466081.2|	Vitis vinifera contig VV78X185514.6, whole genome shotgun sequence	0.0000001
	CaF1_WIE_27_F_02	emb|CT573504.2|	Medicago truncatula chromosome 5 clone mth2-178o19, COMPLETE SEQUENCE	0.0000001

	Contig570	ref|NM_113389.1|	Arabidopsis thaliana CLE41 (CLAVATA3/ESR-RELATED 41); receptor binding (CLE41) mRNA, complete cds	0.0000002
	Contig919	gb|AC149636.14|	Medicago truncatula clone mth2-180a12, complete sequence	0.0000002
	CaF1_WIE_01_B_07	gb|DQ459385.1|	Nicotiana tabacum serine/threonine kinase mRNA, partial cds	0.0000002
	CaF1_WIE_35_F_11	dbj|AP004971.1|	Lotus japonicus genomic DNA, chromosome 5, clone:LjT45G21, TM0151, complete sequence	0.0000002
	CaF1_JIE_34_B_03	emb|AJ749800.1|	Photobacterium damselae subsp. piscicida partial coi genes for putative cytochrome C oxidase proteins, clone pRDA19	0.0000003
	CaF1_JIE_41_A_10	emb|AJ749800.1|	Photobacterium damselae subsp. piscicida partial coi genes for putative cytochrome C oxidase proteins, clone pRDA19	0.0000003
	CaF1_WIE_45_B_10	emb|CT573508.2|	Medicago truncatula chromosome 5 clone mth2-29p15, COMPLETE SEQUENCE	0.0000003
	CaF1_JIE_17_E_07	emb|AJ749800.1|	Photobacterium damselae subsp. piscicida partial coi genes for putative cytochrome C oxidase proteins, clone pRDA19	0.0000005
	CaF1_WIE_07_E_10	emb|Z31720.1|NTL19RIB	N.tabacum (cv.Samsun NN) L19 mRNA for ribosomal protein L19	0.0000005
	CaF1_WIE_10_F_11	gb|AC126784.11|	Medicago truncatula clone mth2-36b12, complete sequence	0.0000005
	CaF1_WIE_32_E_09	gb|L34658.1|FSOGAGPOL	Fusarium oxysporum gag polyprotein (gag) gene, complete cds; pol polyprotein (pol) gene, complete cds	0.0000005
	CaF1_WIE_44_C_11	dbj|AB182973.1|	Rana pirica mRNA for trypsinogen, partial sequence, clone:No 316	0.0000005
	CaF1_WIE_48_E_02	dbj|AK224696.2|	Solanum lycopersicum cDNA, clone: FC08DG08, HTC in fruit	0.0000005
	CaF1_WIE_37_E_04	emb|AM748410.1|	Vigna unguiculata partial mRNA for putative CBL-interacting protein kinase 12 (CIPK12 gene), clone 24	0.0000008
	CaF1_WIE_39_H_08	emb|AM748410.1|	Vigna unguiculata partial mRNA for putative CBL-interacting protein kinase 12 (CIPK12 gene), clone 24	0.0000008
	Contig699	gb|DQ251457.1|	Siniperca chuatsi transposase mRNA, partial cds	0.000001
	Contig700	gb|DQ459385.1|	Nicotiana tabacum serine/threonine kinase mRNA, partial cds	0.000001
	CaF1_JIE_25_F_03	gb|DQ459385.1|	Nicotiana tabacum serine/threonine kinase mRNA, partial cds	0.000001
	CaF1_JIE_40_G_04	gb|AC186194.28|	Medicago truncatula chromosome 6 clone mth2-77b24, complete sequence	0.000001
	CaF1_WIE_01_H_08	emb|CT028787.1|	Poplar cDNA sequences	0.000001
	CaF1_WIE_27_E_02	gb|DQ251457.1|	Siniperca chuatsi transposase mRNA, partial cds	0.000001
	CaF1_WIE_55_C_01	gb|AF520576.1|	Glycine max extensin-like protein gene, promoter region and complete cds	0.000001
	Contig447	gb|DQ251457.1|	Siniperca chuatsi transposase mRNA, partial cds	0.000002
	Contig476	emb|CT028615.1|	Poplar cDNA sequences	0.000002
	Contig898	gb|DQ507301.1|	Belgica antarctica clone Ba-U40 CG32816-like mRNA, partial cds	0.000002
	Contig924	gb|AC150244.2|	Medicago truncatula chromosome 7 clone mth2-180p14, complete sequence	0.000002
	Contig801	emb|CU013517.1|	Medicago truncatula chromosome 5 clone mth2-76o9, COMPLETE SEQUENCE	0.000003
	Contig96	emb|AJ749794.1|	Photobacterium damselae subsp. piscicida trpA gene for putative transposase and partial ORF1 DNA for hypothetical protein, clone pRDA13	0.000004
	Contig504	gb|DQ251457.1|	Siniperca chuatsi transposase mRNA, partial cds	0.000004
	CaF1_JIE_29_D_09	emb|AJ749803.1|	Photobacterium damselae subsp. piscicida partial ORF1 DNA for hypothetical protein, clone pRDA24	0.000004
	CaF1_WIE_32_E_07	emb|CT028787.1|	Poplar cDNA sequences	0.000004
	CaF1_WIE_36_D_07	emb|AJ534351.1|ABI534351	Agaricus bisporus partial mRNA for putative myosin heavy chain kinase (mhck gene), clone pm31	0.000004
	CaF1_WIE_36_E_03	gb|DQ465789.1|	Sesbania drummondii clone SSH-36_01_A09_T3 mRNA sequence	0.000004
	CaF1_WIE_12_C_11	emb|AJ279846.1|MMU279846	Mus musculus partial mRNA for hypothetical protein, clone mvx2002	0.000005
	Contig122	gb|DQ406807.1|	Ceratitis capitata clone 17a mRNA sequence	0.000006
	Contig812	gb|DQ251457.1|	Siniperca chuatsi transposase mRNA, partial cds	0.000006
	CaF1_WIE_31_E_05	emb|CU302248.6|	M.truncatula DNA sequence from clone MTH2-33P23 on chromosome 3, complete sequence	0.000006
	Contig238	gb|AC166543.2|	Nectria haematococca clone JGIAYWI-5I13, complete sequence	0.000007
	Contig841	gb|DQ251457.1|	Siniperca chuatsi transposase mRNA, partial cds	0.000008
	CaF1_WIE_13_E_08	emb|AM422097.2|	Danio rerio sox3 mRNA, 3'UTR	0.000008
	CaF1_WIE_54_D_10	emb|AM697674.1|	Platynereis dumerilii mRNA for hypothetical protein (ORF1), isolate 2	0.000008
	CaF1_WIE_55_H_06	gb|AY273895.1|	Brugia malayi transglutaminase mRNA, partial cds	0.000008
	Contig251	gb|AF537102.1|	Plasmodiophora brassicae 16S ribosomal RNA gene, partial sequence; mitochondrial gene for mitochondrial product	0.000009
	Contig642	gb|AF537102.1|	Plasmodiophora brassicae 16S ribosomal RNA gene, partial sequence; mitochondrial gene for mitochondrial product	0.00001
	CaF1_JIE_07_D_04	gb|AY461597.1|	Synthetic construct arsenic-like protein gene, complete cds	0.00001
	CaF1_WIE_05_G_07	emb|AJ749797.1|	Photobacterium damselae subsp. piscicida trpB gene for putative transposase, clone pRDA16	0.00001
	CaF1_WIE_47_E_02	gb|DQ465754.1|	Sesbania drummondii clone SSH-1_01_F12_T3 mRNA sequence	0.00001
	CaF1_WIE_48_D_09	gb|DQ251457.1|	Siniperca chuatsi transposase mRNA, partial cds	0.00001
	CaF1_WIE_54_E_09	emb|AM422122.1|	Danio rerio tcamp mRNA, 3' UTR	0.00001

	Contig262	emb|AM706411.1|	Eristalis tenax partial mRNA for hypothetical protein (ORF1), isolate 3	0.00002
	Contig354	gb|DQ251457.1|	Siniperca chuatsi transposase mRNA, partial cds	0.00002
	Contig498	dbj|AB182104.1|	Silene latifolia SlSS mRNA for strictosidine synthase family protein, partial cds	0.00002
	Contig661	gb|DQ251457.1|	Siniperca chuatsi transposase mRNA, partial cds	0.00002
	Contig872	emb|AM422095.1|	Danio rerio partial mRNA for vox protein (vox gene)	0.00002
	CaF1_JIE_01_A_03	emb|AM497806.1|	Nidula niveotomentosa partial mRNA for putative betatubulin (btt gene)	0.00002
	CaF1_JIE_29_A_08	gb|AY887902.1|	Homo sapiens mutant GSTP1 (GSTP1) mRNA, complete cds	0.00002
	CaF1_JIE_29_G_02	gb|AF537102.1|	Plasmodiophora brassicae 16S ribosomal RNA gene, partial sequence; mitochondrial gene for mitochondrial product	0.00002
	CaF1_JIE_34_G_01	gb|DQ251457.1|	Siniperca chuatsi transposase mRNA, partial cds	0.00002
	CaF1_JIE_36_A_06	emb|AJ749794.1|	Photobacterium damselae subsp. piscicida trpA gene for putative transposase and partial ORF1 DNA for hypothetical protein, clone pRDA13	0.00002
	CaF1_JIE_37_H_06	gb|AF537102.1|	Plasmodiophora brassicae 16S ribosomal RNA gene, partial sequence; mitochondrial gene for mitochondrial product	0.00002
	CaF1_JIE_41_C_07	gb|AF537102.1|	Plasmodiophora brassicae 16S ribosomal RNA gene, partial sequence; mitochondrial gene for mitochondrial product	0.00002
	CaF1_WIE_07_G_04	gb|DQ251457.1|	Siniperca chuatsi transposase mRNA, partial cds	0.00002
	CaF1_WIE_12_H_10	emb|AJ749794.1|	Photobacterium damselae subsp. piscicida trpA gene for putative transposase and partial ORF1 DNA for hypothetical protein, clone pRDA13	0.00002
	CaF1_WIE_48_D_03	emb|CT028615.1|	Poplar cDNA sequences	0.00002
	CaF1_WIE_48_E_03	gb|DQ251457.1|	Siniperca chuatsi transposase mRNA, partial cds	0.00002
	Contig15	gb|AF537102.1|	Plasmodiophora brassicae 16S ribosomal RNA gene, partial sequence; mitochondrial gene for mitochondrial product	0.00003
	Contig29	gb|DQ251457.1|	Siniperca chuatsi transposase mRNA, partial cds	0.00003
	Contig75	gb|DQ251457.1|	Siniperca chuatsi transposase mRNA, partial cds	0.00003
	Contig121	emb|AM748509.1|	Vigna unguiculata partial mRNA for putative single-stranded nucleic acid binding R3H (MtrDRAFT_AC183371g11v1 gene), clone 54	0.00003
	Contig172	gb|DQ251457.1|	Siniperca chuatsi transposase mRNA, partial cds	0.00003
	Contig541	gb|AC134242.17|	Medicago truncatula clone mth2-10p20, complete sequence	0.00003
	Contig684	gb|AF537102.1|	Plasmodiophora brassicae 16S ribosomal RNA gene, partial sequence; mitochondrial gene for mitochondrial product	0.00003
	CaF1_JIE_22_B_08	gb|DQ251457.1|	Siniperca chuatsi transposase mRNA, partial cds	0.00003
	CaF1_JIE_40_H_02	emb|AM706411.1|	Eristalis tenax partial mRNA for hypothetical protein (ORF1), isolate 3	0.00003
	CaF1_WIE_43_F_02	emb|CT028787.1|	Poplar cDNA sequences	0.00003
	CaF1_WIE_46_C_07	gb|AF537102.1|	Plasmodiophora brassicae 16S ribosomal RNA gene, partial sequence; mitochondrial gene for mitochondrial product	0.00003
	Contig394	emb|AJ749801.1|	Photobacterium damselae subsp. piscicida partial trpA gene for putative transposase, clone pRDA20	0.00004
	CaF1_WIE_24_F_04	emb|AM706411.1|	Eristalis tenax partial mRNA for hypothetical protein (ORF1), isolate 3	0.00004
	CaF1_WIE_46_E_03	emb|AM706411.1|	Eristalis tenax partial mRNA for hypothetical protein (ORF1), isolate 3	0.00004
	CaF1_WIE_47_D_08	emb|AM706411.1|	Eristalis tenax partial mRNA for hypothetical protein (ORF1), isolate 3	0.00004
	CaF1_WIE_49_G_08	emb|AJ276466.1|KPN276466	Klebsiella pneumoniae contig region pSL004	0.00004
	Contig384	emb|AM497810.1|	Nidula niveotomentosa partial mRNA for glucose-6-phosphate-1-dehydrogenase (g6pd gene)	0.00005
	Contig414	emb|AM706411.1|	Eristalis tenax partial mRNA for hypothetical protein (ORF1), isolate 3	0.00005
	Contig835	gb|AC174308.7|	Medicago truncatula clone mth2-139i23, complete sequence	0.00006
	CaF1_WIE_30_E_07	gb|BT009458.1|	Triticum aestivum clone wlsu2.pk0001.h3:fis, full insert mRNA sequence	0.00006
	CaF1_WIE_32_H_09	emb|AJ749803.1|	Photobacterium damselae subsp. piscicida partial ORF1 DNA for hypothetical protein, clone pRDA24	0.00006
	CaF1_WIE_49_A_02	emb|AJ293849.1|KPN293849	Klebsiella pneumoniae contig region pSL029	0.00006
	CaF1_WIE_51_A_10	gb|BT009458.1|	Triticum aestivum clone wlsu2.pk0001.h3:fis, full insert mRNA sequence	0.00006
	Contig620	gb|AY972077.1|	Synthetic construct RLS (RLS) gene, complete cds	0.00007
	CaF1_JIE_10_C_01	emb|AM706411.1|	Eristalis tenax partial mRNA for hypothetical protein (ORF1), isolate 3	0.00007
	CaF1_WIE_05_B_10	emb|AJ749800.1|	Photobacterium damselae subsp. piscicida partial coi genes for putative cytochrome C oxidase proteins, clone pRDA19	0.00007
	CaF1_WIE_16_C_09	emb|AJ749800.1|	Photobacterium damselae subsp. piscicida partial coi genes for putative cytochrome C oxidase proteins, clone pRDA19	0.00007
	Contig701	ref|XM_390948.1|	Gibberella zeae PH-1 chromosome 3 hypothetical protein (FG10772.1) partial mRNA	0.00008

	CaF1_WIE_35_E_06	emb|AM706411.1|	Eristalis tenax partial mRNA for hypothetical protein (ORF1), isolate 3	0.00008
	CaF1_WIE_40_G_08	gb|DQ459385.1|	Nicotiana tabacum serine/threonine kinase mRNA, partial cds	0.00008
	Contig514	emb|CT028662.1|	Poplar cDNA sequences	0.00009
	Contig808	emb|AJ749797.1|	Photobacterium damselae subsp. piscicida trpB gene for putative transposase, clone pRDA16	0.00009
	CaF1_JIE_30_H_05	emb|AM497808.1|	Nidula niveotomentosa partial mRNA for putative thiolase c (thio c gene)	0.00009
	CaF1_JIE_32_E_10	emb|AJ293848.1|KPN293848	Klebsiella pneumoniae contig region pSL022	0.00009
	Contig35	emb|AM706411.1|	Eristalis tenax partial mRNA for hypothetical protein (ORF1), isolate 3	0.0001
	Contig44	emb|AJ749797.1|	Photobacterium damselae subsp. piscicida trpB gene for putative transposase, clone pRDA16	0.0001
	Contig101	emb|CR954185.3|	Medicago truncatula chromosome 5 clone mth4-20m5, COMPLETE SEQUENCE	0.0001
	Contig146	gb|AY972077.1|	Synthetic construct RLS (RLS) gene, complete cds	0.0001
	Contig211	emb|AM706411.1|	Eristalis tenax partial mRNA for hypothetical protein (ORF1), isolate 3	0.0001
	Contig418	emb|AJ749794.1|	Photobacterium damselae subsp. piscicida trpA gene for putative transposase and partial ORF1 DNA for hypothetical protein, clone pRDA13	0.0001
	Contig448	emb|AM706411.1|	Eristalis tenax partial mRNA for hypothetical protein (ORF1), isolate 3	0.0001
	Contig471	gb|AY972077.1|	Synthetic construct RLS (RLS) gene, complete cds	0.0001
	Contig512	emb|AM484893.2|	Vitis vinifera contig VV78X135458.6, whole genome shotgun sequence	0.0001
	Contig519	gb|BT009458.1|	Triticum aestivum clone wlsu2.pk0001.h3:fis, full insert mRNA sequence	0.0001
	Contig560	emb|AM706411.1|	Eristalis tenax partial mRNA for hypothetical protein (ORF1), isolate 3	0.0001
	Contig621	gb|AY972077.1|	Synthetic construct RLS (RLS) gene, complete cds	0.0001
	Contig761	gb|DQ990796.1|	Hetreulophus sp. CD021 enolase mRNA, partial cds	0.0001
	Contig888	emb|AM422119.2|	Danio rerio ca7 mRNA, 3' UTR	0.0001
	Contig950	emb|AM706411.1|	Eristalis tenax partial mRNA for hypothetical protein (ORF1), isolate 3	0.0001
	Contig446	gb|AC174296.26|	Medicago truncatula clone mth2-72l21, complete sequence	0.0005
	CaF1_JIE_22_D_10	emb|AM706411.1|	Eristalis tenax partial mRNA for hypothetical protein (ORF1), isolate 3	0.0001
	CaF1_JIE_32_G_09	emb|AJ749800.1|	Photobacterium damselae subsp. piscicida partial coi genes for putative cytochrome C oxidase proteins, clone pRDA19	0.0001
	CaF1_WIE_06_H_09	ref|XM_001068112.1|	PREDICTED: Rattus norvegicus hypothetical protein LOC688741 (LOC688741), mRNA	0.0001
	CaF1_WIE_08_H_08	emb|AJ293850.1|KPN293850	Klebsiella pneumoniae partial EVGA gene for putative positive transcription regulator EVGA, contig region pSL042	0.0001
	CaF1_WIE_17_A_10	gb|AY972077.1|	Synthetic construct RLS (RLS) gene, complete cds	0.0001
	CaF1_WIE_29_F_08	dbj|AB182103.1|	Silene latifolia SlAPG mRNA for anther-specific proline-rich protein APG precursor, partial cds	0.0001
	CaF1_WIE_33_C_08	dbj|AB182972.1|	Rana pirica mRNA for trypsinogen, partial sequence, clone:No 304	0.0001
	CaF1_WIE_37_C_10	emb|AM706411.1|	Eristalis tenax partial mRNA for hypothetical protein (ORF1), isolate 3	0.0001
	CaF1_WIE_46_G_10	emb|AM706411.1|	Eristalis tenax partial mRNA for hypothetical protein (ORF1), isolate 3	0.0001
	CaF1_WIE_49_D_09	gb|DQ465754.1|	Sesbania drummondii clone SSH-1_01_F12_T3 mRNA sequence	0.0001
	CaF1_WIE_50_D_06	emb|AM706411.1|	Eristalis tenax partial mRNA for hypothetical protein (ORF1), isolate 3	0.0001
	CaF1_WIE_51_G_06	gb|AY461597.1|	Synthetic construct arsenic-like protein gene, complete cds	0.0001
	CaF1_WIE_54_E_06	gb|AY972077.1|	Synthetic construct RLS (RLS) gene, complete cds	0.0001
	Contig137	gb|BT009458.1|	Triticum aestivum clone wlsu2.pk0001.h3:fis, full insert mRNA sequence	0.0002
	Contig309	gb|BT009458.1|	Triticum aestivum clone wlsu2.pk0001.h3:fis, full insert mRNA sequence	0.0002
	Contig402	emb|AM706411.1|	Eristalis tenax partial mRNA for hypothetical protein (ORF1), isolate 3	0.0002
	Contig638	emb|AJ749797.1|	Photobacterium damselae subsp. piscicida trpB gene for putative transposase, clone pRDA16	0.0002
	Contig782	gb|BT009458.1|	Triticum aestivum clone wlsu2.pk0001.h3:fis, full insert mRNA sequence	0.0002
	CaF1_JIE_08_A_08	emb|AM422099.2|	Danio rerio tf2a mRNA, 3'UTR	0.0002
	CaF1_JIE_15_C_05	gb|AY327035.1|	Ixodes ricinus cytochrome oxidase subunit I mRNA, partial cds; mitochondrial gene for mitochondrial product	0.0002
	CaF1_JIE_15_E_02	gb|BT009458.1|	Triticum aestivum clone wlsu2.pk0001.h3:fis, full insert mRNA sequence	0.0002
	CaF1_WIE_36_E_04	gb|AF075691.1|	Crassostrea gigas BAT1 homolog mRNA, complete cds	0.0002

	CaF1_WIE_40_G_07	gb|AY972077.1|	Synthetic construct RLS (RLS) gene, complete cds	0.0002
	CaF1_WIE_48_D_08	emb|AJ749797.1|	Photobacterium damselae subsp. piscicida trpB gene for putative transposase, clone pRDA16	0.0002
	Contig584	emb|AM748403.1|	Vigna unguiculata partial mRNA for putative rubisco activase (Rca gene), clone 17	0.0003
	CaF1_WIE_08_H_07	dbj|AB019573.1|	Homo sapiens mRNA expressed only in placental villi, clone SMAP31	0.0003
	CaF1_WIE_09_F_10	emb|AM422099.2|	Danio rerio tf2a mRNA, 3'UTR	0.0003
	CaF1_WIE_19_A_04	gb|BT009458.1|	Triticum aestivum clone wlsu2.pk0001.h3:fis, full insert mRNA sequence	0.0003
	CaF1_WIE_22_C_02	gb|AY327035.1|	Ixodes ricinus cytochrome oxidase subunit I mRNA, partial cds; mitochondrial gene for mitochondrial product	0.0003
	CaF1_WIE_27_A_05	emb|AM706414.1|	Eristalis tenax partial mRNA for hypothetical protein (ORF1), isolate 6	0.0003
	CaF1_WIE_55_C_08	gb|AY972077.1|	Synthetic construct RLS (RLS) gene, complete cds	0.0003
	CaF1_WIE_55_E_03	gb|AY972077.1|	Synthetic construct RLS (RLS) gene, complete cds	0.0003
	Contig256	emb|AM706411.1|	Eristalis tenax partial mRNA for hypothetical protein (ORF1), isolate 3	0.0004
	Contig375	gb|AY972077.1|	Synthetic construct RLS (RLS) gene, complete cds	0.0004
	Contig489	gb|AY972077.1|	Synthetic construct RLS (RLS) gene, complete cds	0.0004
	Contig542	gb|AY972077.1|	Synthetic construct RLS (RLS) gene, complete cds	0.0004
	Contig789	emb|AJ749795.1|	Photobacterium damselae subsp. piscicida partial ORF1 DNA for hypothetical protein, clone pRDA14	0.0004
	Contig791	gb|AC152347.14|	Medicago truncatula clone mth2-28c12, complete sequence	0.0004
	CaF1_JIE_20_A_07	emb|AJ749797.1|	Photobacterium damselae subsp. piscicida trpB gene for putative transposase, clone pRDA16	0.0004
	CaF1_JIE_27_D_01	emb|CT028832.1|	Poplar cDNA sequences	0.0004
	CaF1_JIE_30_F_04	dbj|AB286673.1|	Lethenteron japonicum LjHox10s gene for LjHox10s homeobox, parital cds	0.0004
	CaF1_WIE_01_B_06	gb|AY972077.1|	Synthetic construct RLS (RLS) gene, complete cds	0.0004
	CaF1_WIE_32_F_07	gb|DQ507301.1|	Belgica antarctica clone Ba-U40 CG32816-like mRNA, partial cds	0.0004
	CaF1_WIE_54_F_06	emb|AM697674.1|	Platynereis dumerilii mRNA for hypothetical protein (ORF1), isolate 2	0.0004
	Contig115	gb|DQ459385.1|	Nicotiana tabacum serine/threonine kinase mRNA, partial cds	0.0005
	Contig200	gb|AY972077.1|	Synthetic construct RLS (RLS) gene, complete cds	0.0005
	Contig535	emb|AJ749797.1|	Photobacterium damselae subsp. piscicida trpB gene for putative transposase, clone pRDA16	0.0005
	Contig549	gb|DQ072007.1|	Streptococcus mutans clone D7, genomic sequence	0.0005
	Contig692	gb|DQ459385.1|	Nicotiana tabacum serine/threonine kinase mRNA, partial cds	0.0005
	CaF1_JIE_05_G_09	gb|DQ465789.1|	Sesbania drummondii clone SSH-36_01_A09_T3 mRNA sequence	0.0005
	CaF1_JIE_24_A_06	emb|AM422119.2|	Danio rerio ca7 mRNA, 3' UTR	0.0005
	CaF1_JIE_25_G_08	emb|AJ749797.1|	Photobacterium damselae subsp. piscicida trpB gene for putative transposase, clone pRDA16	0.0005
	CaF1_WIE_08_F_10	emb|AJ293392.1|HSA293392	Homo sapiens mRNA differentially expressed in malignant melanoma, clone MM K2	0.0005
	CaF1_WIE_25_F_09	emb|AM706411.1|	Eristalis tenax partial mRNA for hypothetical protein (ORF1), isolate 3	0.0005
	CaF1_WIE_25_G_04	gb|M31921.1|VVCH2AB	V.carteri histone H2A-III and H2B-III genes, complete cds	0.0005
	CaF1_WIE_44_H_04	emb|AJ534345.1|ABI534345	Agaricus bisporus partial mRNA for putative glyoxylate pathway regulator (gpr gene), clone pm166	0.0005
	CaF1_WIE_49_A_06	emb|AJ749797.1|	Photobacterium damselae subsp. piscicida trpB gene for putative transposase, clone pRDA16	0.0005
	CaF1_WIE_49_G_11	emb|AM447853.2|	Vitis vinifera contig VV78X083454.9, whole genome shotgun sequence	0.0005
	Contig940	gb|BT009458.1|	Triticum aestivum clone wlsu2.pk0001.h3:fis, full insert mRNA sequence	0.0006
	CaF1_WIE_51_C_10	dbj|AP006431.1|	Lotus japonicus genomic DNA, chromosome 5, clone:LjT38F20, TM0328, complete sequence	0.0006
	Contig41	emb|AM422870.1|	Fagopyrum esculentum gFeAP9 gene for aspartic proteinase, exons 1-13	0.0007
	CaF1_WIE_36_B_10	emb|CT028832.1|	Poplar cDNA sequences	0.0007
	Contig770	emb|AM748504.1|	Vigna unguiculata partial mRNA for putative carbamoyl-phosphate synthase, GATase region (MtrDRAFT_AC135505g12v2 gene), clone 49	0.0008
	CaF1_JIE_02_A_02	emb|AM706411.1|	Eristalis tenax partial mRNA for hypothetical protein (ORF1), isolate 3	0.0008
	Contig294	emb|AM697674.1|	Platynereis dumerilii mRNA for hypothetical protein (ORF1), isolate 2	0.001
	Contig520	emb|AM697674.1|	Platynereis dumerilii mRNA for hypothetical protein (ORF1), isolate 2	0.001
	CaF1_JIE_16_C_06	emb|AM697674.1|	Platynereis dumerilii mRNA for hypothetical protein (ORF1), isolate 2	0.001

	CaF1_WIE_02_H_11	emb|AJ534351.1|ABI534351	Agaricus bisporus partial mRNA for putative myosin heavy chain kinase (mhck gene), clone pm31	0.001
	CaF1_WIE_48_G_01	emb|AM697674.1|	Platynereis dumerilii mRNA for hypothetical protein (ORF1), isolate 2	0.001
	CaF1_WIE_54_D_11	gb|AY972077.1|	Synthetic construct RLS (RLS) gene, complete cds	0.001
	Contig158	emb|AM748481.1|	Vigna unguiculata partial mRNA for putative ATP synthase CF1 alpha subunit (atpA gene), clone 26	0.002
	CaF1_JIE_23_F_05	emb|AM748481.1|	Vigna unguiculata partial mRNA for putative ATP synthase CF1 alpha subunit (atpA gene), clone 26	0.002
	CaF1_JIE_30_E_06	emb|AM748481.1|	Vigna unguiculata partial mRNA for putative ATP synthase CF1 alpha subunit (atpA gene), clone 26	0.002
	CaF1_JIE_41_E_08	emb|AM706411.1|	Eristalis tenax partial mRNA for hypothetical protein (ORF1), isolate 3	0.002
	CaF1_WIE_03_H_07	gb|DQ306770.1|	Hevea brasiliensis isolate SSH41 mRNA sequence	0.002
	CaF1_WIE_12_F_08	gb|AF033850.1|AF033850	Homo sapiens phospholipase D2 (PLD2) mRNA, complete cds	0.002
	CaF1_WIE_27_F_01	gb|L10211.1|ALFIOM	Medicago sativa isoliquiritigenin 2'-O-methyltransferase mRNA, complete cds	0.002
	Contig248	emb|CT029313.1|	Poplar cDNA sequences	0.003
	Contig915	emb|AM422093.1|	Danio rerio partial mRNA for lecithin retinol acetyltransferase (lrat gene)	0.003
	CaF1_JIE_40_D_01	gb|AY461597.1|	Synthetic construct arsenic-like protein gene, complete cds	0.003
	CaF1_WIE_04_F_06	gb|AC124216.27|	Medicago truncatula clone mth2-34o22, complete sequence	0.003
	Contig93	emb|AJ534351.1|ABI534351	Agaricus bisporus partial mRNA for putative myosin heavy chain kinase (mhck gene), clone pm31	0.004
	Contig551	gb|AY972077.1|	Synthetic construct RLS (RLS) gene, complete cds	0.004
	CaF1_JIE_11_E_08	gb|DQ459385.1|	Nicotiana tabacum serine/threonine kinase mRNA, partial cds	0.004
	CaF1_JIE_24_E_01	gb|AY972077.1|	Synthetic construct RLS (RLS) gene, complete cds	0.004
	CaF1_WIE_07_A_03	emb|AM748481.1|	Vigna unguiculata partial mRNA for putative ATP synthase CF1 alpha subunit (atpA gene), clone 26	0.004
	CaF1_WIE_07_A_05	gb|AC091047.10|	Homo sapiens chromosome 8, clone RP11-102F4, complete sequence	0.004
	Contig653	dbj|AB307742.1|	Antheraea yamamai ap mRNA for aminopeptidase N, partial cds	0.005
	CaF1_WIE_01_A_05	emb|AL672270.12|	Mouse DNA sequence from clone RP23-351A10 on chromosome X Contains the Rbm41 gene for RNA binding motif protein 41, the gene for a novel Nsp1-like C-terminal region containing protein, a novel gene (E230019M04Rik), a similar to glyceraldehyde-3-phosphate	0.005
	CaF1_WIE_40_C_03	gb|AY461597.1|	Synthetic construct arsenic-like protein gene, complete cds	0.005
	Contig579	gb|DQ068118.1|	Brassica napus isolate mutant Cr3529 clone Bncr10 unknown mRNA	0.006
	CaF1_JIE_29_E_08	emb|AL445196.7|	Human DNA sequence from clone RP11-31K13 on chromosome 6 Contains a heterogeneous nuclear ribonucleoprotein A1 (HNRPA1) pseudogene, the 5' end of the VMP gene for vesicular membrane protein p24 and one CpG island, complete sequence	0.006
	Contig333	gb|DQ068118.1|	Brassica napus isolate mutant Cr3529 clone Bncr10 unknown mRNA	0.007
	CaF1_WIE_06_B_01	gb|DQ465791.1|	Sesbania drummondii clone SSH-38_01_F05_T3 mRNA sequence	0.007
	Contig113	gb|DQ465767.1|	Sesbania drummondii clone SSH-14_01_F04_T3 mRNA sequence	0.008
	CaF1_WIE_13_H_04	emb|AM706411.1|	Eristalis tenax partial mRNA for hypothetical protein (ORF1), isolate 3	0.009
	CaF1_WIE_36_E_09	gb|DQ445143.1|	Beta vulgaris chromosome 9 clone BAC123 genomic sequence	0.009
	CaF1_WIE_02_A_06	emb|AM461517.2|	Vitis vinifera contig VV78X154143.3, whole genome shotgun sequence	0.013
	CaF1_WIE_28_F_06	emb|AM464167.1|	Vitis vinifera contig VV78X141930.41, whole genome shotgun sequence	0.015
	CaF1_JIE_09_E_06	ref|XM_711830.1|	Candida albicans SC5314 hypothetical protein (CaO19_12635), mRNA	0.017
	Contig389	gb|AF493232.1|	Lycopersicon pimpinellifolium cysteine protease (Rcr3) gene, complete cds	0.018
	CaF1_JIE_25_G_10	emb|CT029282.1|	Poplar cDNA sequences	0.019
	Contig432	ref|XM_458371.1|	Debaryomyces hansenii CBS767 hypothetical protein (DEHA0C16984g) partial mRNA	0.021
	Contig856	emb|CT029620.1|	Poplar cDNA sequences	0.021
	Contig74	gb|AC195567.2|	Medicago truncatula chromosome 2 BAC clone mth2-22c17, complete sequence	0.022
	Contig501	emb|CT029336.1|	Poplar cDNA sequences	0.022
	CaF1_WIE_47_H_06	emb|CT029405.1|	Poplar cDNA sequences	0.022
	CaF1_WIE_55_H_01	gb|AY972077.1|	Synthetic construct RLS (RLS) gene, complete cds	0.023
	Contig901	ref|XM_001011326.2|	Tetrahymena thermophila SB210 hypothetical protein (TTHERM_00430020) mRNA, complete cds	0.028
	CaF1_WIE_20_E_10	gb|AC110027.5|	Homo sapiens chromosome 15, clone RP11-452K20, complete sequence	0.03
	CaF1_WIE_27_B_10	gb|AY972077.1|	Synthetic construct RLS (RLS) gene, complete cds	0.039
	CaF1_WIE_48_C_01	emb|CT029389.1|	Poplar cDNA sequences	0.039
	Contig705	emb|CT028654.1|	Poplar cDNA sequences	0.044

	Contig138	emb|AM490066.1|	Dicentrarchus labrax mRNA for dicentrarchus labrax CC chemokine 2 (cc2 gene)	0.053
	Contig766	emb|CT029636.1|	Poplar cDNA sequences	0.053
	CaF1_WIE_47_A_06	gb|AY972077.1|	Synthetic construct RLS (RLS) gene, complete cds	0.075
	Contig881	emb|CT029528.1|	Poplar cDNA sequences	0.08
	CaF1_WIE_26_H_06	gb|AC122458.3|	Mus musculus BAC clone RP24-270D10 from chromosome 8, complete sequence	0.083
	CaF1_WIE_48_F_04	emb|AM231535.1|	Photorhabdus asymbiotica subsp. asymbiotica partial tra for ArsR family/rhodanese-like protein, clone 83	0.086
	CaF1_WIE_42_E_09	emb|CR626927.1|	Bacteroides fragilis NCTC 9343, complete genome	0.088
	CaF1_WIE_01_D_04	emb|AM431760.2|	Vitis vinifera contig VV78X085461.2, whole genome shotgun sequence	0.095
	Contig954	gb|AC138452.10|	Medicago truncatula clone mth2-17p11, complete sequence	0.11
	CaF1_WIE_20_H_07	emb|AM486371.2|	Vitis vinifera contig VV78X233435.4, whole genome shotgun sequence	0.11
	CaF1_WIE_43_G_05	emb|Z93765.1|MDZ93765	M.domestica mRNA for lignostilbene dioxygenase-like protein	0.11
	CaF1_WIE_47_H_01	ref|XM_001349534.1|	Plasmodium falciparum 3D7 DNA repair endonuclease, putative (PFB0265c) mRNA, complete cds	0.13
	CaF1_WIE_26_C_06	ref|XM_846823.1|	PREDICTED: Canis familiaris similar to ribosomal protein L31 (LOC609548), mRNA	0.19
	CaF1_JIE_39_G_10	emb|AL929433.10|	Mouse DNA sequence from clone RP23-193M23 on chromosome 4, complete sequence	0.21
	CaF1_WIE_27_D_07	emb|CU075768.4|	Zebrafish DNA sequence from clone DKEY-177P5 in linkage group 14, complete sequence	0.21
	Contig949	ref|XM_001309854.1|	Trichomonas vaginalis G3 variable membrane protein precursor, putative (TVAG_087500) mRNA, complete cds	0.28
	CaF1_WIE_51_D_09	emb|AJ534347.1|ABI534347	Agaricus bisporus partial mRNA for putative hexose transporter protein (htp gene)	0.28
	Contig765	gb|CP000302.1|	Shewanella denitrificans OS217, complete genome	0.3
	CaF1_WIE_01_C_07	gb|AC020551.5|	Homo sapiens BAC clone RP11-404J23 from 4, complete sequence	0.3
	CaF1_WIE_31_E_09	emb|AM748415.1|	Vigna unguiculata partial mRNA for putative proton-dependent oligopeptide transport (POT) family protein (AT3G54140 gene), clone 29	0.36
	CaF1_JIE_07_D_11	emb|AL592227.4|	Human DNA sequence from clone RP11-32D4 on chromosome 9 Contains a CpG island, complete sequence	0.41
	CaF1_WIE_39_B_02	ref|XM_001524953.1|	Lodderomyces elongisporus NRRL YB-4239 hypothetical protein (LELG_04035) mRNA, complete cds	0.49
	CaF1_JIE_38_E_01	gb|AC104660.4|	Homo sapiens BAC clone RP11-614H7 from 4, complete sequence	0.57
	Contig187	emb|CT029789.1|	Poplar cDNA sequences	0.62
	CaF1_JIE_06_A_10	gb|AC123882.6|	Mus musculus chromosome 1, clone RP23-447L15, complete sequence	0.88
	CaF1_JIE_10_G_06	gb|AE013599.4|	Drosophila melanogaster chromosome 2R, complete sequence	1.5
	Contig860	emb|CU406989.5|	Mouse DNA sequence from clone CH29-63I6 on chromosome 11, complete sequence	1.8
	CaF1_WIE_47_B_06	emb|AL392048.9|	Human DNA sequence from clone RP11-305D15 on chromosome 13 Contains the 5' end of the RB1 gene for retinoblastoma 1 (including osteosarcoma), two novel genes, the gene for purinergic receptor (family A group 5) (P2Y5), a PEST-containing nuclear protein (P	2.3
	CaF1_JIE_35_C_04	ref|NM_145102.2|	Homo sapiens zinc finger with KRAB and SCAN domains 5 (ZKSCAN5), transcript variant 2, mRNA	5.7

a. In the clone ID, the first two letters (Ca) signify the source plant, *Cicer arietinum* , the third letter (F) designates the pathogen name, *Fusarium* , and the numeral 1 designates race 1 of *Fusarium* . In the three letter abbreviation JIE and WIE, the first letter J and W refers to the JG-62 and WR-315 genotypes of chickpea, followed by (IE) signifying *Fusarium* induced library and the early time points taken for the tissue collection. For additional details, see Materials and Methods.

b. Accession number shown refers to the NCBI database.

c. The possible function of the chickpea sequences was assigned by performing BLASTX and BLASTN against nonredundant protein and nucleotide database in NCBI.

d. For BLASTX the E-value cutoff used was 10^{15} and for BLASTN the cutoff used wad 10^{-20}
