# Supplementary material for: Comparative analyses of genotype dependent expressed sequence tags and stress-responsive transcriptome of chickpea wilt illustrate predicted and unexpected genes and novel regulators of plant immunity
Source: BMC Genomics. 2009 Sep 5;10:415. doi: 10.1186/1471-2164-10-415 (PMC2755012; doi:10.1186/1471-2164-10-415)
Supplement: Additional file 4 — Gene family identification. Table listing chickpea gene families identified by single linkage clustering. [file 1471-2164-10-415-S4.pdf]

Additional file 4 - Identification of chickpea gene families by single linkage clustering

Group number[a]	Sequence name[b]	Top BLAST HIT[c]
1	CaF1_WIE_54_A_01	110 kDa 4SNc-Tudor domain protein [Pisum sativum]
1	Contig833	110 kDa 4SNc-Tudor domain protein [Pisum sativum]
2	Contig527	14-3-3-like protein [Cicer arietinum] gb|ABQ95994.1| 14-3-3-like protein [Cicer arietinum]
2	Contig947	14-3-3-like protein [Cicer arietinum] gb|ABQ95994.1| 14-3-3-like protein [Cicer arietinum]
3	CaF1_WIE_25_D_05	2,3-bisphosphoglycerate-independent phosphoglycerate mutase (Phosphoglyceromutase) (BPG-independent PGAM) (PGAM-I) gb|AAA86979.1| phosphoglyceromutase
3	Contig718	2,3-bisphosphoglycerate-independent phosphoglycerate mutase (Phosphoglyceromutase) (BPG-independent PGAM) (PGAM-I) gb|AAA86979.1| phosphoglyceromutase
4	CaF1_JIE_04_D_05	2OG-Fe(II) oxygenase [Medicago truncatula]
4	CaF1_JIE_25_A_06	2OG-Fe(II) oxygenase [Medicago truncatula]
5	CaF1_WIE_21_F_02	2-oxoglutarate dehydrogenase, E1 component [Medicago truncatula]
5	CaF1_WIE_55_H_09	2-oxoglutarate dehydrogenase, E1 component [Medicago truncatula]
6	Contig517	40S ribosomal protein S8 gb|AAC24583.1| 40S ribosomal protein S8 [Prunus armeniaca]
6	Contig66	40S ribosomal protein S8 gb|AAC24583.1| 40S ribosomal protein S8 [Prunus armeniaca]
7	CaF1_WIE_09_H_01	5-methyltetrahydropteroyltriglutamate--homocysteine S-methyltransferase; Prismane-like [Medicago truncatula] gb|ABE81639.2| 5-methyltetrahydropteroyltriglutamate--homocysteine S-methyltransferase; Prismane-like [Medicago truncatula]
7	Contig440	5-methyltetrahydropteroyltriglutamate--homocysteine S-methyltransferase; Prismane-like [Medicago truncatula] gb|ABE81639.2| 5-methyltetrahydropteroyltriglutamate--homocysteine S-methyltransferase; Prismane-like [Medicago truncatula]
7	Contig741	5-methyltetrahydropteroyltriglutamate--homocysteine S-methyltransferase; Prismane-like [Medicago truncatula] gb|ABE81639.2| 5-methyltetrahydropteroyltriglutamate--homocysteine S-methyltransferase; Prismane-like [Medicago truncatula]
7	Contig793	5-methyltetrahydropteroyltriglutamate--homocysteine S-methyltransferase; Prismane-like [Medicago truncatula] gb|ABE81639.2| 5-methyltetrahydropteroyltriglutamate--homocysteine S-methyltransferase; Prismane-like [Medicago truncatula]
8	CaF1_WIE_26_C_01	60S ribosomal protein [Medicago sativa]
8	Contig497	60S ribosomal protein [Medicago sativa]
9	CaF1_WIE_48_E_04	60S ribosomal protein L37a [Capsicum chinense]
9	Contig40	60S ribosomal protein L37a [Capsicum chinense]
10	CaF1_JIE_34_D_01	Acyl-coA-binding protein, ACBP; Serine/threonine protein phosphatase, BSU1 [Medicago truncatula]
10	Contig545	Acyl-coA-binding protein, ACBP; Serine/threonine protein phosphatase, BSU1 [Medicago truncatula]
11	CaF1_WIE_02_H_11	Agaricus bisporus partial mRNA for putative myosin heavy chain kinase (mhck gene), clone pm31
11	CaF1_WIE_36_D_07	Agaricus bisporus partial mRNA for putative myosin heavy chain kinase (mhck gene), clone pm31
11	Contig93	Agaricus bisporus partial mRNA for putative myosin heavy chain kinase (mhck gene), clone pm31
12	CaF1_JIE_42_A_04	Albumin-2 (PA2) gb|AAA02981.1| albumin 2 gb|AAA33641.1| major seed albumin prf||1314296A albumin
12	Contig532	Albumin-2 (PA2) gb|AAA02981.1| albumin 2 gb|AAA33641.1| major seed albumin prf||1314296A albumin
13	CaF1_JIE_16_B_05	Alcohol dehydrogenase 1 emb|CAA29609.1| alcohol dehydrogenase [Pisum sativum]
13	CaF1_JIE_41_B_07	Alcohol dehydrogenase 1 emb|CAA29609.1| alcohol dehydrogenase [Pisum sativum]
13	Contig286	Alcohol dehydrogenase 1 emb|CAA29609.1| alcohol dehydrogenase [Pisum sativum]
13	Contig435	Alcohol dehydrogenase 1 emb|CAA29609.1| alcohol dehydrogenase [Pisum sativum]
13	Contig488	Alcohol dehydrogenase 1 emb|CAA32934.1| unnamed protein product [Trifolium repens]
14	CaF1_JIE_05_F_11	Aldo/keto reductase [Medicago truncatula]
14	CaF1_WIE_56_D_11	Aldo/keto reductase [Medicago truncatula]
15	Contig3	Amino acid/polyamine transporter II [Medicago truncatula]
15	Contig612	Amino acid/polyamine transporter II [Medicago truncatula]
16	Contig632	AMP-dependent synthetase and ligase [Medicago truncatula]
16	Contig900	AMP-dependent synthetase and ligase [Medicago truncatula]
17	CaF1_JIE_25_B_07	Avr9/Cf-9 induced kinase 1 [Nicotiana tabacum]
17	Contig863	Avr9/Cf-9 induced kinase 1 [Nicotiana tabacum]
18	CaF1_WIE_32_F_07	Belgica antarctica clone Ba-U40 CG32816-like mRNA, partial cds
18	Contig898	Belgica antarctica clone Ba-U40 CG32816-like mRNA, partial cds
19	Contig333	Brassica napus isolate mutant Cr3529 clone Bncr10 unknown mRNA
19	Contig579	Brassica napus isolate mutant Cr3529 clone Bncr10 unknown mRNA
20	CaF1_WIE_02_H_03	C.arietinum mRNA for metallothionein (clone: CanMT-1)
20	CaF1_WIE_21_C_04	C.arietinum mRNA for metallothionein (clone: CanMT-1)
21	CaF1_WIE_36_C_09	calcium-dependent calmodulin-independent protein kinase isoform 2 [Cicer arietinum]
21	Contig377	calcium-dependent/calmodulin-independent protein kinase isoform 3 [Cicer arietinum]
22	CaF1_JIE_07_H_04	cAMP response element binding (CREB) protein [Medicago truncatula]
22	CaF1_JIE_33_D_01	cAMP response element binding (CREB) protein; Prefoldin [Medicago truncatula]
22	Contig521	cAMP response element binding (CREB) protein [Medicago truncatula]
23	Contig225	CAP protein [Medicago truncatula]

23	Contig283	hypothetical protein [Vitis vinifera]
24	CaF1_WIE_39_C_10	Catharanthus roseus clone CrP15 T-DNA sequence
24	CaF1_WIE_39_F_09	Catharanthus roseus clone CrP15 T-DNA sequence
25	CaF1_WIE_38_B_11	Catharanthus trichophyllus genotype CtN58 microsatellite CATR10 sequence
25	CaF1_WIE_38_C_11	Catharanthus trichophyllus genotype CtN58 microsatellite CATR10 sequence
25	CaF1_WIE_38_G_11	Catharanthus trichophyllus genotype CtN58 microsatellite CATR10 sequence
25	CaF1_WIE_38_H_11	Catharanthus trichophyllus genotype CtN58 microsatellite CATR10 sequence
26	CaF1_JIE_07_G_06	Cationic peroxidase 2 precursor (PNPC2) gb|AAA32676.1| cationic peroxidase
26	Contig953	Cationic peroxidase 2 precursor (PNPC2) gb|AAA32676.1| cationic peroxidase
27	CaF1_WIE_54_D_07	Cell division protein FtsZ [Medicago truncatula]
27	Contig494	Cell division protein FtsZ [Medicago truncatula]
27	Contig733	Cell division protein FtsZ [Medicago truncatula]
27	Contig876	Cell division protein FtsZ [Medicago truncatula]
28	CaF1_JIE_37_F_08	chalcone synthase [Cicer arietinum]
28	Contig780	chalcone synthase [Cicer arietinum]
29	CaF1_WIE_18_D_11	Chaperone DnaK [Medicago truncatula]
29	CaF1_WIE_40_A_06	Chaperone DnaK [Medicago truncatula]
30	CaF1_WIE_32_G_11	chitinase-related agglutinin [Robinia pseudoacacia]
30	CaF1_WIE_48_F_10	chitinase-related agglutinin [Robinia pseudoacacia]
31	CaF1_WIE_06_A_08	Cicer arietinum partial mRNA for putative extensin (ORF), clone CanEXT-1
31	CaF1_WIE_34_A_03	Cicer arietinum partial mRNA for putative extensin (ORF), clone CanEXT-1
31	CaF1_WIE_42_G_01	Cicer arietinum partial mRNA for putative extensin (ORF), clone CanEXT-1
31	Contig649	Cicer arietinum partial mRNA for putative extensin (ORF), clone CanEXT-1
32	CaF1_JIE_38_C_11	cinnamoyl-CoA reductase [Acacia mangium x Acacia auriculiformis]
32	CaF1_JIE_38_E_01	Homo sapiens BAC clone RP11-614H7 from 4, complete sequence
32	CaF1_JIE_38_E_11	Crassostrea gigas BAT1 homolog mRNA, complete cds
33	CaF1_JIE_20_A_02	class I chitinase [Cicer arietinum]
33	CaF1_WIE_01_C_08	class I chitinase [Cicer arietinum]
33	Contig411	class I chitinase [Cicer arietinum]
34	CaF1_WIE_32_B_02	Concanavalin A-like lectin/glucanase [Medicago truncatula]
34	Contig204	Concanavalin A-like lectin/glucanase [Medicago truncatula]
35	CaF1_WIE_04_A_09	copper amine oxidase [Cicer arietinum]
35	Contig381	copper amine oxidase [Cicer arietinum]
36	CaF1_WIE_32_E_11	Crassostrea gigas BAT1 homolog mRNA, complete cds
36	CaF1_WIE_36_E_04	Crassostrea gigas BAT1 homolog mRNA, complete cds
36	CaF1_WIE_40_G_02	Crassostrea gigas BAT1 homolog mRNA, complete cds
36	Contig306	Crassostrea gigas BAT1 homolog mRNA, complete cds
36	Contig460	Crassostrea gigas BAT1 homolog mRNA, complete cds
37	Contig280	Curculin-like (mannose-binding) lectin [Medicago truncatula]
37	Contig926	Curculin-like (mannose-binding) lectin [Medicago truncatula]
38	CaF1_WIE_10_B_03	Cyclic peptide transporter [Medicago truncatula]
38	CaF1_WIE_17_E_05	Cyclic peptide transporter [Medicago truncatula]
39	CaF1_WIE_44_D_03	Cyclin-like F-box [Medicago truncatula]
39	Contig421	Cyclin-like F-box [Medicago truncatula]
40	Contig190	cytochrome P450 [Cicer arietinum]
40	Contig197	cytochrome P450 [Cicer arietinum]
40	Contig315	cytochrome P450 [Cicer arietinum]
40	Contig486	cytochrome P450 [Cicer arietinum]
40	Contig598	cytochrome P450 [Cicer arietinum]
40	Contig613	cytochrome P450 [Cicer arietinum]
40	Contig669	cytochrome P450 [Cicer arietinum]
41	CaF1_JIE_23_C_09	cytochrome P450 monooxygenase [Cicer arietinum]
41	CaF1_WIE_05_E_09	cytochrome P450 monooxygenase [Cicer arietinum]
41	CaF1_WIE_19_E_01	cytochrome P450 monooxygenase [Cicer arietinum]
41	Contig313	cytochrome P450 monooxygenase [Cicer arietinum]
42	Contig317	cytochrome P450 monooxygenase CYP83G1 [Medicago truncatula]
42	Contig868	cytochrome P450 monooxygenase CYP83G1 [Medicago truncatula]
43	CaF1_JIE_01_D_02	cytosolic malate dehydrogenase [Cicer arietinum]
43	CaF1_WIE_16_G_04	cytosolic malate dehydrogenase [Cicer arietinum]
44	CaF1_JIE_08_A_08	Danio rerio tf2a mRNA, 3'UTR
44	CaF1_WIE_09_F_10	Danio rerio tf2a mRNA, 3'UTR
45	Contig235	DEAD box RNA helicase [Pisum sativum] gb|AAR97917.1| DEAD box RNA helicase [Pisum sativum]
45	Contig47	DEAD box RNA helicase [Pisum sativum] gb|AAR97917.1| DEAD box RNA helicase [Pisum sativum]
45	Contig779	DEAD box RNA helicase [Pisum sativum]
46	CaF1_JIE_30_D_06	DECOY (exp=-1; , putative [Medicago truncatula]
46	Contig141	DECOY (exp=-1; , putative [Medicago truncatula]
47	CaF1_WIE_03_C_03	Dihydrolipoyl dehydrogenase, mitochondrial precursor (Dihydrolipoamide dehydrogenase) (Pyruvate dehydrogenase complex E3 subunit) (PDC-E3) (E3) (Glycine cleavage system L protein) emb|CAA44729.1| lipoamide dehydrogenase [Pisum sativum] emb|CAA45066.2| dihydrolipoamide dehydrogenase [Pisum sativum]
47	CaF1_WIE_03_H_03	Dihydrolipoyl dehydrogenase, mitochondrial precursor (Dihydrolipoamide dehydrogenase) (Pyruvate dehydrogenase complex E3 subunit) (PDC-E3) (E3) (Glycine cleavage system L protein) emb|CAA44729.1| lipoamide dehydrogenase [Pisum sativum] emb|CAA45066.2| dihydrolipoamide dehydrogenase [Pisum sativum]

48	CaF1_WIE_52_E_05	DNA binding / transcription factor [Arabidopsis thaliana]
48	Contig934	DNA binding / transcription factor [Arabidopsis thaliana]
49	Contig619	DnaJ-like protein [Medicago sativa] gb|AAC19391.1| DnaJ-like protein MsJ1 [Medicago sativa]
49	Contig721	DnaJ-like protein [Medicago sativa] gb|AAC19391.1| DnaJ-like protein MsJ1 [Medicago sativa]
50	CaF1_WIE_54_D_03	elongation factor EF-2 [Pisum sativum]
50	Contig566	elongation factor EF-2 [Pisum sativum]
51	CaF1_WIE_23_A_04	enolase [Glycine max]
51	Contig134	enolase [Glycine max]
52	CaF1_JIE_10_C_01	Eristalis tenax partial mRNA for hypothetical protein (ORF1), isolate 3
52	CaF1_JIE_12_C_01	Eristalis tenax partial mRNA for hypothetical protein (ORF1), isolate 3
52	CaF1_JIE_19_A_09	Eristalis tenax partial mRNA for hypothetical protein (ORF1), isolate 3
52	CaF1_JIE_19_G_10	Eristalis tenax partial mRNA for hypothetical protein (ORF1), isolate 3
52	CaF1_JIE_22_D_10	Eristalis tenax partial mRNA for hypothetical protein (ORF1), isolate 3
52	CaF1_JIE_25_D_02	Eristalis tenax partial mRNA for hypothetical protein (ORF1), isolate 3
52	CaF1_JIE_35_H_02	Eristalis tenax partial mRNA for hypothetical protein (ORF1), isolate 3
52	CaF1_JIE_40_C_08	Eristalis tenax partial mRNA for hypothetical protein (ORF1), isolate 3
52	CaF1_JIE_40_H_02	Eristalis tenax partial mRNA for hypothetical protein (ORF1), isolate 3
52	CaF1_JIE_41_E_08	Eristalis tenax partial mRNA for hypothetical protein (ORF1), isolate 3
52	CaF1_WIE_13_H_04	Eristalis tenax partial mRNA for hypothetical protein (ORF1), isolate 3
52	CaF1_WIE_24_F_04	Eristalis tenax partial mRNA for hypothetical protein (ORF1), isolate 3
52	CaF1_WIE_25_F_09	Eristalis tenax partial mRNA for hypothetical protein (ORF1), isolate 3
52	CaF1_WIE_27_A_05	Eristalis tenax partial mRNA for hypothetical protein (ORF1), isolate 6
52	CaF1_WIE_35_E_06	Eristalis tenax partial mRNA for hypothetical protein (ORF1), isolate 3
52	CaF1_WIE_37_C_10	Eristalis tenax partial mRNA for hypothetical protein (ORF1), isolate 3
52	CaF1_WIE_46_E_03	Eristalis tenax partial mRNA for hypothetical protein (ORF1), isolate 3
52	CaF1_WIE_46_G_10	Eristalis tenax partial mRNA for hypothetical protein (ORF1), isolate 3
52	CaF1_WIE_47_D_08	Eristalis tenax partial mRNA for hypothetical protein (ORF1), isolate 3
52	CaF1_WIE_50_D_06	Eristalis tenax partial mRNA for hypothetical protein (ORF1), isolate 3
52	Contig211	Eristalis tenax partial mRNA for hypothetical protein (ORF1), isolate 3
52	Contig256	Eristalis tenax partial mRNA for hypothetical protein (ORF1), isolate 3
52	Contig262	Eristalis tenax partial mRNA for hypothetical protein (ORF1), isolate 3
52	Contig35	Eristalis tenax partial mRNA for hypothetical protein (ORF1), isolate 3
52	Contig402	Eristalis tenax partial mRNA for hypothetical protein (ORF1), isolate 3
52	Contig414	Eristalis tenax partial mRNA for hypothetical protein (ORF1), isolate 3
52	Contig448	Eristalis tenax partial mRNA for hypothetical protein (ORF1), isolate 3
52	Contig560	Eristalis tenax partial mRNA for hypothetical protein (ORF1), isolate 3
52	Contig950	Eristalis tenax partial mRNA for hypothetical protein (ORF1), isolate 3
53	CaF1_WIE_41_G_03	flag-tagged protein kinase domain of putative mitogen-activated protein kinase kinase kinase [synthetic construct]
53	Contig461	flag-tagged protein kinase domain of putative mitogen-activated protein kinase kinase kinase [synthetic construct]
54	Contig224	Fructose-bisphosphate aldolase, cytoplasmic isozyme emb|CAA06308.1| cytosolic fructose-1,6-bisphosphate aldolase [Cicer arietinum]
54	Contig495	Fructose-bisphosphate aldolase, cytoplasmic isozyme emb|CAA06308.1| cytosolic fructose-1,6-bisphosphate aldolase [Cicer arietinum]
55	CaF1_JIE_26_B_04	Fusarium oxysporum f. sp. lycopersici insertion sequence Foxy
55	CaF1_WIE_03_H_08	Fusarium oxysporum f. sp. lycopersici insertion sequence Foxy
55	CaF1_WIE_07_E_04	Fusarium oxysporum f. sp. lycopersici insertion sequence Foxy
55	Contig124	Fusarium oxysporum f. sp. lycopersici insertion sequence Foxy
55	Contig636	Fusarium oxysporum f. sp. lycopersici insertion sequence Foxy
56	CaF1_WIE_47_E_01	Fusarium oxysporum f. sp. lycopersici six1 gene, fot5 gene, six2 gene, shh1 gene and ORF2 (partial)
56	Contig123	Fusarium oxysporum f. sp. lycopersici six1 gene, fot5 gene, six2 gene, shh1 gene and ORF2 (partial)
56	Contig276	Fusarium oxysporum f. sp. lycopersici six1 gene, fot5 gene, six2 gene, shh1 gene and ORF2 (partial)
56	Contig361	Fusarium oxysporum f. sp. lycopersici six1 gene, fot5 gene, six2 gene, shh1 gene and ORF2 (partial)
56	Contig70	Fusarium oxysporum f. sp. lycopersici six1 gene, fot5 gene, six2 gene, shh1 gene and ORF2 (partial)
56	Contig722	Fusarium oxysporum f. sp. lycopersici six1 gene, fot5 gene, six2 gene, shh1 gene and ORF2 (partial)
56	Contig73	Fusarium oxysporum f. sp. lycopersici six1 gene, fot5 gene, six2 gene, shh1 gene and ORF2 (partial)
56	Contig762	Fusarium oxysporum f. sp. lycopersici six1 gene, fot5 gene, six2 gene, shh1 gene and ORF2 (partial)
57	CaF1_JIE_32_G_08	Fusarium oxysporum f. sp. vasinfectum strain Ag149 Foxy transposable element, partial sequence
57	CaF1_WIE_12_C_02	Fusarium oxysporum f. sp. vasinfectum strain Ag149 Foxy transposable element, partial sequence
57	CaF1_WIE_32_G_05	Fusarium oxysporum f. sp. vasinfectum strain Ag149 Foxy transposable element, partial sequence
58	CaF1_JIE_01_H_08	Fusarium oxysporum f. sp. vasinfectum strain Ag149-III Foxy transposable element, partial sequence

58	CaF1_WIE_13_F_03	Fusarium oxysporum f. sp. vasinfectum strain Ag149-III Foxy transposable element, partial sequence
59	CaF1_JIE_05_H_07	Fusarium oxysporum f. sp. vasinfectum strain X515-II Foxy transposable element, partial sequence
59	CaF1_JIE_06_E_10	Fusarium oxysporum f. sp. vasinfectum strain X515-II Foxy transposable element, partial sequence
59	Contig318	Fusarium oxysporum f. sp. vasinfectum strain X515-II Foxy transposable element, partial sequence
59	Contig351	Fusarium oxysporum f. sp. vasinfectum strain X515-II Foxy transposable element, partial sequence
60	Contig805	Fusarium oxysporum voucher VPRI 19292 mitochondrion, partial genome
60	Contig893	Fusarium oxysporum voucher VPRI 19292 mitochondrion, partial genome
61	CaF1_JIE_32_G_03	GDP dissociation inhibitor [Cicer arietinum]
61	CaF1_WIE_36_A_09	GDP dissociation inhibitor [Cicer arietinum]
62	CaF1_WIE_51_F_04	General substrate transporter [Medicago truncatula] gb|ABN09010.1| General substrate transporter [Medicago truncatula]
62	Contig219	General substrate transporter [Medicago truncatula]
62	Contig938	General substrate transporter [Medicago truncatula]
63	CaF1_WIE_03_H_10	glucan-endo-1,3-beta-glucosidase [Cicer arietinum]
63	Contig153	glucan-endo-1,3-beta-glucosidase [Cicer arietinum]
63	Contig603	glucan-endo-1,3-beta-glucosidase [Cicer arietinum]
64	Contig278	Glutathione S-transferase, C-terminal-like; Thioredoxin fold [Medicago truncatula]
64	Contig917	Glutathione S-transferase, C-terminal-like; Thioredoxin fold [Medicago truncatula]
65	CaF1_WIE_06_A_01	Glycoside hydrolase, family 1 [Medicago truncatula]
65	CaF1_WIE_47_A_05	Glycoside hydrolase, family 1 [Medicago truncatula]
65	Contig870	Glycoside hydrolase, family 1 [Medicago truncatula]
66	CaF1_WIE_52_B_09	Granulin; Peptidase C1A, papain [Medicago truncatula]
66	Contig481	Granulin; Peptidase C1A, papain [Medicago truncatula]
66	Contig874	Granulin; Peptidase C1A, papain [Medicago truncatula]
67	Contig290	GTP-binding protein [Pisum sativum] emb|CAA82707.1| guanine nucleotide regulatory protein [Vicia faba] prf||2001457K GTP-binding protein prf||2115367A small GTP-binding protein
67	Contig5	GTP-binding protein [Pisum sativum] prf||2001457G GTP-binding protein
68	CaF1_WIE_03_F_01	Haem peroxidase, plant/fungal/bacterial [Medicago truncatula]
68	CaF1_WIE_32_H_10	Haem peroxidase, plant/fungal/bacterial [Medicago truncatula]
68	Contig193	Haem peroxidase, plant/fungal/bacterial [Medicago truncatula]
68	Contig194	serine/threonine/tyrosine kinase [Arachis hypogaea]
69	CaF1_WIE_14_G_09	Heat shock protein DnaJ [Medicago truncatula]
69	CaF1_WIE_36_D_01	Heat shock protein DnaJ [Medicago truncatula]
70	Contig212	Histone deacetylase HDT1 (Histone deacetylase 2a) (HD2a) (Nucleolar histone deacetylase HD2-p39) gb|AAN03465.1| nucleolar histone deacetylase HD2-P39 [Glycine max]
70	Contig713	Histone deacetylase HDT1 (Histone deacetylase 2a) (HD2a) (Nucleolar histone deacetylase HD2-p39) gb|AAN03465.1| nucleolar histone deacetylase HD2-P39 [Glycine max]
71	CaF1_WIE_48_C_04	HMG-CoA reductase [Cucumis melo]
71	CaF1_WIE_48_D_02	HMG-CoA reductase [Cucumis melo]
72	CaF1_WIE_37_A_10	homeodomain leucine zipper protein HDZ1 [Phaseolus vulgaris]
72	Contig452	homeodomain leucine zipper protein HDZ1 [Phaseolus vulgaris]
73	CaF1_WIE_31_D_10	hypothetical protein [Arabidopsis thaliana]
73	Contig680	hypothetical protein [Arabidopsis thaliana]
74	CaF1_JIE_35_E_02	C2 [Medicago truncatula]
74	Contig608	C2; Peptidase, cysteine peptidase active site [Medicago truncatula]
75	CaF1_WIE_46_D_06	Calcium-binding EF-hand; Ras small GTPase, Rho type [Medicago truncatula]
75	Contig234	Calcium-binding EF-hand [Medicago truncatula]
76	CaF1_WIE_51_C_04	hypothetical protein [Trifolium pratense]
76	CaF1_WIE_56_F_04	hypothetical protein [Trifolium pratense]
77	CaF1_WIE_15_H_09	hypothetical protein [Nicotiana tabacum]
77	Contig428	hypothetical protein [Nicotiana tabacum]
77	Contig451	hypothetical protein [Nicotiana tabacum]
78	CaF1_JIE_39_H_08	hypothetical protein [Cicer arietinum]
78	CaF1_JIE_40_D_03	hypothetical protein [Cicer arietinum]
79	CaF1_JIE_06_E_05	hypothetical protein [Gibberella fujikuroi]
79	Contig292	hypothetical protein [Gibberella fujikuroi]
80	CaF1_WIE_02_C_03	hypothetical protein [Vitis vinifera]
80	Contig964	hypothetical protein [Vitis vinifera]
81	Contig106	hypothetical protein [Vitis vinifera]
81	Contig797	hypothetical protein [Vitis vinifera]
82	CaF1_WIE_47_C_02	hypothetical protein [Vitis vinifera]
82	Contig442	hypothetical protein [Vitis vinifera]
83	CaF1_JIE_32_A_06	hypothetical protein [Vitis vinifera]
83	CaF1_WIE_04_D_11	hypothetical protein [Vitis vinifera]
84	CaF1_WIE_46_B_07	hypothetical protein [Vitis vinifera]
84	Contig63	hypothetical protein [Vitis vinifera]
85	CaF1_WIE_28_D_11	hypothetical protein [Vitis vinifera]
85	CaF1_WIE_42_F_08	hypothetical protein [Vitis vinifera]

86	CaF1_WIE_56_E_08	hypothetical protein [Picea mariana]
86	Contig829	hypothetical protein [Picea mariana]
87	CaF1_JIE_15_A_09	hypothetical protein OsI_018180 [Oryza sativa (indica cultivar-group)]
87	CaF1_WIE_05_G_05	hypothetical protein OsI_018180 [Oryza sativa (indica cultivar-group)]
88	Contig338	hypothetical protein OsJ_009000 [Oryza sativa (japonica cultivar-group)]
88	Contig529	hypothetical protein OsJ_009000 [Oryza sativa (japonica cultivar-group)]
89	CaF1_JIE_18_B_01	hypothetical protein OsJ_025206 [Oryza sativa (japonica cultivar-group)]
89	CaF1_JIE_18_C_07	hypothetical protein DEHA0F03157g [Debaryomyces hansenii CBS767] emb|CAG88798.1| unnamed protein product [Debaryomyces hansenii CBS767]
90	Contig37	hypothetical protein FG02001.1 [Gibberella zeae PH-1]
90	Contig72	hypothetical protein FG02001.1 [Gibberella zeae PH-1]
91	CaF1_JIE_20_G_10	hypothetical protein FG04176.1 [Gibberella zeae PH-1]
91	CaF1_JIE_21_B_05	putative glycyl tRNA synthetase [Arabidopsis thaliana]
92	CaF1_JIE_19_C_02	hypothetical protein FG07152.1 [Gibberella zeae PH-1]
92	Contig364	hypothetical protein FG07152.1 [Gibberella zeae PH-1]
93	Contig195	hypothetical protein FG09053.1 [Gibberella zeae PH-1]
93	Contig409	hypothetical protein FG09053.1 [Gibberella zeae PH-1]
94	Contig591	isoflavone 3'-hydroxylase [Medicago truncatula]
94	Contig719	isoflavone 3'-hydroxylase [Medicago truncatula]
95	CaF1_JIE_15_C_05	Ixodes ricinus cytochrome oxidase subunit I mRNA, partial cds; mitochondrial gene for mitochondrial product
95	CaF1_WIE_22_C_02	Ixodes ricinus cytochrome oxidase subunit I mRNA, partial cds; mitochondrial gene for mitochondrial product
96	CaF1_WIE_25_A_01	LEM3 (ligand-effect modulator 3) family protein / CDC50 family protein [Arabidopsis thaliana] gb|AAD25612.1|AC005287_14 Unknown protein [Arabidopsis thaliana] gb|AAL38602.1|AF446869_1 At1g54320/F20D21_50 [Arabidopsis thaliana] gb|AAK74030.1| At1g54320/F20D21_50 [Arabidopsis thaliana] gb|AAK96636.1| At1g54320/F20D21_50 [Arabidopsis thaliana]
96	Contig513	LEM3 (ligand-effect modulator 3) family protein / CDC50 family protein [Arabidopsis thaliana] gb|AAD25612.1|AC005287_14 Unknown protein [Arabidopsis thaliana] gb|AAL38602.1|AF446869_1 At1g54320/F20D21_50 [Arabidopsis thaliana] gb|AAK74030.1| At1g54320/F20D21_50 [Arabidopsis thaliana] gb|AAK96636.1| At1g54320/F20D21_50 [Arabidopsis thaliana]
97	CaF1_WIE_30_B_03	Lotus japonicus genomic DNA, chromosome 3, clone:LjT10E18, TM0035, complete sequence
97	CaF1_WIE_41_H_04	Lotus japonicus genomic DNA, chromosome 3, clone:LjT10E18, TM0035, complete sequence
98	CaF1_WIE_52_F_05	M.truncatula DNA sequence from clone MTH2-28N4 on chromosome 3, complete sequence
98	Contig420	M.truncatula DNA sequence from clone MTH2-28N4 on chromosome 3, complete sequence
99	Contig148	Malic oxidoreductase [Medicago truncatula]
99	Contig804	Malic oxidoreductase [Medicago truncatula]
99	Contig918	Malic oxidoreductase [Medicago truncatula]
100	CaF1_WIE_10_C_07	Medicago truncatula chromosome 5 clone mth4-20m5, COMPLETE SEQUENCE
100	Contig101	Medicago truncatula chromosome 5 clone mth4-20m5, COMPLETE SEQUENCE
101	CaF1_WIE_25_D_07	Medicago truncatula clone mth2-17d15, complete sequence
101	Contig547	Medicago truncatula clone mth2-17d15, complete sequence
102	CaF1_WIE_37_G_09	Medicago truncatula clone mth2-18h17, complete sequence
102	Contig407	Medicago truncatula clone mth2-18h17, complete sequence
103	CaF1_WIE_23_D_03	Medicago truncatula clone mth2-1o14, complete sequence
103	CaF1_WIE_48_D_10	Medicago truncatula clone mth2-1o14, complete sequence
104	CaF1_WIE_04_G_10	Medicago truncatula clone mth2-69j4, complete sequence
104	CaF1_WIE_05_E_04	Medicago truncatula clone mth2-69j4, complete sequence
104	CaF1_WIE_48_G_04	Medicago truncatula clone mth2-69j4, complete sequence
105	CaF1_WIE_36_A_08	Medicago truncatula clone mth2-85g12, complete sequence
105	CaF1_WIE_47_B_07	Medicago truncatula clone mth2-85g12, complete sequence
106	Contig423	Medicago truncatula mRNA for MtN4 gene, partial
106	Contig772	Medicago truncatula mRNA for MtN4 gene, partial
107	Contig152	methionine sulfoxide reductase A [Populus trichocarpa x Populus deltoides]
107	Contig555	methionine sulfoxide reductase A [Populus trichocarpa x Populus deltoides]
108	Contig289	NAD-dependent epimerase/dehydratase [Medicago truncatula]
108	Contig660	NAD-dependent epimerase/dehydratase [Medicago truncatula]
108	Contig706	NAD-dependent epimerase/dehydratase [Medicago truncatula]
109	Contig390	Nascent polypeptide-associated complex NAC; UBA-like [Medicago truncatula]
109	Contig398	Nascent polypeptide-associated complex NAC [Medicago truncatula]
109	Contig526	Nascent polypeptide-associated complex NAC; UBA-like [Medicago truncatula]
110	CaF1_WIE_38_D_10	Nerium oleander microsatellite CATR25 sequence
110	CaF1_WIE_38_E_07	Nerium oleander microsatellite CATR25 sequence
110	Contig825	Nerium oleander microsatellite CATR25 sequence
111	CaF1_JIE_11_E_08	Nicotiana tabacum serine/threonine kinase mRNA, partial cds
111	CaF1_JIE_25_F_03	Nicotiana tabacum serine/threonine kinase mRNA, partial cds
111	CaF1_WIE_01_B_07	Nicotiana tabacum serine/threonine kinase mRNA, partial cds
111	CaF1_WIE_03_F_02	Nicotiana tabacum serine/threonine kinase mRNA, partial cds
111	CaF1_WIE_40_G_08	Nicotiana tabacum serine/threonine kinase mRNA, partial cds
111	Contig115	Nicotiana tabacum serine/threonine kinase mRNA, partial cds

111	Contig692	Nicotiana tabacum serine/threonine kinase mRNA, partial cds
111	Contig700	Nicotiana tabacum serine/threonine kinase mRNA, partial cds
111	Contig944	Nicotiana tabacum serine/threonine kinase mRNA, partial cds
112	Contig678	nitrite reductase [Fusarium oxysporum]
112	Contig91	nitrite reductase [Fusarium oxysporum]
113	Contig767	O-methyltransferase, family 2; Dimerisation [Medicago truncatula]
113	Contig933	O-methyltransferase, family 2; Dimerisation [Medicago truncatula]
114	CaF1_JIE_01_C_09	Orn/DAP/Arg decarboxylase 2; Protease-associated PA; Proteinase inhibitor I9, subtilisin propeptide [Medicago truncatula]
114	CaF1_WIE_50_C_09	Orn/DAP/Arg decarboxylase 2; Protease-associated PA; Proteinase inhibitor I9, subtilisin propeptide [Medicago truncatula]
115	Contig111	Pathogenesis-related transcriptional factor and ERF [Medicago truncatula]
115	Contig458	Pathogenesis-related transcriptional factor and ERF [Medicago truncatula]
116	CaF1_JIE_41_E_01	Pectinesterase [Medicago truncatula]
116	Contig36	Pectinesterase [Medicago truncatula]
117	CaF1_JIE_28_D_10	pectinesterase family protein [Arabidopsis thaliana] gb|AAF02886.1|AC009525_20 Similar to pectinesterases [Arabidopsis thaliana] gb|ABO38784.1| At1g02810 [Arabidopsis thaliana]
117	CaF1_WIE_32_H_04	pectinesterase family protein [Arabidopsis thaliana] gb|AAF02886.1|AC009525_20 Similar to pectinesterases [Arabidopsis thaliana] gb|ABO38784.1| At1g02810 [Arabidopsis thaliana]
118	CaF1_WIE_03_E_02	Peptidase S10, serine carboxypeptidase [Medicago truncatula]
118	Contig687	Peptidase S10, serine carboxypeptidase [Medicago truncatula]
118	Contig730	Peptidase S10, serine carboxypeptidase [Medicago truncatula]
119	Contig557	Peptidase S24, S26A and S26B [Medicago truncatula]
119	Contig746	Peptidase S24, S26A and S26B [Medicago truncatula]
120	CaF1_WIE_55_D_03	Peptidase T1A, proteasome beta-subunit [Medicago truncatula]
120	Contig618	Peptidase T1A, proteasome beta-subunit [Medicago truncatula]
121	CaF1_JIE_40_E_05	peroxidase [Pisum sativum]
121	Contig732	peroxidase [Pisum sativum]
122	CaF1_WIE_02_H_08	peroxidase, putative [Arabidopsis thaliana] sp|Q9FX85|PER10_ARATH Peroxidase 10 precursor (Atperox P10) (ATP5a) gb|AAG13043.1|AC011807_2 peroxidase ATP5a [Arabidopsis thaliana] dbj|BAC43700.1| putative peroxidase [Arabidopsis thaliana] gb|AAP12891.1| At1g49570 [Arabidopsis thaliana]
122	Contig254	peroxidase, putative [Arabidopsis thaliana]
123	Contig323	phenylalanine ammonia lyase [Trifolium pratense]
123	Contig616	phenylalanine ammonia lyase [Trifolium pratense]
124	CaF1_WIE_47_A_11	Phosphoesterase, DHHA1 [Medicago truncatula]
124	CaF1_WIE_47_B_06	Human DNA sequence from clone RP11-305D15 on chromosome 13 Contains the 5' end of the RB1 gene for retinoblastoma 1 (including osteosarcoma), two novel genes, the gene for purinergic receptor (family A group 5) (P2Y5), a PEST-containing nuclear protein (P
125	CaF1_JIE_17_E_07	Photobacterium damselae subsp. piscicida partial coi genes for putative cytochrome C oxidase proteins, clone pRDA19
125	CaF1_JIE_18_E_07	Photobacterium damselae subsp. piscicida partial coi genes for putative cytochrome C oxidase proteins, clone pRDA19
125	CaF1_JIE_32_G_09	Photobacterium damselae subsp. piscicida partial coi genes for putative cytochrome C oxidase proteins, clone pRDA19
125	CaF1_JIE_34_B_03	Photobacterium damselae subsp. piscicida partial coi genes for putative cytochrome C oxidase proteins, clone pRDA19
125	CaF1_JIE_41_A_10	Photobacterium damselae subsp. piscicida partial coi genes for putative cytochrome C oxidase proteins, clone pRDA19
125	CaF1_WIE_04_C_07	Photobacterium damselae subsp. piscicida partial coi genes for putative cytochrome C oxidase proteins, clone pRDA19
125	CaF1_WIE_05_B_10	Photobacterium damselae subsp. piscicida partial coi genes for putative cytochrome C oxidase proteins, clone pRDA19
125	CaF1_WIE_16_C_09	Photobacterium damselae subsp. piscicida partial coi genes for putative cytochrome C oxidase proteins, clone pRDA19
126	CaF1_JIE_29_D_09	Photobacterium damselae subsp. piscicida partial ORF1 DNA for hypothetical protein, clone pRDA24
126	CaF1_WIE_32_H_09	Photobacterium damselae subsp. piscicida partial ORF1 DNA for hypothetical protein, clone pRDA24
127	CaF1_JIE_36_A_06	Photobacterium damselae subsp. piscicida trpA gene for putative transposase and partial ORF1 DNA for hypothetical protein, clone pRDA13
127	CaF1_WIE_12_H_10	Photobacterium damselae subsp. piscicida trpA gene for putative transposase and partial ORF1 DNA for hypothetical protein, clone pRDA13
127	Contig418	Photobacterium damselae subsp. piscicida trpA gene for putative transposase and partial ORF1 DNA for hypothetical protein, clone pRDA13
127	Contig96	Photobacterium damselae subsp. piscicida trpA gene for putative transposase and partial ORF1 DNA for hypothetical protein, clone pRDA13
128	CaF1_JIE_20_A_07	Photobacterium damselae subsp. piscicida trpB gene for putative transposase, clone pRDA16
128	CaF1_JIE_20_A_11	HHP4 (heptahelical protein 4); receptor [Arabidopsis thaliana] gb|AAK25883.1|AF360173_1 unknown protein [Arabidopsis thaliana] emb|CAB38307.1| putative protein [Arabidopsis thaliana] emb|CAB80433.1| putative protein [Arabidopsis thaliana] gb|AAL07197.1| unknown protein [Arabidopsis thaliana]

128	CaF1_JIE_25_G_08	Photobacterium damselae subsp. piscicida trpB gene for putative transposase, clone pRDA16
128	CaF1_WIE_05_G_07	Photobacterium damselae subsp. piscicida trpB gene for putative transposase, clone pRDA16
128	CaF1_WIE_48_D_08	Photobacterium damselae subsp. piscicida trpB gene for putative transposase, clone pRDA16
128	CaF1_WIE_49_A_06	Photobacterium damselae subsp. piscicida trpB gene for putative transposase, clone pRDA16
128	Contig44	Photobacterium damselae subsp. piscicida trpB gene for putative transposase, clone pRDA16
128	Contig535	Photobacterium damselae subsp. piscicida trpB gene for putative transposase, clone pRDA16
128	Contig638	Photobacterium damselae subsp. piscicida trpB gene for putative transposase, clone pRDA16
128	Contig808	Photobacterium damselae subsp. piscicida trpB gene for putative transposase, clone pRDA16
129	Contig773	Plant lipid transfer/seed storage/trypsin-alpha amylase inhibitor [Medicago truncatula]
129	Contig941	Plant lipid transfer/seed storage/trypsin-alpha amylase inhibitor [Medicago truncatula]
130	CaF1_JIE_29_G_02	Plasmodiophora brassicae 16S ribosomal RNA gene, partial sequence; mitochondrial gene for mitochondrial product
130	CaF1_JIE_37_H_06	Plasmodiophora brassicae 16S ribosomal RNA gene, partial sequence; mitochondrial gene for mitochondrial product
130	CaF1_JIE_41_C_07	Plasmodiophora brassicae 16S ribosomal RNA gene, partial sequence; mitochondrial gene for mitochondrial product
130	Contig15	Plasmodiophora brassicae 16S ribosomal RNA gene, partial sequence; mitochondrial gene for mitochondrial product
130	Contig251	Plasmodiophora brassicae 16S ribosomal RNA gene, partial sequence; mitochondrial gene for mitochondrial product
130	Contig642	Plasmodiophora brassicae 16S ribosomal RNA gene, partial sequence; mitochondrial gene for mitochondrial product
130	Contig684	Plasmodiophora brassicae 16S ribosomal RNA gene, partial sequence; mitochondrial gene for mitochondrial product
131	CaF1_JIE_16_C_06	Platynereis dumerilii mRNA for hypothetical protein (ORF1), isolate 2
131	CaF1_WIE_48_G_01	Platynereis dumerilii mRNA for hypothetical protein (ORF1), isolate 2
131	CaF1_WIE_54_D_10	Platynereis dumerilii mRNA for hypothetical protein (ORF1), isolate 2
131	CaF1_WIE_54_F_06	Platynereis dumerilii mRNA for hypothetical protein (ORF1), isolate 2
131	Contig294	Platynereis dumerilii mRNA for hypothetical protein (ORF1), isolate 2
131	Contig520	Platynereis dumerilii mRNA for hypothetical protein (ORF1), isolate 2
132	CaF1_WIE_27_C_03	PMA1_NEUCR Plasma membrane ATPase (Proton pump) [Gibberella zeae PH-1]
132	Contig806	PMA1_NEUCR Plasma membrane ATPase (Proton pump) [Gibberella zeae PH-1]
133	Contig543	polygalacturonase-like protein [Fragaria x ananassa]
133	Contig694	polygalacturonase-like protein [Fragaria x ananassa]
134	CaF1_JIE_27_D_01	Poplar cDNA sequences
134	CaF1_WIE_36_B_10	Poplar cDNA sequences
135	CaF1_WIE_01_H_08	Poplar cDNA sequences
135	CaF1_WIE_32_E_07	Poplar cDNA sequences
135	CaF1_WIE_43_F_02	Poplar cDNA sequences
136	CaF1_WIE_42_H_11	PpiC-type peptidyl-prolyl cis-trans isomerase [Medicago truncatula] gb|ABE89370.1| peptidyl-prolyl cis-trans isomerase 1 (ec 5.2.1.8) (rotamase pin1)(ppiase pin1) (mdpin1) [Medicago truncatula]
136	Contig325	PpiC-type peptidyl-prolyl cis-trans isomerase; Rhodanese-like [Medicago truncatula] gb|ABE82359.1| PpiC-type peptidyl-prolyl cis-trans isomerase; Rhodanese-like [Medicago truncatula]
137	CaF1_WIE_12_H_05	Prefoldin [Medicago truncatula]
137	CaF1_WIE_34_F_11	Prefoldin; Helix-loop-helix DNA-binding [Medicago truncatula]
138	Contig12	hypothetical protein [Vitis vinifera]
138	Contig757	hypothetical protein [Vitis vinifera]
139	CaF1_JIE_15_B_11	Protein kinase [Medicago truncatula]
139	CaF1_JIE_34_H_11	Protein kinase [Medicago truncatula]
139	CaF1_WIE_05_E_05	Protein kinase [Medicago truncatula]
139	CaF1_WIE_09_E_11	Protein kinase [Medicago truncatula]
139	CaF1_WIE_47_C_03	Protein kinase [Medicago truncatula]
139	CaF1_WIE_53_H_05	Protein kinase [Medicago truncatula]
139	Contig161	Protein kinase [Medicago truncatula]
139	Contig645	Protein kinase [Medicago truncatula]
139	Contig815	Protein kinase [Medicago truncatula]
140	CaF1_WIE_51_C_11	Protein kinase [Medicago truncatula] gb|ABE81974.1| Protein kinase [Medicago truncatula]
140	Contig903	Protein kinase [Medicago truncatula] gb|ABE81653.1| Protein kinase [Medicago truncatula]
141	Contig11	Protein of unknown function DUF676, hydrolase-like [Medicago truncatula]
141	Contig463	Protein of unknown function DUF676, hydrolase-like [Medicago truncatula] gb|ABE80808.2| Protein of unknown function DUF676, hydrolase-like [Medicago truncatula]
142	CaF1_WIE_51_A_02	Protein of unknown function DUF707 [Medicago truncatula]
142	Contig120	Protein of unknown function DUF707 [Medicago truncatula]

143	CaF1_JIE_29_G_07	Protein phosphatase 2C-like [Medicago truncatula]
143	Contig662	Protein phosphatase 2C [Medicago truncatula]
144	CaF1_WIE_35_G_07	Protein prenyltransferase [Medicago truncatula]
144	CaF1_WIE_43_H_02	Protein prenyltransferase [Medicago truncatula]
145	CaF1_WIE_10_D_09	Solanum lycopersicum cDNA, clone: FC06CB10, HTC in fruit
145	CaF1_WIE_51_H_05	Prunus armeniaca 40S ribosomal protein S8 (RPS8) mRNA, complete cds
146	CaF1_WIE_38_A_08	Pseudomonas aeruginosa gene for 16S rRNA, partial sequence, strain: Hg2
146	CaF1_WIE_38_D_08	Pseudomonas aeruginosa gene for 16S rRNA, partial sequence, strain: Hg2
146	CaF1_WIE_38_F_09	Pseudomonas aeruginosa gene for 16S rRNA, partial sequence, strain: Hg2
146	CaF1_WIE_38_F_10	Nerium oleander microsatellite CATR25 sequence
146	CaF1_WIE_38_G_10	Pseudomonas aeruginosa gene for 16S rRNA, partial sequence, strain: Hg2
146	Contig826	Pseudomonas aeruginosa gene for 16S rRNA, partial sequence, strain: Hg2
147	CaF1_WIE_35_F_01	putative ADP,ATP carrier-like protein [Trifolium pratense]
147	Contig231	putative ADP,ATP carrier-like protein [Trifolium pratense]
148	CaF1_JIE_20_D_06	putative beta-amylase [Arabidopsis thaliana]
148	CaF1_JIE_20_D_10	hypothetical protein [Vitis vinifera]
148	CaF1_WIE_40_D_06	putative beta-amylase [Arabidopsis thaliana]
149	CaF1_JIE_22_A_09	putative desaturase-like protein [Trifolium repens]
149	Contig454	putative desaturase-like protein [Trifolium repens]
150	Contig530	putative imbibition protein [Cicer arietinum]
150	Contig640	putative imbibition protein [Cicer arietinum]
151	CaF1_JIE_02_C_04	putative protein [Arabidopsis thaliana]
151	CaF1_WIE_05_E_03	putative protein [Arabidopsis thaliana]
151	CaF1_WIE_15_B_02	putative protein [Arabidopsis thaliana]
151	CaF1_WIE_26_H_04	putative protein [Arabidopsis thaliana]
152	CaF1_WIE_06_F_11	putative receptor-like GPI-anchored protein 2 [Trifolium pratense]
152	Contig357	putative receptor-like GPI-anchored protein 2 [Trifolium pratense]
153	Contig266	putative ripening related protein [Cicer arietinum]
153	Contig654	putative ripening related protein [Cicer arietinum]
154	Contig575	putative senescence-associated protein [Pisum sativum]
154	Contig962	putative senescence-associated protein [Pisum sativum]
155	CaF1_WIE_53_E_05	putative spindle disassembly related protein CDC48 [Nicotiana tabacum]
155	Contig624	putative spindle disassembly related protein CDC48 [Nicotiana tabacum]
156	CaF1_JIE_15_B_02	Pyridoxal-5-phosphate-dependent enzyme, beta subunit [Medicago truncatula] gb|ABE91493.1| Pyridoxal-5-phosphate-dependent enzyme, beta subunit [Medicago truncatula]
156	Contig265	Pyridoxal-5-phosphate-dependent enzyme, beta subunit [Medicago truncatula]
157	CaF1_JIE_19_F_11	Pyruvate decarboxylase isozyme 1 (PDC) emb|CAA91444.1| pyruvate decarboxylase [Pisum sativum]
157	CaF1_WIE_15_A_04	Pyruvate decarboxylase isozyme 1 (PDC) emb|CAA91444.1| pyruvate decarboxylase [Pisum sativum]
158	CaF1_WIE_34_B_08	Pyruvate dehydrogenase E1 component subunit beta, mitochondrial precursor (PDHE1-B) gb|AAB01223.1| pyruvate dehydrogenase E1beta
158	CaF1_WIE_50_D_11	Pyruvate dehydrogenase E1 component subunit beta, mitochondrial precursor (PDHE1-B) gb|AAB01223.1| pyruvate dehydrogenase E1beta
159	CaF1_WIE_12_E_04	Pyruvate kinase [Medicago truncatula] gb|ABE89087.1| Pyruvate kinase [Medicago truncatula]
159	Contig9	Pyruvate kinase [Medicago truncatula] gb|ABE89087.1| Pyruvate kinase [Medicago truncatula]
160	CaF1_WIE_33_C_08	Rana pirica mRNA for trypsinogen, partial sequence, clone:No 304
160	CaF1_WIE_44_C_11	Rana pirica mRNA for trypsinogen, partial sequence, clone:No 316
161	CaF1_WIE_29_B_02	Ras small GTPase, Rab type [Medicago truncatula]
161	Contig871	Ras small GTPase, Rab type [Medicago truncatula]
162	CaF1_WIE_38_A_05	Rattus norvegicus obese protein gene, 5' flanking region and partial cds
162	CaF1_WIE_38_C_05	Rattus norvegicus obese protein gene, 5' flanking region and partial cds
162	CaF1_WIE_38_H_04	Rattus norvegicus obese protein gene, 5' flanking region and partial cds
162	Contig838	Rattus norvegicus obese protein gene, 5' flanking region and partial cds
163	Contig112	Response regulator receiver; CCT [Medicago truncatula]
163	Contig270	Response regulator receiver; CCT [Medicago truncatula]
164	CaF1_WIE_52_A_07	ribosomal Pr 117 [Triticum aestivum]
164	Contig457	ribosomal Pr 117 [Triticum aestivum]
165	Contig728	Ribosomal protein L19e [Medicago truncatula]
165	Contig796	Ribosomal protein L19e [Medicago truncatula]
166	CaF1_JIE_09_D_03	Ribosomal protein L7Ae/L30e/S12e/Gadd45 [Medicago truncatula]
166	Contig906	Ribosomal protein L7Ae/L30e/S12e/Gadd45 [Medicago truncatula]
167	CaF1_WIE_50_E_03	ribosomal protein S6 [Glycine max]
167	Contig715	ribosomal protein S6 [Glycine max]
168	CaF1_WIE_10_C_04	RNA-binding region RNP-1 (RNA recognition motif) [Medicago truncatula]
168	CaF1_WIE_27_G_10	RNA-binding region RNP-1 (RNA recognition motif) [Medicago truncatula]
168	CaF1_WIE_27_H_10	RNA-binding region RNP-1 (RNA recognition motif) [Medicago truncatula]
168	Contig206	RNA-binding region RNP-1 (RNA recognition motif) [Medicago truncatula]
168	Contig744	RNA-binding region RNP-1 (RNA recognition motif) [Medicago truncatula]
168	Contig883	RNA-binding region RNP-1 (RNA recognition motif) [Medicago truncatula]
169	CaF1_JIE_10_B_07	S25 ribosomal protein [Medicago truncatula] gb|ABE85967.1| S25 ribosomal protein [Medicago truncatula]

169	Contig847	S25 ribosomal protein [Medicago truncatula] gb|ABE85967.1| S25 ribosomal protein [Medicago truncatula]
170	CaF1_WIE_01_A_01	S-adenosyl-L-homocysteine hydrolase [Cicer arietinum]
170	Contig536	S-adenosyl-L-homocysteine hydrolase [Cicer arietinum]
171	Contig714	S-adenosylmethionine synthetase 2 (Methionine adenosyltransferase 2) (AdoMet synthetase 2) emb|CAA57581.1| methionine adenosyltransferase [Pisum sativum] gb|AAA58773.1| S-adenosylmethionine synthase
171	Contig927	S-adenosylmethionine synthetase [Medicago truncatula]
172	CaF1_JIE_26_C_07	SAM (and some other nucleotide) binding motif [Medicago truncatula]
172	Contig135	SAM (and some other nucleotide) binding motif [Medicago truncatula] gb|ABE79986.1| SAM (and some other nucleotide) binding motif [Medicago truncatula] gb|ABE83018.1| SAM (and some other nucleotide) binding motif [Medicago truncatula]
172	Contig755	SAM (and some other nucleotide) binding motif [Medicago truncatula]
173	Contig823	secretory peroxidase [Catharanthus roseus]
173	Contig837	secretory peroxidase [Catharanthus roseus]
174	CaF1_WIE_50_E_05	selenium binding protein [Medicago sativa]
174	CaF1_WIE_56_G_07	selenium binding protein [Medicago sativa]
174	Contig441	selenium binding protein [Medicago sativa]
175	Contig617	serine C-palmitoyltransferase like protein [Arabidopsis thaliana] emb|CAB80314.1| serine C-palmitoyltransferase like protein [Arabidopsis thaliana]
175	Contig783	serine C-palmitoyltransferase like protein [imported] - Arabidopsis thaliana-related [Medicago truncatula]
176	CaF1_JIE_03_A_05	Serine/threonine protein kinase, active site [Medicago truncatula]
176	CaF1_JIE_03_B_10	Serine/threonine protein kinase, active site [Medicago truncatula]
176	Contig86	Serine/threonine protein kinase, active site [Medicago truncatula] gb|ABE89881.1| Serine/threonine protein kinase, active site [Medicago truncatula]
177	CaF1_WIE_24_F_06	serine/threonine/tyrosine kinase [Arachis hypogaea]
177	Contig668	serine/threonine/tyrosine kinase [Arachis hypogaea]
178	CaF1_WIE_02_D_09	Serine/threonine-protein phosphatase PP1 emb|CAA56766.1| potentially catalitic subunit of the ser /thr protein phosphatase 1 [Medicago sativa subsp. x varia]
178	CaF1_WIE_02_E_09	Serine/threonine-protein phosphatase PP1 emb|CAA56766.1| potentially catalitic subunit of the ser /thr protein phosphatase 1 [Medicago sativa subsp. x varia]
179	CaF1_WIE_47_E_02	Sesbania drummondii clone SSH-1_01_F12_T3 mRNA sequence
179	CaF1_WIE_49_D_09	Sesbania drummondii clone SSH-1_01_F12_T3 mRNA sequence
180	CaF1_JIE_05_G_09	Sesbania drummondii clone SSH-36_01_A09_T3 mRNA sequence
180	CaF1_WIE_36_E_03	Sesbania drummondii clone SSH-36_01_A09_T3 mRNA sequence
181	CaF1_JIE_15_G_09	signal recognition particle 54 kDa subunit
181	Contig171	signal recognition particle 54 kDa subunit precursor [Pisum sativum]
182	CaF1_JIE_22_B_08	Siniperca chuatsi transposase mRNA, partial cds
182	CaF1_JIE_34_G_01	Siniperca chuatsi transposase mRNA, partial cds
182	CaF1_WIE_07_G_04	Siniperca chuatsi transposase mRNA, partial cds
182	CaF1_WIE_27_E_02	Siniperca chuatsi transposase mRNA, partial cds
182	CaF1_WIE_48_D_09	Siniperca chuatsi transposase mRNA, partial cds
182	CaF1_WIE_48_E_03	Siniperca chuatsi transposase mRNA, partial cds
182	Contig172	Siniperca chuatsi transposase mRNA, partial cds
182	Contig29	Siniperca chuatsi transposase mRNA, partial cds
182	Contig354	Siniperca chuatsi transposase mRNA, partial cds
182	Contig447	Siniperca chuatsi transposase mRNA, partial cds
182	Contig504	Siniperca chuatsi transposase mRNA, partial cds
182	Contig661	Siniperca chuatsi transposase mRNA, partial cds
182	Contig699	Siniperca chuatsi transposase mRNA, partial cds
182	Contig75	Siniperca chuatsi transposase mRNA, partial cds
182	Contig812	Siniperca chuatsi transposase mRNA, partial cds
182	Contig841	Siniperca chuatsi transposase mRNA, partial cds
183	Contig405	somatic embryogenesis receptor-like kinase 3 [Arabidopsis thaliana]
183	Contig406	protein binding [Arabidopsis thaliana] dbj|BAB08479.1| leucine-rich repeat disease resistance protein-like [Arabidopsis thaliana] gb|AAM91553.1| Cf-5 disease resistance protein-like [Arabidopsis thaliana] gb|AAN15323.1| Cf-5 disease resistance protein-like [Arabidopsis thaliana]
184	Contig178	squalene epoxidase [Medicago sativa]
184	Contig365	squalene epoxidase [Medicago sativa]
184	Contig393	squalene epoxidase [Medicago sativa]
185	CaF1_WIE_46_D_11	Sucrose synthase 2 (Sucrose-UDP glucosyltransferase 2) emb|CAA04512.1| second sucrose synthase [Pisum sativum]
185	CaF1_WIE_54_E_05	Sucrose synthase 2 (Sucrose-UDP glucosyltransferase 2) emb|CAA04512.1| second sucrose synthase [Pisum sativum]
185	Contig574	Sucrose synthase 2 (Sucrose-UDP glucosyltransferase 2) emb|CAA04512.1| second sucrose synthase [Pisum sativum]
186	CaF1_JIE_07_D_04	Synthetic construct arsenic-like protein gene, complete cds
186	CaF1_JIE_40_D_01	Synthetic construct arsenic-like protein gene, complete cds
186	CaF1_WIE_40_C_03	Synthetic construct arsenic-like protein gene, complete cds
186	CaF1_WIE_51_G_06	Synthetic construct arsenic-like protein gene, complete cds
187	CaF1_JIE_24_E_01	Synthetic construct RLS (RLS) gene, complete cds
187	CaF1_WIE_01_B_06	Synthetic construct RLS (RLS) gene, complete cds
187	CaF1_WIE_17_A_10	Synthetic construct RLS (RLS) gene, complete cds
187	CaF1_WIE_27_B_10	Synthetic construct RLS (RLS) gene, complete cds

187	CaF1_WIE_40_G_07	Synthetic construct RLS (RLS) gene, complete cds
187	CaF1_WIE_47_A_06	Synthetic construct RLS (RLS) gene, complete cds
187	CaF1_WIE_54_D_11	Synthetic construct RLS (RLS) gene, complete cds
187	CaF1_WIE_54_E_06	Synthetic construct RLS (RLS) gene, complete cds
187	CaF1_WIE_55_C_08	Synthetic construct RLS (RLS) gene, complete cds
187	CaF1_WIE_55_E_03	Synthetic construct RLS (RLS) gene, complete cds
187	CaF1_WIE_55_H_01	Synthetic construct RLS (RLS) gene, complete cds
187	Contig146	Synthetic construct RLS (RLS) gene, complete cds
187	Contig200	Synthetic construct RLS (RLS) gene, complete cds
187	Contig375	Synthetic construct RLS (RLS) gene, complete cds
187	Contig471	Synthetic construct RLS (RLS) gene, complete cds
187	Contig489	Synthetic construct RLS (RLS) gene, complete cds
187	Contig542	Synthetic construct RLS (RLS) gene, complete cds
187	Contig551	Synthetic construct RLS (RLS) gene, complete cds
187	Contig620	Synthetic construct RLS (RLS) gene, complete cds
187	Contig621	Synthetic construct RLS (RLS) gene, complete cds
187	Contig952	unknown protein [Arabidopsis thaliana]
188	CaF1_WIE_34_E_11	tonoplast intrinsic protein [Cicer arietinum]
188	Contig118	tonoplast intrinsic protein [Cicer arietinum]
189	CaF1_WIE_12_G_04	Translation factor [Medicago truncatula]
189	Contig218	Translation factor [Medicago truncatula]
189	Contig23	phaseolin G-box binding protein PG2 [Phaseolus vulgaris]
189	Contig230	Translation factor [Medicago truncatula]
190	CaF1_JIE_11_E_07	Translation factor; Elongation factor G, III and V [Medicago truncatula]
190	Contig840	Translation factor; Elongation factor G, III and V [Medicago truncatula]
190	Contig921	Translation factor; Elongation factor G, III and V [Medicago truncatula]
191	Contig496	Translation protein SH3-like [Medicago truncatula]
191	Contig589	Translation protein SH3-like [Medicago truncatula]
192	CaF1_JIE_26_G_09	Translocase of chloroplast 34 (34 kDa chloroplast outer envelope protein) (GTP-binding protein OEP34) (GTP-binding protein IAP34) emb|CAA82196.1| chloroplast outer envelope protein 34 [Pisum sativum] gb|AAC25785.1| GTP-binding protein [Pisum sativum]
192	Contig626	Translocase of chloroplast 34 (34 kDa chloroplast outer envelope protein) (GTP-binding protein OEP34) (GTP-binding protein IAP34) emb|CAA82196.1| chloroplast outer envelope protein 34 [Pisum sativum] gb|AAC25785.1| GTP-binding protein [Pisum sativum]
193	CaF1_WIE_20_F_02	triosephosphate isomerase [Glycine max]
193	Contig704	triosephosphate isomerase [Glycine max]
194	CaF1_JIE_15_E_02	Triticum aestivum clone wlsu2.pk0001.h3:fis, full insert mRNA sequence
194	CaF1_WIE_19_A_04	Triticum aestivum clone wlsu2.pk0001.h3:fis, full insert mRNA sequence
194	CaF1_WIE_30_E_07	Triticum aestivum clone wlsu2.pk0001.h3:fis, full insert mRNA sequence
194	CaF1_WIE_51_A_10	Triticum aestivum clone wlsu2.pk0001.h3:fis, full insert mRNA sequence
194	Contig137	Triticum aestivum clone wlsu2.pk0001.h3:fis, full insert mRNA sequence
194	Contig257	Triticum aestivum clone wlsu2.pk0001.h3:fis, full insert mRNA sequence
194	Contig309	Triticum aestivum clone wlsu2.pk0001.h3:fis, full insert mRNA sequence
194	Contig385	Triticum aestivum clone wlsu2.pk0001.h3:fis, full insert mRNA sequence
194	Contig519	Triticum aestivum clone wlsu2.pk0001.h3:fis, full insert mRNA sequence
194	Contig782	Triticum aestivum clone wlsu2.pk0001.h3:fis, full insert mRNA sequence
194	Contig940	Triticum aestivum clone wlsu2.pk0001.h3:fis, full insert mRNA sequence
195	Contig641	tRNA synthetase class II (G, H, P and S) family protein [Arabidopsis thaliana] ref|NP_850736.1| tRNA synthetase class II (G, H, P and S) family protein [Arabidopsis thaliana] emb|CAB71872.1| multifunctional aminoacyl-tRNA ligase-like protein [Arabidopsis thaliana] gb|AAL24294.1| multifunctional aminoacyl-tRNA ligase-like protein [Arabidopsis thaliana] gb|AAM91120.1| multifunctional aminoacyl-tRNA ligase-like protein [Arabidopsis thaliana]
195	Contig748	tRNA synthetase class II (G, H, P and S) family protein [Arabidopsis thaliana] ref|NP_850736.1| tRNA synthetase class II (G, H, P and S) family protein [Arabidopsis thaliana] emb|CAB71872.1| multifunctional aminoacyl-tRNA ligase-like protein [Arabidopsis thaliana] gb|AAL24294.1| multifunctional aminoacyl-tRNA ligase-like protein [Arabidopsis thaliana] gb|AAM91120.1| multifunctional aminoacyl-tRNA ligase-like protein [Arabidopsis thaliana]
196	CaF1_JIE_40_B_07	Tubulin beta chain (Beta tubulin) emb|CAA42777.1| beta-tubulin [Glycine max]
196	CaF1_WIE_41_E_04	Tubulin beta chain (Beta tubulin) emb|CAA67056.1| beta-tubulin [Cicer arietinum]
197	CaF1_WIE_46_A_09	Medicago truncatula chromosome 2 clone mth2-33b11, complete sequence
197	Contig735	ubiquitin [Antirrhinum majus]
198	Contig57	Ubiquitin-conjugating enzyme, E2 [Medicago truncatula]
198	Contig794	Ubiquitin-conjugating enzyme, E2 [Medicago truncatula]
199	CaF1_JIE_13_C_01	Uncharacterized Cys-rich domain [Medicago truncatula]
199	CaF1_JIE_41_G_02	Uncharacterized Cys-rich domain [Medicago truncatula]
200	CaF1_WIE_37_G_08	Universal stress protein (Usp) [Medicago truncatula]

200	Contig345	universal stress protein (USP) family protein [Arabidopsis thaliana] gb|AAG40033.1|AF324682_1 AT3g53990 [Arabidopsis thaliana] gb|AAG40390.1|AF325038_1 AT3g53990 [Arabidopsis thaliana] gb|AAG41484.1|AF326902_1 unknown protein [Arabidopsis thaliana] gb|AAK00403.1|AF339721_1 unknown protein [Arabidopsis thaliana] gb|AAK32867.1|AF361855_1 AT3g53990/F5K20_290 [Arabidopsis thaliana] emb|CAB88361.1| hypothetical protein [Arabidopsis thaliana] gb|AAK96518.1| AT3g53990/F5K20_290 [Arabidopsis thaliana] gb|AAL31227.1| AT3g53990/F5K20_290 [Arabidopsis thaliana] gb|AAL49942.1| AT3g53990/F5K20_290 [Arabidopsis thaliana] dbj|BAD94963.1| hypothetical protein [Arabidopsis thaliana]
200	Contig720	Universal stress protein (Usp) [Medicago truncatula] gb|ABE88281.1| Universal stress protein (Usp) [Medicago truncatula]
200	Contig908	Universal stress protein (Usp) [Medicago truncatula]
201	Contig580	unknown [Vitis pseudoreticulata]
201	Contig677	unknown [Vitis pseudoreticulata]
202	CaF1_JIE_23_F_05	Vigna unguiculata partial mRNA for putative ATP synthase CF1 alpha subunit (atpA gene), clone 26
202	CaF1_JIE_30_E_06	Vigna unguiculata partial mRNA for putative ATP synthase CF1 alpha subunit (atpA gene), clone 26
202	CaF1_WIE_07_A_03	Vigna unguiculata partial mRNA for putative ATP synthase CF1 alpha subunit (atpA gene), clone 26
202	Contig158	Vigna unguiculata partial mRNA for putative ATP synthase CF1 alpha subunit (atpA gene), clone 26
203	CaF1_WIE_37_E_04	Vigna unguiculata partial mRNA for putative CBL-interacting protein kinase 12 (CIPK12 gene), clone 24
203	CaF1_WIE_39_H_08	Vigna unguiculata partial mRNA for putative CBL-interacting protein kinase 12 (CIPK12 gene), clone 24
204	CaF1_JIE_29_F_07	WD40-like [Medicago truncatula]
204	CaF1_WIE_24_F_03	WD40-like [Medicago truncatula]
204	CaF1_WIE_46_H_04	WD40-like [Medicago truncatula]
204	Contig114	WD-40 repeat [Medicago truncatula] gb|ABO84189.1| WD40-like [Medicago truncatula]
205	CaF1_WIE_31_G_06	wound-induced GSK-3-like protein [Medicago sativa subsp. x varia]
205	Contig959	wound-induced GSK-3-like protein [Medicago sativa subsp. x varia]
206	Contig891	Zinc finger, RING-type [Medicago truncatula]
206	Contig970	Zinc finger, RING-type [Medicago truncatula]
207	Contig247	Zinc finger, RING-type; RINGv [Medicago truncatula] gb|ABE79868.2| Zinc finger, RING-type [Medicago truncatula]
207	Contig726	Zinc finger, RING-type; RINGv [Medicago truncatula]
208	Contig663	Heat shock protein DnaJ [Medicago truncatula] gb|ABE83817.1| Heat shock protein DnaJ [Medicago truncatula] gb|ABP02364.1| Heat shock protein DnaJ [Medicago truncatula]
208	Contig664	Heat shock protein DnaJ [Medicago truncatula] gb|ABE83817.1| Heat shock protein DnaJ [Medicago truncatula] gb|ABP02364.1| Heat shock protein DnaJ [Medicago truncatula]
209	CaF1_WIE_07_D_07	Thioredoxin domain 2; Thioredoxin fold [Medicago truncatula]
209	CaF1_WIE_47_B_08	Thioredoxin domain 2; Thioredoxin fold [Medicago truncatula]

a. Group numbers are arbitrary identifiers assigned during single linkage clustering analysis of homologous sequences. Sequences belonging to specific group represent a potential gene family.

b. Sequence names containing 'Contig' represent a consensus sequence for contigged ESTs. All other sequences represent singletons

c. Top BLASTX or BLASTN hit when sequences were compared to the non redundant protein or nucleotide databases in NCBI
