# Supplementary material for: Comparative analyses of genotype dependent expressed sequence tags and stress-responsive transcriptome of chickpea wilt illustrate predicted and unexpected genes and novel regulators of plant immunity
Source: BMC Genomics. 2009 Sep 5;10:415. doi: 10.1186/1471-2164-10-415 (PMC2755012; doi:10.1186/1471-2164-10-415)
Supplement: Additional file 5 — Genotype dependent CaSNP identification. Table listing identification of single nucleotide polymorphisms (SNPs) from chickpea genotypes. [file 1471-2164-10-415-S5.pdf]

**Additional file 5 - Identification of single nucleotide polymorphisms (SNPs) from chickpea genotypes**

| Name of contig that contains SNP | Position of SNP[a] | Probability of SNP | Polymorphism JG-62 or WR-315) | Quality of SNP | Number of sequences from JG-62 that contain SNP | Number of sequences from WR-315 that contain SNP |
|---|---|---|---|---|---|---|
| Contig10 | 333 | 0.999999959 | g or a | H | 4 | 2 |
| Contig10 | 257 | 1 | c or t | L | 2 | 1 |
| Contig100 | 235 | 0.999905088 | t or c | L | 2 | 1 |
| Contig100 | 237 | 0.999622676 | t or c | L | 2 | 1 |
| Contig100 | 256 | 0.999622805 | c or a | L | 2 | 1 |
| Contig100 | 308 | 0.999801899 | t or c | L | 2 | 1 |
| Contig102 | 97 | 0.990692741 | a or c | L | 2 | 1 |
| Contig102 | 335 | 0.990132364 | c or t | L | 4 | 1 |
| Contig103 | 152 | 0.991130166 | g or a | L | 1 | 8 |
| Contig103 | 292 | 0.997180208 | c or t | L | 1 | 8 |
| Contig103 | 343 | 0.991131642 | a or g | L | 1 | 8 |
| Contig103 | 367 | 0.997906189 | c or t | L | 1 | 8 |
| Contig109 | 122 | 0.999998931 | t or c | L | 1 | 2 |
| Contig119 | 288 | 0.999999988 | a or g | L | 1 | 2 |
| Contig119 | 406 | 0.999999882 | a or c | L | 1 | 2 |
| Contig12 | 225 | 0.99980191 | t or c | L | 3 | 1 |
| Contig122 | 302 | 0.999999911 | a or c | L | 3 | 1 |
| Contig123 | 102 | 0.992891419 | t or a | H | 3 | 2 |
| Contig123 | 116 | 0.999999922 | g or a | H | 3 | 2 |
| Contig123 | 152 | 0.999999999 | c or t | H | 2 | 4 |
| Contig123 | 211 | 1 | g or a | H | 2 | 4 |
| Contig123 | 213 | 0.999999992 | t or c | H | 3 | 4 |
| Contig123 | 217 | 0.999999866 | t or c | H | 2 | 4 |
| Contig123 | 235 | 1 | a or g | H | 2 | 4 |
| Contig123 | 274 | 0.999999997 | t or c | H | 3 | 4 |
| Contig123 | 288 | 0.99999957 | t or a | H | 2 | 4 |
| Contig123 | 372 | 0.997274466 | t or a | H | 2 | 4 |
| Contig123 | 448 | 0.998808175 | a or g | H | 3 | 3 |
| Contig148 | 10 | 0.997295519 | g- | | | |
| Contig123 | 31 | 1 | a or g | L | 3 | 1 |
| Contig123 | 86 | 1 | g or a | L | 1 | 2 |
| Contig123 | 103 | 1 | g or t | L | 1 | 2 |
| Contig124 | 175 | 0.999999254 | g or a | H | 5 | 2 |
| Contig124 | 79 | 0.999999988 | a or t | L | 5 | 1 |
| Contig134 | 91 | 0.999998019 | a or g | H | 3 | 4 |
| Contig134 | 272 | 0.995274847 | c or t | H | 2 | 5 |
| Contig14 | 102 | 1 | a or g | L | 2 | 1 |
| Contig165 | 331 | 0.999247692 | g or a | H | 3 | 3 |
| Contig165 | 57 | 1 | c or t | L | 3 | 1 |
| Contig170 | 155 | 0.999999999 | t or a | L | 2 | 1 |
| Contig170 | 295 | 0.999987529 | t or c | L | 4 | 1 |
| Contig176 | 403 | 0.997025784 | c or t | L | 1 | 2 |
| Contig199 | 147 | 0.995860991 | a or t | L | 3 | 1 |
| Contig206 | 112 | 0.999734459 | g or c | H | 10 | 5 |
| Contig206 | 218 | 0.999999999 | g or t | H | 11 | 2 |
| Contig206 | 254 | 0.999999479 | a or g | H | 10 | 5 |
| Contig206 | 302 | 0.99951042 | a or g | H | 12 | 2 |
| Contig206 | 393 | 0.991219972 | a or g | H | 14 | 2 |
| Contig206 | 149 | 0.999999796 | c or t | H | 5 | 33 |
| Contig206 | 432 | 1 | t or c | L | 8 | 1 |
| Contig218 | 87 | 0.999668819 | c or t | L | 5 | 1 |
| Contig218 | 488 | 0.999767291 | a or g | L | 4 | 1 |
| Contig218 | 570 | 0.992893946 | t or c | L | 4 | 1 |
| Contig222 | 92 | 0.999999974 | t or c | L | 4 | 1 |
| Contig226 | 162 | 0.995841139 | g or a | L | 2 | 1 |
| Contig230 | 137 | 0.999582692 | c or t | H | 7 | 2 |
| Contig230 | 330 | 0.995841861 | c or t | H | 5 | 2 |
| Contig25 | 309 | 0.999999925 | t or c | H | 4 | 2 |
| Contig253 | 91 | 0.995036637 | c or t | L | 1 | 4 |

| | | | | | | |
|---|---|---|---|---|---|---|
| Contig263 | 398 | 0.999058524 | a or g | L | 2 | 1 |
| Contig264 | 147 | 0.999622814 | g or a | L | 1 | 2 |
| Contig264 | 181 | 0.994692525 | a or g | L | 1 | 2 |
| Contig267 | 269 | 0.998808176 | t or g | L | 1 | 3 |
| Contig272 | 411 | 0.992898102 | t or a | H | 2 | 4 |
| Contig272 | 44 | 0.999823569 | t or c | L | 2 | 1 |
| Contig272 | 234 | 0.992578304 | a or g | L | 1 | 5 |
| Contig275 | 102 | 0.998015349 | c or t | H | 2 | 3 |
| Contig30 | 217 | 1 | t or c | H | 2 | 3 |
| Contig315 | 292 | 0.999668375 | c or a | L | 1 | 5 |
| Contig315 | 293 | 0.994462829 | t or g | L | 1 | 5 |
| Contig315 | 357 | 0.998242195 | g or a | L | 2 | 1 |
| Contig315 | 413 | 0.997025825 | c or t | L | 2 | 1 |
| Contig318 | 99 | 0.999791757 | t or a | H | 3 | 32 |
| Contig318 | 546 | 0.999930676 | a or g | H | 4 | 32 |
| Contig318 | 401 | 0.999999958 | g or t | L | 1 | 2 |
| Contig323 | 325 | 0.997025997 | t or c | L | 3 | 1 |
| Contig350 | 521 | 0.997949648 | g or a | H | 3 | 8 |
| Contig350 | 553 | 0.999998656 | a or t | H | 4 | 2 |
| Contig350 | 558 | 0.994964198 | g or a | H | 4 | 2 |
| Contig350 | 574 | 1 | g or a | H | 3 | 2 |
| Contig356 | 104 | 0.999999836 | c or t | H | 2 | 3 |
| Contig356 | 298 | 0.995843187 | g or a | H | 2 | 4 |
| Contig359 | 195 | 0.997122065 | t or c | L | 3 | 1 |
| Contig361 | 278 | 0.997743262 | a or g | L | 5 | 1 |
| Contig361 | 322 | 0.999557889 | g or a | L | 1 | 3 |
| Contig361 | 424 | 0.998808175 | t or c | L | 5 | 1 |
| Contig363 | 251 | 1 | g or a | H | 2 | 2 |
| Contig363 | 132 | 0.999999974 | t or a | L | 14 | 1 |
| Contig368 | 267 | 1 | g or a | L | 4 | 1 |
| Contig368 | 292 | 0.99586647 | a or g | L | 8 | 1 |
| Contig376 | 360 | 0.995841564 | c or t | L | 4 | 1 |
| Contig381 | 248 | 0.99812132 | g or a | L | 1 | 3 |
| Contig39 | 99 | 0.999999925 | t or c | L | 3 | 1 |
| Contig40 | 24 | 0.999996328 | t or g | H | 5 | 3 |
| Contig40 | 426 | 0.999999908 | c or g | H | 2 | 2 |
| Contig40 | 434 | 0.999999975 | g or a | H | 2 | 2 |
| Contig40 | 437 | 0.999999686 | g or a | H | 2 | 2 |
| Contig40 | 439 | 0.999999975 | t or g | H | 2 | 2 |
| Contig40 | 441 | 0.999999927 | g or t | H | 2 | 2 |
| Contig408 | 297 | 1 | g or c | H | 3 | 2 |
| Contig408 | 303 | 1 | a or g | H | 3 | 2 |
| Contig408 | 187 | 1 | c or t | L | 1 | 3 |
| Contig411 | 185 | 0.999999985 | t or c | L | 3 | 1 |
| Contig411 | 447 | 0.998025943 | c or t | L | 2 | 1 |
| Contig411 | 468 | 1 | a or t | L | 3 | 1 |
| Contig415 | 226 | 0.999999994 | g or a | L | 3 | 1 |
| Contig425 | 191 | 0.999999795 | a or g | H | 2 | 9 |
| Contig425 | 239 | 0.997446775 | g or a | H | 2 | 2 |
| Contig435 | 419 | 1 | c or a | H | 3 | 4 |
| Contig435 | 588 | 1 | t or c | H | 5 | 11 |
| Contig435 | 622 | 0.999999955 | c or t | H | 3 | 12 |
| Contig435 | 657 | 1 | t or c | H | 3 | 12 |
| Contig435 | 665 | 1 | a or g | H | 3 | 13 |
| Contig435 | 684 | 0.999999913 | a or g | H | 10 | 9 |
| Contig435 | 747 | 1 | a or g | H | 2 | 12 |
| Contig435 | 322 | 0.991130699 | g or a | L | 8 | 1 |
| Contig435 | 323 | 0.999274137 | a or c | L | 8 | 1 |
| Contig441 | 70 | 0.999215324 | a or g | L | 1 | 2 |
| Contig456 | 195 | 0.999842097 | c or g | L | 2 | 1 |
| Contig456 | 297 | 0.999905226 | c or t | L | 2 | 1 |
| Contig488 | 279 | 0.999957453 | a or t | H | 2 | 2 |
| Contig488 | 280 | 0.999997318 | c or a | H | 2 | 2 |
| Contig488 | 346 | 0.999998948 | c or a | H | 2 | 2 |
| Contig488 | 352 | 0.999999998 | g or a | H | 2 | 2 |

| | | | | | | |
|---|---|---|---|---|---|---|
| Contig488 | 430 | 0.996553143 | t or g | L | 2 | 1 |
| Contig488 | 444 | 0.995146774 | c or t | L | 1 | 2 |
| Contig53 | 250 | 0.998346902 | g or a | H | 3 | 2 |
| Contig53 | 66 | 1 | c or t | L | 4 | 1 |
| Contig575 | 52 | 0.999885307 | t or c | H | 7 | 2 |
| Contig575 | 53 | 0.999961459 | a or g | H | 7 | 2 |
| Contig575 | 103 | 1 | t or c | H | 7 | 2 |
| Contig575 | 119 | 0.999999999 | t or g | H | 5 | 8 |
| Contig575 | 132 | 0.999974692 | t or c | H | 6 | 3 |
| Contig575 | 153 | 1 | t or c | H | 7 | 2 |
| Contig575 | 194 | 0.999979158 | c or t | H | 5 | 2 |
| Contig575 | 214 | 0.999947628 | a or g | H | 6 | 2 |
| Contig575 | 218 | 0.995039335 | a or g | H | 6 | 2 |
| Contig575 | 275 | 0.999999977 | a or g | H | 6 | 2 |
| Contig575 | 277 | 1 | a or g | H | 6 | 3 |
| Contig576 | 270 | 0.999996382 | t or c | H | 2 | 2 |
| Contig594 | 381 | 0.999905226 | c or t | H | 3 | 3 |
| Contig594 | 265 | 1 | g or a | L | 3 | 1 |
| Contig597 | 76 | 1 | a or g | L | 1 | 3 |
| Contig605 | 11 | 0.999999998 | a or t | L | 2 | 1 |
| Contig605 | 201 | 0.991193462 | g or a | L | 2 | 1 |
| Contig609 | 329 | 1 | t or c | L | 1 | 2 |
| Contig609 | 413 | 0.999763101 | a or g | L | 1 | 5 |
| Contig616 | 400 | 0.998808176 | c or a | L | 2 | 1 |
| Contig636 | 81 | 1 | g or a | L | 3 | 1 |
| Contig636 | 83 | 0.999999995 | g or t | L | 2 | 1 |
| Contig636 | 84 | 0.999999995 | c or g | L | 2 | 1 |
| Contig636 | 85 | 1 | a or c | L | 2 | 1 |
| Contig636 | 120 | 0.991130104 | g or a | L | 2 | 1 |
| Contig636 | 218 | 0.999999937 | t or c | L | 8 | 1 |
| Contig636 | 454 | 0.999998427 | c or t | L | 2 | 1 |
| Contig636 | 461 | 0.999970837 | t or c | L | 2 | 1 |
| Contig647 | 223 | 0.998808178 | a or t | L | 2 | 1 |
| Contig647 | 225 | 0.992187203 | a or c | L | 2 | 1 |
| Contig647 | 231 | 0.999735927 | c or g | L | 2 | 1 |
| Contig66 | 119 | 0.997916347 | c or t | H | 2 | 5 |
| Contig66 | 418 | 1 | a or g | H | 2 | 6 |
| Contig66 | 464 | 0.999999792 | c or t | H | 3 | 4 |
| Contig66 | 601 | 0.999812528 | c or t | H | 3 | 3 |
| Contig680 | 102 | 0.999999995 | c or t | H | 2 | 3 |
| Contig680 | 215 | 0.999999498 | t or c | H | 8 | 3 |
| Contig680 | 238 | 1 | g or t | H | 8 | 2 |
| Contig680 | 297 | 0.999993898 | c or t | H | 6 | 81 |
| Contig680 | 304 | 0.999897142 | t or c | H | 10 | 2 |
| Contig680 | 317 | 0.999532067 | t or c | H | 2 | 84 |
| Contig680 | 403 | 0.999796321 | g or a | H | 2 | 81 |
| Contig680 | 430 | 1 | a or g | H | 8 | 2 |
| Contig680 | 465 | 0.99409078 | a or g | H | 9 | 9 |
| Contig680 | 560 | 0.995599191 | a or t | H | 6 | 3 |
| Contig680 | 624 | 0.999997302 | t or c | H | 6 | 2 |
| Contig680 | 717 | 0.992519793 | a or g | H | 2 | 9 |
| Contig680 | 892 | 1 | c or t | H | 5 | 3 |
| Contig680 | 916 | | a or t | H | 5 | 3 |
| Contig680 | 853 | 1 | a or g | L | 5 | 1 |
| Contig691 | 564 | 0.999905221 | a or g | L | 1 | 2 |
| Contig691 | 630 | 0.99955788 | c or a | L | 1 | 2 |
| Contig70 | 317 | 0.999431791 | a or g | H | 2 | 2 |
| Contig704 | 372 | 0.997751503 | t or c | H | 3 | 2 |
| Contig704 | 433 | 0.997382993 | g or c | L | 6 | 1 |
| Contig704 | 470 | 1 | g or a | L | 5 | 1 |
| Contig707 | 397 | 0.990203571 | g or a | L | 1 | 4 |
| Contig710 | 27 | 1 | a or t | L | 2 | 1 |
| Contig715 | 136 | 0.999905226 | t or c | L | 2 | 1 |
| Contig722 | 49 | 1 | a or t | H | 2 | 15 |
| Contig722 | 97 | 0.999939489 | g or a | H | 8 | 35 |

| | | | | | | |
|---|---|---|---|---|---|---|
| Contig722 | 182 | 1 | c or t | H | 4 | 35 |
| Contig722 | 200 | 0.999998227 | a or g | H | 6 | 35 |
| Contig722 | 209 | 0.999474674 | a or t | H | 4 | 34 |
| Contig722 | 228 | 0.999999991 | a or g | H | 2 | 40 |
| Contig722 | 266 | 0.999999983 | g or a | H | 3 | 39 |
| Contig722 | 382 | 0.994789057 | a or g | H | 2 | 35 |
| Contig722 | 392 | 0.999064042 | t or g | H | 65 | 4 |
| Contig722 | 393 | 1 | g or a | H | 3 | 35 |
| Contig722 | 394 | 0.999999109 | a or g | H | 65 | 4 |
| Contig722 | 397 | 1 | c or t | H | 3 | 35 |
| Contig722 | 400 | 0.999999923 | a or g | H | 4 | 31 |
| Contig722 | 415 | 0.999990564 | g or a | H | 2 | 34 |
| Contig722 | 466 | 0.999861579 | g or a | H | 59 | 5 |
| Contig722 | 495 | 0.999999998 | c or g | H | 4 | 26 |
| Contig722 | 501 | 1 | g or a | H | 4 | 23 |
| Contig722 | 504 | 1 | g or a | H | 4 | 22 |
| Contig722 | 506 | 1 | t or g | H | 4 | 22 |
| Contig722 | 508 | 1 | g or t | H | 4 | 21 |
| Contig722 | 385 | 1 | t or g | H | 3 | 36 |
| Contig722 | 75 | 0.99999787 | a or g | L | 1 | 23 |
| Contig722 | 85 | 0.999970527 | a or g | L | 1 | 32 |
| Contig722 | 498 | 1 | c or g | L | 2 | 1 |
| Contig724 | 410 | 0.999763148 | g or c | H | 3 | 2 |
| Contig733 | 444 | 0.994055103 | g or a | L | 1 | 2 |
| Contig733 | 447 | 0.9980173 | g or a | L | 1 | 2 |
| Contig735 | 441 | 0.997178435 | c or g | H | 2 | 2 |
| Contig735 | 727 | 0.998781923 | t or c | L | 2 | 1 |
| Contig735 | 878 | 0.999431795 | c or t | L | 2 | 1 |
| Contig735 | 1067 | 0.991130699 | a or g | L | 2 | 1 |
| Contig736 | 739 | 0.990131535 | c or t | L | 2 | 1 |
| Contig750 | 204 | 1 | t or c | H | 3 | 7 |
| Contig750 | 495 | 1 | c or g | H | 21 | 3 |
| Contig750 | 499 | 1 | g or c | H | 21 | 3 |
| Contig750 | 501 | 1 | c or a | H | 18 | 3 |
| Contig750 | 502 | 1 | g or c | H | 18 | 3 |
| Contig750 | 503 | 1 | g or c | H | 18 | 3 |
| Contig750 | 504 | 0.999289636 | c or a | H | 16 | 3 |
| Contig757 | 190 | 0.996644644 | t or c | L | 1 | 3 |
| Contig763 | 284 | 0.99955828 | c or t | H | 2 | 3 |
| Contig763 | 409 | 0.999999981 | a or g | L | 2 | 1 |
| Contig77 | 329 | 0.99876935 | c or t | H | 10 | 8 |
| Contig77 | 467 | 0.999290202 | t or g | L | 4 | 1 |
| Contig770 | 429 | 0.993750552 | c or t | H | 2 | 2 |
| Contig81 | 548 | 1 | t or c | H | 4 | 2 |
| Contig81 | 268 | 0.999289684 | g or a | L | 1 | 2 |
| Contig81 | 311 | 0.999971702 | c or t | L | 1 | 3 |
| Contig82 | 53 | 0.998808176 | c or t | L | 2 | 1 |
| Contig827 | 263 | 1 | a or g | L | 2 | 1 |
| Contig827 | 278 | 0.9999903 | c or t | L | 1 | 2 |
| Contig84 | 29 | 1 | t or a | H | 3 | 3 |
| Contig84 | 201 | 1 | a or g | H | 3 | 3 |
| Contig84 | 225 | 0.999999911 | g or a | H | 3 | 3 |
| Contig840 | 46 | 0.998958794 | a or g | H | 2 | 4 |
| Contig840 | 287 | 0.999558268 | c or t | H | 3 | 3 |
| Contig840 | 423 | 1 | g or t | H | 2 | 4 |
| Contig840 | 182 | 0.997742574 | g or a | L | 4 | 1 |
| Contig840 | 272 | 1 | t or c | L | 3 | 1 |
| Contig840 | 314 | 0.999999344 | a or g | L | 3 | 1 |
| Contig847 | 136 | 0.999621991 | t or c | L | 1 | 3 |
| Contig85 | 235 | 0.999843807 | g or a | L | 1 | 3 |
| Contig85 | 411 | 0.997025756 | a or g | L | 1 | 4 |
| Contig878 | 306 | 0.999842125 | g or a | L | 1 | 2 |
| Contig9 | 258 | 0.999622805 | t or c | L | 2 | 1 |
| Contig90 | 321 | 0.998816777 | a or t | H | 2 | 2 |
| Contig90 | 333 | 0.999905226 | t or c | H | 2 | 2 |

| | | | | | | |
|---|---|---|---|---|---|---|
| Contig906 | 195 | 0.999557884 | t or c | H | 2 | 3 |
| Contig908 | 278 | 0.994462366 | c or t | L | 1 | 2 |
| Contig91 | 392 | 0.997036978 | c or t | L | 3 | 1 |
| Contig936 | 83 | 0.999999807 | a or g | L | 1 | 3 |
| Contig962 | 230 | 0.996019723 | c or t | L | 1 | 21 |
| Contig968 | 193 | 0.9940833 | a or g | L | 1 | 2 |
| Contig98 | 148 | 0.999298462 | c or t | L | 8 | 1 |
| Contig98 | 331 | 1 | c or t | L | 3 | 1 |
| | | | | | | |
| a. Position of SNP refers to the site of SNP relative to the consensus sequence of the contig | | | | | | |
