# Supplementary material for: Comparative analyses of genotype dependent expressed sequence tags and stress-responsive transcriptome of chickpea wilt illustrate predicted and unexpected genes and novel regulators of plant immunity
Source: BMC Genomics. 2009 Sep 5;10:415. doi: 10.1186/1471-2164-10-415 (PMC2755012; doi:10.1186/1471-2164-10-415)
Supplement: Additional file 6 — Microarray analysis of CaESTs in resistant genotype. Table showing list of chickpea genes showing differential expression (upregulated and downregulated) in response to Fusarium infection at 24 hrs post inoculation during incompatible interaction. [file 1471-2164-10-415-S6.pdf]

**Additional file 6 - List of chickpea genes showing differential expression in response to *Fusarium* infection at 24hrs post inoculation during incompatible interaction**			
**Functional class**	**Clone ID[a]**	**Functional annotation[b]**	**Fold change [c]**
Cell cycle control and cell division	CaF1_JIE_25_B_10	putative kinetochore protein [Vicia faba var. minor]	2.6499524
	CaF1_WIE_26_B_02	Cell division protein FtsZ [Medicago truncatula]	-1.8213085
Cellular redox state	CaF1_JIE_10_H_08	glutathione S-transferase [Pisum sativum]	-1.7554958
	CaF1_JIE_16_E_10	cytochrome P450 [Cicer arietinum]	2.2396235
	CaF1_JIE_24_F_04	Cicer arietinum mRNA for cytochrome P450 (cyp93C3 gene)	3.381197
	CaF1_JIE_26_A_02	cytochrome p450 [Arabidopsis thaliana]	3.1926048
	CaF1_JIE_26_C_02	peroxidase [Glycine max]	1.9496702
	CaF1_JIE_28_B_04	cytochrome P450 [Cicer arietinum]	-1.9883561
	CaF1_JIE_29_B_03	cytochrome P450 monooxygenase CYP83G1 [Medicago truncatula]	3.461603
	CaF1_WIE_11_F_02	cytochrome P450 [Cicer arietinum]	1.3607495
	CaF1_WIE_13_H_05	thioredoxin h [Medicago sativa]	-2.7370684
	CaF1_WIE_19_E_01	cytochrome P450 monooxygenase [Cicer arietinum]	-1.3670851
	CaF1_WIE_26_H_11	cytochrome P450 [Cicer arietinum]	-1.8609934
	CaF1_WIE_29_A_07	peroxidase 1 precursor [Phaseolus vulgaris]	-2.5340927
	CaF1_WIE_30_B_01	superoxide dismutase [Cicer arietinum] emb|CAA10160.1| superoxide dismutase [Cicer arietinum]	-3.0009537
	CaF1_WIE_34_H_05	cytochrome P450 [Cicer arietinum]	-1.3469903
	CaF1_WIE_37_C_09	cytochrome P450 [Cicer arietinum]	-3.3505738
	CaF1_WIE_38_C_02	secretory peroxidase [Catharanthus roseus]	-2.4560368
	CaF1_WIE_38_F_02	Thioredoxin-related; Thioredoxin domain 2 [Medicago truncatula]	-3.0952504
	CaF1_WIE_38_G_09	Cicer arietinum mRNA for putative quinone oxidoreductase (qor gene)	-1.5214212
Cellular Transport/inorganic ion transport and metabolism	CaF1_JIE_26_F_04	GOS11 (GOLGI SNARE 11); SNARE binding [Arabidopsis thaliana] sp|Q9LMP7|GOS11_ARATH Golgi SNARE 11 protein (AtGOS11) (Golgi SNAP receptor complex member 1-1) gb|AAF82157.1|AC034256_21 Contains similarity to GOS28/P28 protein from Homo sapiens gb|AF047438. ESTs gb|F14225, gb|AA395297, gb|BE038320 come from this gene. [Arabidopsis thaliana] gb|AAG48789.1|AF332426_1 unknown protein [Arabidopsis thaliana] gb|AAK48904.1|AF357528_1 Golgi SNARE 11 protein [Arabidopsis thaliana] gb|AAK95249.1|AF410263_1 At1g15880/F7H2_20 [Arabidopsis thaliana] gb|AAM10347.1| At1g15880/F7H2_20 [Arabidopsis thaliana]	4.008237
	CaF1_JIE_27_C_09	Zinc finger, Sec23/Sec24-type; Sec23/Sec24 trunk region; Sec23/Sec24 helical region; Gelsolin region; Sec23/Sec24 beta-sandwich [Medicago truncatula]	2.6878982
	CaF1_WIE_37_A_11	integral membrane HRF1 family protein [Arabidopsis thaliana] ref|NP_001077633.1| integral membrane HRF1 family protein [Arabidopsis thaliana] gb|AAK64057.1| unknown protein [Arabidopsis thaliana] gb|AAM44952.1| unknown protein [Arabidopsis thaliana] gb|AAM63447.1| unknown [Arabidopsis thaliana]	-1.6932428
	CaF1_WIE_50_A_10	Mitochondrial import inner membrane translocase, subunit Tim17/22 [Medicago truncatula]	-1.9101465
	CaF1_WIE_50_E_05	selenium binding protein [Medicago sativa]	-1.9499032
	CaF1_WIE_55_B_05	Metallothionein-like protein 1 (MT-1) emb|CAA65008.1| metallothionein [Cicer arietinum]	-3.4266684
	CaF1_WIE_09_H_11	Intracellular chloride channel [Medicago truncatula]	-1.9668914
Defense mechanism	CaF1_JIE_22_B_09	non-specific lipid transfer-like protein	4.0182133
	CaF1_JIE_25_D_07	Plant lipid transfer/seed storage/trypsin-alpha amylase inhibitor [Medicago truncatula]	-1.6106298
	CaF1_JIE_27_C_01	Plant lipid transfer/seed storage/trypsin-alpha amylase inhibitor [Medicago truncatula]	1.5957952
	CaF1_JIE_42_A_11	class 10 pathogenesis-related protein [Lupinus luteus]	-2.124977
	CaF1_WIE_19_C_05	putative Bet v I family protein [Cicer arietinum]	-2.7545862
	CaF1_WIE_19_C_07	Disease resistance response protein DRRG49-C gb|AAA33663.1| disease resistance response protein (DRRG49-c)	1.809834
	CaF1_WIE_22_B_06	class I chitinase [Cicer arietinum]	-1.6433175
Development/storage/dormancy and senescence	CaF1_JIE_22_B_06	putative ripening related protein [Cicer arietinum]	-2.0621543

	CaF1_JIE_42_A_04	Albumin-2 (PA2) gb|AAA02981.1| albumin 2 gb|AAA33641.1| major seed albumin prf||1314296A albumin	-2.7018754
	CaF1_WIE_39_C_03	agglutinin [Amaranthus hypochondriacus] emb|CAA77664.1| seed specific protein of balanced nutritional quality [Amaranthus hypochondriacus]	-3.0905976
	CaF1_WIE_07_D_03	Albumin-2 (PA2) gb|AAA02981.1| albumin 2 gb|AAA33641.1| major seed albumin prf||1314296A albumin	1.7020338
	CaF1_WIE_09_E_10	germin-like protein [Cicer arietinum]	-1.8525877
Energy production and conversion	CaF1_JIE_26_C_09	H+-transporting two-sector ATPase, alpha/beta subunit, central region; H+-transporting two-sector ATPase, alpha/beta subunit, C-terminal [Medicago truncatula]	-1.5708983
	CaF1_JIE_33_E_02	putative ADP,ATP carrier-like protein [Trifolium pratense]	-1.5171654
	CaF1_WIE_33_H_10	vacuolar H+-ATPase subunit A [Vigna unguiculata]	1.4925897
Hypothetical protein	CaF1_JIE_10_B_10	hypothetical protein [Vitis vinifera]	3.349024
	CaF1_JIE_11_G_08	hypothetical protein [Vitis vinifera]	1.8081638
	CaF1_JIE_16_E_08	hypothetical protein FG03894.1 [Gibberella zeae PH-1]	3.747655
	CaF1_JIE_17_D_01	hypothetical protein MtrDRAFT_AC155898g5v1 [Medicago truncatula] gb|ABO83845.1| mitochondrial ATP synthase precursor, putative [Medicago truncatula]	2.0948586
	CaF1_JIE_18_A_09	hypothetical protein OsJ_006334 [Oryza sativa (japonica cultivar-group)]	3.0527568
	CaF1_JIE_18_G_09	Hypothetical protein [Oryza sativa]	3.9192092
	CaF1_JIE_18_H_09	hypothetical protein CaO19_11777 [Candida albicans SC5314] ref|XP_720608.1| hypothetical protein CaO19_4301 [Candida albicans SC5314] gb|EAL01773.1| hypothetical protein CaO19.4301 [Candida albicans SC5314] gb|EAL01907.1| hypothetical protein CaO19.11777 [Candida albicans SC5314]	4.3569336
	CaF1_JIE_19_F_09	hypothetical protein FG00681.1 [Gibberella zeae PH-1]	3.4991708
	CaF1_JIE_21_E_08	hypothetical protein [Vitis vinifera]	-1.7811298
	CaF1_JIE_23_A_11	hypothetical protein FG03168.1 [Gibberella zeae PH-1]	4.2875843
	CaF1_JIE_23_B_11	hypothetical protein [Vitis vinifera] emb|CAN65595.1| hypothetical protein [Vitis vinifera]	-3.0053186
	CaF1_JIE_24_G_05	hypothetical protein [Vitis vinifera]	-1.7787375
	CaF1_JIE_27_B_03	hypothetical protein FG00429.1 [Gibberella zeae PH-1]	3.1002305
	CaF1_JIE_28_A_11	hypothetical protein FG01158.1 [Gibberella zeae PH-1]	3.6839495
	CaF1_JIE_28_F_05	hypothetical protein [Vitis vinifera]	-1.6325347
	CaF1_JIE_28_G_09	hypothetical protein [Vitis vinifera] emb|CAN81281.1| hypothetical protein [Vitis vinifera]	-2.1359808
	CaF1_JIE_29_A_06	hypothetical protein [Vitis vinifera]	1.5978707
	CaF1_JIE_31_F_05	hypothetical protein [Vitis vinifera]	3.2638698
	CaF1_JIE_31_H_09	hypothetical protein FG10772.1 [Gibberella zeae PH-1]	-2.7946744
	CaF1_JIE_32_D_03	hypothetical protein MtrDRAFT_AC146866g9v2 [Medicago truncatula]	-1.583424
	CaF1_JIE_36_D_11	hypothetical protein [Vitis vinifera]	-1.7658386
	CaF1_JIE_37_D_07	hypothetical protein [Cucumis melo]	1.7110869
	CaF1_JIE_37_H_01	hypothetical protein [Vitis vinifera]	3.1456552
	CaF1_JIE_04_H_02	hypothetical protein [Capsicum chinense]	1.960922
	CaF1_JIE_09_D_06	hypothetical protein FG01008.1 [Gibberella zeae PH-1]	1.4545205
	CaF1_WIE_10_E_04	hypothetical protein OsJ_030893 [Oryza sativa (japonica cultivar-group)]	-2.503602
	CaF1_WIE_13_B_11	hypothetical protein [Vitis vinifera]	1.8265208
	CaF1_WIE_16_C_07	hypothetical protein CaO19.14167 [Candida albicans SC5314] ref|XP_712807.1| hypothetical protein CaO19.6878 [Candida albicans SC5314] gb|EAK93638.1| questionable orf [Candida albicans SC5314] gb|EAK93667.1| questionable orf [Candida albicans SC5314]	1.8619967
	CaF1_WIE_19_F_01	hypothetical protein [Vitis vinifera]	-1.5943851
	CaF1_WIE_22_A_09	hypothetical protein MtrDRAFT_AC139707g22v2 [Medicago truncatula]	2.7047126
	CaF1_WIE_26_H_03	hypothetical protein OsI_009223 [Oryza sativa (indica cultivar-group)]	-1.4990019
	CaF1_WIE_28_C_02	hypothetical protein [Vitis vinifera]	-2.341772
	CaF1_WIE_29_F_05	hypothetical protein [Arabidopsis thaliana]	2.8439627
	CaF1_WIE_30_A_11	hypothetical protein OsJ_008062 [Oryza sativa (japonica cultivar-group)]	2.506845
	CaF1_WIE_30_E_11	hypothetical protein [Vitis vinifera]	-2.267281
	CaF1_WIE_33_B_06	hypothetical protein [Vitis vinifera]	-2.4805522
	CaF1_WIE_37_H_04	hypothetical protein [Oryza sativa (japonica cultivar-group)]	-1.952332

	CaF1_WIE_38_G_03	hypothetical protein [Picea mariana]	-2.3017952
	CaF1_WIE_03_A_01	hypothetical protein [Vitis vinifera]	1.9258058
	CaF1_WIE_49_E_08	hypothetical protein [Vitis vinifera]	-2.924792
	CaF1_WIE_50_B_07	hypothetical protein [Vitis vinifera]	-2.2613893
	CaF1_WIE_50_C_11	hypothetical protein [Vitis vinifera]	1.7182666
	CaF1_WIE_54_F_08	hypothetical protein [Cicer arietinum]	-1.7703321
	CaF1_WIE_06_B_10	hypothetical protein [Vitis vinifera]	-1.3353833
	CaF1_WIE_07_F_02	hypothetical protein OsJ_026277 [Oryza sativa (japonica cultivar-group)]	-1.4917526
Metabolism	CaF1_JIE_10_D_03	adenine nucleotide translocator [Lupinus albus]	4.429408
	CaF1_JIE_18_C_04	Fructose-bisphosphate aldolase, cytoplasmic isozyme emb|CAA06308.1| cytosolic fructose-1,6-bisphosphate aldolase [Cicer arietinum]	1.395575
	CaF1_JIE_20_H_01	glyceraldehyde 3-phosphate dehydrogenase, cytosolic [Cicer arietinum]	-1.709953
	CaF1_JIE_23_F_10	triosephosphate isomerase [Glycine max]	-1.7474434
	CaF1_JIE_25_D_08	Pyridoxal-5-phosphate-dependent enzyme, beta subunit [Medicago truncatula]	3.2544267
	CaF1_JIE_26_B_02	oleate desaturase [Caragana korshinskii var. intermedia]	3.2350082
	CaF1_JIE_27_F_08	Triose phosphate/phosphate translocator, chloroplast precursor (cTPT) (p36) (E30) emb|CAA38451.1| chloroplast import receptor p36 [Pisum sativum] emb|CAA48210.1| phosphate translocator [Pisum sativum] prf||1805409A phosphate translocator	3.2534337
	CaF1_JIE_37_C_04	Probable pyridoxal biosynthesis protein PDX1 (Sor-like protein) gb|AAK18310.1|AF344827_1 Sor-like protein [Ginkgo biloba]	-3.1873424
	CaF1_JIE_37_G_02	Alcohol dehydrogenase 1 emb|CAA29609.1| alcohol dehydrogenase [Pisum sativum]	2.6967018
	CaF1_JIE_06_D_01	pfkB-type carbohydrate kinase family protein [Arabidopsis thaliana] gb|AAF79436.1|AC025808_18 F18O14.35 [Arabidopsis thaliana] gb|AAF98405.1|AC024609_6 Unknown protein [Arabidopsis thaliana] gb|AAO44087.1| At1g19600 [Arabidopsis thaliana] dbj|BAE99744.1| putative ribokinase [Arabidopsis thaliana]	2.4469779
	CaF1_WIE_10_C_03	methionine sulfoxide reductase A [Populus trichocarpa x Populus deltoides]	1.5684775
	CaF1_WIE_15_C_06	F25A4.24 [Arabidopsis thaliana]	2.328292
	CaF1_WIE_16_F_11	C2; Peptidase, cysteine peptidase active site [Medicago truncatula]	-1.4686471
	CaF1_WIE_16_H_10	S-adenosylmethionine synthetase [Medicago truncatula]	-2.9682486
	CaF1_WIE_16_H_04	Glutamine synthetase nodule isozyme (Glutamate--ammonia ligase) (GS) gb|AAA34239.1| glutamine synthetase prf||2106409A Gln synthetase	-2.5428405
	CaF1_WIE_17_C_08	polygalacturonase-like protein [Fragaria x ananassa]	2.0523713
	CaF1_WIE_21_G_03	putative His-Asp phosphotransfer protein [Pisum sativum]	1.3294389
	CaF1_WIE_22_G_06	Non-symbiotic hemoglobin 1 (MEDsa GLB1) gb|AAG29748.1|AF172172_1 non-symbiotic hemoglobin [Medicago sativa]	-1.7638232
	CaF1_WIE_28_A_07	S-adenosylmethionine synthetase 2 (Methionine adenosyltransferase 2) (AdoMet synthetase 2) emb|CAA57581.1| methionine adenosyltransferase [Pisum sativum] gb|AAA58773.1| S-adenosylmethionine synthase	-2.672482
	CaF1_WIE_34_C_04	proline dehydrogenase [Medicago sativa] gb|AAT45085.1| proline dehydrogenase [Medicago sativa]	2.297549
	CaF1_WIE_35_B_08	CXE carboxylesterase [Actinidia deliciosa]	-1.4484546
	CaF1_WIE_45_E_05	5-methyltetrahydropteroyltriglutamate--homocysteine S-methyltransferase; Prismane-like [Medicago truncatula] gb|ABE81639.2| 5-methyltetrahydropteroyltriglutamate--homocysteine S-methyltransferase; Prismane-like [Medicago truncatula]	-1.7037696
	CaF1_WIE_48_G_10	S-adenosyl-L-methionine synthetase [Dendrobium crumenatum]	-4.3793335
	CaF1_WIE_49_F_08	Malic oxidoreductase [Medicago truncatula]	-1.6109502
	CaF1_WIE_50_B_10	NAD-dependent malate dehydrogenase [Prunus persica]	-2.5581868
	CaF1_WIE_51_G_04	Glyceraldehyde-3-phosphate dehydrogenase, cytosolic	-1.5330108

	CaF1_WIE_52_D_06	S-adenosylmethionine decarboxylase proenzyme (AdoMetDC) (SamDC) [Contains: S-adenosylmethionine decarboxylase alpha chain; S-adenosylmethionine decarboxylase beta chain] emb|CAB76966.1| S-adenosylmethionine decarboxylase [Vicia faba]	-2.8252633
	CaF1_WIE_52_E_06	glyceraldehyde-3-phosphate dehydrogenase [Populus maximowiczii x Populus nigra]	-1.6146357
	CaF1_WIE_52_H_07	malate dehydrogenase precursor [Medicago sativa]	-2.216991
	CaF1_WIE_56_F_11	Chain X, Crystal Structure Of Vestitone Reductase From Alfalfa (Medicago Sativa L.)	2.7259107
	CaF1_WIE_07_C_11	putative dTDP-glucose 4-6-dehydratase [Arabidopsis thaliana]	-3.120588
Miscellaneous	CaF1_JIE_12_D_11	Fusarium oxysporum f. sp. lycopersici six1 gene, fot5 gene, six2 gene, shh1 gene and ORF2 (partial)	4.476898
	CaF1_JIE_13_F_06	Fusarium oxysporum f. sp. vasinfectum strain X515-II Foxy transposable element, partial sequence	4.57299
	CaF1_JIE_17_E_01	Fusarium oxysporum f. sp. lycopersici six1 gene, fot5 gene, six2 gene, shh1 gene and ORF2 (partial)	2.6930747
	CaF1_JIE_18_F_03	F-actin capping protein, alpha subunit [Medicago truncatula]	4.039521
	CaF1_JIE_18_G_05	Fusarium oxysporum f. sp. vasinfectum strain Ag149-I Foxy transposable element, partial sequence	3.5210824
	CaF1_JIE_20_C_11	Fusarium oxysporum f. sp. lycopersici insertion sequence Foxy	4.4632626
	CaF1_JIE_28_E_10	Solanum lycopersicum cDNA, clone: LEFL2005N13, HTC in fruit	2.7017455
	CaF1_JIE_38_A_02	Os05g0103600 [Oryza sativa (japonica cultivar-group)] gb|AAS88829.1| putative ankyrin protein [Oryza sativa (japonica cultivar-group)] dbj|BAF16310.1| Os05g0103600 [Oryza sativa (japonica cultivar-group)]	-2.6423128
	CaF1_JIE_38_G_08	putative transposase [Tolypocladium inflatum]	2.2806792
	CaF1_JIE_39_H_05	Nascent polypeptide-associated complex NAC [Medicago truncatula]	1.6941837
	CaF1_JIE_40_B_01	Fusarium oxysporum f. sp. lycopersici six1 gene, fot5 gene, six2 gene, shh1 gene and ORF2 (partial)	3.944921
	CaF1_JIE_07_C_06	Medicago truncatula chromosome 7 BAC clone mth2-51a12, complete sequence	2.3896542
	CaF1_WIE_11_C_10	Fusarium oxysporum f. sp. lycopersici insertion sequence Foxy	3.1880765
	CaF1_WIE_12_H_01	specific tissue protein 1 [Cicer arietinum]	-1.8219059
	CaF1_WIE_13_H_06	Dimerisation [Medicago truncatula]	1.8083761
	CaF1_WIE_16_H_05	Medicago truncatula clone mth2-30j23, complete sequence	-1.3234448
	CaF1_WIE_22_H_07	ZIM [Medicago truncatula]	-2.0779278
	CaF1_WIE_29_H_06	Medicago truncatula clone mth2-15j20, complete sequence	2.006845
	CaF1_WIE_35_B_03	Medicago truncatula clone mth2-18h17, complete sequence	-1.5256133
	CaF1_WIE_38_B_08	Nerium oleander microsatellite CATR25 sequence	-1.9346721
	CaF1_WIE_39_F_09	Catharanthus roseus clone CrP15 T-DNA sequence	2.7109427
	CaF1_WIE_39_G_04	Glycine max clone gmw1-105h23, complete sequence	-2.0859592
	CaF1_WIE_40_G_03	ARG10 [Vigna radiata]	-1.7371304
	CaF1_WIE_46_F_03	Medicago truncatula clone mth2-53h4, complete sequence	-2.143268
	CaF1_WIE_51_F_08	Translationally-controlled tumor protein homolog (TCTP) gb|AAD10032.1| translationally controlled tumor protein [Hevea brasiliensis]	-2.919891
	CaF1_WIE_52_A_11	Lotus japonicus genomic DNA, chromosome 2, clone:LjT10B11, TM0008, complete sequence	-1.4487596
	CaF1_WIE_52_D_07	Fusarium oxysporum f. sp. vasinfectum strain X515-II Foxy transposable element, partial sequence	2.1134303
	CaF1_WIE_09_A_07	Medicago truncatula clone mth2-30b20, complete sequence	-2.2580106
	CaF1_WIE_09_H_09	Fusarium oxysporum f. sp. lycopersici six1 gene, fot5 gene, six2 gene, shh1 gene and ORF2 (partial)	3.2260544
No significant homology	CaF1_JIE_12_C_01	Eristalis tenax partial mRNA for hypothetical protein (ORF1), isolate 3	1.9321637
	CaF1_JIE_19_C_11	Aspergillus niger CBS 513.88 contig An17c0060, complete genome emb|AM270388.1| Aspergillus niger contig An17c0060, complete genome	1.4543701
	CaF1_JIE_20_F_02	Plasmodiophora brassicae 16S ribosomal RNA gene, partial sequence; mitochondrial gene for mitochondrial product	-1.521175
	CaF1_JIE_24_E_01	Synthetic construct RLS (RLS) gene, complete cds	-1.5735109
	CaF1_JIE_26_B_04	Fusarium oxysporum f. sp. lycopersici insertion sequence Foxy	3.8522556
	CaF1_JIE_26_E_11	Synthetic construct RLS (RLS) gene, complete cds	1.3411266
	CaF1_JIE_27_D_04	Platynereis dumerilii mRNA for hypothetical protein (ORF1), isolate 5	2.8255823
	CaF1_JIE_35_H_11	Poplar cDNA sequences	-1.6188391

	CaF1_JIE_37_E_04	Triticum aestivum clone wlsu2.pk0001.h3:fis, full insert mRNA sequence	-2.2904372
	CaF1_JIE_37_G_03	Poplar cDNA sequences	3.1770217
	CaF1_JIE_39_G_10	Mouse DNA sequence from clone RP23-193M23 on chromosome 4, complete sequence	2.7961936
	CaF1_WIE_10_A_06	Photobacterium damselae subsp. piscicida trpA gene for putative transposase and partial ORF1 DNA for hypothetical protein, clone pRDA14	-2.32272
	CaF1_WIE_10_G_05	gigas BAT1 homolog mRNA, complete cds	3.6682801
	CaF1_WIE_13_G_09	Medicago truncatula clone mth2-11d24, complete sequence	1.5342212
	CaF1_WIE_14_B_11	Medicago truncatula chromosome 5 clone mth2-27a1, COMPLETE SEQUENCE	1.3577583
	CaF1_WIE_14_F_03	Vigna unguiculata partial mRNA for putative single-stranded nucleic acid binding R3H (MtrDRAFT_AC183371g11v1 gene), clone 55	-1.7718854
	CaF1_WIE_19_H_04	Photobacterium damselae subsp. piscicida trpB gene for putative transposase, clone pRDA21	-1.8637674
	CaF1_WIE_24_G_01	Poplar cDNA sequences	-1.9014621
	CaF1_WIE_27_D_02	Trichomonas vaginalis G3 variable membrane protein precursor, putative (TVAG_087500) mRNA, complete cds	-1.5804558
	CaF1_WIE_27_D_07	Zebrafish DNA sequence from clone DKEY-177P5 in linkage group 14, complete sequence	-2.164645
	CaF1_WIE_38_C_05	Rattus norvegicus obese protein gene, 5' flanking region and partial cds	-1.5439398
	CaF1_WIE_39_B_09	Medicago truncatula clone mth2-139i23, complete sequence	2.2461684
	CaF1_WIE_39_D_08	Rattus norvegicus obese protein gene, 5' flanking region and partial cds	1.5130666
	CaF1_WIE_50_G_09	Shewanella denitrificans OS217, complete genome	-1.5409979
	CaF1_WIE_51_E_01	Synthetic construct RLS (RLS) gene, complete cds	1.5241569
	CaF1_WIE_07_F_04	Medicago truncatula clone mth2-6k4, complete sequence	-2.0686364
	CaF1_WIE_08_E_09	Medicago truncatula clone mth2-64j6, complete sequence	-1.325752
	CaF1_WIE_09_D_10	Pisum sativum ent-kaurene oxidase (LH) mRNA, complete cds	-1.6395019
Nucleotide binding proteins	CaF1_JIE_25_F_04	GTP-binding protein [Capsicum annuum]	3.9421172
	CaF1_WIE_29_B_02	Ras small GTPase, Rab type [Medicago truncatula]	-1.9962513
	CaF1_WIE_07_D_10	RabGAP/TBC [Medicago truncatula] gb|ABO83596.1| RabGAP/TBC [Medicago truncatula]	1.588139
Post translational modification, protein turn over and chaperons	CaF1_JIE_12_A_01	Oligosaccharyl transferase, STT3 subunit [Medicago truncatula] gb|ABO83793.1| Oligosaccharyl transferase, STT3 subunit [Medicago truncatula]	4.251155
	CaF1_JIE_17_E_04	cystatin [Medicago sativa]	3.1015394
	CaF1_JIE_21_H_10	Sec61beta [Medicago truncatula]	3.620227
	CaF1_JIE_26_G_10	Proteinase inhibitor I25, cystatin [Medicago truncatula]	1.6123501
	CaF1_JIE_37_D_03	PsHSC71.0 [Pisum sativum]	2.7307675
	CaF1_JIE_39_E_02	Heat shock protein DnaJ [Medicago truncatula] gb|ABE83817.1| Heat shock protein DnaJ [Medicago truncatula] gb|ABP02364.1| Heat shock protein DnaJ [Medicago truncatula]	-1.7368543
	CaF1_WIE_11_A_06	F20B17.14 [Arabidopsis thaliana] gb|AAG52249.1|AC011717_17 putative aspartyl protease; 105611-106921 [Arabidopsis thaliana]	-1.9655722
	CaF1_WIE_18_F_03	putative ubiquitin-conjugating enzyme [Artemisia annua]	-2.2143378
	CaF1_WIE_28_C_06	ubiquitin [Antirrhinum majus]	-1.8636589
	CaF1_WIE_41_A_08	QM family protein [Caragana jubata]	-1.5502679
	CaF1_WIE_50_F_06	protein disufide isomerase-like protein [Glycine max]	-2.6089149
	CaF1_WIE_51_F_01	ubiquitin extension protein [Capsicum annuum]	3.3871148
	CaF1_WIE_55_D_08	cysteine proteinase [Cicer arietinum]	-1.5178095
Replication, recombination and repair	CaF1_JIE_28_E_08	putative helicase [Oryza sativa (japonica cultivar-group)] gb|AAP54108.1| AT hook motif-containing protein, putative [Oryza sativa (japonica cultivar-group)]	2.9626129
RNA processing and modification	CaF1_JIE_16_F_02	DEAD box RNA helicase [Pisum sativum] gb|AAR97917.1| DEAD box RNA helicase [Pisum sativum]	-1.3797443
	CaF1_JIE_19_B_01	DEAD box RNA helicase [Pisum sativum] gb|AAR97917.1| DEAD box RNA helicase [Pisum sativum]	1.3288932
	CaF1_WIE_27_F_10	RNA-binding region RNP-1 (RNA recognition motif) [Medicago truncatula]	-3.5912557
	CaF1_WIE_31_B_11	RNA-binding region RNP-1 (RNA recognition motif) [Medicago truncatula]	-1.6504152

Secondary metabolism	CaF1_JIE_24_C_03	squalene epoxidase [Medicago sativa]	2.2767234
	CaF1_JIE_27_B_02	putative sterol-C-methyltransferase [Arabidopsis thaliana]	-1.9982643
	CaF1_JIE_27_C_08	Chalcone--flavonone isomerase 1 (Chalcone isomerase 1) pdb|1EYP|A Chain A, Chalcone Isomerase pdb|1EYP|B Chain B, Chalcone Isomerase pdb|1EYQ|A Chain A, Chalcone Isomerase And Naringenin pdb|1EYQ|B Chain B, Chalcone Isomerase And Naringenin pdb|1JEP|A Chain A, Chalcone Isomerase Complexed With 4'-Hydroxyflavanone pdb|1JEP|B Chain B, Chalcone Isomerase Complexed With 4'-Hydroxyflavanone pdb|1FM8|A Chain A, Chalcone Isomerase Complexed With 5,4'-Dideoxyflavanone pdb|1FM8|B Chain B, Chalcone Isomerase Complexed With 5,4'-Dideoxyflavanone pdb|1FM7|A Chain A, Chalcone Isomerase Complexed With 5-Deoxyflavanone pdb|1FM7|B Chain B, Chalcone Isomerase Complexed With 5-Deoxyflavanone gb|AAB41524.1| chalcone isomerase [Medicago sativa]	4.281432
	CaF1_JIE_28_D_04	chalcone reductase [Cicer arietinum]	2.8933275
	CaF1_JIE_35_A_02	squalene epoxidase [Medicago sativa]	2.8060534
	CaF1_WIE_10_B_09	Trans-cinnamate 4-monooxygenase (Cinnamic acid 4-hydroxylase) (CA4H) (C4H) (P450C4H) (Cytochrome P450 73) dbj|BAA13414.1|	-1.9162018
	CaF1_WIE_33_A_10	chalcone synthase [Cicer arietinum]	-1.9705621
	CaF1_WIE_51_G_01	isoflavone 3'-hydroxylase [Medicago truncatula]	-1.9426444
Signaling	CaF1_JIE_20_G_02	14-3-3-like protein B (VFA-1433B) emb|CAA88416.1| 14-3-3 brain protein homolog [Vicia faba]	-1.9031892
	CaF1_JIE_26_H_11	leucine-rich repeat transmembrane protein kinase, putative [Arabidopsis thaliana] dbj|BAA96896.1| receptor-like protein kinase [Arabidopsis thaliana]	2.8978066
	CaF1_JIE_35_G_11	Calmodulin (CaM)	-2.4961448
	CaF1_JIE_39_F_05	serine/threonine/tyrosine kinase [Arachis hypogaea]	2.8402255
	CaF1_JIE_42_A_07	Serine/threonine protein kinase, active site [Medicago truncatula] gb|ABE89881.1| Serine/threonine protein kinase, active site [Medicago truncatula]	2.420997
	CaF1_JIE_08_B_09	putative protein kinase APK1A [Trifolium pratense]	1.7424483
	CaF1_WIE_26_E_06	serine/threonine kinase [Arabidopsis thaliana]	2.9525948
	CaF1_WIE_30_H_01	Protein kinase; GroEL-like chaperone, ATPase [Medicago truncatula]	-1.4742881
	CaF1_WIE_33_G_10	type 2A protein phosphatase-3 [Vicia faba]	-1.7404718
Stress	CaF1_JIE_39_B_10	Universal stress protein (Usp) [Medicago truncatula]	1.4538505
	CaF1_WIE_19_A_03	putative imbibition protein [Cicer arietinum]	-2.169288
	CaF1_WIE_27_B_07	Universal stress protein (Usp) [Medicago truncatula] gb|ABE88281.1| Universal stress protein (Usp) [Medicago truncatula]	-1.7875396
	CaF1_WIE_04_A_11	aquaporin protein PIP1;1 [Medicago truncatula]	-1.7399144
Transcription	CaF1_JIE_12_B_03	BEL1-like homeodomain transcription factor [Trifolium pratense]	-1.5660806
	CaF1_JIE_32_G_05	zinc finger (ZPR1-type) family protein [Arabidopsis thaliana]	-1.573273
	CaF1_WIE_18_H_03	HMG-protein [Plantago major]	-1.9353588
	CaF1_WIE_31_E_01	SAR DNA-binding protein-1 [Pisum sativum]	-1.8757493
	CaF1_WIE_31_F_11	Zinc finger, ZZ-type; Zinc finger, C2H2-type [Medicago truncatula]	-1.3666931
	CaF1_WIE_34_D_01	110 kDa 4SNc-Tudor domain protein [Pisum sativum]	-1.5988247
	CaF1_WIE_41_H_07	Nucleic acid-binding, OB-fold, subgroup [Medicago truncatula]	-2.1592114
	CaF1_WIE_07_H_01	putative DNA binding protein [Trifolium pratense]	-2.7320666
Translation, ribosomal structure and biogenesis	CaF1_JIE_10_B_07	S25 ribosomal protein [Medicago truncatula] gb|ABE85967.1| S25 ribosomal protein [Medicago truncatula]	-4.2116227
	CaF1_JIE_10_B_08	ribosomal protein L42	-3.1429372
	CaF1_JIE_16_C_11	Ribosomal protein S4, bacterial and organelle form [Medicago truncatula]	-2.512944
	CaF1_JIE_18_H_02	Translation factor [Medicago truncatula]	-1.6090437
	CaF1_JIE_20_D_04	Translation protein SH3-like [Medicago truncatula]	-1.3560902
	CaF1_JIE_23_E_04	Ribosomal protein L7Ae/L30e/S12e/Gadd45 [Medicago truncatula]	-2.261133
	CaF1_JIE_24_F_08	Translation factor [Medicago truncatula]	-1.3947332
	CaF1_JIE_27_F_01	40S ribosomal protein S13 gb|AAS47510.1| ribosomal protein S13 [Glycine max]	3.5183063
	CaF1_WIE_13_D_05	40S ribosomal protein S8 gb|AAC24583.1| 40S ribosomal protein S8 [Prunus armeniaca]	-1.968343

	CaF1_WIE_14_G_01	Glycine max mRNA for ribosomal protein L2 (rpL2 gene)	-1.7430153
	CaF1_WIE_16_C_10	40S ribosomal protein S19 [Cicer arietinum]	-2.1084013
	CaF1_WIE_16_D_09	elongation factor 1-alpha (EF1-a) [Cicer arietinum]	-1.3417867
	CaF1_WIE_22_A_08	Translation protein SH3-like [Medicago truncatula]	2.6027722
	CaF1_WIE_26_D_09	ribosomal protein S6 [Glycine max]	-2.8412929
	CaF1_WIE_27_C_01	ribosomal protein S3a [Cicer arietinum]	-1.6394892
	CaF1_WIE_29_A_01	putative senescence-associated protein [Pisum sativum]	1.5737662
	CaF1_WIE_31_B_01	60S ribosomal protein [Medicago sativa]	-2.3020163
	CaF1_WIE_33_C_05	Ribosomal L23 protein; Ribosomal protein L23, N-terminal [Medicago truncatula]	-3.9396408
	CaF1_WIE_38_C_08	Pseudomonas aeruginosa gene for 16S rRNA, partial sequence, strain: Hg2	2.180675
	CaF1_WIE_39_H_06	Eukaryotic translation initiation factor 5A (eIF-5A) emb|CAB65463.1| translation initiation factor 5A precursor protein (eIF-5A) [Senecio vernalis]	-1.7927139
	CaF1_WIE_45_D_05	60S ribosomal protein L37a [Capsicum chinense]	-3.1700969
	CaF1_WIE_46_D_03	Ribosomal L18ae protein [Medicago truncatula]	-1.3736346
	CaF1_WIE_04_F_11	ribosomal Pr 117 [Triticum aestivum]	-1.691469
	CaF1_WIE_50_C_06	Translation factor; Elongation factor G, III and V [Medicago truncatula]	-2.6527877
	CaF1_WIE_50_D_08	Ribosomal protein L10E [Medicago truncatula]	-2.7410398
	CaF1_WIE_52_C_11	40S ribosomal protein S7-like protein [Solanum tuberosum] gb|ABA46775.1| unknown [Solanum tuberosum] gb|ABB17004.1| ribosomal protein S7-like protein [Solanum tuberosum] gb|ABB87101.1| 40S ribosomal protein S7-like protein-like [Solanum tuberosum]	-1.4858263
	CaF1_WIE_53_A_10	40S ribosomal protein S5 emb|CAA06491.1| 40S ribosomal protein S5 [Cicer arietinum]	-1.4658971
	CaF1_WIE_53_G_02	Probable 60 ribosomal protein L14 (Hydroxyproline-rich glycoprotein HRGP1)	-3.5085804
	CaF1_WIE_07_H_10	Ribosomal protein S13 [Medicago truncatula] gb|ABE81211.1| Ribosomal protein S13 [Medicago truncatula]	1.7094357
Unknown function	CaF1_JIE_23_D_01	unknown [Solanum tuberosum] gb|ABB02647.1| unknown [Solanum tuberosum]	-3.2177794
	CaF1_JIE_38_H_11	unknown protein [Arabidopsis thaliana] gb|AAK44146.1|AF370331_1 unknown protein [Arabidopsis thaliana] gb|AAN13152.1| unknown protein [Arabidopsis thaliana]	1.4721597
	CaF1_WIE_13_E_10	unknown protein [Arabidopsis thaliana] gb|AAG50831.1|AC074395_5 unknown protein, 5' partial [Arabidopsis thaliana]	-2.5149403

a. In the clone ID, the first two letters (Ca) signify the source plant, *Cicer arietinum* , the third letter (F) designates the pathogen name, *Fusarium* , and the numeral 1 designates race 1 of *Fusarium* . In the three letter abbreviation JIE and WIE, the first letter J and W refers to the JG-62 and WR-315 genotypes of chickpea, followed by (IE) signifying *Fusarium* induced library and the early time points taken for the tissue collection. For additional details, see Materials and Methods.

b. The possible function of the chickpea sequences was assigned by performing BLASTX and BLASTN against nonredundant protein and nucleotide database in NCBI.

c. The values signify the log2 transformed fold change
